# Spatio-temporal modeling of particulate air pollution in the conterminous United States using geographic and meteorological predictors

**DOI:** 10.1186/1476-069X-13-63

**Published:** 2014-08-05

**Authors:** Jeff D Yanosky, Christopher J Paciorek, Francine Laden, Jaime E Hart, Robin C Puett, Duanping Liao, Helen H Suh

**Affiliations:** 1 Department of Public Health Sciences, The Pennsylvania State University College of Medicine, Hershey, PA, USA; 2 Department of Statistics, University of California, Berkeley, CA, USA; 3 Exposure, Epidemiology, and Risk Program, Department of Environmental Health, Harvard School of Public Health, Boston, MA, USA; 4 Channing Division of Network Medicine, Department of Medicine, Brigham and Women’s Hospital and Harvard Medical School, Boston, MA, USA; 5 Maryland Institute of Applied Environmental Health, University of Maryland School of Public Health, College Park, MD, USA; 6 Department of Health Sciences, Bouve College of Health Sciences, Northeastern University, Boston, MA, USA

**Keywords:** Particulate matter, Spatio-temporal models, Land use regression, Spatial smoothing, Penalized splines, Generalized additive mixed model

## Abstract

**Background:**

Exposure to atmospheric particulate matter (PM) remains an important public health concern, although it remains difficult to quantify accurately across large geographic areas with sufficiently high spatial resolution. Recent epidemiologic analyses have demonstrated the importance of spatially- and temporally-resolved exposure estimates, which show larger PM-mediated health effects as compared to nearest monitor or county-specific ambient concentrations.

**Methods:**

We developed generalized additive mixed models that describe regional and small-scale spatial and temporal gradients (and corresponding uncertainties) in monthly mass concentrations of fine (PM_2.5_), inhalable (PM_10_), and coarse mode particle mass (PM_2.5–10_) for the conterminous United States (U.S.). These models expand our previously developed models for the Northeastern and Midwestern U.S. by virtue of their larger spatial domain, their inclusion of an additional 5 years of PM data to develop predictions through 2007, and their use of refined geographic covariates for population density and point-source PM emissions. Covariate selection and model validation were performed using 10-fold cross-validation (CV).

**Results:**

The PM_2.5_ models had high predictive accuracy (CV R^2^=0.77 for both 1988–1998 and 1999–2007). While model performance remained strong, the predictive ability of models for PM_10_ (CV R^2^=0.58 for both 1988–1998 and 1999–2007) and PM_2.5–10_ (CV R^2^=0.46 and 0.52 for 1988–1998 and 1999–2007, respectively) was somewhat lower. Regional variation was found in the effects of geographic and meteorological covariates. Models generally performed well in both urban and rural areas and across seasons, though predictive performance varied somewhat by region (CV R^2^=0.81, 0.81, 0.83, 0.72, 0.69, 0.50, and 0.60 for the Northeast, Midwest, Southeast, Southcentral, Southwest, Northwest, and Central Plains regions, respectively, for PM_2.5_ from 1999–2007).

**Conclusions:**

Our models provide estimates of monthly-average outdoor concentrations of PM_2.5_, PM_10_, and PM_2.5–10_ with high spatial resolution and low bias. Thus, these models are suitable for estimating chronic exposures of populations living in the conterminous U.S. from 1988 to 2007.

## Background

Understanding the health impacts resulting from exposure to atmospheric particulate matter (PM) air pollution remains a priority for environmental public health. The physical and chemical characteristics of PM affect its relevance to human health, as demonstrated by the observed differences in behavior, composition, and health impacts for fine (PM<2.5 μm in aerodynamic diameter: PM_2.5_) and coarse (2.5<=PM<10 μm in aerodynamic diameter: PM_2.5–10_) particles [1-3]. These differences make it important to examine the health effects of PM using exposure assessment methods able to capture variation in the levels of each PM size fraction across the spatial and temporal scales relevant to health outcomes, especially when studies are conducted over large geographic areas. Traditionally, however, epidemiologic studies of the chronic health effects of PM air pollution have used crude methods to assess particulate exposures, estimating subject’s chronic exposure either by imputing ambient concentrations from the nearest monitor or by using area-wide averages [2], thus ignoring within-city spatial gradients in air pollutant levels and restricting these studies to areas with nearby monitoring data.

To avoid these limitations, more sophisticated methods to assess long-term air pollution exposures have been recently developed that provide location-specific (*e.g.*, at a residence) information on exposure and that can be applied to large populations living across large geographic areas [4-16]. Many of these studies [4-8,11-15] used location-specific geographic characteristics such as population density or the proximity of roadways to describe small-scale spatial variations in air pollutant levels (*i.e.*, land use regression (LUR)). Others have used spatial modeling of long-term averages or time-period-specific levels alone [9,10] or in combination with LUR [6,16]. Additionally, spatio-temporal modeling methods have also been developed which include LUR covariates [14,15] to model air pollutant levels at unmeasured locations. In one such application, McMillan et al. [17] incorporate the output of a deterministic Eulerian atmospheric chemistry and transport simulation model (the U.S. Environmental Protection Agency’s Community Multi-scale Air Quality model) in a spatio-temporal model using Bayesian fitting methods. Also, spatio-temporal models have included observations of satellite–based aerosol optical depth (AOD) [18-24] to predict PM concentrations over both small [18,19] and large spatial domains [23,24], with mixed results.

In our previous work, we developed and validated spatio-temporal generalized additive mixed models (GAMMs) of outdoor PM_2.5_ and PM_10_ levels for the Northeastern and Midwestern U.S. that included geographic information system (GIS)-based time-invariant spatial covariates and time-varying covariates such as meteorological data [11-13]. We showed that PM_2.5_, PM_10_, and PM_2.5–10_ levels were estimated with a high degree of accuracy (predicted values did not display bias, on average, in comparison with measured values) and precision (predicted values were strongly correlated with observed values) and that the models were able to account for both within- and between-city variation in PM concentrations. When our models were used to assess chronic PM exposures in epidemiological studies, we found higher health risks than when simpler exposure assessment approaches were used [11,25,26], likely due to the models’ ability to reduce exposure error by estimation of within-city variability in PM levels, specifically in traffic-related PM.

Our previous GIS-based spatio-temporal exposure models used for the Nurses’ Health Study [11-13] were developed only for the Northeastern and Midwestern U.S. and for 1988–2002. In the present analysis, we expand the modeling domain to the conterminous U.S. and include PM_2.5_ and PM_10_ monitoring data through 2007. We demonstrate the predictive accuracy of a computationally efficient but flexible spatio-temporal modeling approach, applied to the conterminous U.S., which combines spatial smoothing and regionally-varying non-linear smooth functions of time-varying and time-invariant geographic and meteorological covariate effects. Also, we evaluate the potential for improved model prediction resulting from the use of geographic covariates with higher spatial resolution than those used previously for traffic density, population density, and point-source emissions density.

## Methods

We developed three separate GIS-based spatio-temporal models of PM levels: 1) PM_2.5_ from 1999–2007, 2) PM_2.5_ from 1988–1998, and 3) PM_10_ from 1988–2007. As with our previous models for the Northeastern and Midwestern U.S. [11-13], these models used measured PM concentrations, monitoring site locations, GIS-based location-specific characteristics and location- and month-specific meteorological data, and spatial smoothing of monthly and long-term average levels to describe large- and small-scale spatial variability and temporal variability in PM_2.5_ and PM_10_ levels over time.

### Air pollution, geographic, and meteorological data

#### Air pollution data

Monthly mean PM_2.5_ and PM_10_ values were calculated from available monitoring data using the same methods as for our previous models [12,13]. Briefly, PM_2.5_ and PM_10_ measurement data from 1988–2007 were obtained from the U.S. Environmental Protection Agency’s Air Quality System (AQS) network, from the Interagency Monitoring of Protected Visual Environments (IMPROVE), Clean Air Status and Trends (CASTNet), Stacked Filter Unit (SFU), Southeastern Aerosol and Visibility Study (SEAVS), Measurement of Haze and Visual Effects (MOHAVE), and Pacific Northwest Regional Visibility Experiment Using Natural Tracers (PREVENT) networks by accessing the Visibility Information Exchange Web System [27], from three Harvard-based research studies: the “Five Cities” study [28], the “24 Cities” study [29], and the “Six Cities” study [30], and from the Southern Aerosol Research and Characterization Study (SEARCH) network [31]; summary statistics on the monitoring data can be found in Additional file 1: Table S1. Monthly means were calculated by first averaging 24-hr mean values at each monitoring site, and then averaging the daily (with the exception of CASTNet which provided 2-week means) site means within the calendar month, provided that greater than approximately 70% of the nominal days had valid PM_2.5_ or PM_10_ values. The AQS contributed the bulk of the monthly means and sites (94 and 91%, respectively, for the 1999–2007 PM_2.5_ model; 89 and 86%, respectively, for the 1988–1998 PM_2.5_ model; and 93 and 89%, respectively, for the PM_10_ model).

#### Geographic data

Characteristics of the PM monitoring sites were quantified using a GIS (ArcMap 10.1, Environmental Systems Research Institute (ESRI), Redlands, CA). We considered only geographic data available (*i.e.*, non-missing) over the conterminous U.S. to facilitate generating model predictions at any location within this domain. The Albers Equal Area Conic U.S. Geological Survey (USGS) projection was used for all geographic data.

We estimated traffic density using data from the U.S. Bureau of Transportation Statistics 2005 National Highway Planning Network (NHPN) [32] using a kernel density function (ESRI Spatial Analyst) evaluated on a 30 m cell size raster. The kernel density approach involves deriving locally varying values by applying weights from a quadratic kernel within a specified neighborhood [33]. The neighborhood for this function was specified at 100 m based on data from previous studies of near-road pollutant decay [34,35]. Distance to nearest road values were also generated for each monitoring site for U.S. Census Feature Class Code (CFCC) road classes A1 (primary roads, typically interstates, with limited access), A2 (primary major, non-interstate roads), A3 (smaller, secondary roads, usually with more than one lane in either direction), and A4 (roads used for local traffic usually with one lane in either direction) roads using ESRI StreetMap Pro 2007 road network data. Distance to road values were truncated at 500 m; as a result this term represented only micro- to middle-scale local variability in PM levels near roadways.

The proportion of residential (low-intensity and high-intensity) and urban (low-intensity and high-intensity residential, and industrial/commercial/transportation) land use was calculated for each location using neighborhoods of 1 and 4 km, using data from the U.S. Geological Survey (USGS) 1992 National Land Cover Dataset [36]. Tract-level population density data derived from the 1990 U.S. Census were obtained [37] and converted to a 500 m cell-size raster, based on the location of the center of each cell. Density values at each cell were averaged with the values at four adjacent cells, one in each cardinal direction, to reduce spatial discontinuities across cells. County-level population density data from the 1990 U.S. Census were obtained from ESRI Data & Maps and were spatially smoothed from county geographic centroids to prediction locations using a generalized additive model (GAM) with spatial bivariate thin-plate penalized splines [38].

We estimated the density of point-source emissions of PM_2.5_ and PM_10_ using kernel density functions (ESRI Spatial Analyst) with neighborhoods of 3, 7.5, and 15 km and data from the U.S. EPA’s 2002 National Emissions Inventory [39]. In our earlier work, 1 and 10 km buffers were used [11-13]. Larger neighborhoods were chosen for this analysis to reflect more distant sources; however, values at greater distances were down-weighted due to use of the kernel density function. Also, neighborhoods with<=50% overlap were chosen, to minimize collinearity.

Elevation data were obtained in raster format from the USGS’s National Elevation Dataset [40] (with a native resolution of ~ 30 m) and averaged using a moving window with a radius of 300 m.

The traffic density within 100 m, distance to nearest road, tract- and county-level population density, and point-source emissions density covariates were natural-log transformed, after the addition of a constant, to obtain a more uniform distribution and thereby improve stability in the estimation of the penalized spline smooth functions, using the formula:

(1) $$Z_{i,t}=\ln({Z^{*}}_{i,t}-\left(10-\min\left({Z^{*}}_{i,t}\right)\right)$$

where *Z*_*i. t*_ is the transformed covariate, Z*_i,*t*_ is the covariate on the native scale, and the constant 10 was chosen to reduce the leverage of values near zero. Similarly, the elevation covariate was transformed using a square root transformation after adding a constant to ensure a minimum value of one.

#### Meteorological data

**Figure 1:**
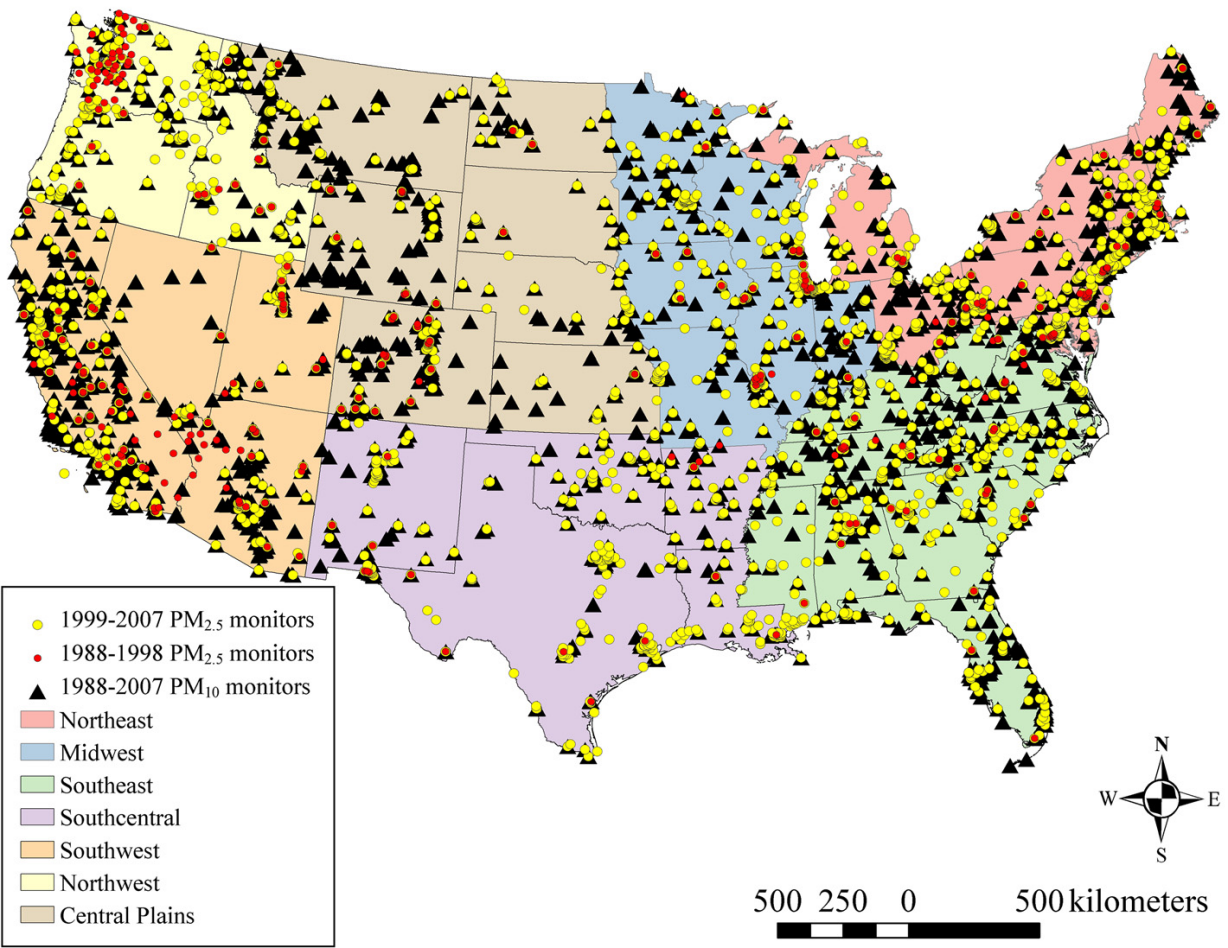
**1999–2007 PM**_**2.5**_**, 1988–1998 PM**_**2.5**_**, and PM**_**10 **_**monitor locations, as well as regions of the conterminous U.S.**

Monthly average wind speed, temperature, and total precipitation measurements were obtained from the National Climatic Data Center (NCDC) and spatially smoothed using separate GAMs, as specified below, for each month and for each of seven regions of the conterminous U.S. (Figure 1), with region boundaries based loosely on the U.S. Census Regions [41]. Monthly predictions of the meteorological parameters at the prediction locations (monitoring sites, grid points, or geocoded subject residences) were then made using the fitted models. The form of these models was:

(2) $$y_{i,t}=\alpha_{t,r}+g_{t,r}\left(s_{i}\right)+e_{i,t};\;e_{i,t}\sim N\left(0,{\sigma^{2}}_{t,r}\right)$$

where *y*_*i*,*t*_ represents the measured values for a given meteorological parameter at *i* = 1… *I*_*r*_ sites in each of seven geographical regions indexed by *r* (Northeast, Midwest, Southeast, Southcentral, Southwest, Northwest, and Central Plains; Figure 1) and *t* = 1…*T* monthly time periods (*T* =240 for 1988–2007), and *s*_*i*_ is the projected spatial coordinate pair for the *i*th location. *g*_*t*,*r*_(*s*_*i*_) accounts for residual monthly spatial variability within the region, specified as spatial bivariate thin-plate penalized spline terms with basis dimension *k*_*t*,*r*_ = *I*_*t*,*r*_ * 0.9. The value of 0.9 was chosen such that the basis dimension was as large as practicable which allowed the data to determine the complexity of the fitted functions, but was essentially arbitrary. To reduce the potential for over-fitting, a multiplier of 1.4 (using the gamma argument to gam()), as recommended by Wood [39], p. 195, was used. Additionally, data on the percentage of stagnant air days per month from the NCDC’s Air Stagnation Index [42] were obtained, natural-log transformed, and spatially smoothed using GAMs (Equation 2) for each month and region.

### Statistical models

#### The 1999–2007 PM_2.5_ model

The generic form of the 1999–2007 PM_2.5_ model was:

(3) $$\begin{array}{l} y_{i,t}=\alpha +\alpha_{t,r}+\sum_{q}d_{q}\left(X_{i,q}\right)+\sum_{p}f_{p,r}\left(Z_{i,t,p}\right)+g_{t,r}\left(s_{i}\right)\\ \;+g\left(s_{i}\right)+b_{i}+e_{i,t};b_{i}\sim N\left(0,{\sigma^{2}}_{b}\right);\;e_{i,t}\sim N\left(0,{\sigma^{2}}_{e\;r,t}\right) \end{array}$$

where *y*_*i*,*t*_ is the natural-log transformed monthly average PM_2.5_ for *i* = 1…*I*_*r*_ sites in each of the seven geographic regions indexed by *r* (Figure 1) and *I* sites in total and *t* = 1…*T* monthly time periods (*T*=108 for PM_2. 5_ from 1999–2007), and *s*_*i*_ is the projected spatial coordinate pair for the *i*th location. *X*_*i*,*q*_ are GIS-based time-invariant spatial covariates for *q* = 1…*Q*, *Z*_*i*,*t*,*p*_ are time-varying covariates for *p* = 1…*P*, and *α*_*t*,*r*_ is a monthly intercept that represents the mean across all sites within the region. *d*_*q*_ and *f*_*p*,*r*_ are one-dimensional penalized spline smooth functions for *Q* GIS-based time-invariant spatial covariates and *P* time-varying meteorological covariates, respectively, each with a basis dimension of 10. *g*_*t*,*r*_(*s*_*i*_) accounts for residual monthly spatial variability within the region, and *g*(*s*_*i*_) for time-invariant spatial variability across the conterminous U.S., with both terms specified as spatial bivariate thin-plate penalized splines with basis dimension values: *k*_*t*,*r*_ = *I*_*t*,*r*_ * 0.9 and *k* = (*I* − *Q*) * 0.9, respectively. The site-specific random effect *b*_*i*_ represents unexplained site-specific variability; thus our characterization of the model as a GAMM.

We used a two-stage modeling approach to fit the above model (Equation 3). In the first stage (Equation 4), we estimated site-specific intercepts (*u*_*i*_) adjusting for time-varying covariates and residual monthly spatial variability separately for each of the seven geographic regions. This allowed the effects of time-varying covariates to vary among the regions, and assumed that the residual monthly spatial terms were stationary and isotropic only within the region rather than across the entire conterminous U.S. Fitting the first stage regionally, rather than for the entire conterminous U.S. at once, also reduced the computational burden of model fitting, necessary due to the large number of monthly observations (120,618 for PM_2.5_ from 1999–2007). Data from areas of adjacent states within about 400 km of each region were included in the regional first-stage models to minimize potential boundary effects.

The first stage model equation was:

(4) $$y_{i,t}=u_{i}+\alpha_{t,r}+\sum_{p}\;f_{p,r}\left(Z_{i,t,p}\right)+g_{t,r}\left(s_{i}\right)+e_{i,t};e_{i,t}\sim N\left(0,{\sigma^{2}}_{e\;r,t}\right)$$

and was fit iteratively for each region in a back-fitting arrangement [43,11-13] with $u_{i}+\alpha_{t,r}+\sum_{p}f_{p,r}\left(Z_{i,t,p}\right)$ estimated jointly and *g*_*t*,*r*_(*s*_*i*_) estimated separately by month, such that variability in the measured concentrations is parsed between the covariates and the residual spatial terms. For the spatial models in the first stage, a multiplier of 1.4 (using the gamma argument to gam()) was used to avoid over-fitting [39], p. 195, except in the Northwest region, where, due to limited data, a value of 1.8 was used. All individual fits within the back-fitting were done using the gam() function in the mgcv package [44] of R [45].

In the second stage, we fit a spatial model to the ${\hat{u}}_{i}$ vector of values obtained from the regional first-stage models using GIS-based time-invariant spatial covariates and residual time-invariant spatial variability. To do this, we combined the regional data sets from the first stage, after eliminating the overlapping data from the 400 km regional buffers. Thus the second stage (Equation 5) was fit to data from the entire conterminous U.S. (*i.e.*, all seven regions), and was:

(5) $${\hat{u}}_{i}=\alpha +\sum_{q}d_{q}\left(X_{i,q}\right)+g\left(s_{i}\right)+b_{i};b_{i}\sim N\left(0,{\sigma^{2}}_{b}\right)$$

where ${\hat{u}}_{i}$ is an estimated site-specific intercept that represents the adjusted long-term mean at each location; the other terms are as above. The second stage was also fit using the gam() function in the mgcv package of R. Because we included data from the entire conterminous U.S. in the second stage, we investigated the extent to which the time-invariant covariate effects varied by region of the country. We did this by including interaction terms by region, adding: $\alpha_{r}+\sum_{r}d_{r,q}\left(X_{i,q}*M_{i}\right)$ separately to the model for each covariate *q*, where *α*_*r*_ is a categorical variable for the main effect of region, and *M*_*i*_ is a zero/one indicator for whether location *i* is in a given region or not. We also explored regional interactions of covariate effects that varied smoothly in space using tensor products of penalized smoothing spline bases [39].

#### The 1988–1998 PM_2.5_ model

As in our previous work [13], the generic form of the 1988–1998 PM_2.5_ model was:

(6) $$\begin{array}{l} y_{i,t}=\alpha +\sum_{q}d_{q}\left(X_{i,q}\right)+\sum_{p}f_{p,r}\left(Z_{i,t,p}\right)+h\left(t\right)+g_{\mathrm{Seas},r}\left(s_{i}\right)\\ \;+g\left(s_{i}\right)+b_{i}+e_{i,t};b_{i}\sim N\left(0,{\sigma^{2}}_{b}\right);\;e_{i,t}\sim N\left(0,{\sigma^{2}}_{e,t}\right) \end{array}$$

where the terms are as above except that the response variable *y*_*i*,*t*_ is the natural-log transformed ratio of monthly average PM_2.5_ to model predicted PM_10_. Note that data from 1988–2007 were used for model fitting. Thus *T* = 240 even though this model was used to predict PM_2.5_ levels for only the 132 months from 1988–1998. The model was fit to 130,594 observations; 419 value were deleted as outliers where monthly average PM_2.5_ was greater than 1.5 times predicted PM_10_. Also, to account for non-linearity in the ratio as predicted PM_10_ levels increase, predicted PM_10_ (from Equation 3) was included in the model as an additional time-varying covariate *Z*_*i*,*t*,*p*_. Finally, *g*_*Seas*,*r*_(*s*_*i*_) accounts for residual seasonal spatial variability within the region for each of four seasons (winter, spring, summer, autumn), rather than for each month. The model was fit using a two-stage approach, as for the 1999–2007 PM_2.5_ model above.

#### The 1988–2007 PM_10_ model

The generic form as well as model fitting of the 1988–2007 PM_10_ model was the same as for the 1999–2007 PM_2.5_ model, except that the response variable, *y*_*i*,*t*_, was the natural-log transformed monthly average PM_10_ and *T* = 240, with 280,060 monthly observations from 1988–2007. The model was fit using a two-stage approach, as for the 1999–2007 PM_2.5_ model above.

#### Model predictions

We obtained model predictions from each model by generating the covariates at locations of interest (either monitoring locations for model evaluation or grid locations for display purposes) for each month, and then transforming to the native scale by exponentiation. To avoid extrapolation, covariates at prediction locations beyond their range among the monitoring locations were set to the appropriate minimum or maximum among the monitoring locations (doing so within each region for time-varying covariates). For the 1988–1998 PM_2.5_ model, exponentiation yields the predicted PM_2.5_:PM_10_ ratio (which was truncated to a maximum value of one, affecting only 0.8% of the data), which was multiplied by predicted PM_10_ to obtain predicted PM_2.5_ (${\hat{\mathrm{PM}}}_{2.5\;i,t}=\exp\left({\hat{y}}_{\mathrm{ratio}\;i,t}\right)*\exp\left({\hat{y}}_{PM_{10}\;i,t}\right)$) for 1988–1998. We calculated PM_2.5–10_ levels at unmeasured locations and months by subtracting predicted PM_2.5_ from predicted PM_10_ (${\overset{ˆ;}{\mathrm{PM}}}_{2.5-10\;i,t}={\hat{\mathrm{PM}}}_{10\;i,t}-{\hat{\mathrm{PM}}}_{2.5\;i,t}$, notation as above).

We generated estimates of uncertainty in model predictions (*i.e.*, standard errors) from the 1999–2007 PM_2.5_ and 1988–2007 PM_10_ models on the natural-log scale using methods described previously [11,12]. For these models, 95% prediction intervals on the natural-log scale were calculated and exponentiated to assess prediction interval coverage. To generate standard errors for the 1988–1998 PM_2.5_ model, we propagated errors in the predicted PM_2.5_:PM_10_ ratio and predicted PM_10_ levels on the native scale (see Additional file 1 for details). We also propagated errors in the PM_2.5–10_ predictions on the native scale using standard methods, assuming independence among the ${\hat{\mathrm{PM}}}_{2.5}$ and ${\hat{\mathrm{PM}}}_{10}$ errors. For 1988–1988 PM_2.5_ model predictions as well as for PM_2.5–10_ predictions, prediction interval coverage was assessed using 95% prediction intervals based on these native-scale standard errors.

#### Model validation

We used 10-fold out-of-sample cross-validation (CV) to evaluate model predictive accuracy and thereby inform covariate selection. For the 1999–2007 PM_2.5_ and 1988–2007 PM_10_ models, monitoring sites were selected at random and assigned exclusively to one of 10 sets. Because few PM_2.5_ data were available prior to 1999, we used data from the year 2000 for CV of the 1988–1998 PM_2.5_ model. To do this, we first identified a subset of sites that reported at least 10 monthly PM_2.5_ values in 2000 and at least 70 monthly PM_2.5_ values across 1988–2007. We then randomly selected from among these sites data not to be used for CV (ensuring reasonable spatial coverage within each region by manipulating the random seed), with the goal of making the data for 2000 similar to that in years prior to 1999 for the purpose of model fitting. We subsequently divided the remaining monitoring sites that reported data in 2000 at random and assigned each site exclusively to one of 10 sets. Since the covariate selection process involved fitting multiple candidate models to the same data, set 10 was reserved (*i.e.*, not used for model fitting) to assess whether the covariate selection process contributed to over-fitting. Data from sets one to nine (each set contains approximately 10% of the data for the 1999–2007 PM_2.5_ and 1988–2007 PM_10_ models) were removed from the data set sequentially, with the model fit to the remaining data and model predictions generated at the locations and months of the left-out observations.

The predictive accuracy of each PM model was determined from the squared Pearson correlation between the monthly left-out observations and model predictions (CV R^2^), with both on the native rather than the natural-log scale. Spatial CV R^2^ values were calculated similarly but on the long-term means (*i.e.*, one mean per site) of the monthly values. Prediction errors were calculated by subtracting left-out observations from the model predictions. Bias in model predictions was determined using the normalized mean bias factor (NMBF) [Shaocai Yu, personal communication] and the slope from major-axis linear regression [46] of the natural-log transformed left-out observations against the natural-log transformed model predictions. The precision of model predictions was obtained by taking the mean of the absolute value of the prediction errors (CVMAE) and using the normalized mean error factor (NMEF) [Shaocai Yu, personal communication]. Formulas for the NMBF and NMEF are provided in Additional file 1. Bias and precision values from CV were evaluated overall, and by region of the country, urban land use, season, monitoring network, and monitoring objective.

#### Model development and covariate selection

For each model, we first fit a ‘base’ model using the following covariates based on our earlier work [11-13]: distance to nearest road for U.S. CFCC road classes A1-A3, smoothed county-level population density, urban land use within 1 km, elevation, point-source emissions density within 7.5 km (of PM_2.5_ emissions for the PM_2.5_ models and PM_10_ emissions for the PM_10_ model), smoothed monthly average wind speed, temperature, total precipitation, and air stagnation. The 1988–2007 PM_10_ model also included tract-level population density. To ensure a parsimonious model specification, we then removed each time-varying term to evaluate its contribution and kept in the model only those that improved predictive accuracy (using the ‘base’ set of covariates in the second-stage model). Using the remaining time-varying covariates, we then added or substituted GIS-based time-invariant spatial covariates into the second stage of the model, selecting the model with the highest spatial CV R^2^, after removing those not statistically significant (p>0.05) per the result of Wald tests [38]. As in prior work, only those covariates expected *a priori* to have a positive or negative physical influence on PM levels were considered for inclusion. For example, increasing wind speed (a proxy for the amount of vertical mixing in the atmosphere) was expected to result in decreased PM_2.5_ concentrations due to dilution of pollutant emissions. Non-linearity in covariate effects was accounted for using penalized spline terms, with the ‘sp’ argument to gam() used to limit each penalized spline terms to at most six degrees of freedom (df). Similarly, we used the ‘sp’ argument to gam() to evaluate reducing the df of the spatial term in the second stage of the 1999–2007 PM_2.5_ model.

## Results

### Spatial patterns in model predicted PM_2.5,_ PM_10_, and PM_2.5–10_ concentrations

**Figure 2:**
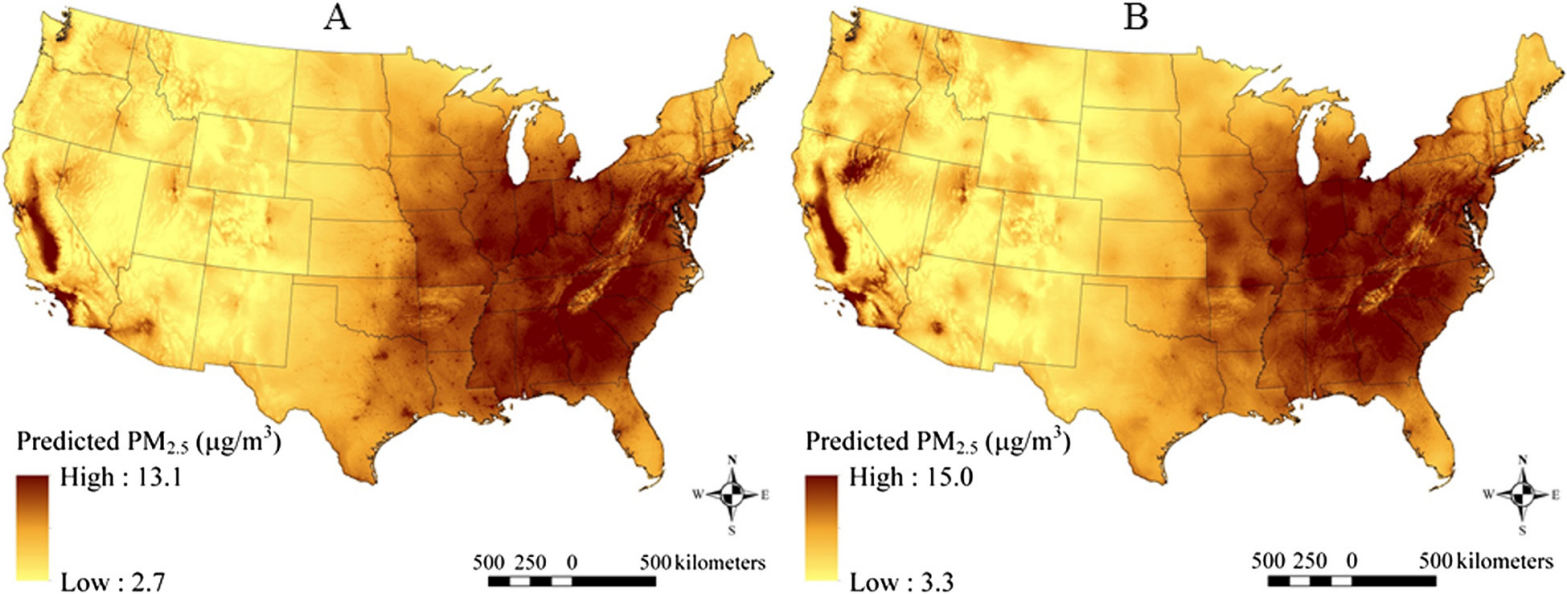
**Means of monthly predicted PM**_**2.5 **_**concentrations on a 6 km grid over the conterminous U.S. (5**^**th **^**to 95**^**th **^**percentiles shown) for A) 1999–2007 and B) 1988–1998.**

**Figure 3:**
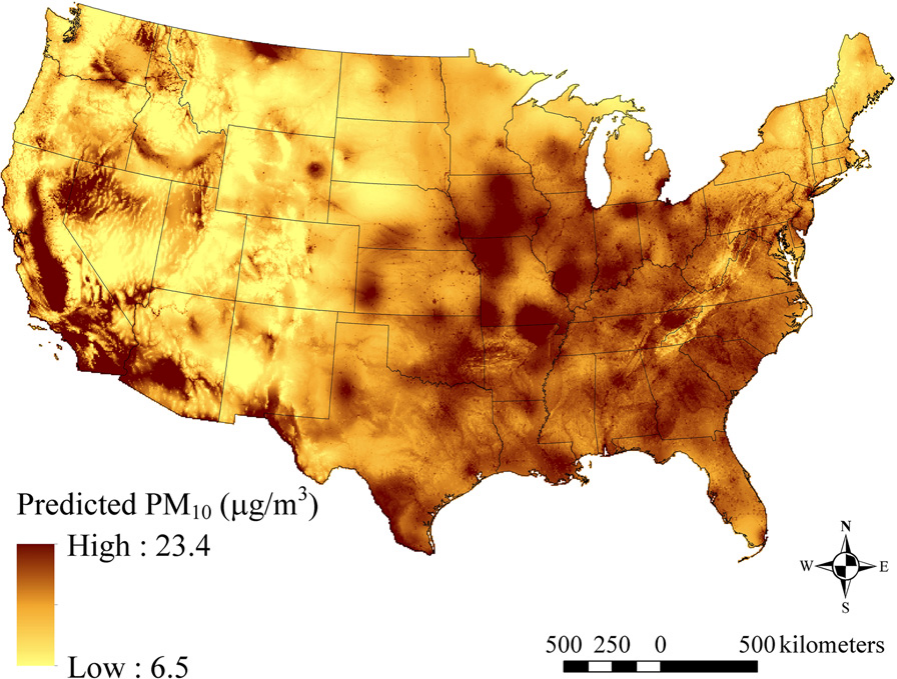
**Means of monthly predicted PM**_**10 **_**concentrations from 1988–2007 on a 6 km grid over the conterminous U.S. (5**^**th **^**to 95**^**th **^**percentiles shown).**

**Figure 4:**
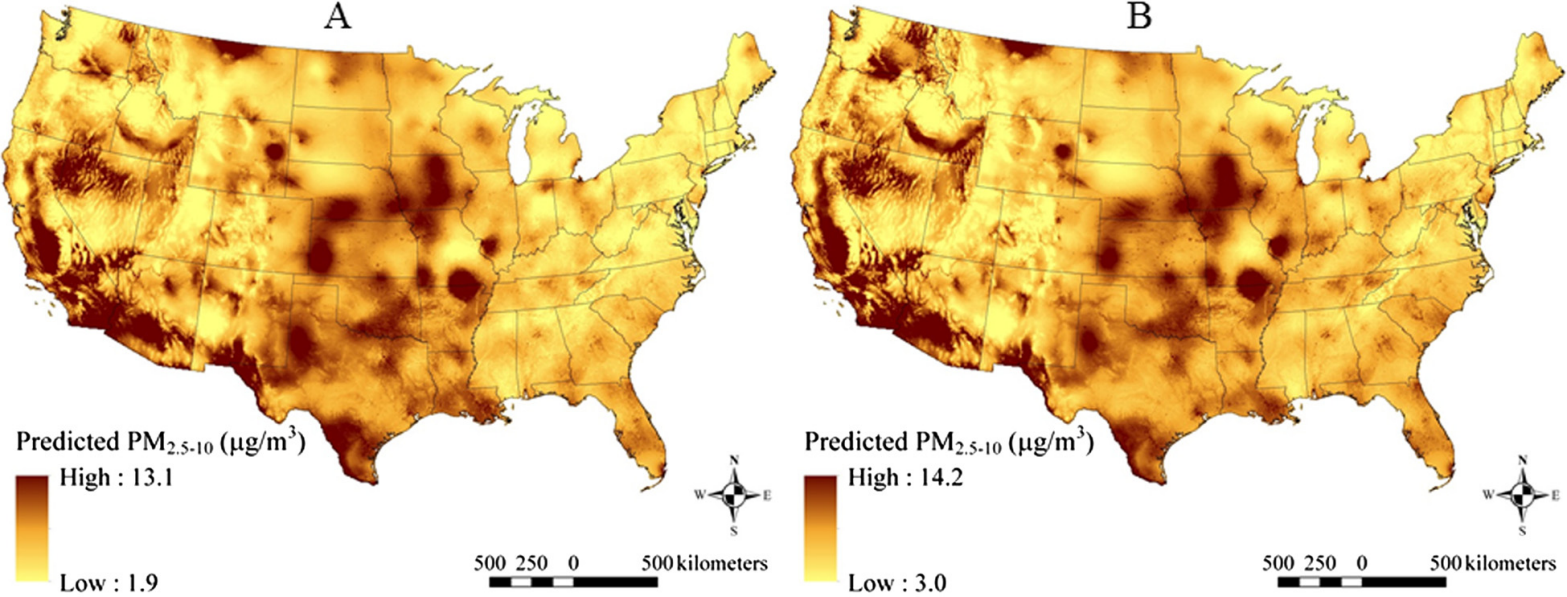
**Means of monthly predicted PM**_**2.5–10 **_**concentrations on a 6 km grid over the conterminous U.S. (5**^**th **^**to 95**^**th **^**percentiles shown) for A) 1999–2007 and B) 1988–1998.**

The maps in Figures 2, 3 and 4 show the spatial distribution of long-term average model predicted PM_2.5_, PM_10_, and PM_2.5–10_ levels across the contiguous U.S. (see Additional file 2 for an atlas of monthly PM_2.5_ levels from January 1988 to December 2007). Summary statistics of measured and predicted levels for each of the PM size fractions are presented in Additional file 1: Table S1 overall, by region, and by network for the 1999–2007 and 1988–1998 time periods. Generally, PM_2.5_ levels were highest in southern California, and were elevated across the eastern as compared to western U.S. PM_10_ and PM_2.5–10_ levels were also highest across the Southwest and Central Plains regions (presumably due to greater contributions from windblown dust than in other areas), and were generally more spatially variable than PM_2.5_. Areas of higher elevation had generally lower predicted PM_2.5_ and PM_10_ levels. Increases in model predicted PM levels in areas with higher urban land use are also evident, especially for PM_2.5_.

**Figure 5:**
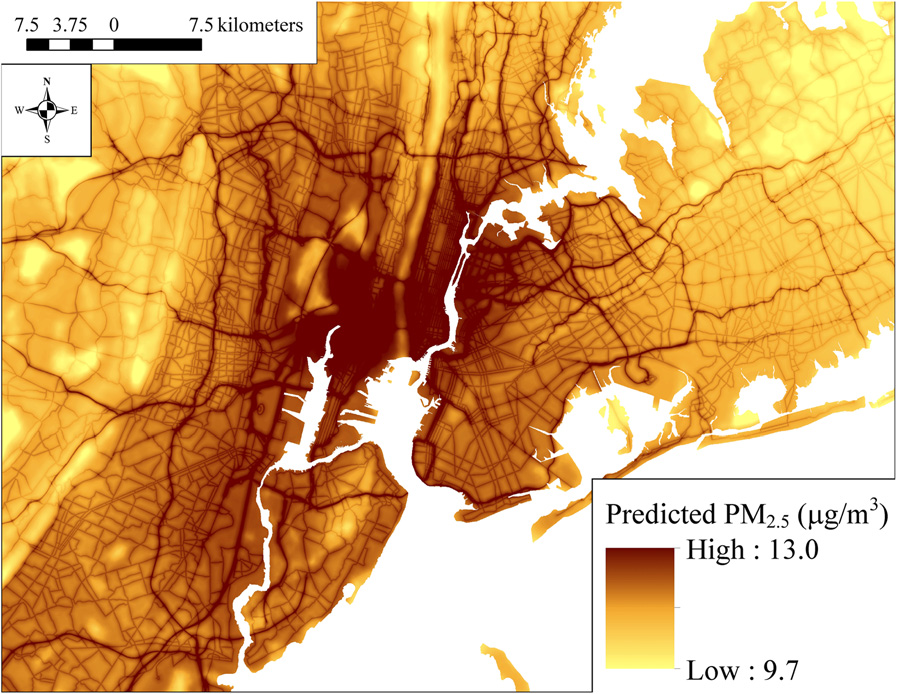
**Predicted PM**_**2.5 **_**concentrations (from the 1999–2007 model) on a 30 m grid in a selected area of New York City, New York for August 2006 showing local spatial variability (5**^**th **^**to 95**^**th **^**percentiles shown).**

**Figure 6:**
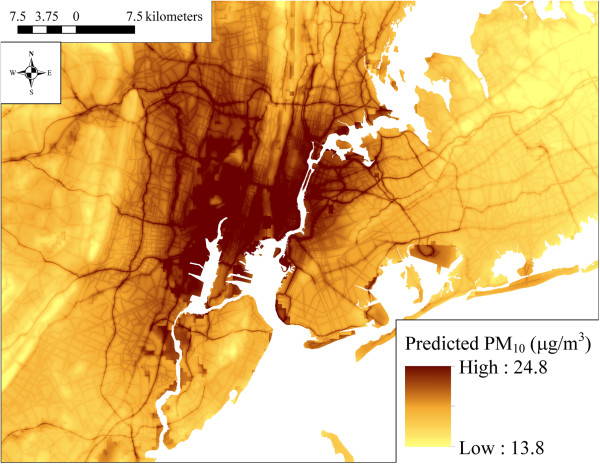
**Predicted PM**_**10 **_**concentrations on a 30 m grid in a selected area of New York City, New York for August 2006 showing local spatial variability (5**^**th **^**to 95**^**th **^**percentiles shown).**

**Figure 7:**
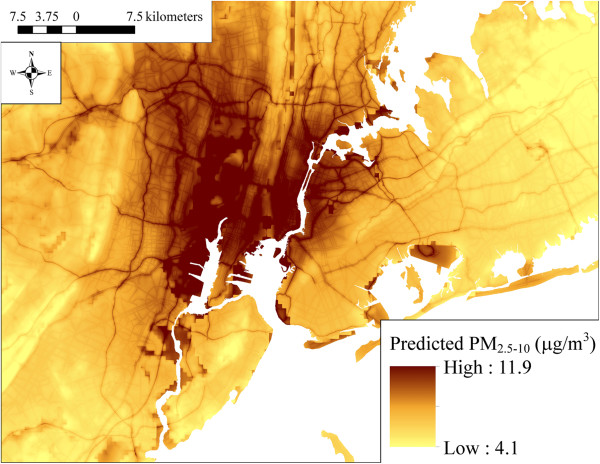
**Predicted PM**_**2.5–10 **_**concentrations on a 30 m grid in a selected area of New York City, New York for August 2006 showing local spatial variability (5**^**th **^**to 95**^**th **^**percentiles shown).**

At the spatial resolution (6 km) shown in Figures 2, 3 and 4, it is not possible to discern the micro- and middle-scale impacts of the distance to road covariates, though they are evident in Figures 5, 6 and 7, which display model predicted PM_2.5_, PM_10_, and PM_2.5–10_ concentrations on a 30 m grid in a selected area of New York City, New York for August 2006. Of note, sharp gradients in tract-level population density in this area together with the decreasing smooth function for this covariate result in several somewhat abrupt changes in predicted PM_10_ and therefore also predicted PM_2.5–10_.

**Figure 8:**
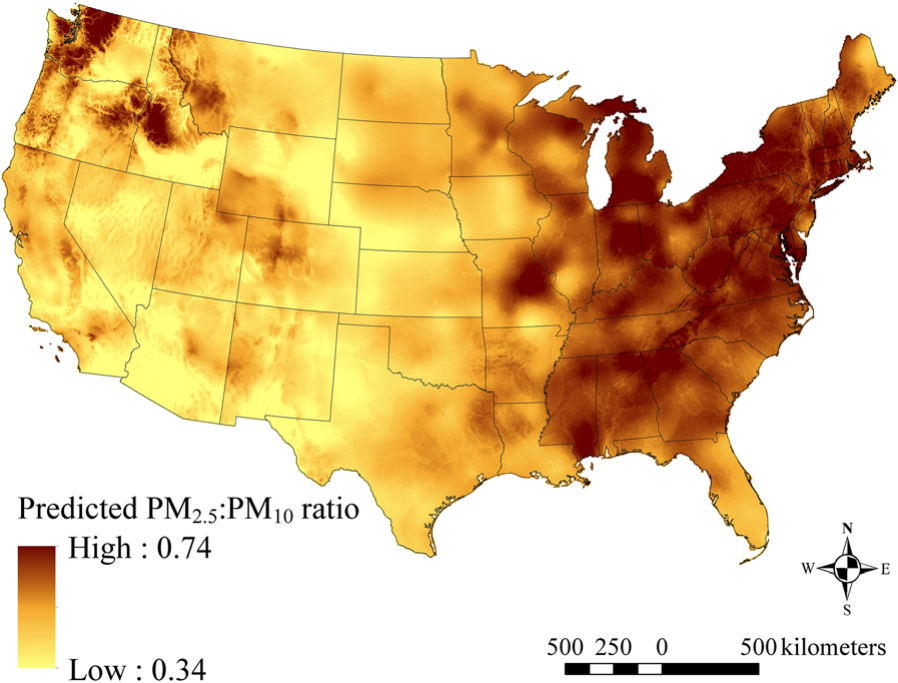
**Means of monthly predicted PM**_**2.5 **_**to PM**_**10 **_**ratios from 1988–1998 on a 6 km grid over the conterminous U.S. (5**^**th **^**to 95**^**th **^**percentiles shown).**

Maps of the mean standard errors of monthly PM_2.5_, PM_10_, and PM_2.5–10_ model predictions for the conterminous U.S. are shown in Additional file 1: Figures S4-S6. Though the spatial patterns in the mean of the standard errors (with higher values corresponding to greater average uncertainty in monthly model predictions) for each PM size fraction are similar to the corresponding spatial pattern in mean model predictions, standard errors from the 1999–2007 PM_2.5_ model are comparatively higher than model predictions in the Central Plains region (in eastern Kansas, for example), and in northwestern Nevada. Also of note, the magnitude of the standard errors from the 1988–1998 PM_2.5_ model is generally greater than that from the 1999–2007 PM_2.5_ model, reflecting uncertainty related to the estimation of the PM_2.5_:PM_10_ ratio and, separately, of PM_10_ levels. A map of the mean predicted PM_2.5_:PM_10_ ratio across 1988–1998 is presented in Figure 8. The estimated ratio is generally higher in the eastern as compared to the western U.S., though areas of the Northwest region are also higher as compared to the rest of the western U.S.

### CV results

Results from CV for 1999–2007 for PM_2.5_, PM_10_, and PM_2.5–10_ are presented in Table 1; for 1988–1998 they are presented in Table 2. Overall and when stratified by region, spatial CV R^2^ values were higher than those for corresponding monthly values for PM_2.5_, PM_10_, and PM_2.5–10_ across 1999–2007 and 1988–1998 (Tables 1 and 2, respectively). CV statistics by season, tertiles of urban land use, monitoring network, and monitoring objective are presented for each of the two time periods above in Additional file 1: Table S2 for PM_2.5_, PM_10_, and PM_2.5–10_. For both time periods, predictive accuracy was generally consistent across tertiles of urban land use, monitoring network, and monitoring objective for PM_2.5_, PM_10_, and PM_2.5–10_. Also model predictive performance was consistent across seasons, though generally slightly lower in the winter season as compared to other seasons. Density scatter plots of monthly measured *vs.* model predicted PM_2.5_, PM_10_, and PM_2.5–10_ levels from CV are shown in Additional file 1: Figure S2.

**Table 1:** **Bias and precision statistics from cross-validation (CV) of PM**_**2.5**_**, PM**_**10**_**, and PM**_**2.5–10 **_**models from 1999-2007**

**Pollutant**	**Region**^**A**^	**Monthly values**									**Spatial CV R**^**2**^^**G**^
		**N**^**B**^	**N excluded**^**C**^	**Model R**^**2D**^	**CV R**^**2**^	**Intercept**^**E**^	**Slope**^**E**^	**NMBF (%)**^**F**^	**CVMAE**^**F**^	**NMEF (%)**^**F**^	
PM_2.5_	All	108,718	4	0.84	0.77	0.3	0.87	−1.6	1.61	14.3	0.89
	Northeast	24,318	0	0.85	0.81	0.2	0.92	−1.4	1.44	11.4	0.88
	Midwest	15,767	0	0.85	0.81	0.2	0.91	−0.7	1.31	10.6	0.89
	Southeast	24,201	1	0.88	0.83	0.2	0.92	−0.4	1.31	9.7	0.82
	Southcentral	12,762	0	0.79	0.72	0.2	0.89	−0.6	1.44	14.1	0.83
	Southwest	13,448	2	0.79	0.69	0.4	0.81	−5.5	2.65	26.8	0.83
	Northwest	9,052	0	0.65	0.50	0.7	0.62	−4.6	2.07	28.9	0.62
	Central Plains	9,170	1	0.72	0.60	0.4	0.81	−2.8	1.66	23.2	0.81
PM_10_	All	104,509	22	0.71	0.58	0.7	0.77	−5.1	5.21	24.4	0.69
	Northeast	16,982	0	0.67	0.57	0.7	0.76	−4.7	4.17	19.8	0.68
	Midwest	10,088	0	0.63	0.48	0.9	0.71	−6.0	4.82	21.2	0.56
	Southeast	20,316	0	0.62	0.49	0.7	0.76	−4.0	3.89	17.6	0.46
	Southcentral	8,092	0	0.61	0.45	0.8	0.74	−6.0	6.24	27.4	0.44
	Southwest	24,050	19	0.76	0.62	0.7	0.79	−4.7	6.92	27.8	0.72
	Northwest	5,943	1	0.59	0.49	0.8	0.71	−1.6	5.33	30.2	0.72
	Central Plains	19,038	2	0.61	0.50	0.8	0.71	−7.6	5.11	31.3	0.66
PM_2.5-10_^H^	All	41,098	1,936	0.67	0.52	0.6	0.76	−3.2	4.18	38.9	0.61
	Northeast	8,375	423	0.49	0.35	0.9	0.58	−8.9	3.46	42.7	0.53
	Midwest	4,567	233	0.61	0.43	0.7	0.70	−1.5	3.72	34.4	0.49
	Southeast	7,178	359	0.45	0.28	0.5	0.75	−4.2	3.02	38.0	0.36
	Southcentral	3,614	23	0.61	0.40	0.9	0.62	−11.4	5.63	44.6	0.33
	Southwest	9,237	296	0.74	0.56	0.5	0.81	−1.6	5.64	36.6	0.64
	Northwest	2,579	340	0.55	0.47	0.3	0.92	18.1	3.97	48.2	0.58
	Central Plains	5,548	262	0.56	0.41	0.6	0.74	−2.3	3.87	39.8	0.61

^A^Corresponds to regions shown in Figure 1.

^B^Includes data from CV sets one through nine; see text for details.

^C^Three PM_2.5_ values above 70 μg/m^3^ (>99.99th percentile) and one low value, as well as 22 PM_10_ values above 150 μg/m^3^ (>99.99th percentile) were excluded from CV statistics as outliers. Extreme values may have been due to local events such as wildland or other fires, dust storms, etc.

^D^Calculated on the native rather than natural-log scale and among observations used for CV for comparison to the CV R^2^. For PM_2.5–10_, predicted levels<=0 were removed.

^E^From major axis regression of predictions on measurements (both are natural-log transformed monthly means); see text for details.

^F^NMBF is normalized mean bias factor; CVMAE is cross-validation mean absolute error; NMEF is normalized mean error factor; see text for details.

^G^Spatial CV R^2^ calculated at 1,245, 1,192, and 512 sites with >35 valid monthly-average measurements for PM_2.5_, PM_10_, and PM_2.5–10_, respectively.

^H^Calculated as the difference between monthly PM_10_ and PM_2.5_ measurements and, separately, monthly PM_10_ and PM_2.5_ model predictions. Of the 1,936 values excluded as outliers, 11 were removed due to extreme PM_10_ or PM_2.5_ measurements; an additional 1,925 were due to measured or predicted PM_2.5–10_ below the limit of detection of 0.57 μg/m^3^ (<3.4th percentile of measured and <1.6th of predicted PM_2.5–10_).

**Table 2:** **Bias and precision statistics from cross-validation (CV) of PM**_**2.5**_**, PM**_**10**_**, and PM**_**2.5–10 **_**models from 1988–1998**

**Pollutant**	**Region**^**A**^	**Monthly values**									**Spatial CV R**^**2**^^**G**^
		**N**^**B**^	**N excluded**^**C**^	**Model R**^**2D**^	**CV R**^**2**^	**Intercept**^**E**^	**Slope**^**E**^	**NMBF (%)**^**F**^	**CVMAE**^**F**^	**NMEF (%)**^**F**^	
PM_2.5_^H^	All	10,823	0	0.82	0.77	0.2	0.92	−0.8	1.81	14.8	0.88
	Northeast	2,455	0	0.77	0.72	0.2	0.93	−0.5	1.73	13.0	0.85
	Midwest	1,564	0	0.74	0.70	0.3	0.88	−1.3	1.64	12.2	0.78
	Southeast	2,385	0	0.76	0.73	0.1	0.97	−1.0	1.74	11.7	0.78
	Southcentral	1,446	0	0.74	0.67	0.4	0.86	0.2	1.67	15.6	0.77
	Southwest	1,205	0	0.84	0.77	0.2	0.93	1.3	2.49	22.8	0.89
	Northwest	809	0	0.75	0.56	0.4	0.81	−6.1	2.12	26.8	0.60
	Central Plains	959	0	0.77	0.67	0.1	0.95	−1.2	1.55	20.2	0.81
PM_10_	All	145,398	12	0.71	0.58	0.5	0.82	−3.3	5.44	21.8	0.66
	Northeast	35,593	5	0.66	0.57	0.5	0.83	−2.5	4.71	18.2	0.57
	Midwest	16,276	0	0.65	0.51	0.7	0.78	−2.9	5.57	20.4	0.55
	Southeast	26,882	0	0.72	0.61	0.5	0.84	−1.7	4.04	15.6	0.57
	Southcentral	12,668	0	0.66	0.50	0.9	0.72	0.1	5.20	21.2	0.50
	Southwest	23,586	5	0.76	0.60	0.5	0.84	−5.1	7.52	27.4	0.68
	Northwest	8,874	2	0.63	0.52	0.9	0.72	−4.2	6.86	26.0	0.66
	Central Plains	21,069	0	0.64	0.50	0.6	0.76	−7.4	5.57	31.7	0.66
PM_2.5-10_^I^	All	4,032	205	0.61	0.45	0.7	0.70	−4.7	4.73	42.6	0.56
	Northeast	802	48	0.52	0.32	0.9	0.56	−14.6	4.28	46.5	0.37
	Midwest	378	21	0.64	0.47	0.8	0.66	−4.4	4.28	36.9	0.44
	Southeast	771	58	0.35	0.12	1.1	0.51	1.7	3.66	43.3	0.09
	Southcentral	453	2	0.58	0.43	0.8	0.68	−5.1	5.35	38.2	0.38
	Southwest	835	34	0.70	0.53	0.4	0.81	−8.4	6.15	44.4	0.70
	Northwest	271	15	0.60	0.54	0.6	0.85	27.0	4.14	47.5	0.63
	Central Plains	522	27	0.42	0.32	0.7	0.69	−4.8	4.81	45.9	0.42

^A^Corresponds to regions shown in Figure 1.

^B^Includes data from CV sets one through nine; see text for details.

^C^12 PM_10_ values above 150 μg/m^3^ (>99.99th percentile) were excluded from CV statistics as outliers. Extreme values may have been due to local events such as wildland or other fires, dust storms, etc.

^D^Calculated on the native rather than natural-log scale and among observations used for CV (for only the year 2000 for PM_2.5_ and PM_2.5–10_) for comparison to the CV R^2^. For PM_2.5–10_, predicted levels<=0 were removed.

^E^From major axis regression of predictions on measurements (both are natural-log transformed monthly means); see text for details.

^F^NMBF is normalized mean bias factor; CVMAE is cross-validation mean absolute error; NMEF is normalized mean error factor; see text for details.

^G^Spatial CV R^2^ calculated at 1,031 and 422 sites with >3 valid monthly-average measurements for PM_2.5_ and PM_2.5–10_, respectively, and at 1,502 sites with >35 valid monthly-average measurements for PM_10_.

^H^Measured and predicted levels (rather than the natural-log of the PM_2.5_ to PM_10_ ratio) were compared.

^I^Calculated as the difference between monthly PM_10_ and PM_2.5_ measurements and, separately, monthly PM_10_ and PM_2.5_ model predictions. Of the 207 values excluded as outliers, 8 were removed due to extreme PM_10_ or PM_2.5_ measurements; an additional 197 were due to measured or predicted PM_2.5–10_ below the limit of detection of 0.57 μg/m^3^ (<3.6th percentile of measured and <1.5th of predicted PM_2.5–10_).

#### CV results for 1999–2007

##### PM_2.5_

Across the conterminous U.S., predictive accuracy of the 1999–2007 PM_2. 5_ model was high (CV R^2^=0.77) at the monthly average level, though lower in the Northwest at 0.50. Across regions, model predictions exhibited low bias and high precision (NMBF of −1.6% and NMEF of 14.3%, respectively), but were less precise in the west (Southwest, Northwest, and Central Plains regions). Standard errors in monthly PM_2.5_ model predictions were reasonably well-scaled (prediction interval coverage of 0.98). The model predicted long-term spatial trends very well (spatial CV R^2^=0.89).

##### PM_10_

Across the conterminous U.S., predictive accuracy for PM_10_ monthly model predictions was moderate (CV R^2^=0.58) for 1999–2007, though lower in the Southcentral, Northwest, and Central Plains regions (>0.45). Across regions, we found low bias in model predictions but only moderate precision (NMBF of −5.1% and NMEF of 24.4% across regions, respectively). Standard errors were reasonably well-scaled for the PM_10_ model (prediction interval coverage (across 1988–2007) of 0.97). The model predicted long-term spatial trends well (spatial CV R^2^=0.69).

##### PM_2.5–10_

Across the conterminous U.S., predictive accuracy for PM_2.5–10_ was moderate (CV R^2^=0.52). Across regions, we found low bias but poorer precision than for PM_2.5_ or PM_10_ (NMBF of −3.2% and NMEF of 38.9%, respectively). In the Southcentral region, bias in PM_2.5–10_ monthly values was slightly larger and negative (NMBF of −11.4%); in the Northwest region it was also larger but positive (NMBF of 18.1%).

#### CV results for 1988–1998

##### PM_2.5_

Across the conterminous U.S., predictive accuracy for PM_2.5_ monthly model predictions was again high (CV R^2^ = 0.77), though again lower in the Northwest region at 0.56. Across regions, model predictions exhibited low bias and high precision (NMBF of −0.8% and NMEF of 14.8%, respectively), but were again less precise in the west (Southwest, Northwest, and Central Plains regions). The prediction interval coverage for the 1988–1998 PM_2.5_ model of 0.99 indicates that the standard errors are slightly inflated, likely due to the use of the delta method to approximate standard errors on the native scale prior to propagation of the uncertainty when multiplying the estimated PM_2.5_:PM_10_ ratio ($\exp\left({\hat{y}}_{\mathrm{ratio}\;i,t}\right)$) by predicted PM_10_ ($\exp\left({\hat{y}}_{PM_{10}\;i,t}\right)$).

##### PM_10_

Across the conterminous U.S., predictive accuracy for PM_10_ monthly model predictions was again moderate (CV R^2^=0.58) for 1988–2007, though again lower in the Southcentral, Northwest, and Central Plains regions for PM_10_ (>0.50). Across regions, model prediction exhibited low bias but only moderate precision (NMBF of −3.3% and NMEF of 21.8% across regions, respectively).

##### PM_2.5–10_

Predictive accuracy was moderate for PM_2.5–10_ (CV R^2^=0.46 across regions). Across regions, model predictions again exhibited low bias but precision was poorer than for PM_2.5_ or PM_10_ during the same time period (NMBF of −4.5% and NMEF of 42.5%, respectively). In the Northeast region, bias in PM_2.5–10_ monthly values was slightly larger and negative (NMBF of −14.6%), whereas in the Northwest region it was also larger but positive (NMBF of 27.0%). Predictive accuracy was also substantially lower in the Southeast region (CV R^2^=0.12). Interestingly, this decrease in predictive accuracy does not appear to be related to the lower levels of measured PM_2.5–10_ in the Southeast region; by contrast the levels in the Northwest region are comparable (Additional file 1: Table S1) but predictive accuracy in this region was not markedly reduced (CV R^2^=0.54).

### Model covariate effects

#### 1999*–*2007 PM_2.5_ model covariates

Several GIS-based time-invariant spatial covariates were found to be important predictors in the 1999–2007 PM_2.5_ model, including: elevation, urbanized land use within 1 km, county-level population density, distance to nearest A1, A2, and A3 roads, and point-source emissions density within 7.5 km.

We found significant interactions by region in the effects of two GIS-based time-invariant spatial covariates: urban land use within 1 km and elevation.

For urban land use within 1 km, regional effects in the Midwest, Southeast, Northwest, and Central Plains regions were significantly different from the remaining regions. The estimated smooth functions for this covariate, from the 1999–2007 PM_2.5_ model, showed that it was generally associated with increasing PM_2.5_ (after adjusting for other model covariates), with the pattern varying slightly by region (Additional file 1: Figure S1 panel A5).

For elevation, regional effects in the Southwest, Northwest, and Central Plains regions were significantly different from the remaining regions. Increasing elevation was generally associated with decreasing PM_2.5_, with the effects varying substantially by region, especially in the Northwest region (Additional file 1: Figure S1 panel A2). Though not visible in Figures 2, 3, 4, 5 and 6, regional covariate effects resulted in small spatial discontinuities at regional boundaries in monthly prediction surfaces.

Surprisingly, traffic density within 100 m performed slightly worse than distance to road covariates (A1-A3). This may have resulted from poorer spatial accuracy of the network of roads used by the NHPN as compared to the ESRI StreetMap Pro 2007 road network. Distance to the nearest A4 road did not increase predictive accuracy and was removed from the 1999–2007 PM_2.5_ model.

Increasing county-level population density was positively associated with measured PM_2.5_ levels, as was increasing point-source emissions density within 7.5 km (Additional file 1: Figure S1 panels A6 and A7, respectively).

As expected due to dilution and wet deposition processes, respectively, increasing levels of wind speed and total precipitation had consistent negative effects on PM_2.5_ levels in each of the seven regions (with the exception of wind speed in the Midwest). The effect of temperature on PM_2.5_ levels differed slightly by region (Additional file 1: Figure S1 panel A1), although PM_2.5_ levels generally decreased with increasing temperature. We hypothesize that this counterintuitive result may be due to cold temperatures acting as a proxy for local wood smoke emissions and less mixing in the atmosphere. In contrast, during warm seasons, higher PM_2.5_ levels due to increased photochemical production of secondary aerosol result in a less spatial variability in PM_2.5_ which is better captured by the monthly intercept and monthly spatial smooth terms in non-winter seasons as compared to in winter. We also note that the moderate correlation between temperature and air stagnation (Pearson’s r = 0.69) may interfere with direct interpretation of the effect of temperature alone. Air stagnation was found to improve predictive accuracy in only the Midwest and Southeast regions, with increasing stagnation associated with increasing PM_2.5_ levels (Additional file 1: Figure S1 panel A11), though in other regions, especially the southwest, it was inversely associated with PM_2.5_ levels.

The second-stage spatial term *g*(*s*_*i*_) exhibited substantial complexity in the 1999–2007 PM_2.5_ model, using 501.6 df. In contrast, the monthly spatial terms *g*_*t*,*r*_(*s*_*i*_) used fewer df (median across region and months of 22.7).

### 1988–1998 PM_2.5_ model covariates

For the 1988–1998 PM_2.5_ model, only predicted PM_10_ and elevation remained in the model as spatial covariates. However, the same four meteorologic covariates as for the 1999–2007 PM_2.5_ model were included in this model. Their effects were similar, except for that of total precipitation where the ratio increases and then decreases, reflecting the complexity of differential wet deposition processes for fine and coarse mode particles. We found a significant interaction by region in the effect of elevation, with the effect in the Northwest region significantly different from that in the remaining regions (Additional file 1: Figure S1 panel B2).

The second stage spatial term *g*(*s*_*i*_) exhibited substantial complexity, using 503.3 df; the seasonal spatial terms *g*_*Seas*,*r*_(*s*_*i*_) used fewer df (median of 174.1 across regions and seasons), indicating greater residual spatial variability in the seasonal (natural-logged) PM_2.5_${\hat{\mathrm{PM}}}_{10}$ ratio than in the monthly spatial terms from the 1999–2007 PM_2.5_ or 1988–2007 PM_10_ models.

### 1988–2007 PM_10_ model covariates

The 1988–2007 PM_10_ model included the same set of meteorological and GIS-based time-invariant spatial covariates as the 1999–2007 PM_2.5_ model, except that in addition it included tract-level population density. The effects of these covariates were similar to those in the 1999–2007 PM_2.5_ model, except as discussed below.

For the 1988–2007 PM_10_ model, we found significant regional interactions only for urban land use within 1 km, with effects in the Northeast, Northwest, and Central Plains regions different from that in the remaining regions. The estimated smooth functions for this covariate showed that it was generally associated with increasing PM_10_ (after adjusting for other model covariates), with the pattern varying slightly by region (Additional file 1: Figure S1 panel C4).

Tract-level population density was negatively associated with measured PM_10_ levels (Additional file 1: Figure S1 panel C8).

The second-stage spatial term *g*(*s*_*i*_) exhibited substantial complexity in the 1988–2007 PM_10_ model, using 882.6 df. In contrast, the monthly spatial terms *g*_*t*,*r*_(*s*_*i*_) used fewer df (median across regions and months of 20.1).

### Modeling assumptions

Our modeling approach assumes stationary and isotropic spatial variation, that covariate effects are additive, and that model residuals are independent and normally distributed, with mean zero and constant variance. We evaluated the assumption of stationarity in the second stage spatial term in alternative second stage models that allowed the smoothing parameter to vary across the domain (adaptive bases), including those that allowed stationarity to vary by urbanness, but these did not substantially change model fit nor increase predictive accuracy. We also evaluated whether the effects of the GIS-based time-invariant spatial covariates (other than urban land use) varied with urbanness by stratifying by tertiles of urban land use within 1 km; we found no evidence of differential covariate effects by urbanness. Finally, we evaluated temporal autocorrrelation in model residuals; the resulting plots are provided in Additional file 1: Figure S3. Though the plot for the 1988–1998 PM_2.5_ model residuals shows only limited evidence of autocorrelation, plots for the other two models show evidence of modest autocorrelation at a lag of one month and, to a lesser extent, seasonal dependence (at lags ~12 months) not accounted for in the modeling.

## Discussion

Our modeling approach provides predictions of monthly outdoor PM_2.5_, PM_10_, and PM_2.5–10_ levels at any location within the conterminous U.S. with high spatial and temporal (*i.e.*, monthly) resolution over a 20-year period (1988–2007). Model performance was particularly strong for PM_2.5_, with a CV R^2^ of 0.77 for both 1988–1988 and 1999–2007 time periods. Although lower, model performance for PM_10_ and PM_2.5–10_ was reasonable (CV R^2^=0.58 and 0.52, respectively). The strong model performance can be attributed to the fact that our models incorporate regionally-varying spatial and spatio-temporal covariate effects and account for residual spatio-temporal interaction using regional time-varying (monthly for the 1999–2007 PM_2.5_ and 1988–2007 PM_10_ models and seasonal for the 1988–1998 PM_2.5_ model) spatial smooth terms in combination with spatially smooth terms of the long-term mean. This approach gives our models the ability to account for micro (<100 m) , middle (100–500 m), neighborhood (500 m-4 km), and urban (4–50 km)-scale spatial gradients as well as larger-scale regional effects that vary over time. Further, this approach has the added benefit of straightforward interpretation of covariate effects on predicted PM levels, albeit where not obscured by collinearity or concurvity. Since model predictions can be made at a subject’s residence or other relevant point location, rather than interpolated from a pre-defined grid, our models offer high spatial resolution which may reduce exposure error when estimating chronic exposures in epidemiologic studies, as has been shown in previous analyses [11,25,26]. The models have been used to provide PM_2.5_, PM_10_, and PM_2.5–10_ monthly exposure estimates at subject residences in recent epidemiologic analyses [47,48].

Of the covariates evaluated for inclusion in the three models, several were found to be important predictors in each of the three models: wind speed, air temperature, total precipitation, air stagnation, and elevation. Also, the 1999–2007 PM_2.5_ and 1988–2007 PM_10_ models both included county population density, point-source emissions density (for the corresponding PM size fraction), distance to nearest road for road classes A1-A3, and urban land use within 1 km. Also, in the 1999–2007 PM_2.5_ and 1988–1998 PM_2.5_ models, we found regional variation in the effects of elevation, and, in the 1999–2007 PM_2.5_ and 1988–2007 PM_10_ models, of urban land use within 1 km. The robustness of our findings may be due to our covariate selection procedures which were performed using the fully specified spatio-temporal model, allowing for residual spatial trends and changing covariate effects, including potential nonlinearity in those effects, to compete with each candidate covariate, in contrast to approaches where covariate selection is based on multiple linear regression before spatial modeling is performed. Su et al. [49] used a more complicated variable selection approach, but one that may lead to over-fitting and that is not practical for models with large geographic and temporal scopes such as ours, with approximately 125,000-250,000 observations and run times for one model fit of between 24 and 96 hours. Kloog et al. [22] and Sampson et al. [16] have described attractive alternative approaches, which allow for the inclusion of large numbers of covariates while shrinking their effects, but these approaches also increase model complexity and may thus not be practical for models applied to the entire conterminous U.S. that span many years of monthly data.

Spatial trends in long-term (1999–2007) mean PM_2.5_ levels from our modeling approach, presented in Figure 2, are broadly similar to those in a recent spatial analysis of annual-average PM_2.5_ levels in the year 2000 [16] and to those in our earlier work in the Northeastern and Midwestern US [11-13]. It is possible that with additional covariates, such as satellite-derived AOD measures [19-24], model predictive accuracy (*i.e.*, CV R^2^) may improve, especially in areas far from monitors [24]. Although models have been developed that incorporate satellite-derived measures, to date there have been limited comparisons to GIS-based spatio-temporal models. For example, Lee et al. [24] used satellite-derived AOD data in combination with a low spatial resolution (2° × 2.5°) global 3-D chemical transport model (GEOS-Chem) to estimate PM_2.5_ levels in the conterminous U.S., but compared it to a kriging model without geographic or meteorological covariates that could explain small-scale spatial variability. Paciorek et al. [18] compared hierarchical spatio-temporal models that included geographic and meteorological covariates with satellite-derived AOD *vs.* those without, but only in mid-Atlantic region of the U.S., at the monthly time scale, and over one year: 2004. These models had high predictive ability, but inclusion of AOD did not improve predictive accuracy (monthly CV R^2^=0.827 without AOD and 0.825 with calibrated Moderate Resolution Imaging Spectroradiometer or Geostationary Operational Environmental Satellite AOD). More recent studies demonstrate the utility of daily as opposed to monthly satellite-derived AOD measures in New England and the mid-Atlantic states [21,22], reporting yearly CV R^2^ values of 0.83 and 0.81, respectively. However, these models cannot be used to predict PM levels before the year 2000, given that they require satellite-derived AOD data that are not available before that time period. Given the air quality monitoring, meteorological, geographic, and other data available from 1988–2007, our modeling approach provides a reasonable balance of computational feasibility (using standard software) and complexity while representing the small- and large-scale spatial, temporal, and spatio-temporal features of the data.

## Conclusions

Our models provide estimates of monthly-average outdoor concentrations of PM_2.5_, PM_10_, and PM_2.5–10_ with high spatial resolution and low bias. For PM_2.5_ and PM_10_, the models performed well in urban and rural areas and across seasons, though performance varied somewhat by region of the conterminous U.S. For PM_2.5–10_, model performance was poorer, particularly in the Southeast and Southcentral regions. Regional variation was found in the effects of geographic and meteorological covariates. The models are suitable for estimating chronic PM exposures of populations living in the conterminous U.S. from 1988 to 2007.

## Abbreviations

AOD: Aerosol optical depth; AQS: Air quality system; CASTNet: Clean air status and trends network; CFCC: U.S. census feature class code; CV: Cross-validation; CVMAE: Mean of the absolute value of the prediction errors; ESRI: Environmental systems research institute; IMPROVE: Interagency monitoring of protected visual environments; LUR: Land use regression; GAM: Generalized additive model; GAMM: Generalized additive mixed model; GIS: Geographic information system; MOHAVE: Measurement of haze and visual effects; NCDC: National climatic data center; NHPN: National highway planning network; NMBF: Normalized mean bias factor; NMEF: Normalized mean error factor; PM: Particulate matter; PM_2.5_: Fine particulate matter; mass concentration of PM<2.5 μm in aerodynamic diameter; PM_10_: Inhalable particulate matter; mass concentration of PM<10 μm in aerodynamic diameter; PM_2.5–10_: Coarse mode particle mass; mass concentration of PM>= 2.5 and <10 μm in aerodynamic diameter; PREVENT: Pacific Northwest Regional visibility experiment using natural tracers; SEAVS: Southeastern aerosol and visibility study; SEARCH: Southern aerosol research and characterization study; SFU: Stacked filter unit; USGS: U.S. geological survey.

## Competing interests

The authors declare that they have no competing interests.

## Authors’ contributions

JDY participated in the inception of the study, participated in its design, compiled and processed the geographic, meteorological, and air pollutant data, developed the statistical models, and drafted and revised the manuscript. CJP participated in the inception of the study, participated in its design, participated in the interpretation of results of the statistical modeling, and reviewed and revised the manuscript. FL obtained the original funding for the study, participated in the inception of the study, participated in the interpretation of results of the statistical modeling, and reviewed and revised the manuscript. JEH and RCP participated in the interpretation of results of the statistical modeling and reviewed and revised the manuscript. DL reviewed and revised the manuscript. HHS participated in the inception of the study, participated in the interpretation of results of the statistical modeling, and reviewed and revised the manuscript. All authors read and approved the final manuscript.

## Supplementary Material

Additional file 1 This file contains the additional results, formulas, tables, and figures referred to the in the main text. It is provided in portable document format (pdf). Click here for file

Additional file 2 **This file contains a 240-page atlas of monthly model predicted PM**_**2.5 **_**mass concentrations (in μg/m**^**3**^**) from January 1988 to December 2007 plotted on a 6 km grid over the conterminous U.S. Note: Model predictions for months prior to January 1999 are from the 1988–1998 PM**_**2.5 **_**model; thereafter they are from the 1999–2007 PM**_**2.5 **_**model.** Also, note that the scale of the legend changes across months to highlight spatial contrasts within a given month. The file is provided in portable document format (pdf). Click here for file
