# Supplementary material for: Spatio-temporal modeling of particulate air pollution in the conterminous United States using geographic and meteorological predictors
Source: Environ Health. 2014 Aug 5;13:63. doi: 10.1186/1476-069X-13-63 (PMC4137272; doi:10.1186/1476-069X-13-63)
Supplement: Additional file 1 — This file contains the additional results, formulas, tables, and figures referred to the in the main text. It is provided in portable document format (pdf). [file 1476-069X-13-63-S1.pdf]

Supplemental Material

Formulas for the normalized mean bias factor (NMBF) and normalized mean error factor (NMEF) [Shaocai Yu, personal communication], where $\hat{y}_j$ represents model predicted values and y_j represents observed (*i.e.*, measured) values, for locations indexed by j, and where bars indicate mean values, were as follows:

NMBF:

For $\bar{\hat{y}} \geq \bar{y}$ (*i.e.*, over-prediction): $NMBF = (\frac{\sum_{j=1}^{N} \hat{y}_j}{\sum_{j=1}^{N} y_j} - 1)$ [1]

For $\bar{\hat{y}} < \bar{y}$ (*i.e.*, under-prediction): $NMBF = (1 - \frac{\sum_{j=1}^{N} y_j}{\sum_{j=1}^{N} \hat{y}_j})$ [2]

NMEF:

For $\bar{\hat{y}} \geq \bar{y}$ (*i.e.*, over-prediction): $NMEF = (\frac{\sum_{j=1}^{N} |\hat{y}_j - y_j|}{\sum_{j=1}^{N} y_j})$ [3]

For $\bar{\hat{y}} < \bar{y}$ (*i.e.*, under-prediction): $NMEF = (\frac{\sum_{j=1}^{N} |\hat{y}_j - y_j|}{\sum_{j=1}^{N} \hat{y}_j})$ [4]

The arithmetic and geometric means, as well as the number of monthly values and the number of sites, for monthly-average measured and model predicted $PM_{2.5}$, PM_{10}, and $PM_{2.5\text{-}10}$ levels are presented in Table S1 across the conterminous U.S., by region, and, for monitors, by monitoring network, for each of the two time periods: 1999-2007 and 1988-1998.

Plots of the penalized spline smooth functions of the covariate effects for each of the PM models are shown in Figure S1. Density scatter plots of monthly-average measured PM levels *vs.* model predictions from cross-validation are shown in Figure S2. Plots of the temporal autocorrelation in model residuals from each of the PM models are shown in Figure S3.

Standard errors in the 1988-1998 $PM_{2.5}$ model predictions were approximated as follows, where standard errors on the native scale $SE(\exp(\hat{y}_{ratio\ i,t}))$ and $SE(\exp(\hat{y}_{PM_{10}\ i,t}))$ were obtained using the delta method:

$$SE(\widehat{PM_{2.5\ i,t}}) \cong \left|\exp(\hat{y}_{ratio\ i,t}) * \exp(\hat{y}_{PM_{10}\ i,t})\right| * \sqrt{\left(\frac{SE(\exp(\hat{y}_{ratio\ i,t}))}{\exp(\hat{y}_{ratio\ i,t})}\right)^2 + \left(\frac{SE(\exp(\hat{y}_{PM_{10}\ i,t}))}{\exp(\hat{y}_{PM_{10}\ i,t})}\right)^2} \qquad [5]$$

Standard errors in $PM_{2.5-10}$ predictions were approximated as follows:

$$SE(\widehat{PM_{2.5-10\ i,t}}) \cong \sqrt{SE(\widehat{PM_{10\ i,t}})^2 + SE(\widehat{PM_{2.5\ i,t}})^2} \qquad [6]$$

Maps of the standard errors are shown in Figures S4-S6.

Tables

Table S1. Summary statistics of monthly-average measured and model predicted $PM_{2.5}$, PM_{10}, and $PM_{2.5-10}$ levels across the conterminous U.S. for each of two time periods: 1999-2007 and 1988-1998; by region, and, for monitors, by monitoring network.

					Monthly-averages of measured values				Model predictions			
Set of locations/ Pollutant	Time period	Category	N[A]	n sites[A]	Arithmetic mean (μg/m3)	SD (μg/m3)	Geometric mean (μg/m3)	GSD	Arithmetic mean (μg/m3)	SD (μg/m3)	Geometric mean (μg/m3)	GSD
$PM_{2.5}$ monitors												
$PM_{2.5}$	1999-2007	All	120,618	2,018	11.5	5.3	10.1	1.7	11.3	4.7	10.2	1.6
		By region										
		Northeast	26,718	427	12.9	4.6	12.0	1.5	12.7	4.3	12.0	1.4
		Midwest	17,366	280	12.6	4.1	11.9	1.4	12.5	3.7	11.9	1.4
		Southeast	26,697	382	13.6	4.4	12.9	1.4	13.5	4.1	12.9	1.3
		Southcentral	14,441	271	10.2	3.8	9.4	1.6	10.1	3.5	9.4	1.5
		Southwest	15,038	260	10.5	7.6	8.3	2.0	10.0	5.9	8.4	1.9
		Northwest	10,223	212	7.5	4.6	6.3	1.9	7.1	3.2	6.4	1.6
		Central Plains	10,135	186	7.5	4.3	6.3	1.9	7.2	3.5	6.3	1.7
		By network[B]										
		AQS	113,141	1,845	11.8	5.2	10.6	1.6	11.6	4.6	10.6	1.6
		IMPROVE	6,754	285	5.7	4.1	4.5	2.1	5.7	3.6	4.8	1.8
		SEARCH	556	8	13.6	4.0	13.1	1.3	13.9	4.1	13.4	1.3
		CASTNet	167	7	11.4	4.2	10.7	1.5	11.4	2.9	11.0	1.3
	1988-1998	All	10,348	348	11.2	9.7	8.1	2.3	10.6	7.9	8.1	2.2
		By region										

			Northeast	1,507	62	17.6	10.2	14.9	1.8	15.5	6.5	13.9	1.6
			Midwest	862	33	13.4	5.3	12.1	1.6	13.9	5.1	12.7	1.6
			Southeast	1,359	47	13.9	5.8	12.8	1.5	13.3	5.3	12.4	1.5
			Southcentral	596	16	7.3	4.3	6.2	1.8	6.7	3.8	5.8	1.7
			Southwest	3,988	102	11.3	11.7	7.7	2.3	10.8	9.6	7.8	2.2
			Northwest	758	53	4.9	2.8	4.2	1.8	5.1	2.5	4.6	1.6
			Central Plains	1,278	35	4.4	3.2	3.6	1.8	4.1	2.4	3.5	1.8
			By network[B]										
			IMPROVE	5,614	83	5.9	4.2	4.7	2.0	6.0	3.9	5.0	1.8
			AQS	3,693	136	12.0	5.6	10.7	1.6	16.8	8.4	15.0	1.6
			Five Cities	296	31	16.1	4.8	15.4	1.3	15.4	4.5	14.8	1.3
			24 Cities	182	16	16.6	6.0	15.5	1.5	16.3	5.8	15.4	1.4
			SFU	75	7	8.0	5.4	6.3	2.0	8.4	5.9	6.6	2.1
			PREVENT	56	30	8.2	2.1	8.0	1.3	7.4	1.8	7.2	1.3
			MOHAVE	45	30	7.3	3.1	6.7	1.5	6.4	2.3	6.0	1.4
			SEARCH	30	5	13.8	4.1	13.2	1.2	17.2	4.3	16.6	1.3
			Six Cities	6	1	13.2	3.8	12.7	1.4	12.6	2.1	12.4	1.2
PM_{10} monitors	PM_{10}	1999-2007	All	117,105	2,044	22.5	12.5	19.3	1.8	21.5	10.0	19.2	1.6
			By region										
			Northeast	18,988	339	21.9	9.2	20.0	1.6	21.1	7.2	19.7	1.5
			Midwest	11,379	222	24.2	9.5	22.3	1.5	23.0	6.9	21.9	1.4
			Southeast	22,454	361	22.9	8.1	21.6	1.4	22.1	6.1	21.3	1.3
			Southcentral	9,234	179	23.6	12.6	20.5	1.8	22.4	9.1	20.4	1.6
			Southwest	27,348	411	26.3	17.5	21.3	2.0	25.1	14.2	21.3	1.8
			Northwest	6,769	151	18.3	11.3	14.4	2.2	17.5	8.9	14.7	1.9
			Central	20,933	381	17.6	10.4	14.5	2.0	16.5	8.2	14.4	1.7

		Plains										
		By network[B]										
		AQS	104,351	1,756	24.0	12.2	21.4	1.6	22.8	9.6	21.0	1.5
		IMPROVE	12,754	299	10.5	6.7	8.3	2.1	10.7	5.9	9.2	1.8
	1988-1998	All	162,955	2,518	25.9	12.9	22.9	1.7	25.1	10.6	22.9	1.6
		By region										
		Northeast	39,413	583	26.3	10.9	24.2	1.5	25.6	8.8	24.1	1.4
		Midwest	18,647	300	28.1	11.5	25.9	1.5	27.3	8.9	25.8	1.4
		Southeast	30,275	450	26.2	8.9	24.7	1.4	25.7	7.6	24.7	1.3
		Southcentral	14,163	192	24.3	10.4	22.2	1.6	24.0	8.2	22.6	1.4
		Southwest	26,665	426	29.6	18.2	24.8	1.8	28.4	15.3	24.7	1.7
		Northwest	10,302	173	27.8	14.7	24.4	1.7	26.6	10.8	24.5	1.5
		Central Plains	23,490	394	19.0	11.8	15.6	1.9	17.9	9.2	15.5	1.7
		By network[B]										
		AQS	157,500	2,394	25.4	12.6	22.6	1.6	25.5	10.4	23.5	1.5
		IMPROVE	4,964	72	10.9	6.8	8.8	2.0	11.5	5.8	10.3	1.6
		Five Cities	286	31	24.4	8.2	23.3	1.4	27.3	8.8	26.0	1.4
		24 Cities	187	17	28.4	8.1	27.3	1.3	28.1	8.2	27.0	1.3
		MOHAVE	11	8	14.5	4.9	13.7	1.4	10.8	3.5	10.3	1.4
		Six Cities	6	1	27.6	8.6	26.2	1.4	29.1	5.0	28.7	1.2
		SEAVS	1	1	34.4	NA[C]	34.4	NA[C]	20.1	NA[C]	20.1	NA[C]
Collocated $PM_{2.5}$ and PM_{10} monitors												

$PM_{2.5-10}$[D]	1999-2007	All	53,408	1,000	10.7	9.4	7.8	2.5	10.3	7.5	8.1	2.2
		By region										
		Northeast	10,598	188	8.4	6.6	6.5	2.3	7.8	4.2	6.7	1.9
		Midwest	6,076	118	10.7	7.3	8.6	2.3	10.4	5.5	8.9	1.9
		Southeast	9,215	158	8.0	5.1	6.7	2.1	7.8	3.6	6.8	1.8
		Southcentral	4,798	104	13.5	11.1	10.1	2.3	12.2	7.0	10.4	1.8
		Southwest	11,986	211	14.9	13.2	10.6	2.6	14.6	11.1	11.0	2.2
		Northwest	3,798	81	8.1	6.9	5.2	3.1	8.6	7.0	5.9	3.0
		Central Plains	6,937	140	9.8	7.4	7.0	2.7	9.4	6.2	7.2	2.3
		By network[B]										
		AQS	46,931	844	11.5	9.6	8.7	2.4	11.0	7.6	9.0	2.0
		IMPROVE	6,477	282	4.7	4.1	3.3	2.5	4.8	3.8	3.7	2.3
	1988-1998	All	8,604	233	11.1	11.0	7.7	2.3	11.4	11.0	7.6	3.7
		By region										
		Northeast	1,218	55	10.9	6.6	9.0	1.9	10.6	5.4	8.5	3.4
		Midwest	632	22	10.4	6.3	8.5	2.0	11.2	5.4	9.5	1.9
		Southeast	1,083	36	7.2	4.4	6.2	1.7	6.8	4.4	4.9	7.4
		Southcentral	513	11	7.7	5.1	6.4	1.8	7.7	5.7	6.5	1.7
		Southwest	3,800	78	15.1	14.2	10.2	2.5	15.8	14.2	10.5	3.1
		Northwest	332	9	4.2	2.7	3.5	1.9	3.9	3.5	2.3	9.2
		Central Plains	1,026	22	5.1	4.7	4.0	1.9	5.0	3.3	4.2	1.8
		By network[B]										
		IMPROVE	4,720	67	5.7	3.9	4.8	1.8	5.5	3.4	4.4	3.7
		AQS	3,415	110	18.7	13.4	15.1	2.0	19.4	13.1	15.4	2.6
		Five Cities	276	31	8.6	6.9	7.2	1.7	12.1	7.8	10.4	1.7
		24 Cities	177	16	12.1	4.8	11.1	1.5	12.0	4.6	11.2	1.4

			MOHAVE	10	8	8.0	2.5	7.5	1.5	5.3	1.8	5.0	1.5
			Six Cities	6	1	14.3	5.1	13.4	1.5	16.5	4.6	16.0	1.3
6 km grid[E]													
	$PM_{2.5}$	1999-2007	All	23,754,600	219,950	-	-	-	-	7.3	4.0	6.3	1.8
			By region			-	-	-	-				
			Northeast	2,296,188	21,261	-	-	-	-	9.1	3.5	8.5	1.5
			Midwest	2,816,100	26,075	-	-	-	-	9.9	3.3	9.3	1.4
			Southeast	3,526,632	32,654	-	-	-	-	11.9	3.5	11.4	1.3
			Southcentral	4,387,824	40,628	-	-	-	-	7.5	3.0	6.8	1.5
			Southwest	3,670,920	33,990	-	-	-	-	5.0	3.2	4.2	1.7
			Northwest	1,955,988	18,111	-	-	-	-	4.5	1.9	4.1	1.5
			Central Plains	5,100,948	47,231	-	-	-	-	4.7	2.0	4.3	1.5
		1988-1998	All	29,033,400	219,950	-	-	-	-	8.5	4.8	7.2	1.8
			By region			-	-	-	-				
			Northeast	2,806,452	21,261	-	-	-	-	11.0	4.1	10.2	1.5
			Midwest	3,441,900	26,075	-	-	-	-	11.1	3.9	10.4	1.4
			Southeast	4,310,328	32,654	-	-	-	-	13.4	4.1	12.8	1.3
			Southcentral	5,362,896	40,628	-	-	-	-	8.1	3.4	7.4	1.6
			Southwest	4,486,680	33,990	-	-	-	-	6.5	5.3	5.3	1.8
			Northwest	2,390,652	18,111	-	-	-	-	6.0	3.8	5.2	1.7
			Central Plains	6,234,492	47,231	-	-	-	-	5.4	2.5	4.8	1.6
	PM_{10}	1999-2007	All	23,754,600	219,950	-	-	-	-	14.1	6.4	12.6	1.6
			By region			-	-	-	-				

		Northeast	2,296,188	21,261	-	-	-	-	12.7	4.5	11.9	1.4
		Midwest	2,816,100	26,075	-	-	-	-	16.8	5.4	16.0	1.4
		Southeast	3,526,632	32,654	-	-	-	-	17.1	4.2	16.5	1.3
		Southcentral	4,387,824	40,628	-	-	-	-	16.5	5.7	15.4	1.5
		Southwest	3,670,920	33,990	-	-	-	-	13.5	9.0	11.2	1.9
		Northwest	1,955,988	18,111	-	-	-	-	9.7	5.0	8.4	1.7
		Central Plains	5,100,948	47,231	-	-	-	-	11.2	5.1	10.1	1.6
	1988-1998	All	29,033,400	219,950	-	-	-	-	16.4	7.5	14.7	1.6
		By region			-	-	-	-				
		Northeast	2,806,452	21,261	-	-	-	-	16.1	5.9	15.1	1.5
		Midwest	3,441,900	26,075	-	-	-	-	19.6	7.1	18.3	1.5
		Southeast	4,310,328	32,654	-	-	-	-	20.4	5.6	19.7	1.3
		Southcentral	5,362,896	40,628	-	-	-	-	17.5	6.2	16.3	1.5
		Southwest	4,486,680	33,990	-	-	-	-	16.0	10.6	13.4	1.8
		Northwest	2,390,652	18,111	-	-	-	-	13.4	6.9	11.7	1.7
		Central Plains	6,234,492	47,231	-	-	-	-	12.4	5.4	11.3	1.6
$PM_{2.5-10}$[D]	1999-2007	All	23,754,600	219,950	-	-	-	-	6.8	4.8	5.4	2.2
		By region			-	-	-	-				
		Northeast	2,296,188	21,261	-	-	-	-	3.5	2.5	3.0	2.3
		Midwest	2,816,100	26,075	-	-	-	-	7.0	4.5	5.8	2.1
		Southeast	3,526,632	32,654	-	-	-	-	5.2	2.7	4.5	1.9
		Southcentral	4,387,824	40,628	-	-	-	-	9.1	4.1	8.1	1.7
		Southwest	3,670,920	33,990	-	-	-	-	8.6	6.9	6.5	2.3
		Northwest	1,955,988	18,111	-	-	-	-	5.2	4.2	3.9	2.6
		Central Plains	5,100,948	47,231	-	-	-	-	6.5	4.2	5.3	2.1

1988-1998	All	29,033,400	219,950	-	-	-	-	7.9	4.9	6.7	1.9
	By region			-	-	-	-				
	Northeast	2,806,452	21,261	-	-	-	-	5.2	2.8	4.4	1.9
	Midwest	3,441,900	26,075	-	-	-	-	8.5	4.8	7.2	1.9
	Southeast	4,310,328	32,654	-	-	-	-	7.0	2.9	6.4	1.6
	Southcentral	5,362,896	40,628	-	-	-	-	9.4	4.1	8.5	1.6
	Southwest	4,486,680	33,990	-	-	-	-	9.5	7.5	7.6	2.1
	Northwest	2,390,652	18,111	-	-	-	-	7.3	5.5	5.7	2.6
	Central Plains	6,234,492	47,231	-	-	-	-	7.0	4.1	6.2	1.8

A: "N" is the number of monthly averages; "n sites" is the number of locations at which the monthly averages were collected.

B: See text for network names and references.

C: NA is "not available".

D: Negative or zero values of measured $PM_{2.5-10}$ were excluded from the calculation of the geometric mean and standard deviation.

E: Corresponds to model predictions at the 6 km grid locations shown in Figures 2-4.

Table S2. Bias and precision statistics from cross-validation (CV) of $PM_{2.5}$, PM_{10}, and $PM_{2.5\text{-}10}$ models overall and by season, tertiles of urban land use, monitoring network, and monitoring objective for each of two time periods: 1999-2007 and 1988-1998.

			Monthly values				
Time period	Pollutant	Group	N[A]	CV R^2	NMBF (%)[B]	CVMAE[B]	NMEF (%)[B]
1999-2007	$PM_{2.5}$	Overall	108,718	0.77	-1.6	1.61	14.3
		By season					
		Winter	26,503	0.64	-5.3	2.05	19.0
		Spring	27,104	0.80	-0.4	1.33	13.0
		Summer	27,360	0.86	0.2	1.55	11.8
		Autumn	27,751	0.78	-1.5	1.54	14.2
		By urban land use					
		Low	28,050	0.76	-2.4	1.65	14.6
		Medium	37,224	0.78	-1.7	1.63	14.4
		High	43,444	0.78	-1.1	1.57	14.1
		By network					
		AQS	101,833	0.76	-1.8	1.62	14.0
		IMPROVE	6,112	0.73	3.9	1.48	25.6
		SEARCH	556	0.85	2.6	1.27	9.4
		CASTNet	167	0.51	-0.9	2.28	20.1
		By monitoring objective[C]					
		Population exposure	83,282	0.74	-1.6	1.62	13.5
		General / background	11,761	0.83	0.0	1.40	17.7
		Highest concentration	5,100	0.66	-3.6	1.73	13.4

		Other / missing	8,575	0.73	-2.3	1.76	22.9
PM_{10}	Overall		104,509	0.58	-5.1	5.21	24.4
		By season					
		Winter	25,907	0.54	-10.4	5.66	30.8
		Spring	26,542	0.56	-4.2	5.00	23.9
		Summer	26,135	0.58	-1.9	4.97	20.4
		Autumn	25,925	0.62	-5.0	5.21	23.8
		By urban land use					
		Low	29,993	0.58	-5.7	4.98	23.7
		Medium	34,526	0.55	-4.1	5.34	24.6
		High	40,050	0.61	-5.4	5.27	24.7
		By network					
		AQS	92,770	0.54	-6.3	5.44	24.1
		IMPROVE	11,739	0.50	15.0	3.41	32.9
		By monitoring objective[C]					
		Population exposure	45,009	0.50	-3.8	5.19	22.1
		General / background	5,423	0.59	-3.3	4.75	25.3
		Highest concentration	22,476	0.50	-12.7	5.92	25.3
		Other / missing	31,601	0.64	-0.6	4.80	27.6
$PM_{2.5-10}$[D]	Overall		41,098	0.52	-3.2	4.18	38.9
		By season					
		Winter	9,342	0.44	-10.8	4.26	51.6
		Spring	10,620	0.49	-2.3	4.11	37.3
		Summer	10,550	0.54	0.9	4.10	34.2
		Autumn	10,586	0.55	-3.5	4.25	37.8

		By urban land use						
		Low	9,621	0.58	-4.7	5.27	37.3	
		Medium	14,008	0.45	-1.6	4.01	38.5	
		High	17,469	0.39	-3.3	3.71	40.7	
		By network						
		AQS	36,093	0.51	-5.3	4.35	38.4	
		IMPROVE	5,005	0.29	31.3	2.96	59.3	
		By monitoring objective[C]						
		Population exposure	29,567	0.51	-5.7	4.46	37.7	
		General / background	5,278	0.40	18.1	2.72	47.0	
		Highest concentration	1,610	0.42	-25.8	5.94	46.6	
		Other / missing	4,643	0.34	19.4	3.42	54.7	
1988-1998	$PM_{2.5}$[E]	Overall	10,823	0.77	-0.8	1.81	14.8	
		By season						
		Winter	2,518	0.68	-1.4	2.27	17.5	
		Spring	2,684	0.79	0.1	1.45	13.5	
		Summer	2,775	0.81	0.1	1.66	12.7	
		Autumn	2,846	0.78	-2.0	1.90	15.7	
		By urban land use						
		Low	3,345	0.73	-1.7	1.86	15.2	
		Medium	3,682	0.79	0.5	1.79	14.4	
		High	3,796	0.78	-1.4	1.79	15.0	
		By network						
		AQS	9,888	0.73	-0.8	1.86	14.6	

		IMPROVE	806	0.80	-0.7	1.16	21.8
		SEARCH	85	0.78	1.7	1.72	11.6
		CASTNet	44	0.67	-6.1	2.11	19.6
		By monitoring objective[C]					
		Population exposure	8,207	0.72	-0.8	1.88	14.5
		General / background	611	0.81	2.0	1.54	14.6
		Highest concentration	567	0.69	-2.9	1.91	13.4
		Other / missing	1,438	0.84	-1.0	1.50	19.1
PM_{10}	Overall		145,398	0.58	-3.3	5.44	21.8
		By season					
		Winter	35,448	0.55	-6.8	6.16	26.6
		Spring	36,380	0.52	-2.7	5.07	21.6
		Summer	36,605	0.62	-0.8	5.13	18.1
		Autumn	36,965	0.62	-3.4	5.42	21.7
		By urban land use					
		Low	39,274	0.57	-4.5	5.35	21.5
		Medium	47,591	0.58	-3.0	5.50	21.6
		High	58,533	0.59	-2.6	5.45	22.0
		By network					
		AQS	140,287	0.57	-3.5	5.47	21.5
		IMPROVE	4,655	0.29	8.1	4.50	37.2
		5 Cities	261	0.53	14.8	5.64	23.0
		24 Cities	177	0.52	-3.8	4.92	18.0
		MOHAVE	11	0.64	-40.6	4.64	45.1
		6 Cities	6	0.52	7.9	5.67	20.6
		SEAVS	1	NA[F]	NA[F]	NA[F]	NA[F]

	By monitoring objective[C]					
	Population exposure	54,194	0.54	-0.9	5.21	19.4
	General / background	4,477	0.74	1.9	4.41	20.6
	Highest concentration	39,794	0.54	-6.9	5.72	21.0
	Other / missing	46,933	0.59	-3.2	5.56	26.2
$PM_{2.5-10}$[D, F]	Overall	4,032	0.45	-4.7	4.73	42.6
	By season					
	Winter	864	0.31	-15.9	5.34	63.3
	Spring	1,046	0.45	-3.0	4.67	38.0
	Summer	1,038	0.55	-0.5	4.40	35.0
	Autumn	1,084	0.47	-4.0	4.61	43.1
	By urban land use					
	Low	1,179	0.53	-4.3	5.60	40.4
	Medium	1,383	0.36	-3.9	4.26	42.5
	High	1,470	0.34	-5.8	4.46	45.1
	By network					
	AQS	3,396	0.42	-6.6	5.06	41.8
	IMPROVE	636	0.18	21.3	2.97	62.3
	By monitoring objective[C]					
	Population exposure	2,871	0.44	-5.3	5.01	41.3
	General / background	148	0.37	6.2	5.27	46.7
	Highest concentration	264	0.26	-20.3	5.27	43.2
	Other / missing	749	0.31	7.0	3.36	54.5

A: Includes data from CV sets one through nine; exclusions are the same as in Table 1 for 1999-2007 and Table 2 for 1988-1998; see text for details.

B: NMBF is normalized mean bias factor; CVMAE is cross-validation mean absolute error; NMEF is normalized mean error factor; see text for details.

C: Categories of monitoring objective were collapsed as follows: "Extreme downwind", "Source oriented", and "Maximum precursor emissions impact" were assigned to "Highest concentration; "Regional transport" and "Upwind background" were assigned to "General / background"; "Welfare related impacts", "Maximum ozone concentration", "Quality assurance", and missing monitoring objective were assigned to "Other / missing".

D: Calculated as the difference between monthly PM_{10} and $PM_{2.5}$ measurements and, separately, monthly PM_{10} and $PM_{2.5}$ model predictions.

E: Measured and predicted levels (rather than the natural-log of the $PM_{2.5}$ to PM_{10} ratio) were compared.

F: NA is "not available" due to too little data.

Figures

Figure S1. Penalized spline smooth functions of geographic and meteorological covariate effects for each of the PM models: A1-A11) 1999-2007 $PM_{2.5}$, B1-B6) 1988-1998 $PM_{2.5}$, and C1-C12) 1988-2007 PM_{10}. Regions correspond to those in Figure 1, and are drawn in order from top to bottom of the legend. Therefore, some region-specific smooth functions are behind others (especially for B2, where only the Northwest function differed from that for the other regions). For each model, covariates are listed in decreasing order of the range of the percent change in predicted PM (note the scale of the left axis is not constant across plots).

A1)

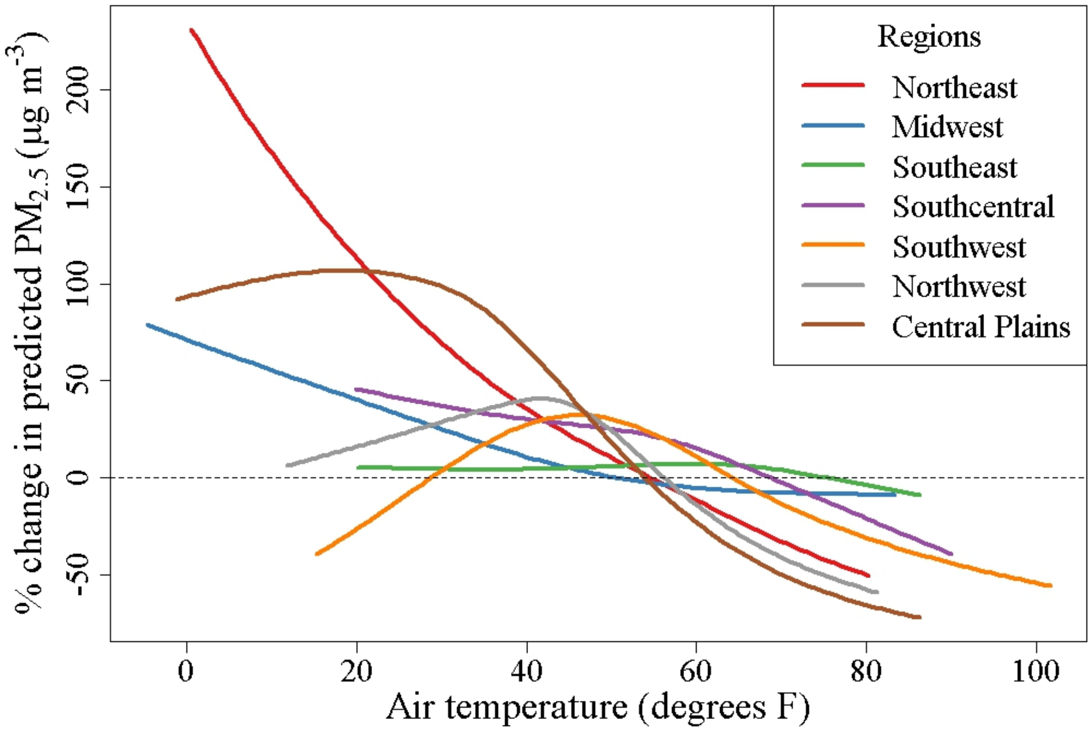

A2)

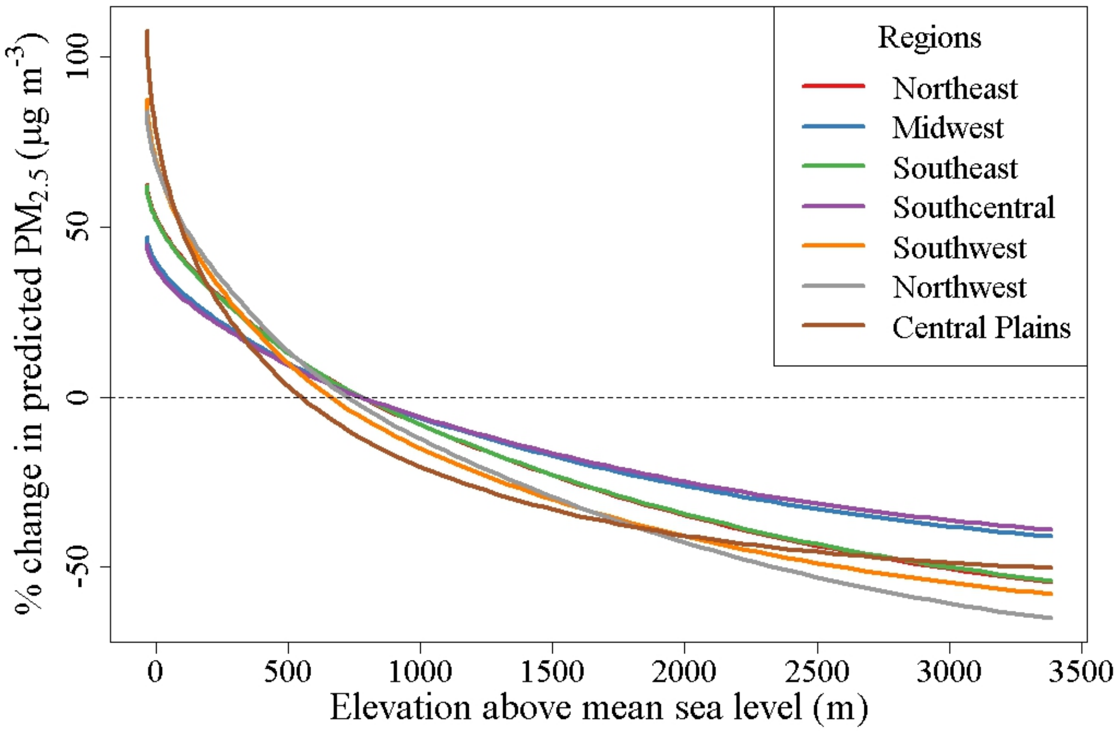

Regions
Northeast
Midwest
Southeast
Southcentral
Southwest
Northwest
Central Plains
% change in predicted $PM_{2.5}$ ($\mu g\ m^{-3}$)
100
50
0
-50
0
500
1000
1500
2000
2500
3000
3500
Elevation above mean sea level (m)

A3)

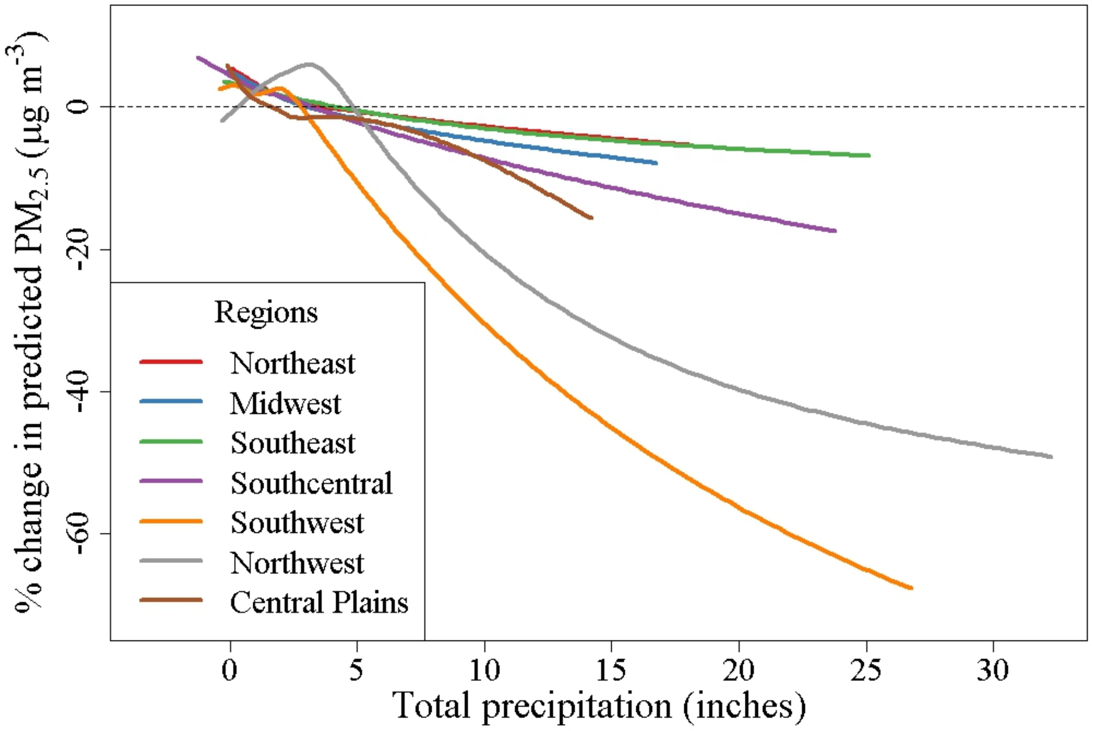

% change in predicted PM2.5 (µg m-3)
0
-20
-40
-60
Regions
Northeast
Midwest
Southeast
Southcentral
Southwest
Northwest
Central Plains
0
5
10
15
20
25
30
Total precipitation (inches)

A4)

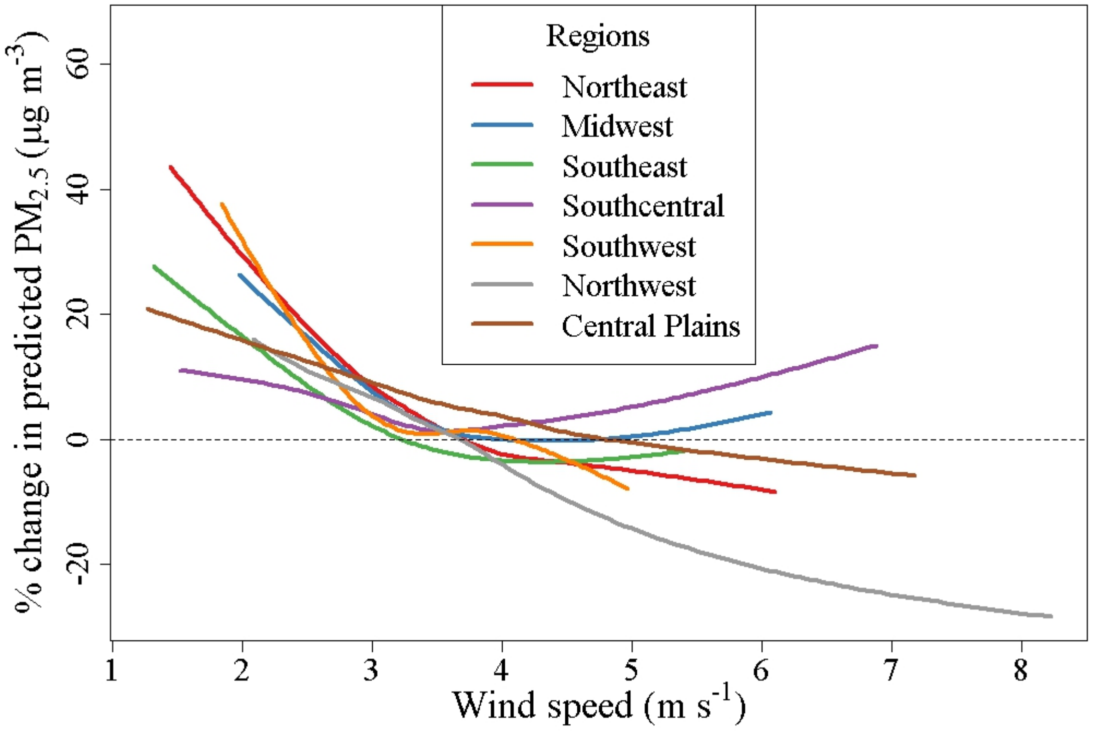

Regions
Northeast
Midwest
Southeast
Southcentral
Southwest
Northwest
Central Plains
% change in predicted PM2.5 (μg m-3)
60
40
20
0
-20
1
2
3
4
5
6
7
8
Wind speed (m s-1)

A5)

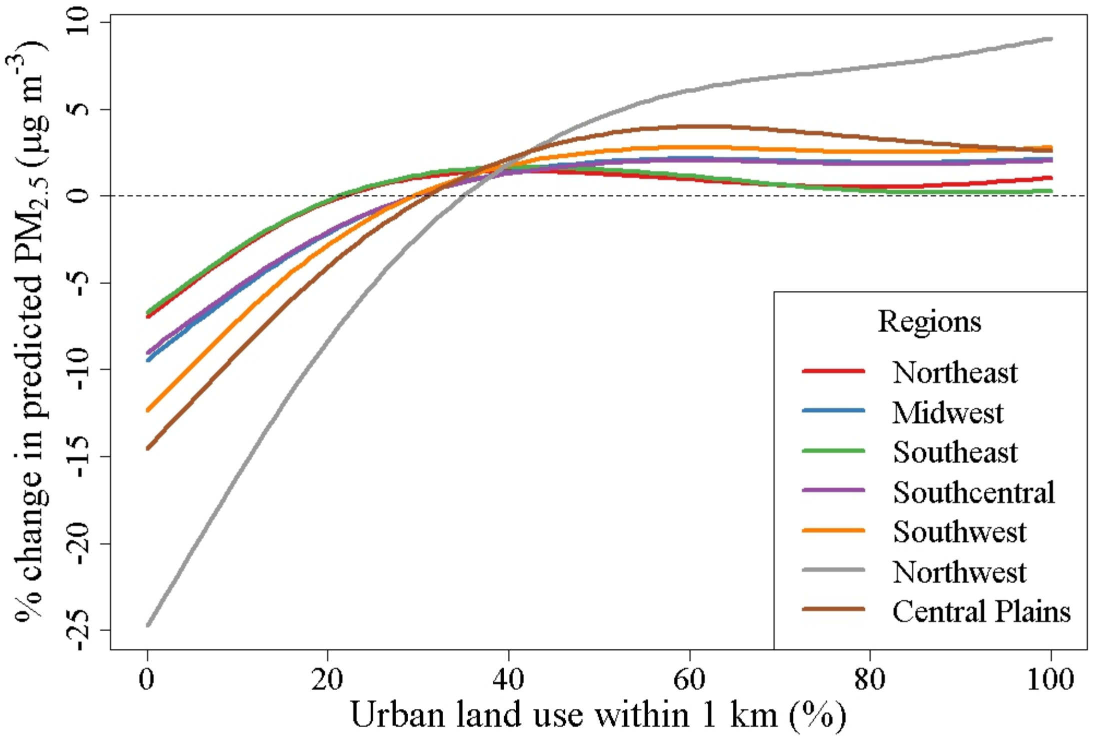

% change in predicted PM2.5 (µg m-3)
10
5
0
-5
-10
-15
-20
-25
0
20
40
60
80
100
Urban land use within 1 km (%)
Regions
Northeast
Midwest
Southeast
Southcentral
Southwest
Northwest
Central Plains

A6)

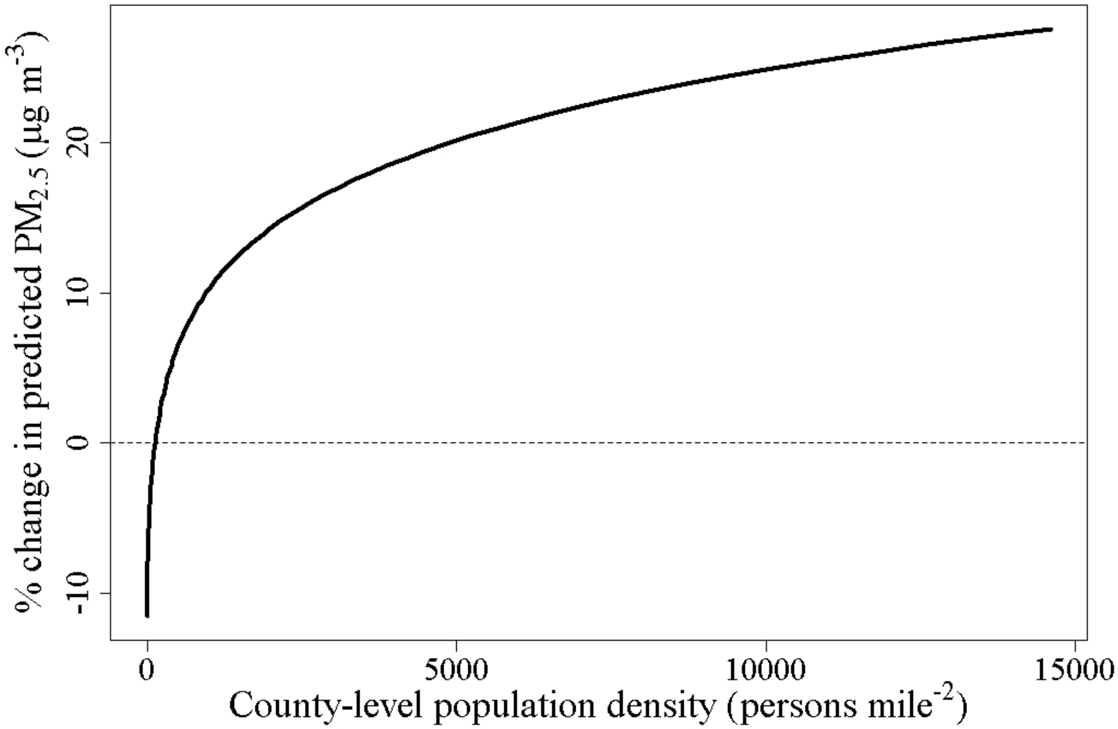

% change in predicted $PM_{2.5}$ ($\mu g\ m^{-3}$)
-10
0
10
20
0
5000
10000
15000
County-level population density (persons $mile^{-2}$)

A7)

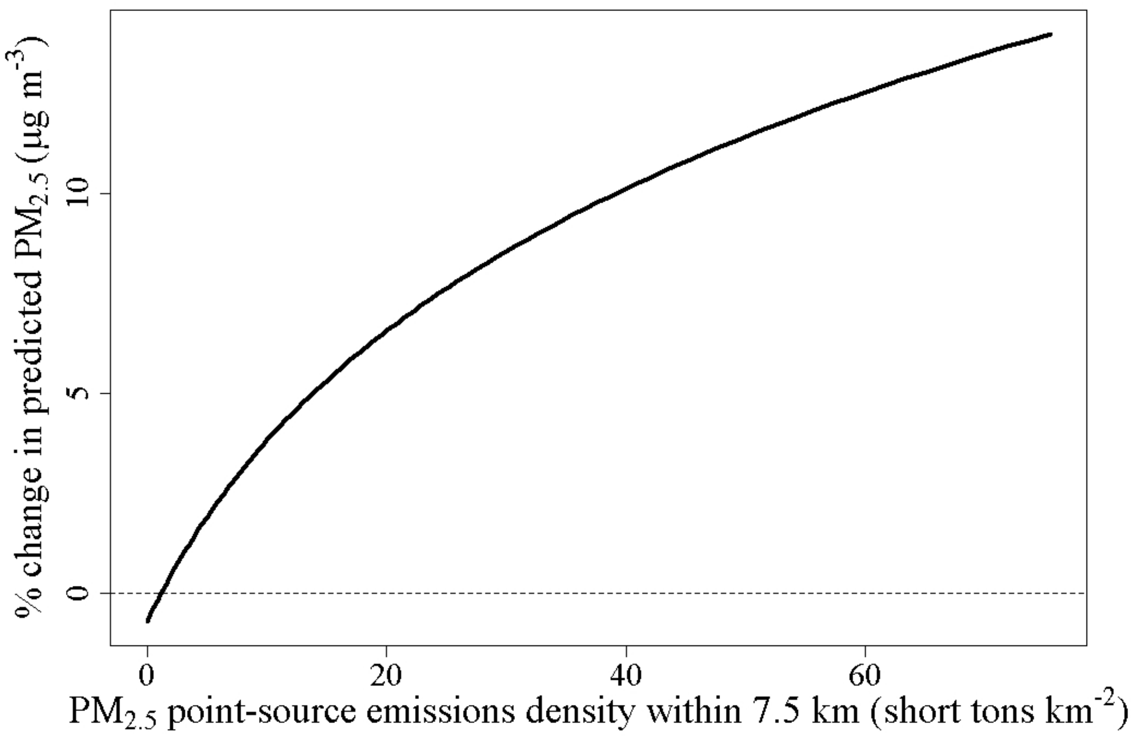

% change in predicted PM2.5 (µg m-3)
0
5
10
0
20
40
60
PM2.5 point-source emissions density within 7.5 km (short tons km-2)

A8)

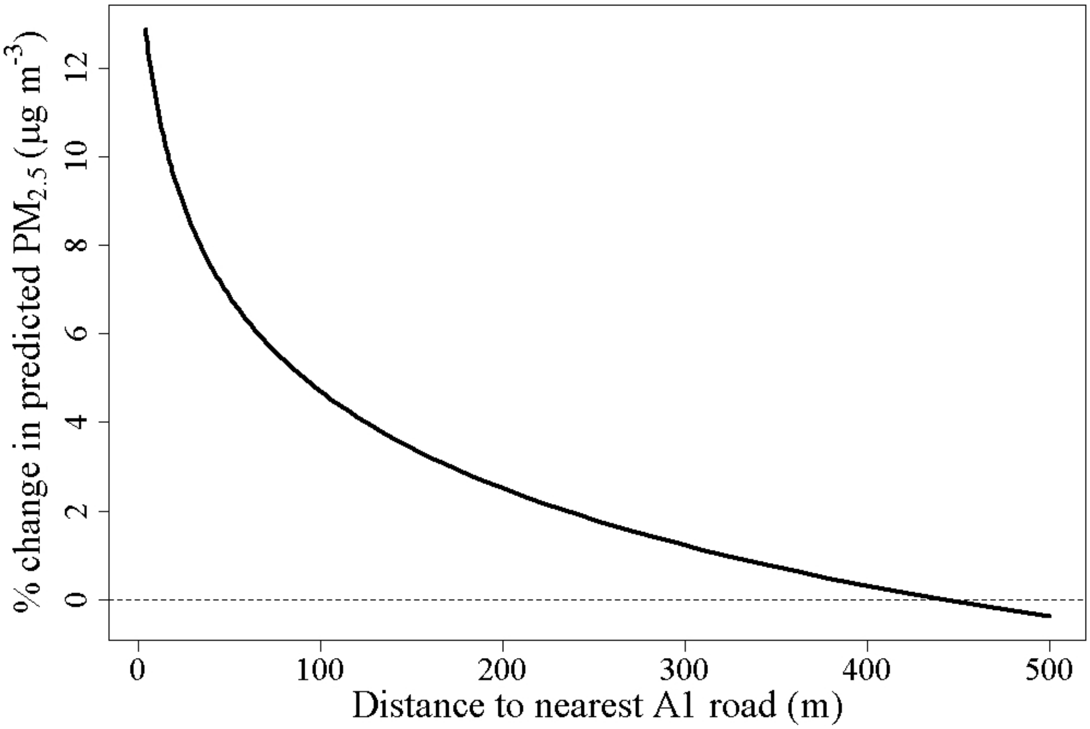

% change in predicted $PM_{2.5}$ (µg m^{-3})
0
2
4
6
8
10
12
0
100
200
300
400
500
Distance to nearest A1 road (m)

A9)

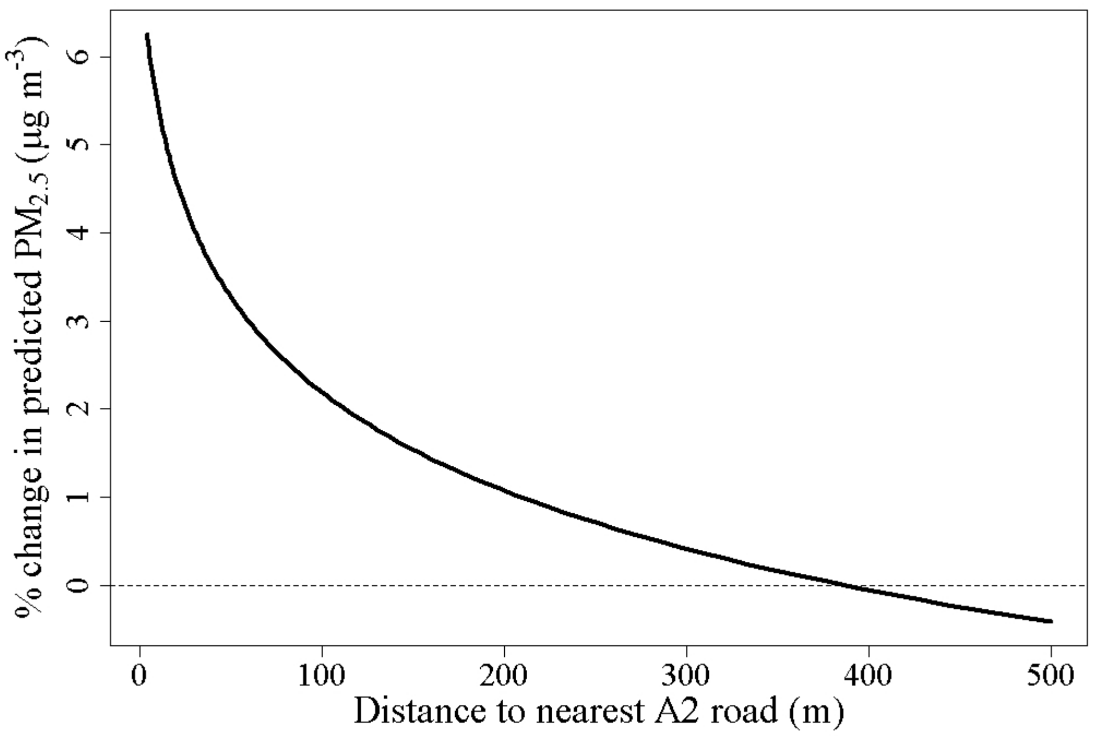

% change in predicted $PM_{2.5}$ (μg m^{-3})
0
1
2
3
4
5
6
0
100
200
300
400
500
Distance to nearest A2 road (m)

A10)

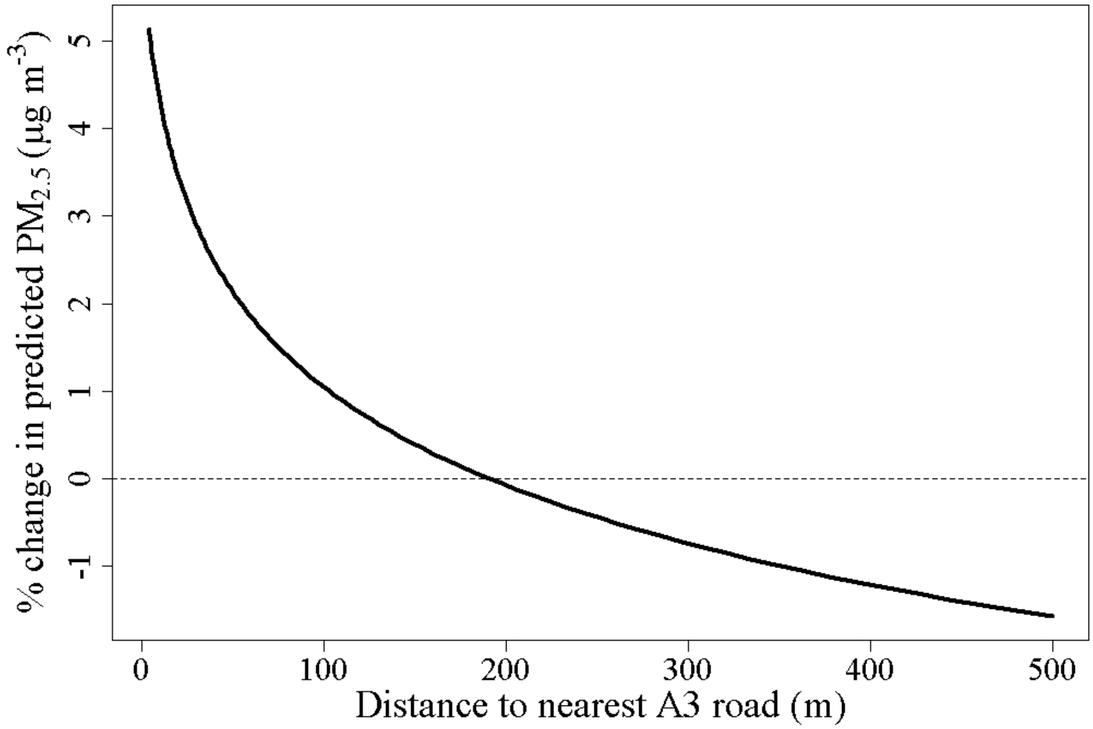

% change in predicted PM2.5 (µg m-3)
5
4
3
2
1
0
-1
0
100
200
300
400
500
Distance to nearest A3 road (m)

A11)

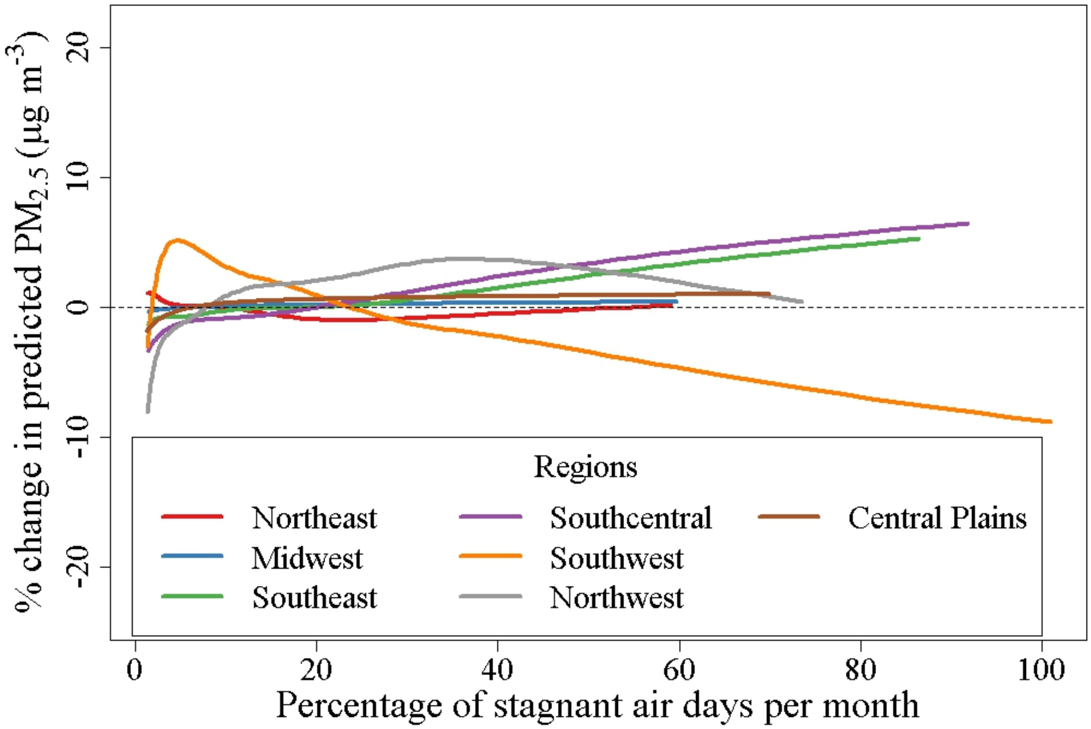

% change in predicted $PM_{2.5}$ (μg m^{-3})
20
10
0
-10
-20
Regions
Northeast
Midwest
Southeast
Southcentral
Southwest
Northwest
Central Plains
0
20
40
60
80
100
Percentage of stagnant air days per month

B1)

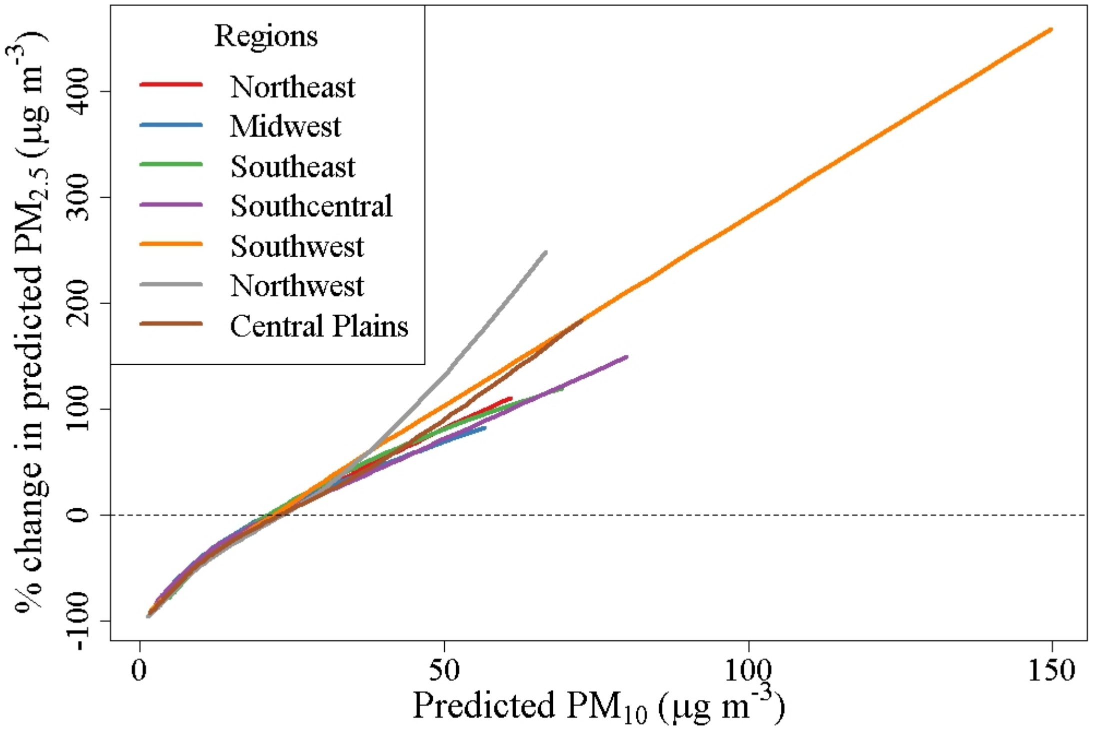

Regions
Northeast
Midwest
Southeast
Southcentral
Southwest
Northwest
Central Plains
% change in predicted PM2.5 (µg m-3)
-100
0
100
200
300
400
0
50
100
150
Predicted PM10 (µg m-3)

B2)

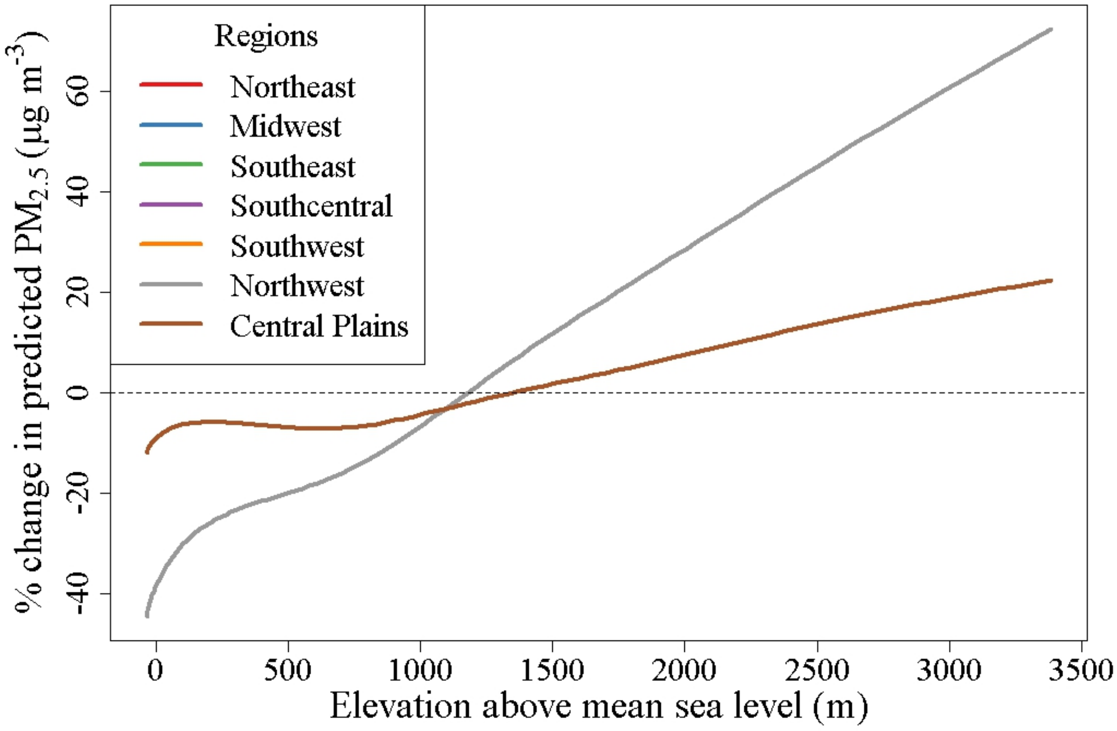
Regions
Northeast
Midwest
Southeast
Southcentral
Southwest
Northwest
Central Plains
% change in predicted $PM_{2.5}$ ($\mu g\ m^{-3}$)
-40
-20
0
20
40
60
0
500
1000
1500
2000
2500
3000
3500
Elevation above mean sea level (m)

B3)

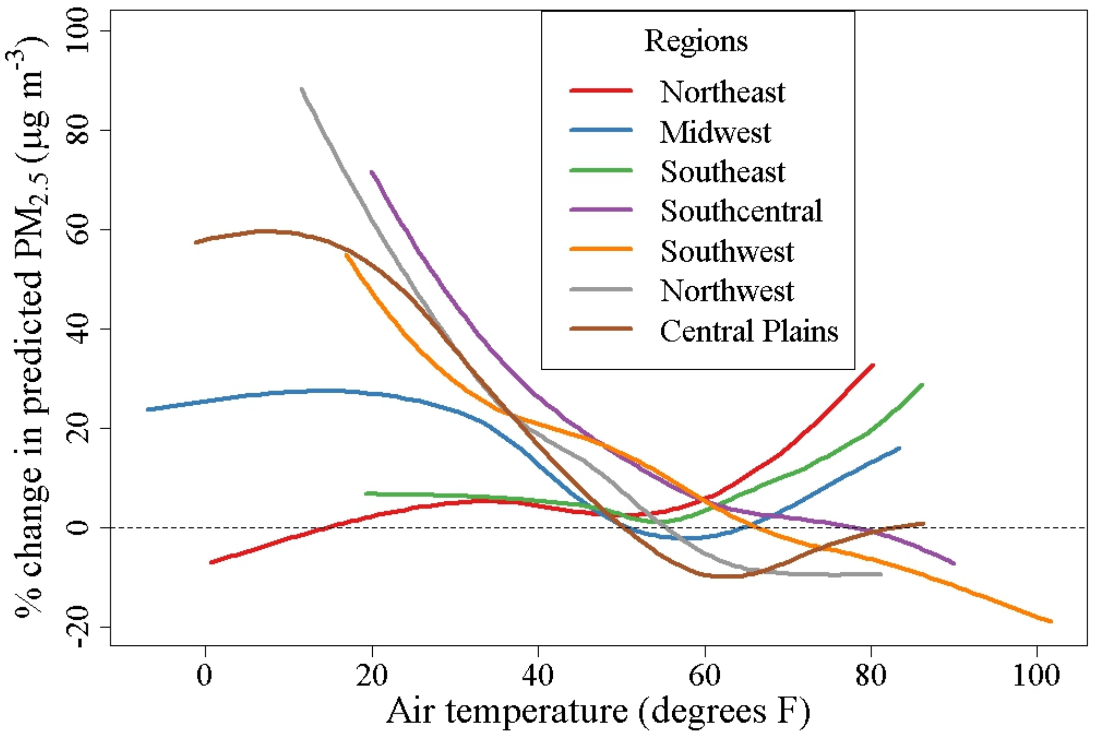
Regions
Northeast
Midwest
Southeast
Southcentral
Southwest
Northwest
Central Plains
% change in predicted PM2.5 (µg m-3)
100
80
60
40
20
0
-20
0
20
40
60
80
100
Air temperature (degrees F)

B4)

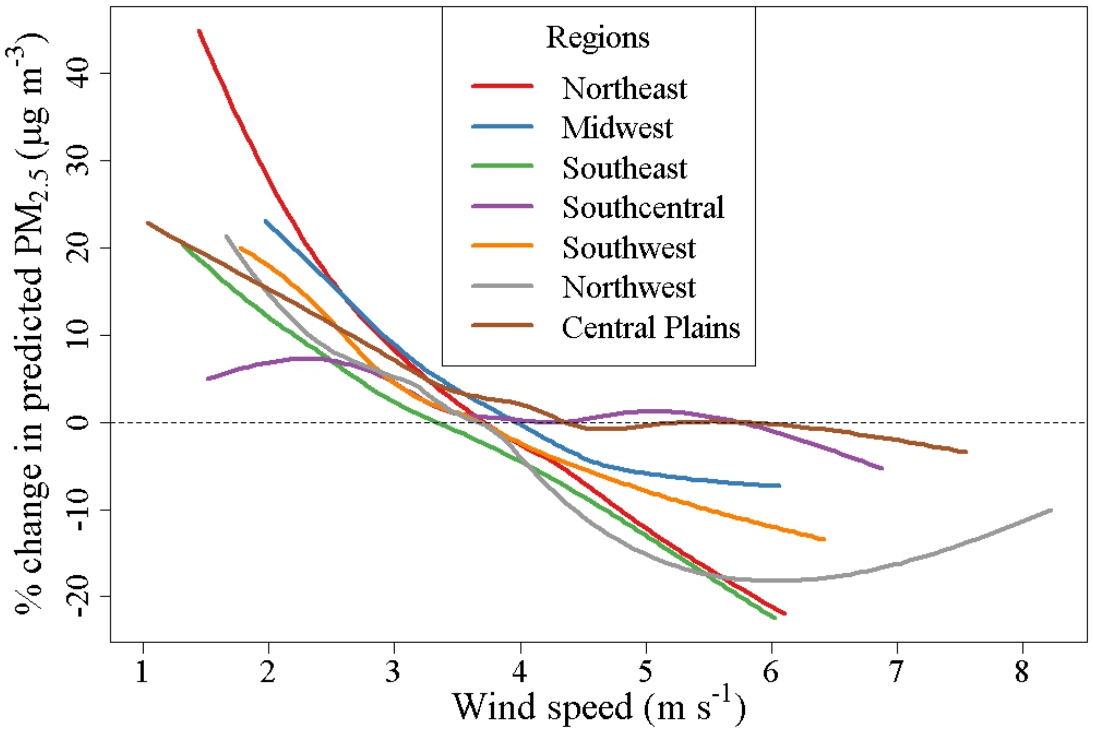

Regions
Northeast
Midwest
Southeast
Southcentral
Southwest
Northwest
Central Plains
% change in predicted PM2.5 (µg m-3)
40
30
20
10
0
-10
-20
1
2
3
4
5
6
7
8
Wind speed (m s-1)

B5)

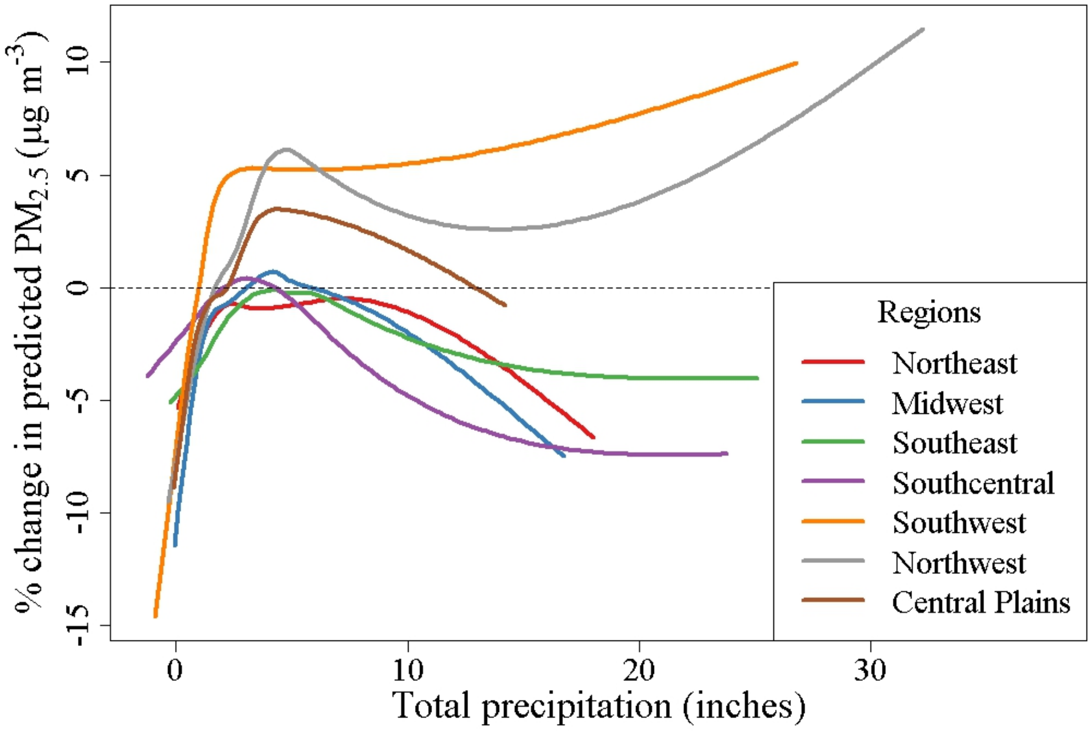
% change in predicted PM2.5 (μg m-3)
10
5
0
-5
-10
-15
0
10
20
30
Total precipitation (inches)
Regions
Northeast
Midwest
Southeast
Southcentral
Southwest
Northwest
Central Plains

B6)

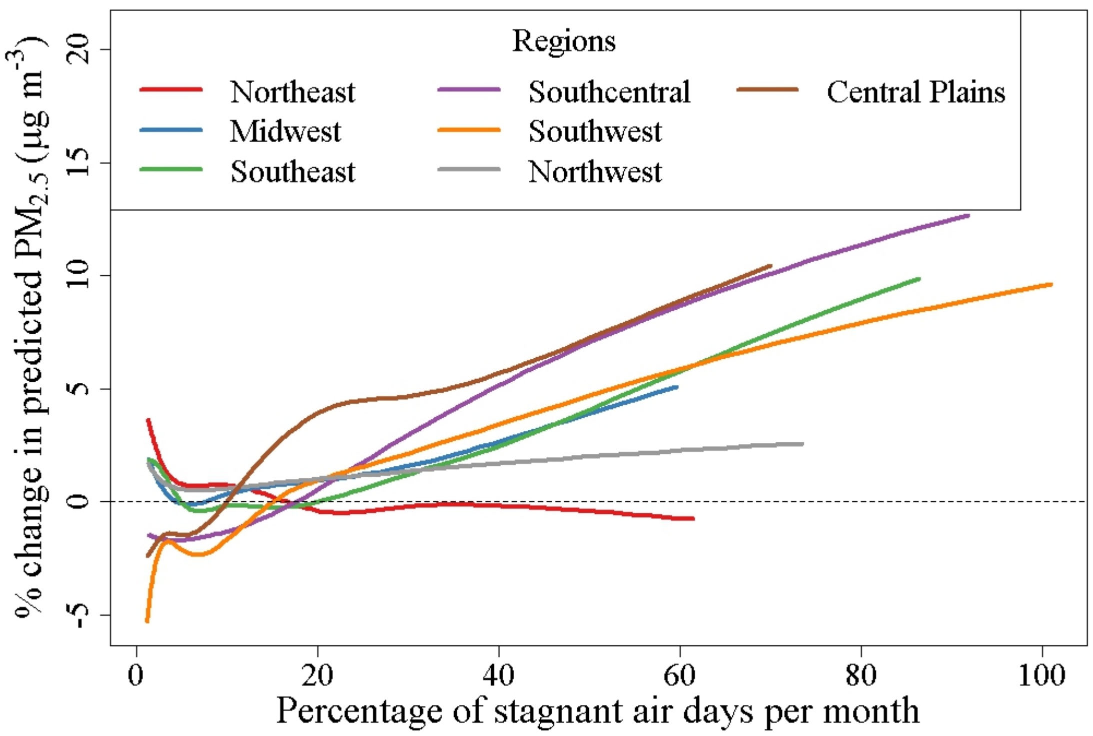
Regions
Northeast
Midwest
Southeast
Southcentral
Southwest
Northwest
Central Plains
% change in predicted $PM_{2.5}$ (µg m^{-3})
20
15
10
5
0
-5
0
20
40
60
80
100
Percentage of stagnant air days per month

C1)

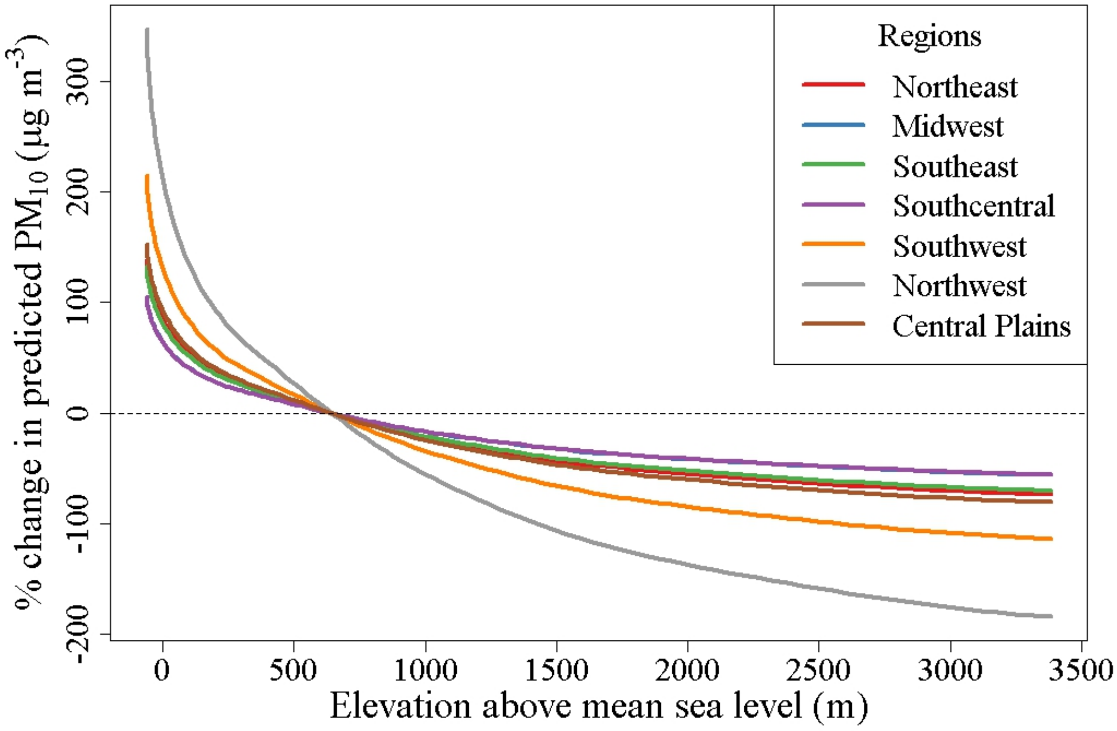

Regions
Northeast
Midwest
Southeast
Southcentral
Southwest
Northwest
Central Plains
% change in predicted PM10 (µg m-3)
-200
-100
0
100
200
300
0
500
1000
1500
2000
2500
3000
3500
Elevation above mean sea level (m)

C2)

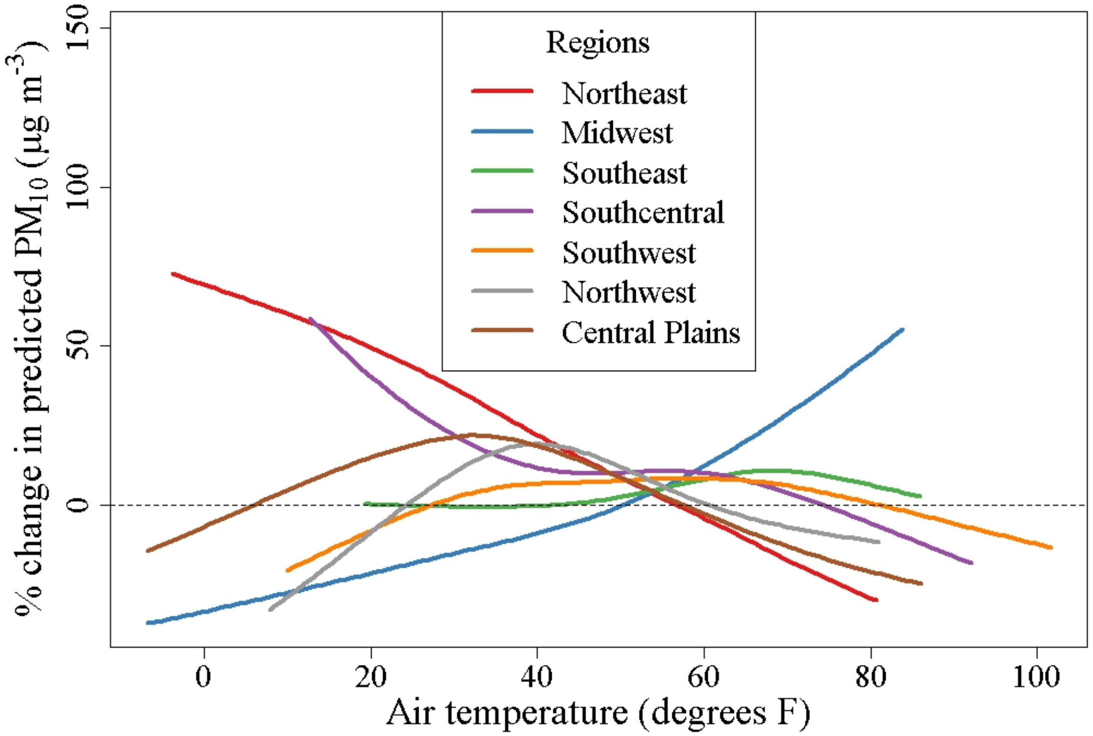

Regions
Northeast
Midwest
Southeast
Southcentral
Southwest
Northwest
Central Plains
% change in predicted PM10 (µg m-3)
150
100
50
0
0
20
40
60
80
100
Air temperature (degrees F)

C3)

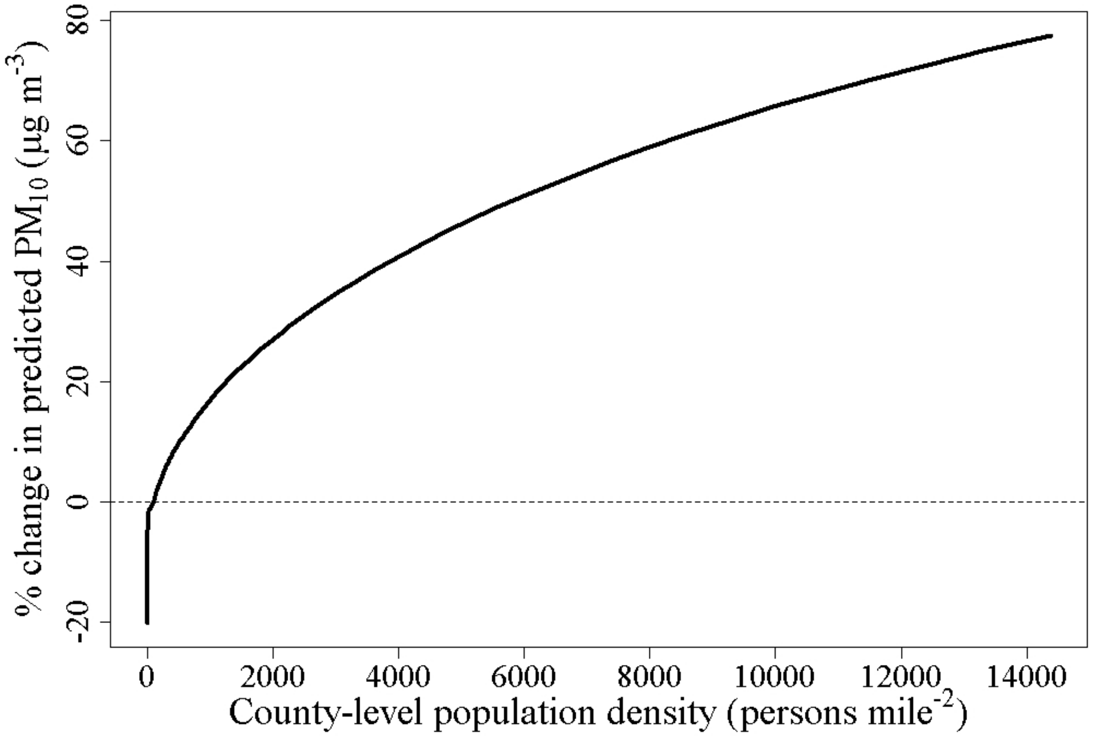

80
60
40
20
0
-20
% change in predicted PM10 (µg m-3)
0
2000
4000
6000
8000
10000
12000
14000
County-level population density (persons mile-2)

C4)

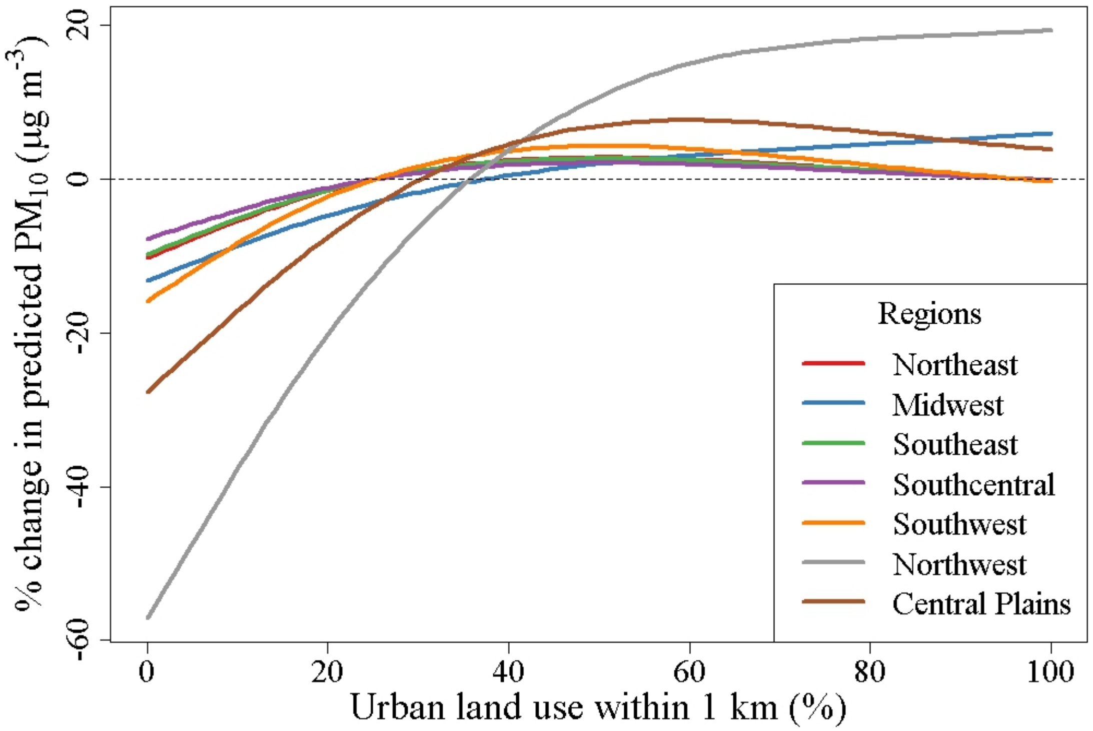
% change in predicted PM10 (µg m-3)
20
0
-20
-40
-60
0
20
40
60
80
100
Urban land use within 1 km (%)
Regions
Northeast
Midwest
Southeast
Southcentral
Southwest
Northwest
Central Plains

C5)

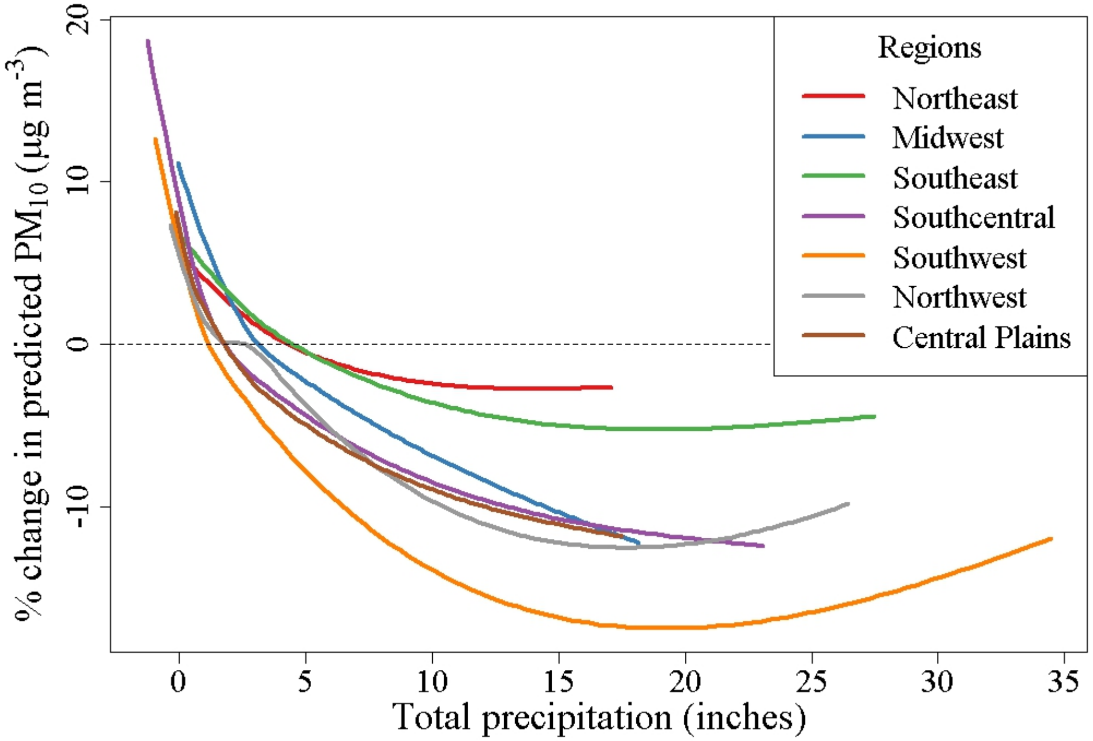
Regions
Northeast
Midwest
Southeast
Southcentral
Southwest
Northwest
Central Plains
% change in predicted PM_{10} ($\mu g\ m^{-3}$)
20
10
0
-10
0
5
10
15
20
25
30
35
Total precipitation (inches)

C6)

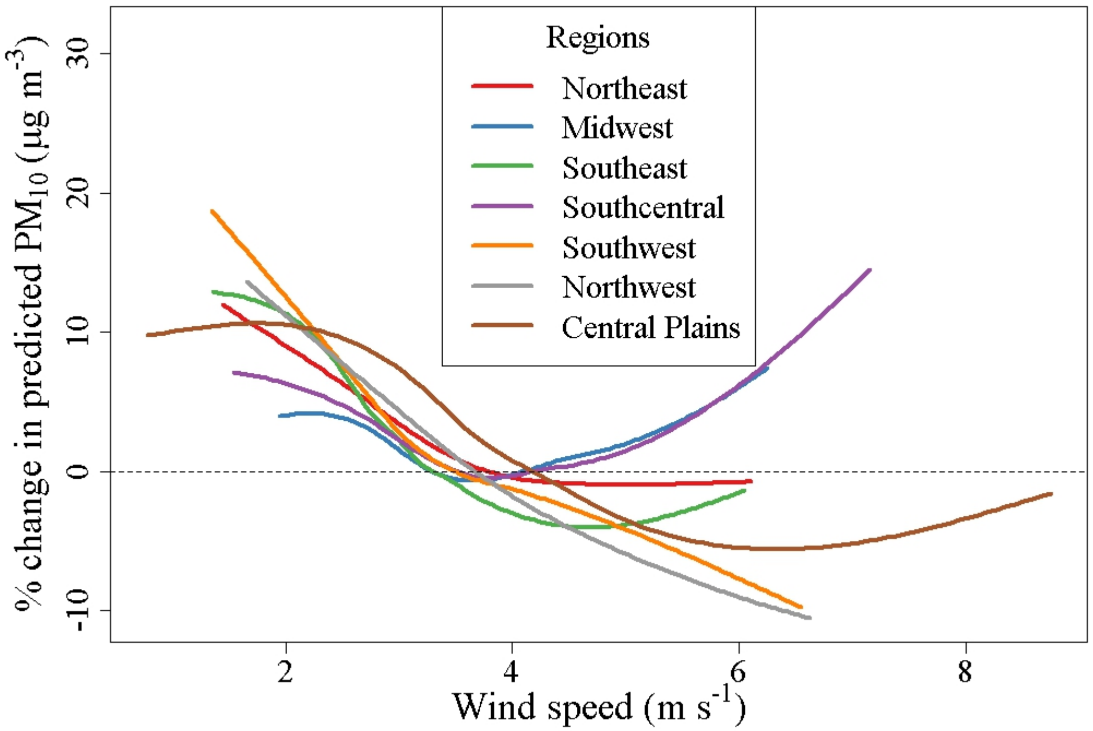

Regions
Northeast
Midwest
Southeast
Southcentral
Southwest
Northwest
Central Plains
% change in predicted PM_{10} ($\mu g\ m^{-3}$)
30
20
10
0
-10
2
4
6
8
Wind speed ($m\ s^{-1}$)

C7)

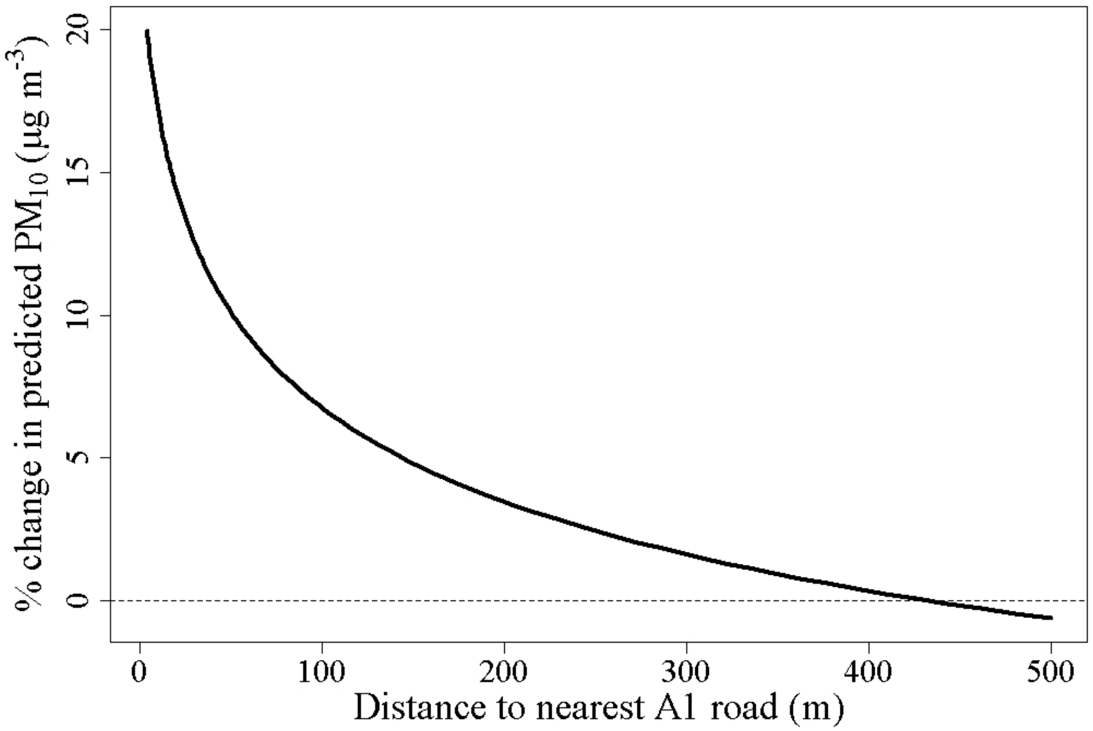

% change in predicted PM10 (µg m-3)
20
15
10
5
0
0
100
200
300
400
500
Distance to nearest A1 road (m)

C8)

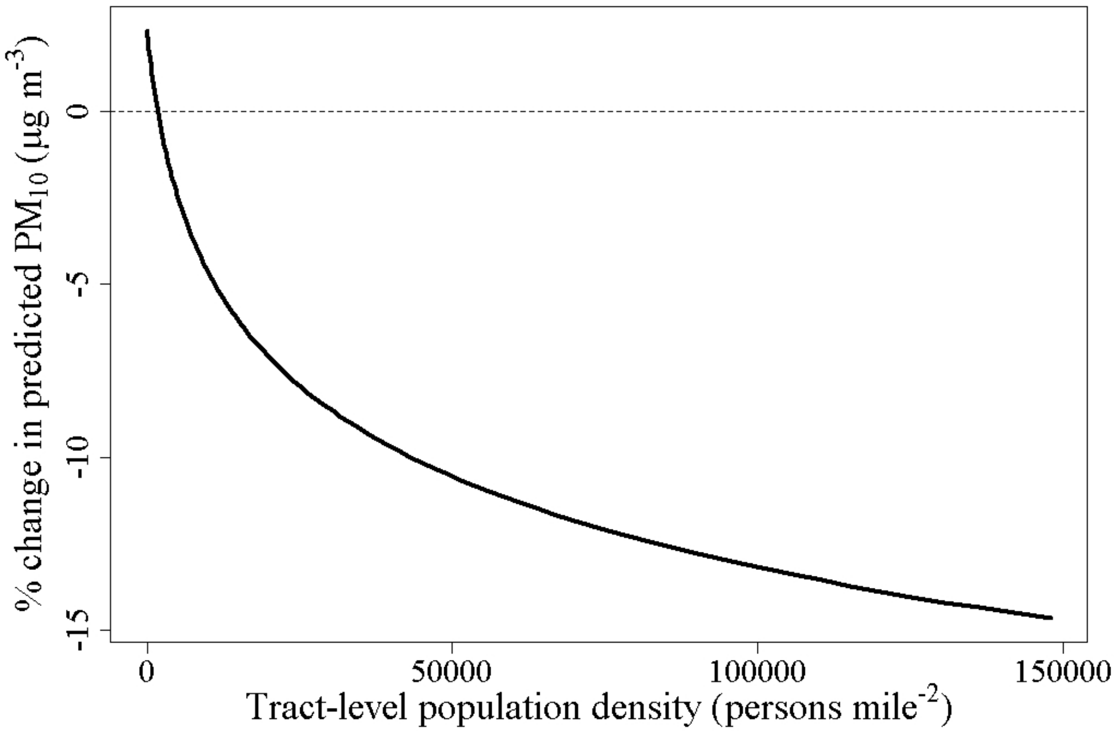
% change in predicted PM_{10} (µg m^{-3})
0
-5
-10
-15
0
50000
100000
150000
Tract-level population density (persons $mile^{-2}$)

C9)

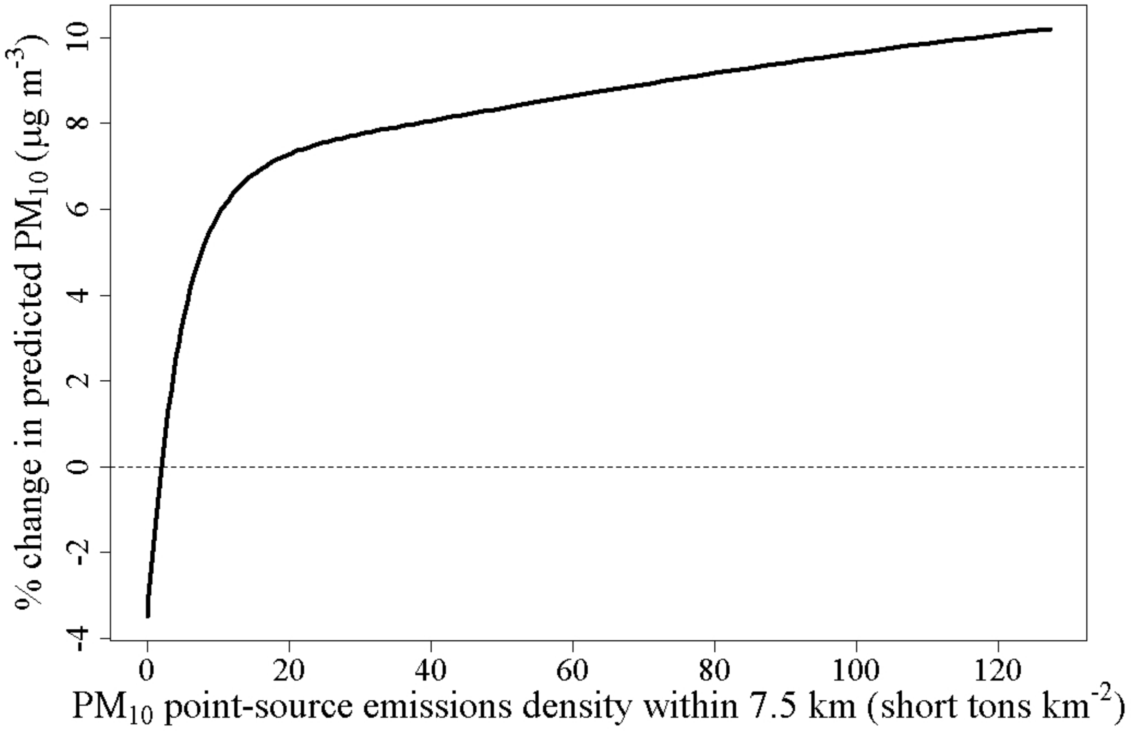
% change in predicted PM10 (µg m-3)
10
8
6
4
2
0
-2
-4
0
20
40
60
80
100
120
PM10 point-source emissions density within 7.5 km (short tons km-2)

C10)

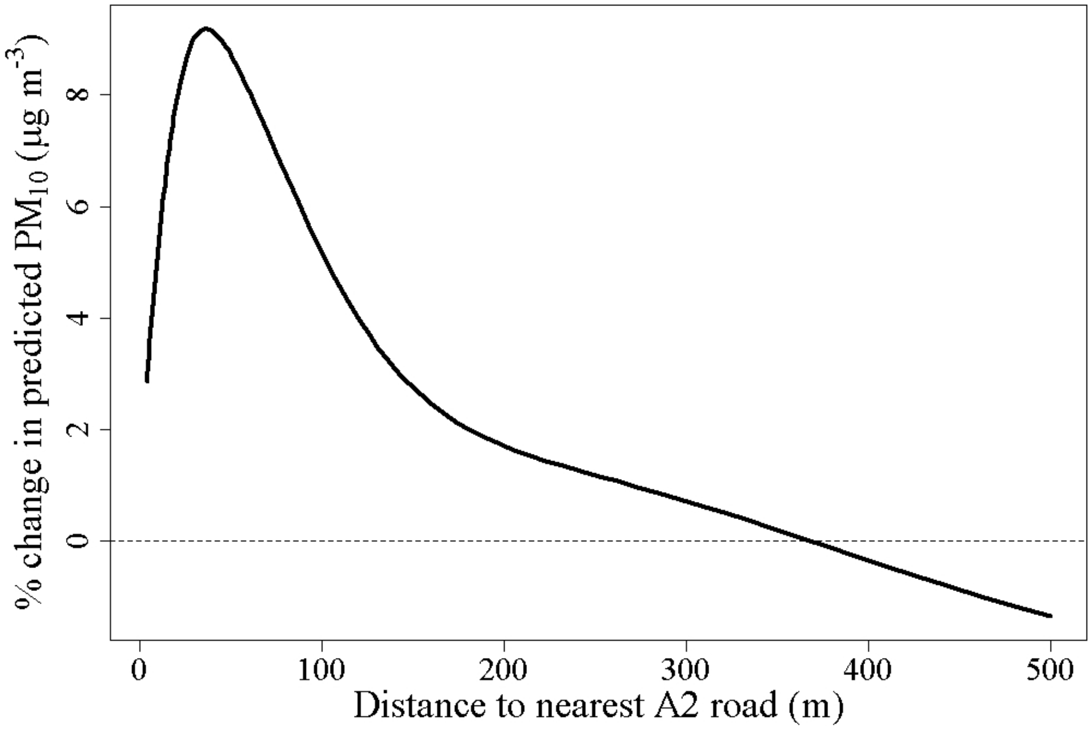

% change in predicted PM10 (µg m-3)
8
6
4
2
0
0
100
200
300
400
500
Distance to nearest A2 road (m)

C11)

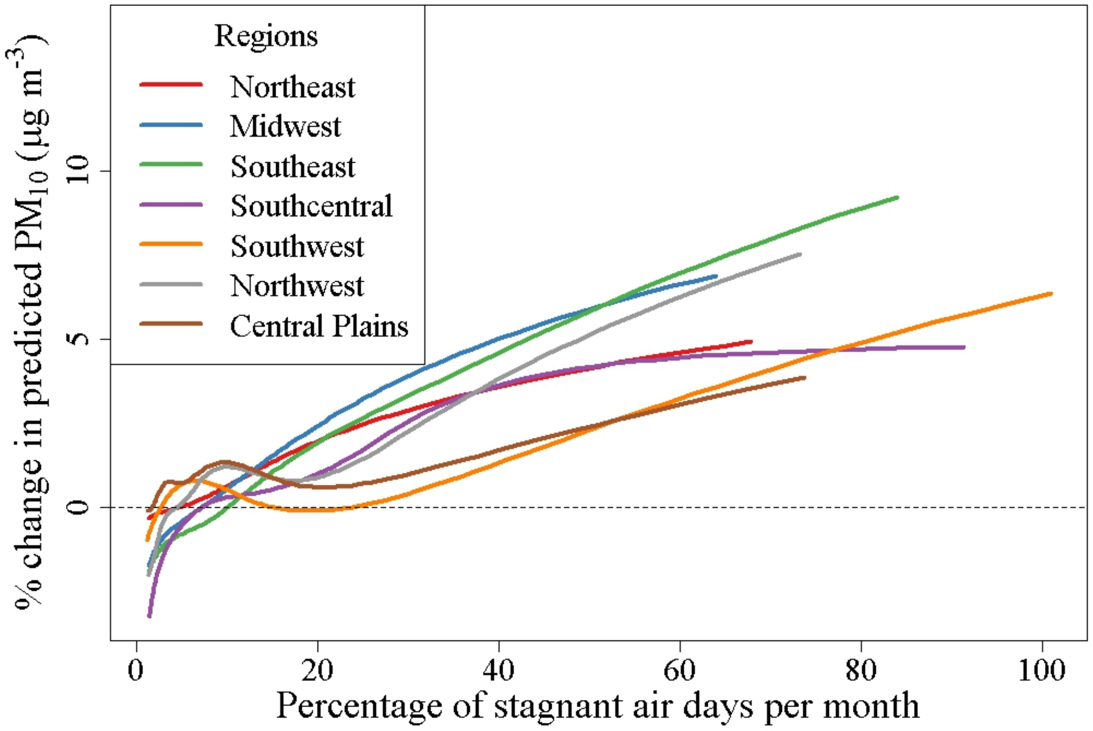

Regions
Northeast
Midwest
Southeast
Southcentral
Southwest
Northwest
Central Plains
% change in predicted PM_{10} ($\mu g\ m^{-3}$)
0
5
10
0
20
40
60
80
100
Percentage of stagnant air days per month

C12)

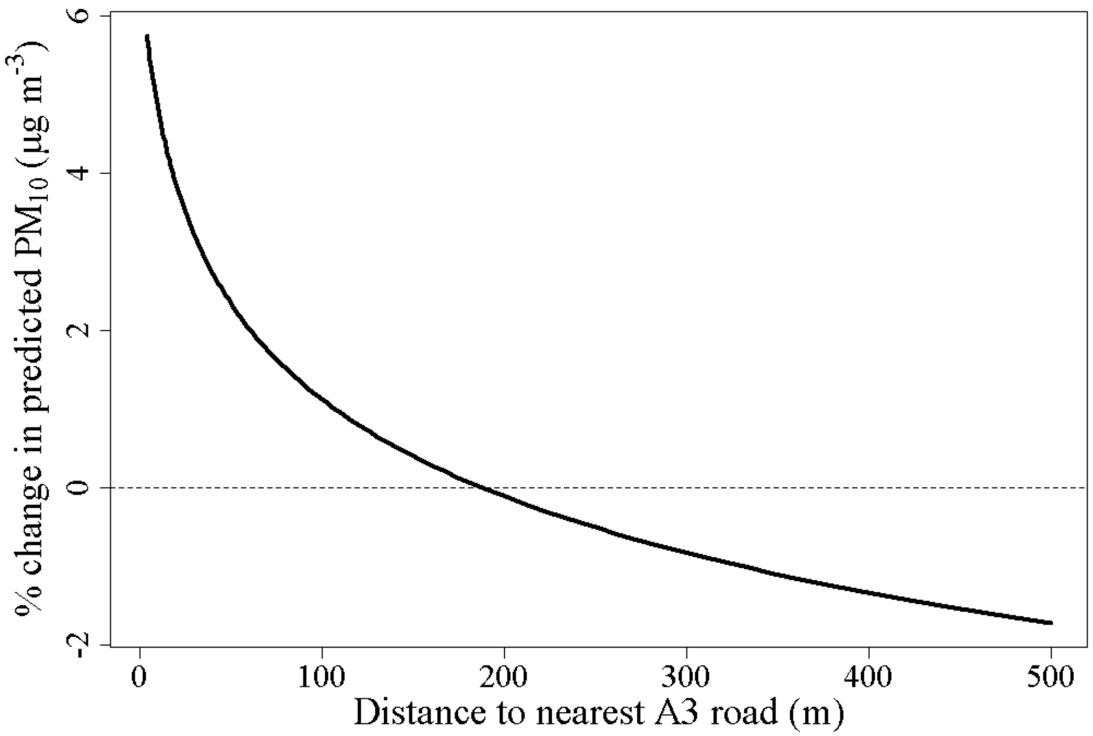

% change in predicted PM_{10} (µg m^{-3})
6
4
2
0
-2
0
100
200
300
400
500
Distance to nearest A3 road (m)

Figure S2. Density scatter plots of measured monthly-average PM levels *vs.* model predictions from cross-validation sets 1-9 for: A) the 1999-2007 $PM_{2.5}$ model, B) the 1988-1998 $PM_{2.5}$ model (using cross-validation data from the year 2000), C) the 1988-2007 PM_{10} model, D) $PM_{2.5\text{-}10}$ from 1999-2007, and E) $PM_{2.5\text{-}10}$ for the year 2000 (using the 1988-1998 $PM_{2.5}$ model). Solid lines are 1:1 lines; dotted lines are the regression lines fit to the natural-log transformed data, after transformation to the original scale by exponentiation.

A)

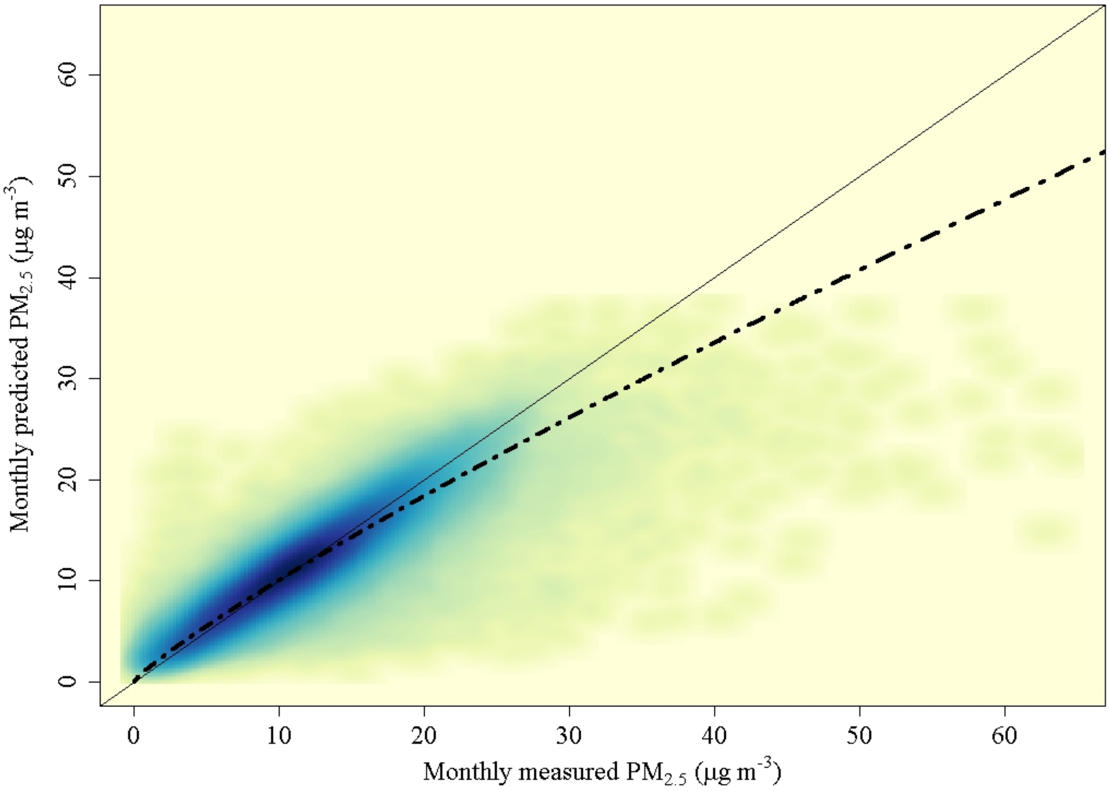

B)

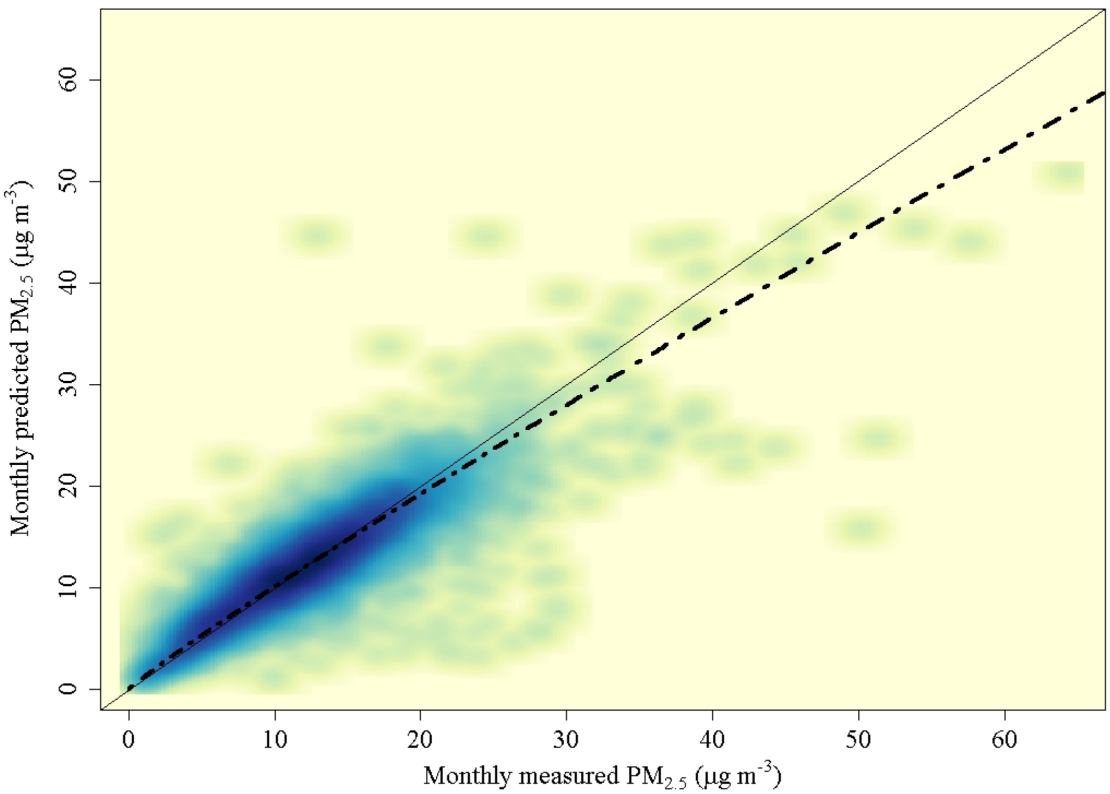

Monthly predicted PM2.5 (μg m-3)
0
10
20
30
40
50
60
Monthly measured PM2.5 (μg m-3)

C)

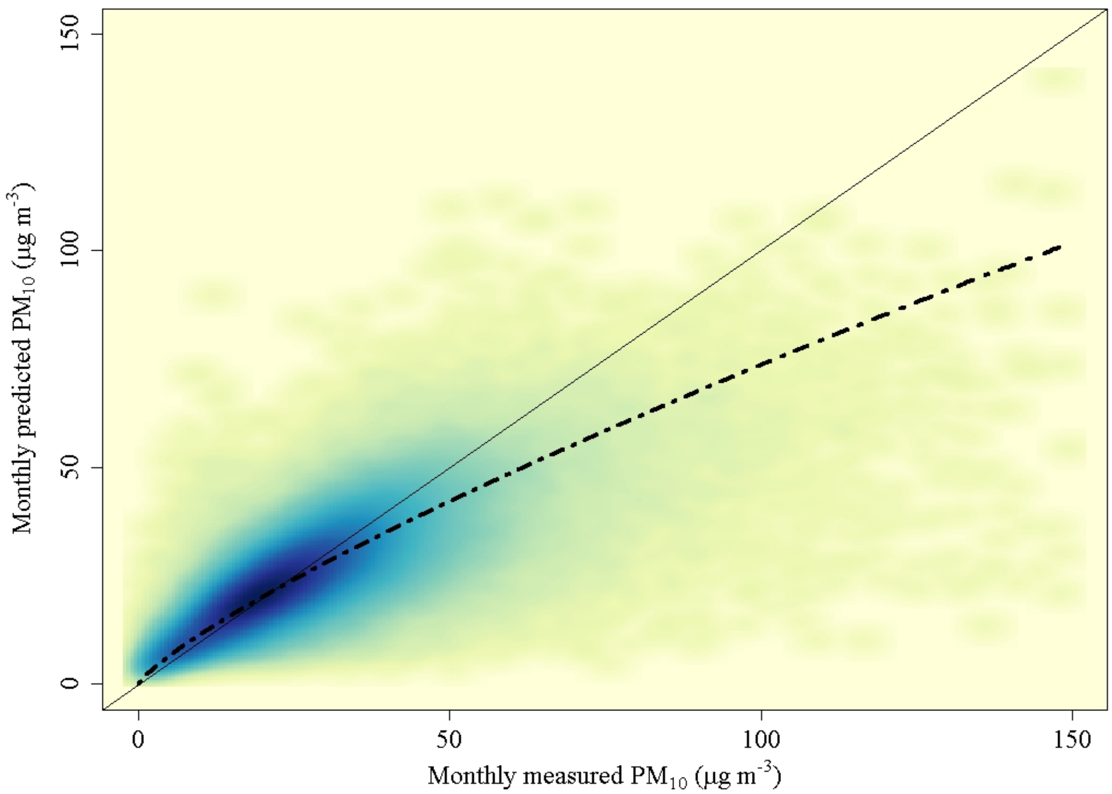

Monthly predicted PM10 (μg m-3)
Monthly measured PM10 (μg m-3)
0
50
100
150
0
50
100
150

D)

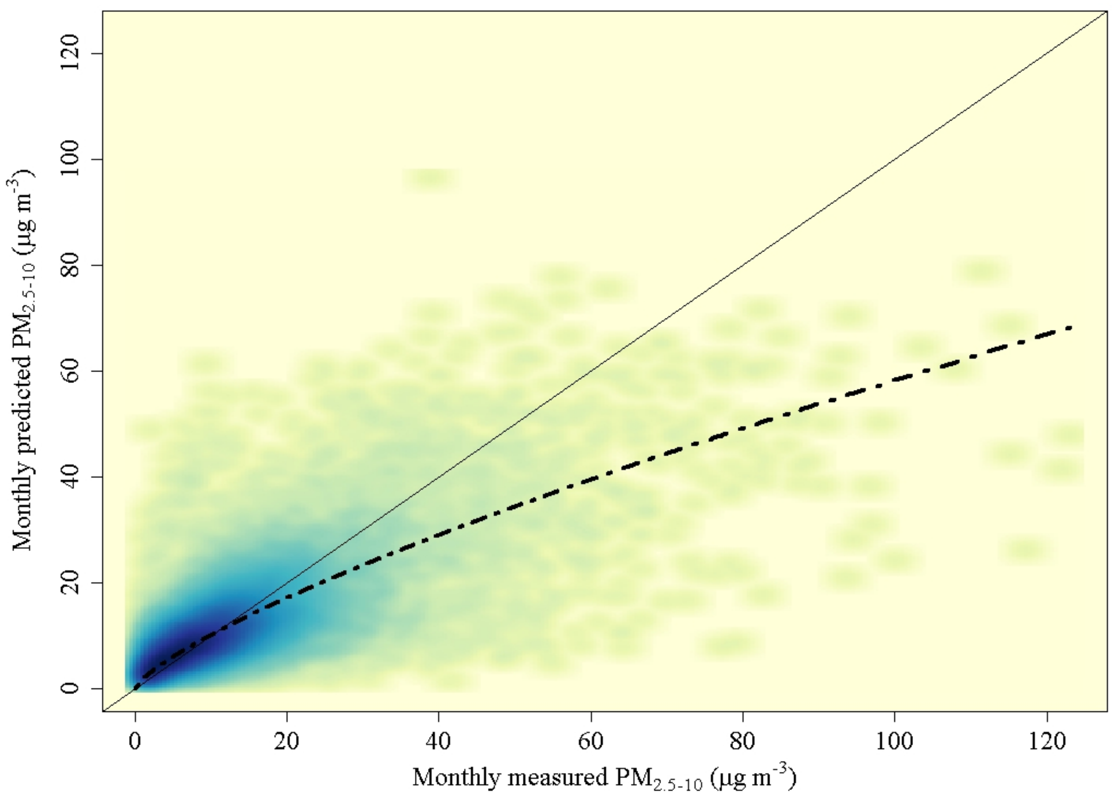

Monthly predicted PM2.5-10 (μg m-3)
0
20
40
60
80
100
120
0
20
40
60
80
100
120
Monthly measured PM2.5-10 (μg m-3)

E)

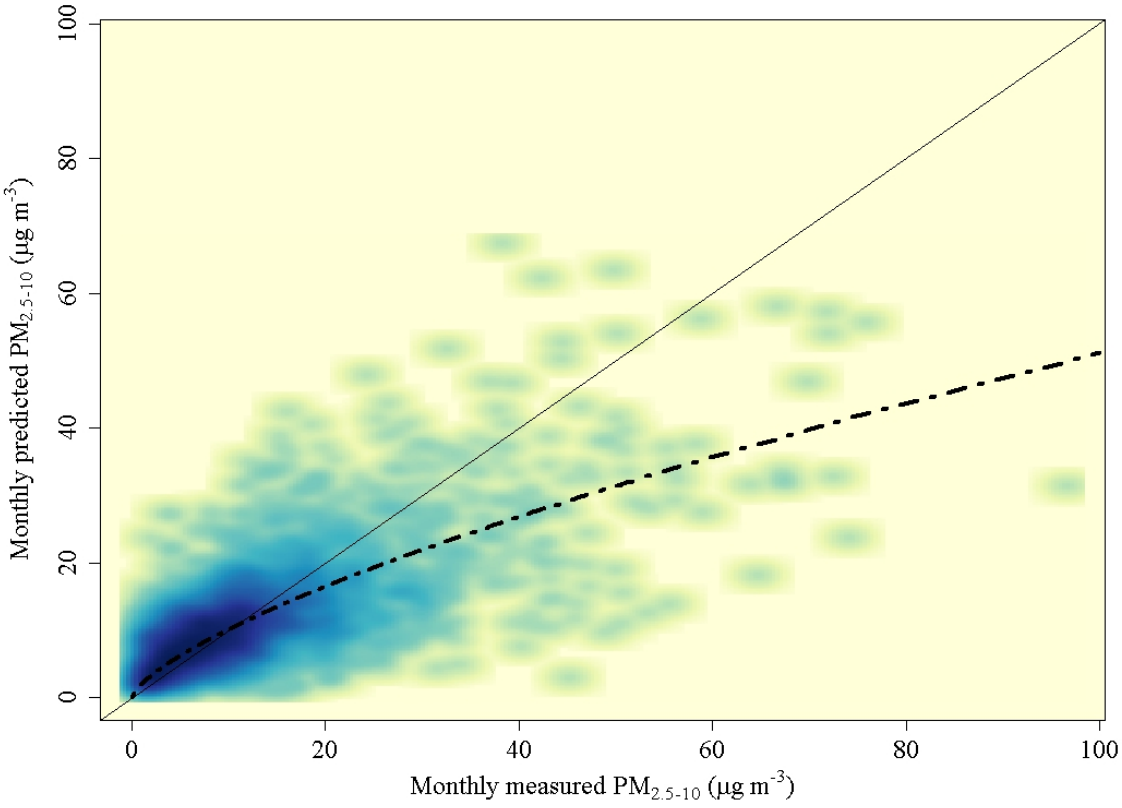

Monthly predicted PM2.5-10 (μg m-3)
0
20
40
60
80
100
Monthly measured PM2.5-10 (μg m-3)

Figure S3. Box plots of the temporal autocorrelation in model residuals (calculated by site, then summarized across sites) for the A) 1999-2007 $PM_{2.5}$, B) 1988-1998 $PM_{2.5}$, and C) 1988-2007 PM_{10} models.

A)

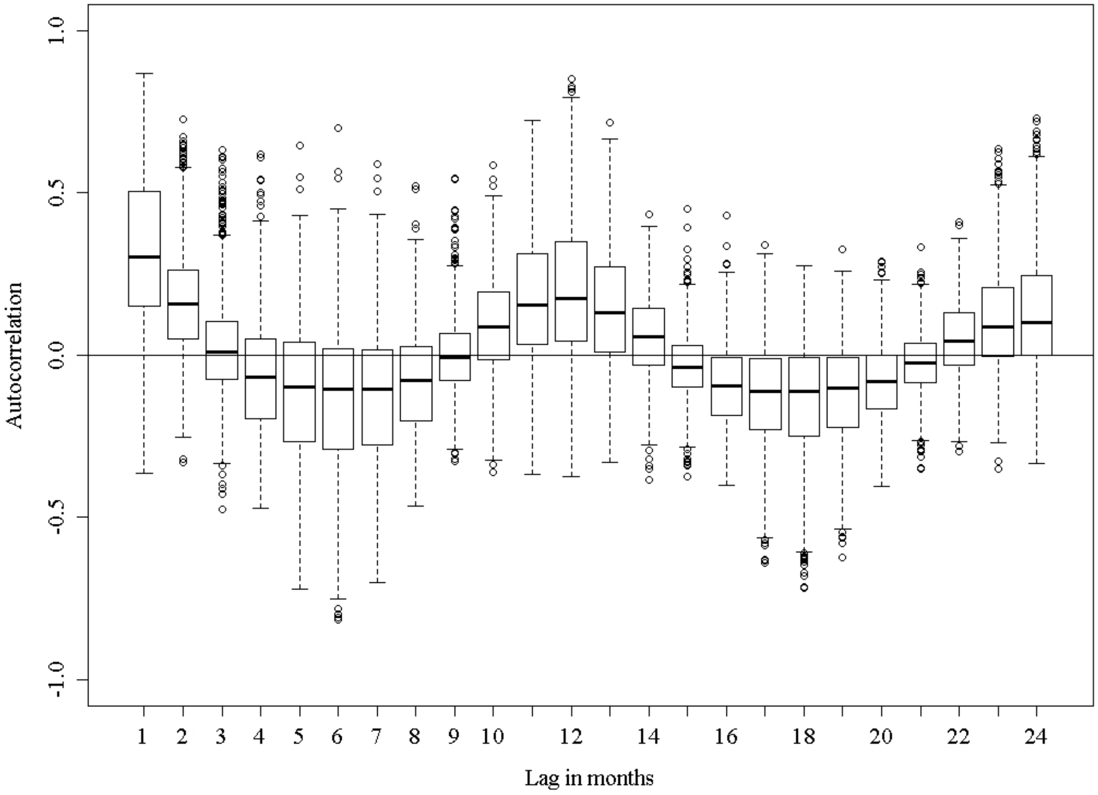

B)

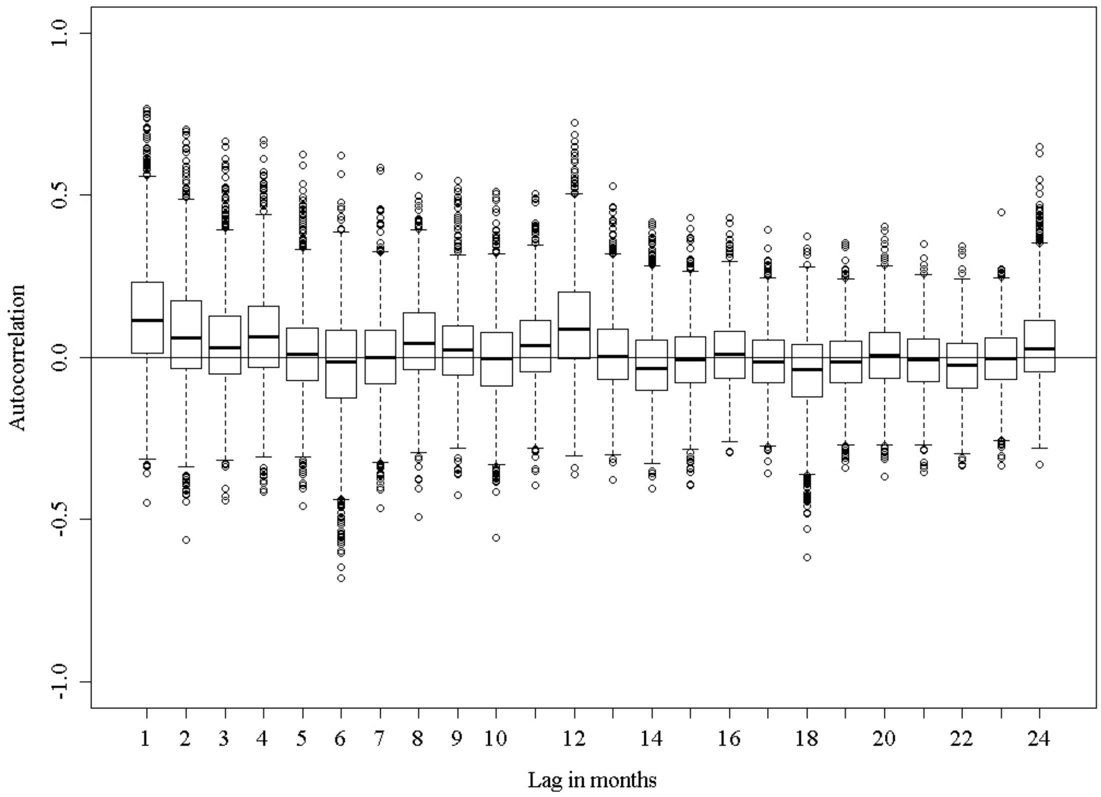

1.0
0.5
0.0
-0.5
-1.0
Autocorrelation
1
2
3
4
5
6
7
8
9
10
12
14
16
18
20
22
24
Lag in months

C)

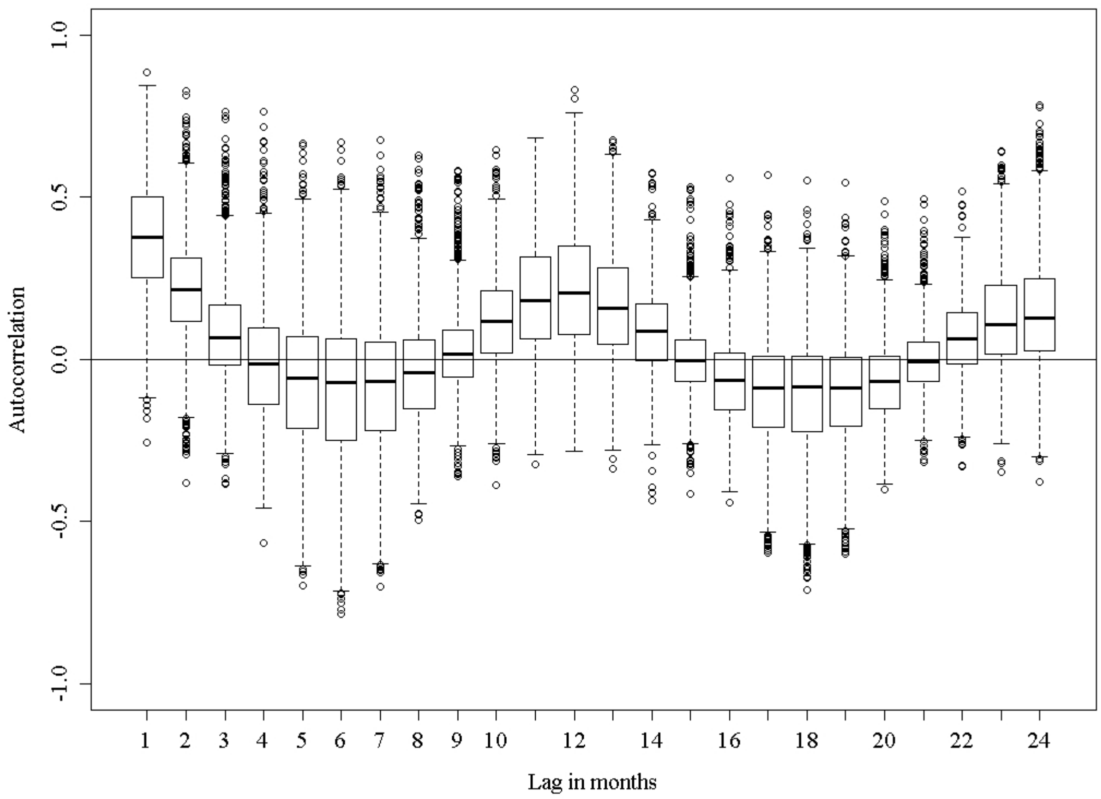

Autocorrelation
1.0
0.5
0.0
-0.5
-1.0
1
2
3
4
5
6
7
8
9
10
12
14
16
18
20
22
24
Lag in months

Figure S4. Means of monthly standard errors in predicted $PM_{2.5}$ concentrations (5th to 95th percentiles shown) for A) 1999-2007 and B) 1988-1998.

A)

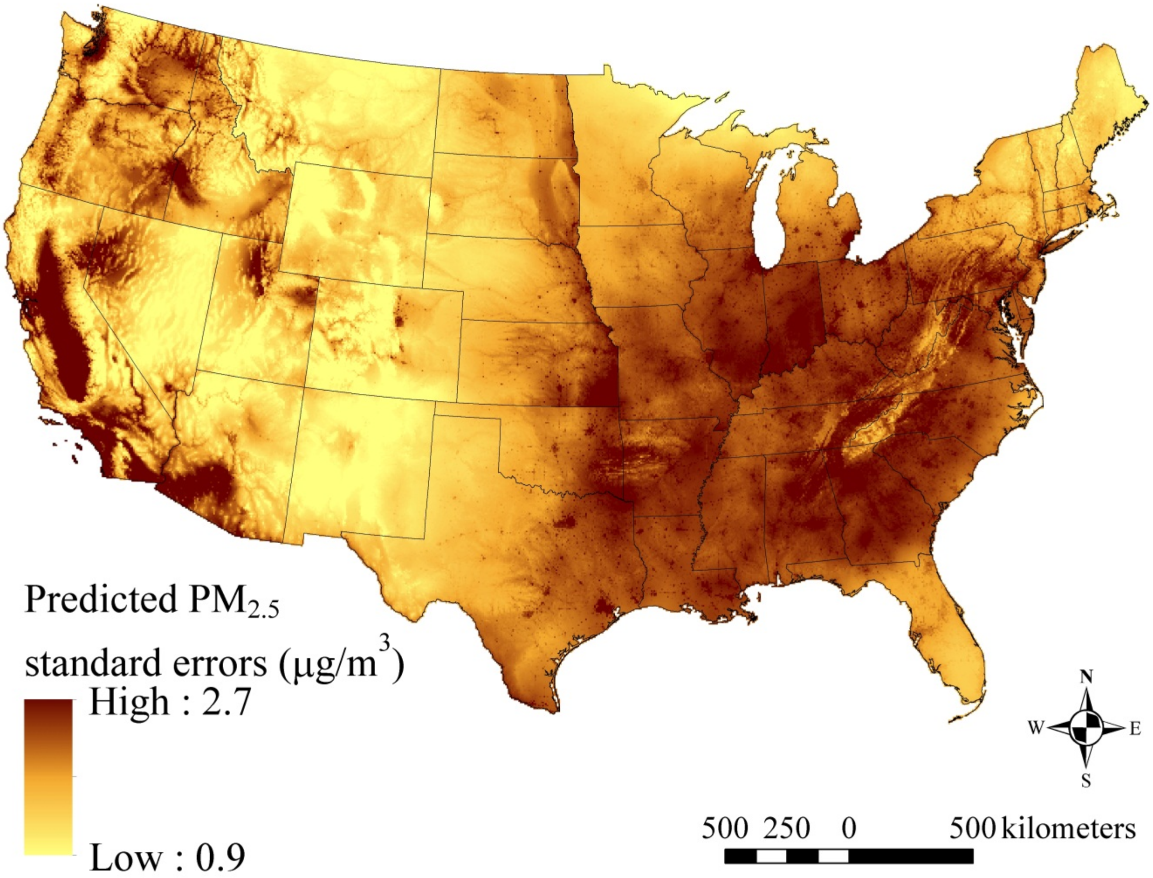

B)

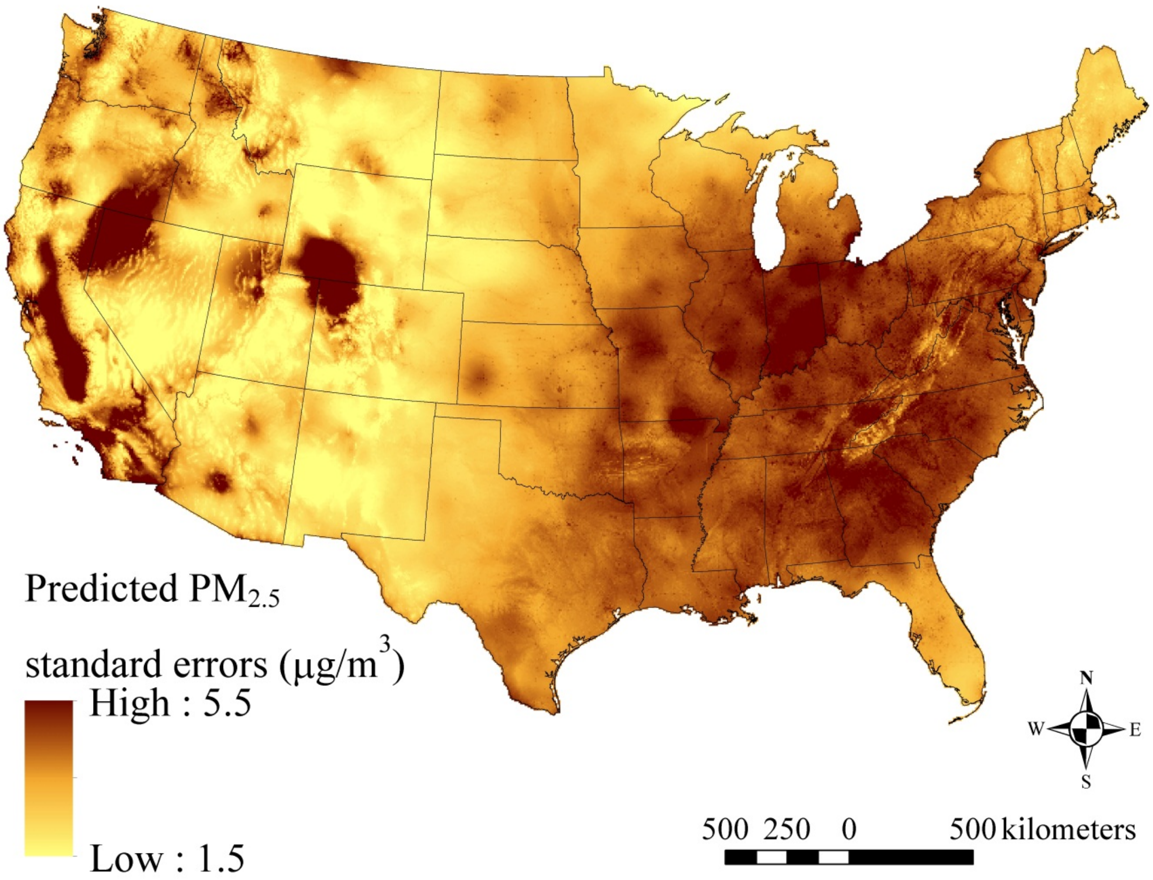
Predicted PM2.5
standard errors (μg/m3)
High : 5.5
Low : 1.5
N
W
E
S
500 250 0 500 kilometers

Figure S5. Means of monthly standard errors in predicted PM_{10} concentrations from 1988-2007 (5th to 95th percentiles shown).

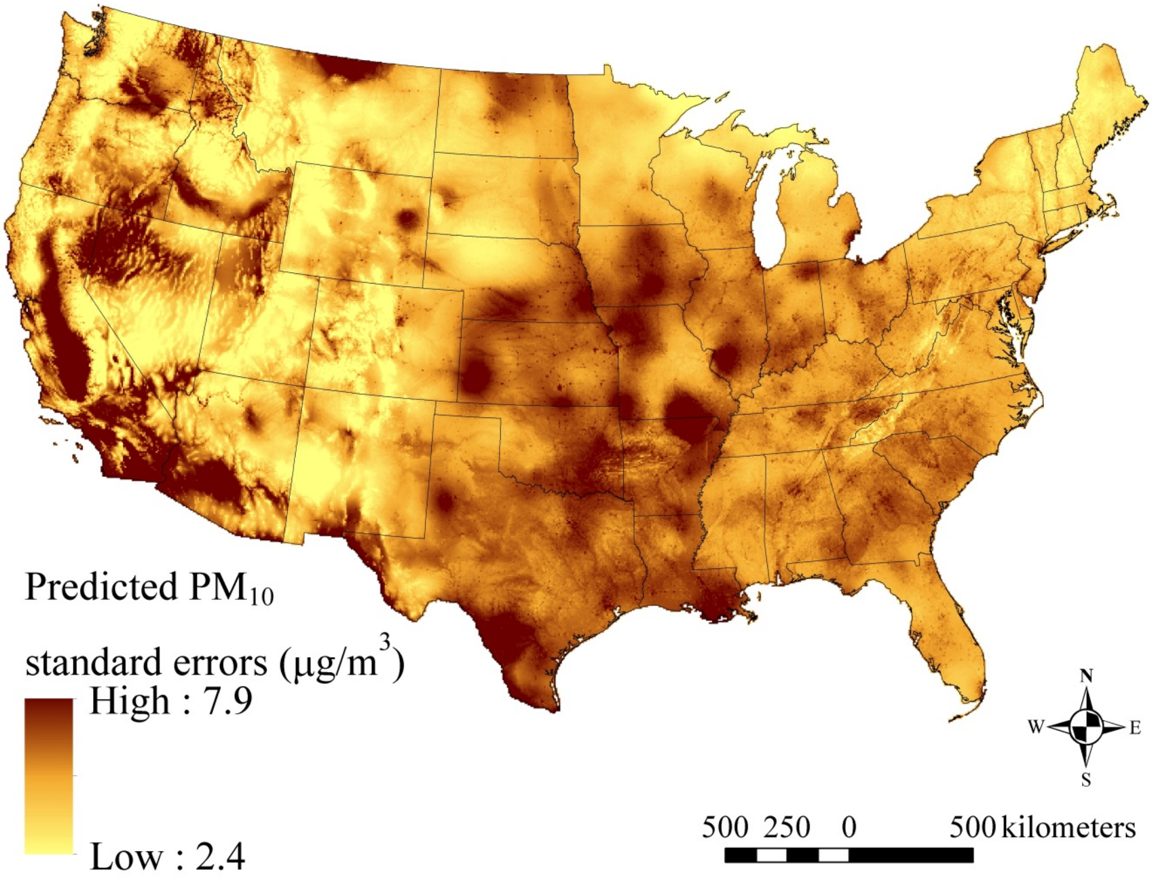

Figure S6. Means of monthly standard errors in predicted $PM_{2.5\text{-}10}$ concentrations (5th to 95th percentiles shown) for A) 1999-2007 and B) 1988-1998.

A)

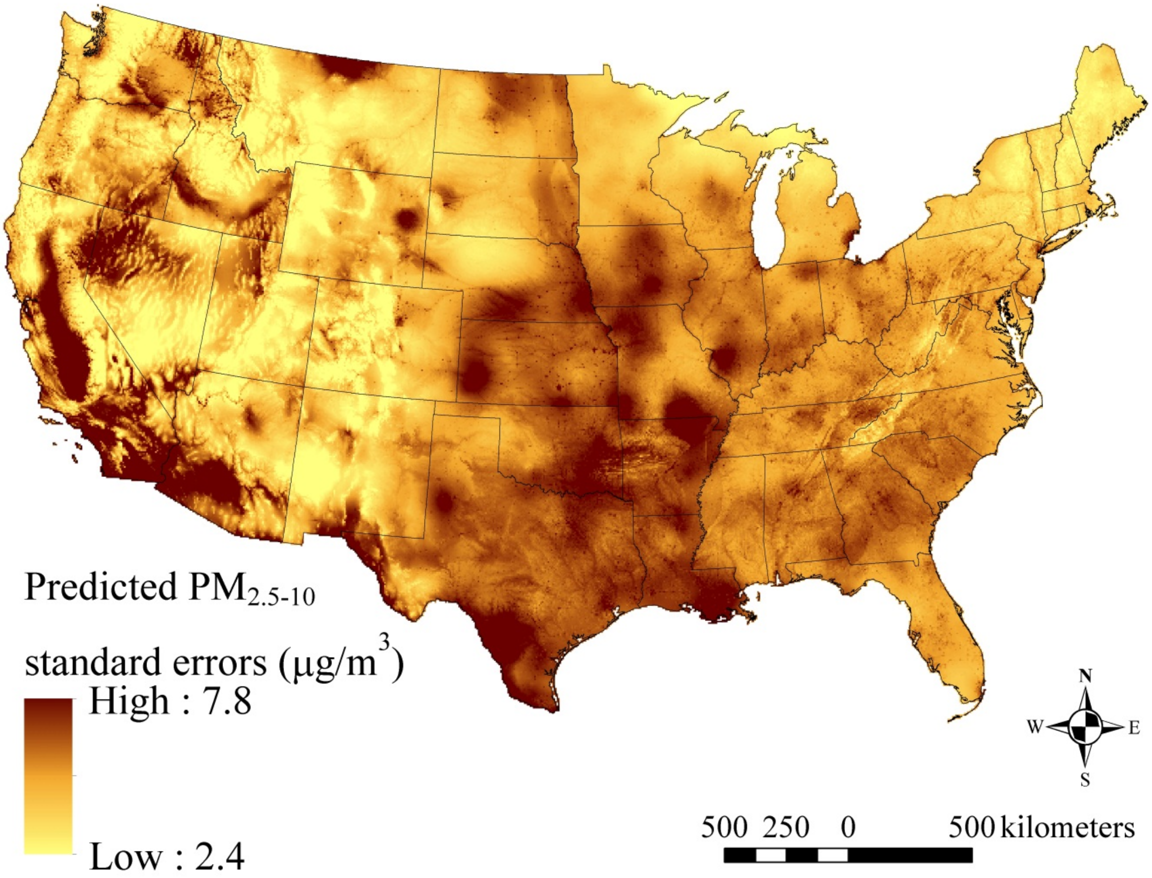

B)

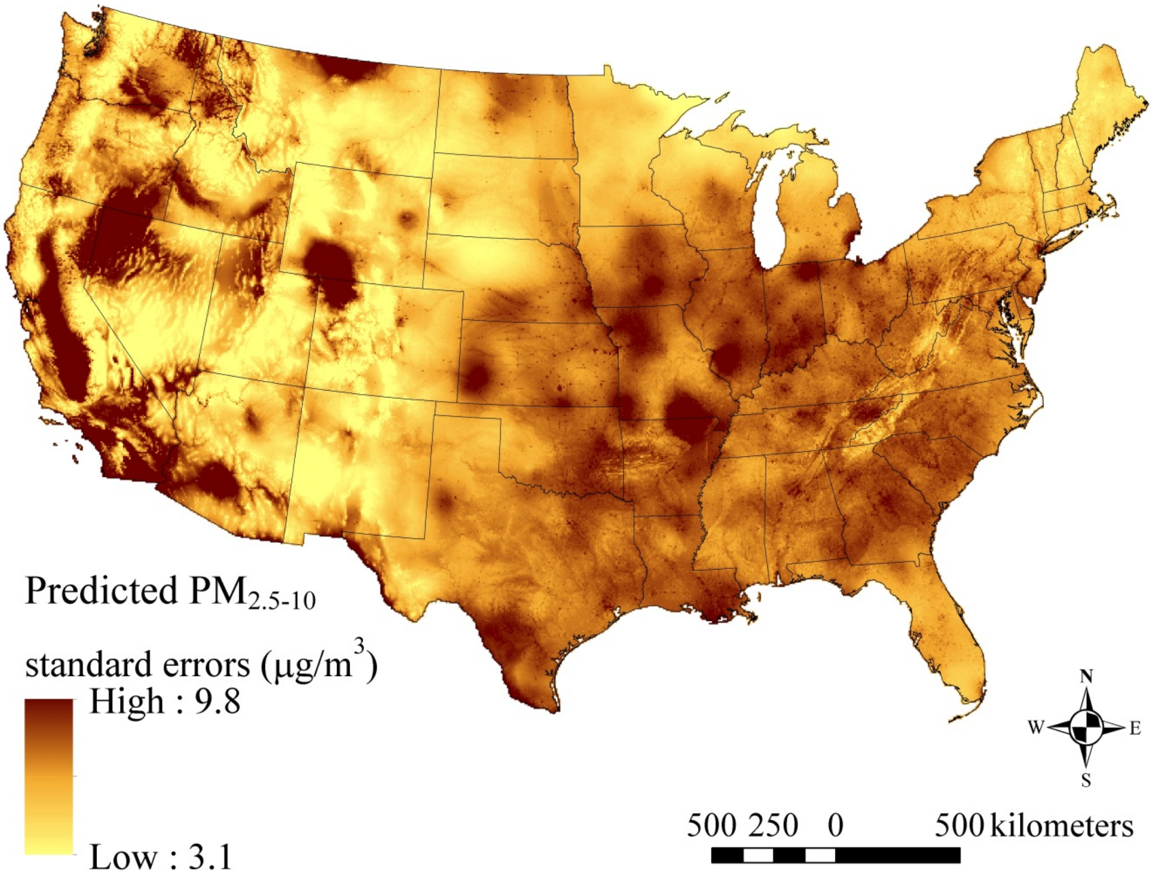
Predicted $PM_{2.5-10}$
standard errors ($\mu g/m^3$)
High : 9.8
Low : 3.1
N
W
E
S
500 250 0 500 kilometers
