# Supplementary material for: Spatio-temporal modeling of particulate air pollution in the conterminous United States using geographic and meteorological predictors
Source: Environ Health. 2014 Aug 5;13:63. doi: 10.1186/1476-069X-13-63 (PMC4137272; doi:10.1186/1476-069X-13-63)
Supplement: Additional file 2 — This file contains a 240-page atlas of monthly model predicted PM2.5 mass concentrations (in μg/m3) from January 1988 to December 2007 plotted on a 6 km grid over the conterminous U.S. Note: Model predictions for months prior to January 1999 are from the 1988–1998 PM2.5 model; thereafter they are from the 1999–2007 PM2.5 model. Also, note that the scale of the legend changes across months to highlight spatial contrasts within a given month. The file is provided in portable document format (pdf). [file 1476-069X-13-63-S2.pdf]

Monthly predicted $PM_{2.5}$ ($\mu g\ m^{-3}$)

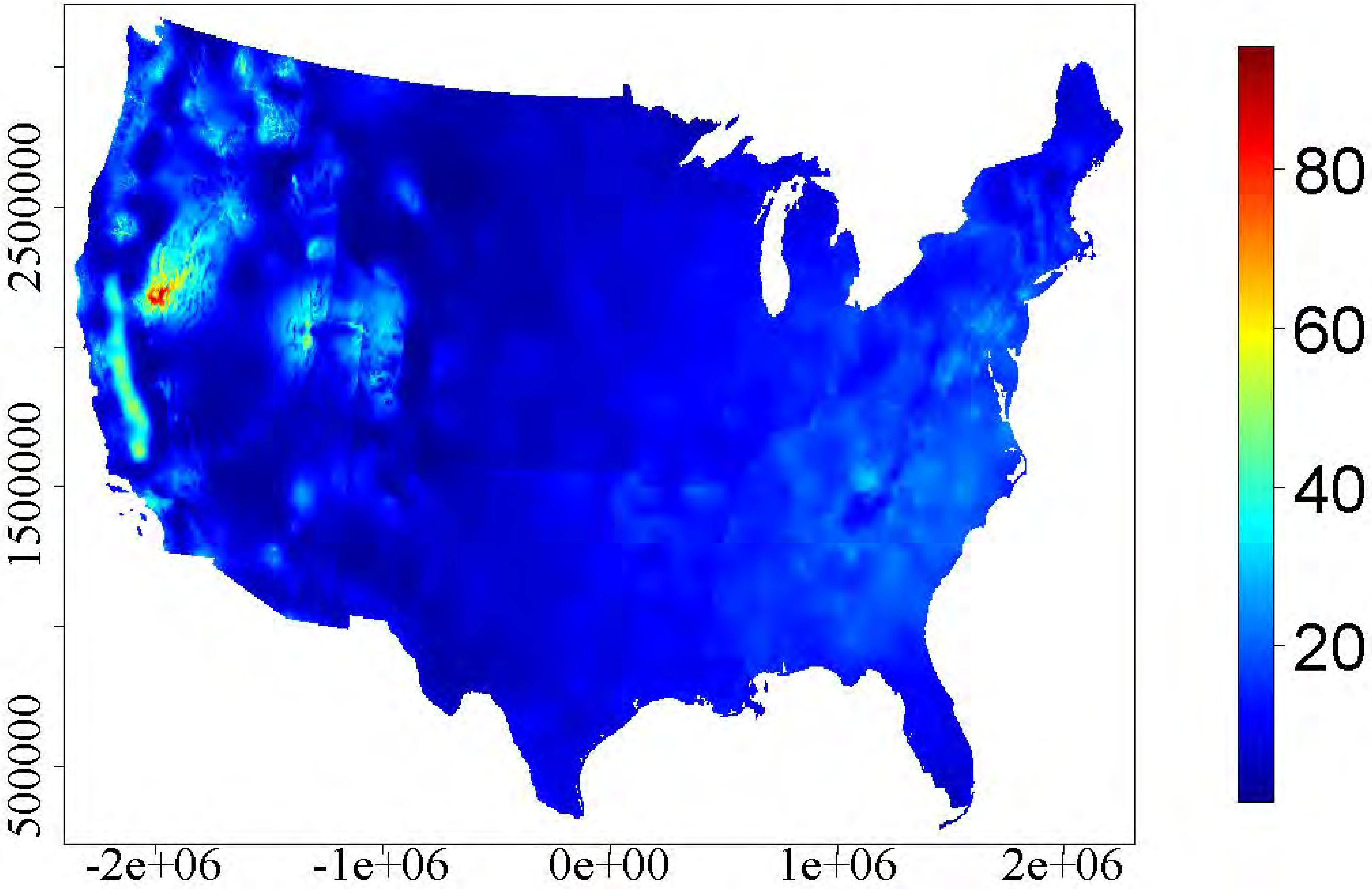

January 1988

Monthly predicted $PM_{2.5}$ ($\mu g\ m^{-3}$)

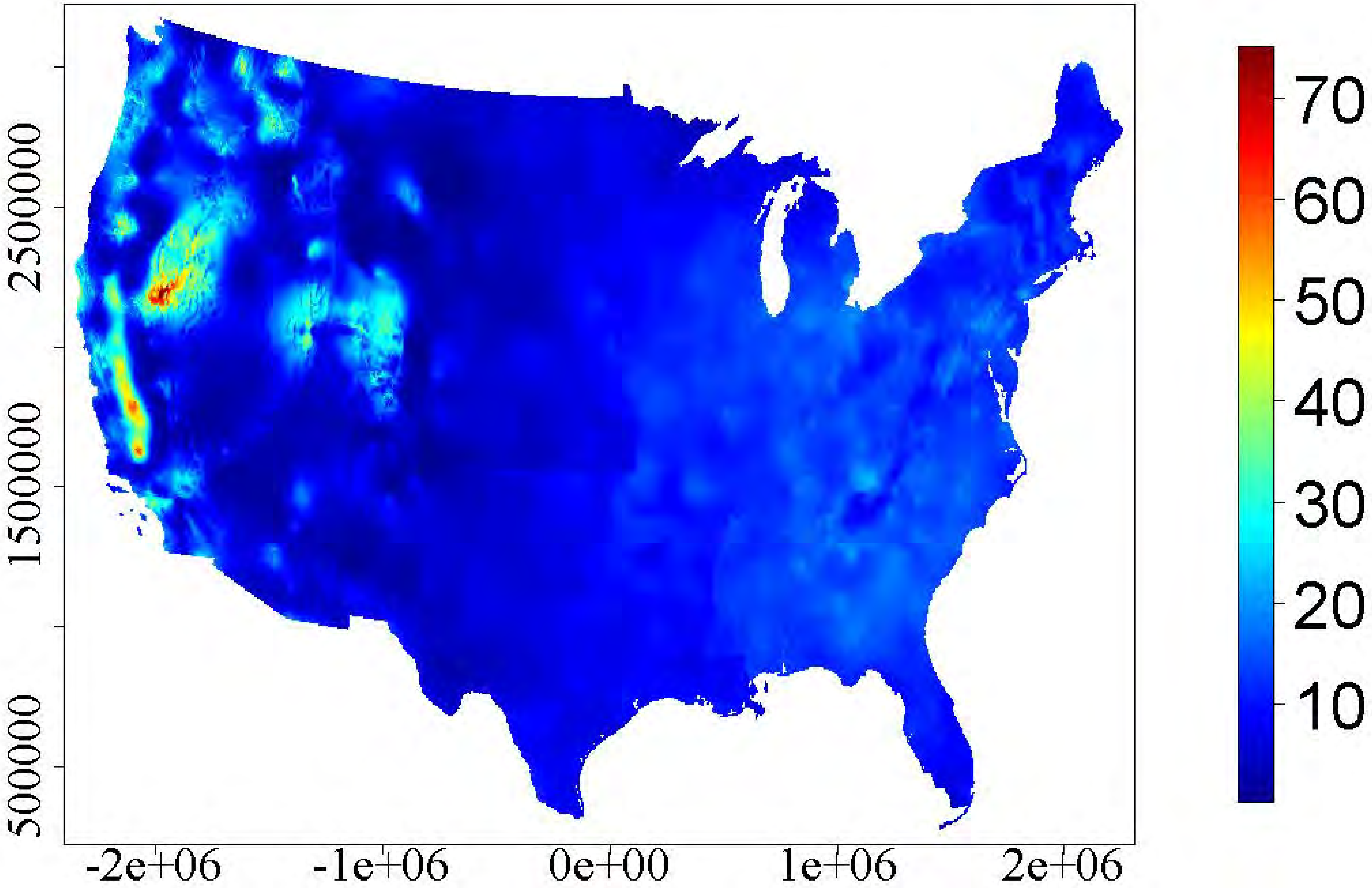

February 1988

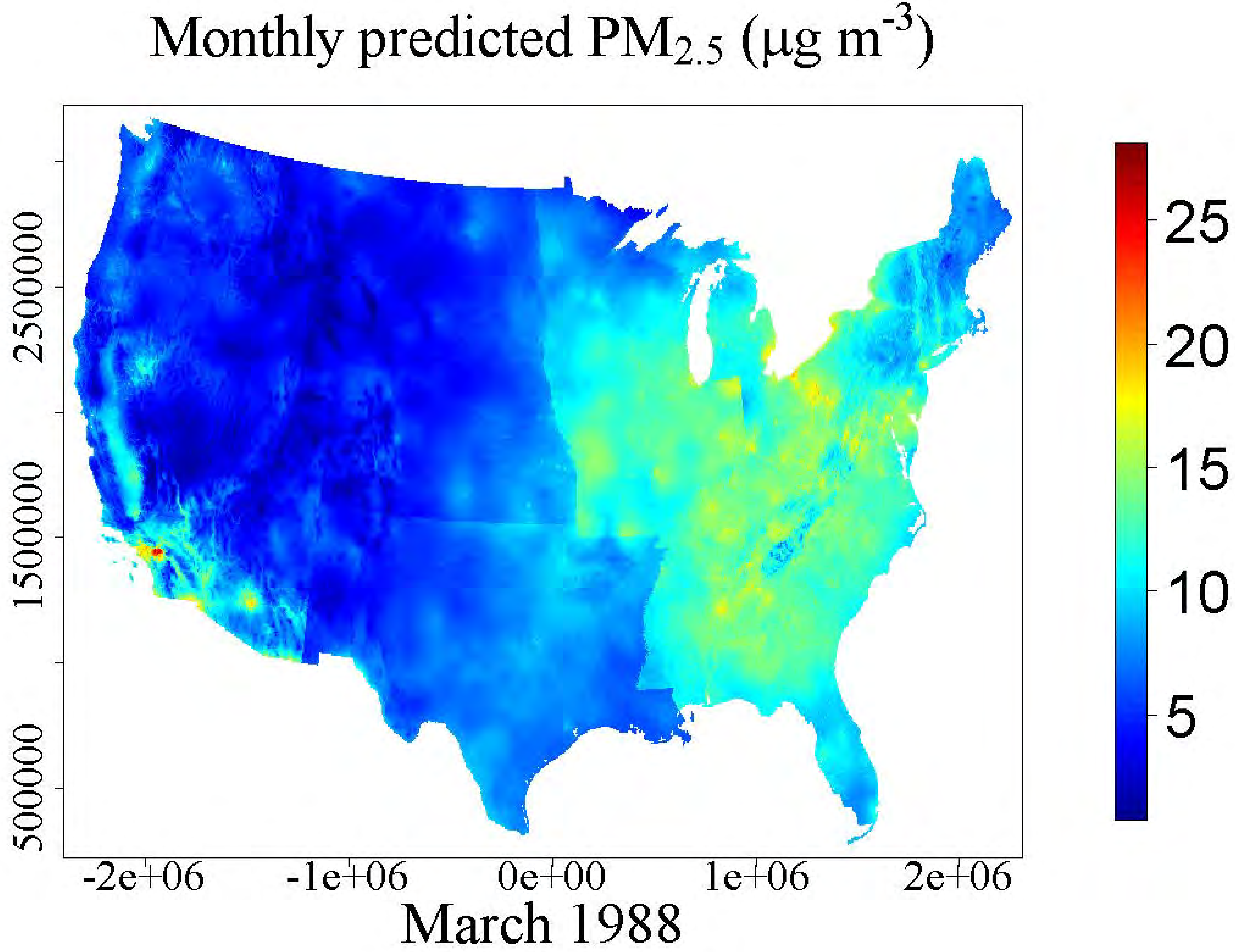

Monthly predicted PM2.5 (μg m-3)
25000000
15000000
5000000
-2e+06
-1e+06
0e+00
1e+06
2e+06
25
20
15
10
5
March 1988

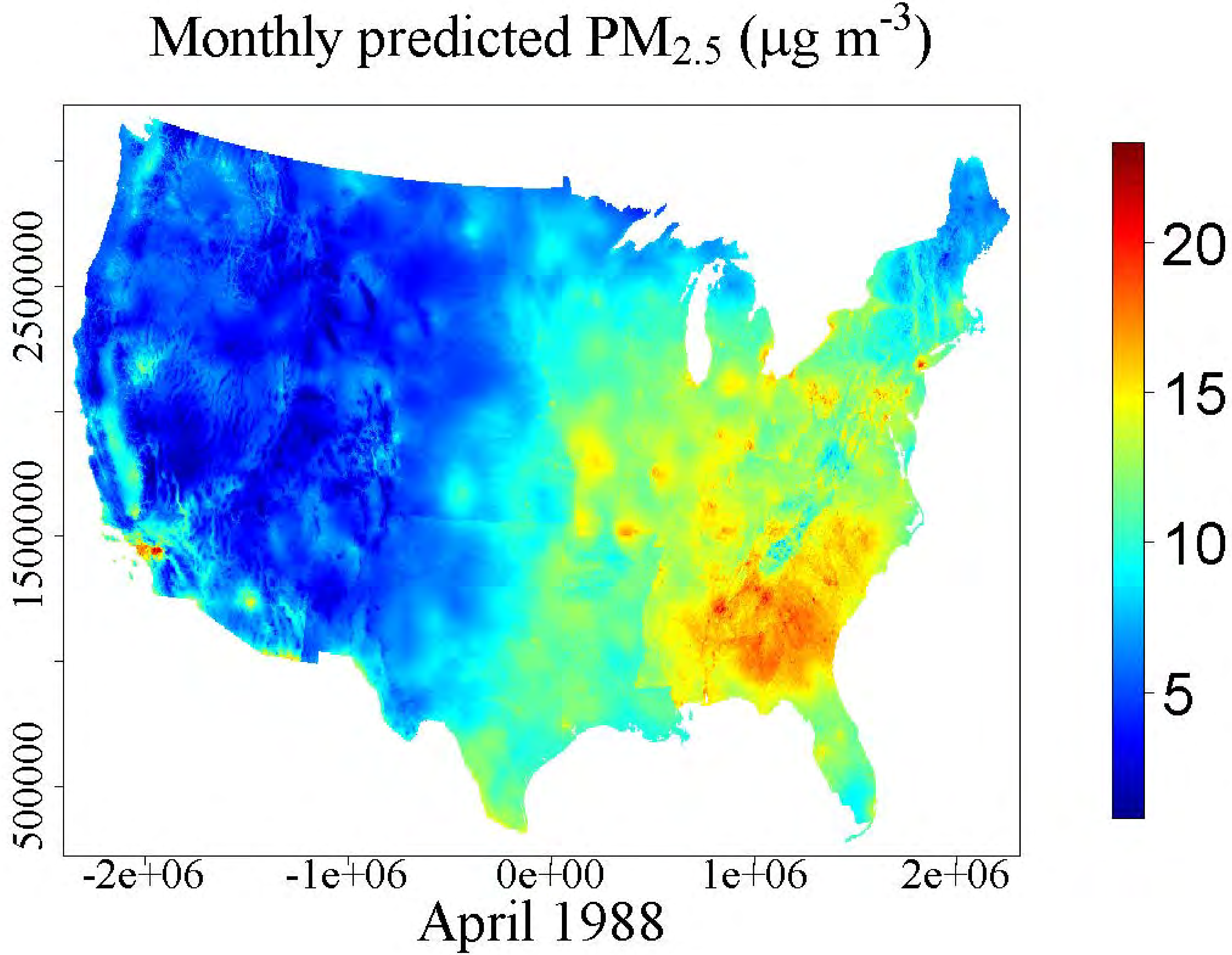
Monthly predicted PM2.5 (μg m-3)
25000000
15000000
5000000
-2e+06
-1e+06
0e+00
1e+06
2e+06
20
15
10
5
April 1988

Monthly predicted $PM_{2.5}$ ($\mu g\ m^{-3}$)

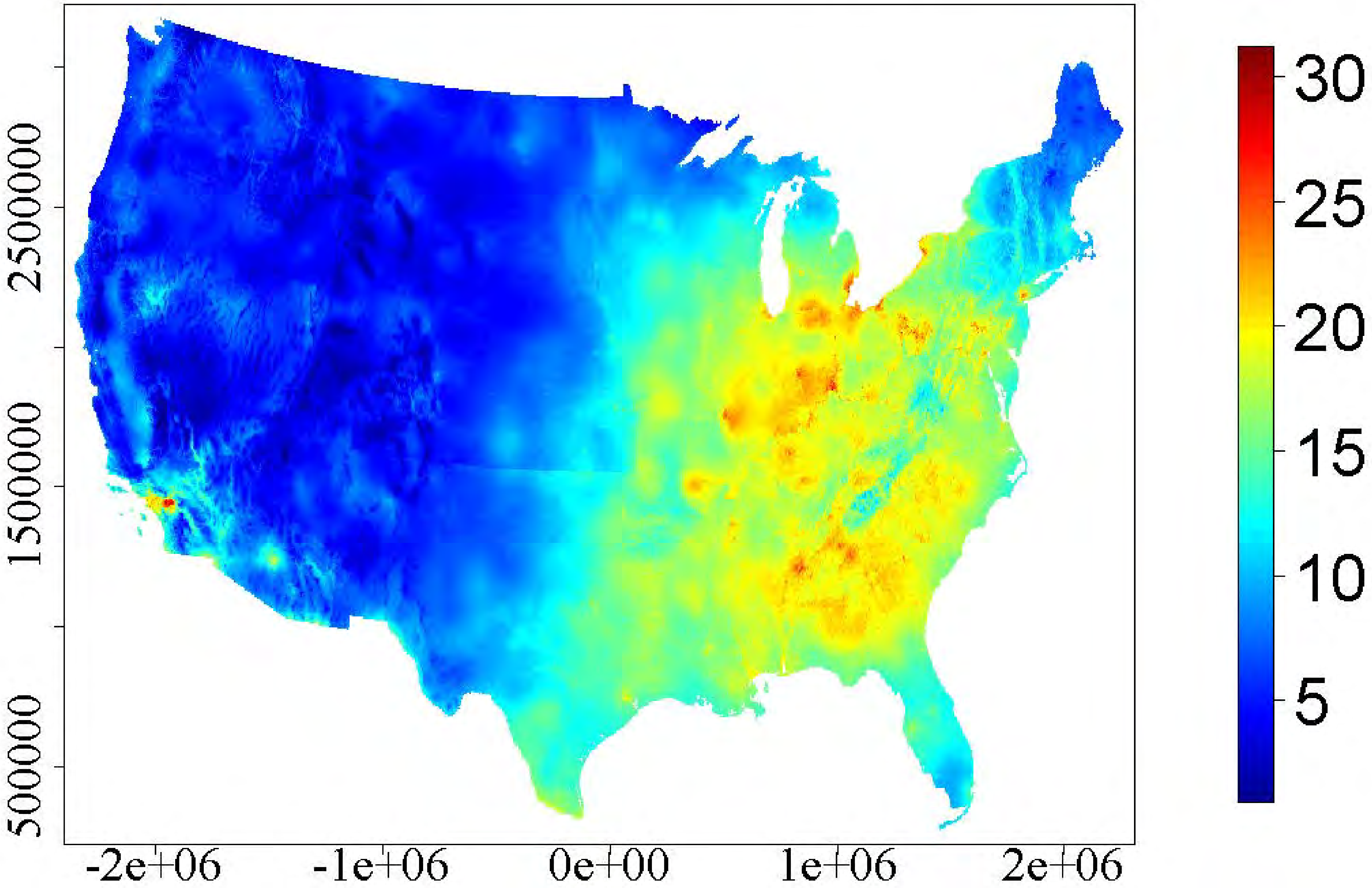

May 1988

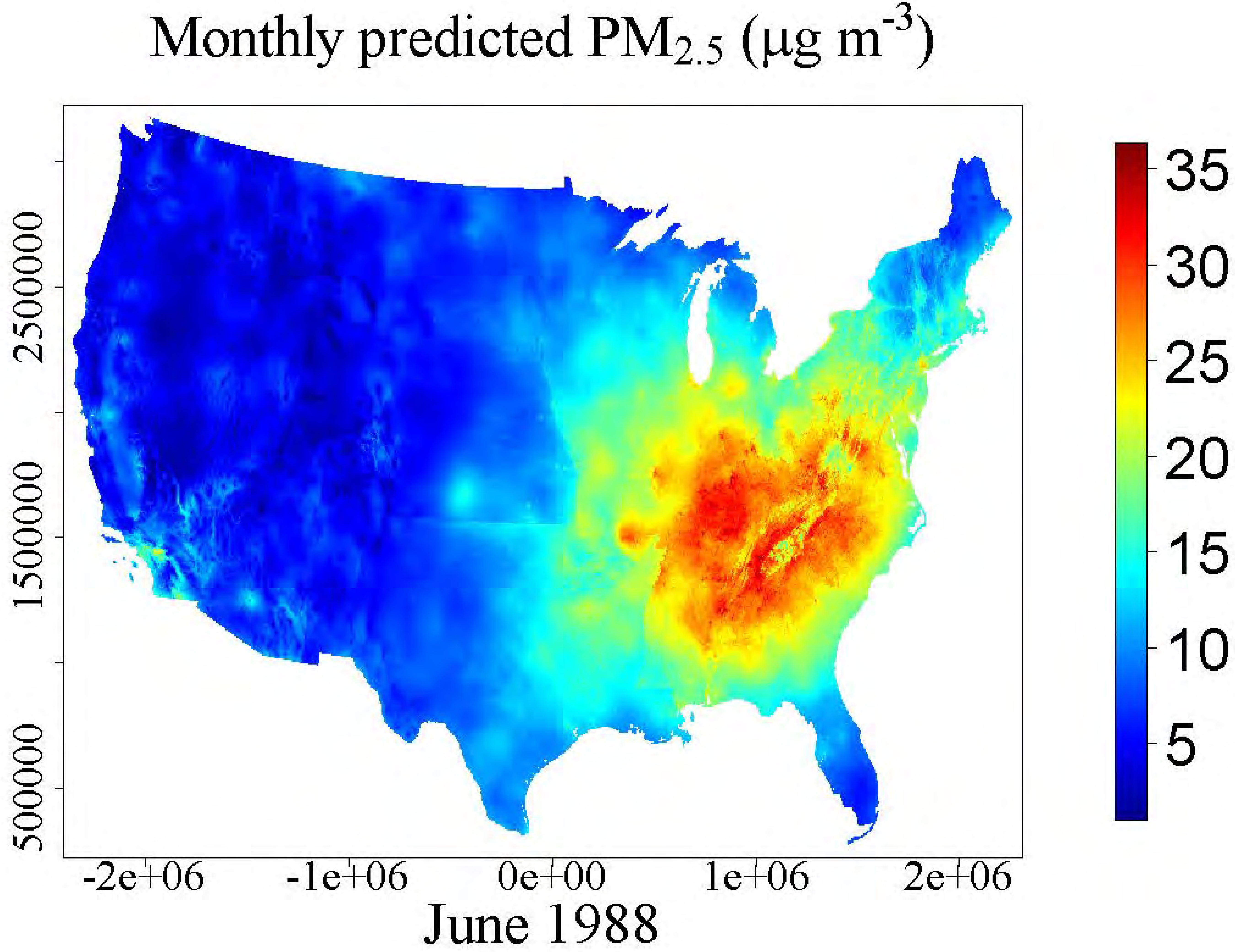
Monthly predicted $PM_{2.5}$ ($\mu g\ m^{-3}$)
25000000
15000000
5000000
-2e+06
-1e+06
0e+00
1e+06
2e+06
35
30
25
20
15
10
5
June 1988

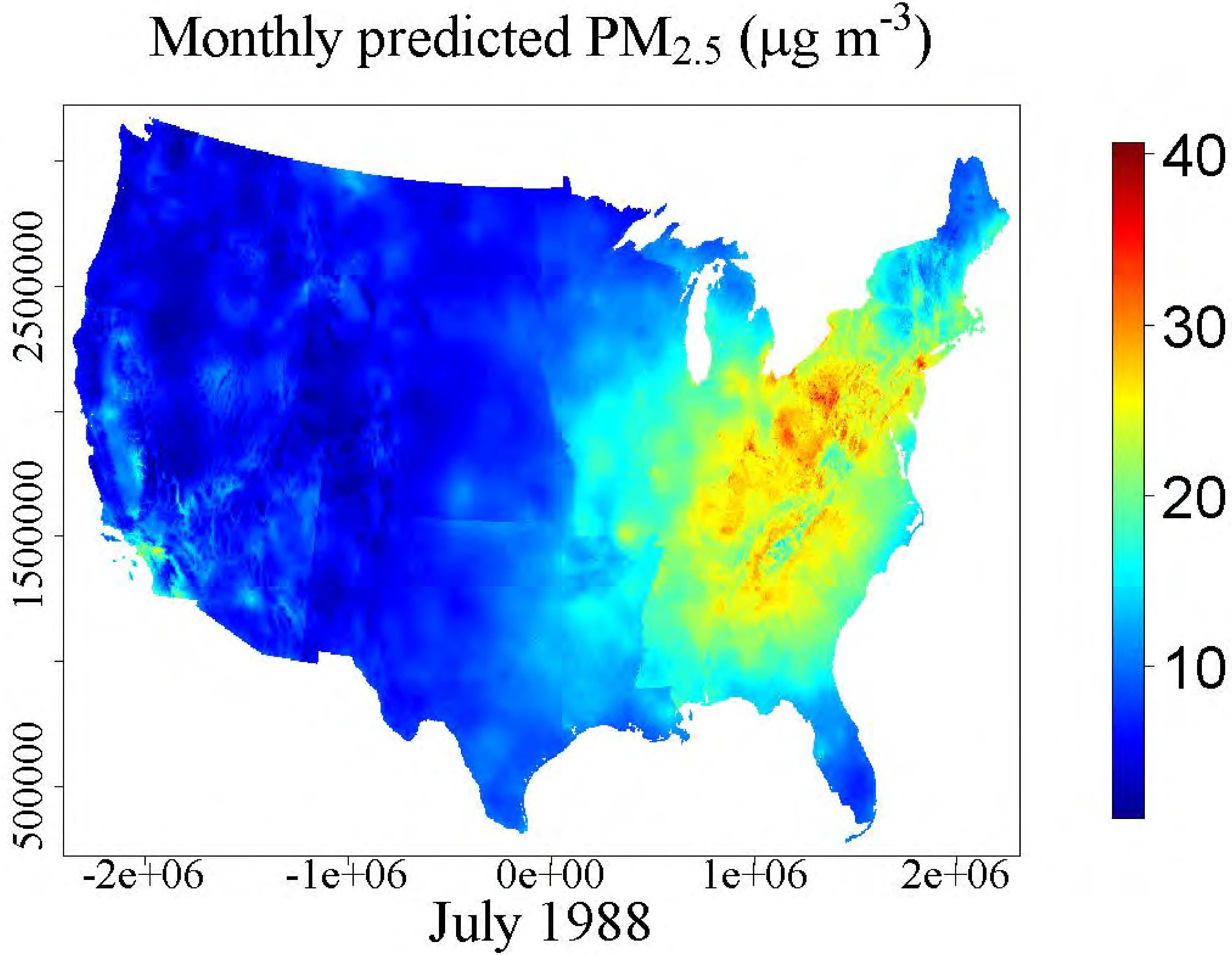
Monthly predicted $PM_{2.5}$ ($\mu g\ m^{-3}$)
25000000
15000000
5000000
-2e+06
-1e+06
0e+00
1e+06
2e+06
July 1988
40
30
20
10

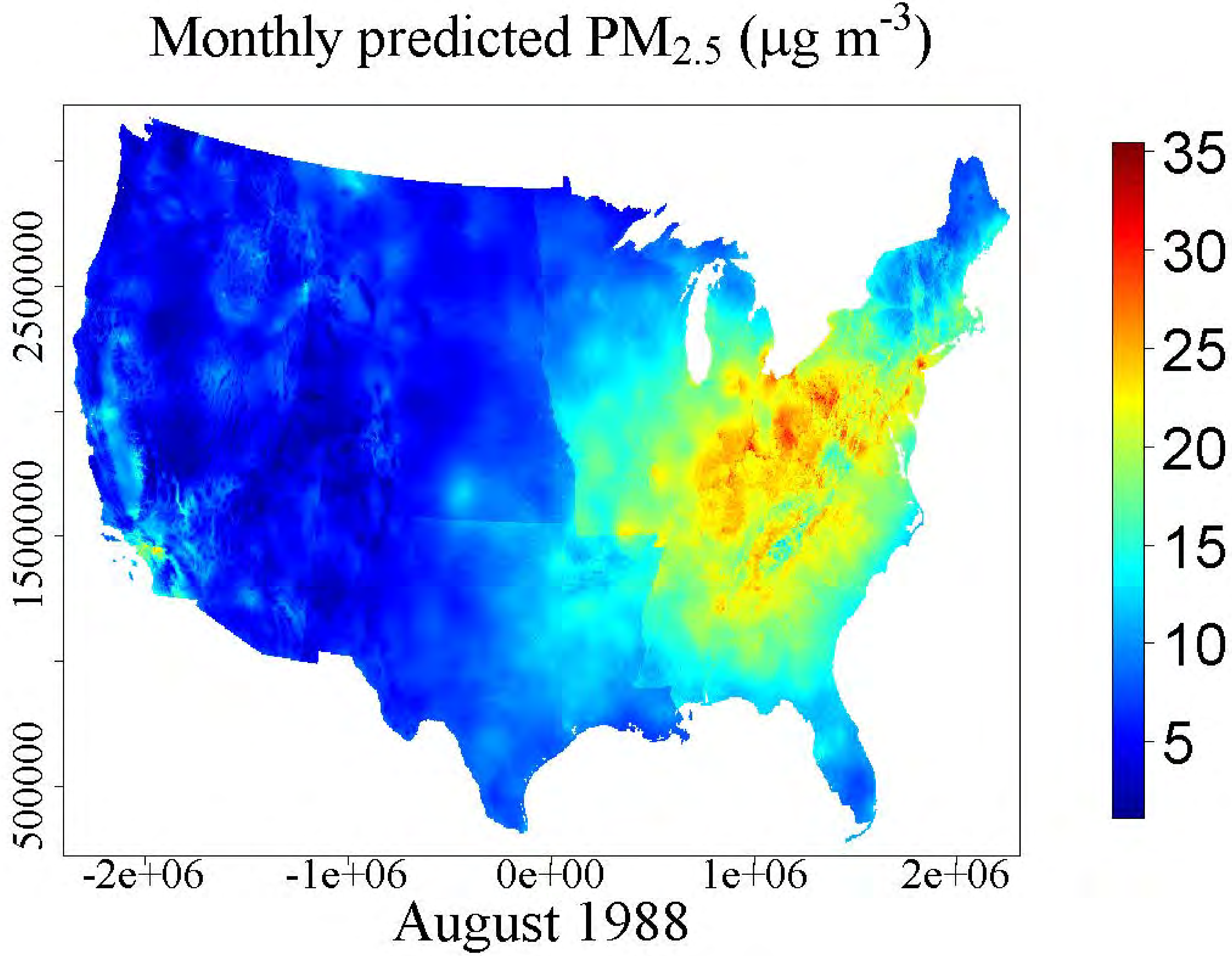

Monthly predicted PM2.5 (μg m-3)
25000000
15000000
5000000
-2e+06
-1e+06
0e+00
1e+06
2e+06
35
30
25
20
15
10
5
August 1988

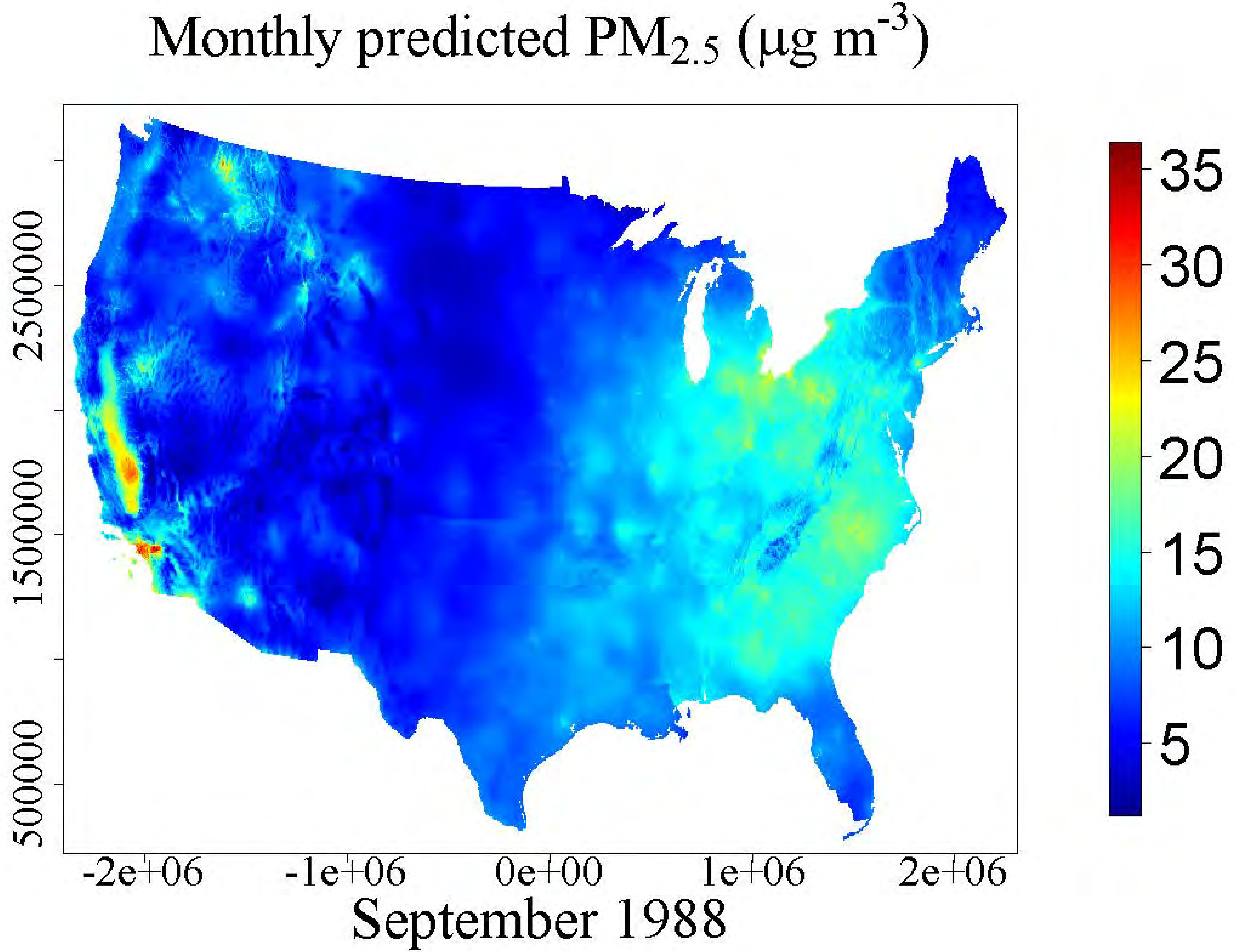
Monthly predicted PM2.5 (μg m-3)
25000000
15000000
5000000
-2e+06
-1e+06
0e+00
1e+06
2e+06
September 1988
35
30
25
20
15
10
5

Monthly predicted $PM_{2.5}$ ($\mu g\ m^{-3}$)

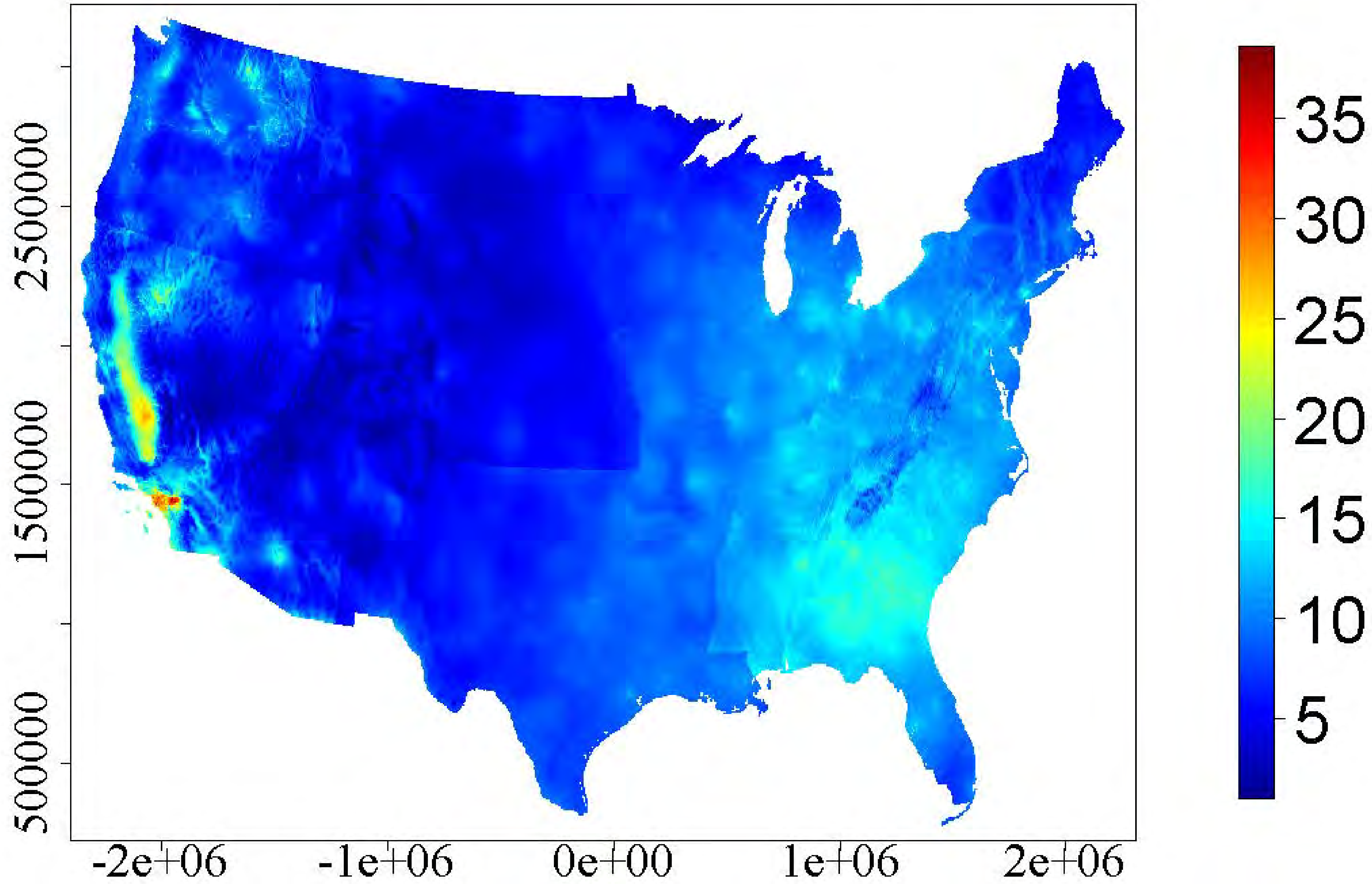

October 1988

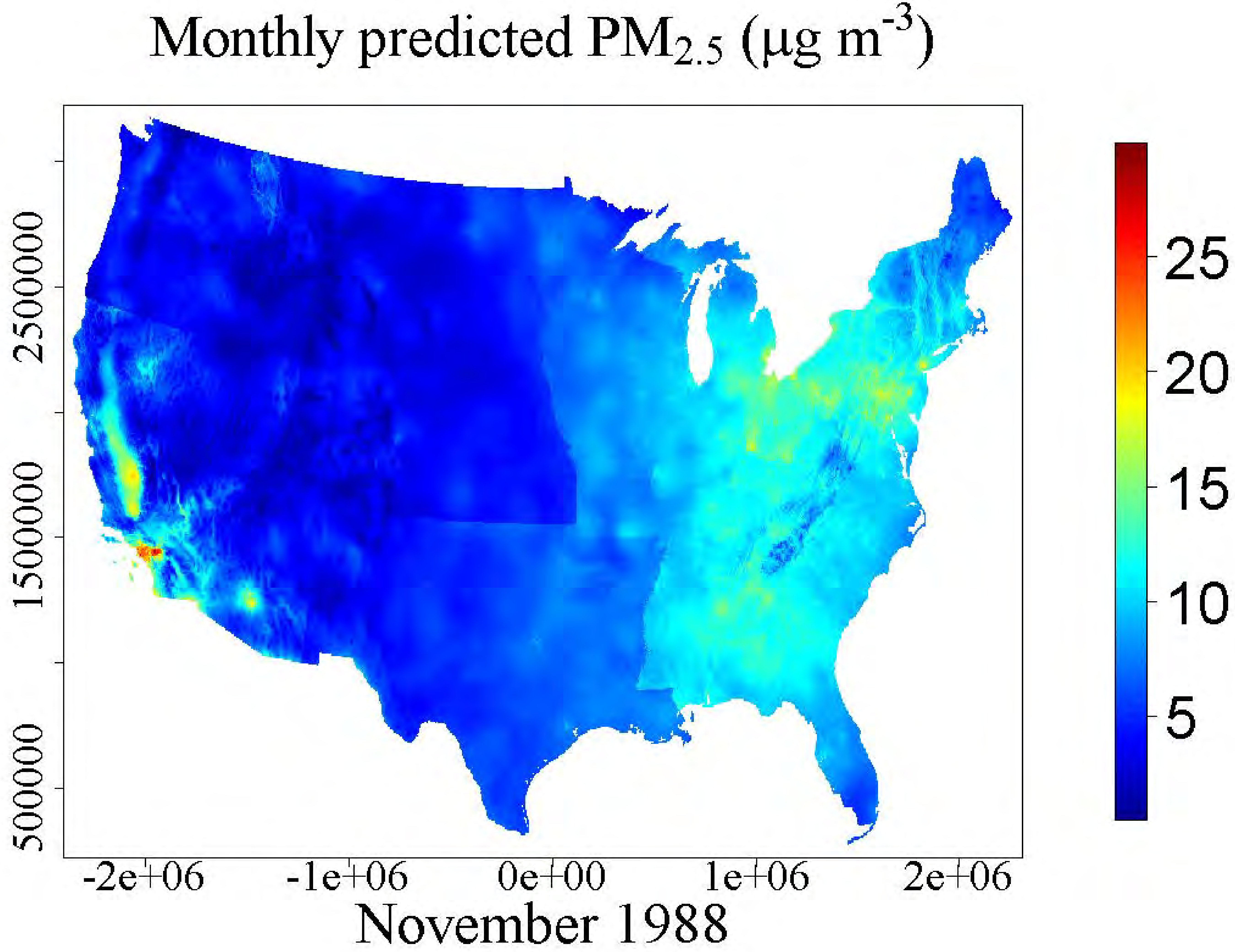
Monthly predicted PM2.5 (μg m-3)
25000000
15000000
5000000
-2e+06
-1e+06
0e+00
1e+06
2e+06
November 1988
25
20
15
10
5

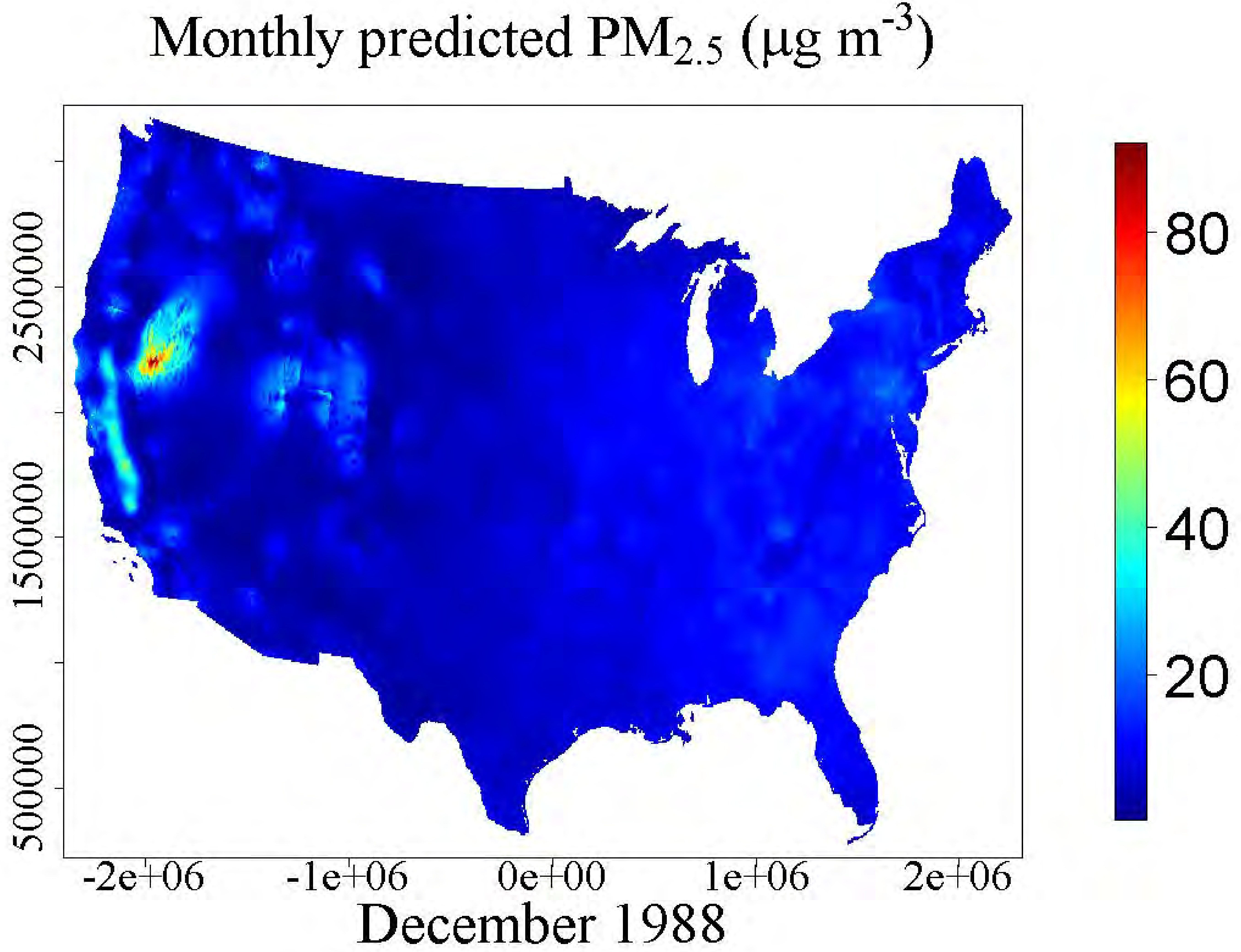
Monthly predicted PM2.5 (μg m-3)
25000000
15000000
5000000
-2e+06
-1e+06
0e+00
1e+06
2e+06
80
60
40
20
December 1988

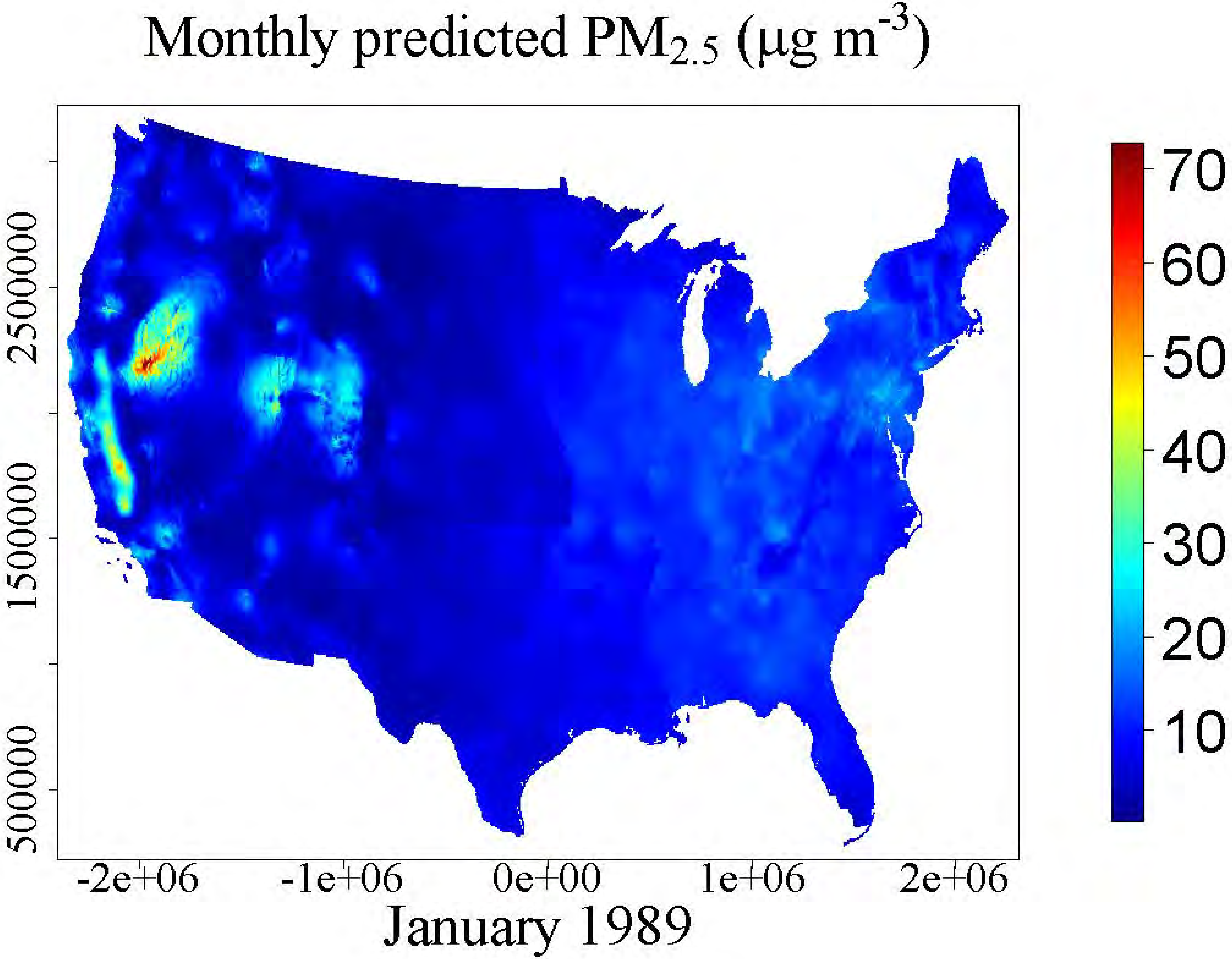

Monthly predicted PM2.5 (μg m-3)
25000000
15000000
5000000
-2e+06
-1e+06
0e+00
1e+06
2e+06
January 1989
70
60
50
40
30
20
10

Monthly predicted $PM_{2.5}$ ($\mu g\ m^{-3}$)

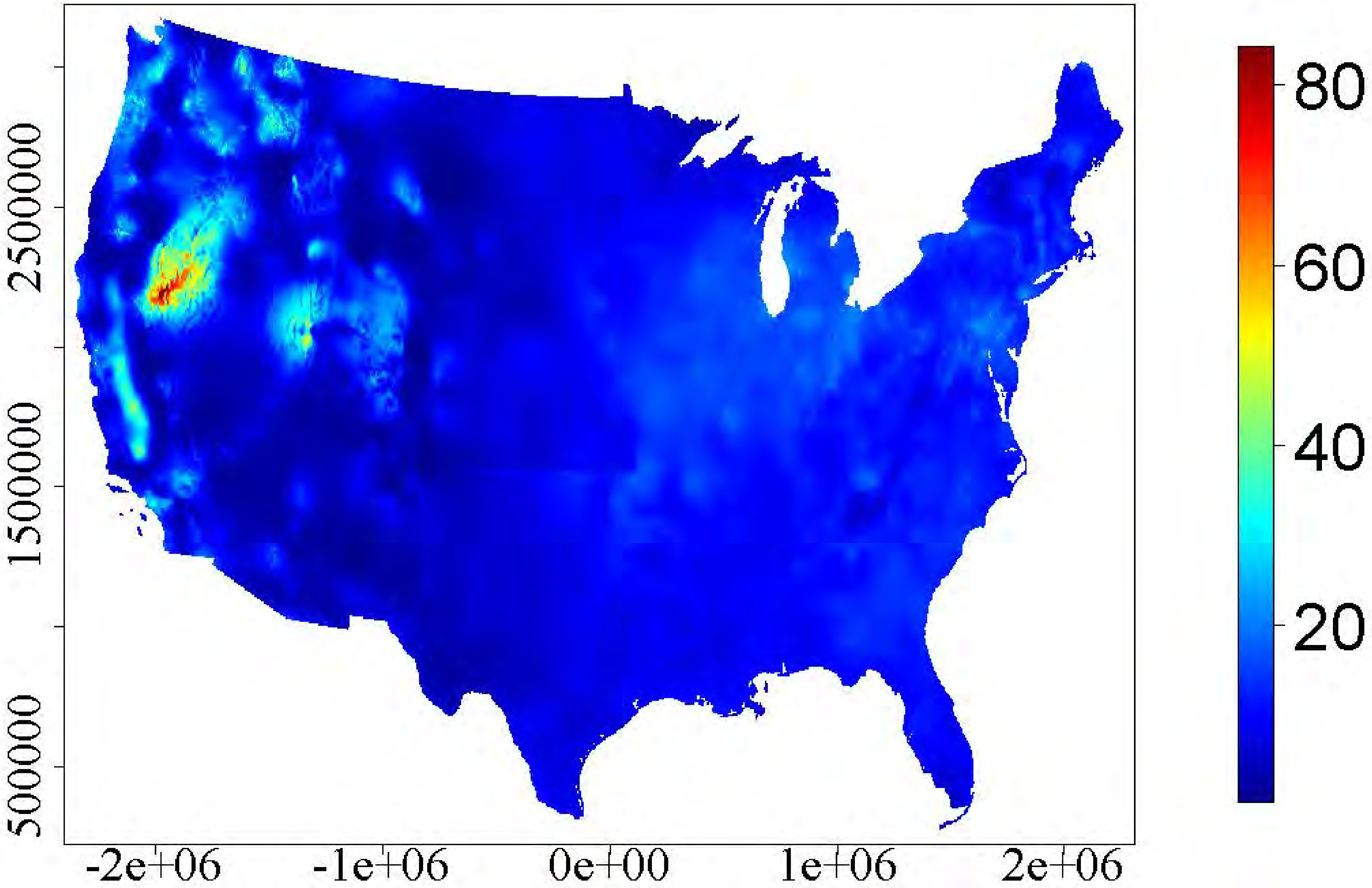

February 1989

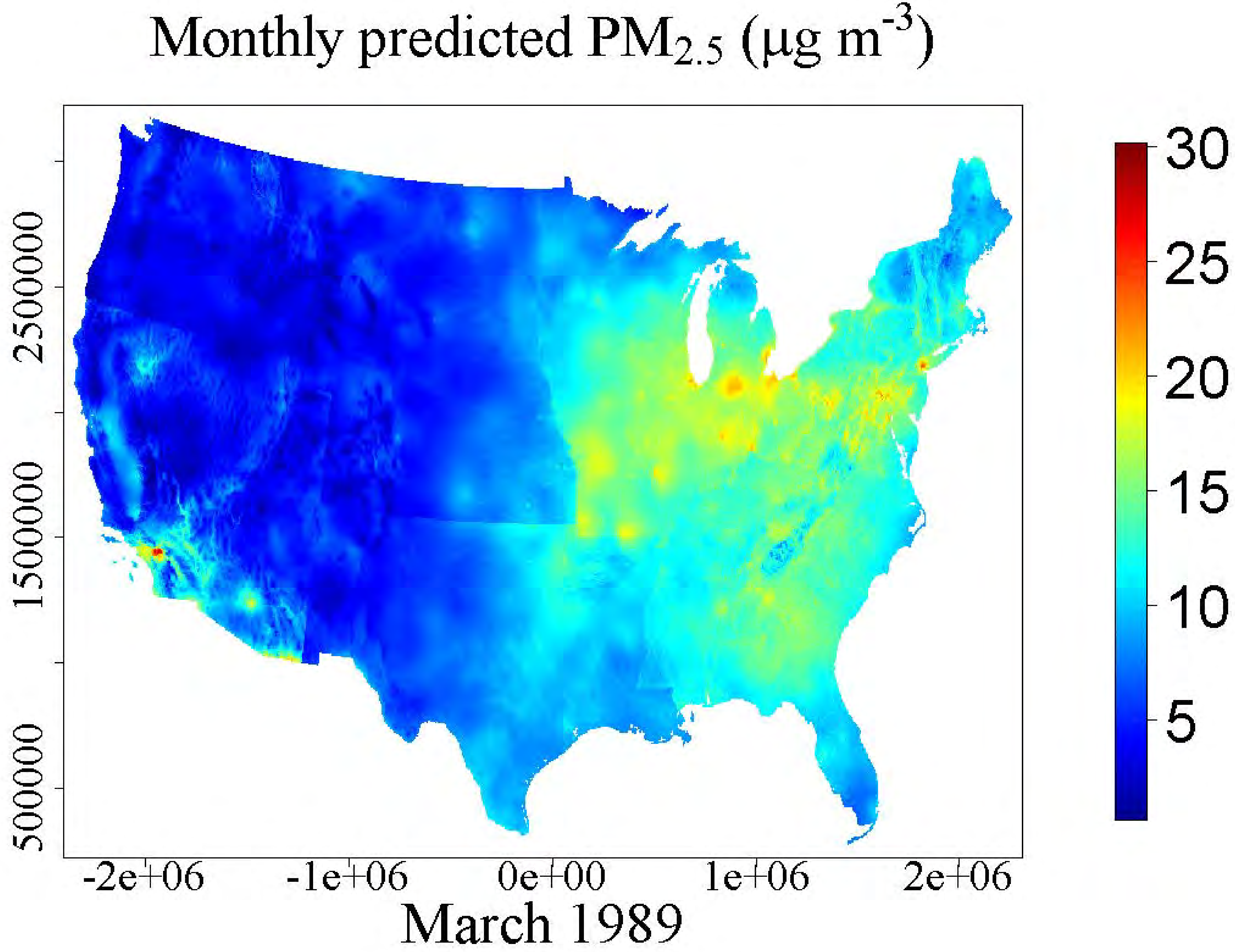
Monthly predicted PM2.5 (μg m-3)
25000000
15000000
5000000
-2e+06
-1e+06
0e+00
1e+06
2e+06
March 1989
30
25
20
15
10
5

Monthly predicted $PM_{2.5}$ ($\mu g\ m^{-3}$)

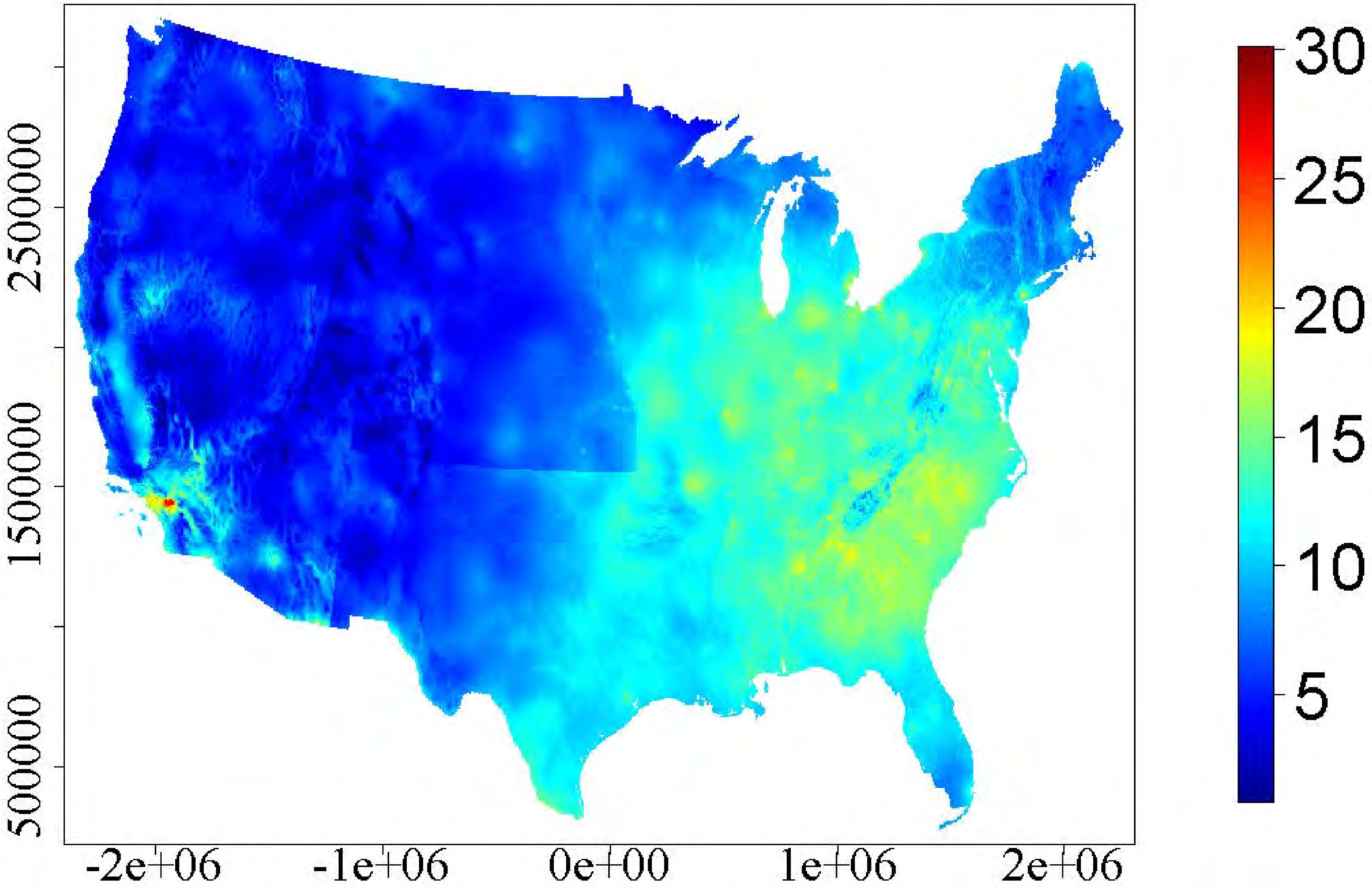

April 1989

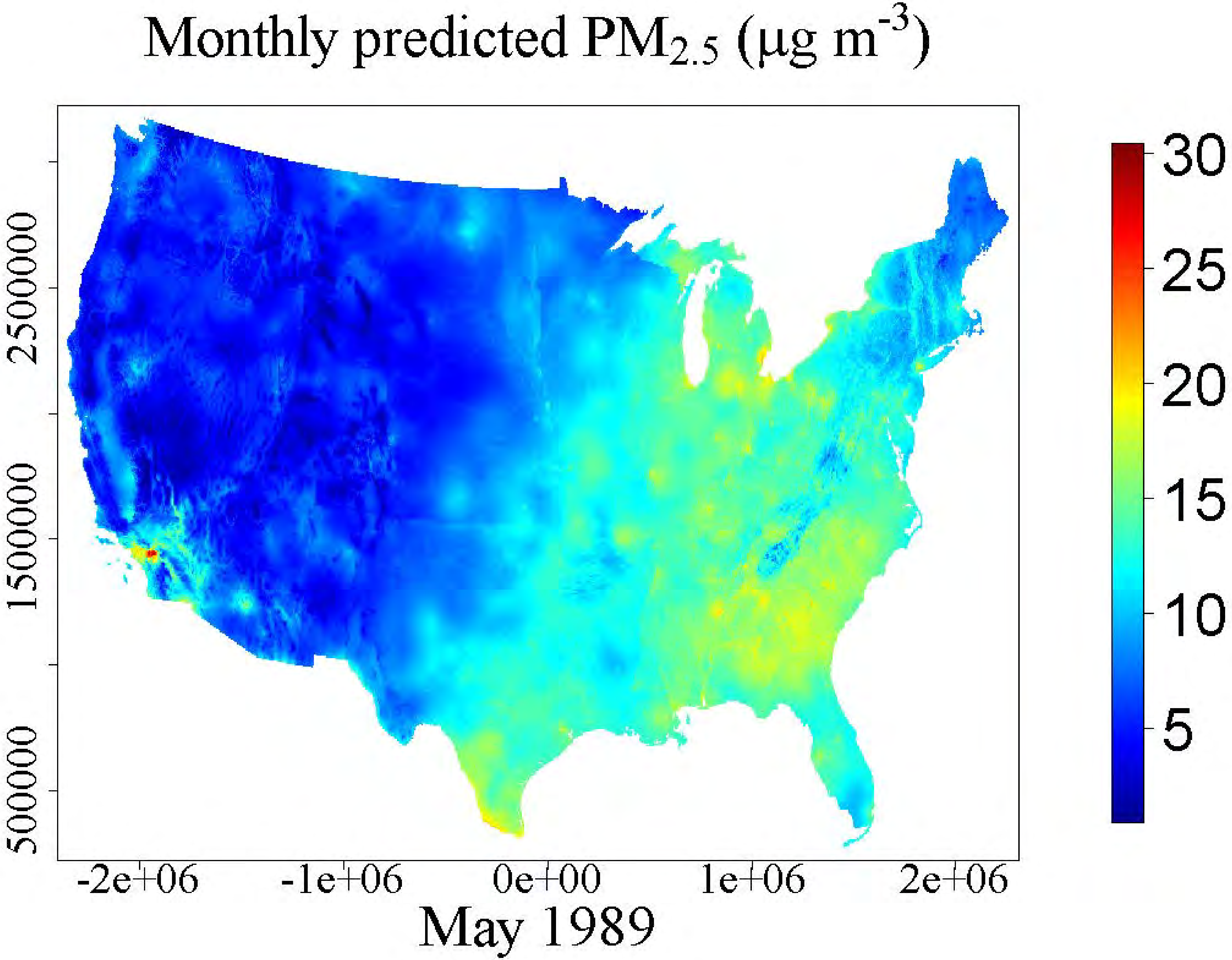

Monthly predicted PM2.5 (μg m-3)
25000000
15000000
5000000
-2e+06
-1e+06
0e+00
1e+06
2e+06
May 1989
30
25
20
15
10
5

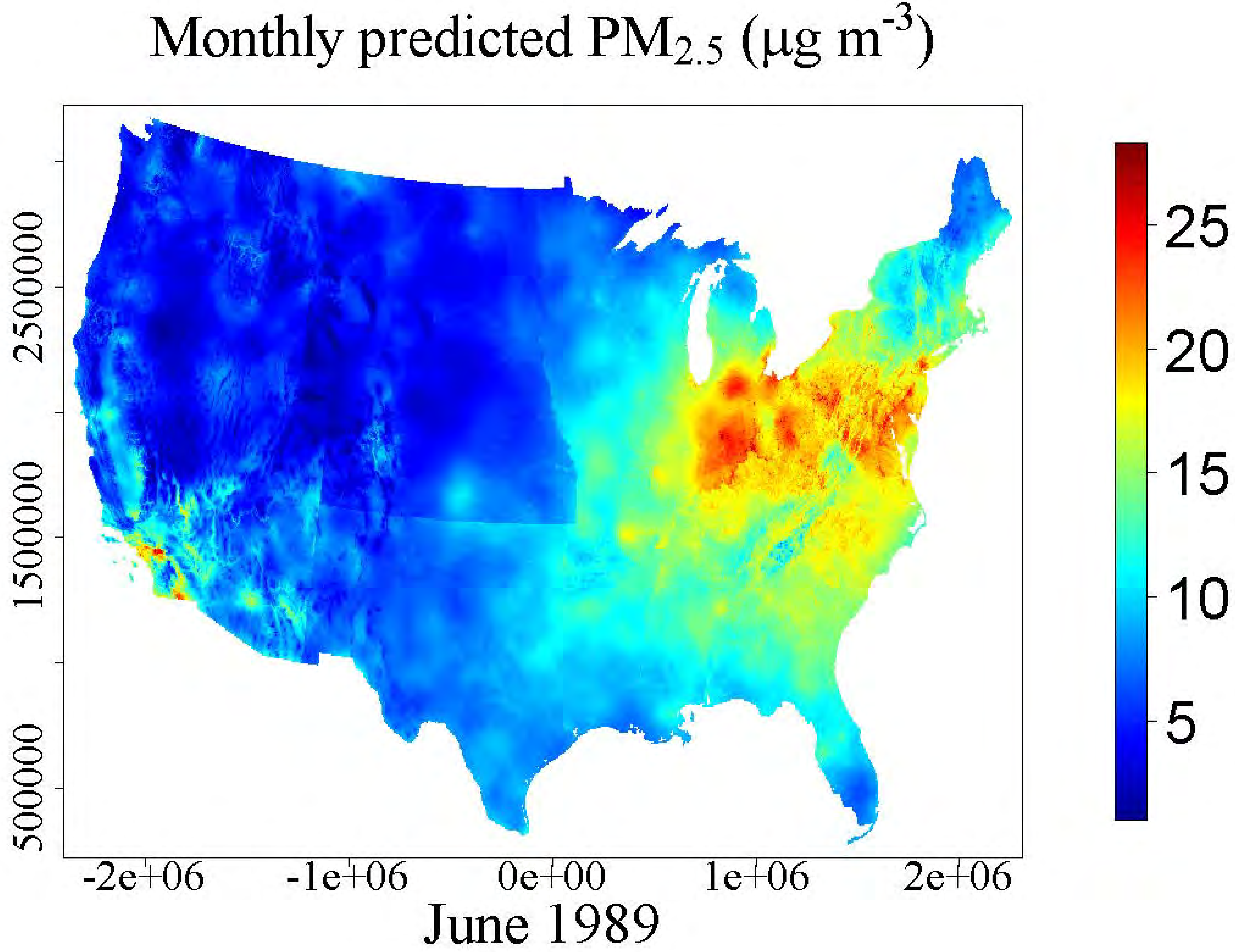
Monthly predicted PM2.5 (μg m-3)
25000000
15000000
5000000
-2e+06
-1e+06
0e+00
1e+06
2e+06
June 1989
25
20
15
10
5

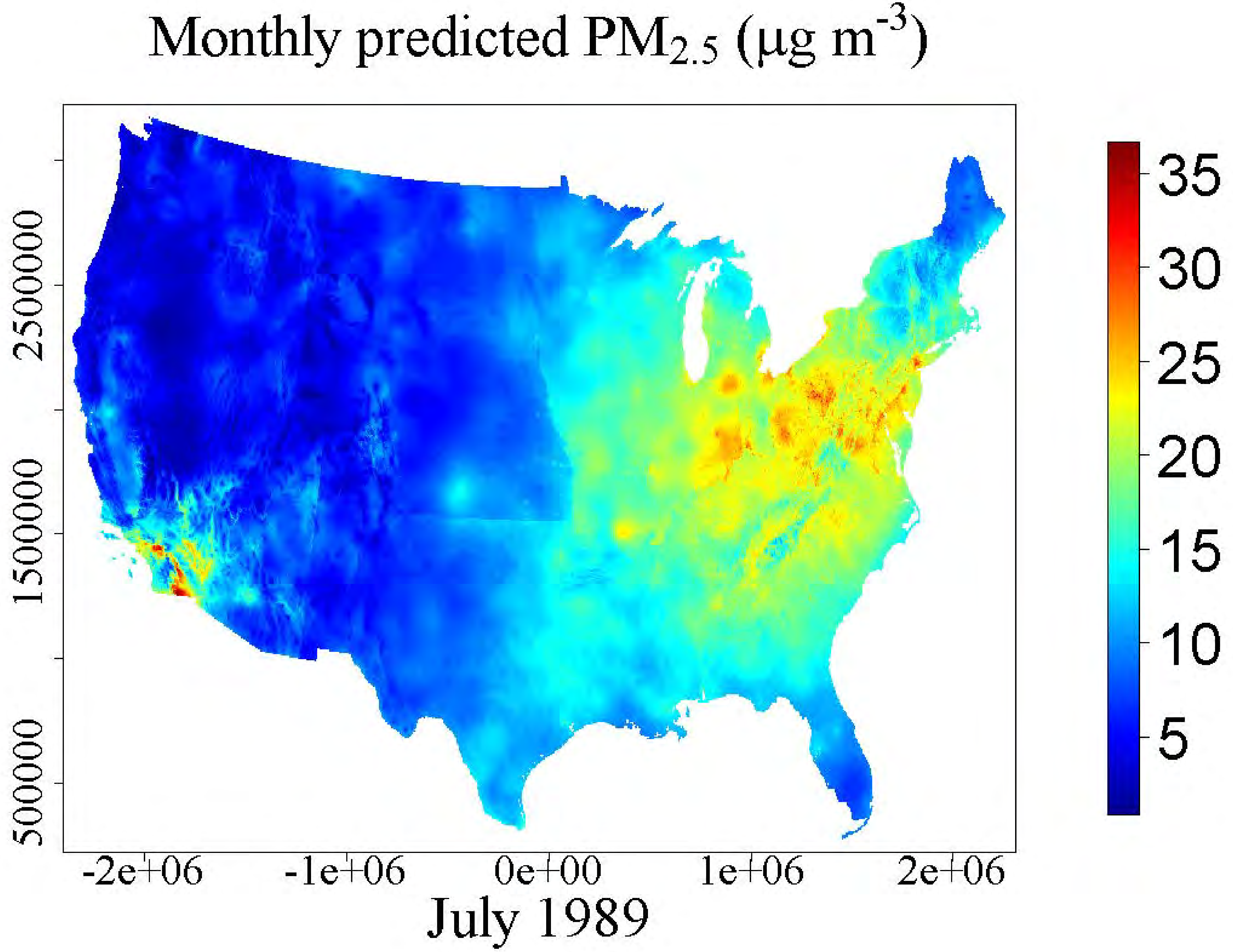
Monthly predicted PM2.5 (μg m-3)
25000000
15000000
5000000
-2e+06
-1e+06
0e+00
1e+06
2e+06
35
30
25
20
15
10
5
July 1989

Monthly predicted $PM_{2.5}$ ($\mu g\ m^{-3}$)

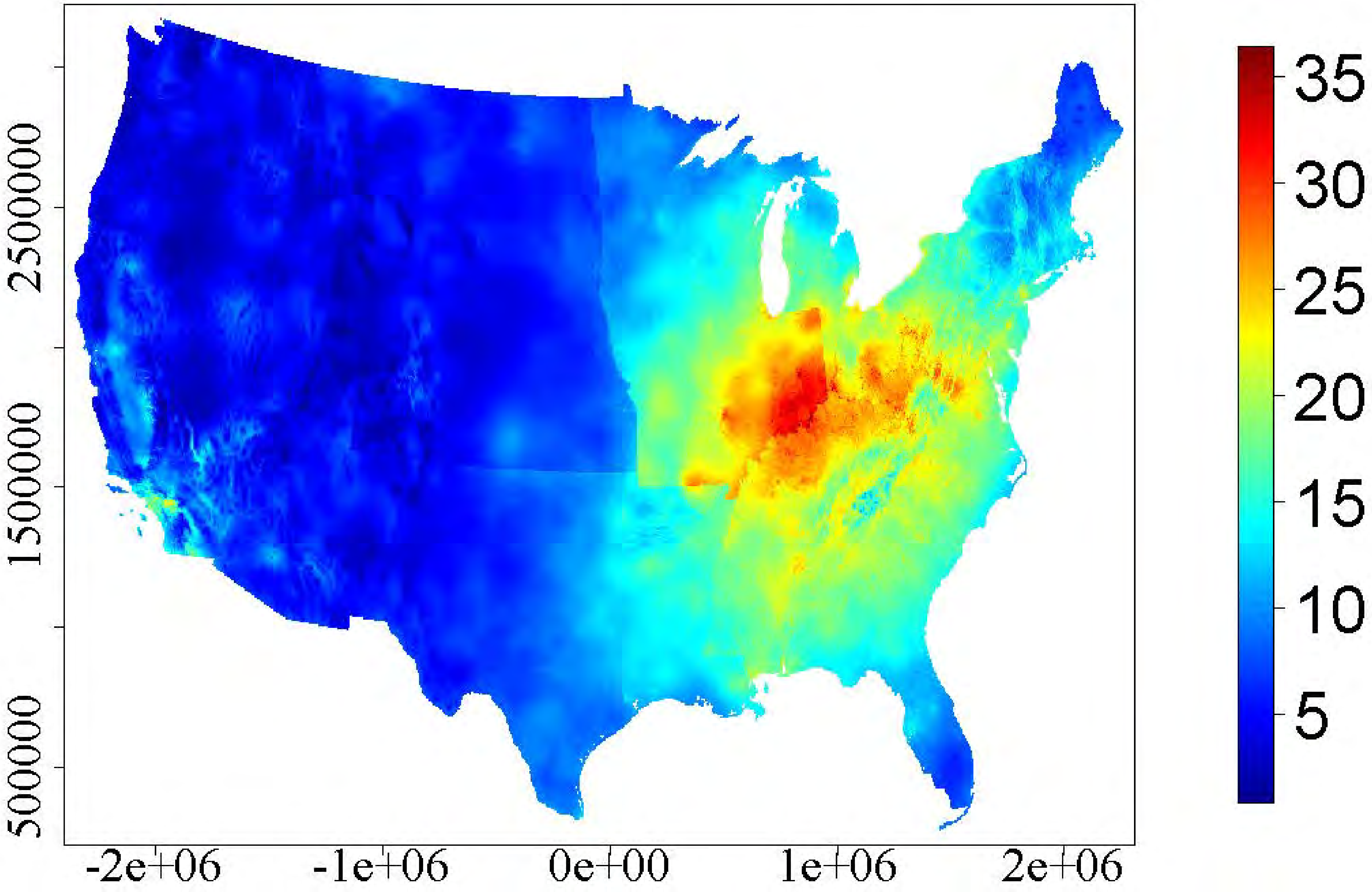

August 1989

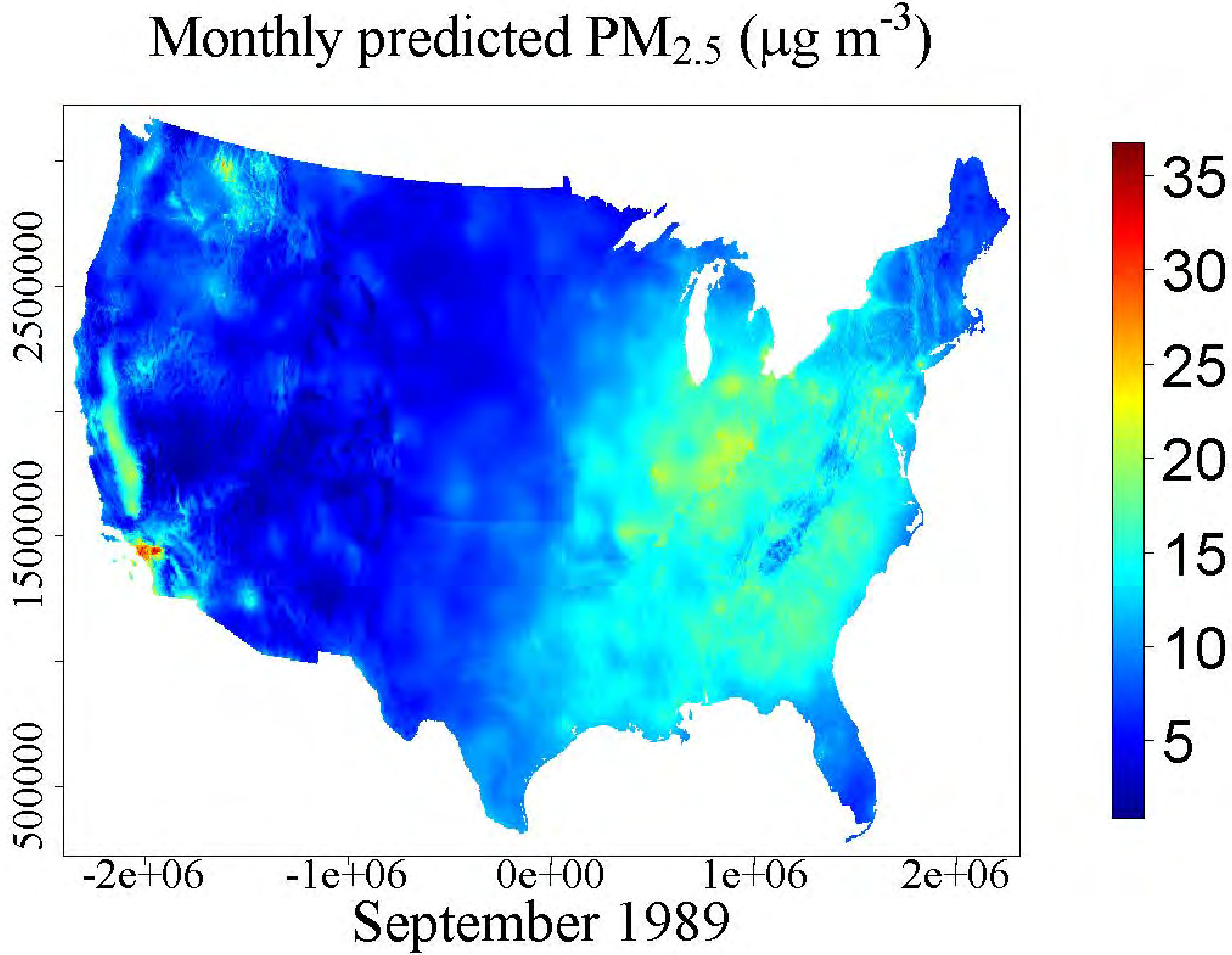
Monthly predicted PM2.5 (μg m-3)
25000000
15000000
5000000
-2e+06
-1e+06
0e+00
1e+06
2e+06
35
30
25
20
15
10
5
September 1989

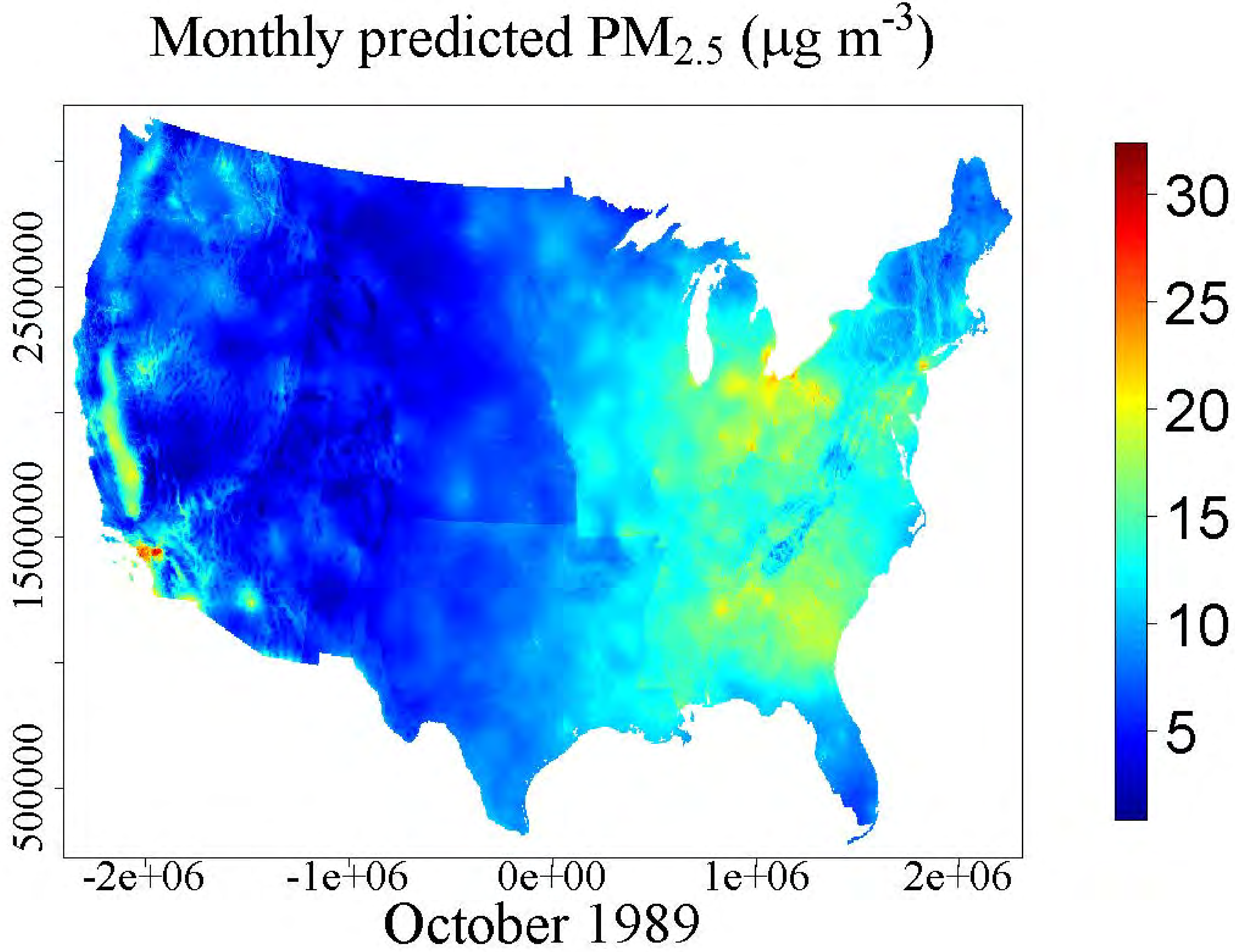

Monthly predicted PM2.5 (μg m-3)
25000000
15000000
5000000
-2e+06
-1e+06
0e+00
1e+06
2e+06
30
25
20
15
10
5
October 1989

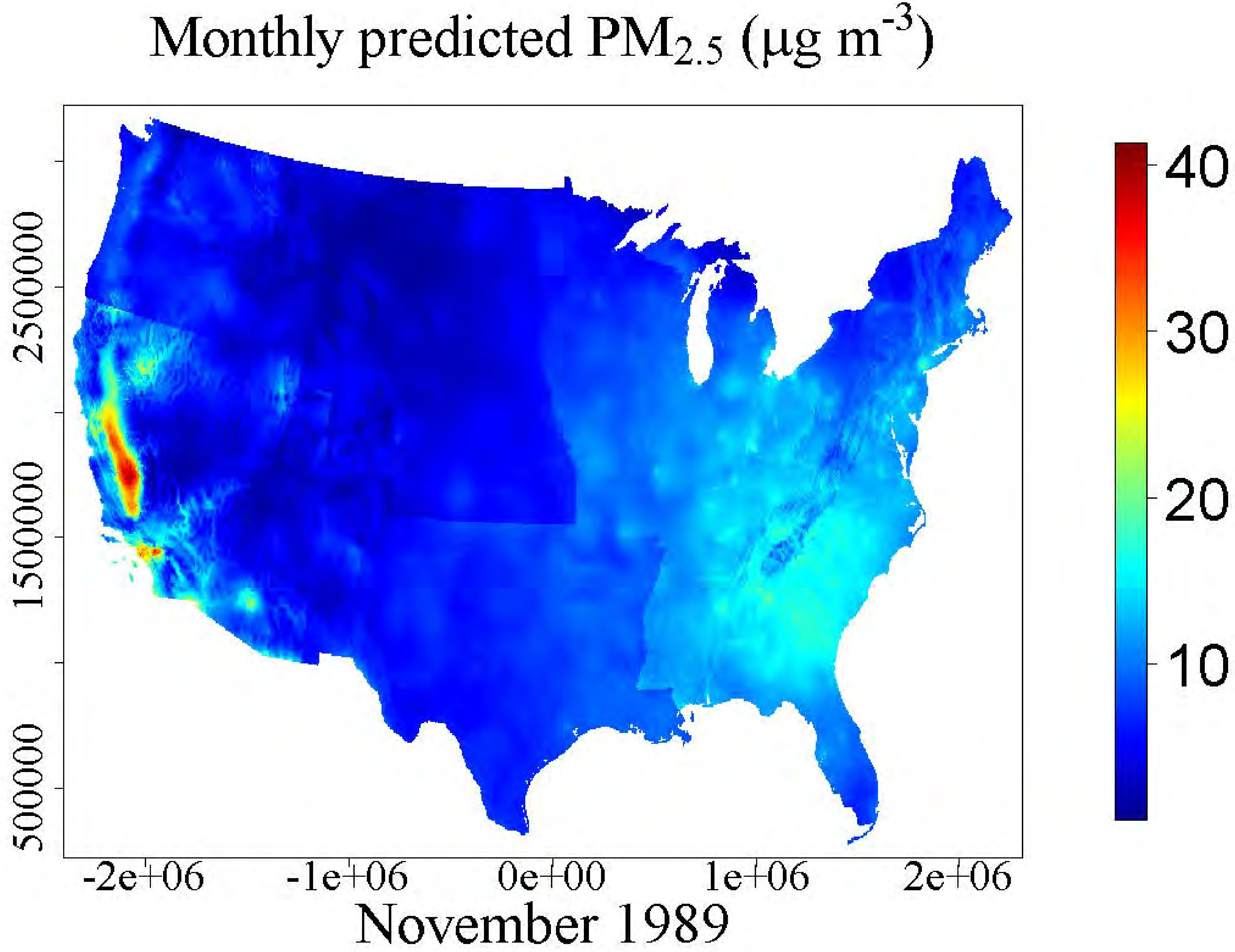

Monthly predicted $PM_{2.5}$ ($\mu g\ m^{-3}$)
5000000
15000000
25000000
-2e+06
-1e+06
0e+00
1e+06
2e+06
10
20
30
40
November 1989

Monthly predicted $PM_{2.5}$ ($\mu g\ m^{-3}$)

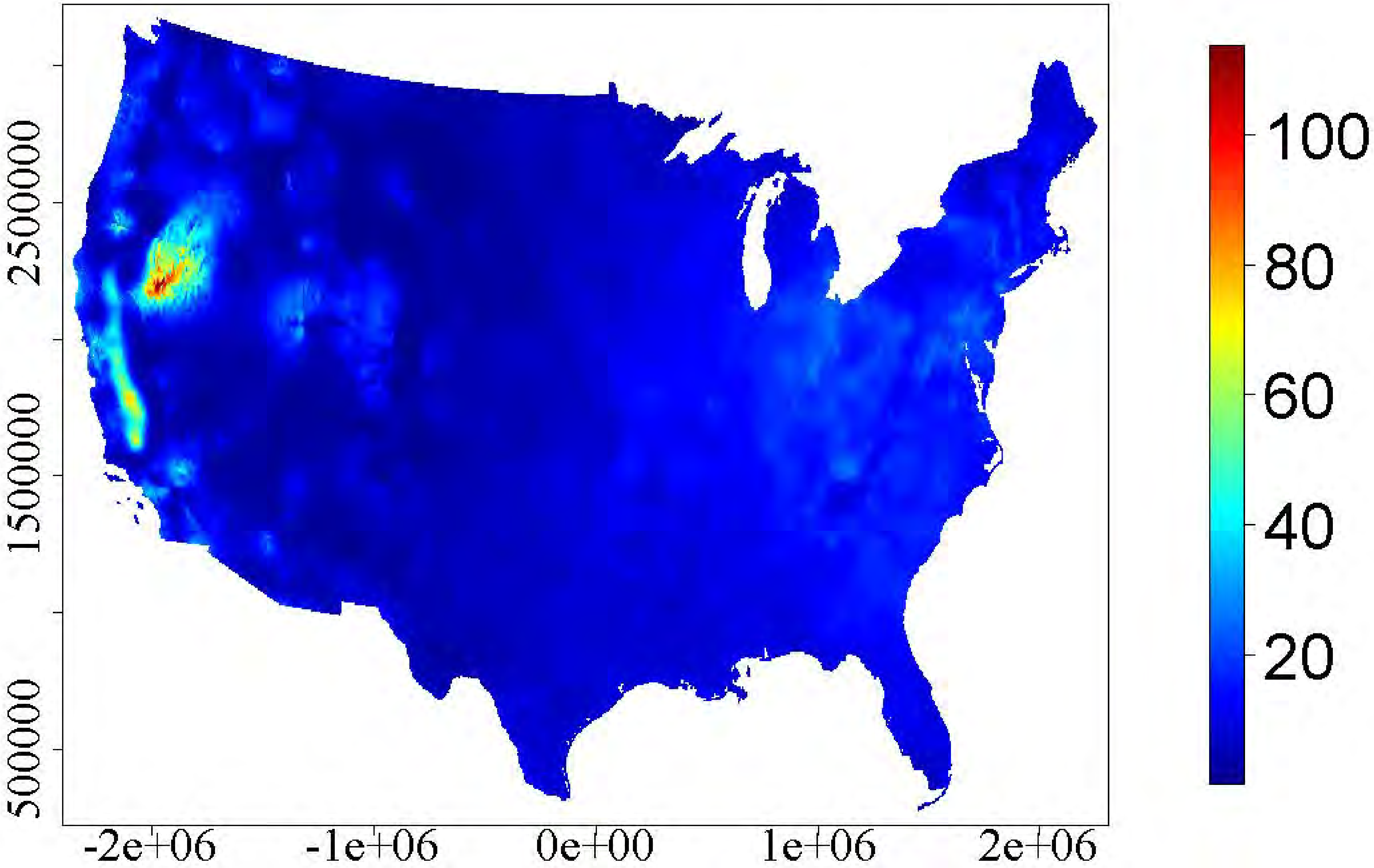

December 1989

Monthly predicted $PM_{2.5}$ ($\mu g\ m^{-3}$)

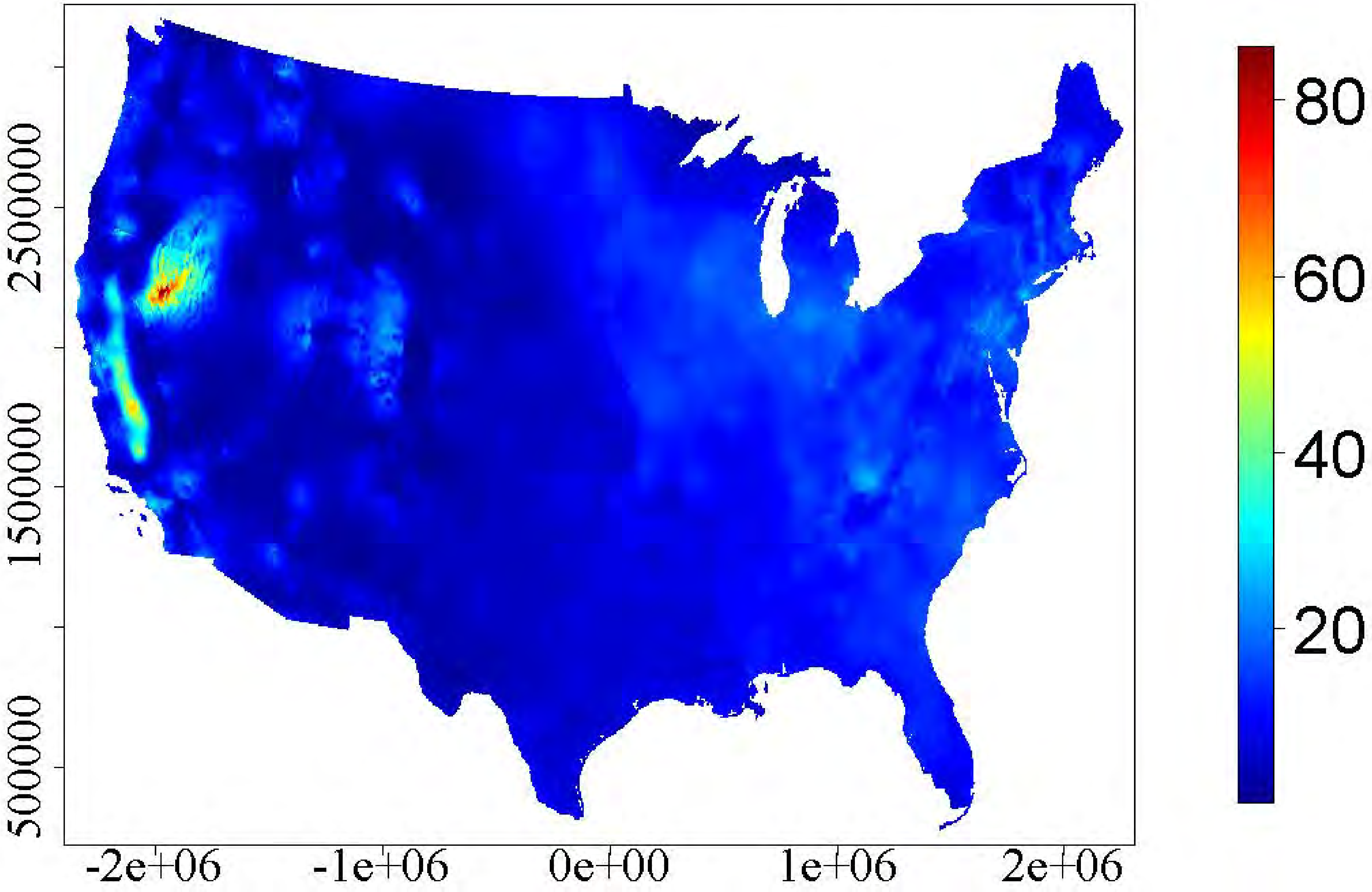

January 1990

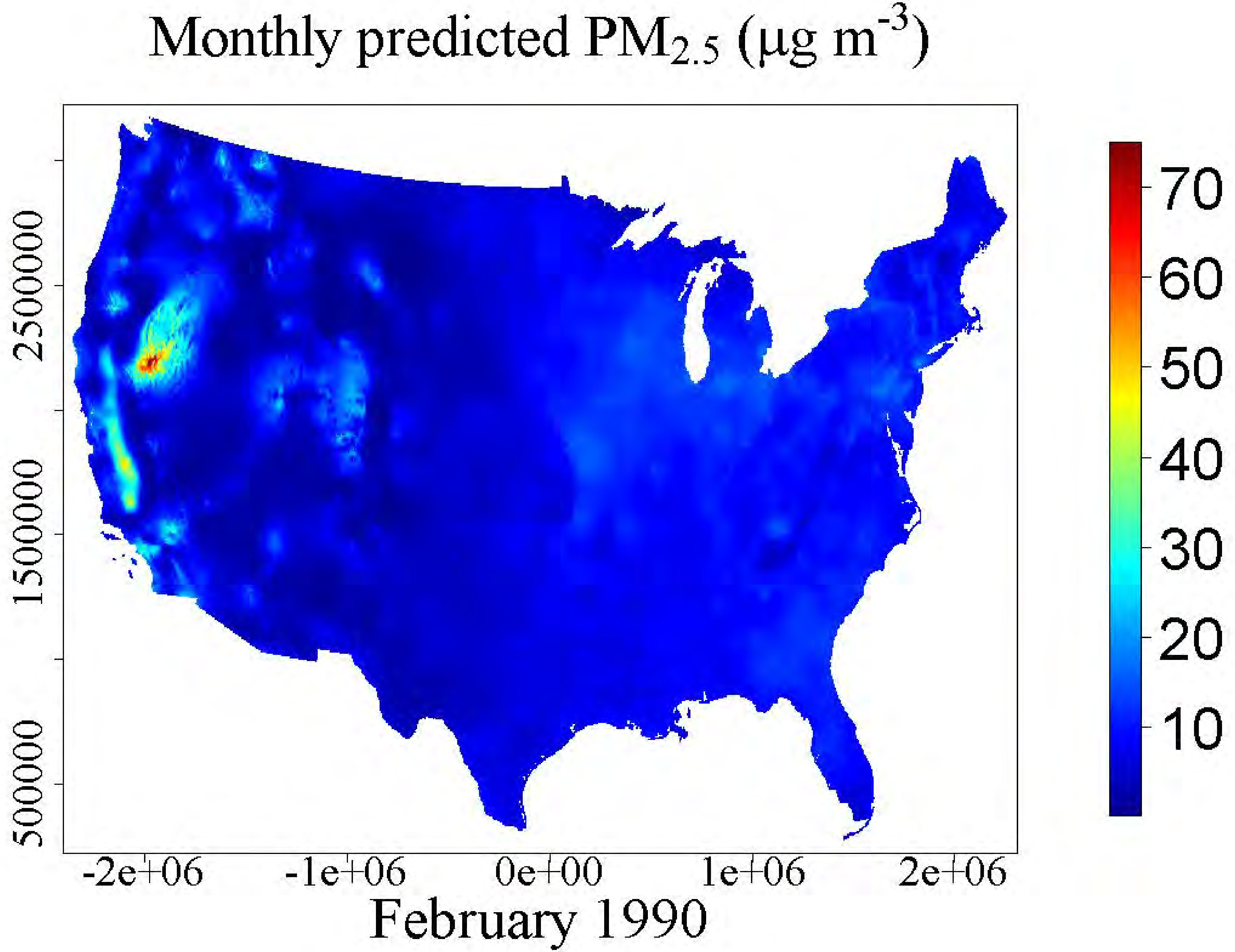
Monthly predicted $PM_{2.5}$ ($\mu g\ m^{-3}$)
2500000
1500000
500000
-2e+06
-1e+06
0e+00
1e+06
2e+06
70
60
50
40
30
20
10
February 1990

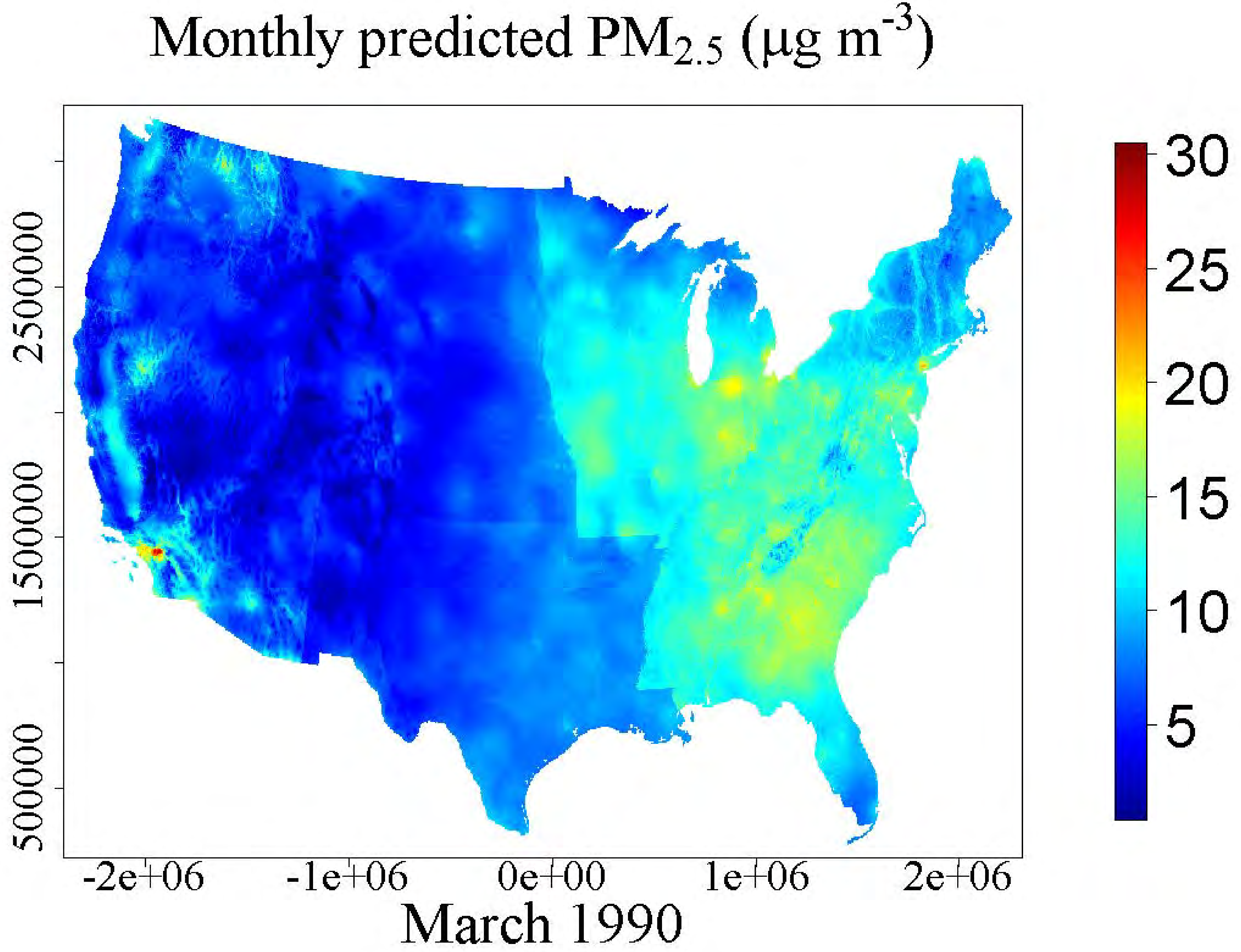
Monthly predicted PM2.5 (μg m-3)
25000000
15000000
5000000
-2e+06
-1e+06
0e+00
1e+06
2e+06
March 1990
30
25
20
15
10
5

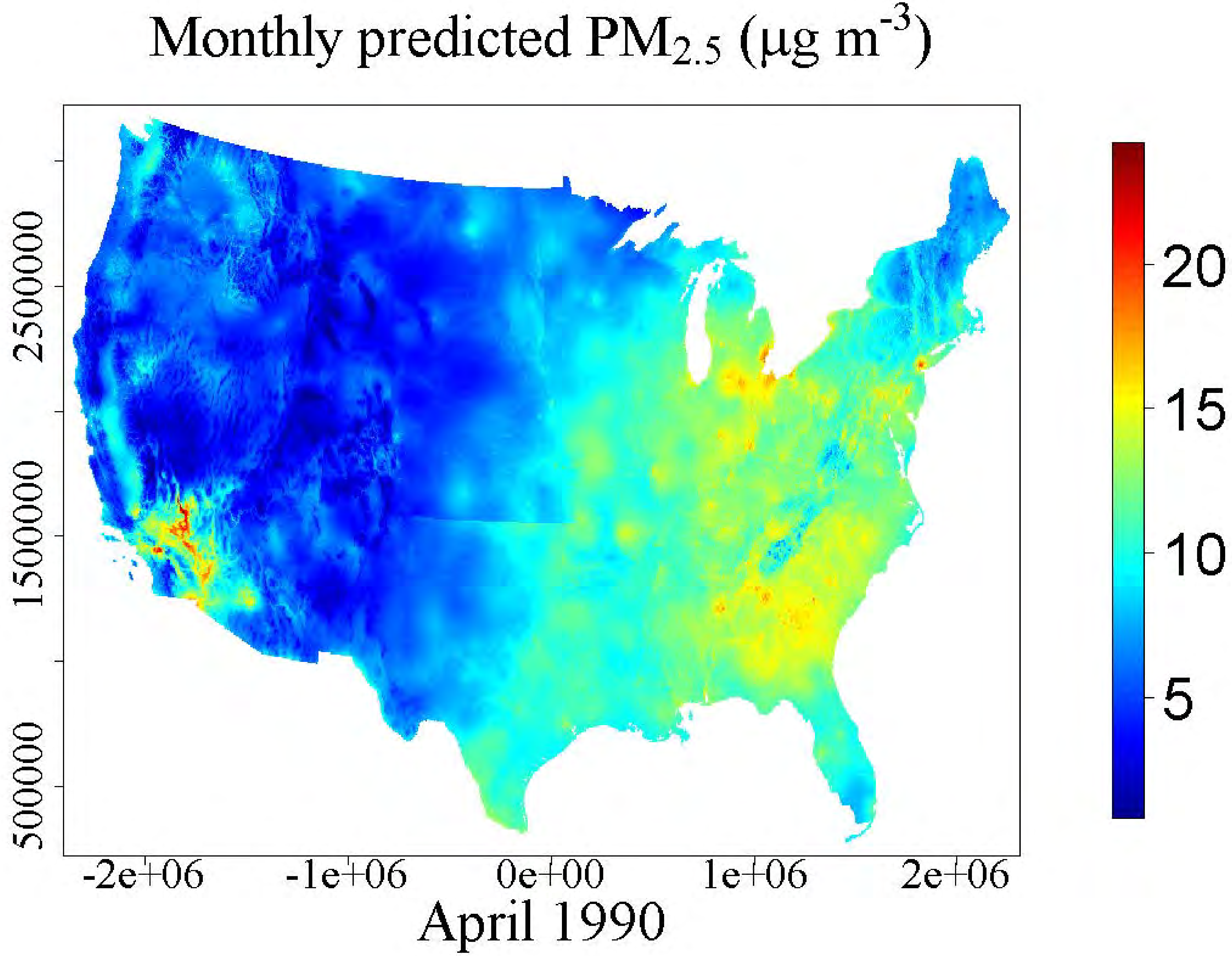
Monthly predicted PM2.5 (μg m-3)
25000000
15000000
5000000
-2e+06
-1e+06
0e+00
1e+06
2e+06
April 1990
20
15
10
5

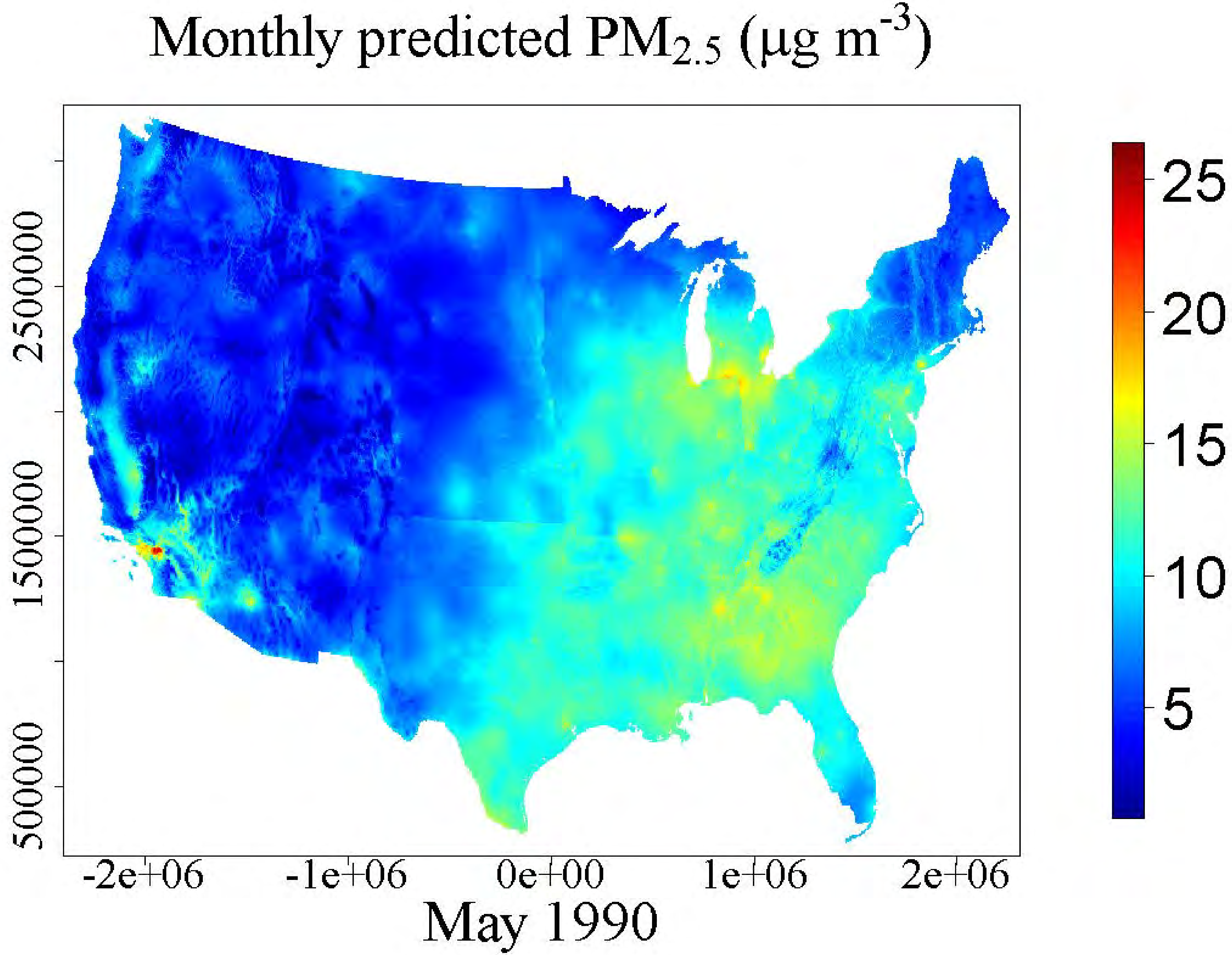
Monthly predicted PM2.5 (µg m-3)
25000000
15000000
5000000
-2e+06
-1e+06
0e+00
1e+06
2e+06
May 1990
25
20
15
10
5

Monthly predicted $PM_{2.5}$ ($\mu g\ m^{-3}$)

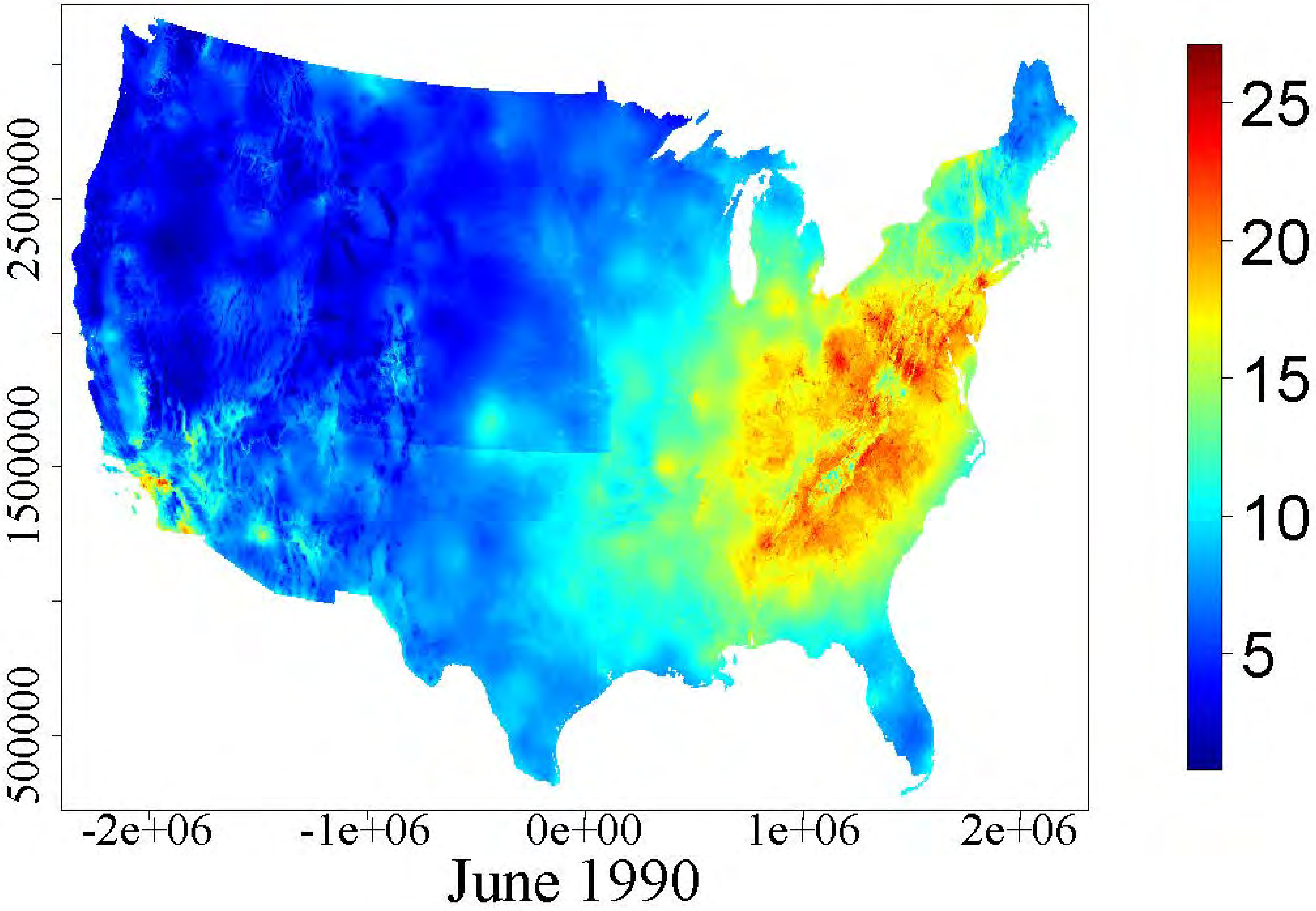

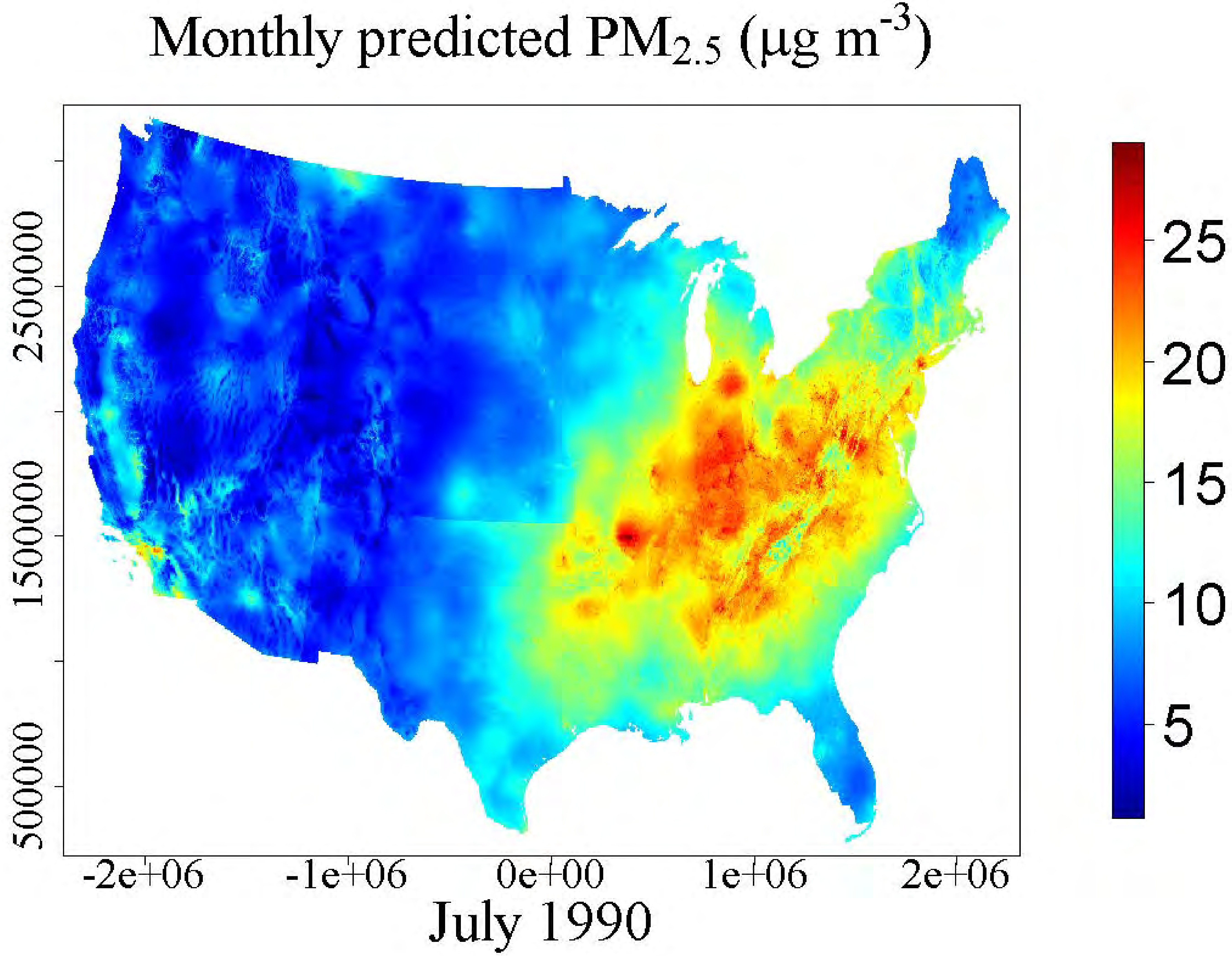

Monthly predicted PM2.5 (μg m-3)
25000000
15000000
5000000
-2e+06
-1e+06
0e+00
1e+06
2e+06
July 1990
25
20
15
10
5

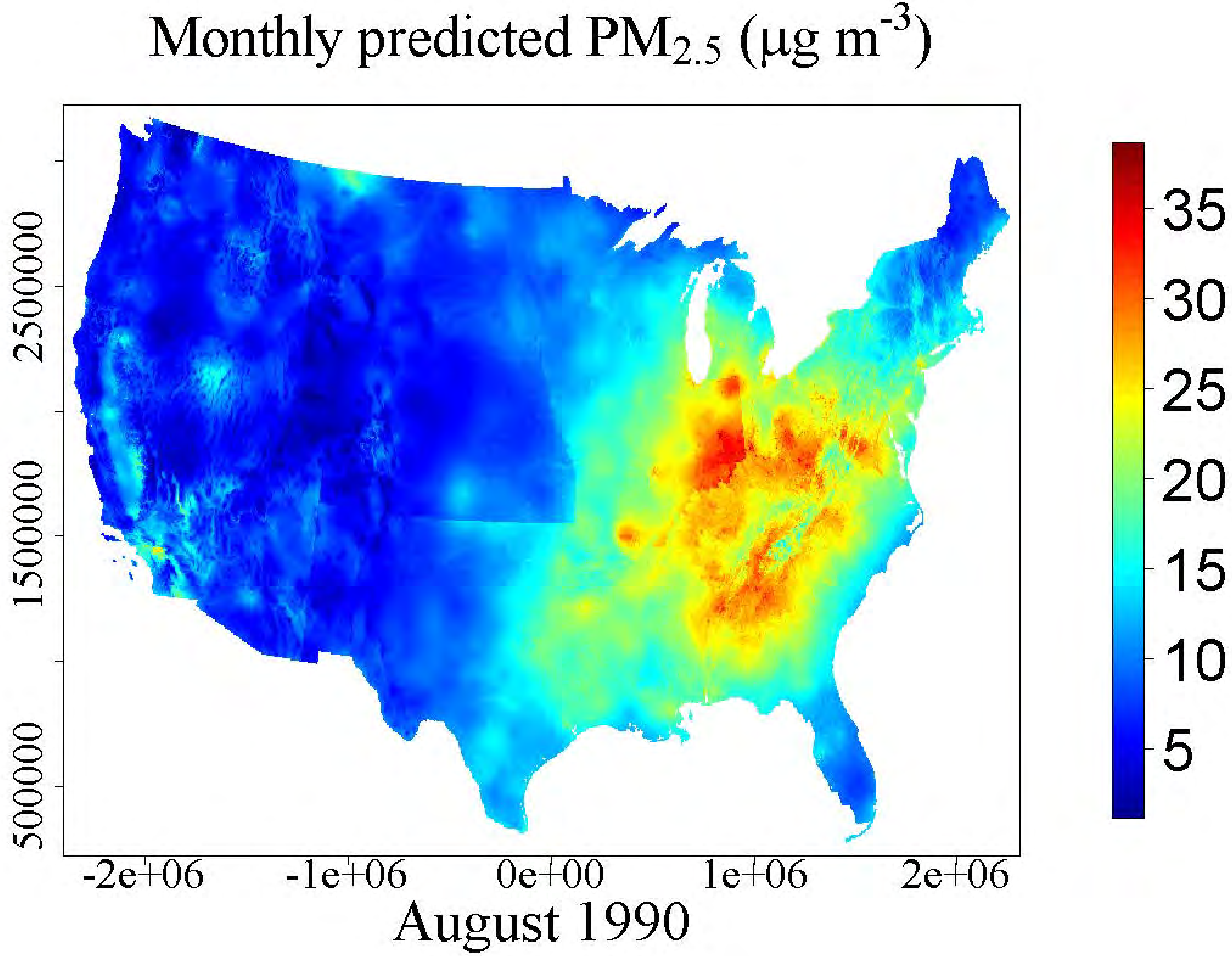
Monthly predicted PM2.5 (μg m-3)
25000000
15000000
5000000
-2e+06
-1e+06
0e+00
1e+06
2e+06
August 1990
35
30
25
20
15
10
5

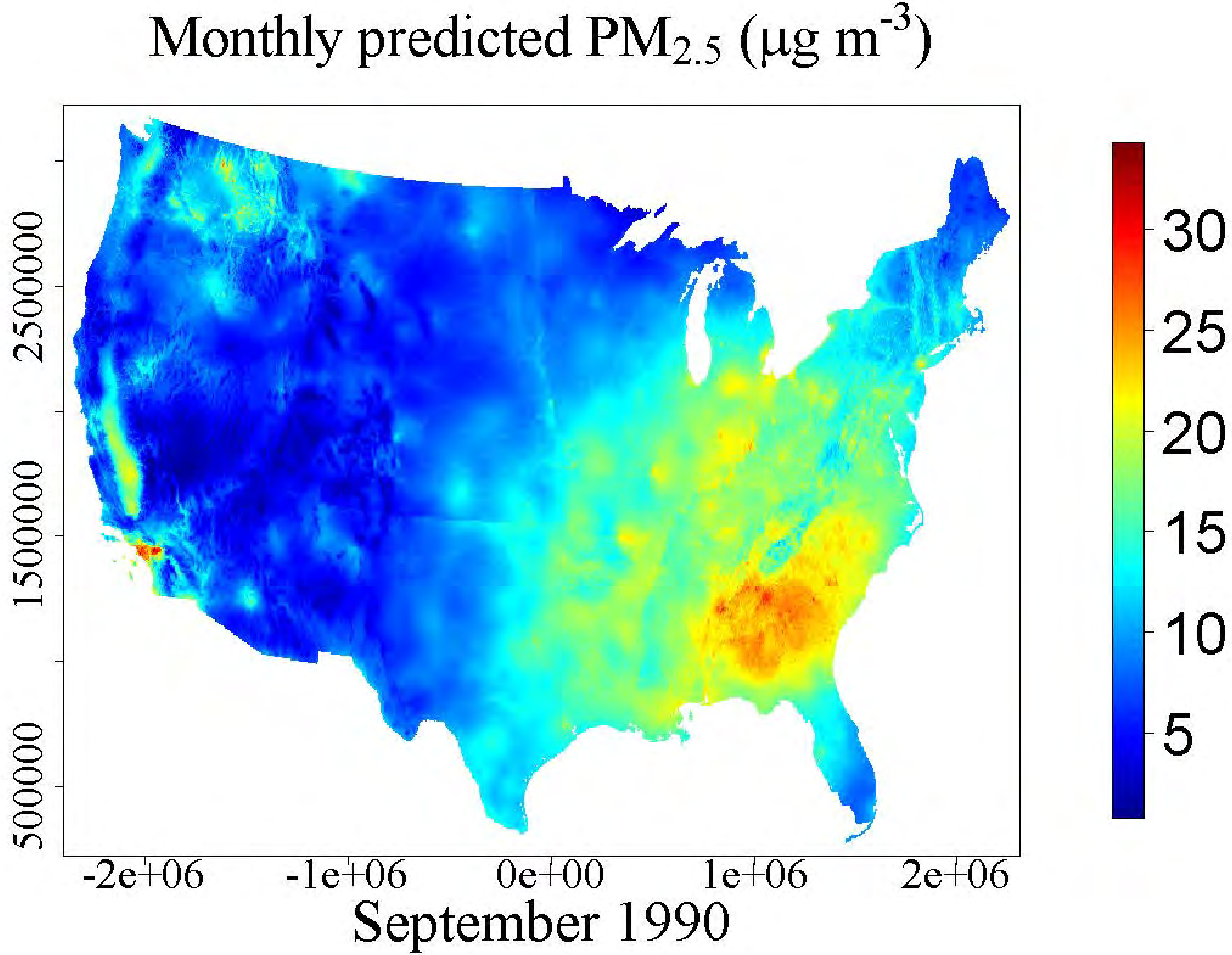

Monthly predicted PM2.5 (μg m-3)
25000000
15000000
5000000
-2e+06
-1e+06
0e+00
1e+06
2e+06
September 1990
30
25
20
15
10
5

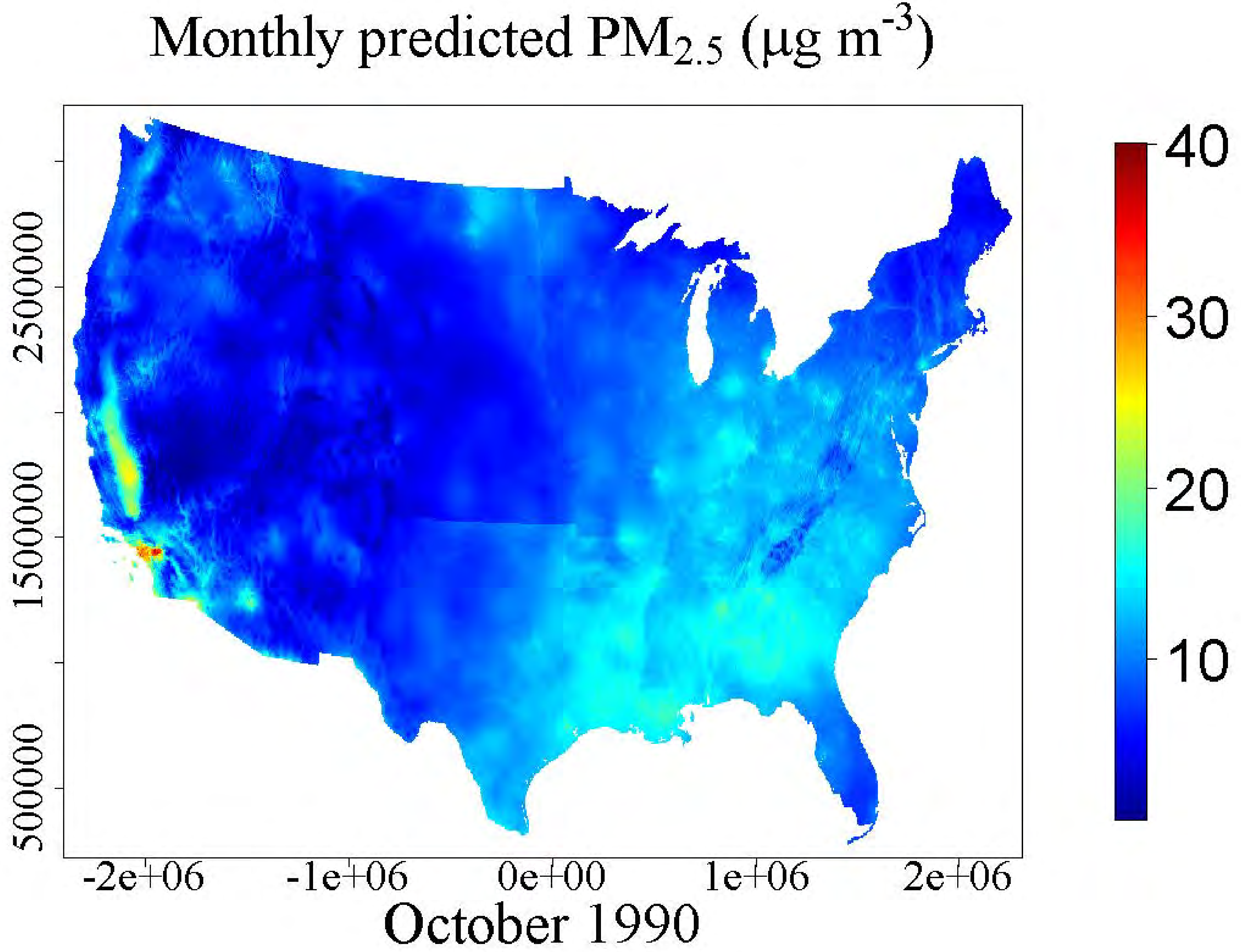

Monthly predicted $PM_{2.5}$ ($\mu g\ m^{-3}$)
25000000
15000000
5000000
-2e+06
-1e+06
0e+00
1e+06
2e+06
40
30
20
10
October 1990

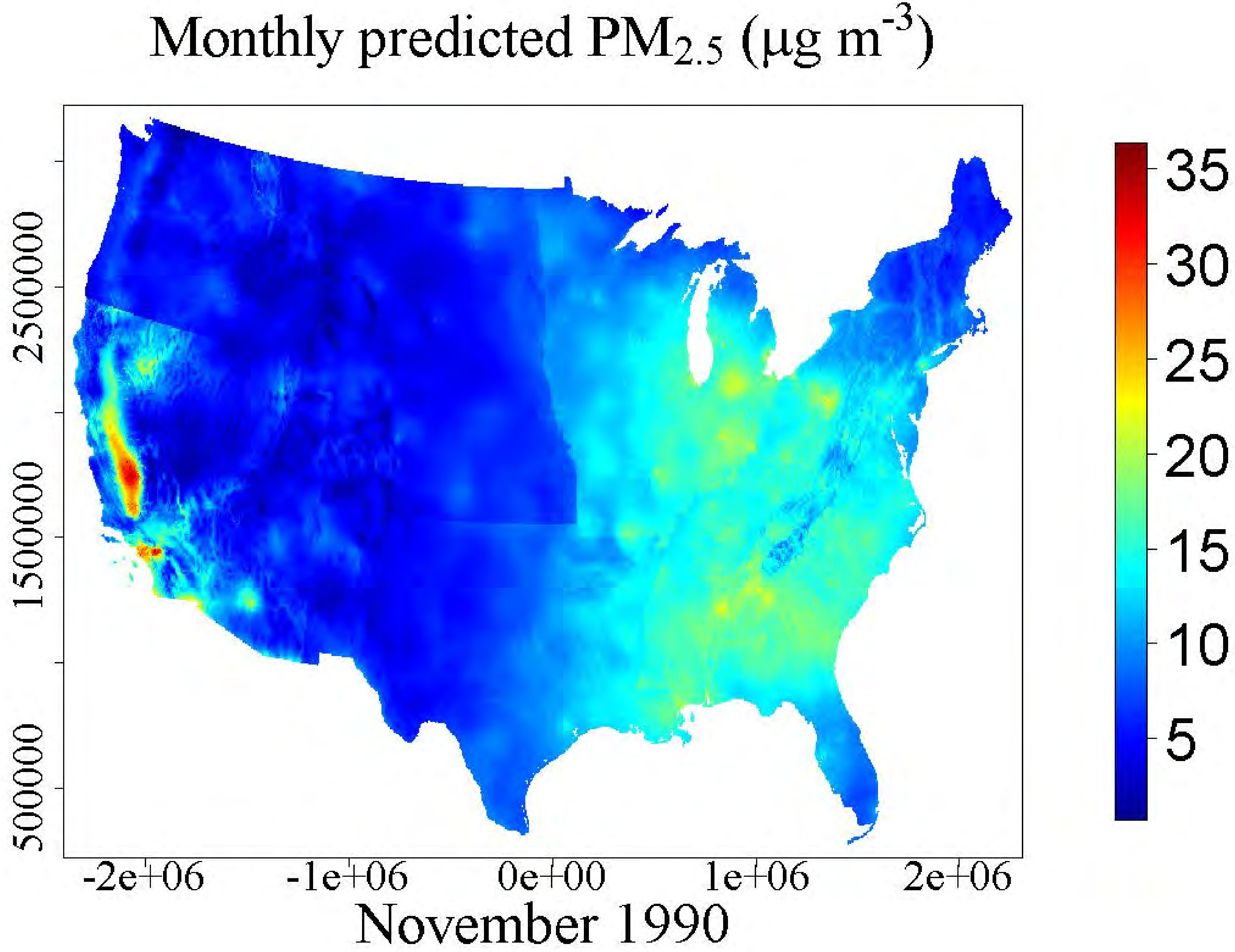
Monthly predicted PM2.5 (μg m-3)
25000000
15000000
5000000
-2e+06
-1e+06
0e+00
1e+06
2e+06
November 1990
35
30
25
20
15
10
5

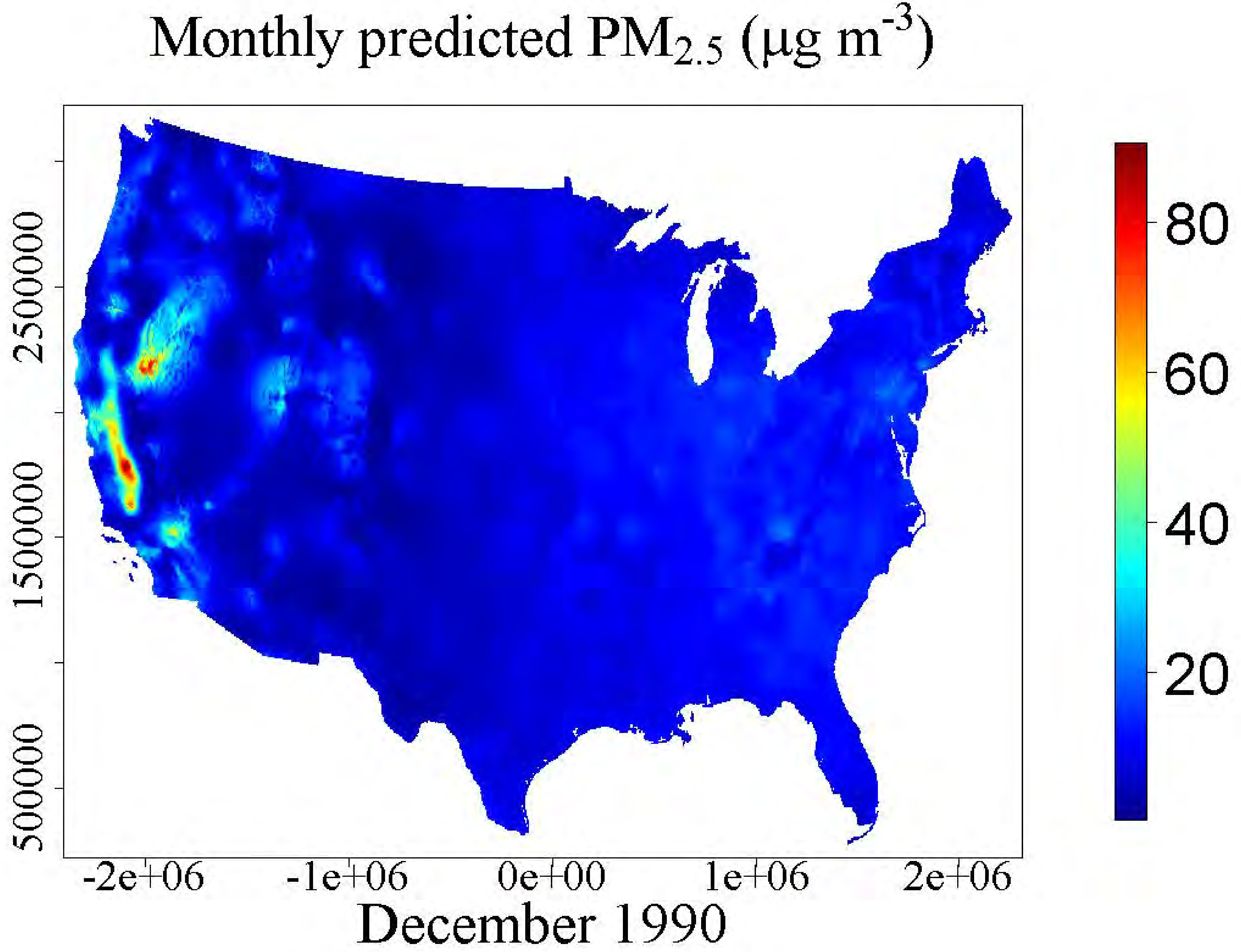

Monthly predicted PM2.5 (μg m-3)
25000000
15000000
5000000
-2e+06
-1e+06
0e+00
1e+06
2e+06
80
60
40
20
December 1990

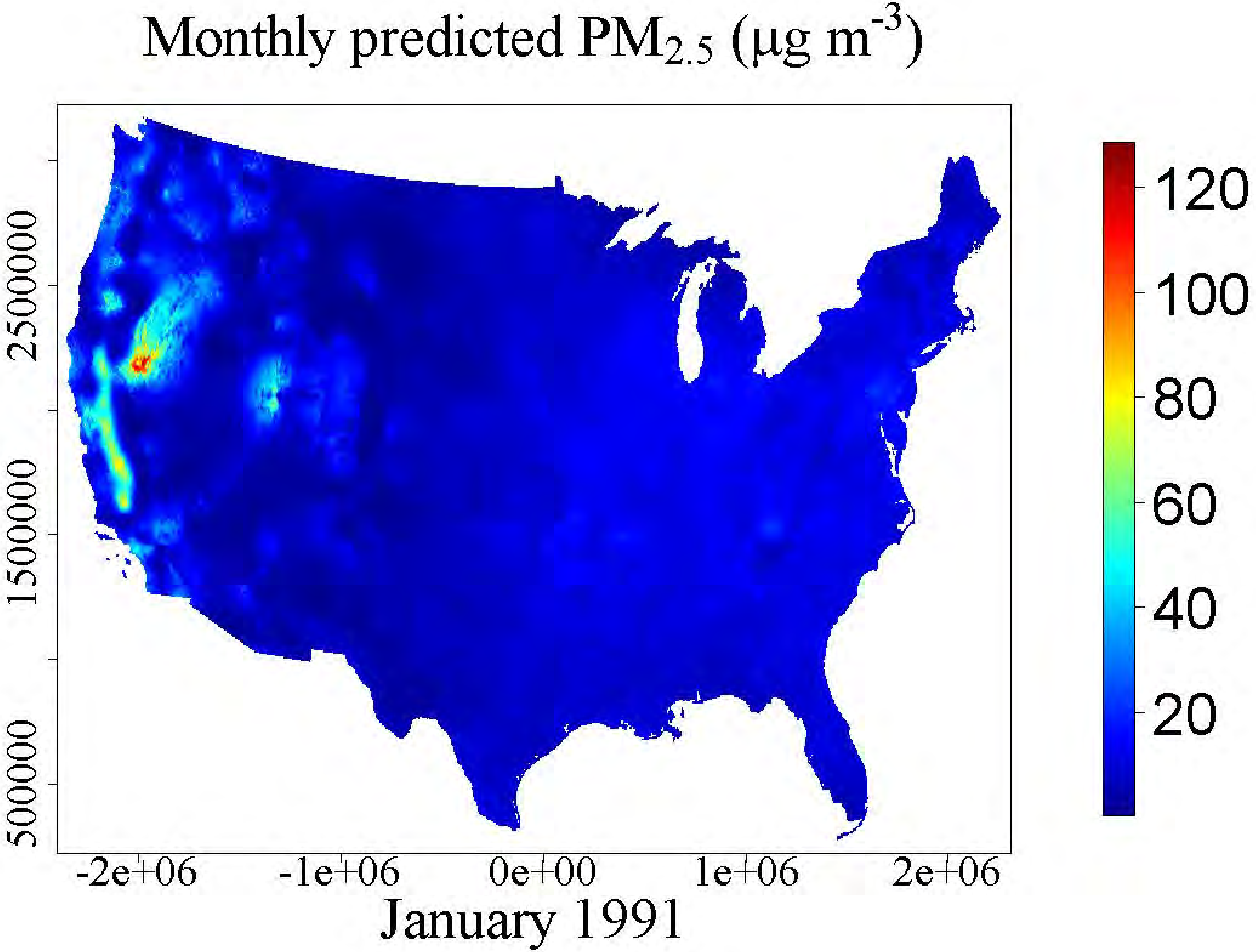
Monthly predicted $PM_{2.5}$ ($\mu g\ m^{-3}$)
5000000
15000000
25000000
-2e+06
-1e+06
0e+00
1e+06
2e+06
120
100
80
60
40
20
January 1991

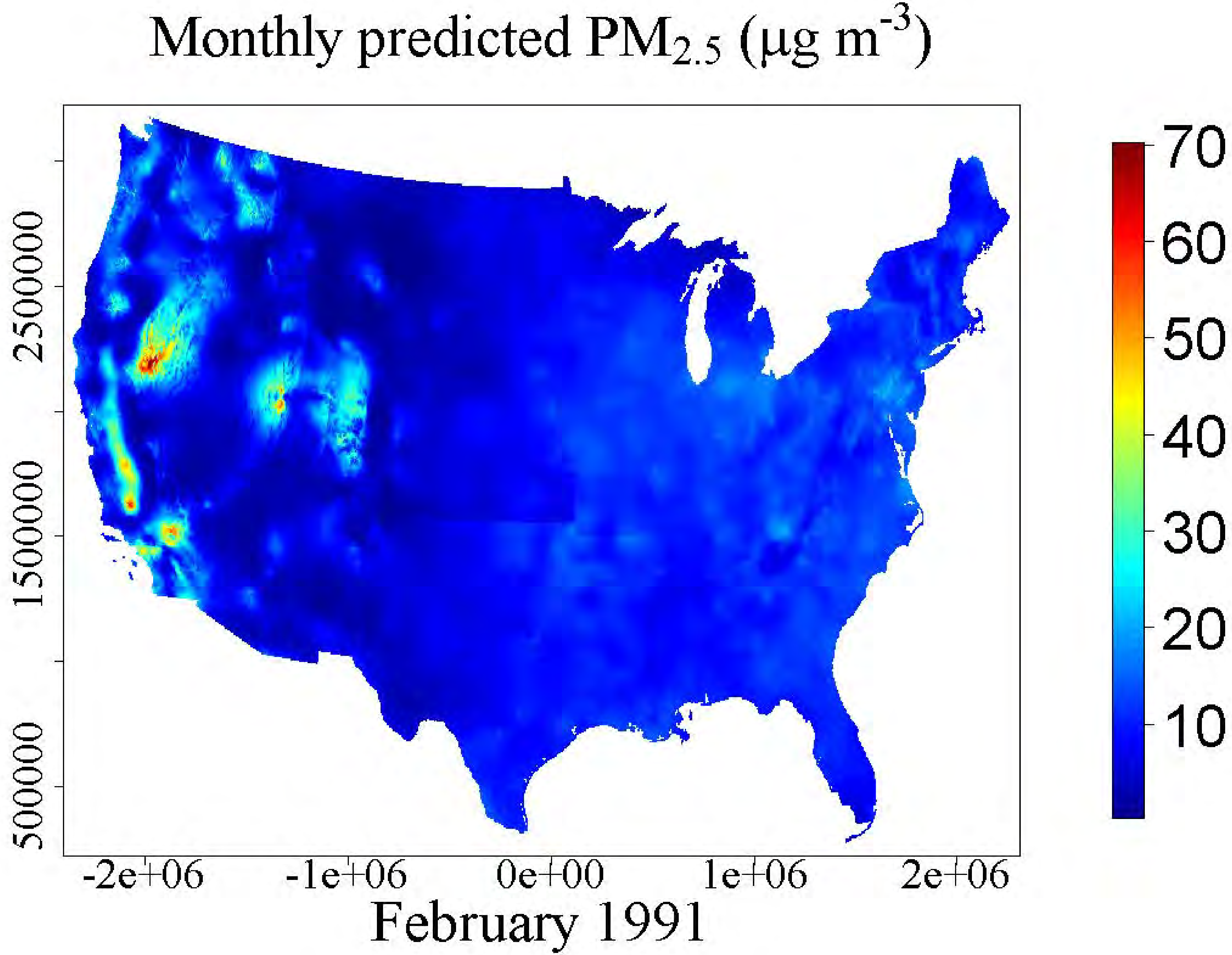
Monthly predicted PM2.5 (μg m-3)
25000000
15000000
5000000
-2e+06
-1e+06
0e+00
1e+06
2e+06
February 1991
70
60
50
40
30
20
10

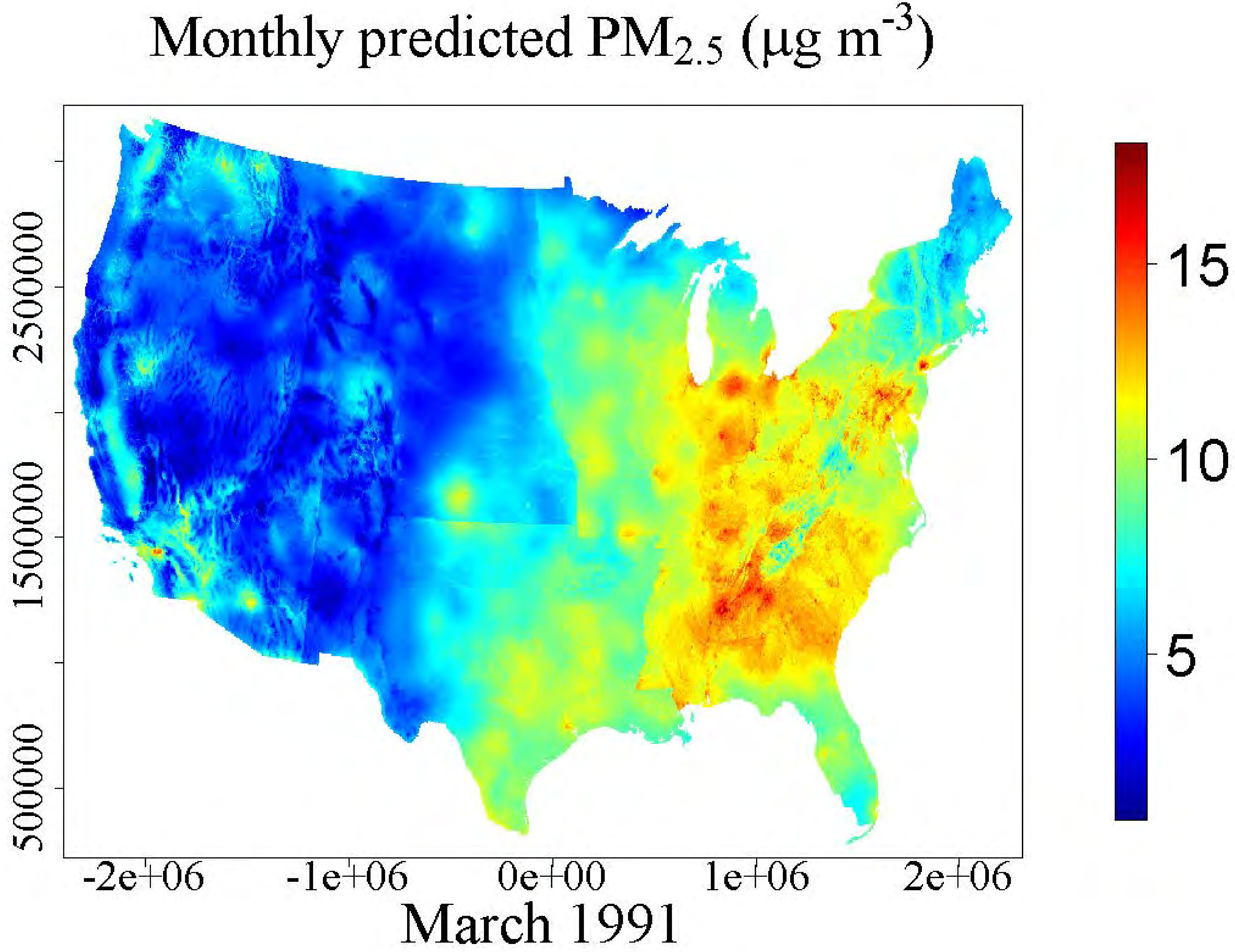
Monthly predicted PM2.5 (μg m-3)
25000000
15000000
5000000
-2e+06
-1e+06
0e+00
1e+06
2e+06
March 1991
15
10
5

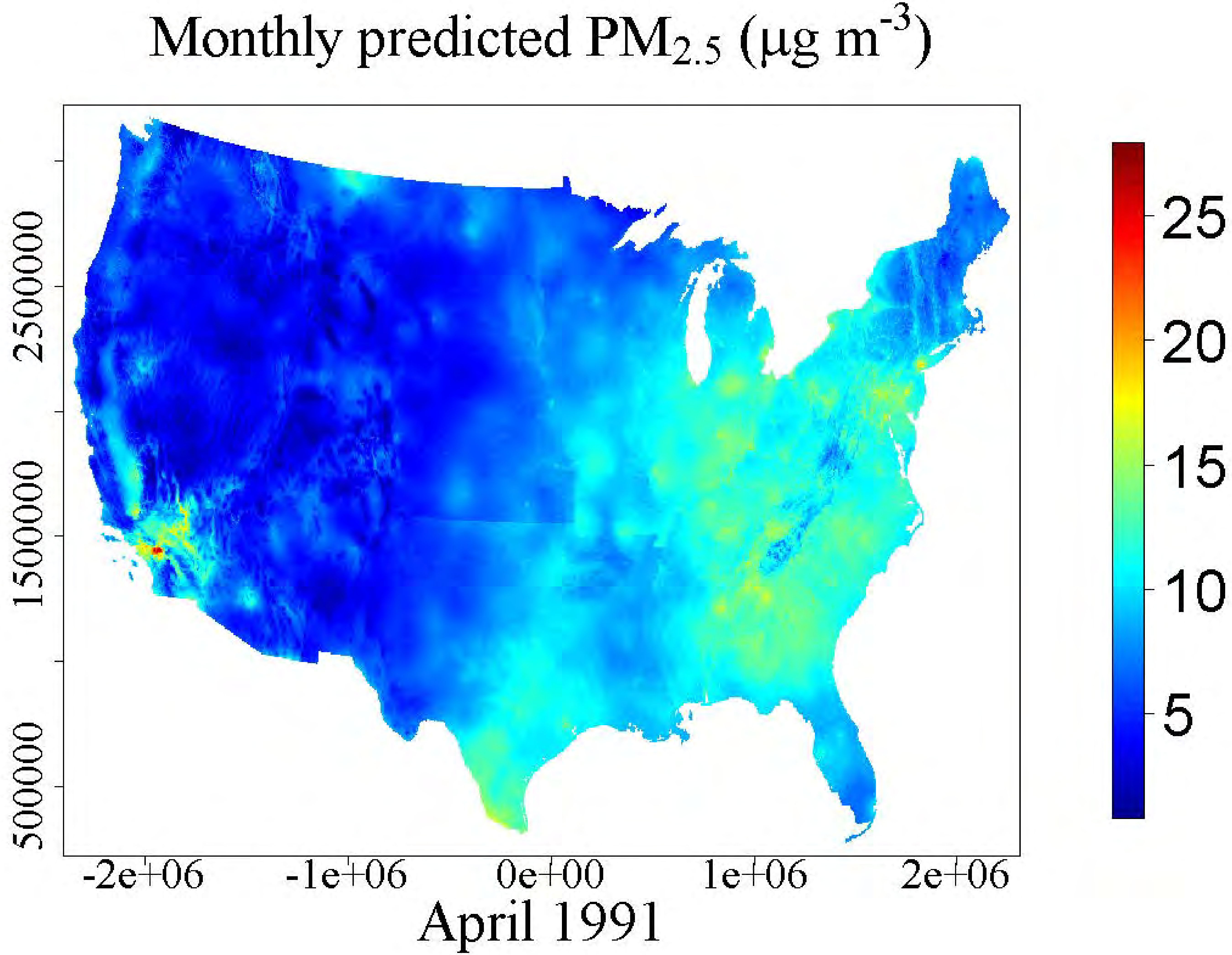
Monthly predicted PM2.5 (μg m-3)
25
20
15
10
5
25000000
15000000
5000000
-2e+06
-1e+06
0e+00
1e+06
2e+06
April 1991

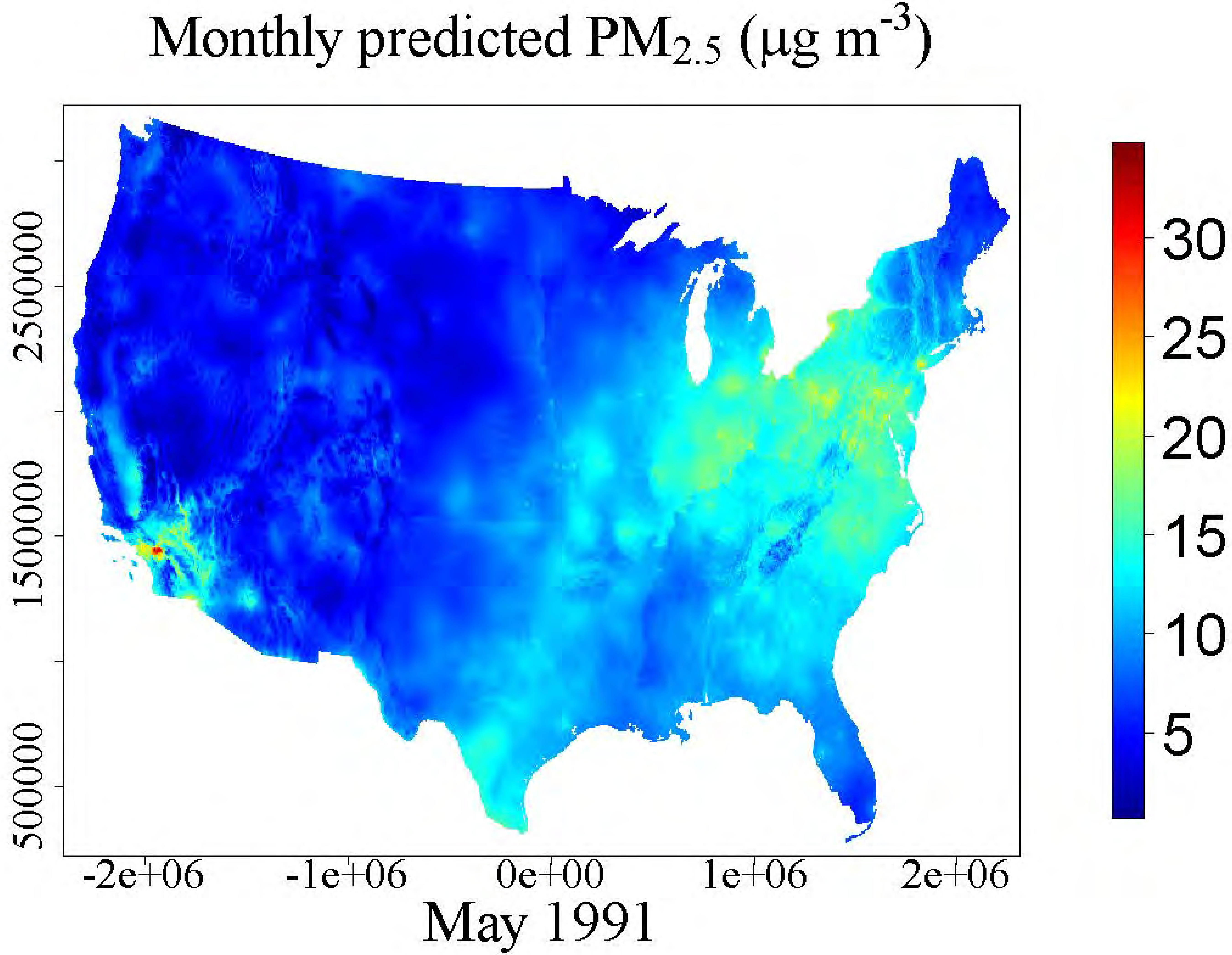

Monthly predicted PM2.5 (μg m-3)
25000000
15000000
5000000
-2e+06
-1e+06
0e+00
1e+06
2e+06
May 1991
30
25
20
15
10
5

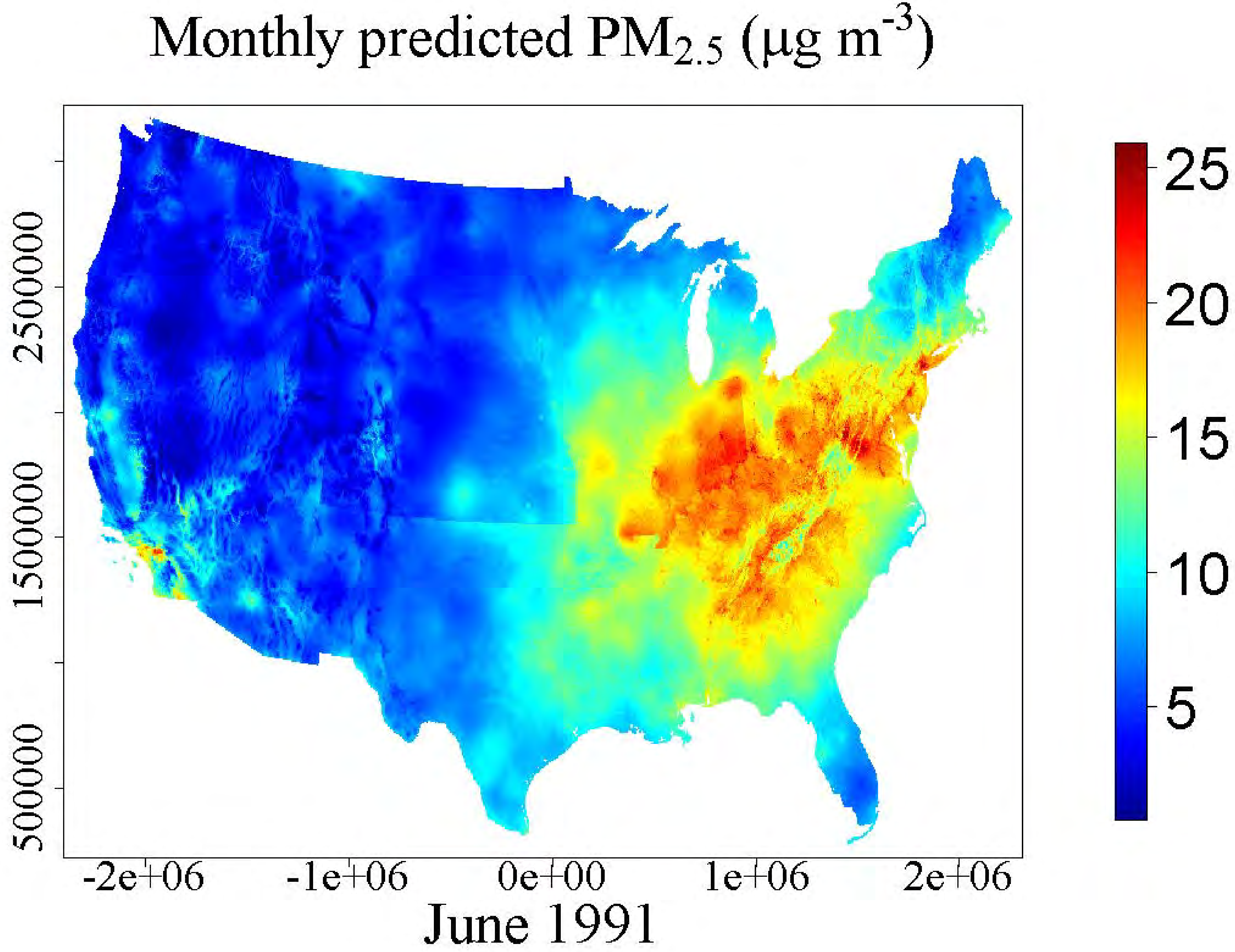

Monthly predicted $PM_{2.5}$ ($\mu g\ m^{-3}$)
25
20
15
10
5
25000000
15000000
5000000
-2e+06
-1e+06
0e+00
1e+06
2e+06
June 1991

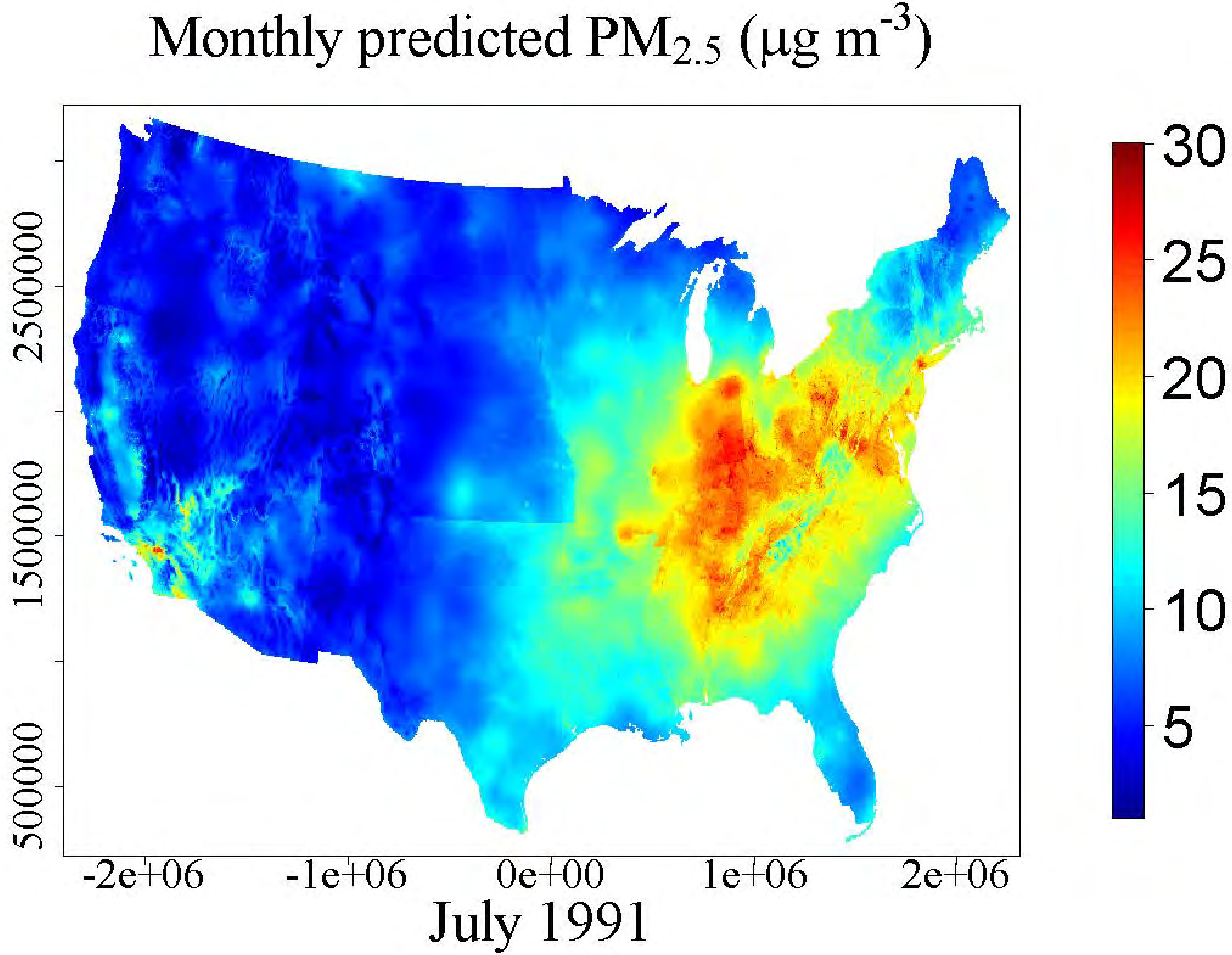
Monthly predicted PM2.5 (μg m-3)
30
25
20
15
10
5
25000000
15000000
5000000
-2e+06
-1e+06
0e+00
1e+06
2e+06
July 1991

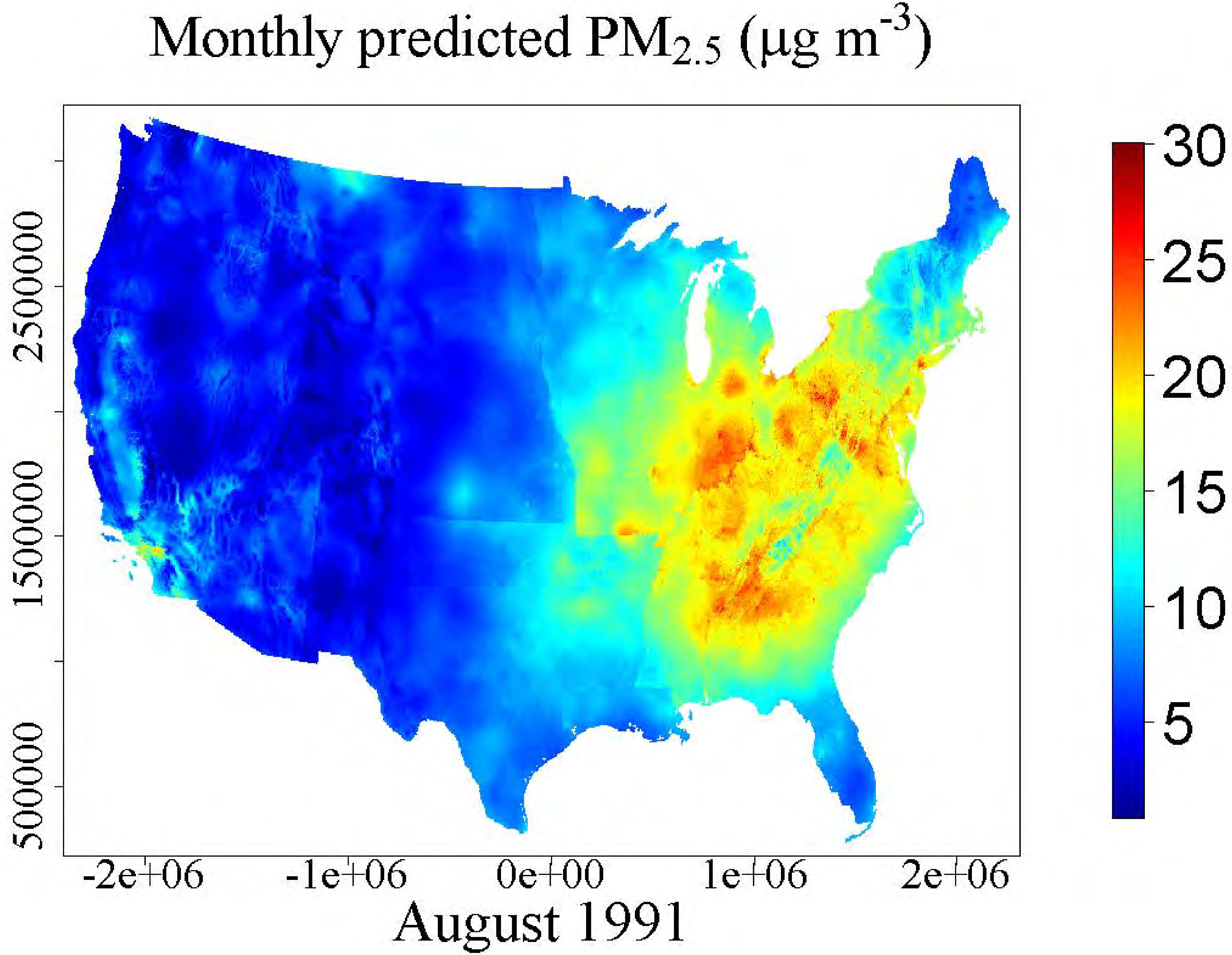
Monthly predicted $PM_{2.5}$ ($\mu g\ m^{-3}$)
25000000
15000000
5000000
-2e+06
-1e+06
0e+00
1e+06
2e+06
30
25
20
15
10
5
August 1991

Monthly predicted $PM_{2.5}$ ($\mu g\ m^{-3}$)

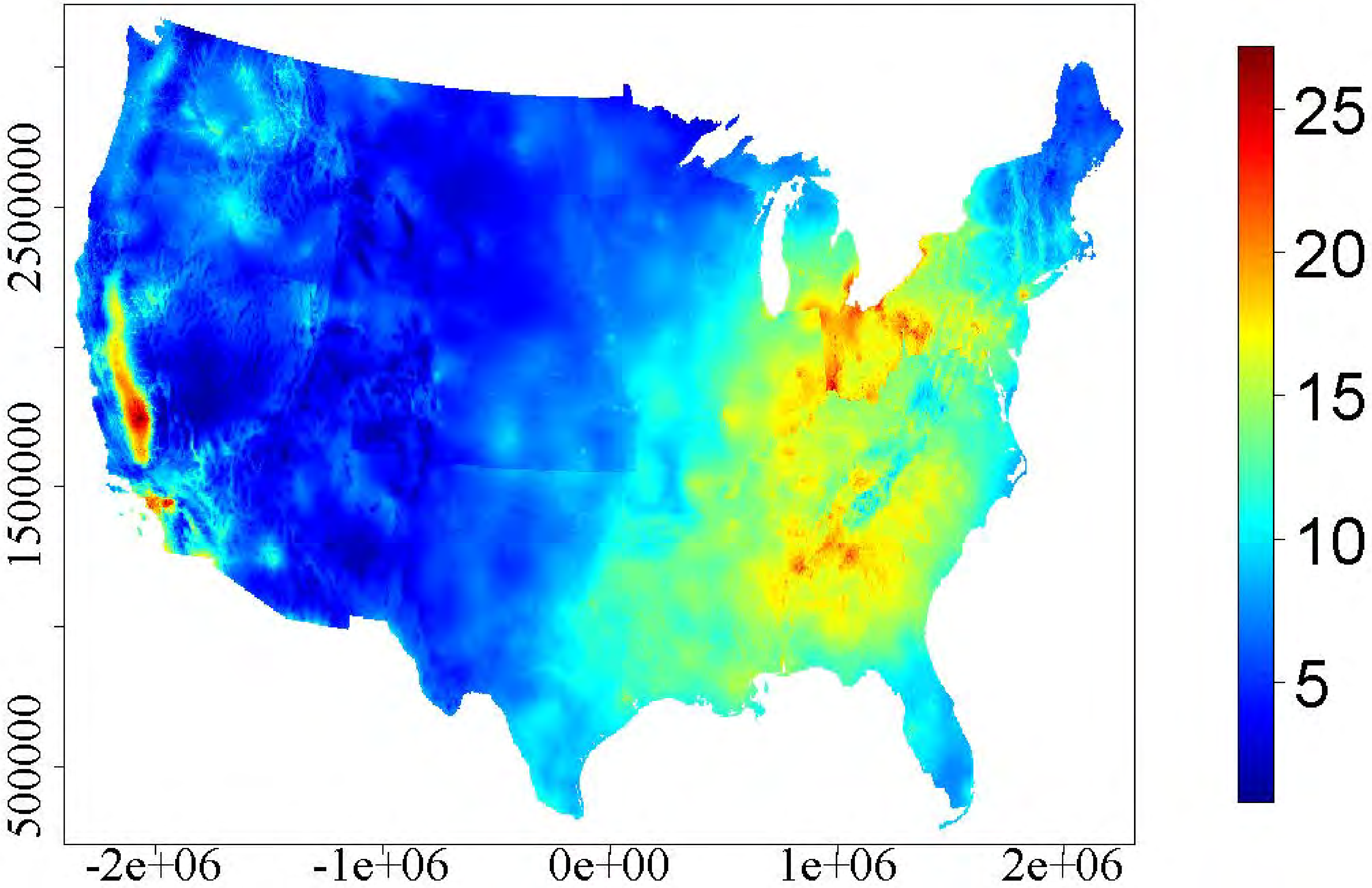

September 1991

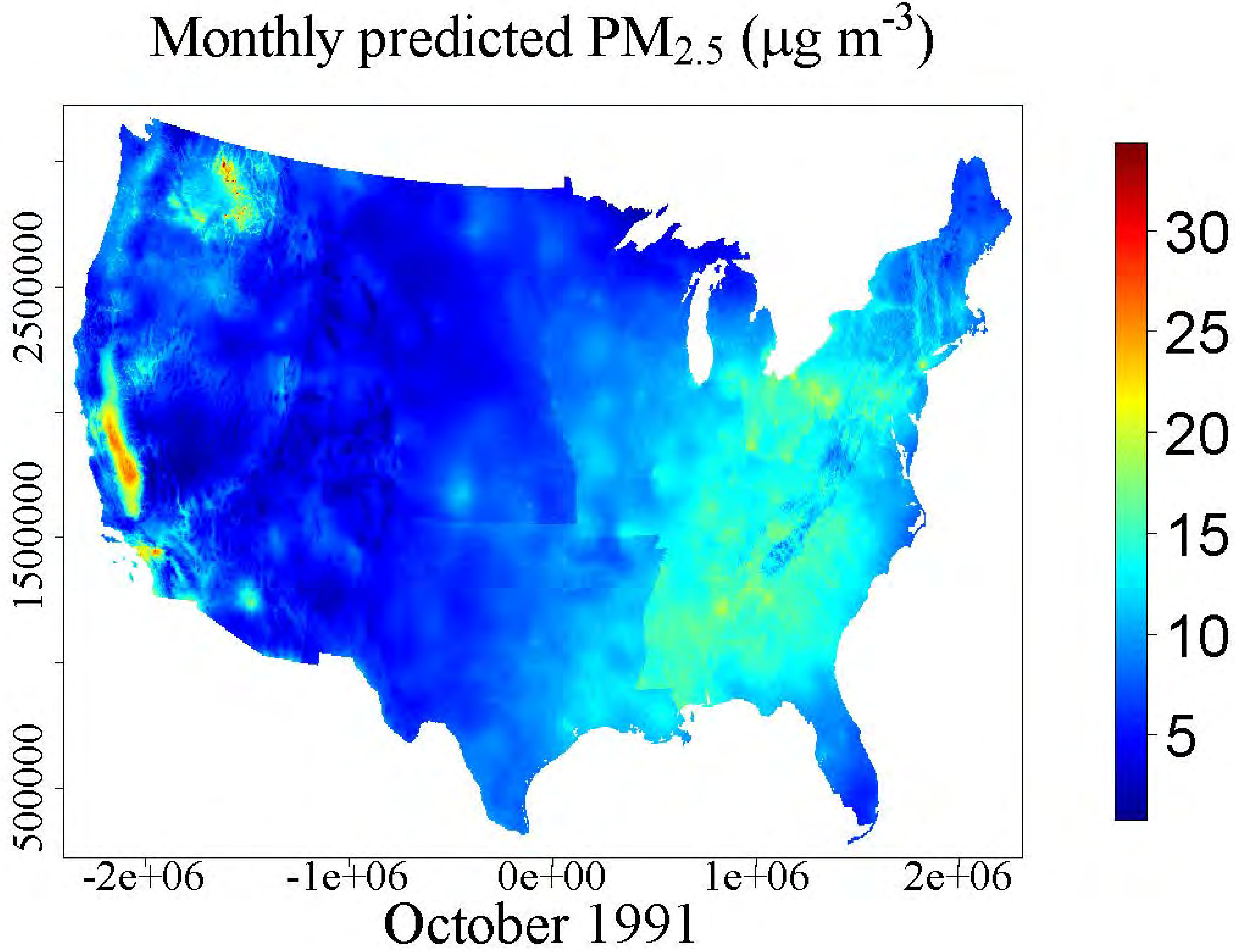

Monthly predicted PM2.5 (μg m-3)
25000000
15000000
5000000
-2e+06
-1e+06
0e+00
1e+06
2e+06
October 1991
30
25
20
15
10
5

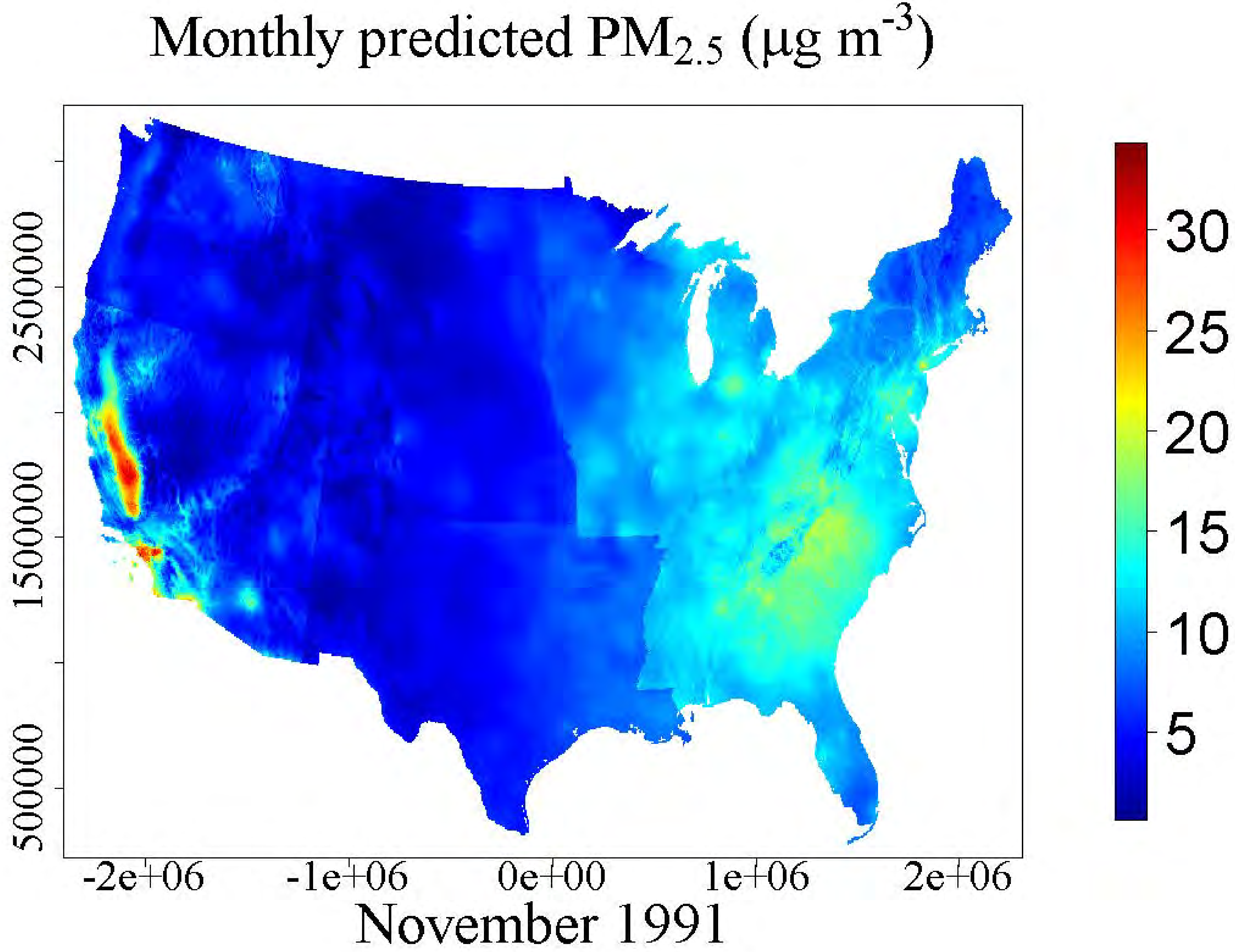
Monthly predicted PM2.5 (μg m-3)
25000000
15000000
5000000
-2e+06
-1e+06
0e+00
1e+06
2e+06
November 1991
30
25
20
15
10
5

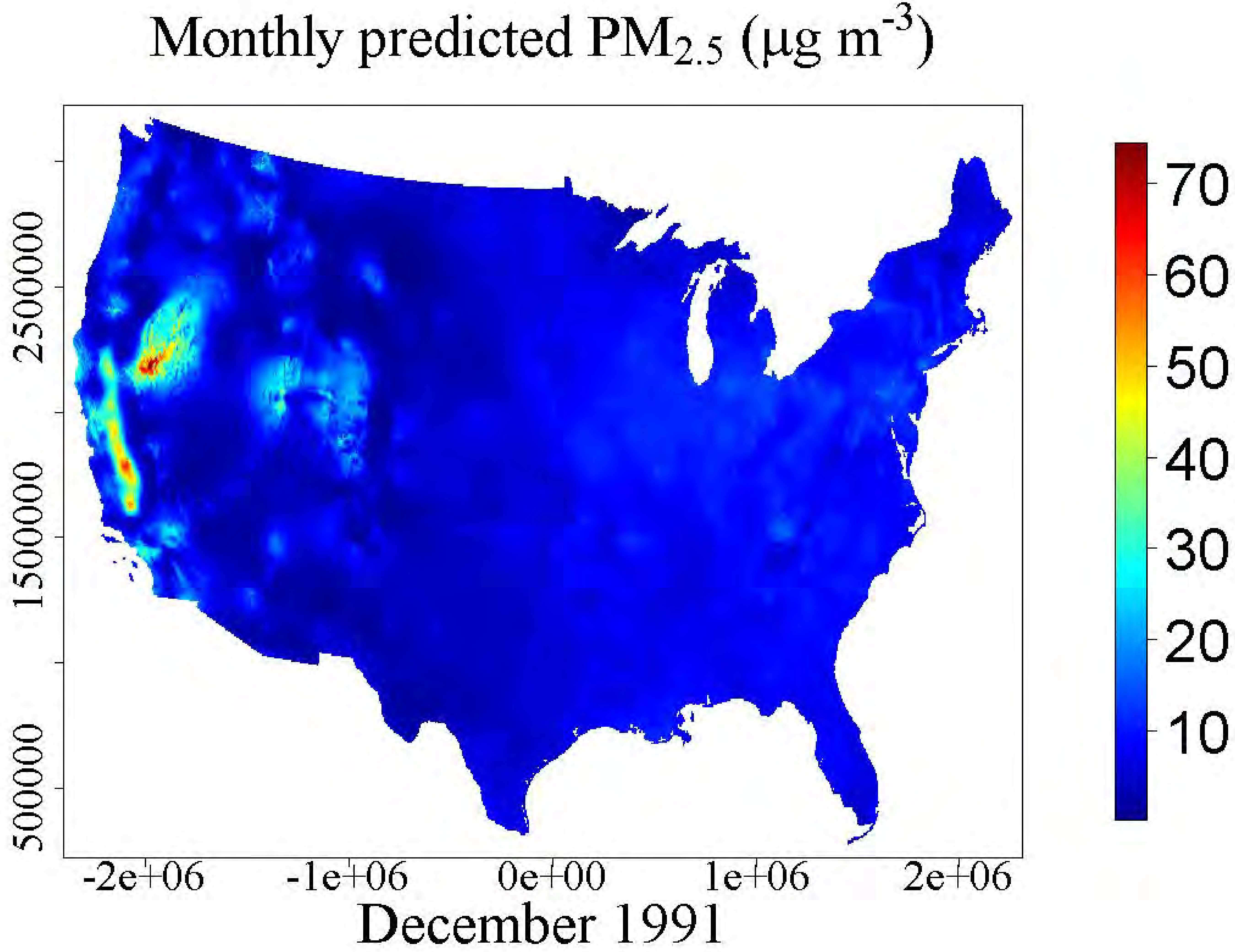
Monthly predicted PM2.5 (μg m-3)
25000000
15000000
5000000
-2e+06
-1e+06
0e+00
1e+06
2e+06
December 1991
70
60
50
40
30
20
10

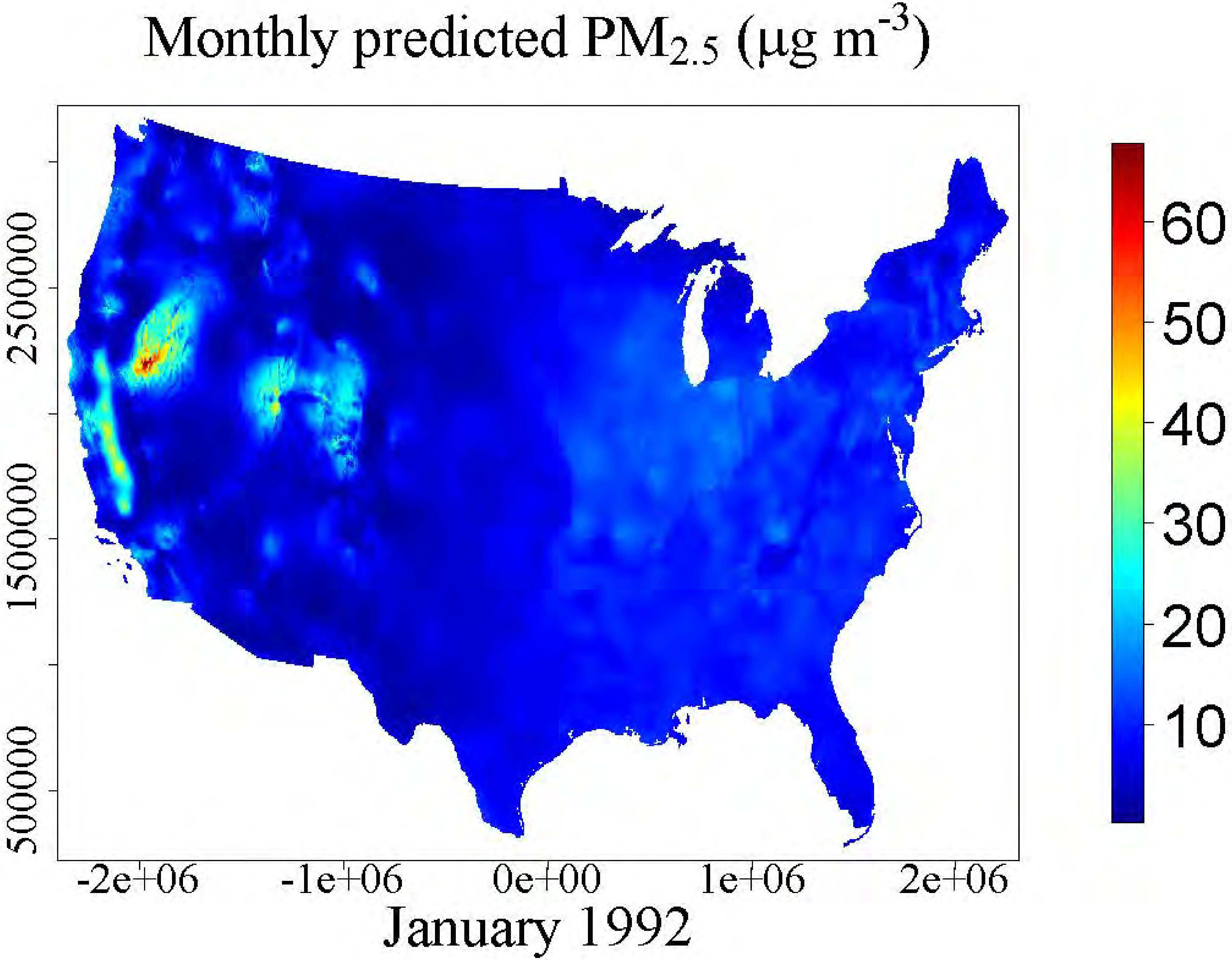
Monthly predicted $PM_{2.5}$ ($\mu g\ m^{-3}$)
25000000
15000000
5000000
-2e+06
-1e+06
0e+00
1e+06
2e+06
January 1992
60
50
40
30
20
10

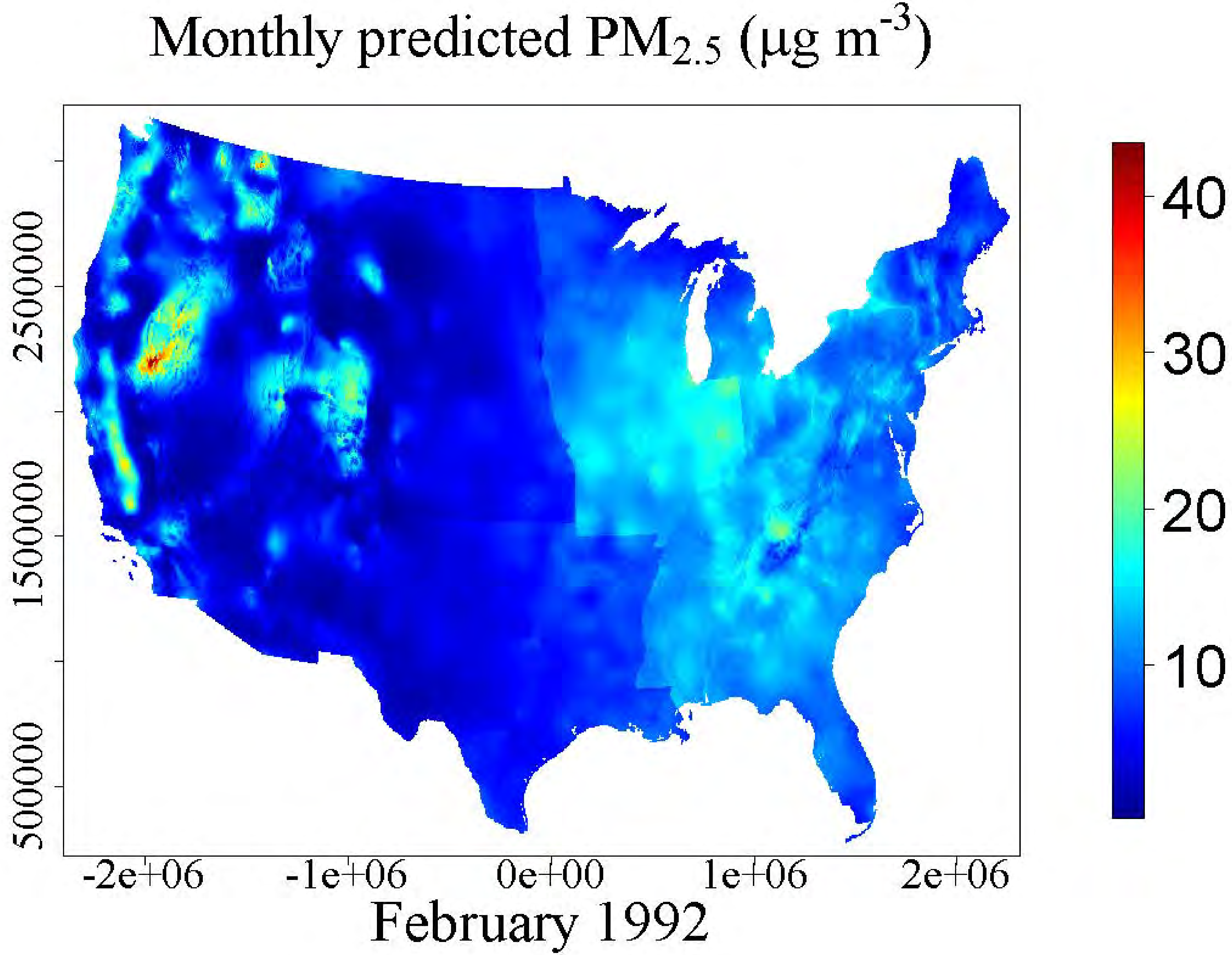

Monthly predicted $PM_{2.5}$ ($\mu g\ m^{-3}$)
25000000
15000000
5000000
-2e+06
-1e+06
0e+00
1e+06
2e+06
40
30
20
10
February 1992

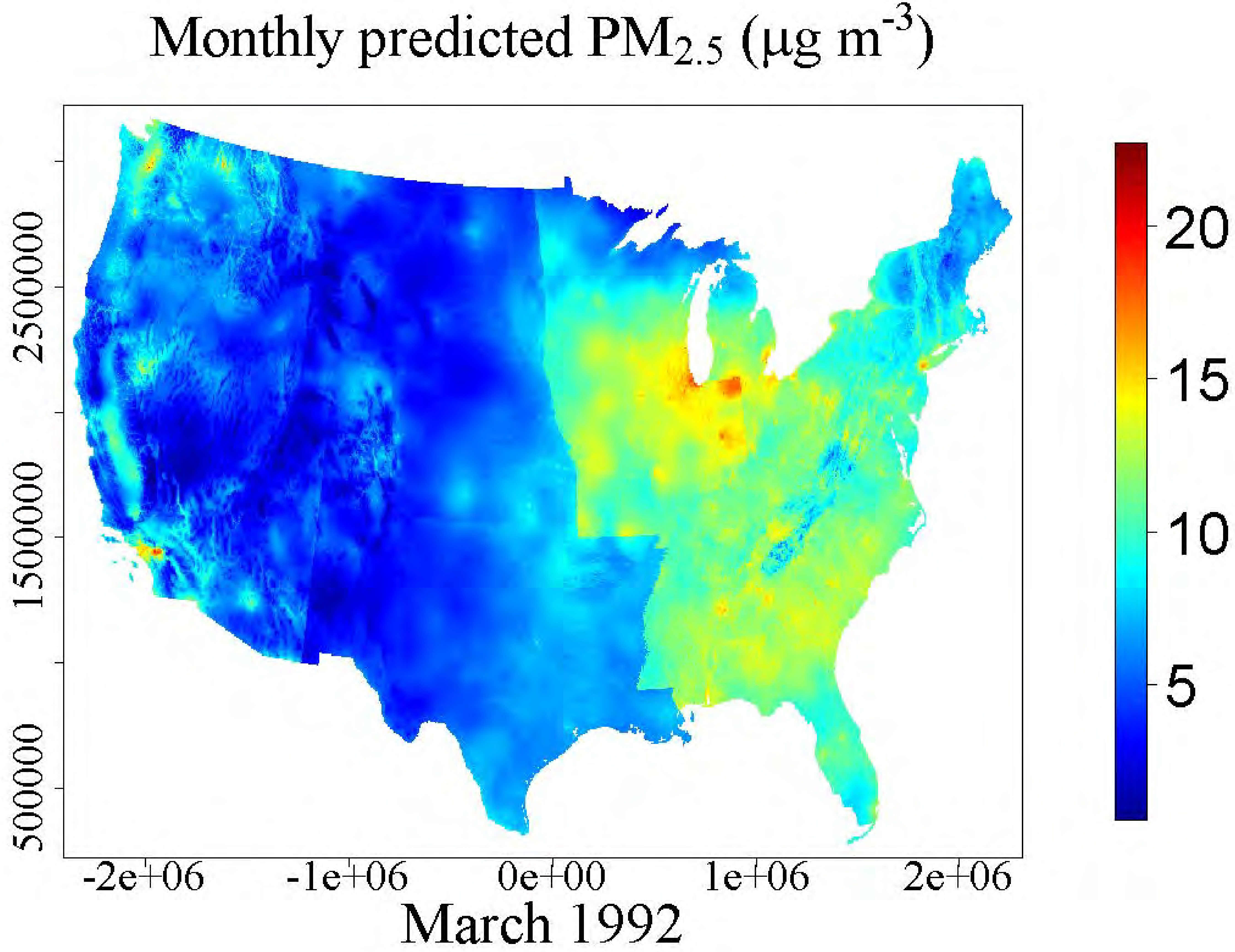
Monthly predicted PM2.5 (μg m-3)
25000000
15000000
5000000
-2e+06
-1e+06
0e+00
1e+06
2e+06
March 1992
20
15
10
5

Monthly predicted $PM_{2.5}$ ($\mu g\ m^{-3}$)

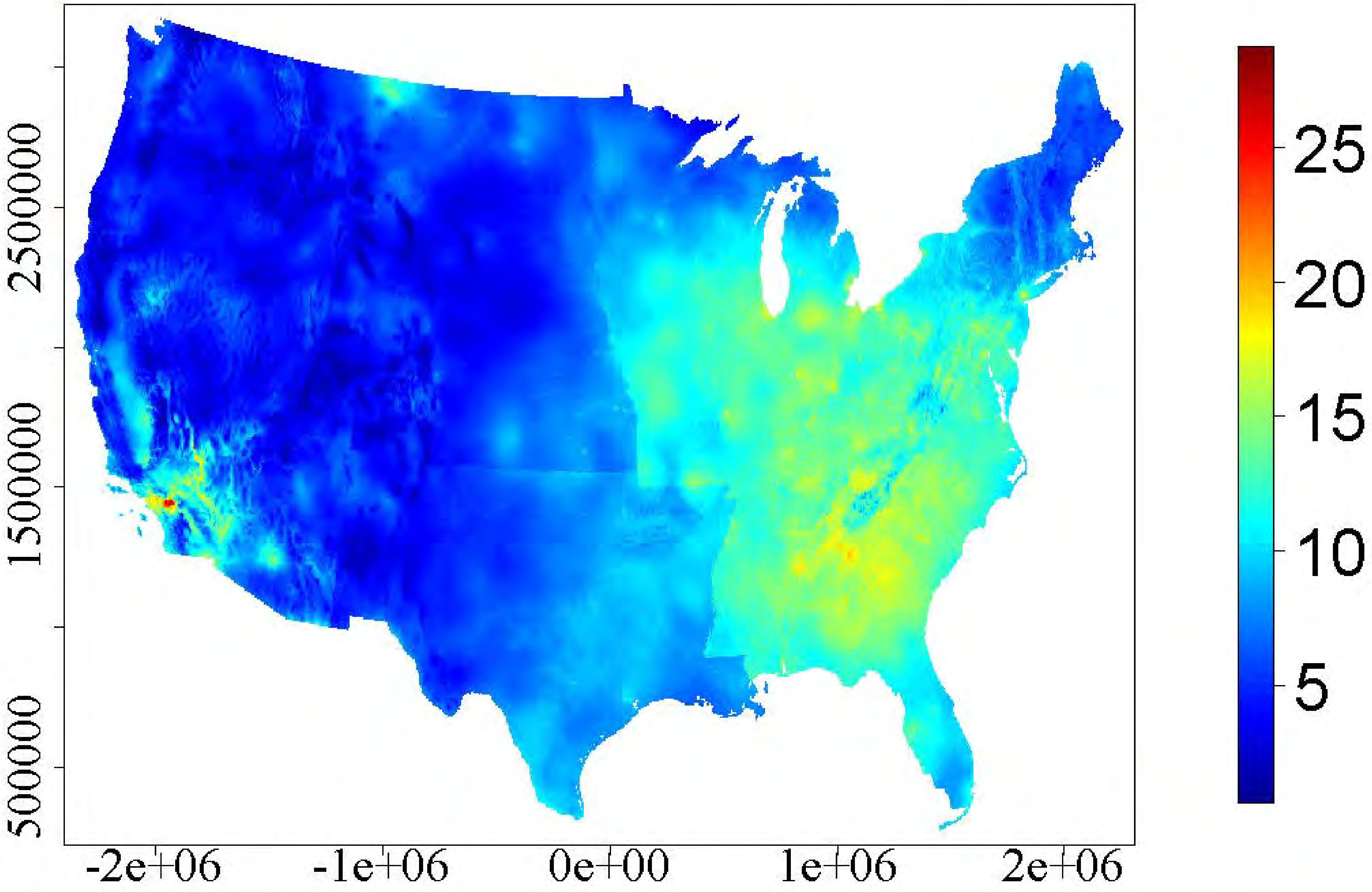

April 1992

Monthly predicted $PM_{2.5}$ ($\mu g\ m^{-3}$)

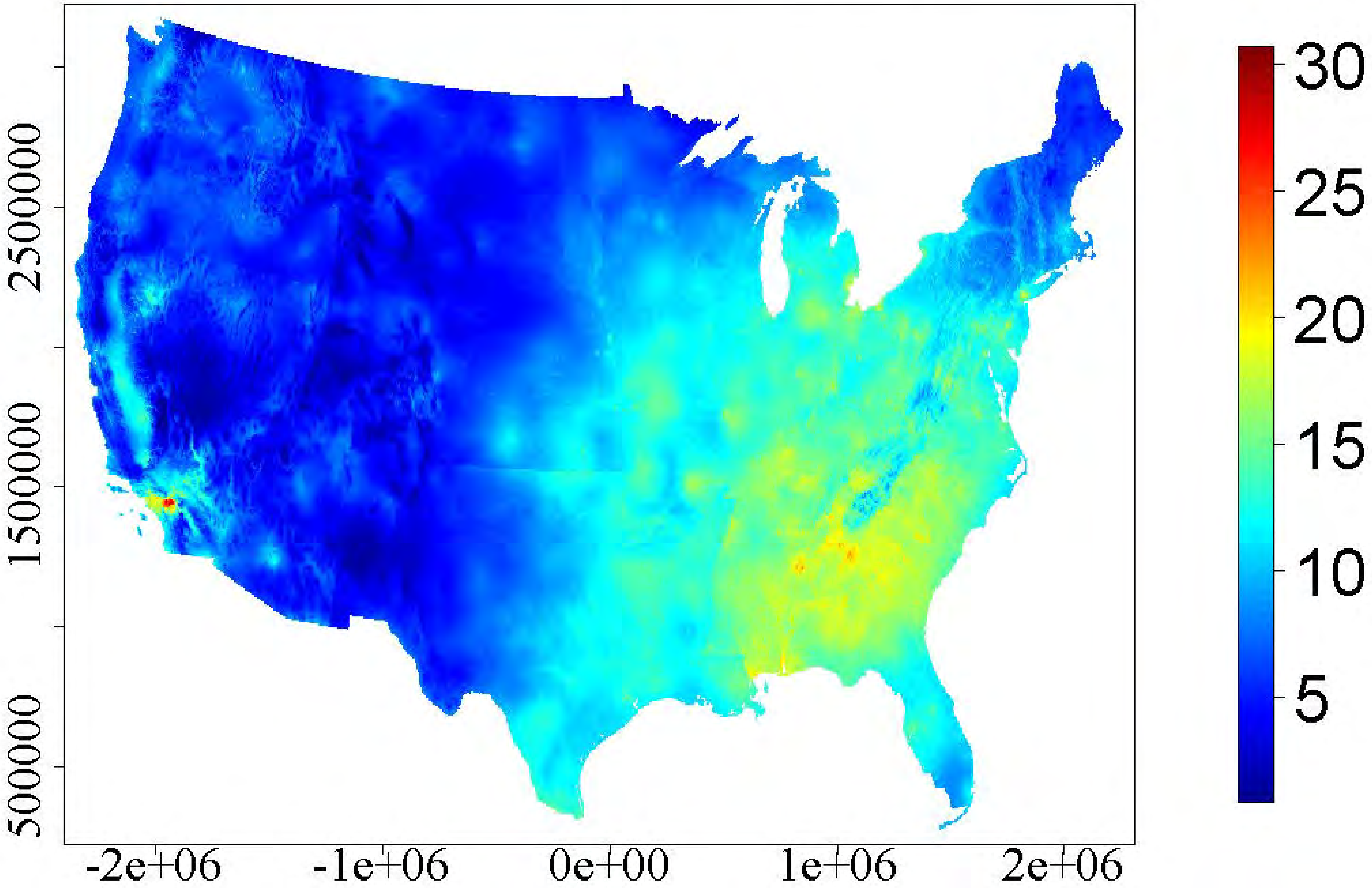

May 1992

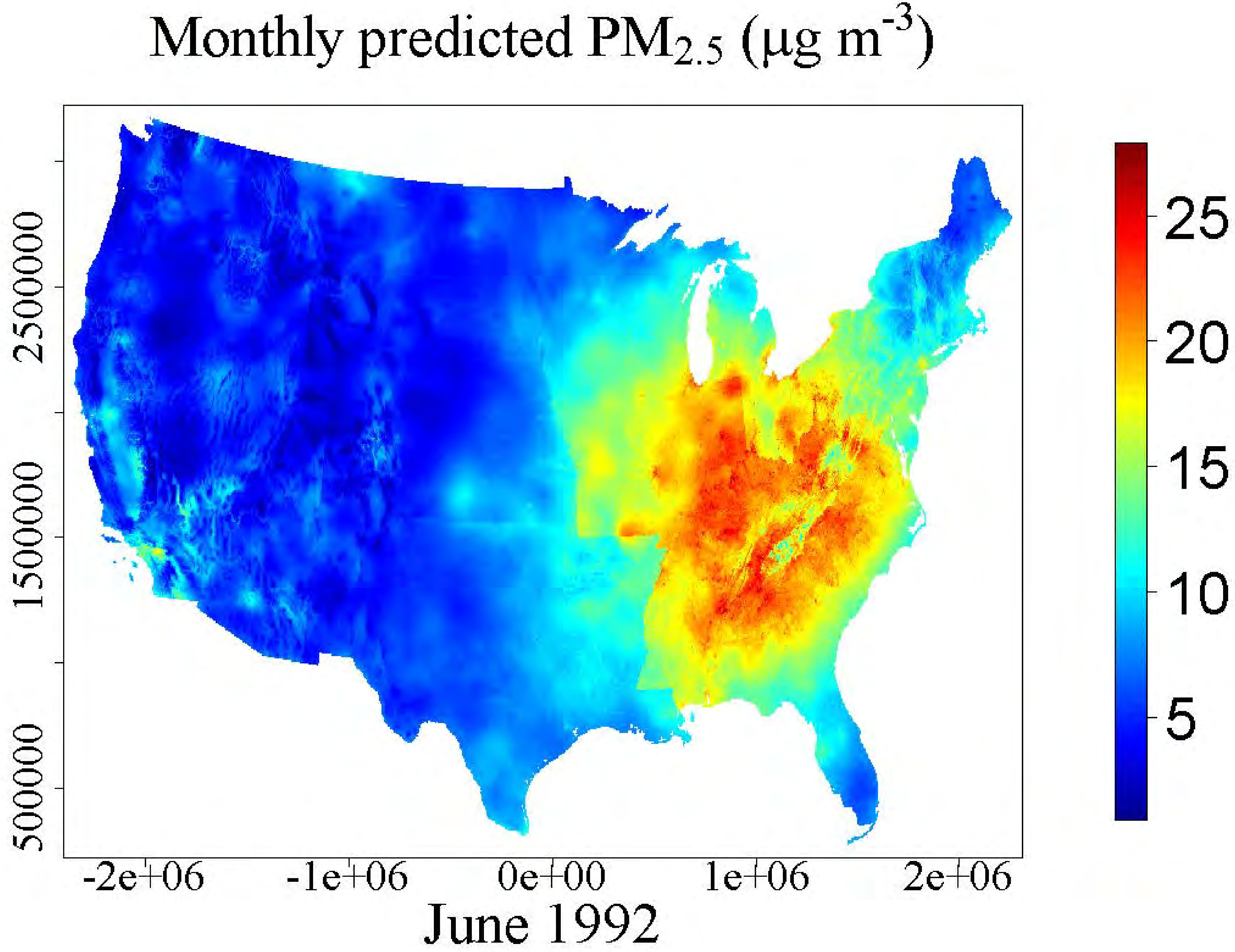

Monthly predicted PM2.5 (μg m-3)
25000000
15000000
5000000
-2e+06
-1e+06
0e+00
1e+06
2e+06
June 1992
25
20
15
10
5

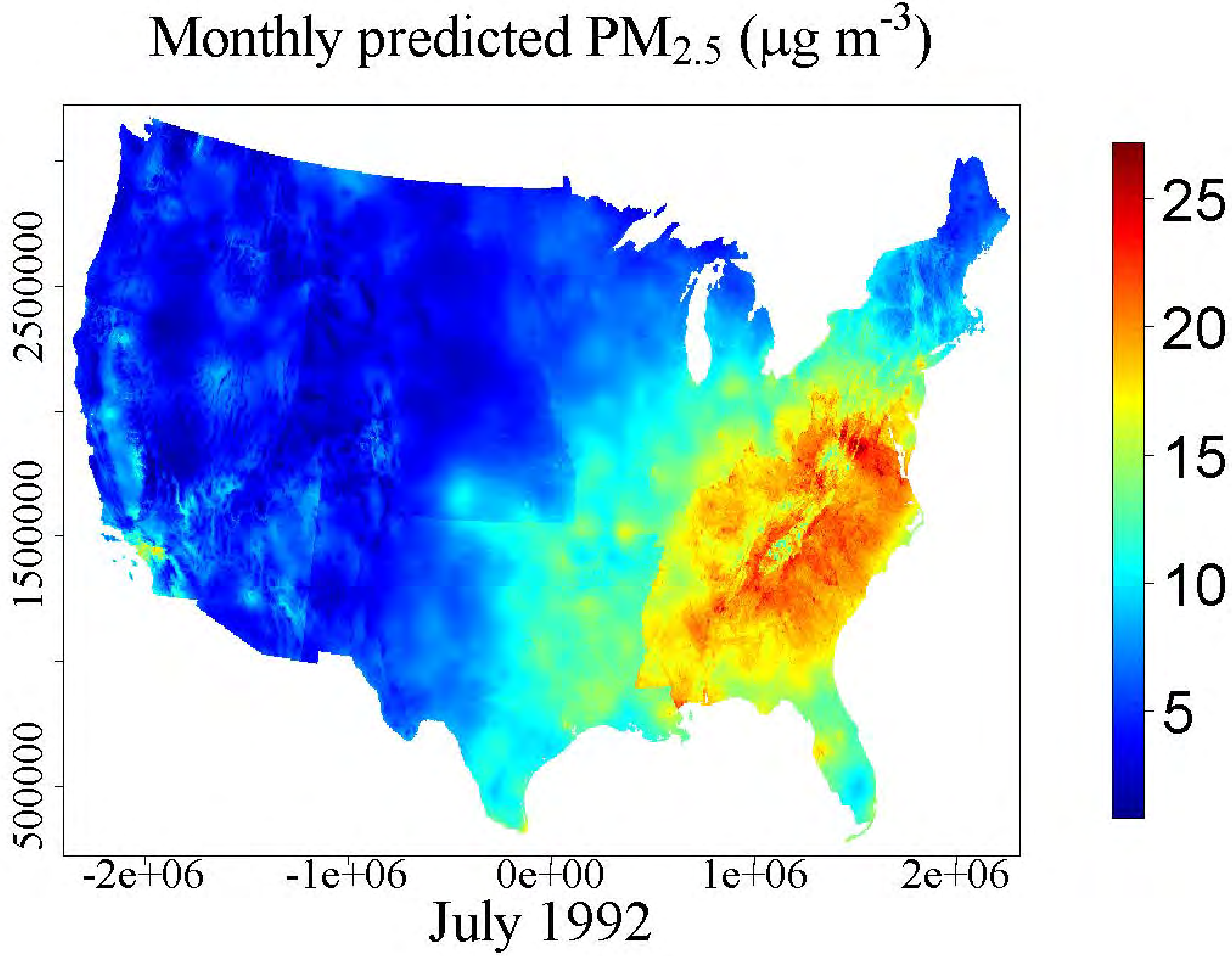
Monthly predicted PM2.5 (μg m-3)
25000000
15000000
5000000
-2e+06
-1e+06
0e+00
1e+06
2e+06
July 1992
25
20
15
10
5

Monthly predicted $PM_{2.5}$ ($\mu g\ m^{-3}$)

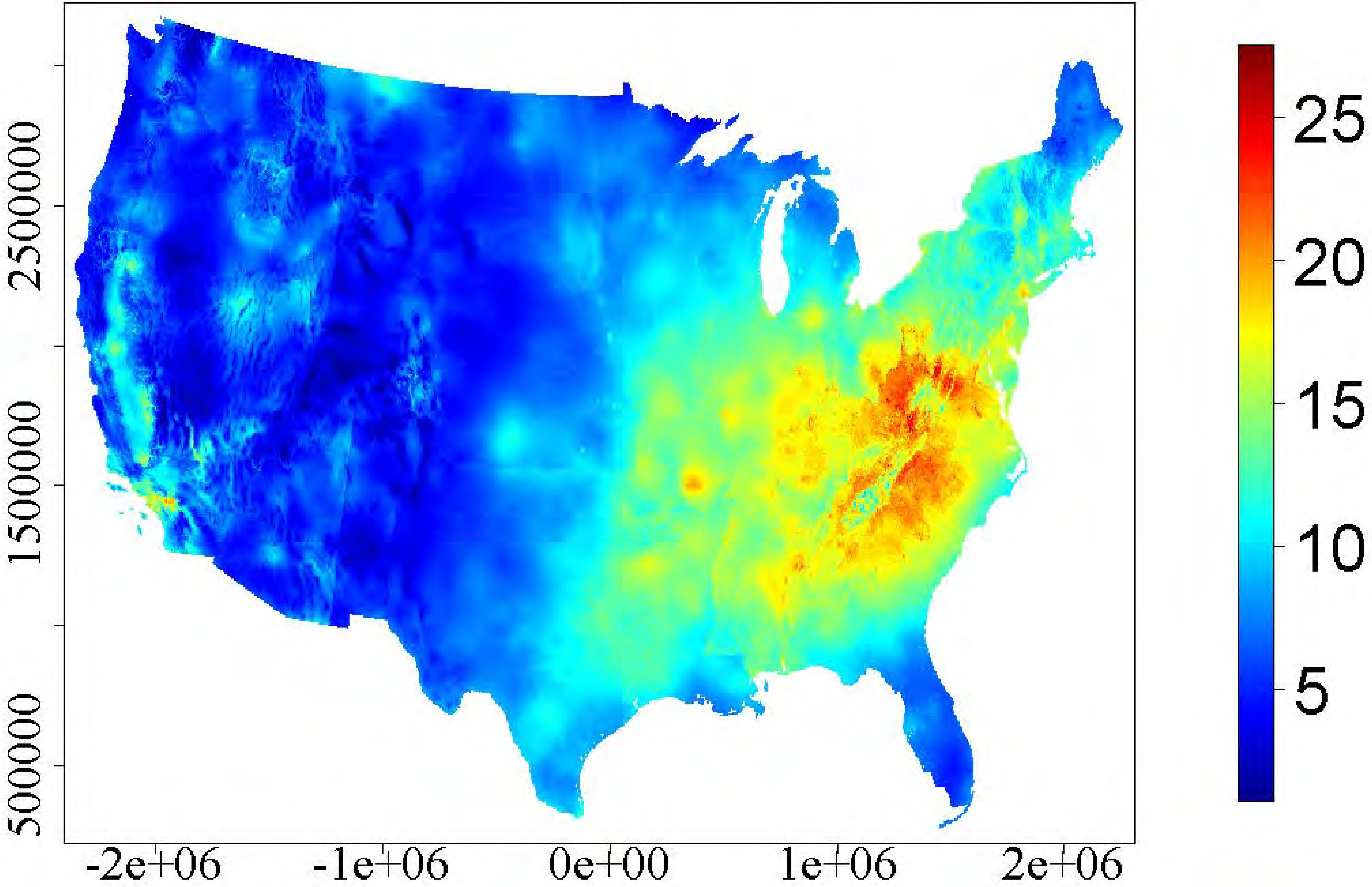

August 1992

Monthly predicted $PM_{2.5}$ ($\mu g\ m^{-3}$)

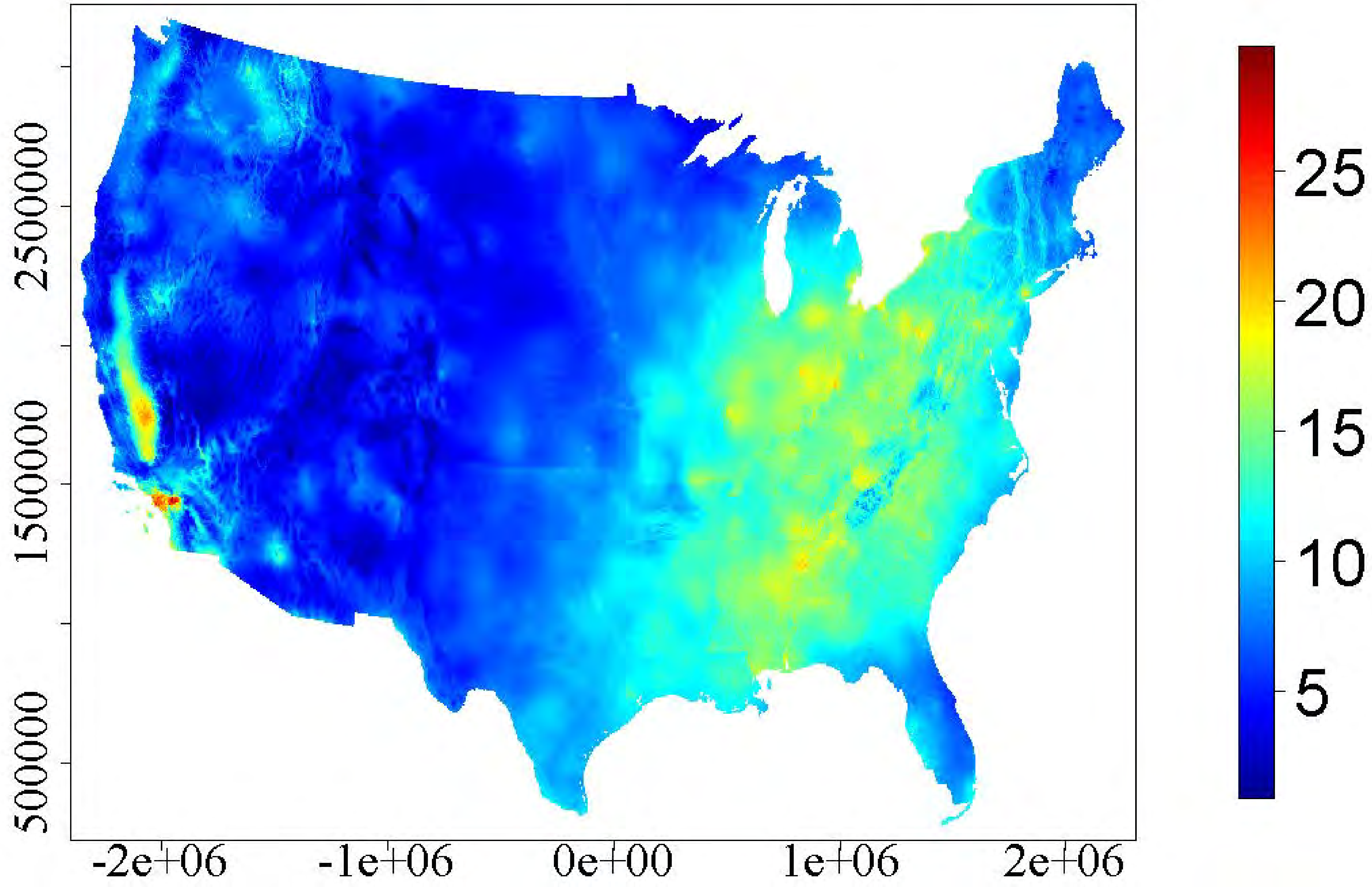

September 1992

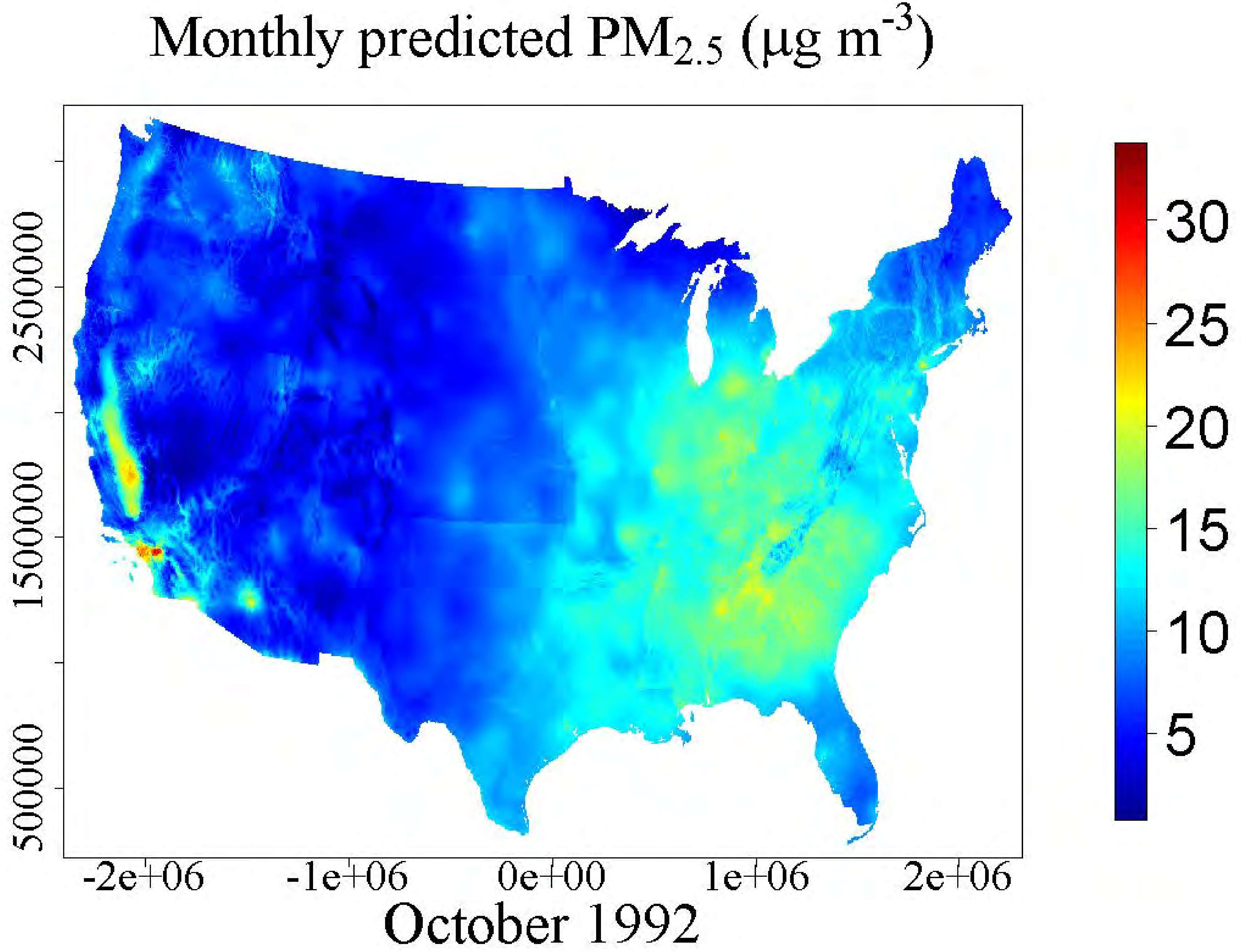
Monthly predicted PM2.5 (μg m-3)
25000000
15000000
5000000
-2e+06
-1e+06
0e+00
1e+06
2e+06
October 1992
30
25
20
15
10
5

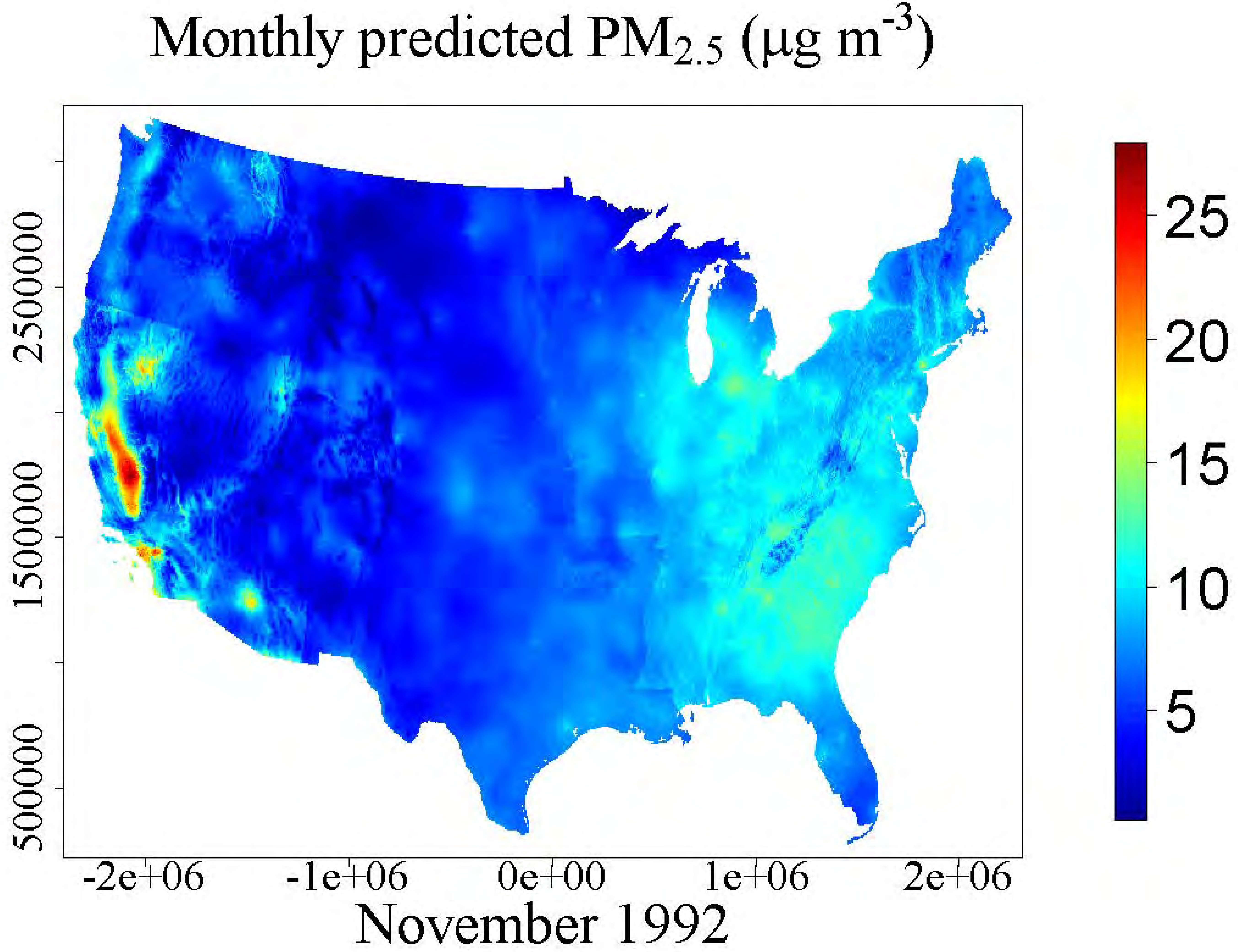
Monthly predicted PM2.5 (μg m-3)
25000000
15000000
5000000
-2e+06
-1e+06
0e+00
1e+06
2e+06
November 1992
25
20
15
10
5

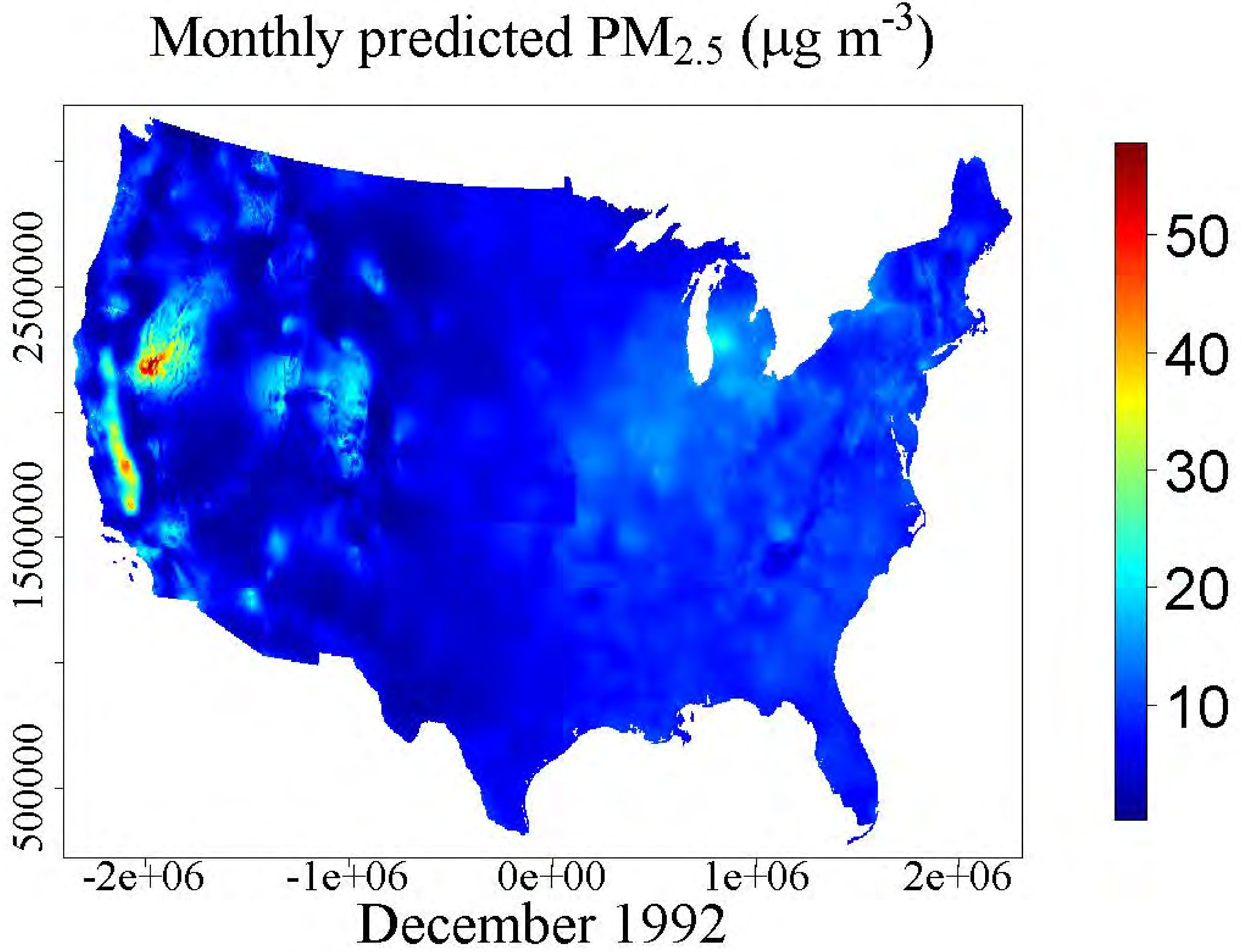

Monthly predicted PM2.5 (µg m-3)
25000000
15000000
5000000
-2e+06
-1e+06
0e+00
1e+06
2e+06
December 1992
50
40
30
20
10

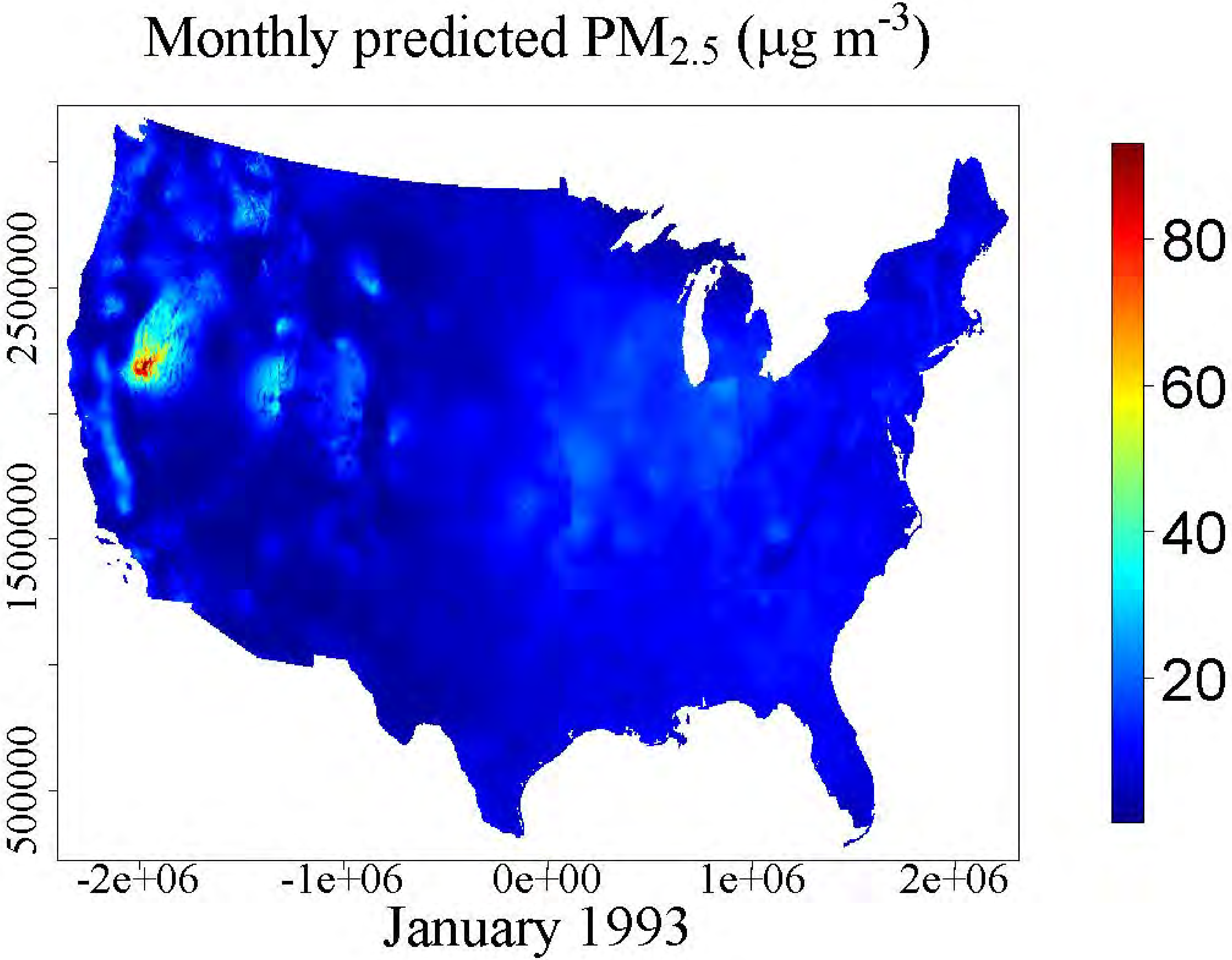
Monthly predicted PM2.5 (μg m-3)
25000000
15000000
5000000
-2e+06
-1e+06
0e+00
1e+06
2e+06
80
60
40
20
January 1993

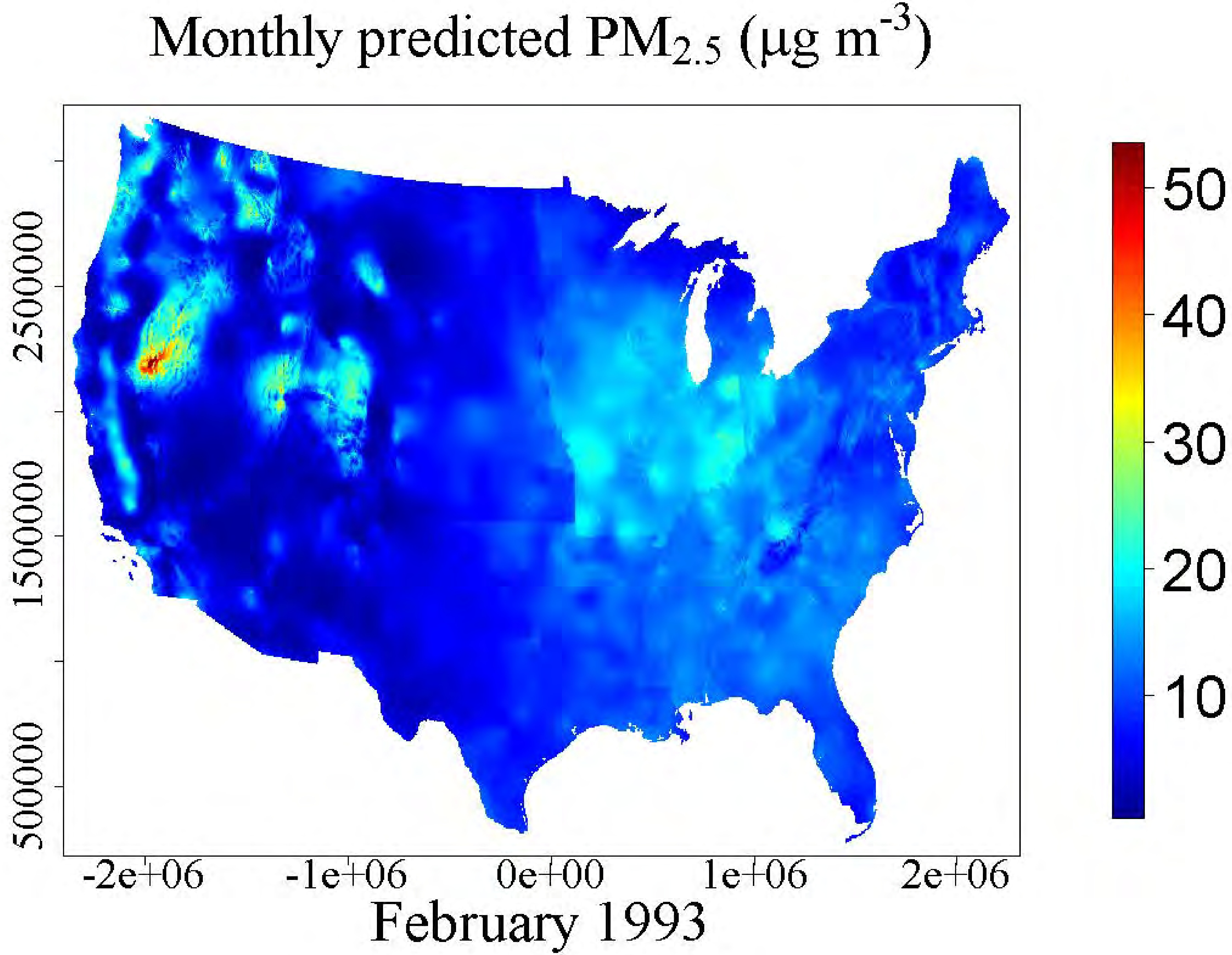
Monthly predicted $PM_{2.5}$ ($\mu g\ m^{-3}$)
25000000
15000000
5000000
-2e+06
-1e+06
0e+00
1e+06
2e+06
February 1993
50
40
30
20
10

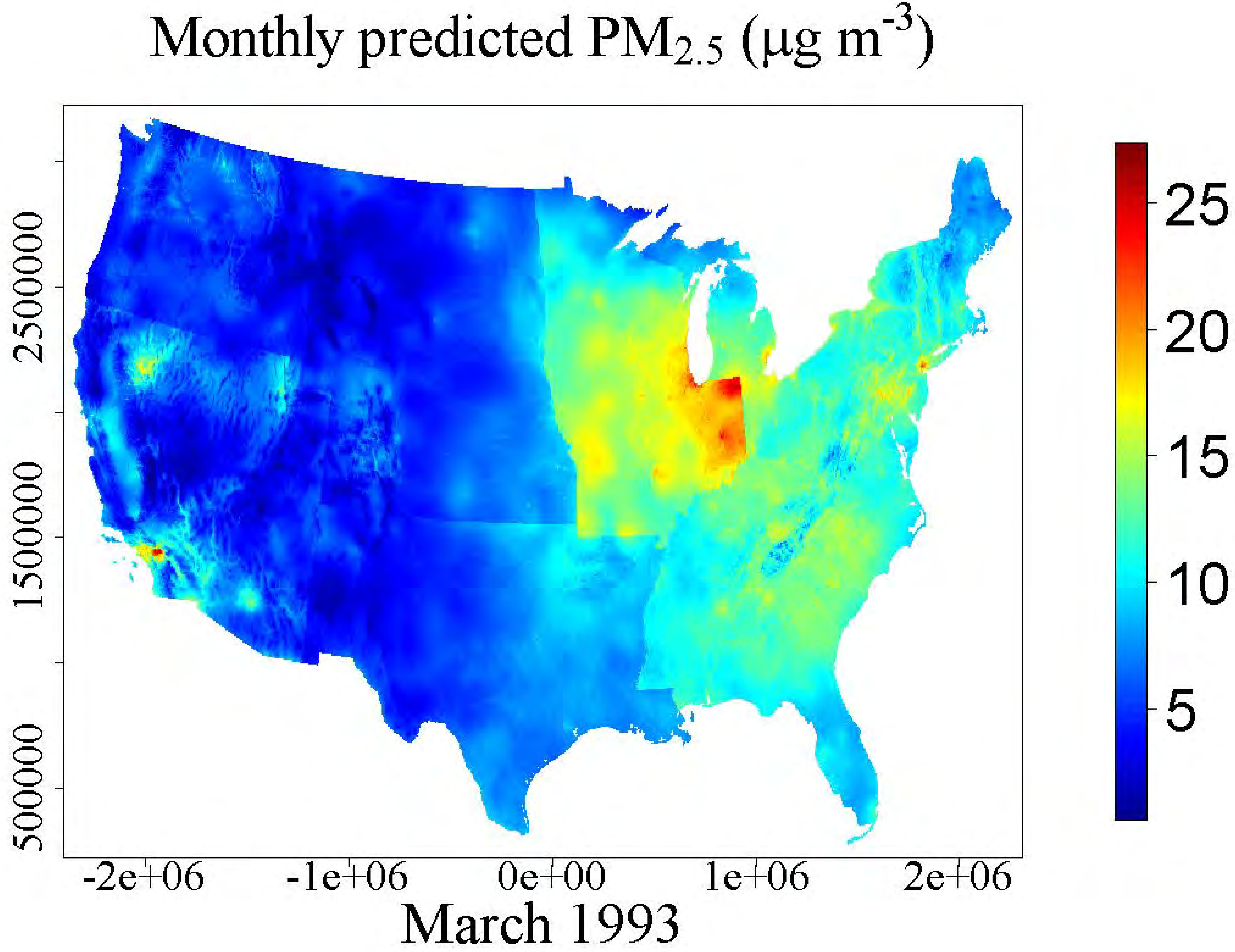

Monthly predicted PM2.5 (μg m-3)
25000000
15000000
5000000
-2e+06
-1e+06
0e+00
1e+06
2e+06
March 1993
25
20
15
10
5

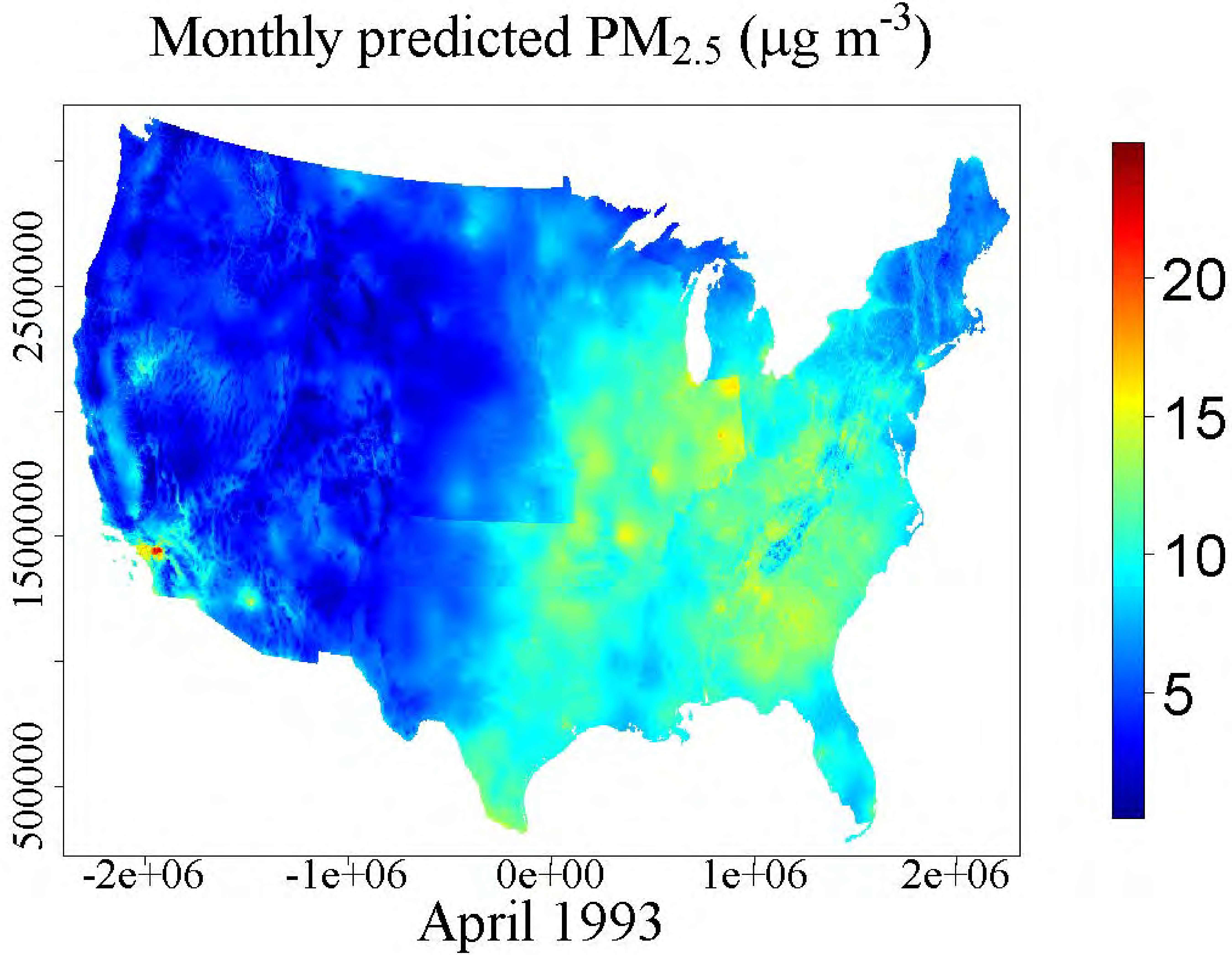

Monthly predicted PM2.5 (μg m-3)
25000000
15000000
5000000
-2e+06
-1e+06
0e+00
1e+06
2e+06
April 1993
20
15
10
5

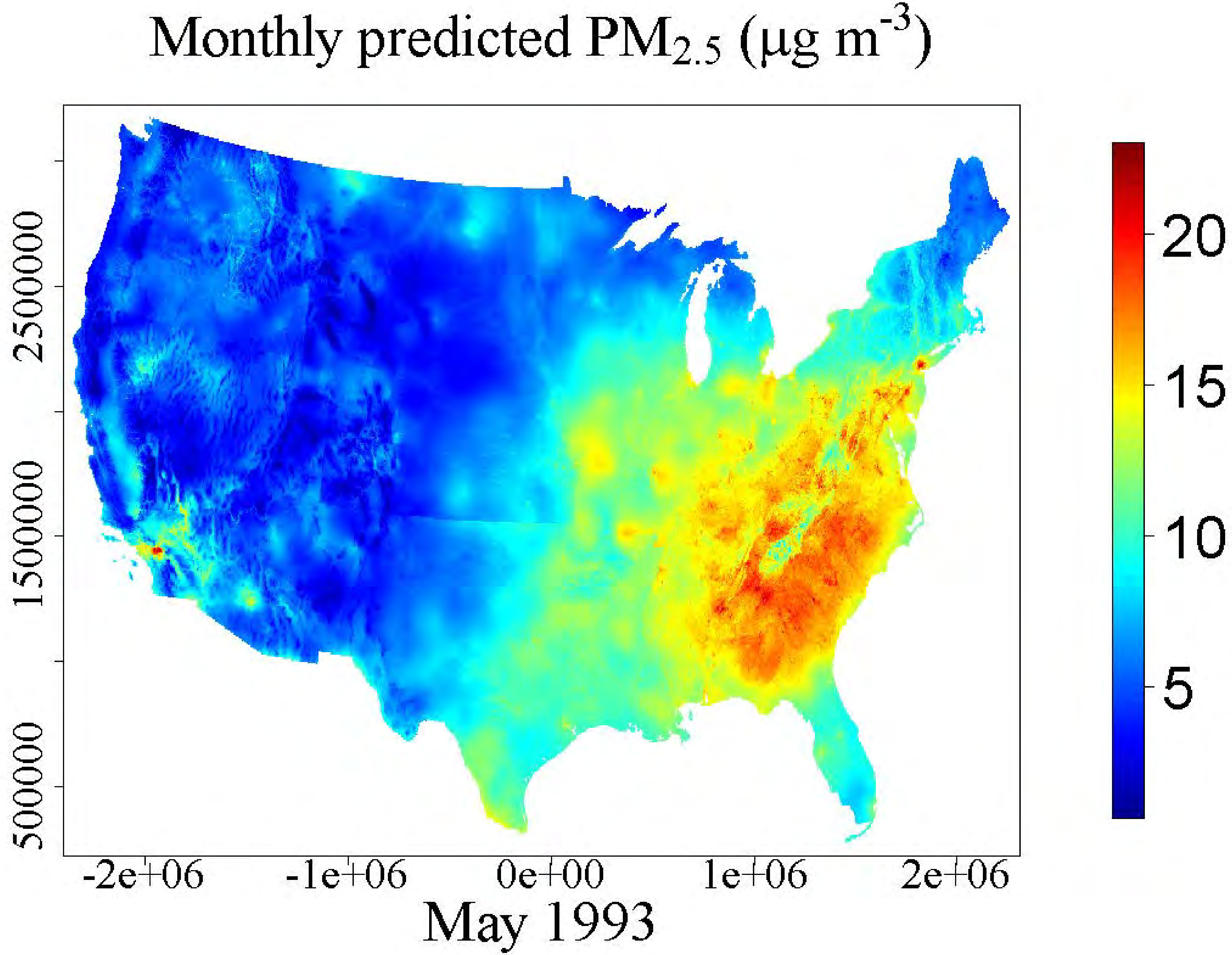

Monthly predicted PM$_{2.5}$ (μg m^{-3})
25000000
15000000
5000000
-2e+06
-1e+06
0e+00
1e+06
2e+06
May 1993
20
15
10
5

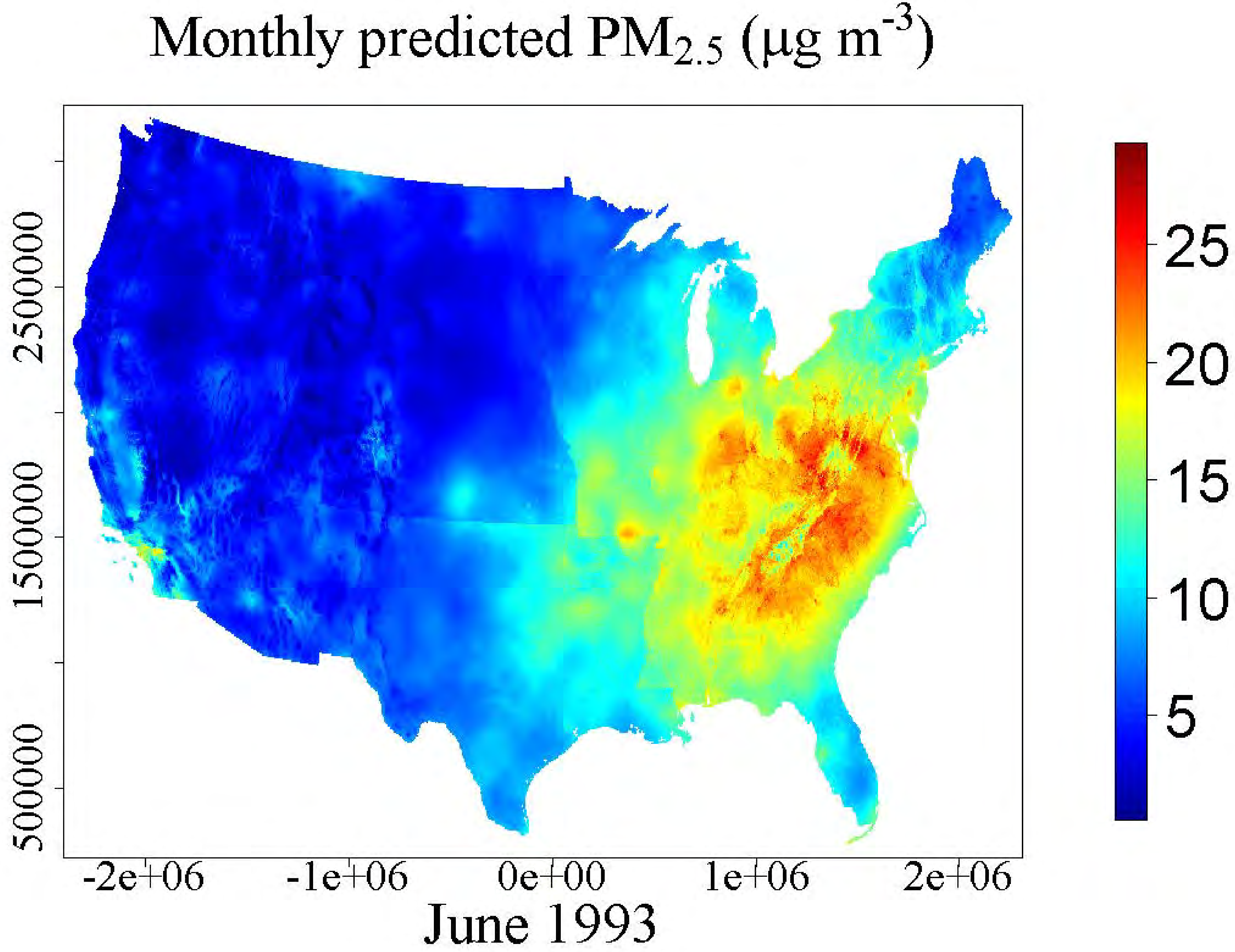

Monthly predicted PM2.5 (μg m-3)
25000000
15000000
5000000
-2e+06
-1e+06
0e+00
1e+06
2e+06
June 1993
25
20
15
10
5

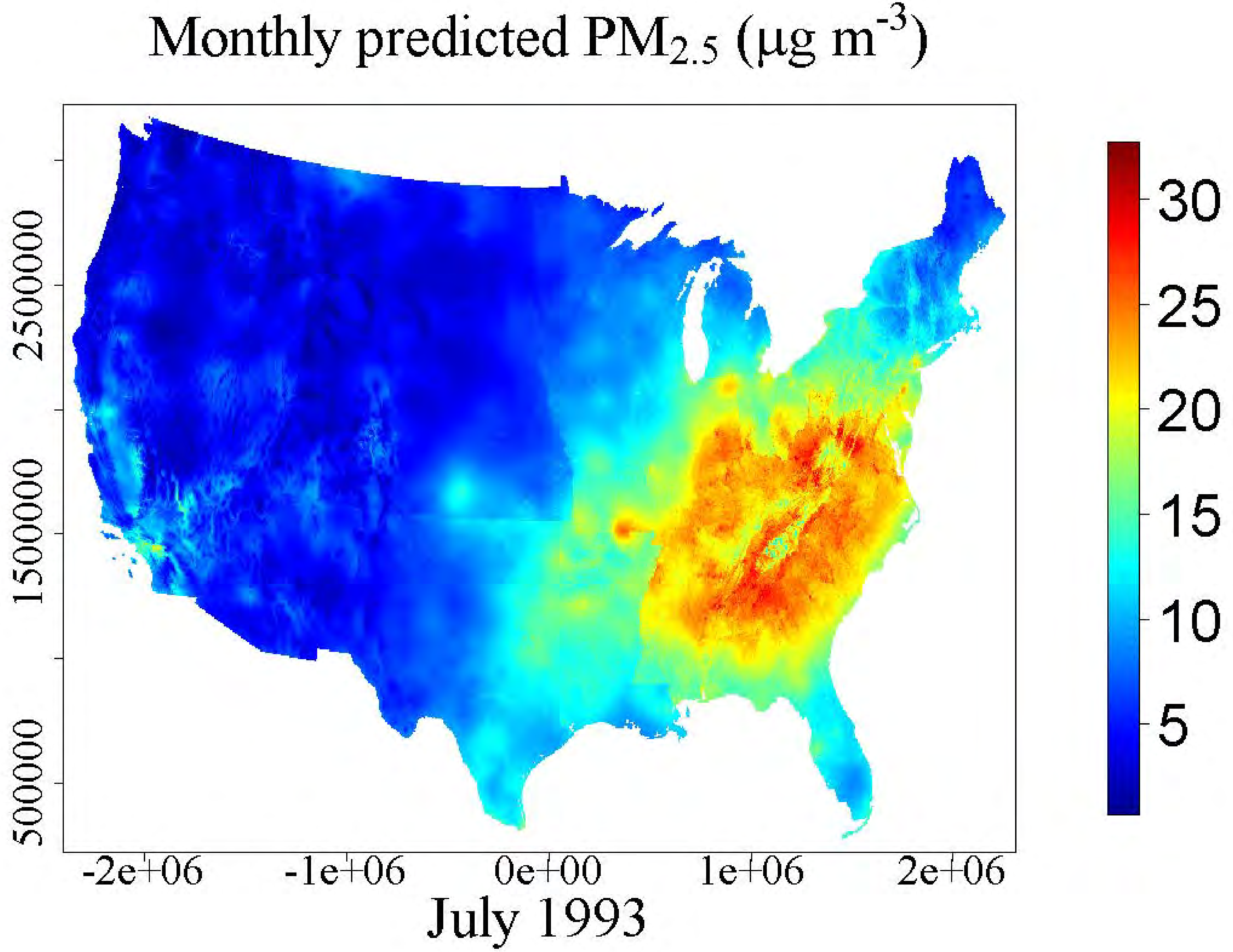

Monthly predicted PM2.5 (μg m-3)
25000000
15000000
5000000
-2e+06
-1e+06
0e+00
1e+06
2e+06
July 1993
30
25
20
15
10
5

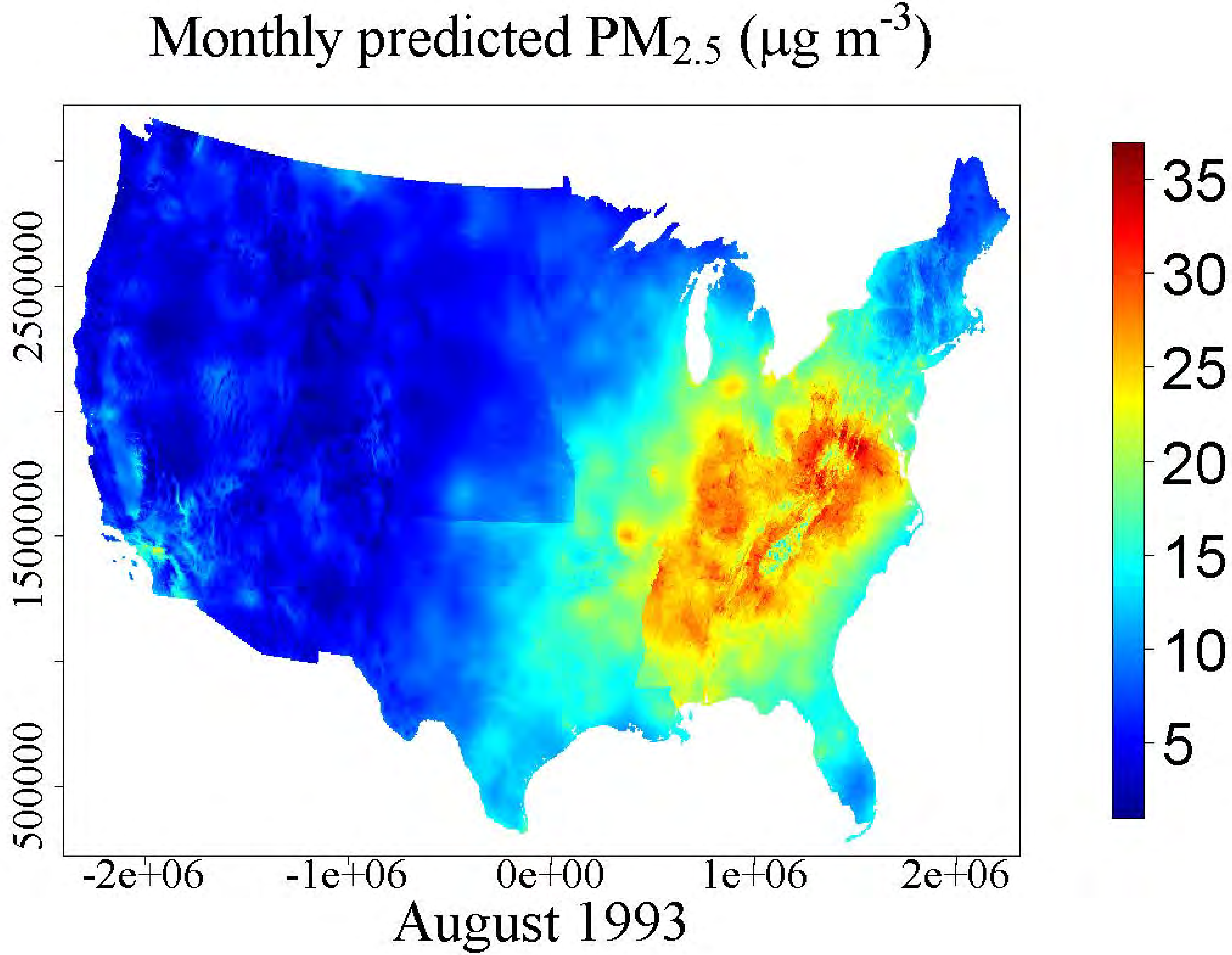
Monthly predicted $PM_{2.5}$ ($\mu g\ m^{-3}$)
25000000
15000000
5000000
-2e+06
-1e+06
0e+00
1e+06
2e+06
35
30
25
20
15
10
5
August 1993

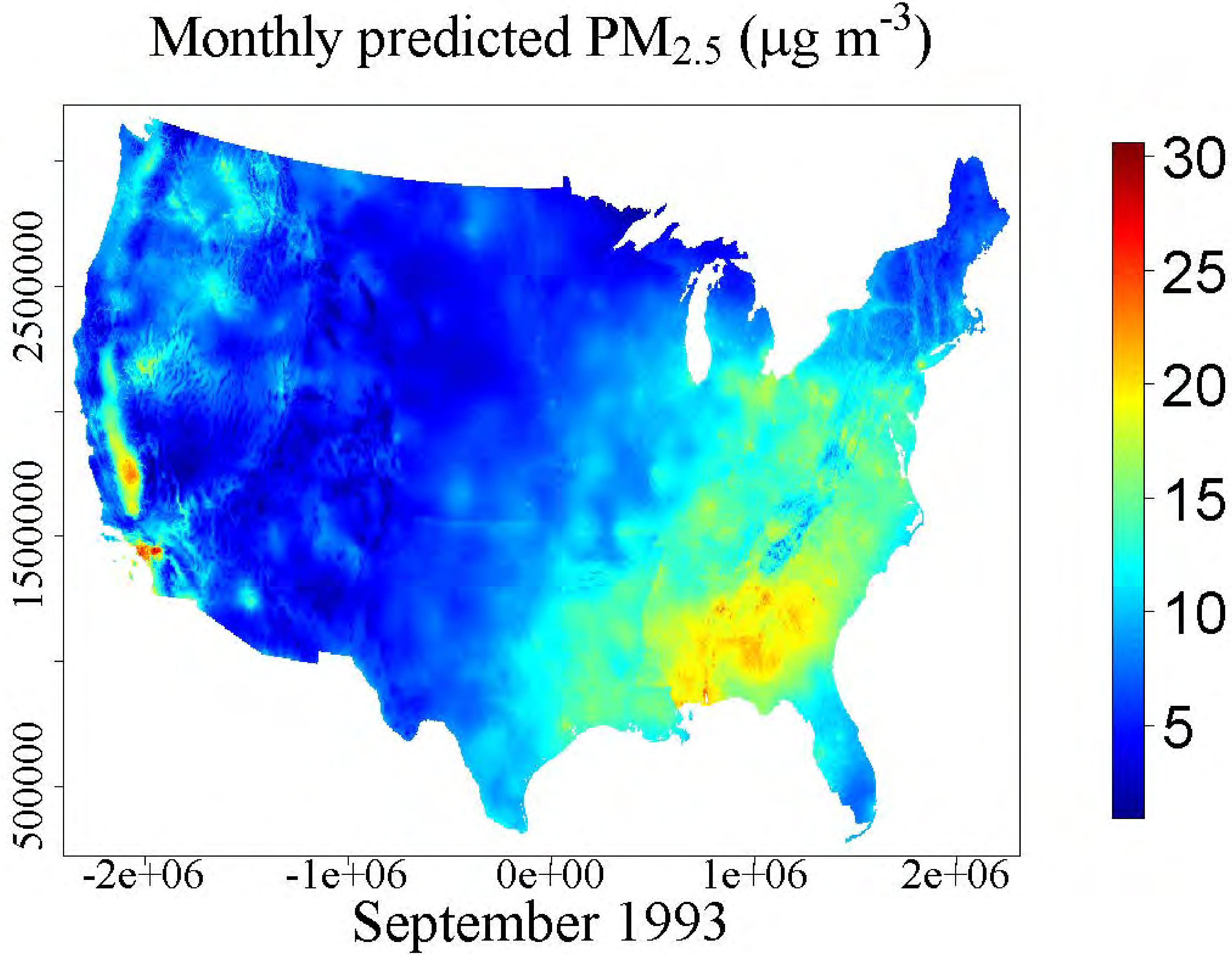
Monthly predicted PM2.5 (μg m-3)
30
25
20
15
10
5
25000000
15000000
5000000
-2e+06
-1e+06
0e+00
1e+06
2e+06
September 1993

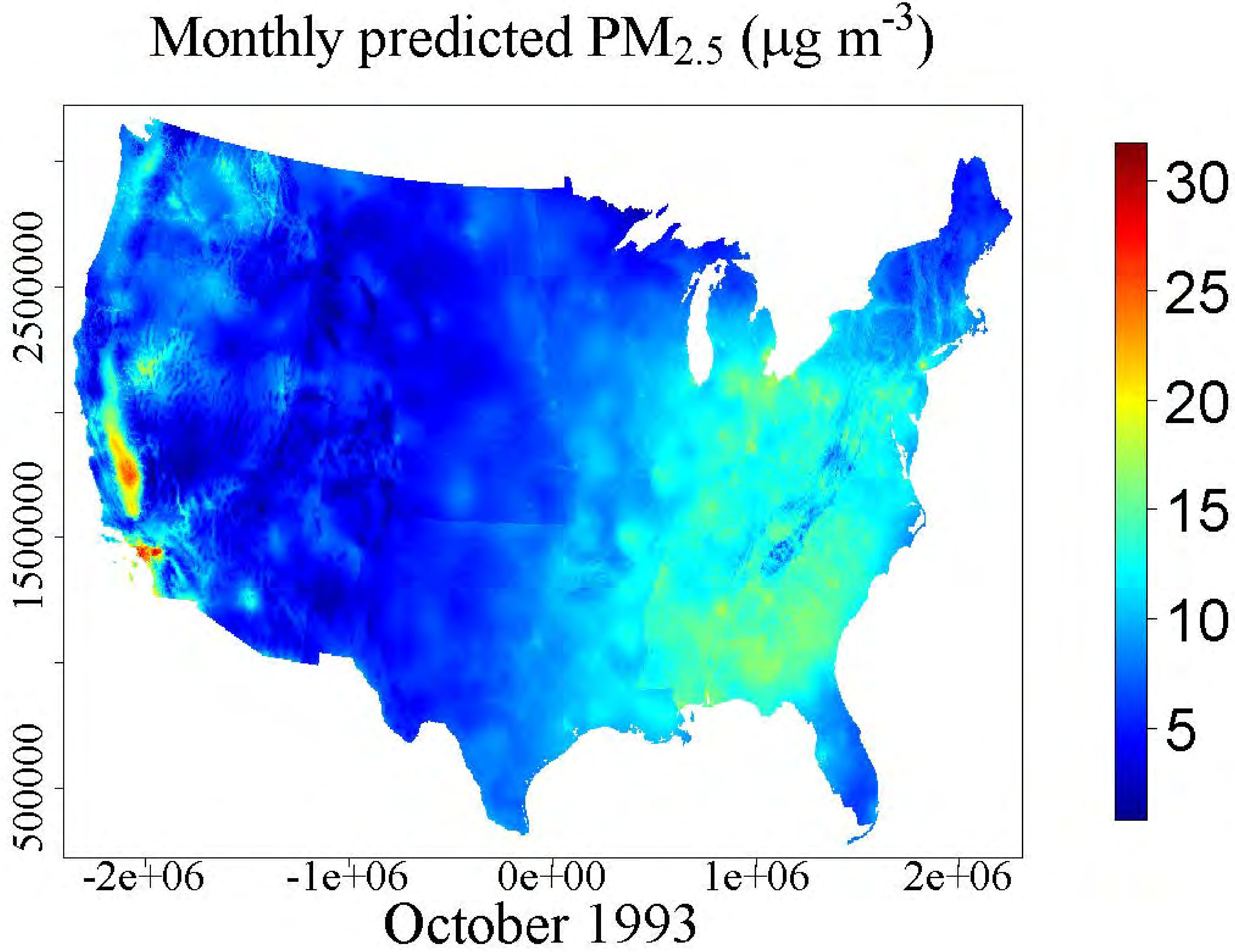
Monthly predicted PM2.5 (μg m-3)
25000000
15000000
5000000
-2e+06
-1e+06
0e+00
1e+06
2e+06
October 1993
30
25
20
15
10
5

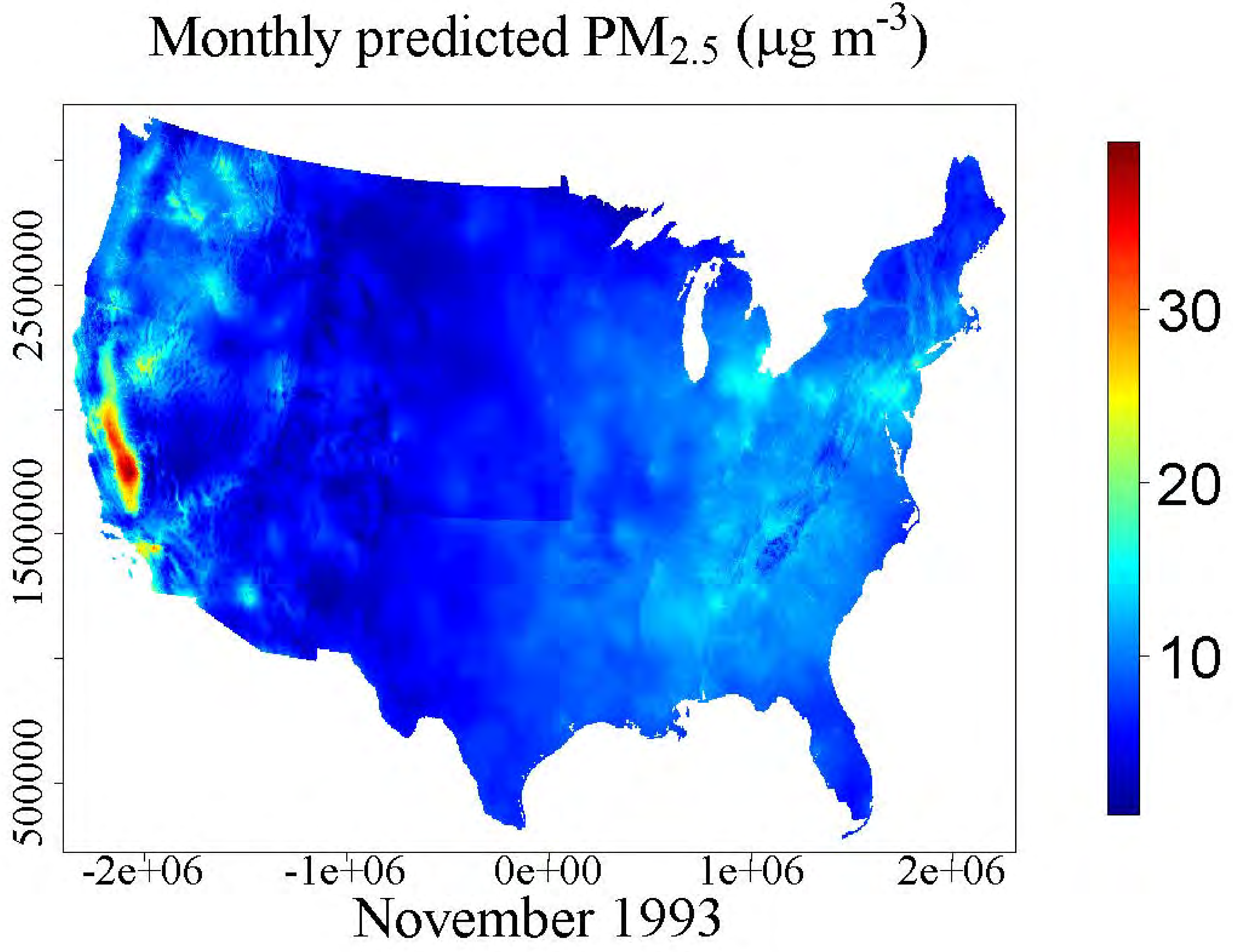
Monthly predicted PM2.5 (μg m-3)
25000000
15000000
5000000
-2e+06
-1e+06
0e+00
1e+06
2e+06
November 1993
30
20
10

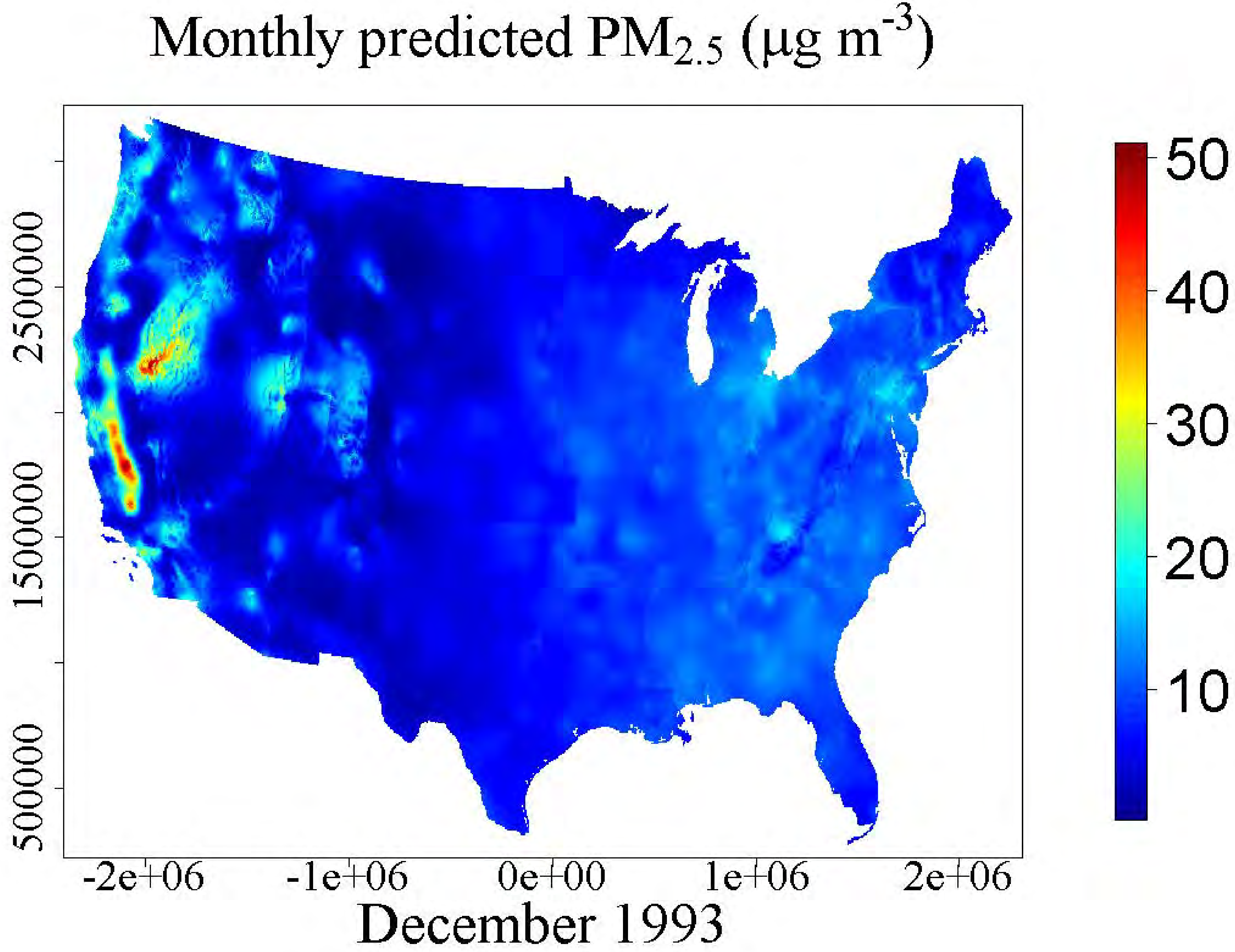
Monthly predicted PM2.5 (μg m-3)
25000000
15000000
5000000
-2e+06
-1e+06
0e+00
1e+06
2e+06
50
40
30
20
10
December 1993

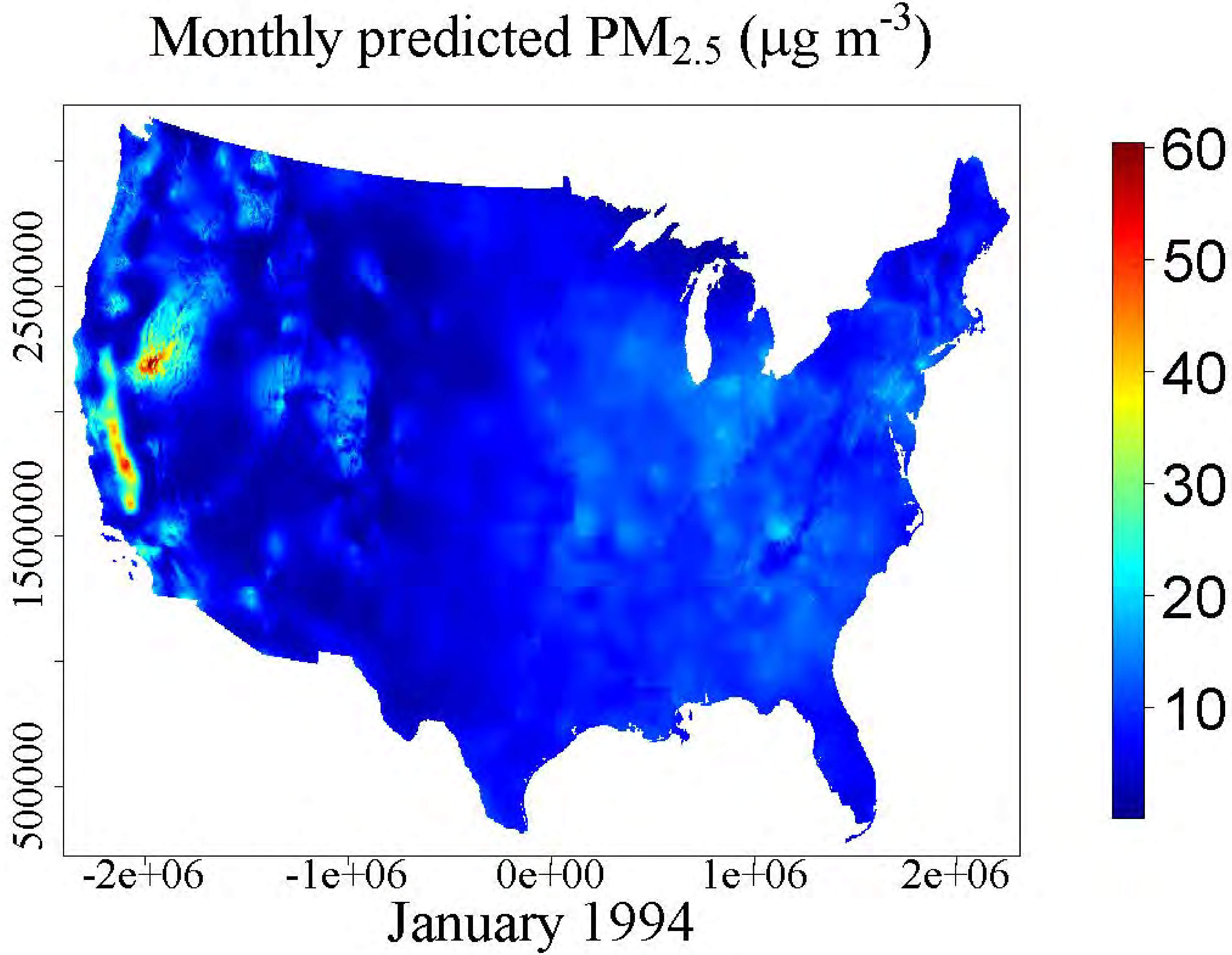

Monthly predicted PM2.5 (μg m-3)
25000000
15000000
5000000
-2e+06
-1e+06
0e+00
1e+06
2e+06
January 1994
60
50
40
30
20
10

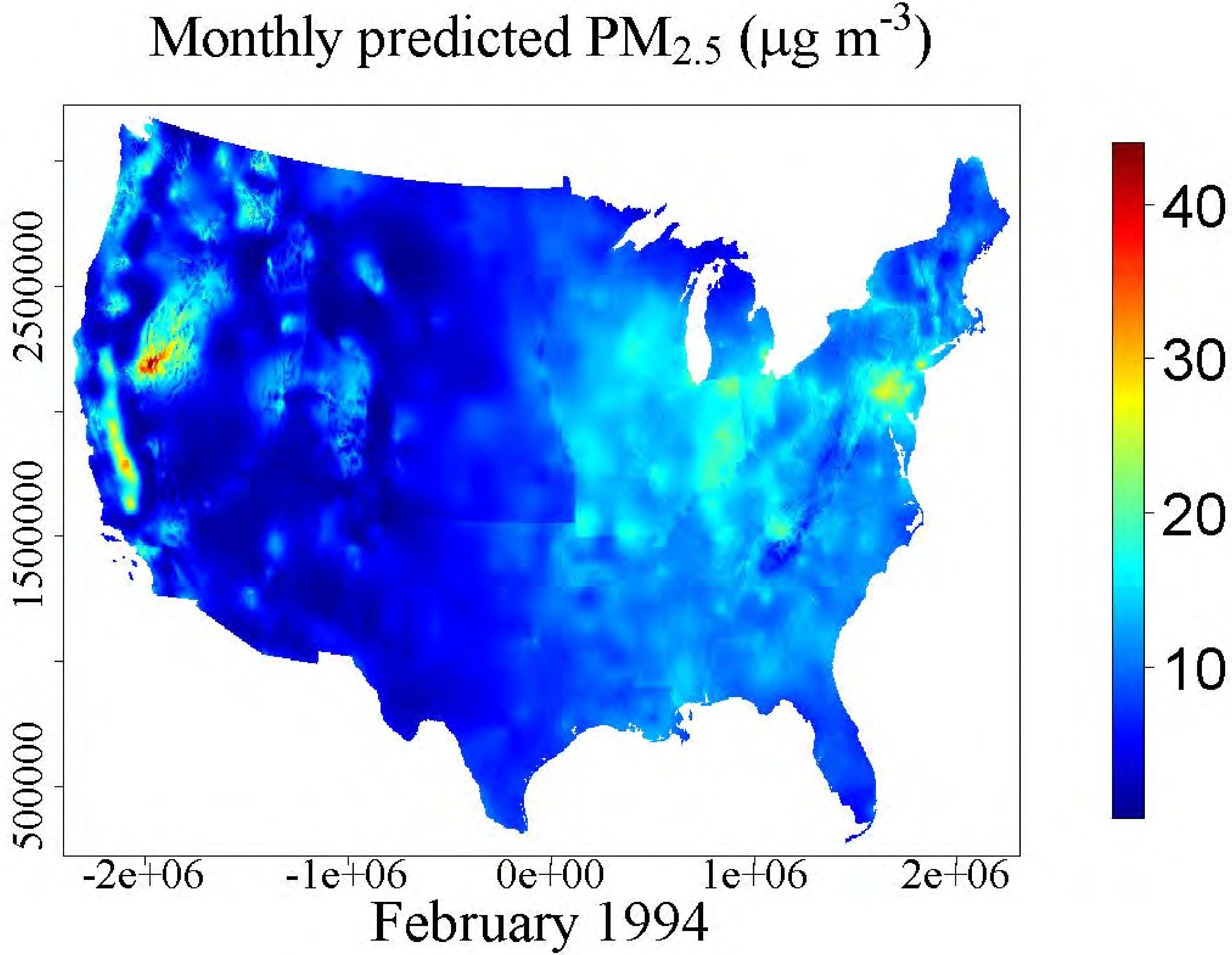
Monthly predicted PM2.5 (μg m-3)
25000000
15000000
5000000
-2e+06
-1e+06
0e+00
1e+06
2e+06
40
30
20
10
February 1994

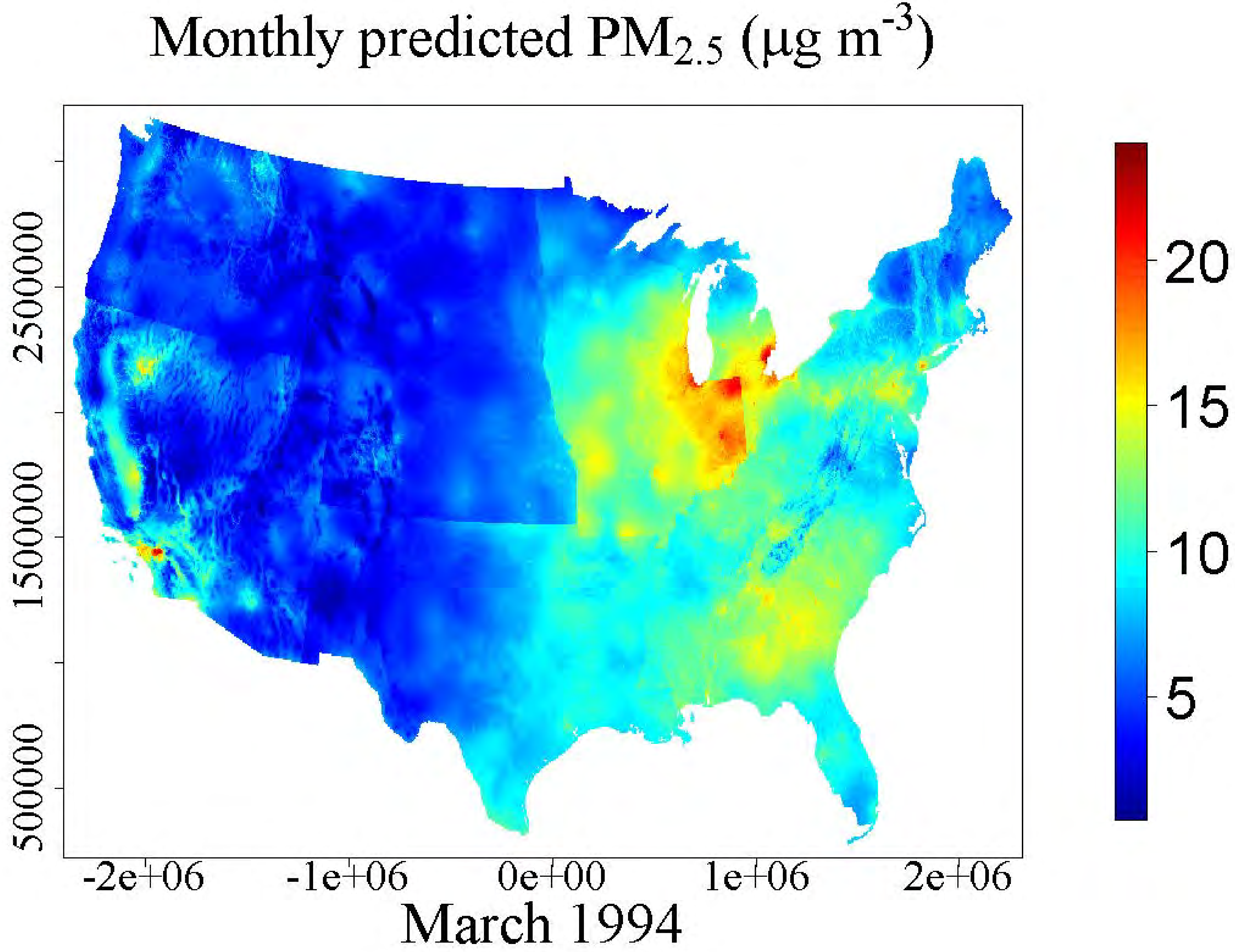

Monthly predicted PM2.5 (μg m-3)
25000000
15000000
5000000
-2e+06
-1e+06
0e+00
1e+06
2e+06
March 1994
20
15
10
5

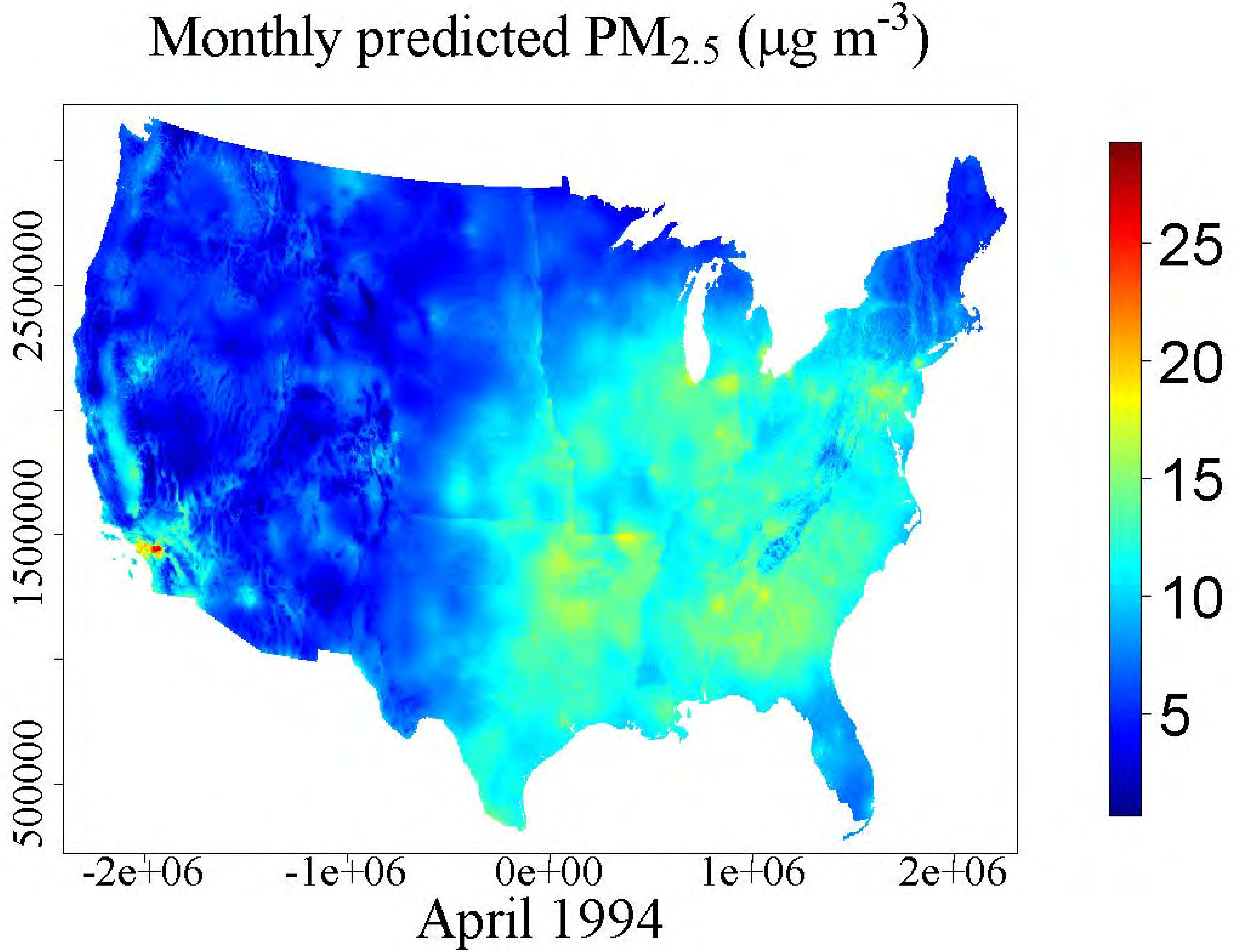

Monthly predicted PM2.5 (µg m-3)
25000000
15000000
5000000
-2e+06
-1e+06
0e+00
1e+06
2e+06
25
20
15
10
5
April 1994

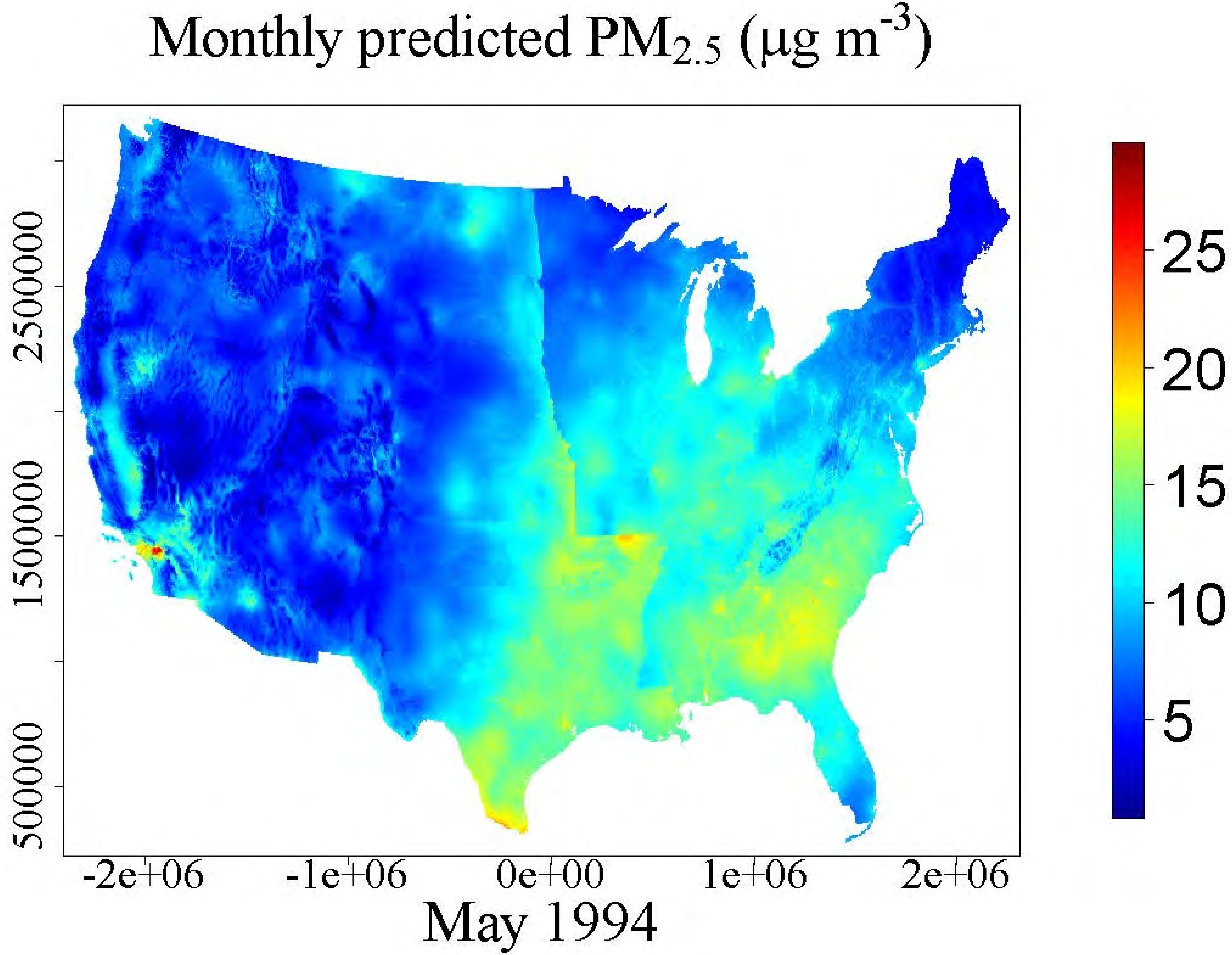

Monthly predicted PM2.5 (μg m-3)
25000000
15000000
5000000
-2e+06
-1e+06
0e+00
1e+06
2e+06
25
20
15
10
5
May 1994

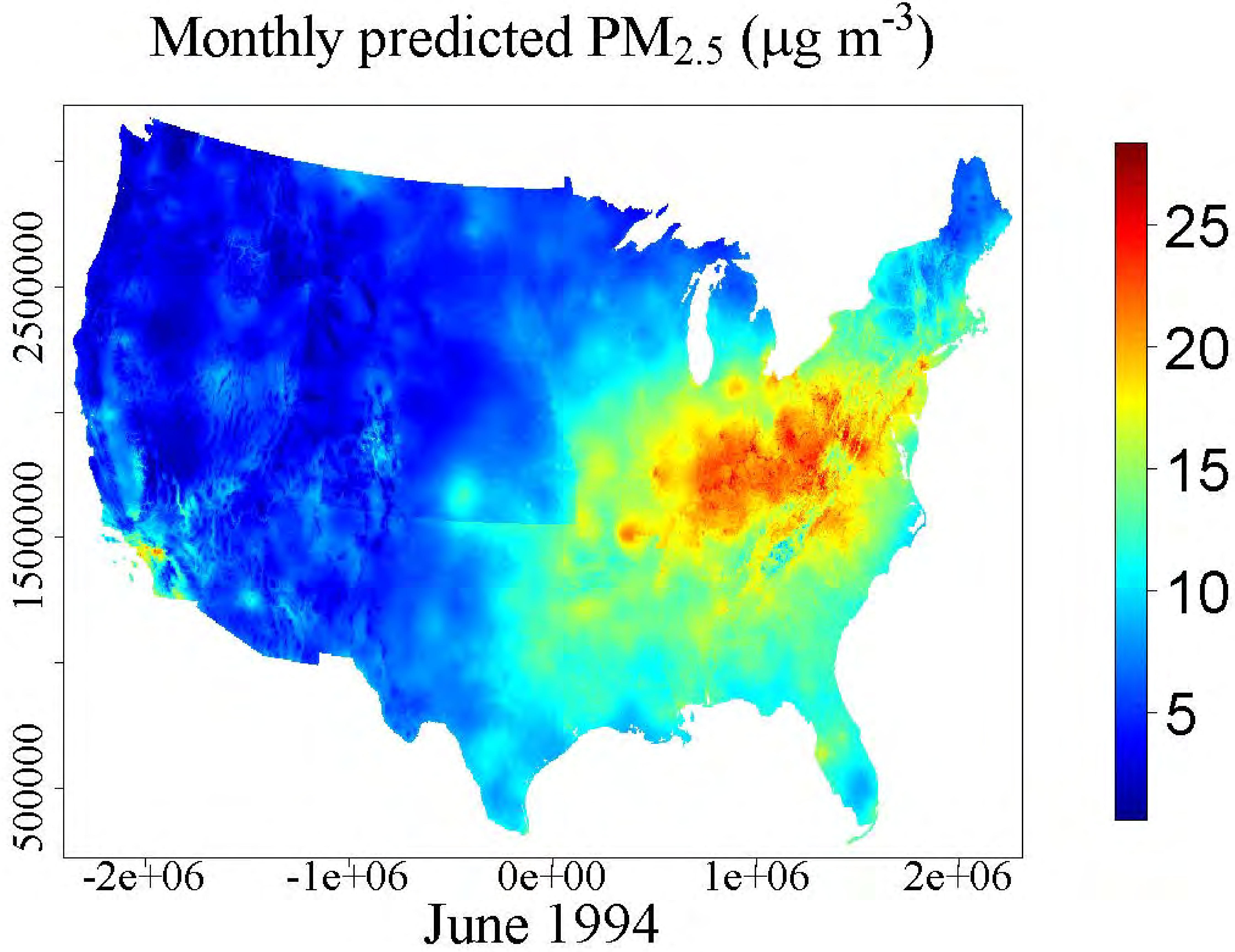

Monthly predicted PM2.5 (μg m-3)
25000000
15000000
5000000
-2e+06
-1e+06
0e+00
1e+06
2e+06
June 1994
25
20
15
10
5

Monthly predicted $PM_{2.5}$ ($\mu g\ m^{-3}$)

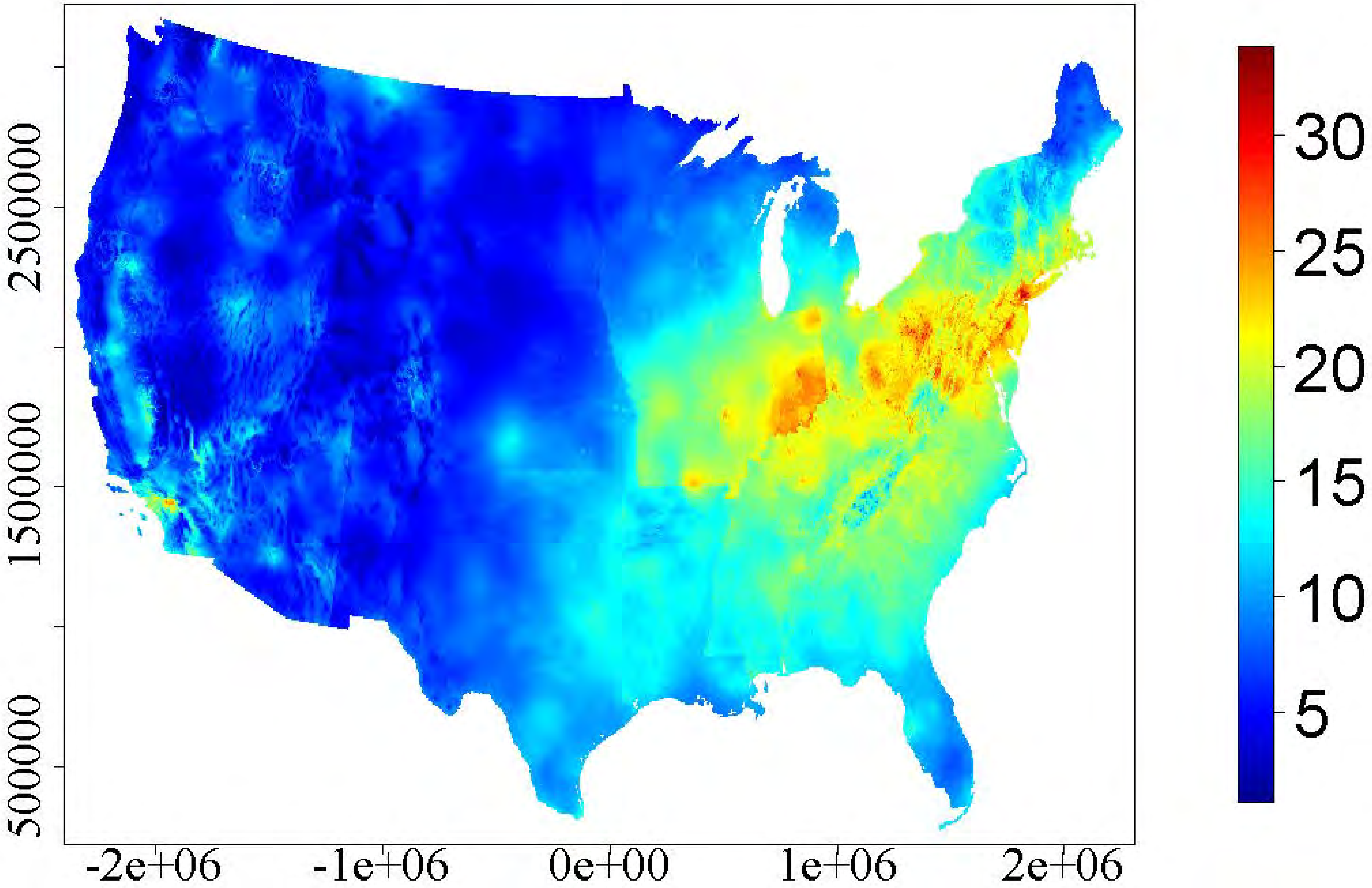

July 1994

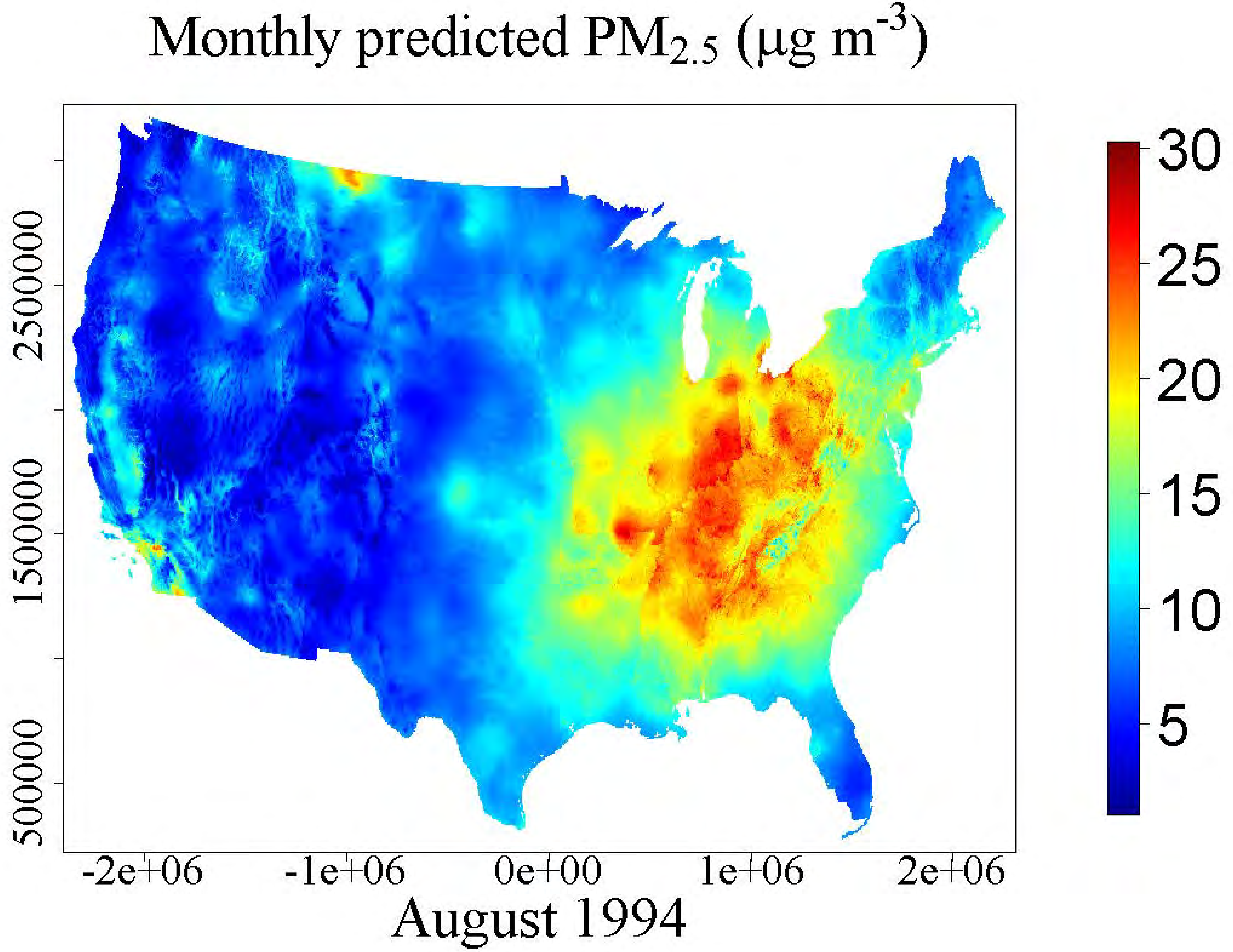
Monthly predicted PM2.5 (μg m-3)
25000000
15000000
5000000
-2e+06
-1e+06
0e+00
1e+06
2e+06
August 1994
30
25
20
15
10
5

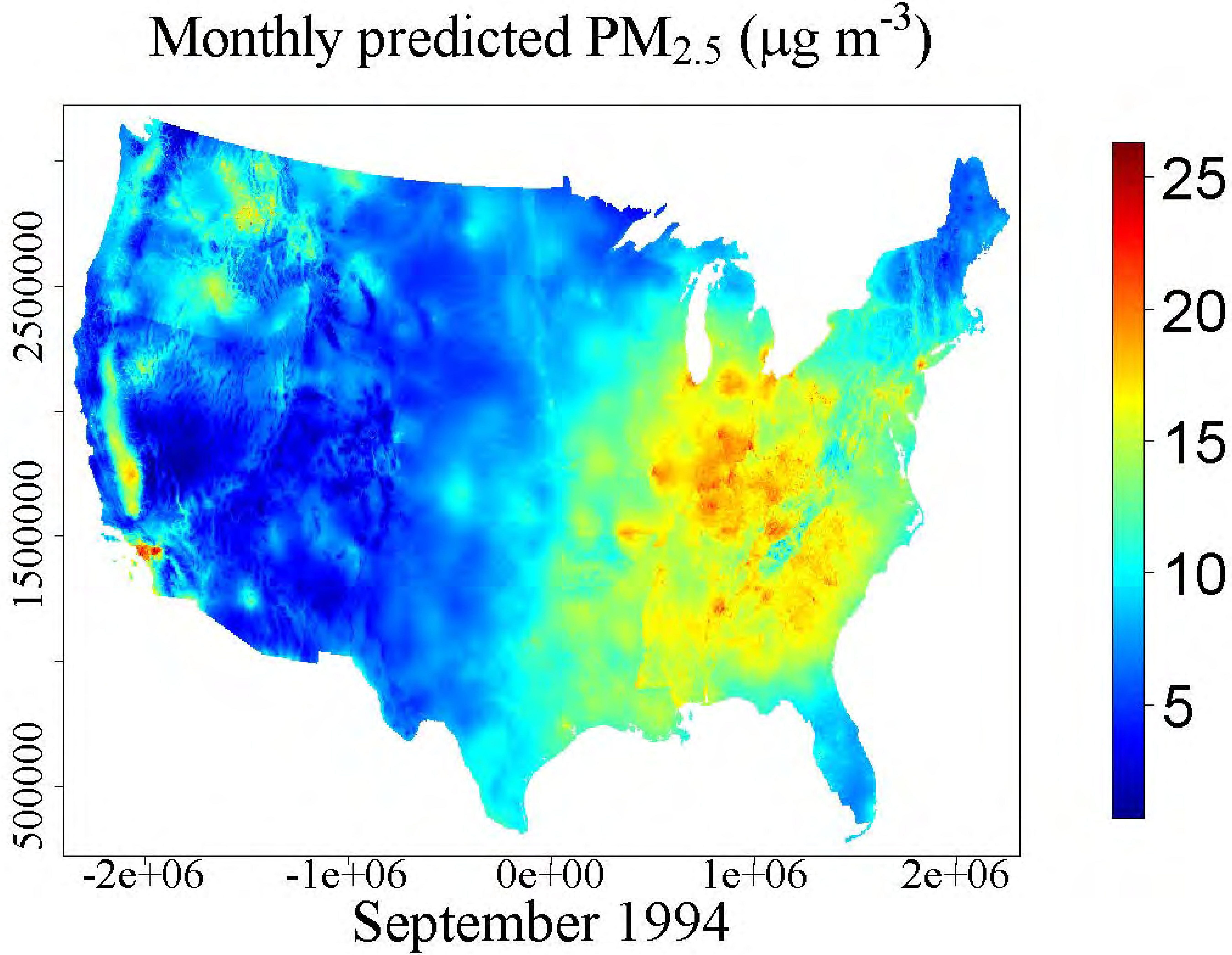
Monthly predicted PM2.5 (μg m-3)
25000000
15000000
5000000
-2e+06
-1e+06
0e+00
1e+06
2e+06
25
20
15
10
5
September 1994

Monthly predicted $PM_{2.5}$ ($\mu g\ m^{-3}$)

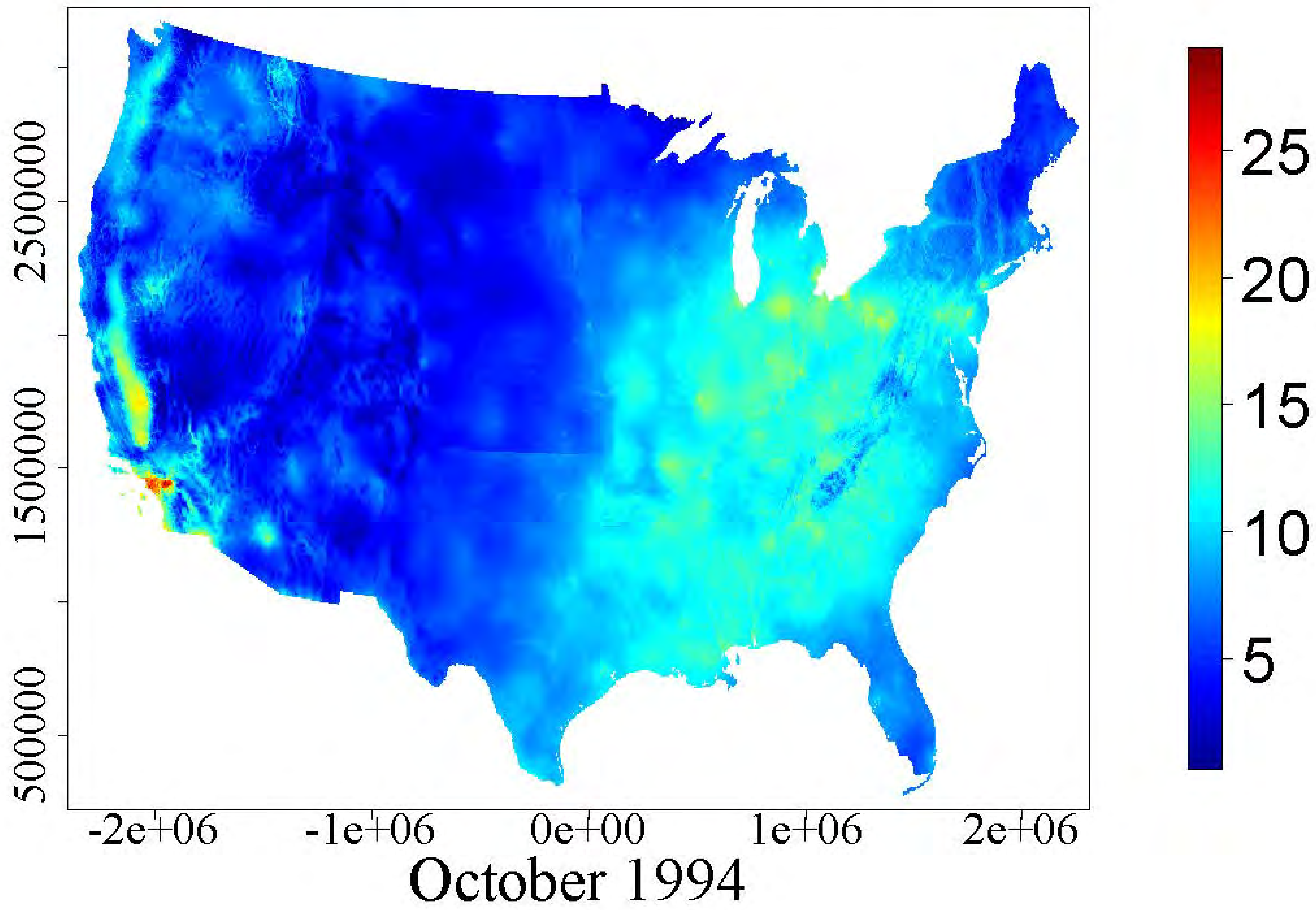

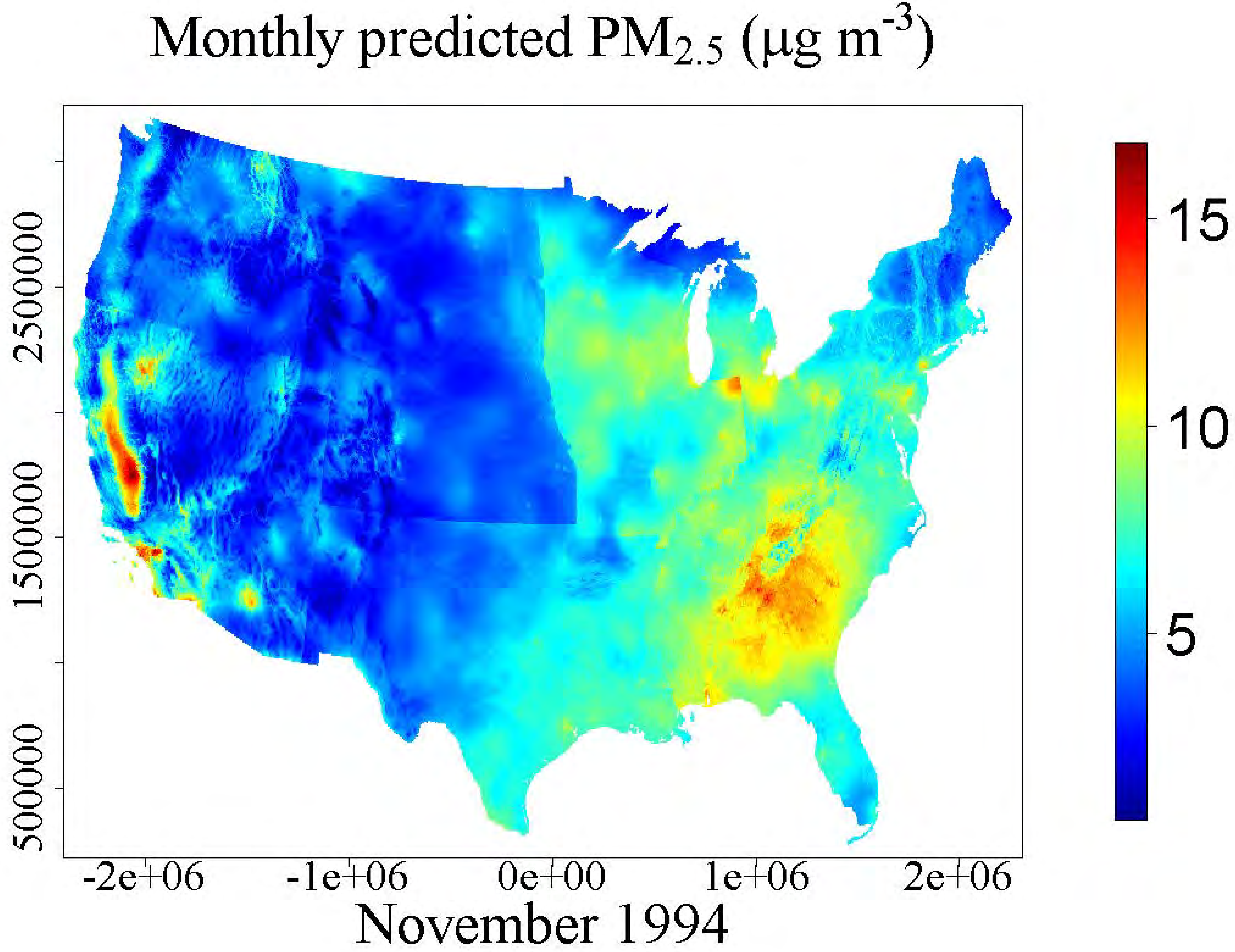

Monthly predicted $PM_{2.5}$ ($\mu g\ m^{-3}$)
25000000
15000000
5000000
-2e+06
-1e+06
0e+00
1e+06
2e+06
15
10
5
November 1994

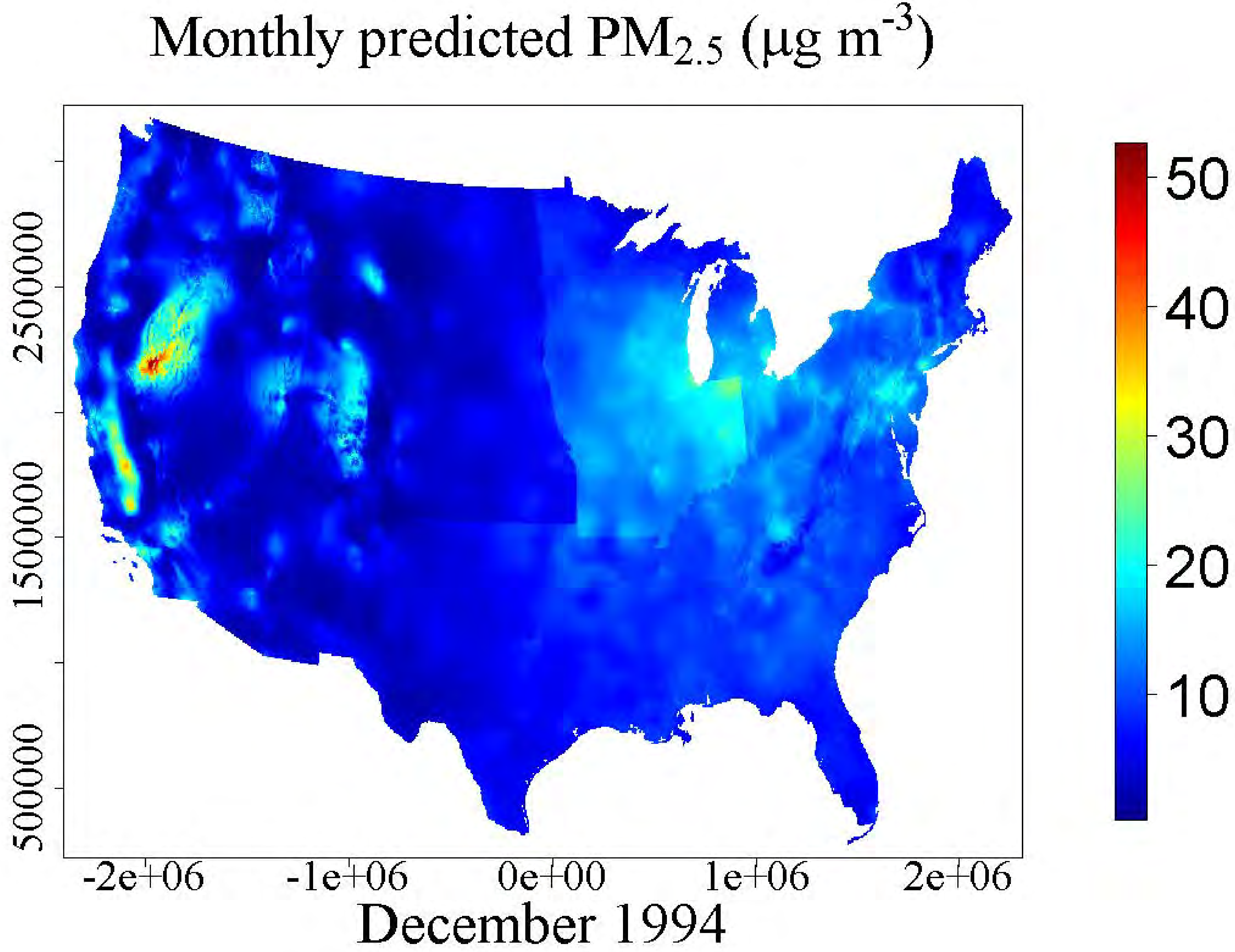

Monthly predicted $PM_{2.5}$ ($\mu g\ m^{-3}$)
25000000
15000000
5000000
-2e+06
-1e+06
0e+00
1e+06
2e+06
December 1994
50
40
30
20
10

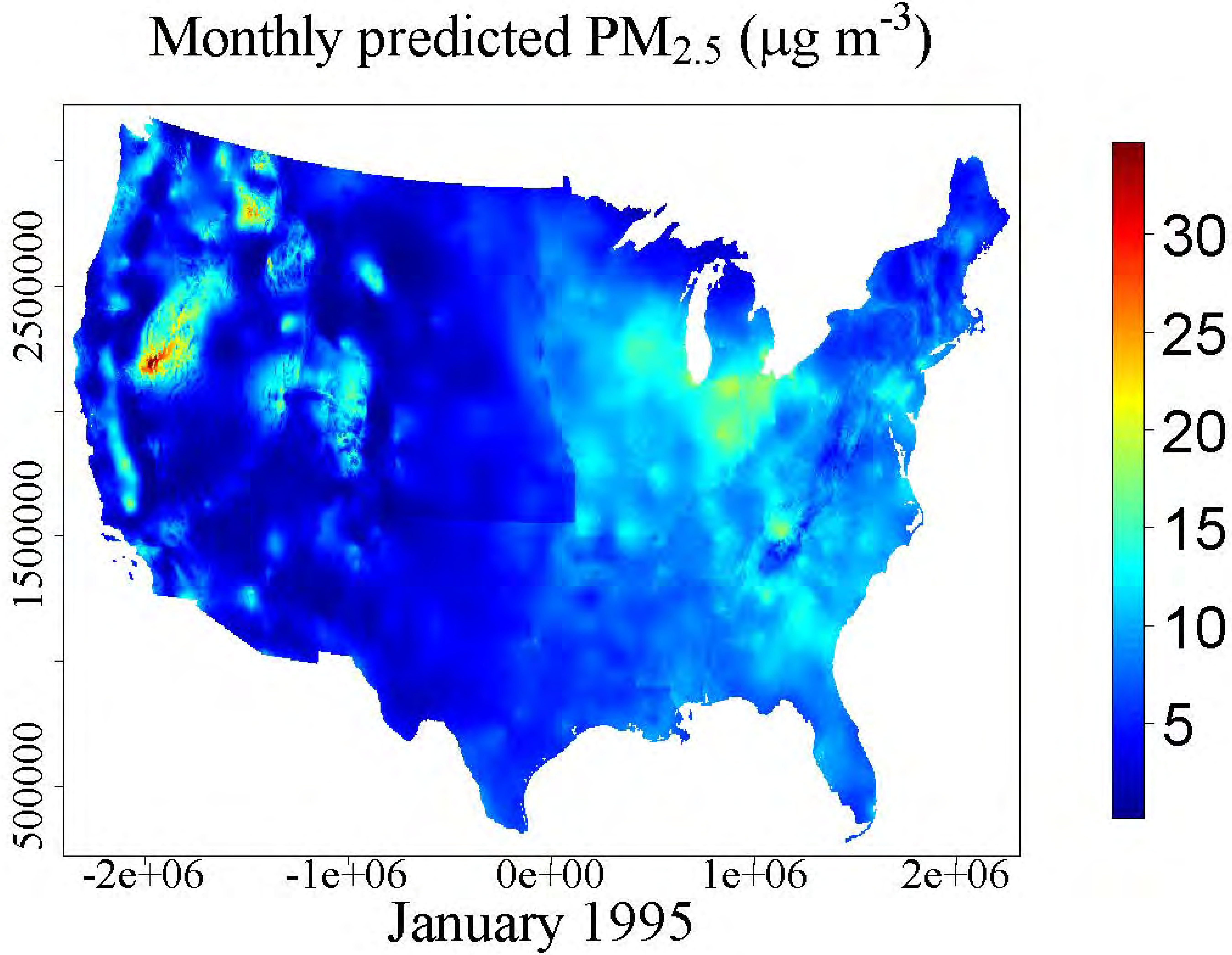
Monthly predicted $PM_{2.5}$ ($\mu g\ m^{-3}$)
25000000
15000000
5000000
-2e+06
-1e+06
0e+00
1e+06
2e+06
January 1995
30
25
20
15
10
5

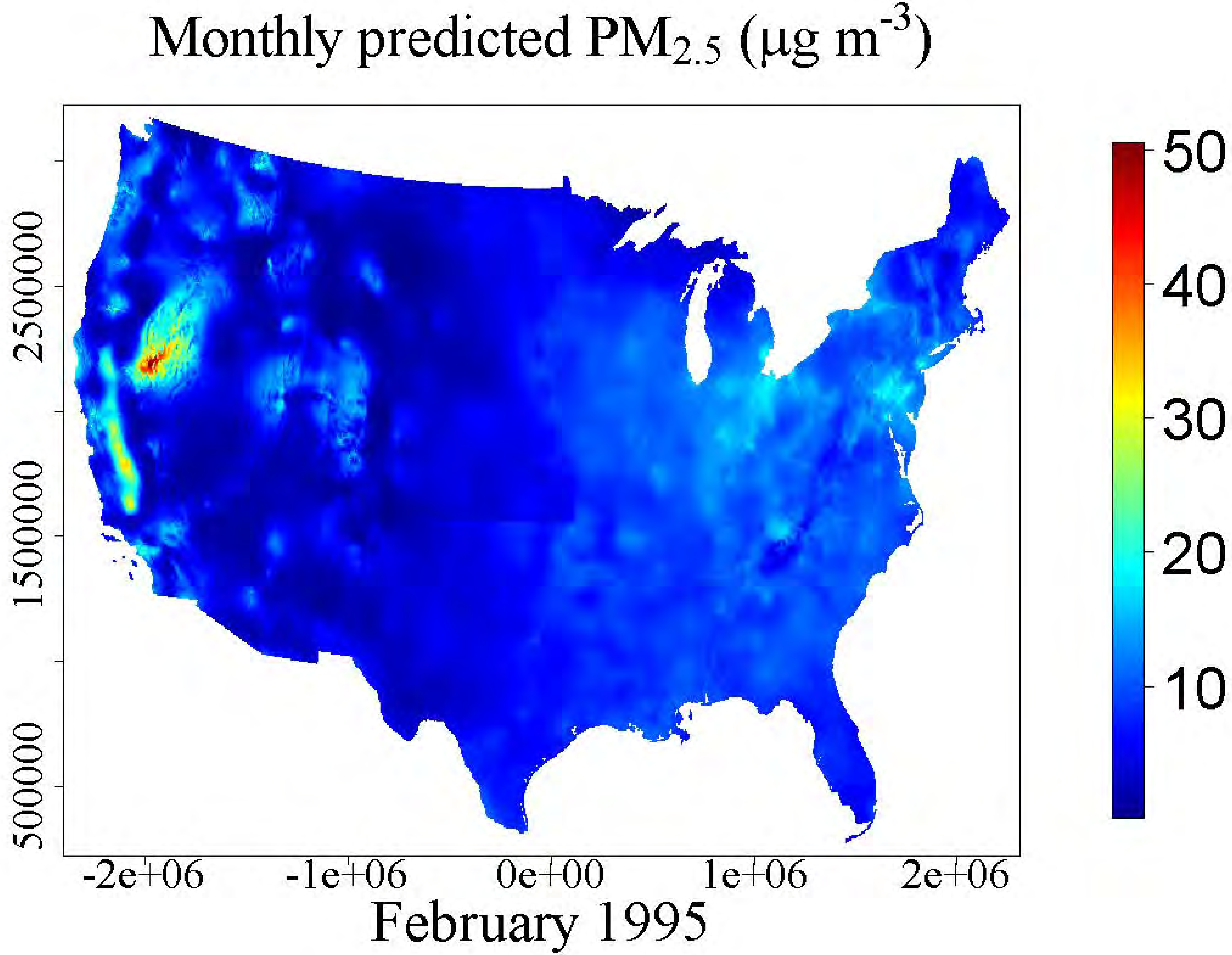
Monthly predicted PM2.5 (μg m-3)
25000000
15000000
5000000
-2e+06
-1e+06
0e+00
1e+06
2e+06
50
40
30
20
10
February 1995

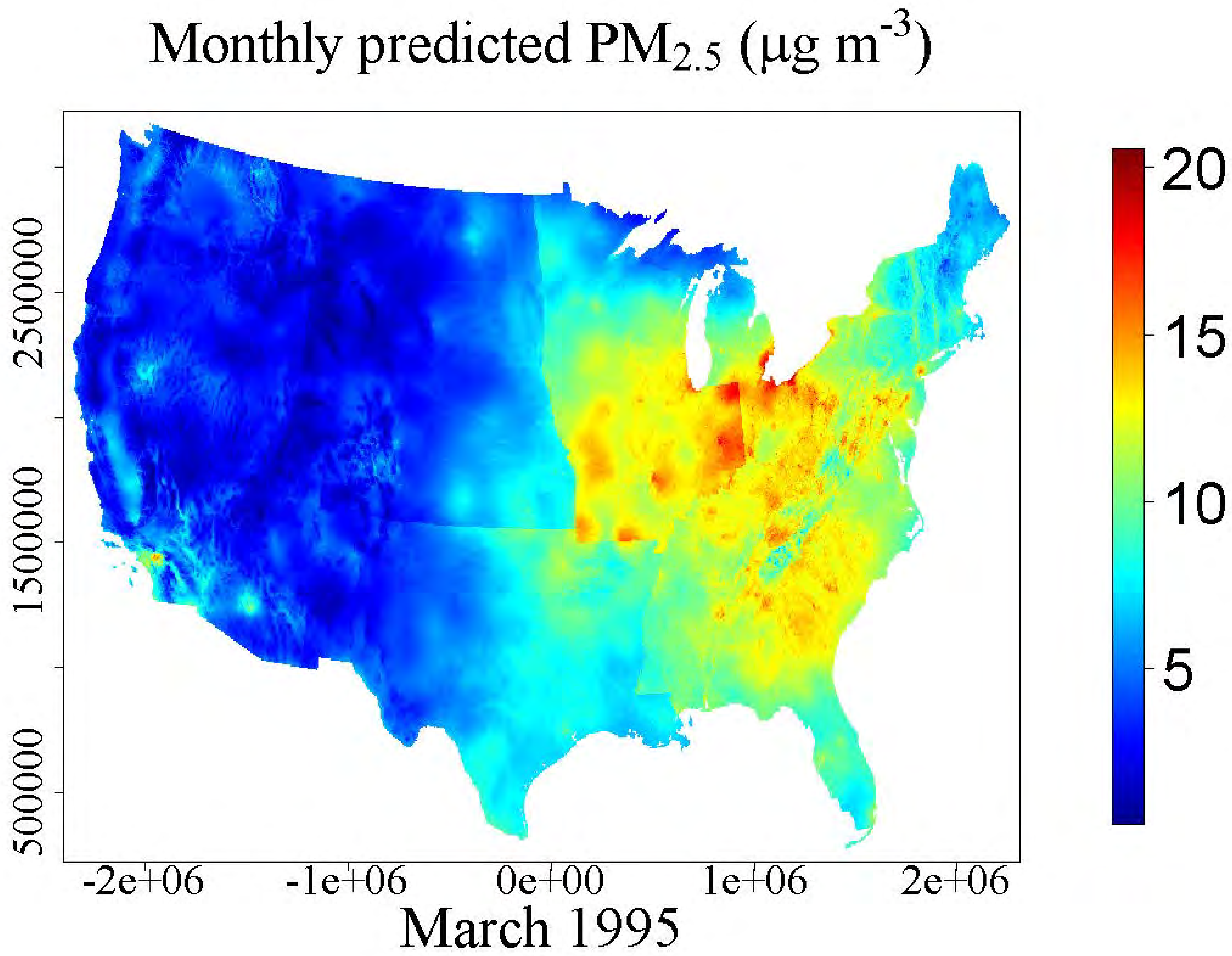
Monthly predicted $PM_{2.5}$ ($\mu g\ m^{-3}$)
25000000
15000000
5000000
-2e+06
-1e+06
0e+00
1e+06
2e+06
20
15
10
5
March 1995

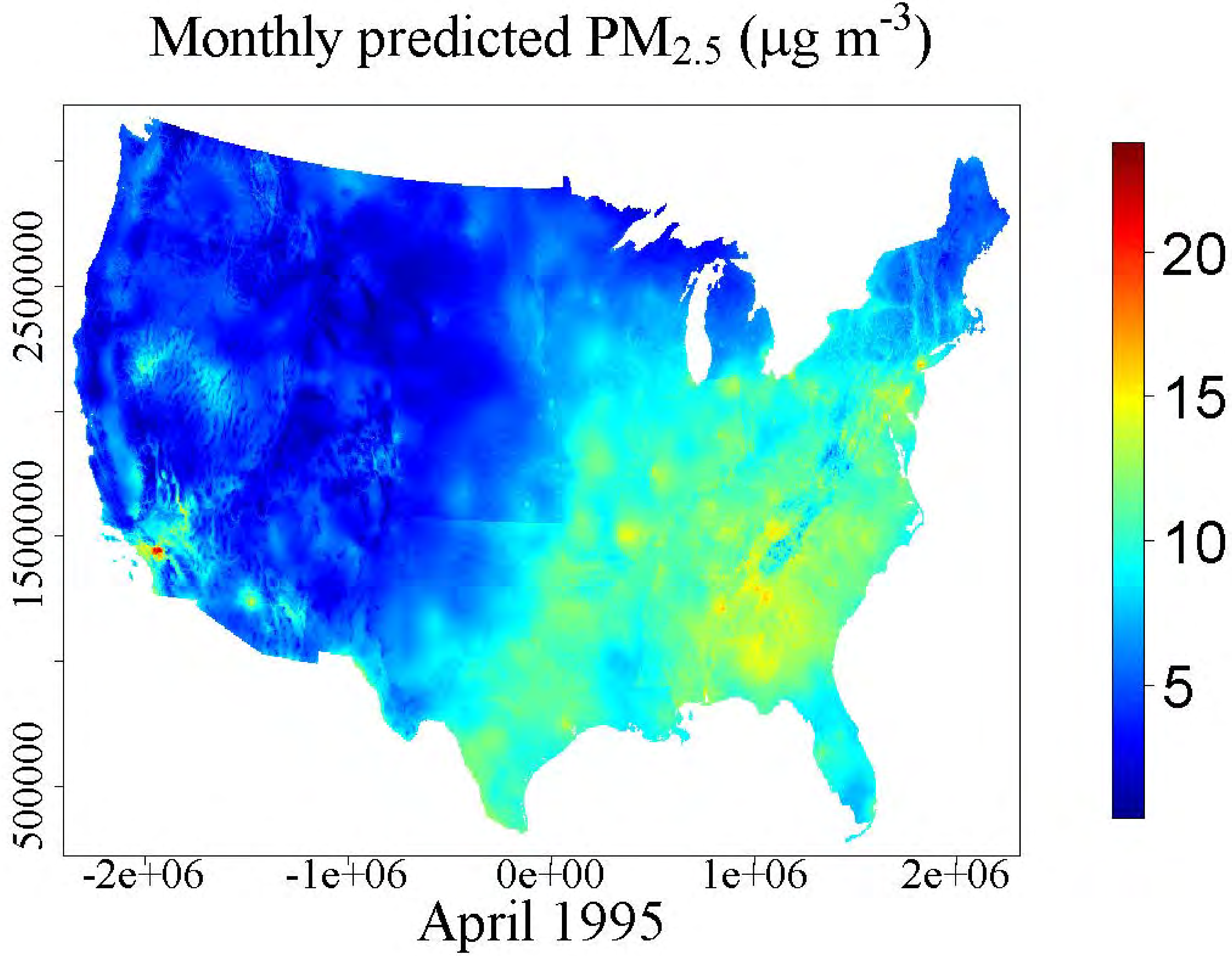

Monthly predicted PM2.5 (µg m-3)
25000000
15000000
5000000
-2e+06
-1e+06
0e+00
1e+06
2e+06
April 1995
20
15
10
5

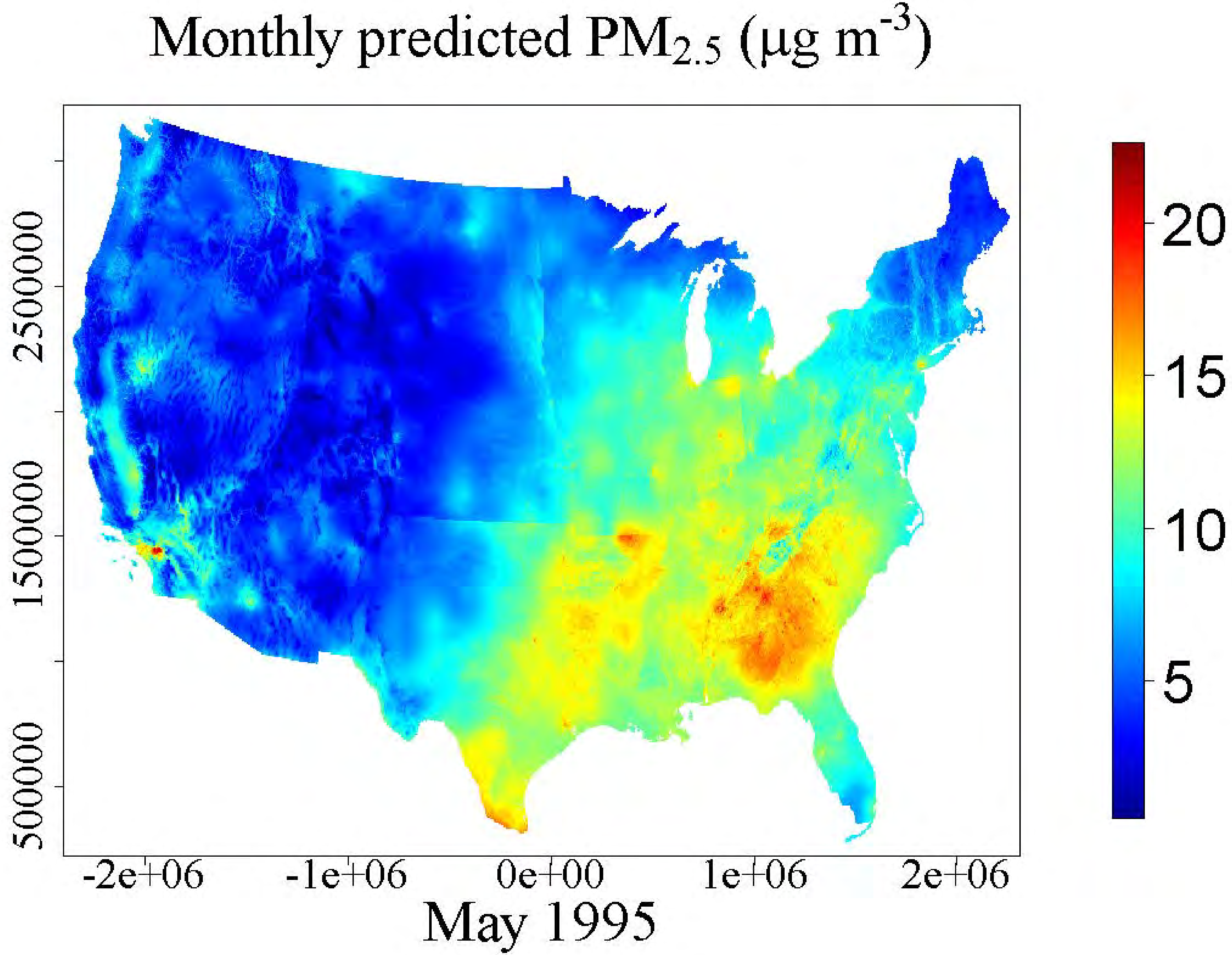

Monthly predicted PM2.5 (μg m-3)
25000000
15000000
5000000
-2e+06
-1e+06
0e+00
1e+06
2e+06
May 1995
20
15
10
5

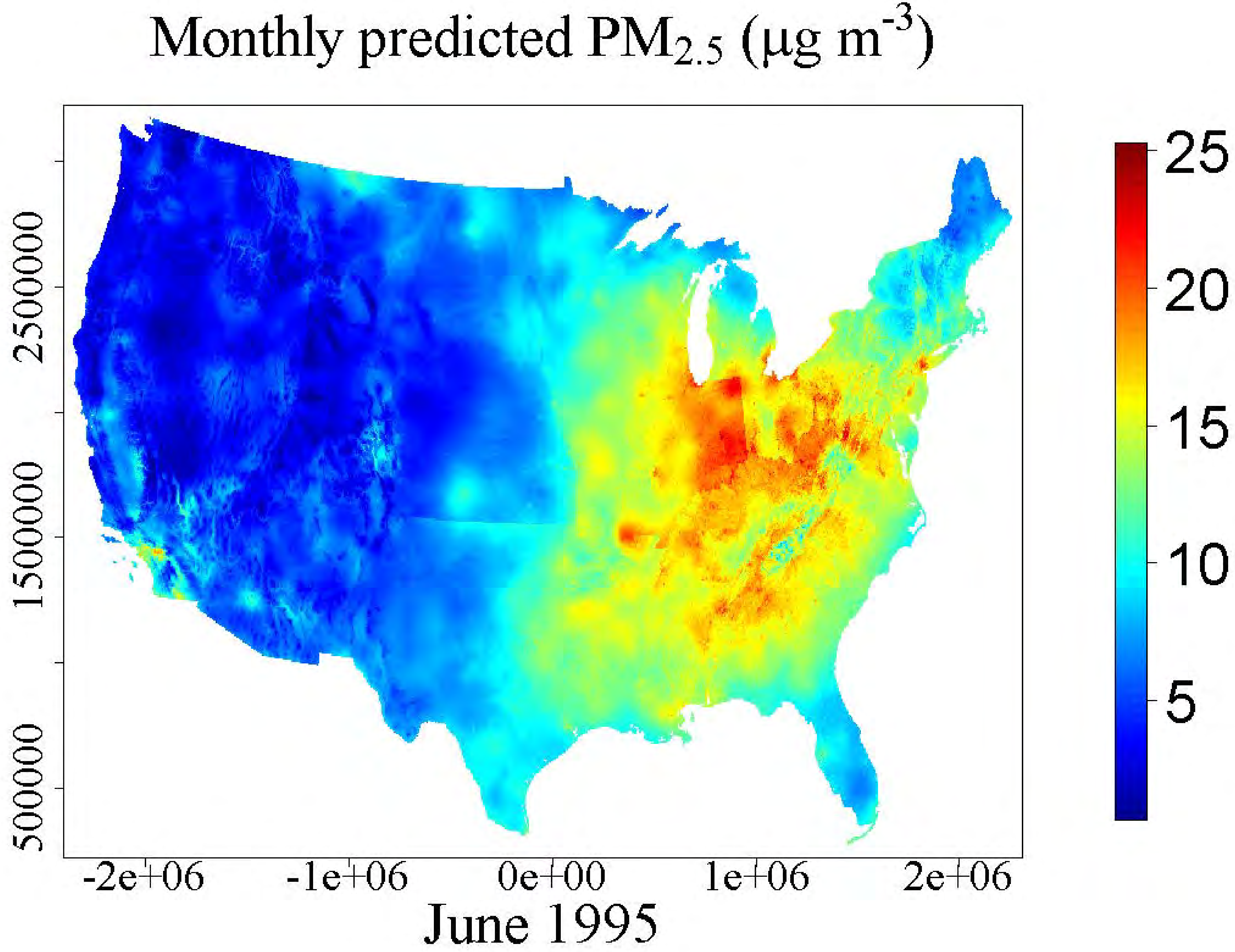
Monthly predicted PM2.5 (μg m-3)
25000000
15000000
5000000
-2e+06
-1e+06
0e+00
1e+06
2e+06
June 1995
25
20
15
10
5

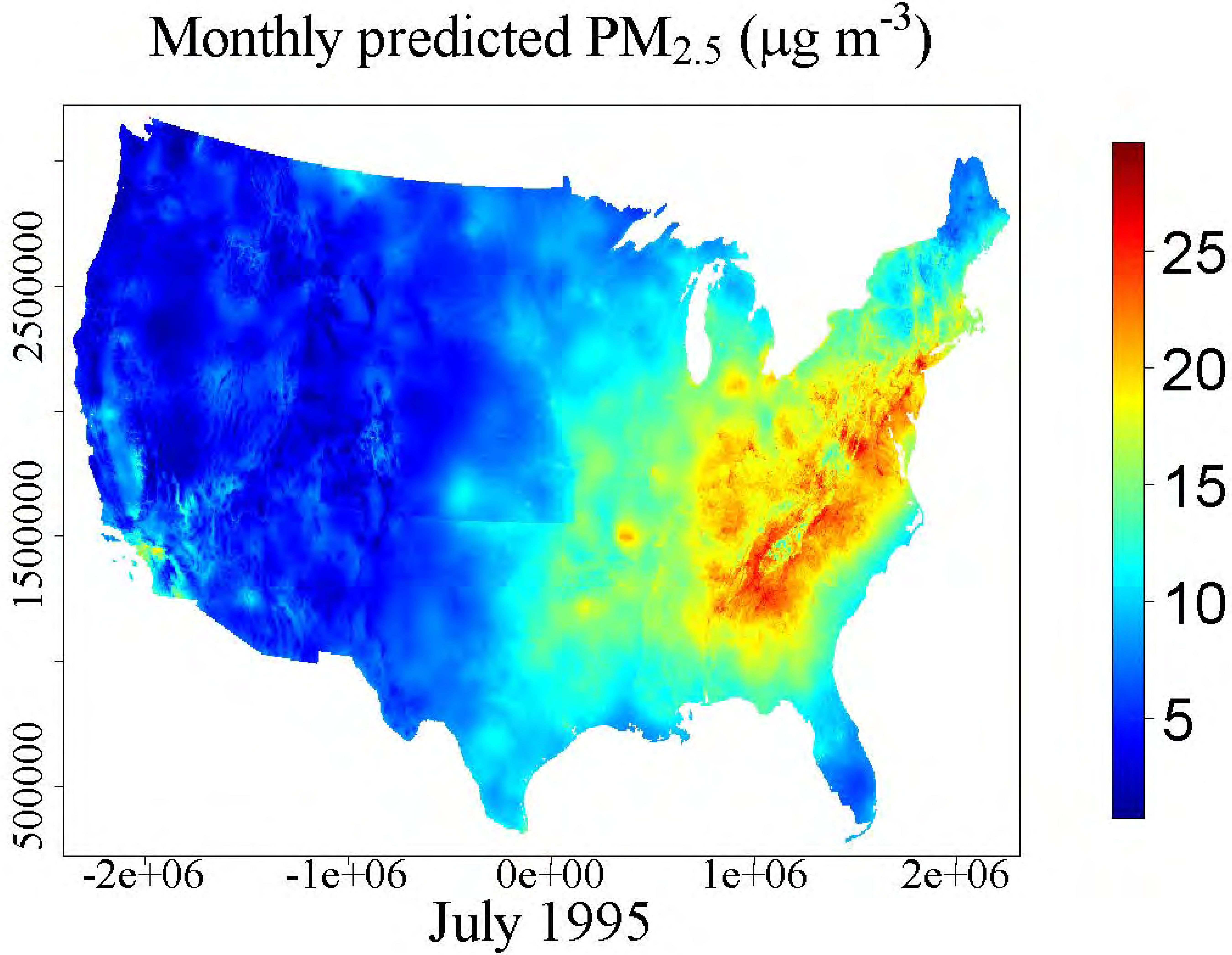

Monthly predicted PM2.5 (μg m-3)
25000000
15000000
5000000
-2e+06
-1e+06
0e+00
1e+06
2e+06
25
20
15
10
5
July 1995

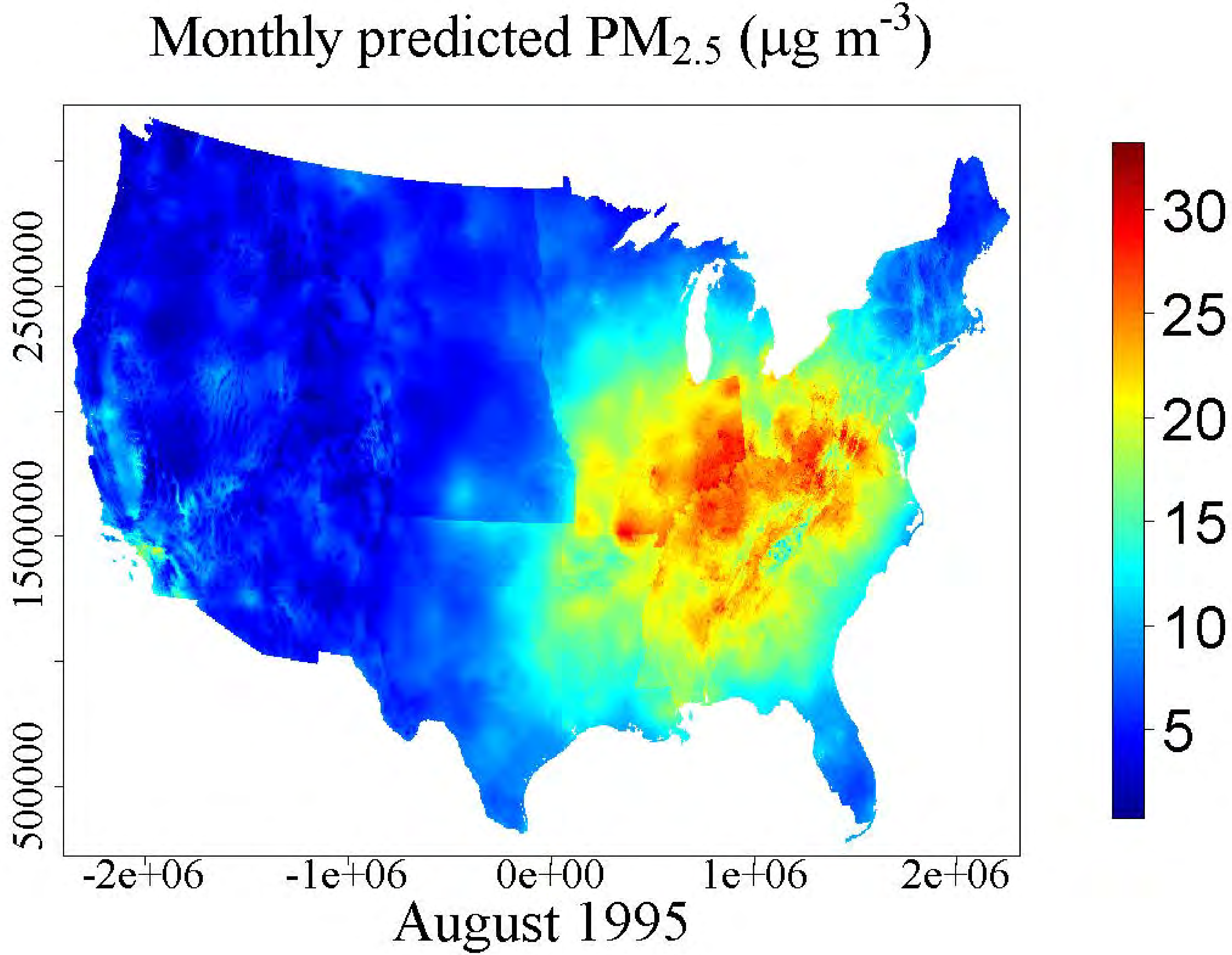
Monthly predicted PM2.5 (μg m-3)
25000000
15000000
5000000
-2e+06
-1e+06
0e+00
1e+06
2e+06
August 1995
30
25
20
15
10
5

Monthly predicted $PM_{2.5}$ ($\mu g\ m^{-3}$)

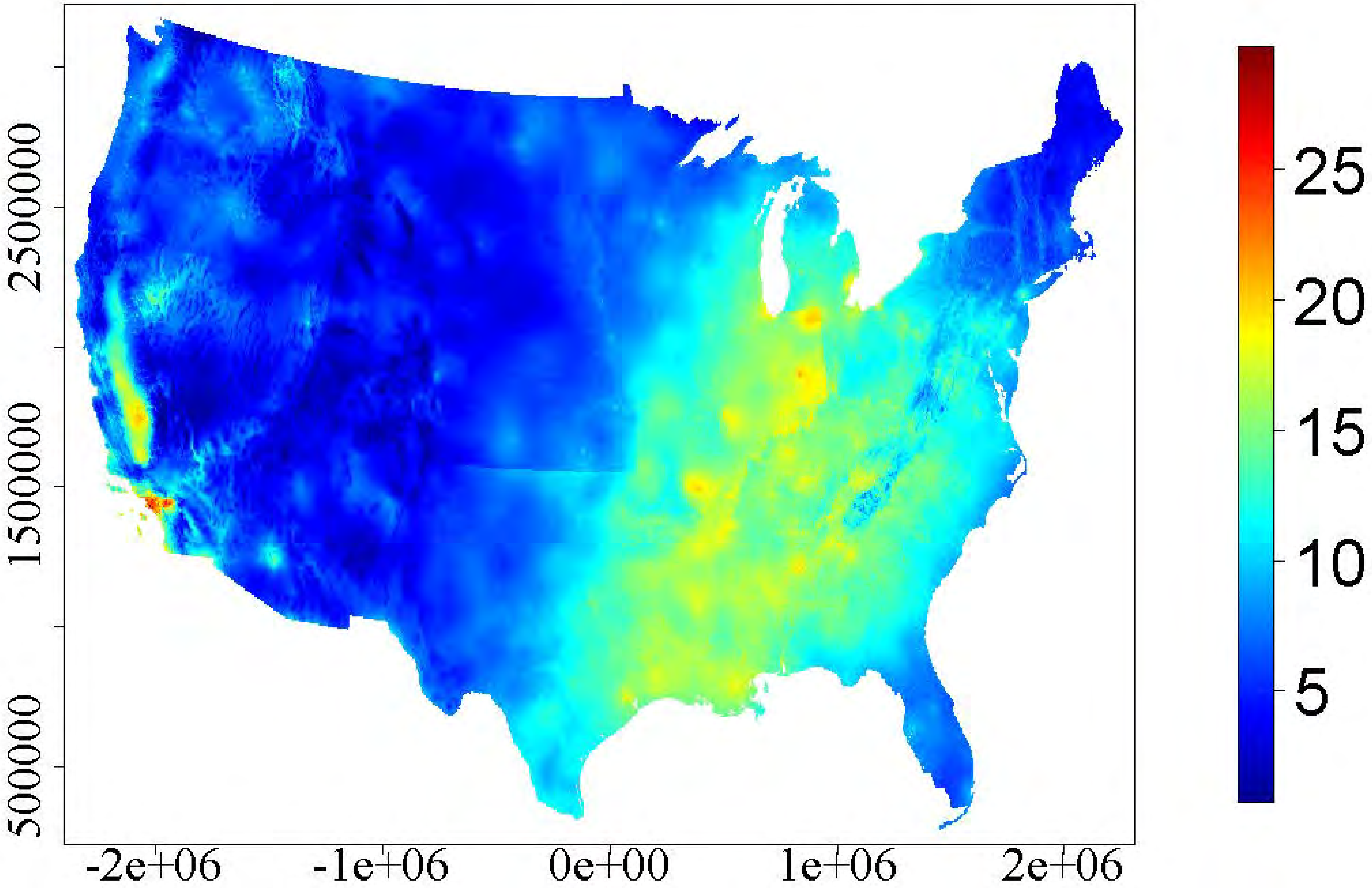

September 1995

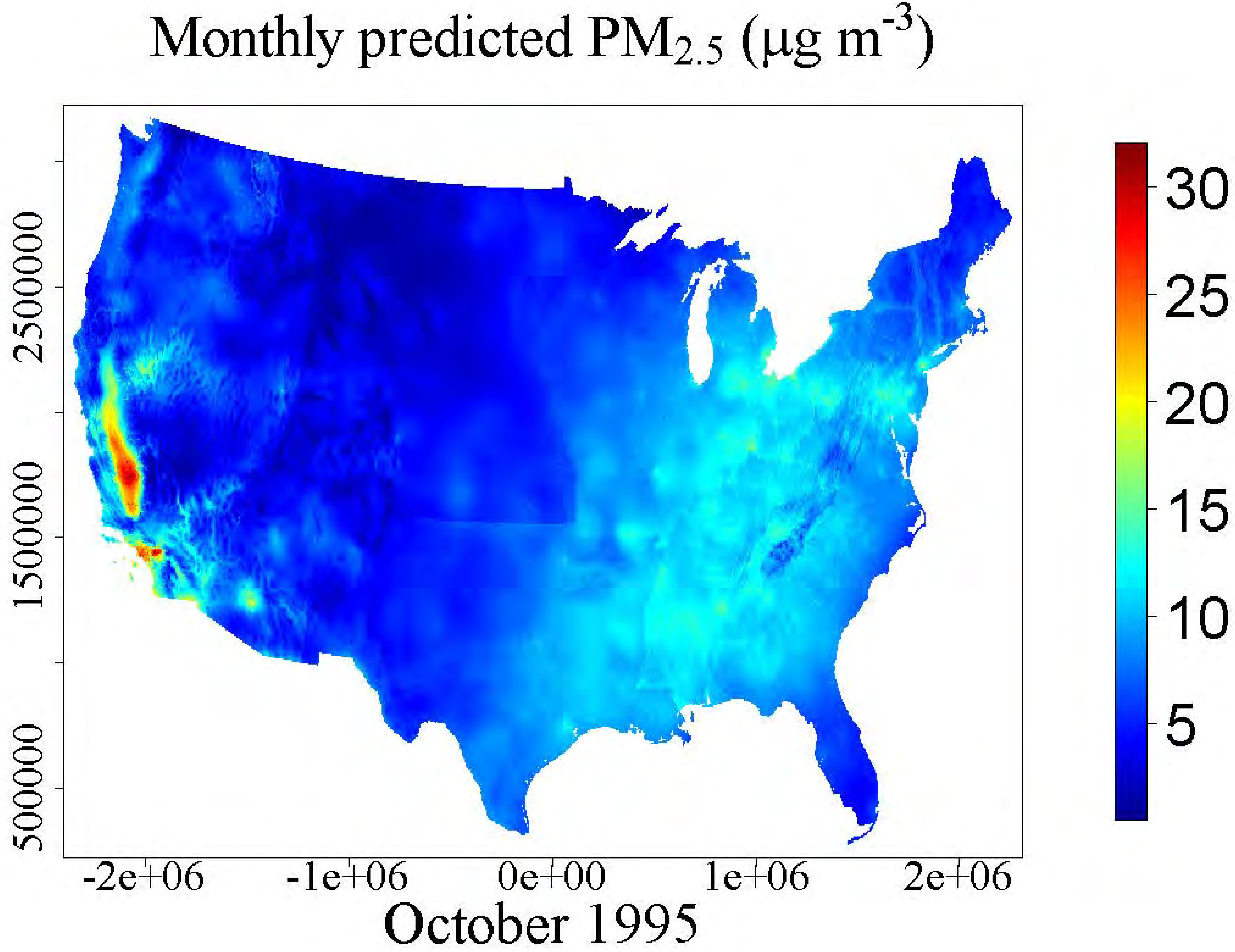
Monthly predicted PM2.5 (μg m-3)
25000000
15000000
5000000
-2e+06
-1e+06
0e+00
1e+06
2e+06
October 1995
30
25
20
15
10
5

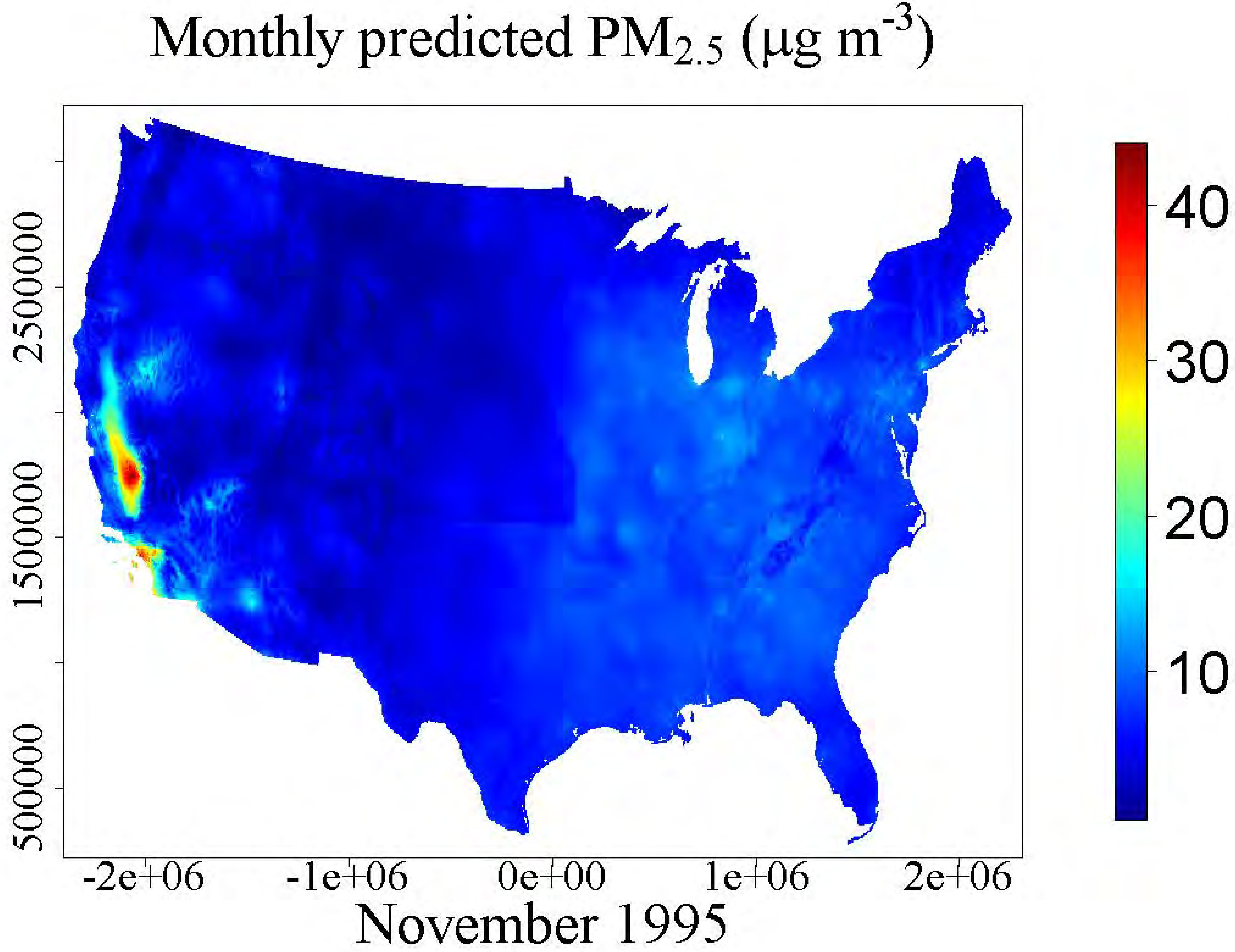
Monthly predicted $PM_{2.5}$ ($\mu g\ m^{-3}$)
25000000
15000000
5000000
-2e+06
-1e+06
0e+00
1e+06
2e+06
40
30
20
10
November 1995

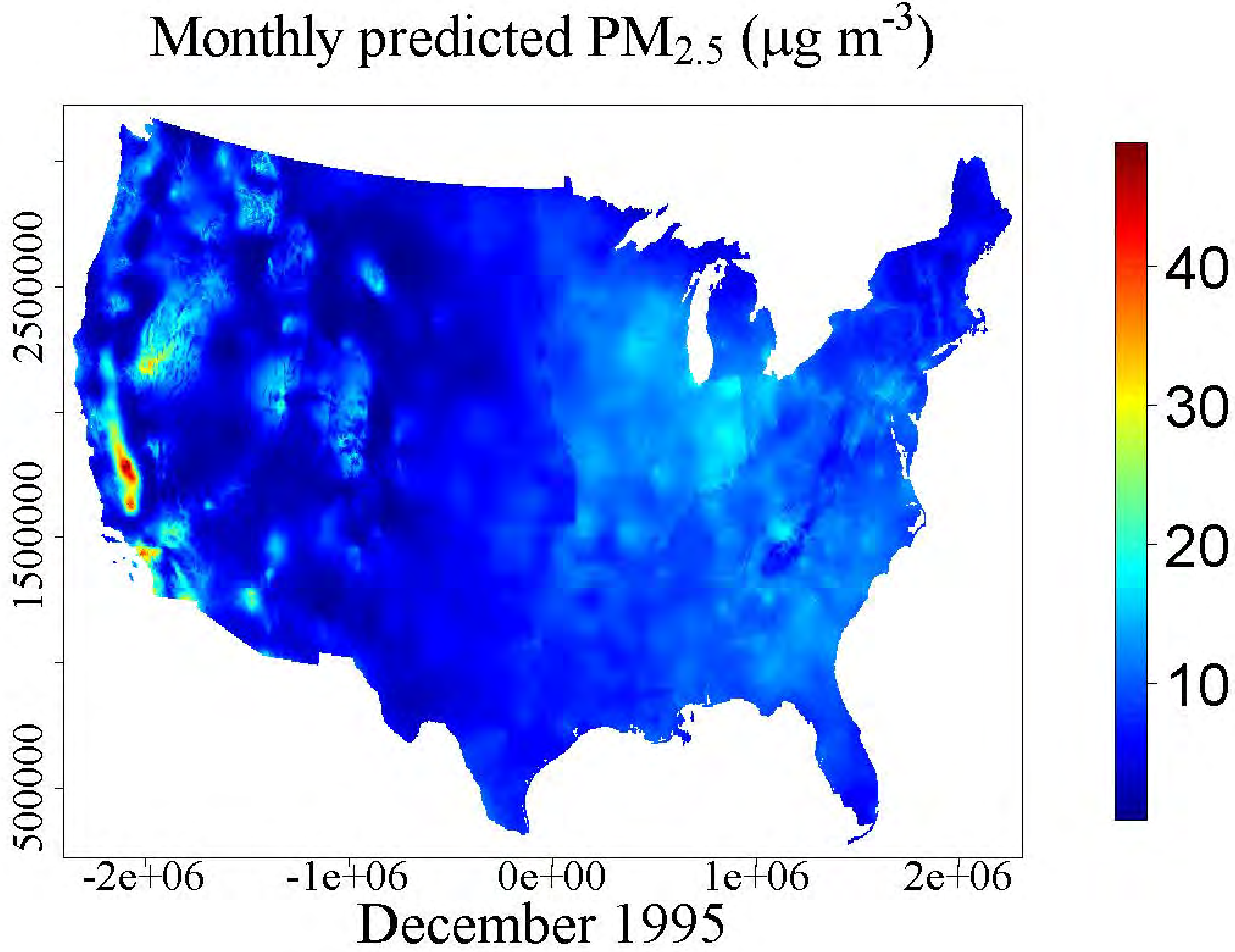
Monthly predicted PM2.5 (μg m-3)
25000000
15000000
5000000
-2e+06
-1e+06
0e+00
1e+06
2e+06
40
30
20
10
December 1995

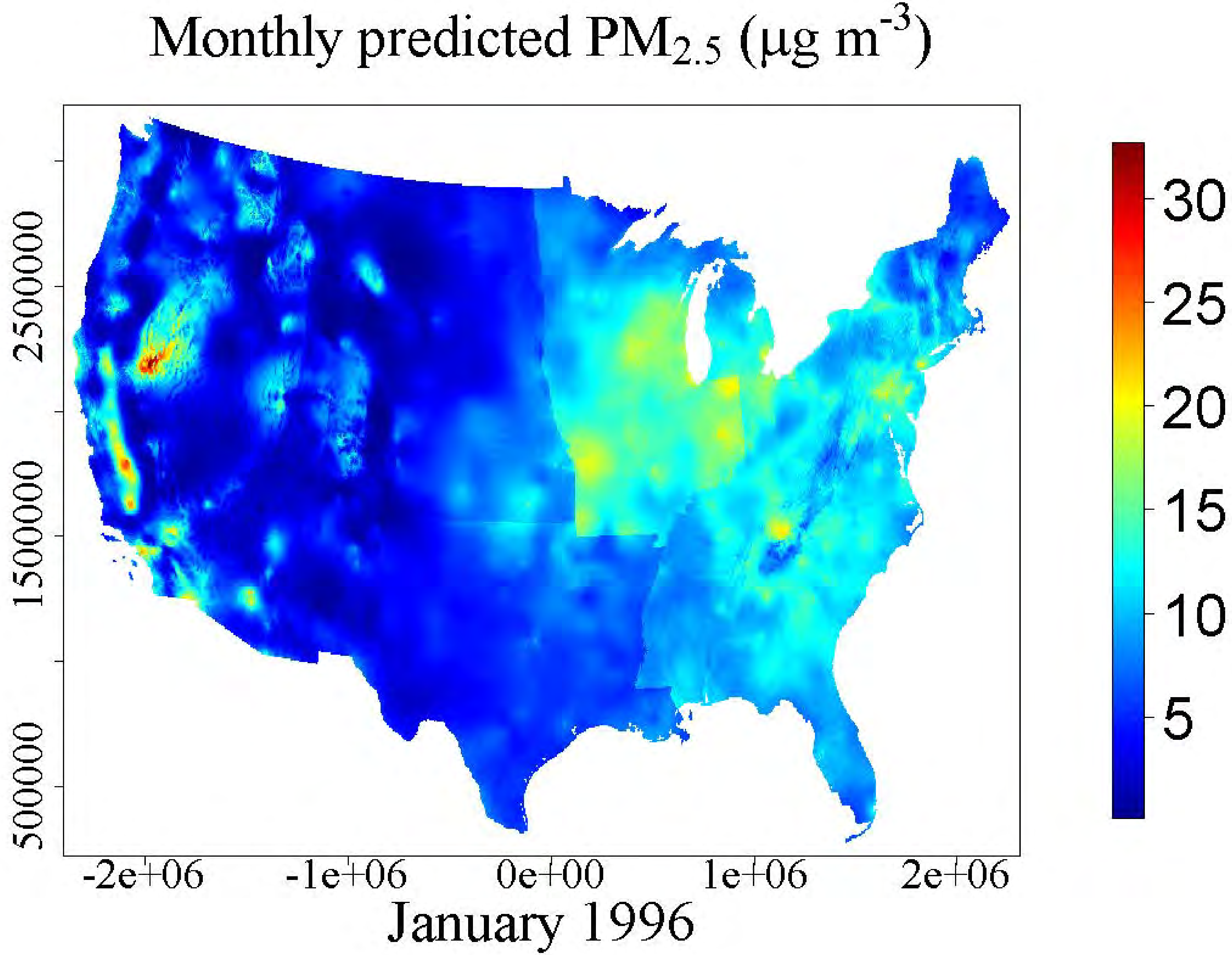
Monthly predicted PM2.5 (μg m-3)
25000000
15000000
5000000
-2e+06
-1e+06
0e+00
1e+06
2e+06
January 1996
30
25
20
15
10
5

Monthly predicted $PM_{2.5}$ ($\mu g\ m^{-3}$)

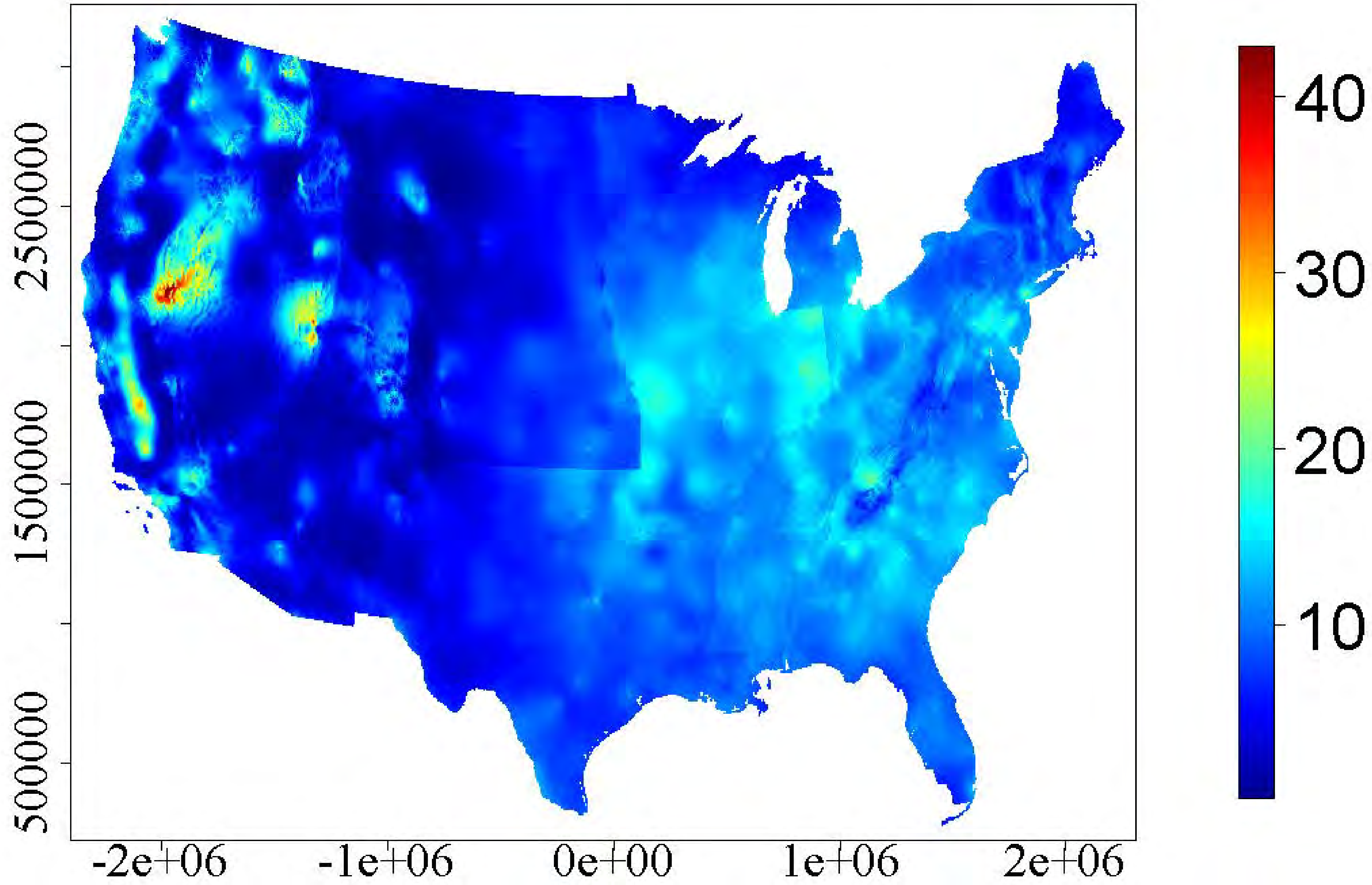

February 1996

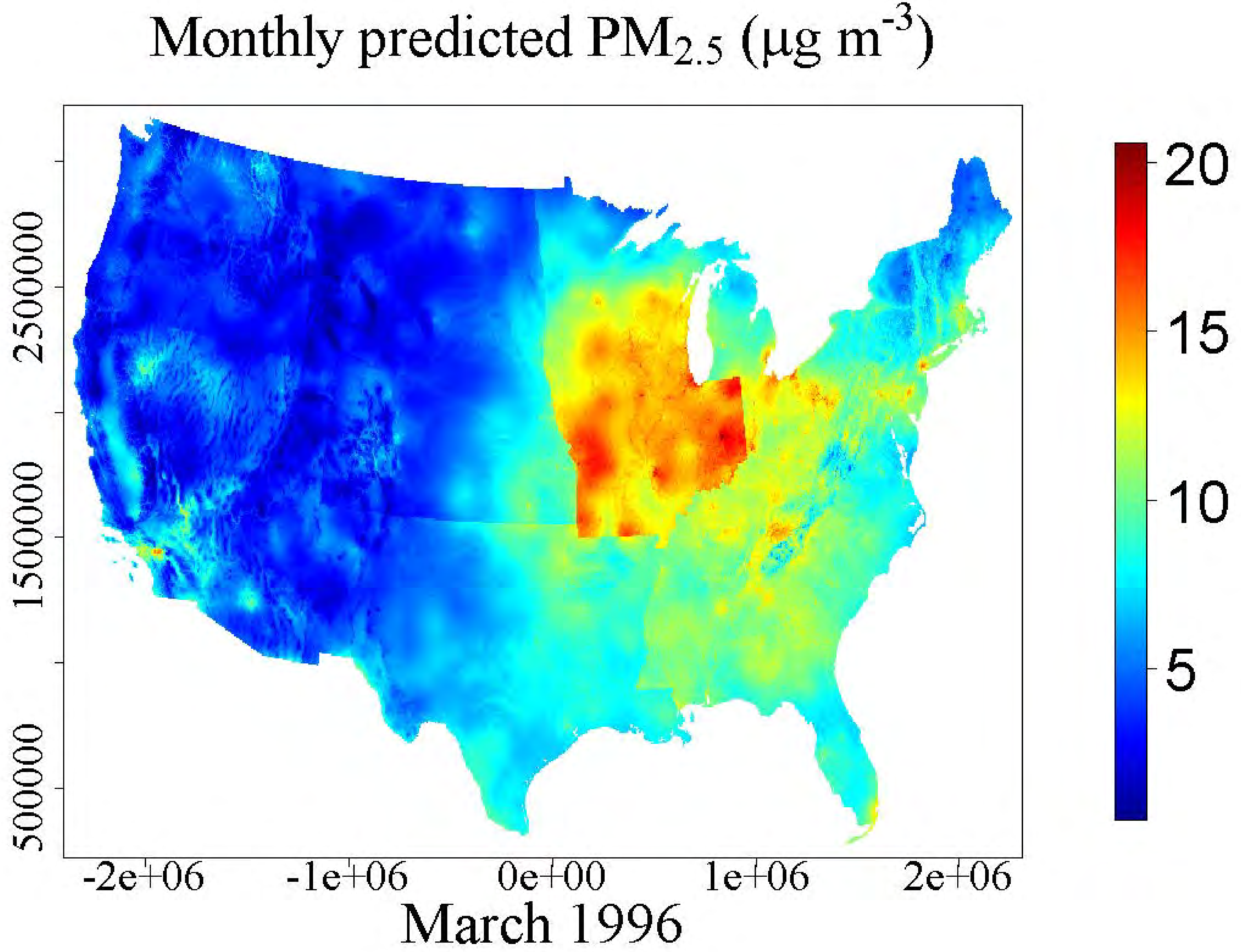
Monthly predicted PM2.5 (μg m-3)
25000000
15000000
5000000
-2e+06
-1e+06
0e+00
1e+06
2e+06
March 1996
20
15
10
5

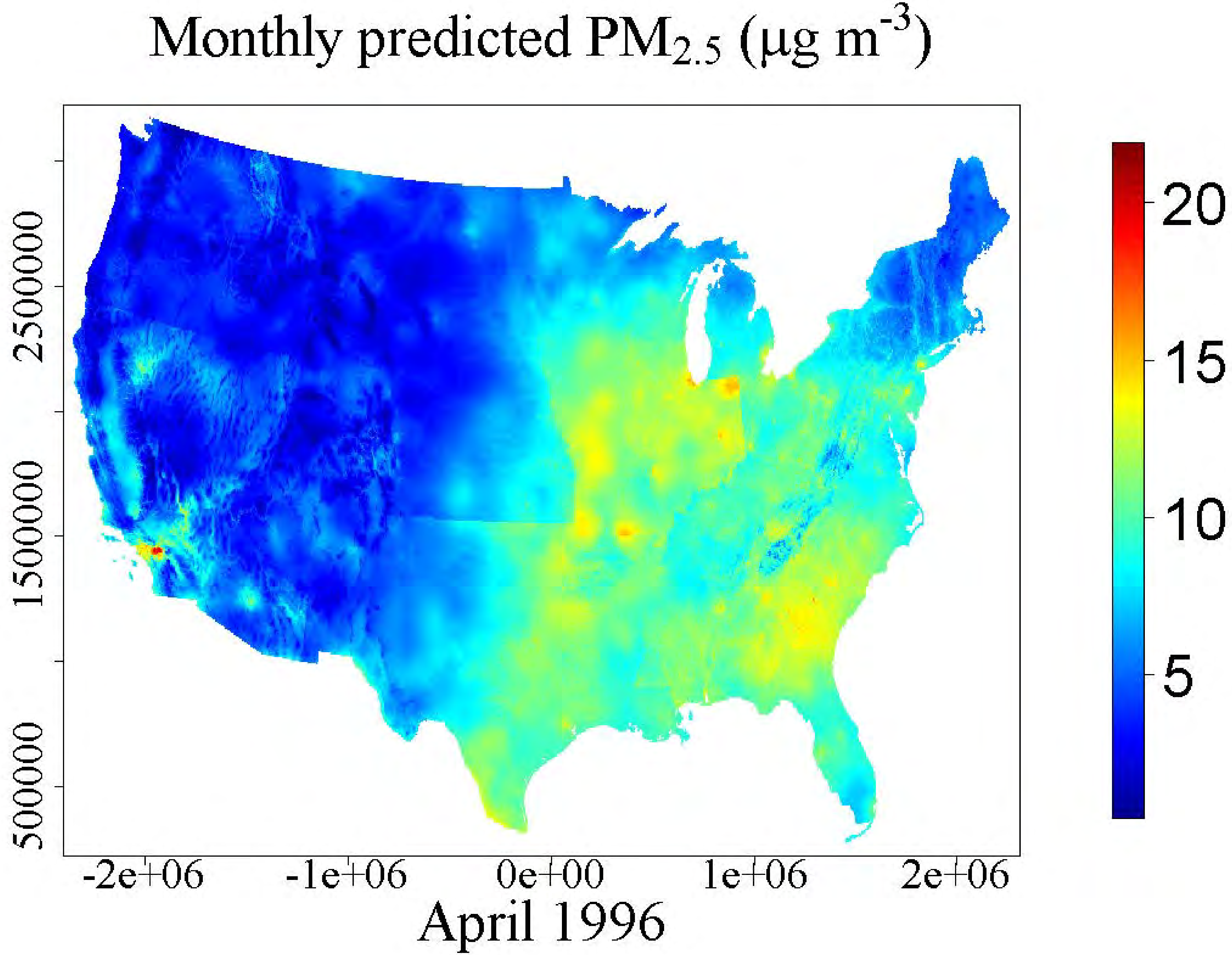
Monthly predicted PM2.5 (μg m-3)
25000000
15000000
5000000
-2e+06
-1e+06
0e+00
1e+06
2e+06
20
15
10
5
April 1996

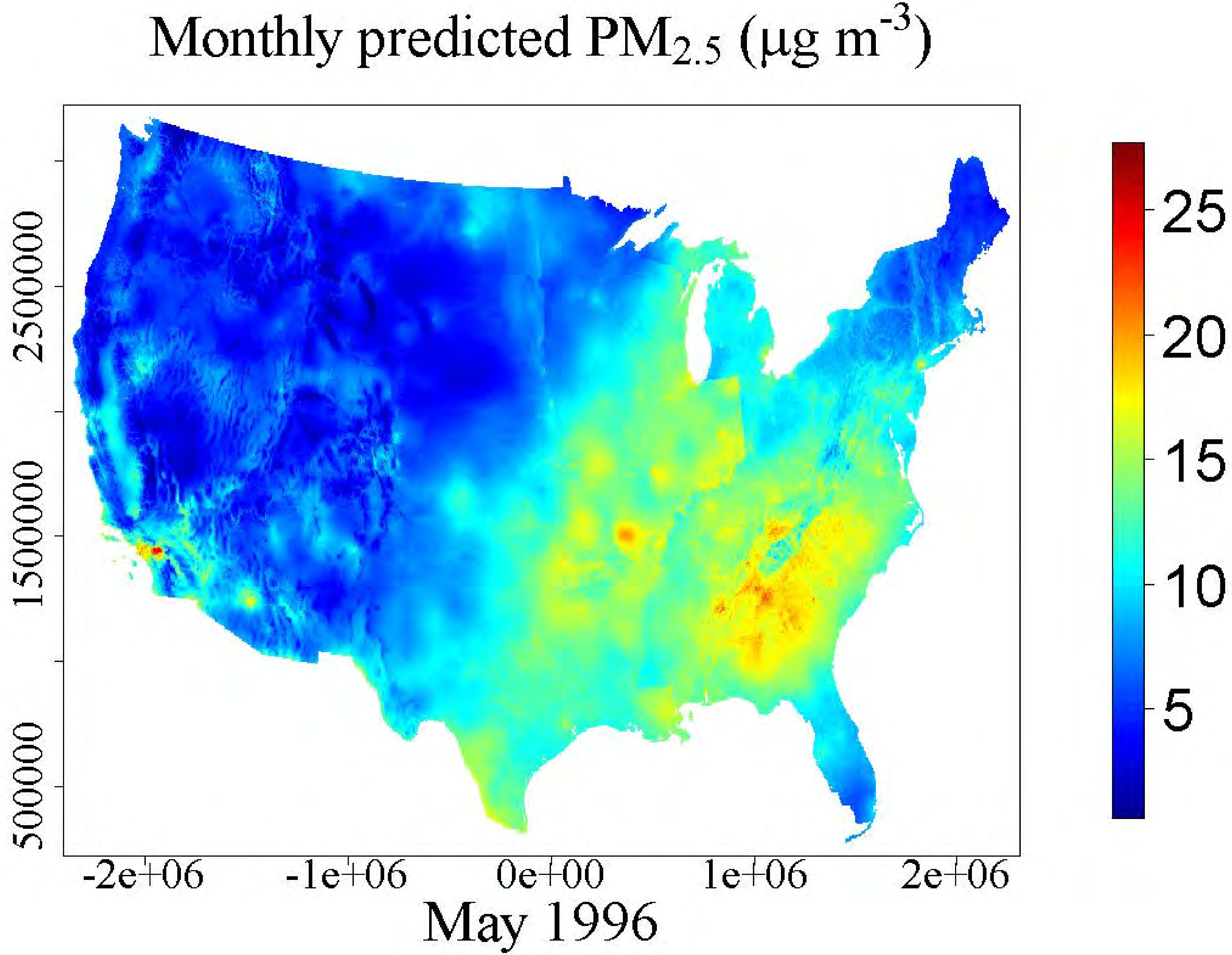
Monthly predicted PM2.5 (μg m-3)
25000000
15000000
5000000
-2e+06
-1e+06
0e+00
1e+06
2e+06
May 1996
25
20
15
10
5

Monthly predicted $PM_{2.5}$ ($\mu g\ m^{-3}$)

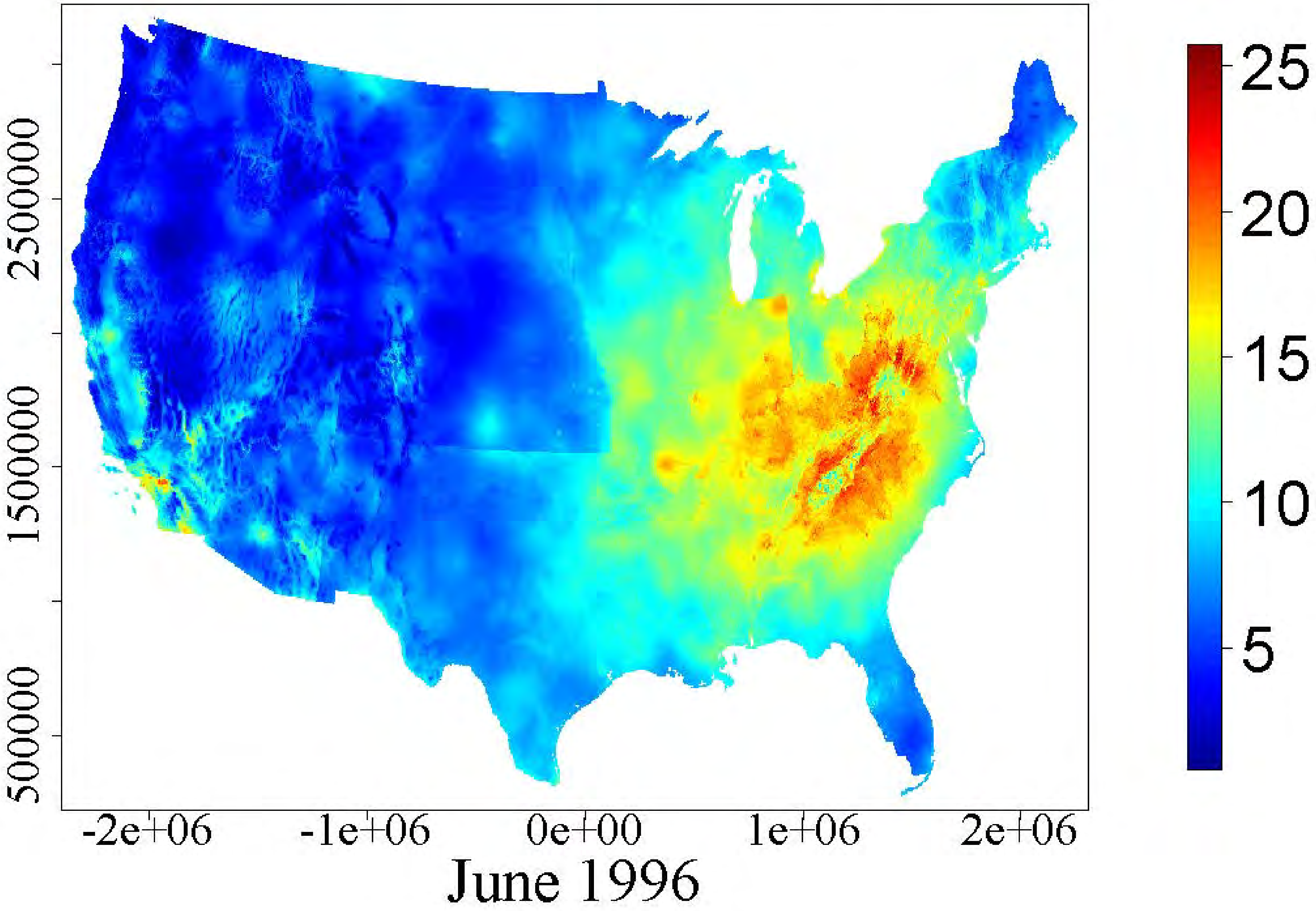

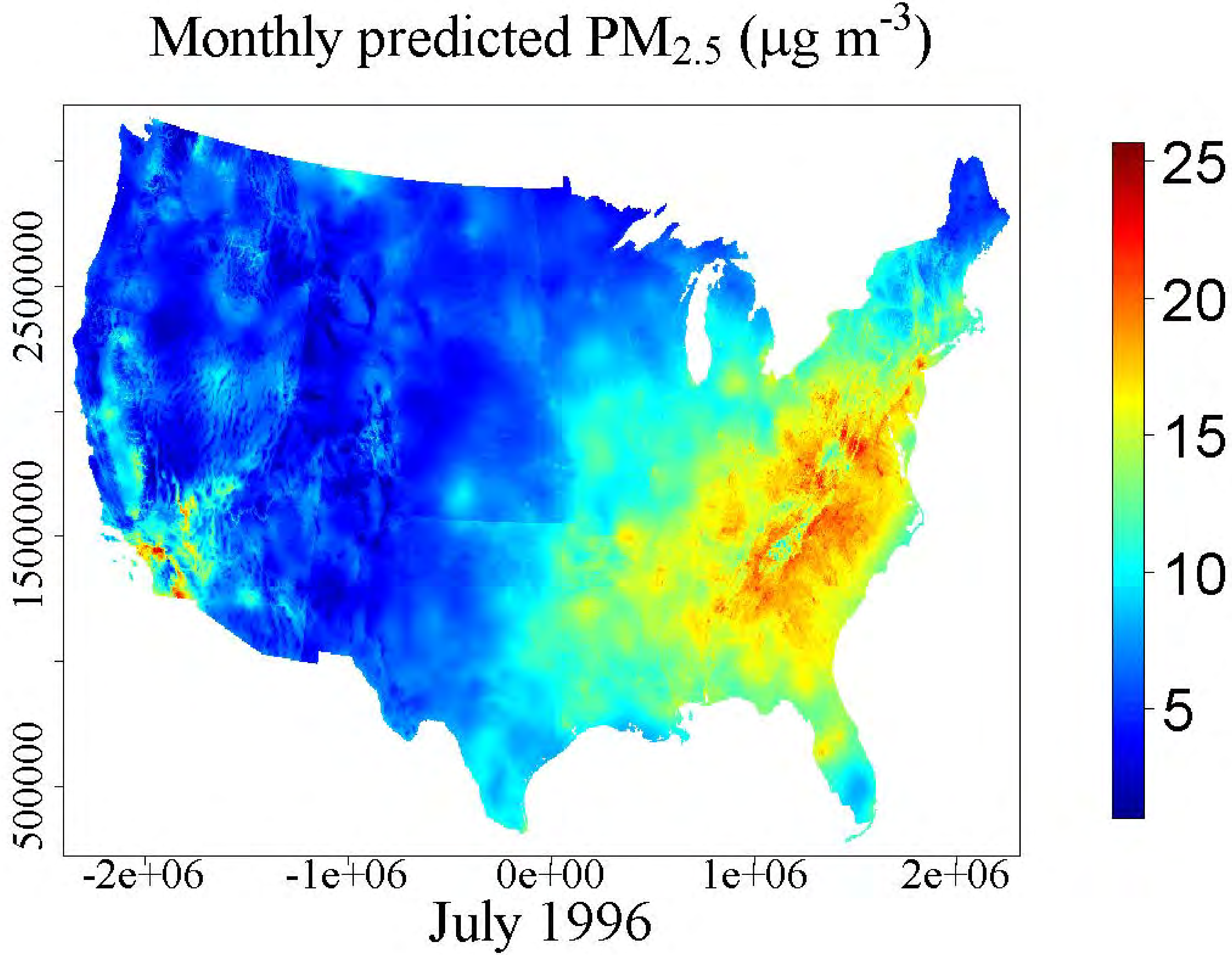
Monthly predicted PM$_{2.5}$ (μg m^{-3})
25
20
15
10
5
25000000
15000000
5000000
-2e+06
-1e+06
0e+00
1e+06
2e+06
July 1996

Monthly predicted $PM_{2.5}$ ($\mu g\ m^{-3}$)

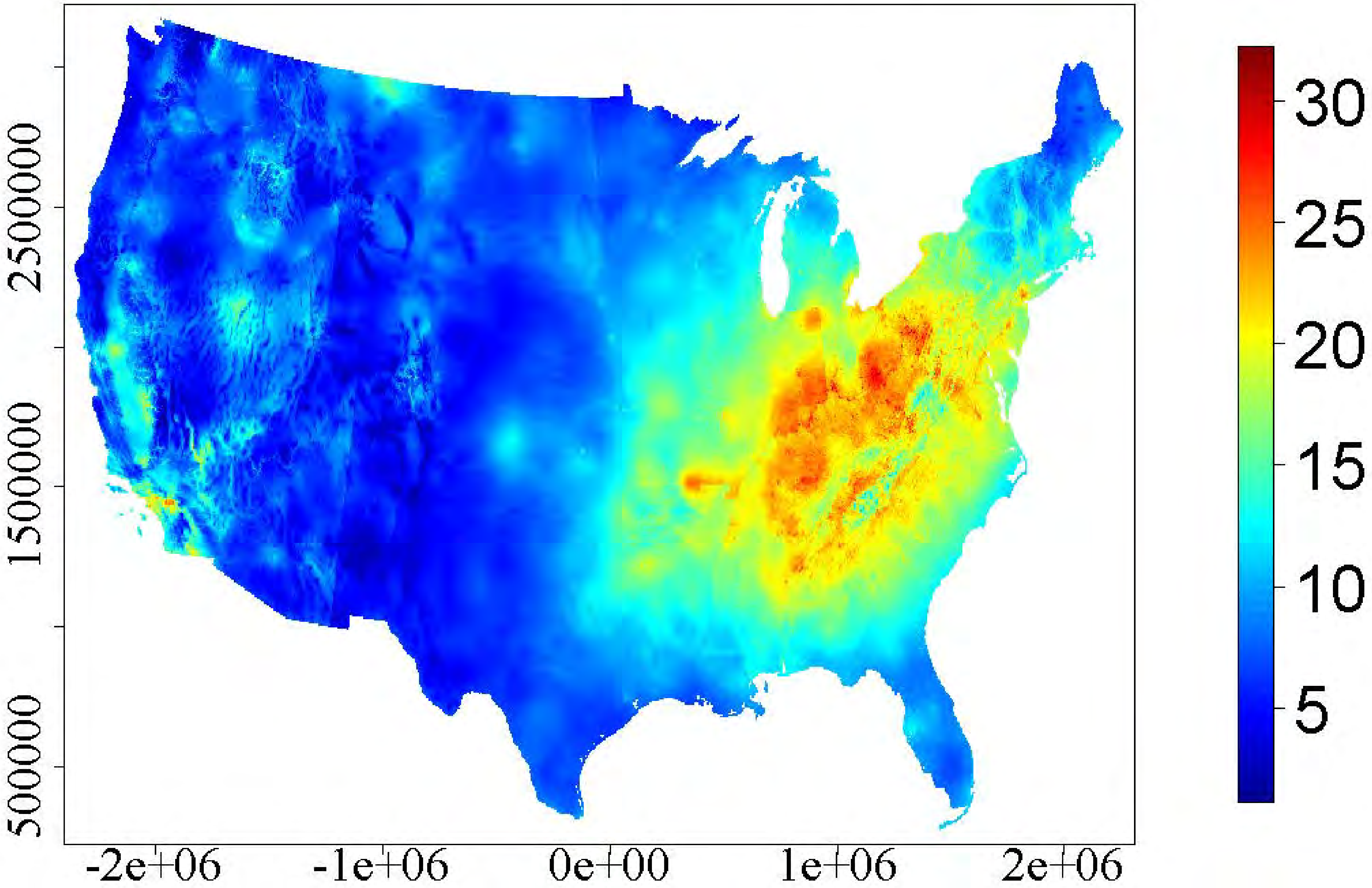

August 1996

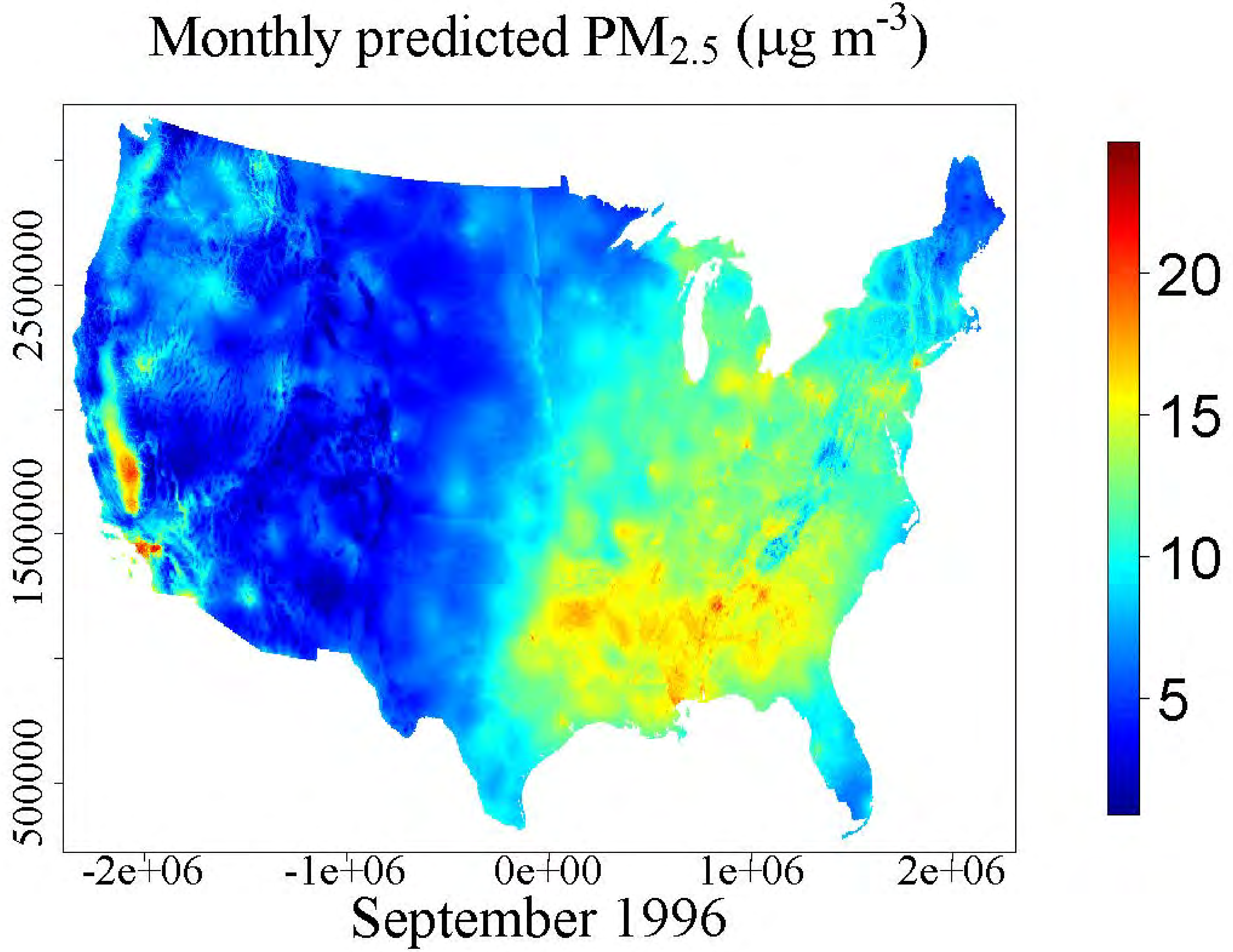
Monthly predicted $PM_{2.5}$ ($\mu g\ m^{-3}$)
25000000
15000000
5000000
-2e+06
-1e+06
0e+00
1e+06
2e+06
September 1996
20
15
10
5

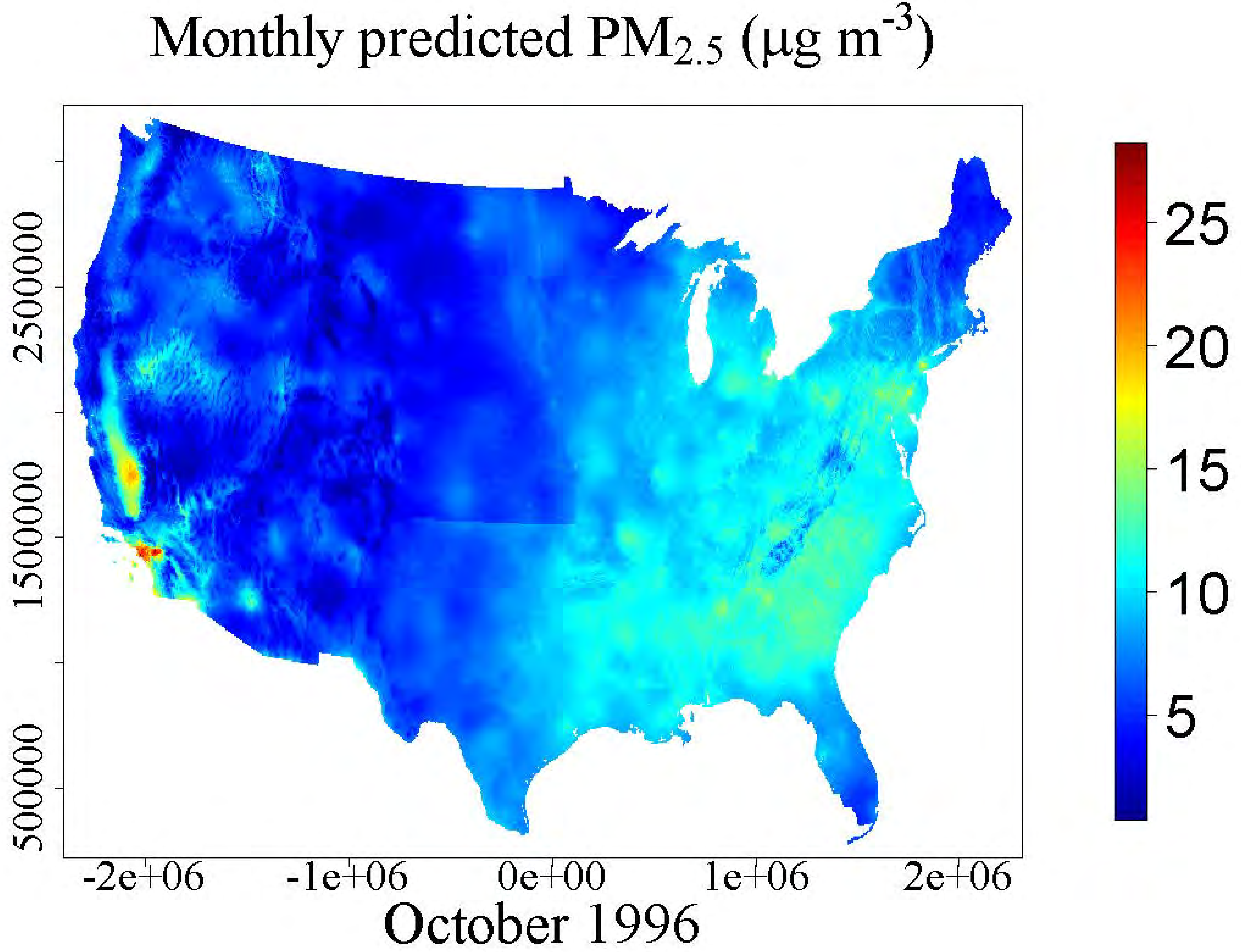
Monthly predicted $PM_{2.5}$ ($\mu g\ m^{-3}$)
25000000
15000000
5000000
-2e+06
-1e+06
0e+00
1e+06
2e+06
October 1996
25
20
15
10
5

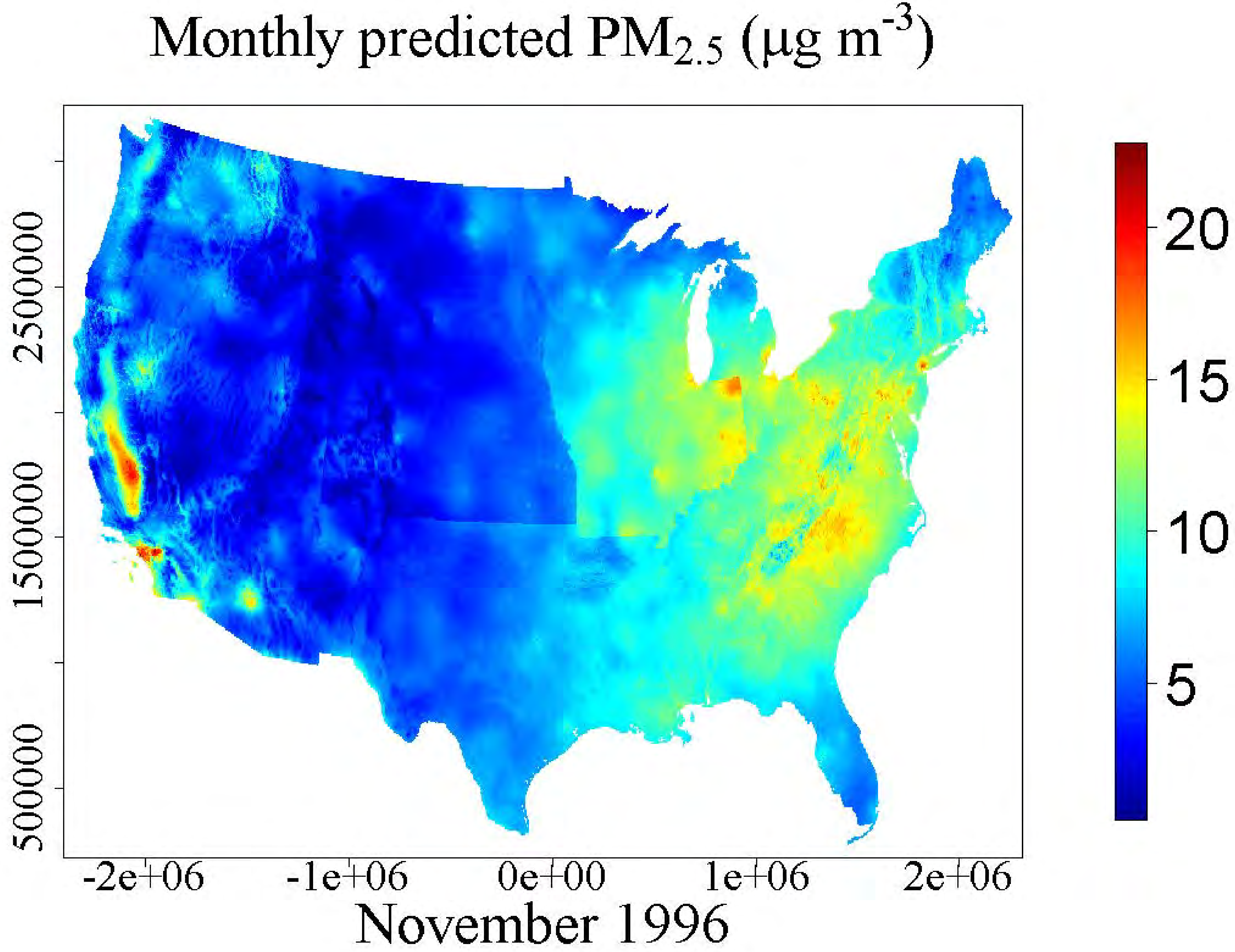
Monthly predicted $PM_{2.5}$ ($\mu g\ m^{-3}$)
25000000
15000000
5000000
-2e+06
-1e+06
0e+00
1e+06
2e+06
20
15
10
5
November 1996

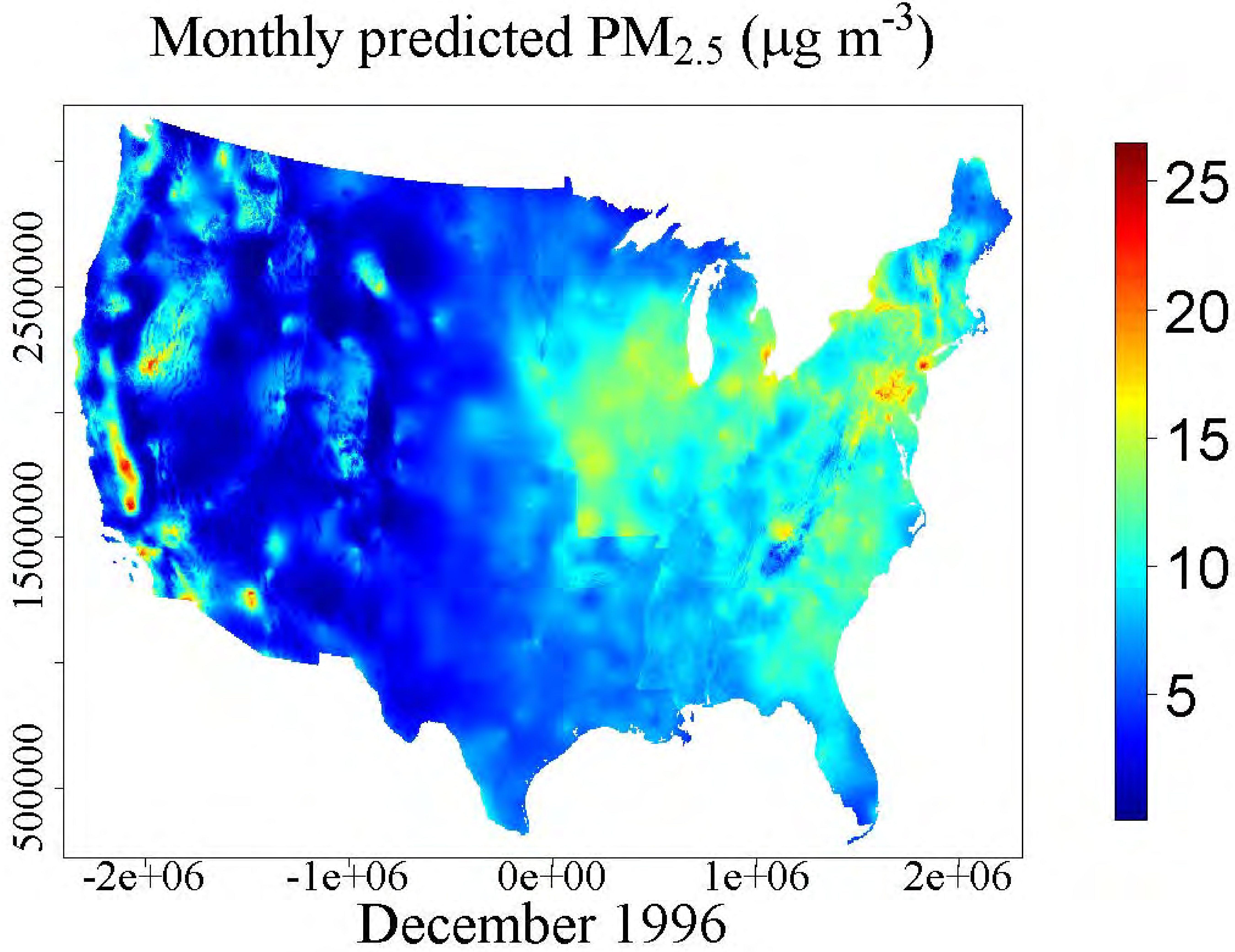
Monthly predicted PM2.5 (μg m-3)
25000000
15000000
5000000
-2e+06
-1e+06
0e+00
1e+06
2e+06
December 1996
25
20
15
10
5

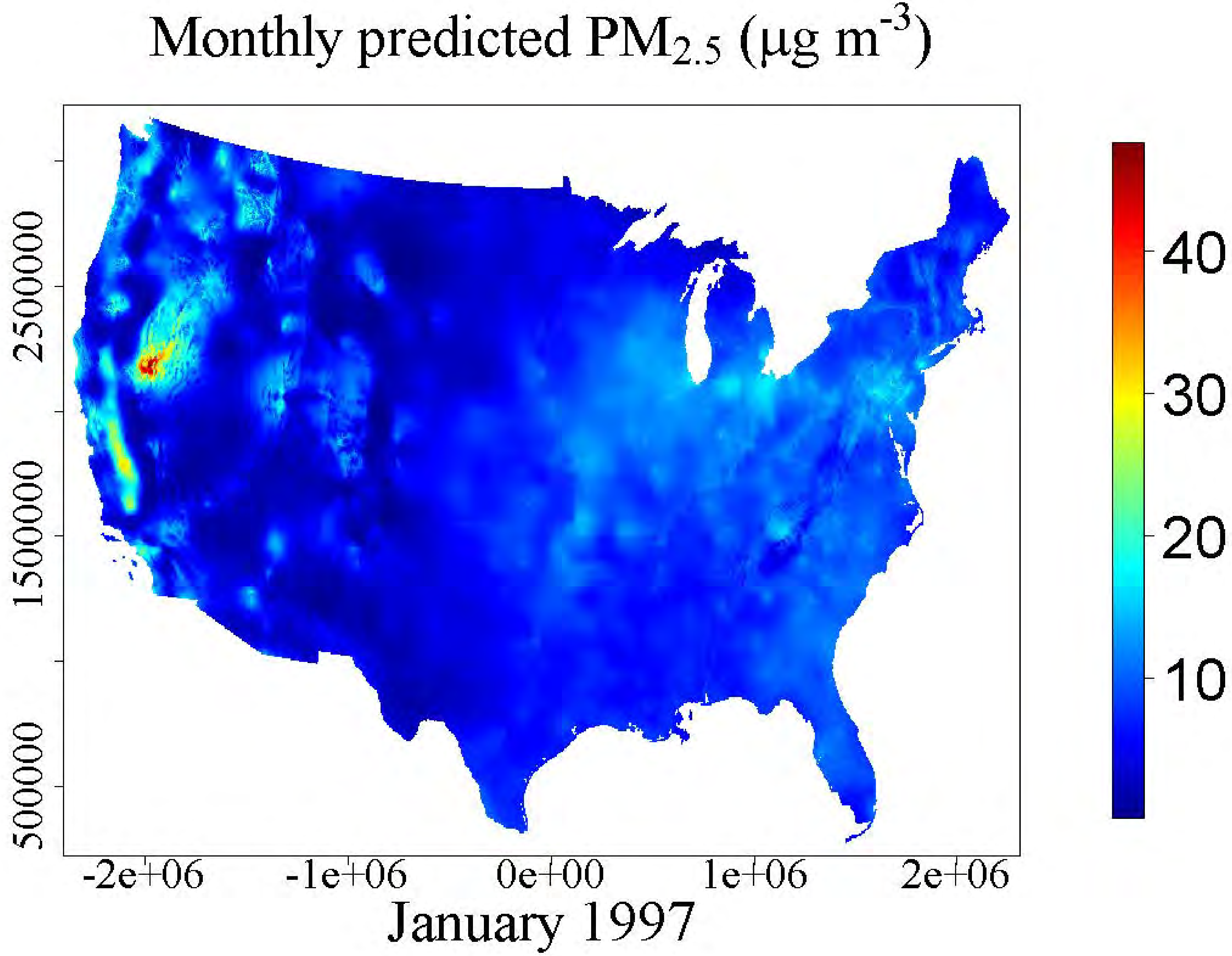
Monthly predicted PM2.5 (μg m-3)
25000000
15000000
5000000
-2e+06
-1e+06
0e+00
1e+06
2e+06
January 1997
40
30
20
10

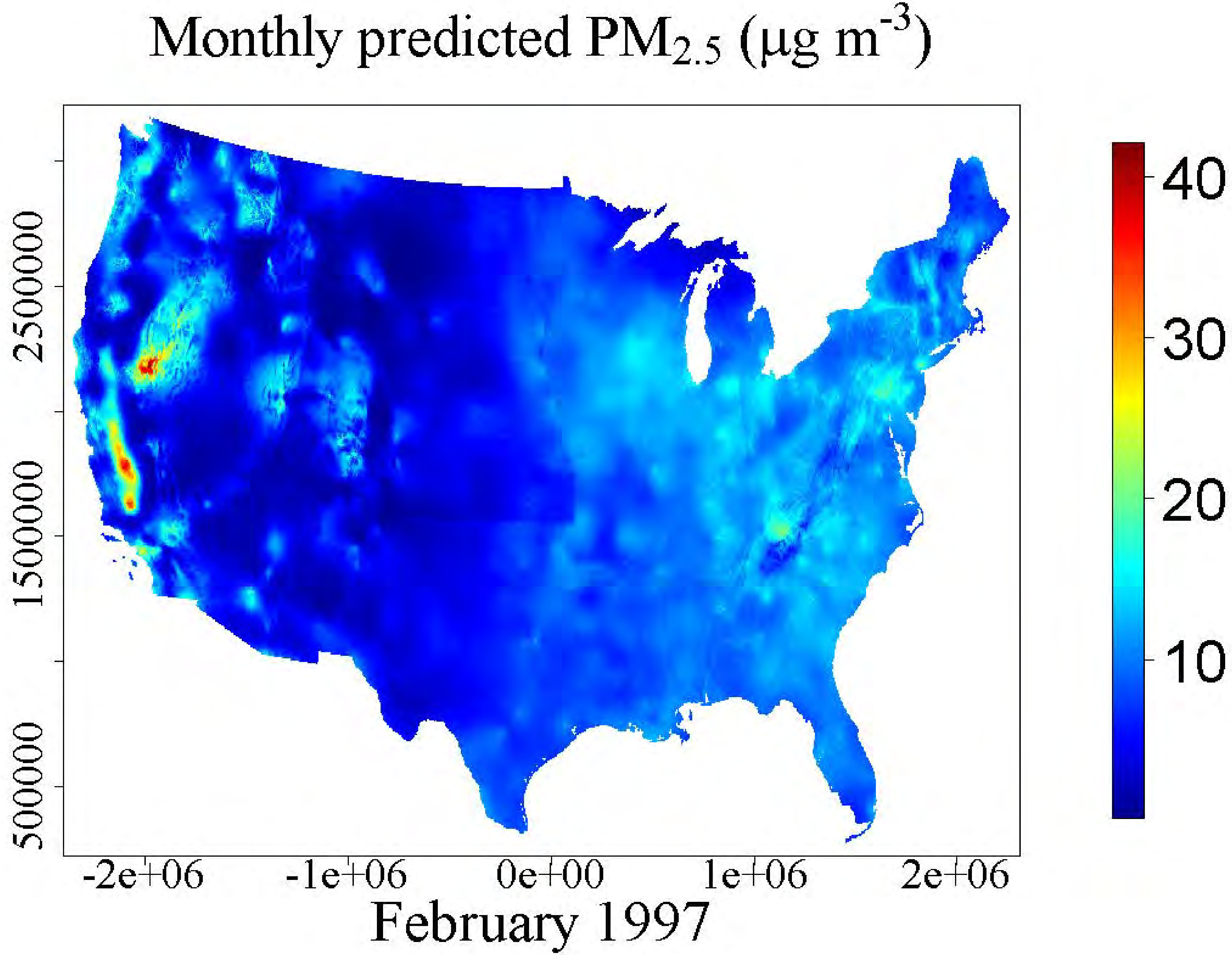
Monthly predicted PM2.5 (μg m-3)
25000000
15000000
5000000
-2e+06
-1e+06
0e+00
1e+06
2e+06
40
30
20
10
February 1997

Monthly predicted $PM_{2.5}$ ($\mu g\ m^{-3}$)

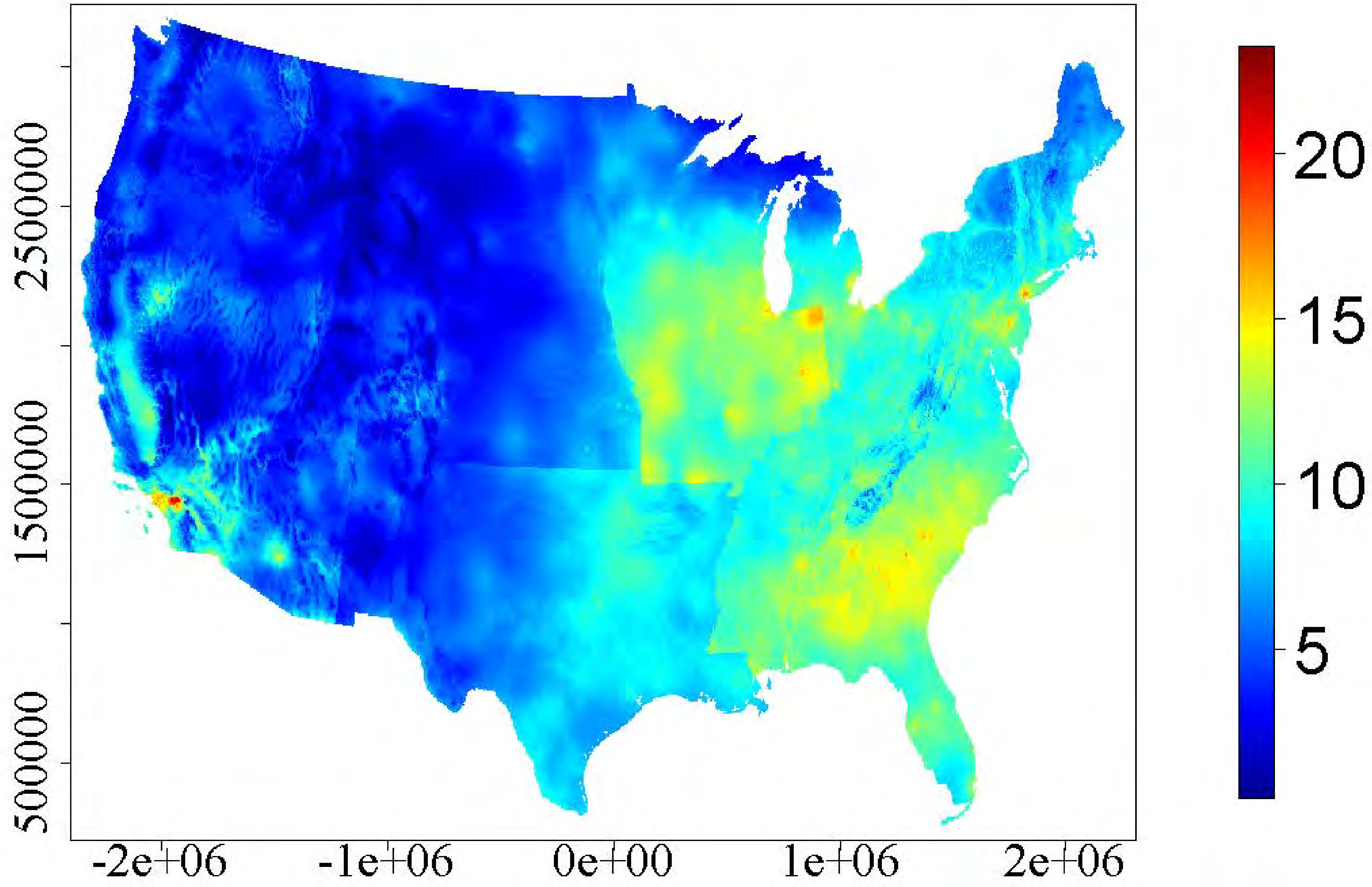

March 1997

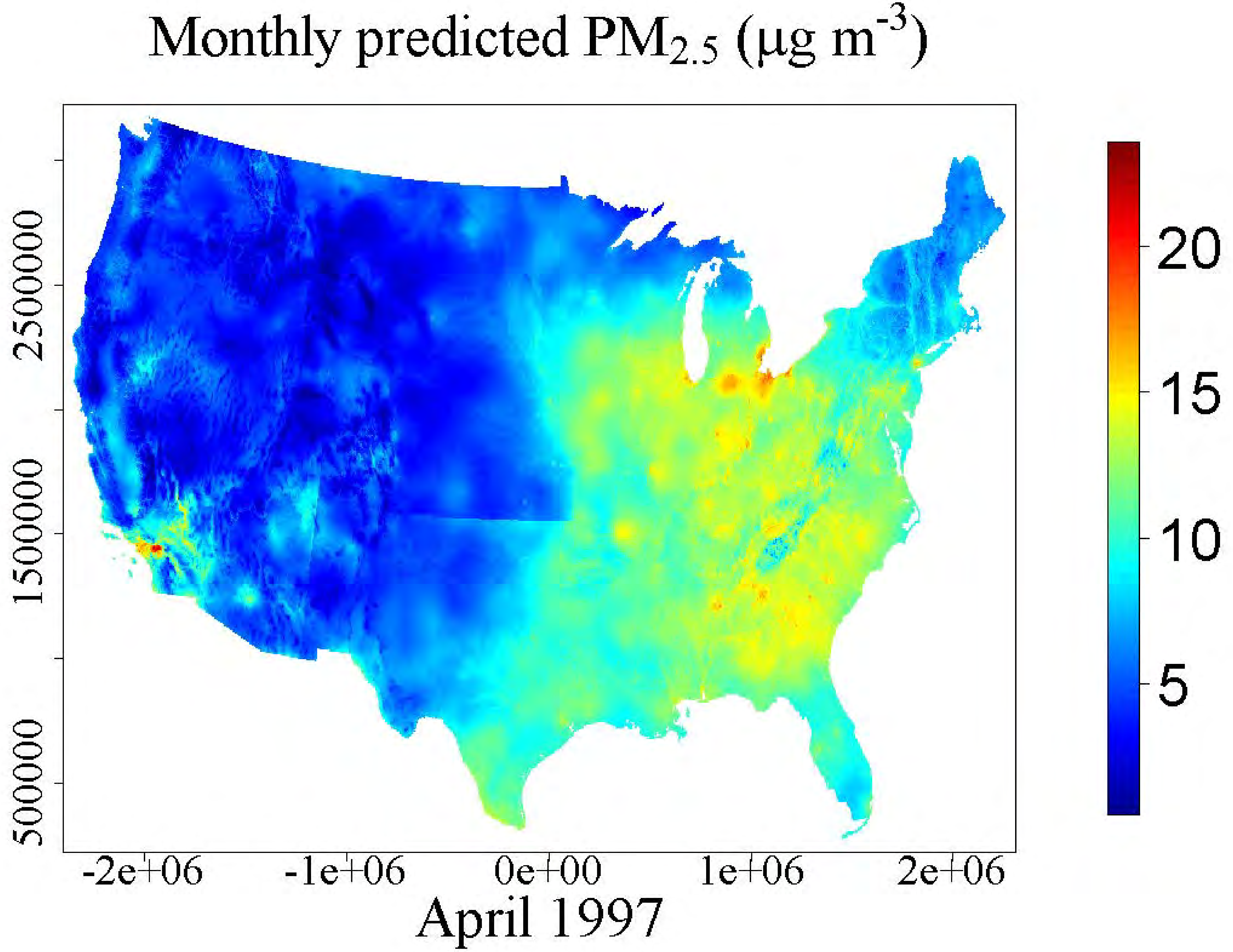

Monthly predicted PM2.5 (μg m-3)
25000000
15000000
5000000
-2e+06
-1e+06
0e+00
1e+06
2e+06
April 1997
20
15
10
5

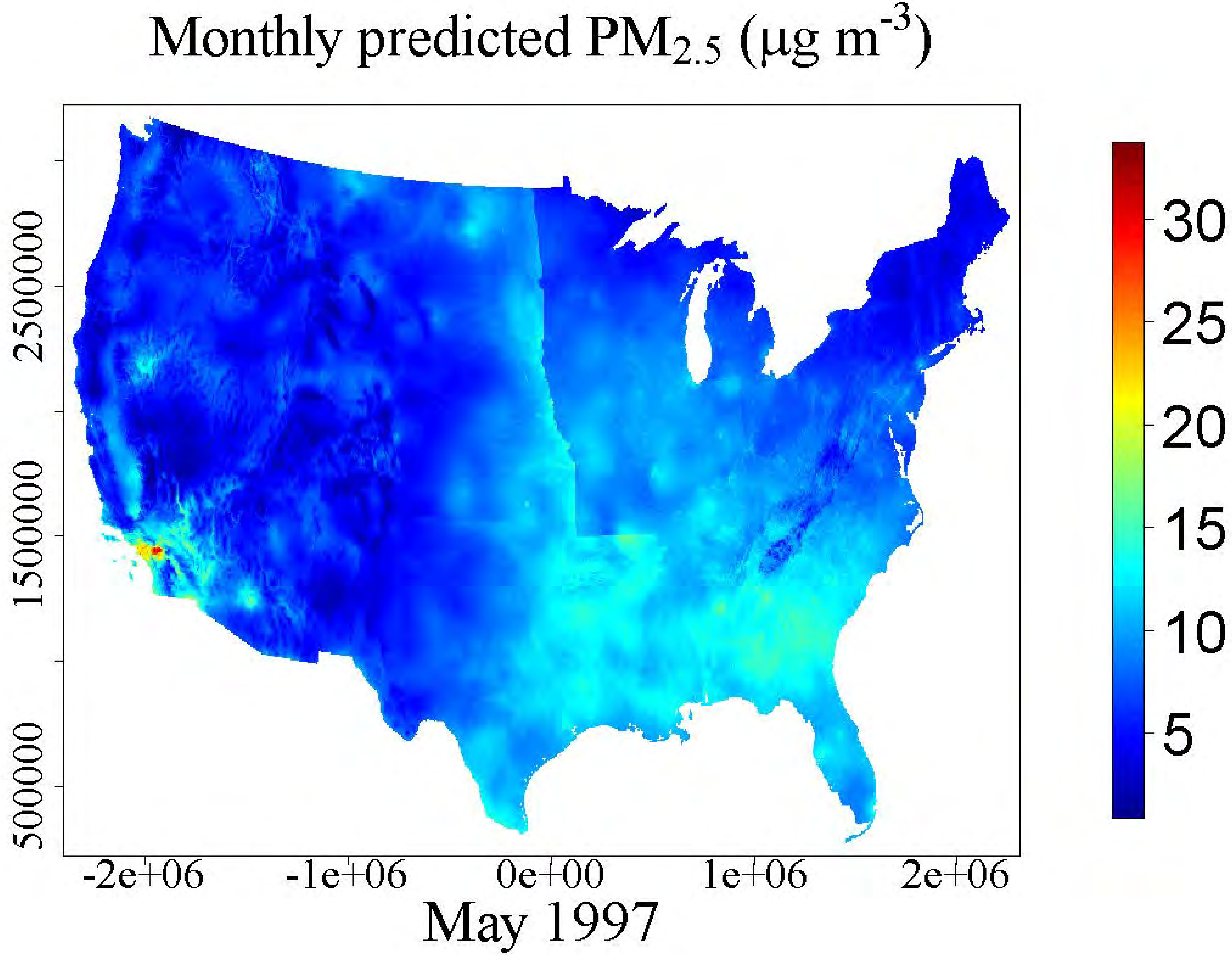
Monthly predicted PM2.5 (μg m-3)
25000000
15000000
5000000
-2e+06
-1e+06
0e+00
1e+06
2e+06
May 1997
30
25
20
15
10
5

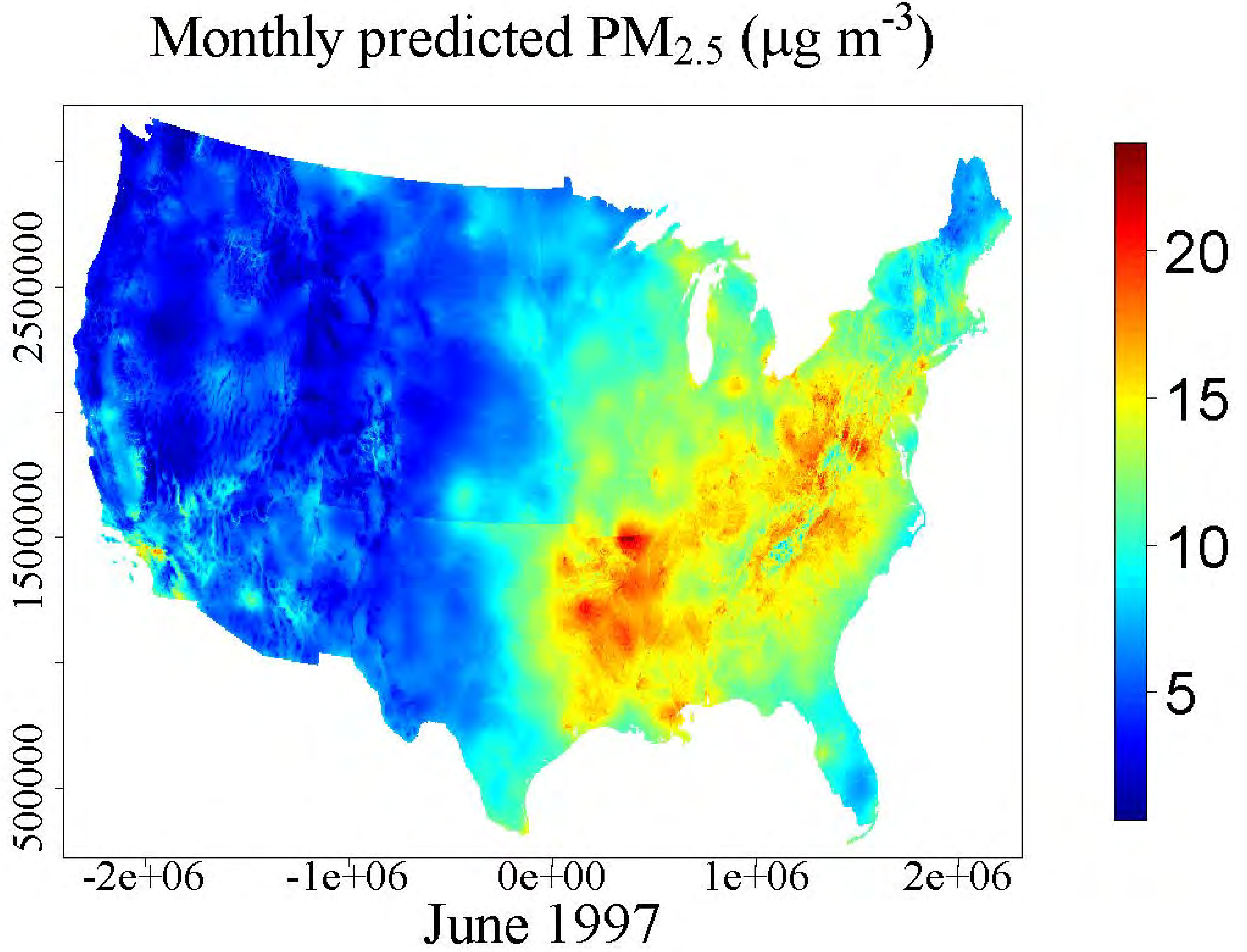
Monthly predicted PM2.5 (μg m-3)
25000000
15000000
5000000
-2e+06
-1e+06
0e+00
1e+06
2e+06
June 1997
20
15
10
5

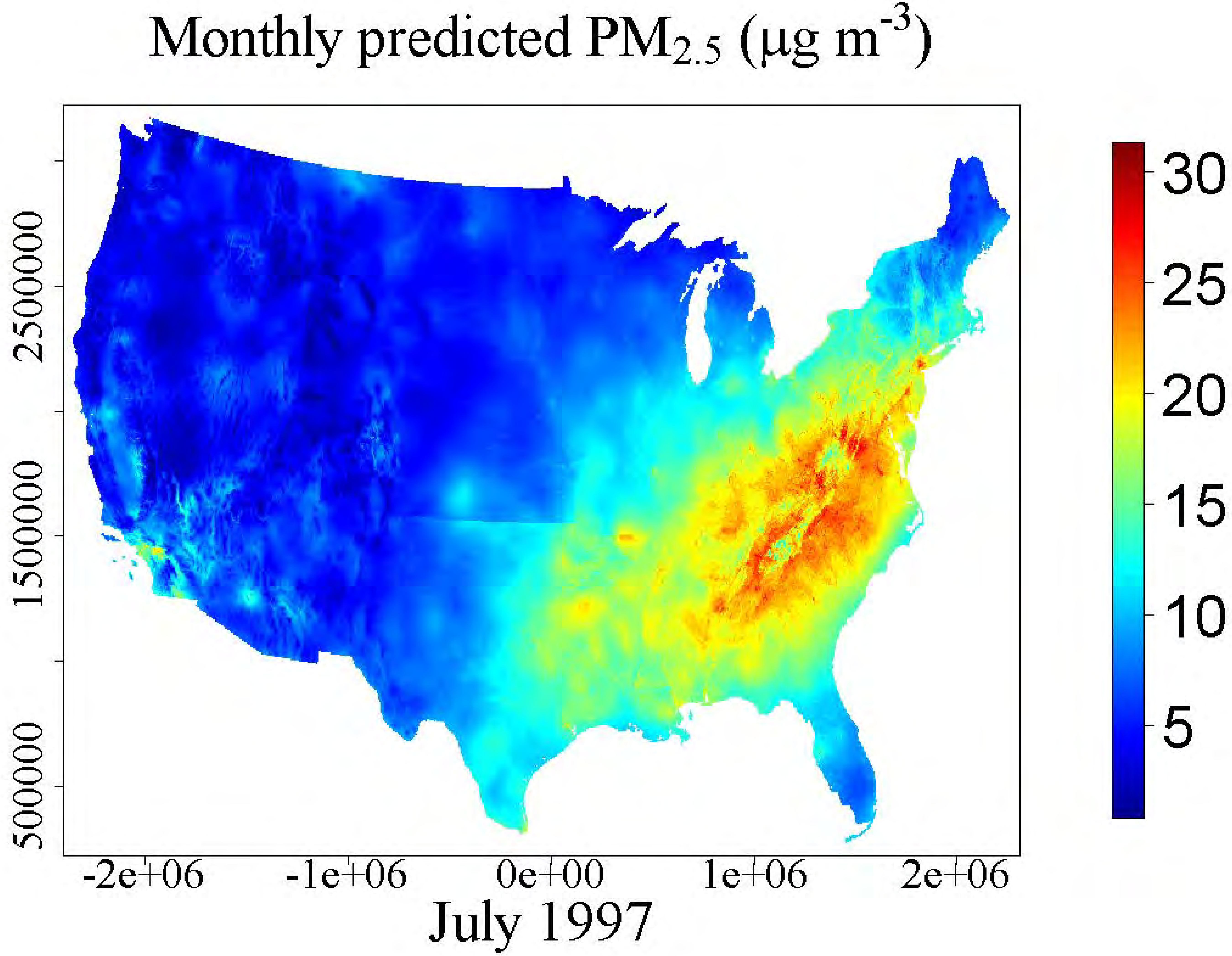

Monthly predicted PM2.5 (μg m-3)
25000000
15000000
5000000
-2e+06
-1e+06
0e+00
1e+06
2e+06
July 1997
30
25
20
15
10
5

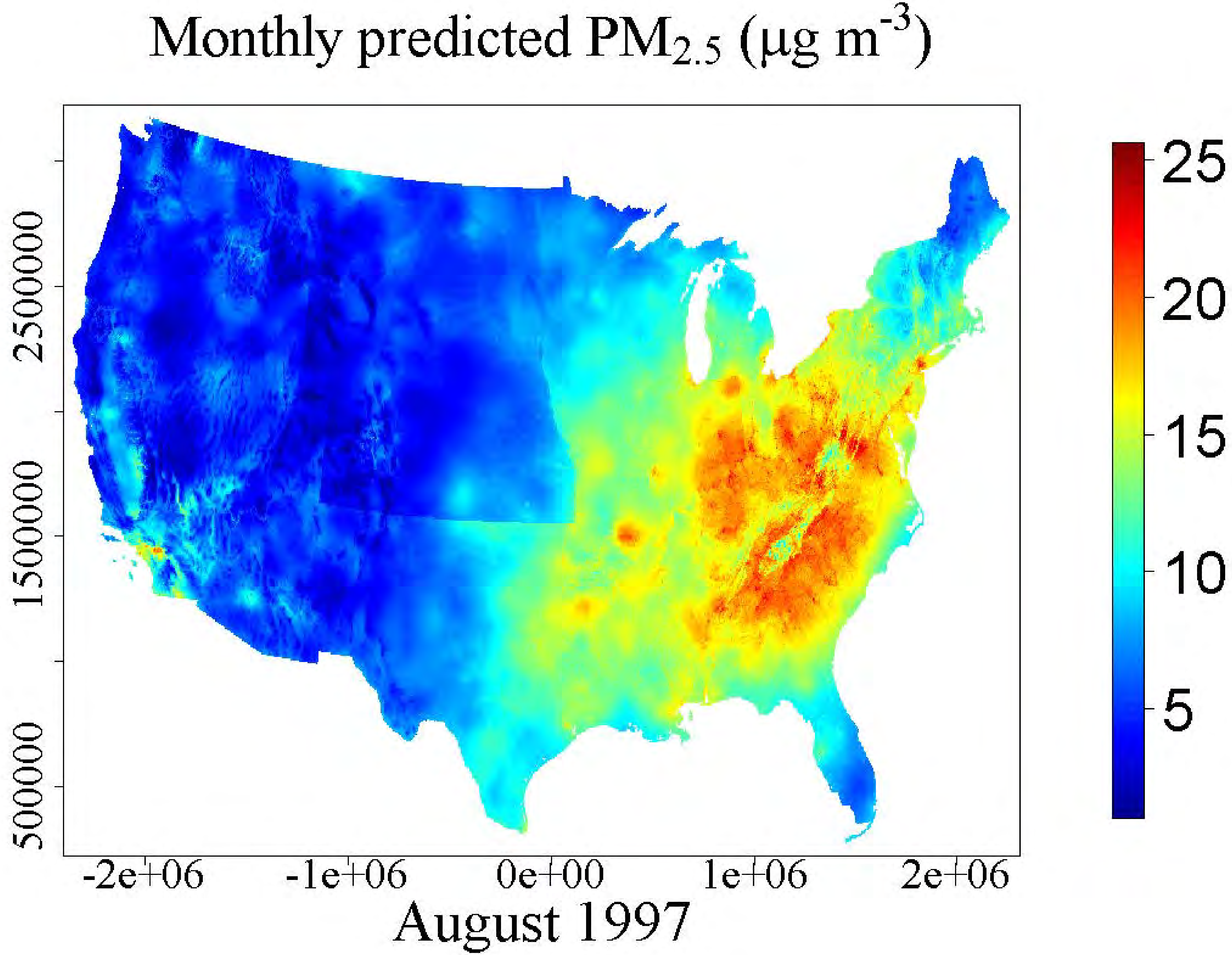

Monthly predicted PM2.5 (μg m-3)
25
20
15
10
5
25000000
15000000
5000000
-2e+06
-1e+06
0e+00
1e+06
2e+06
August 1997

Monthly predicted $PM_{2.5}$ ($\mu g\ m^{-3}$)

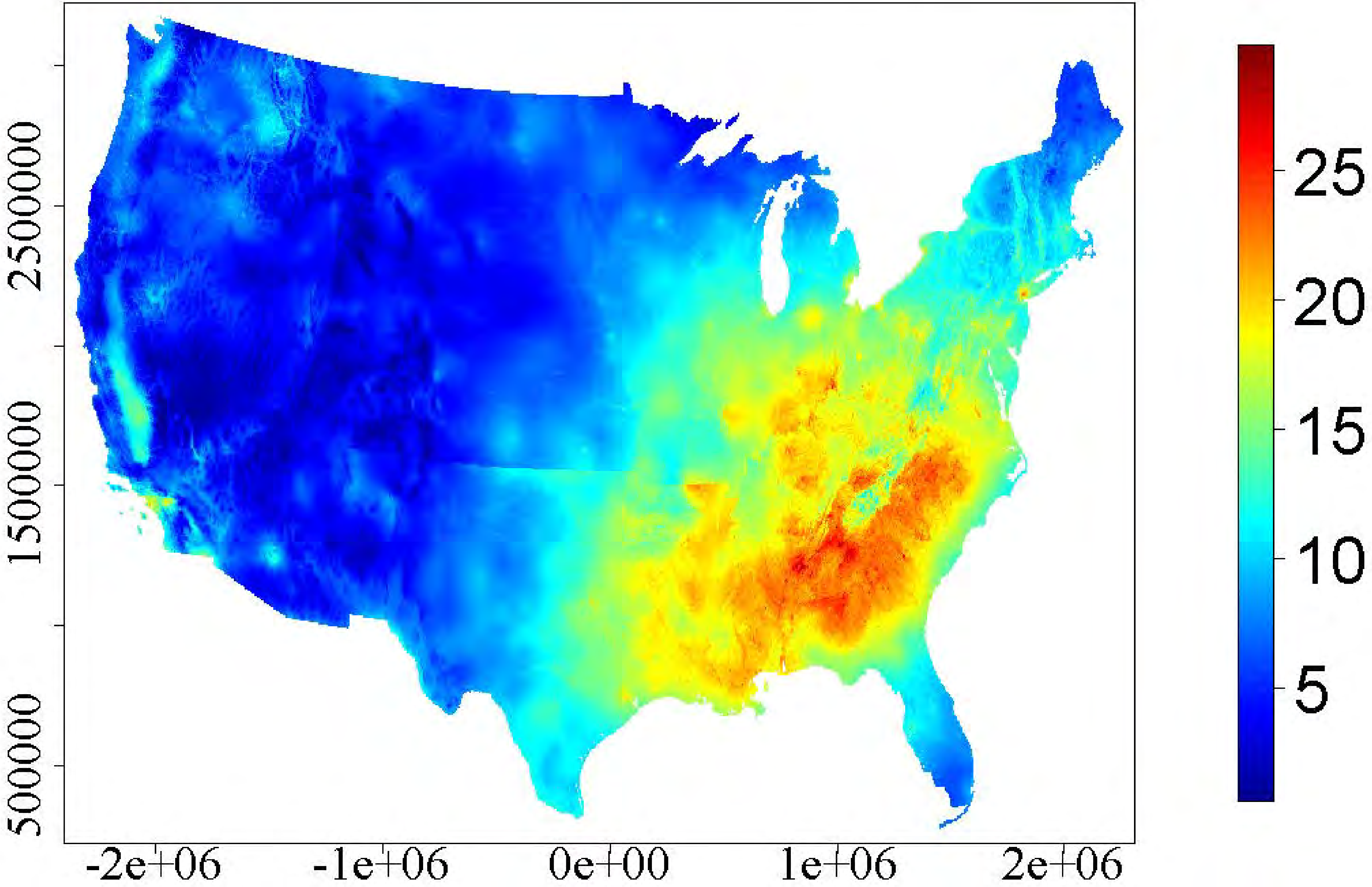

September 1997

Monthly predicted $PM_{2.5}$ ($\mu g\ m^{-3}$)

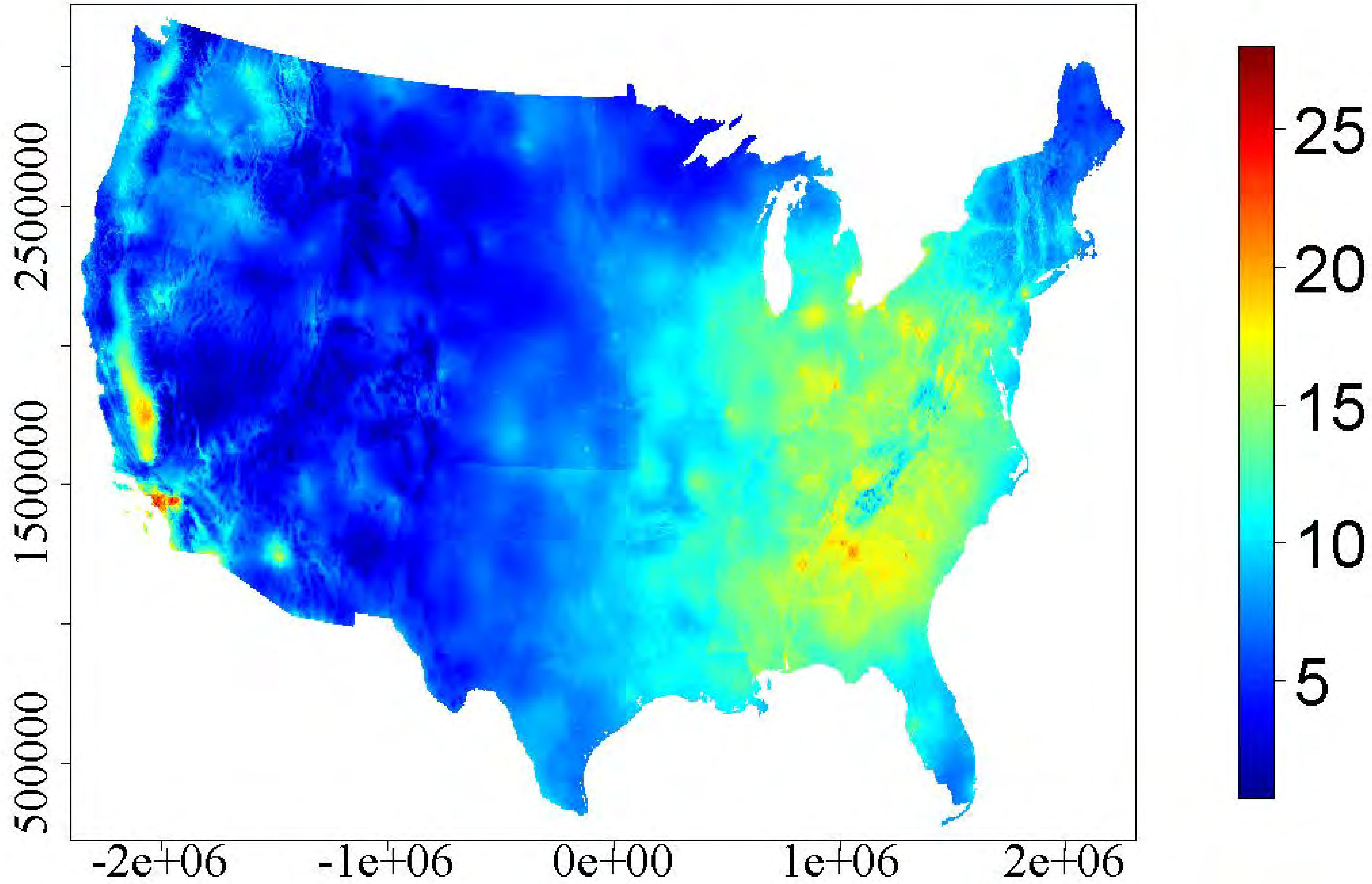

October 1997

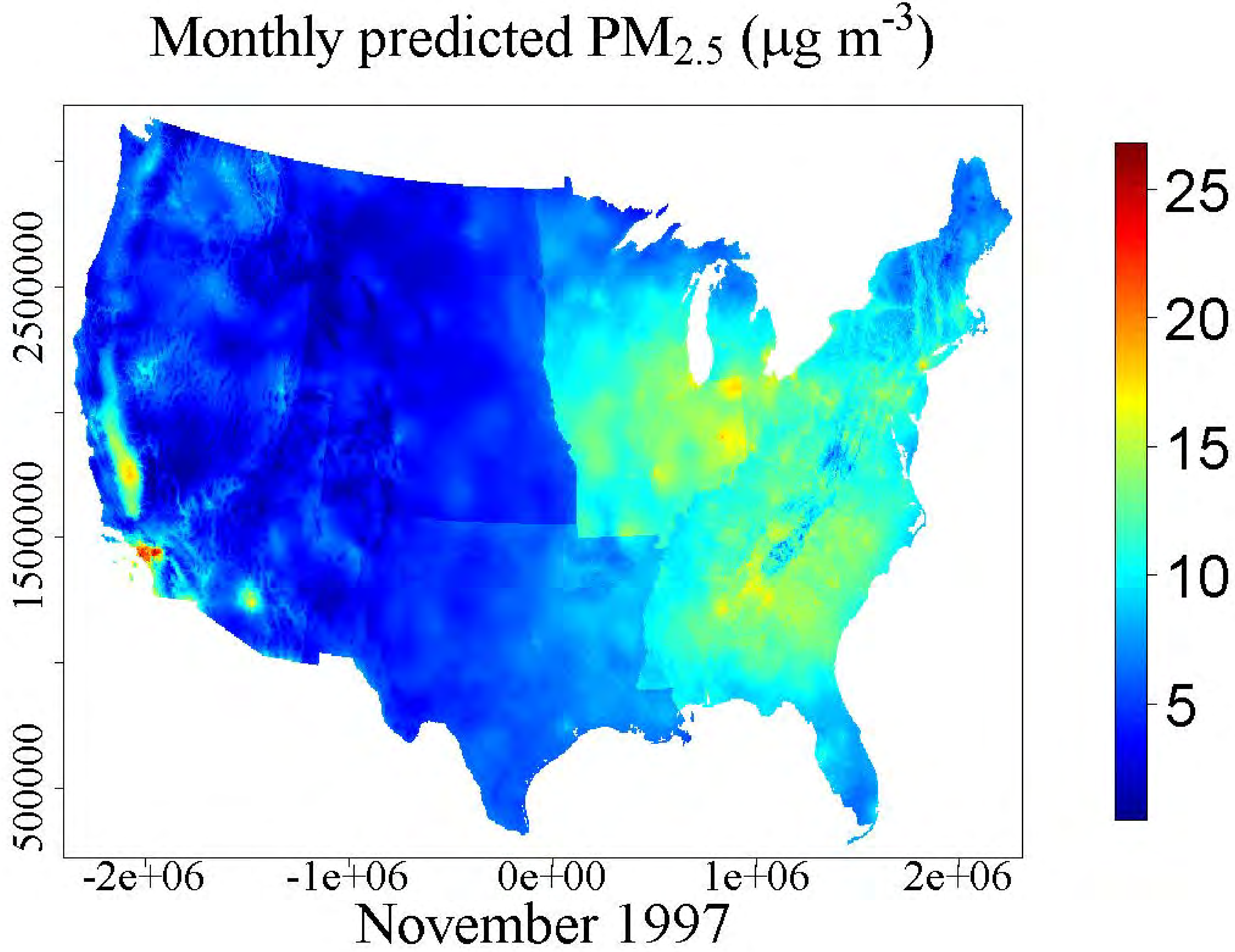
Monthly predicted PM2.5 (μg m-3)
25000000
15000000
5000000
-2e+06
-1e+06
0e+00
1e+06
2e+06
November 1997
25
20
15
10
5

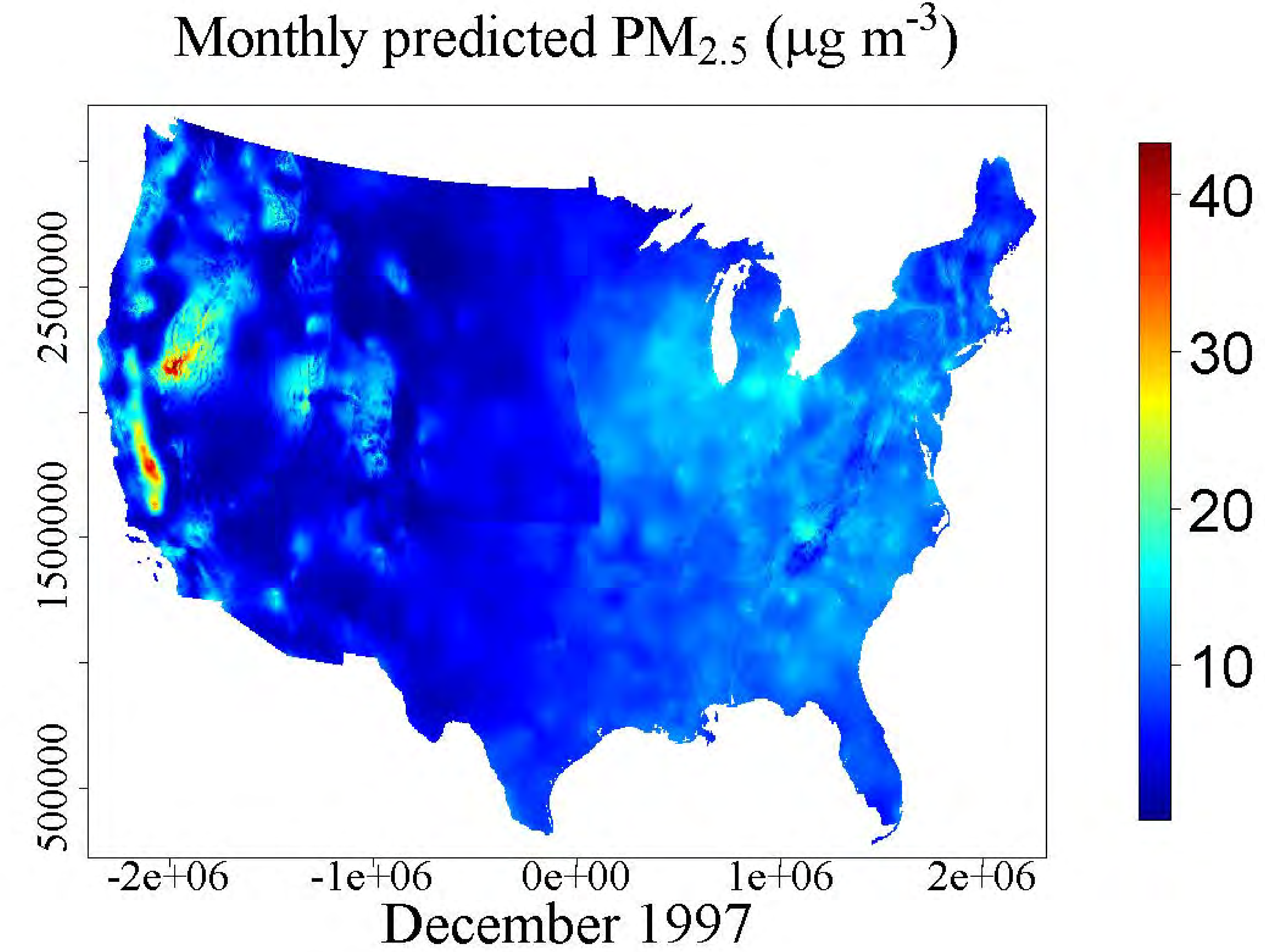

Monthly predicted PM2.5 (μg m-3)
25000000
15000000
5000000
-2e+06
-1e+06
0e+00
1e+06
2e+06
December 1997
40
30
20
10

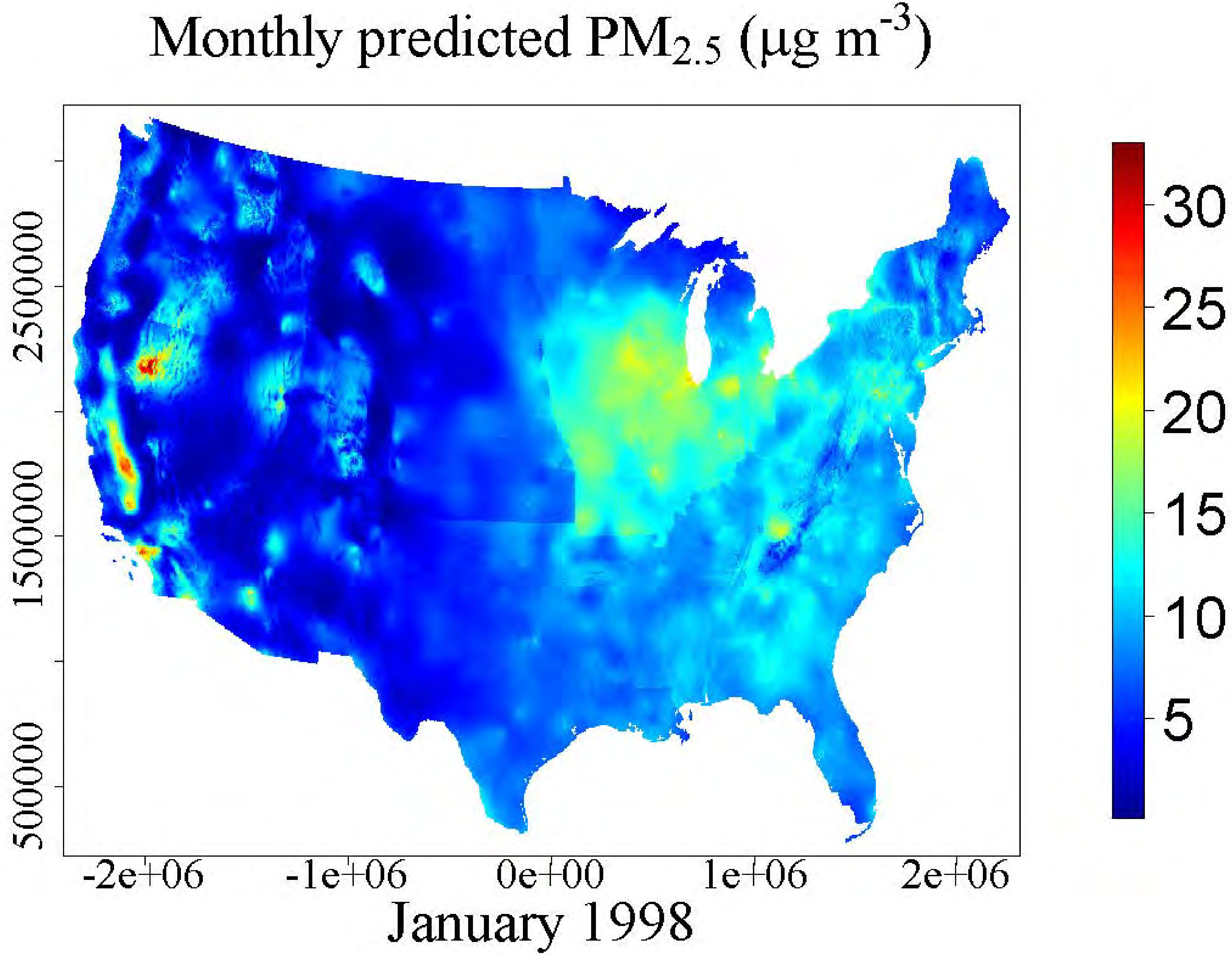
Monthly predicted PM2.5 (μg m-3)
25000000
15000000
5000000
-2e+06
-1e+06
0e+00
1e+06
2e+06
January 1998
30
25
20
15
10
5

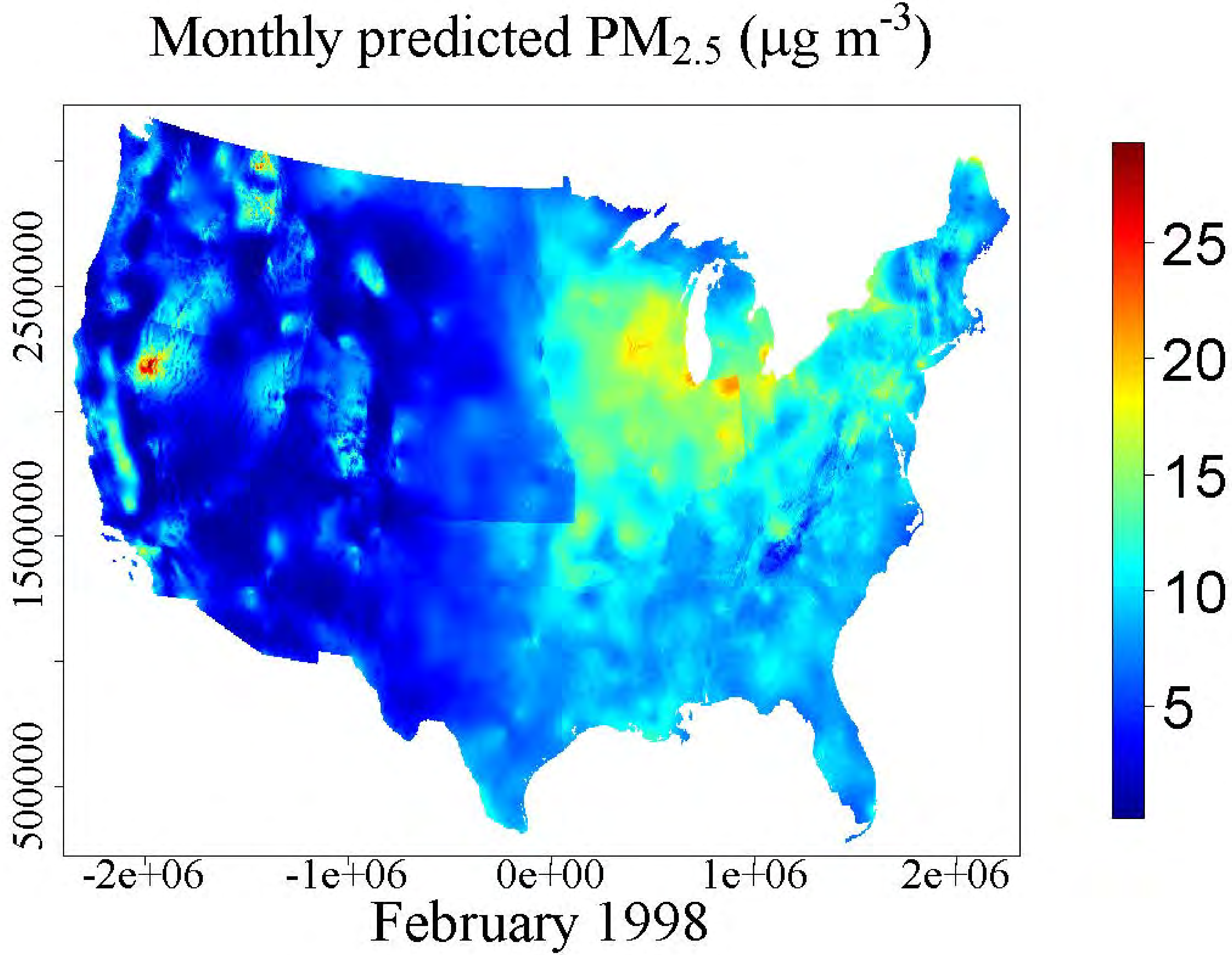
Monthly predicted $PM_{2.5}$ ($\mu g\ m^{-3}$)
25000000
15000000
5000000
-2e+06
-1e+06
0e+00
1e+06
2e+06
February 1998
25
20
15
10
5

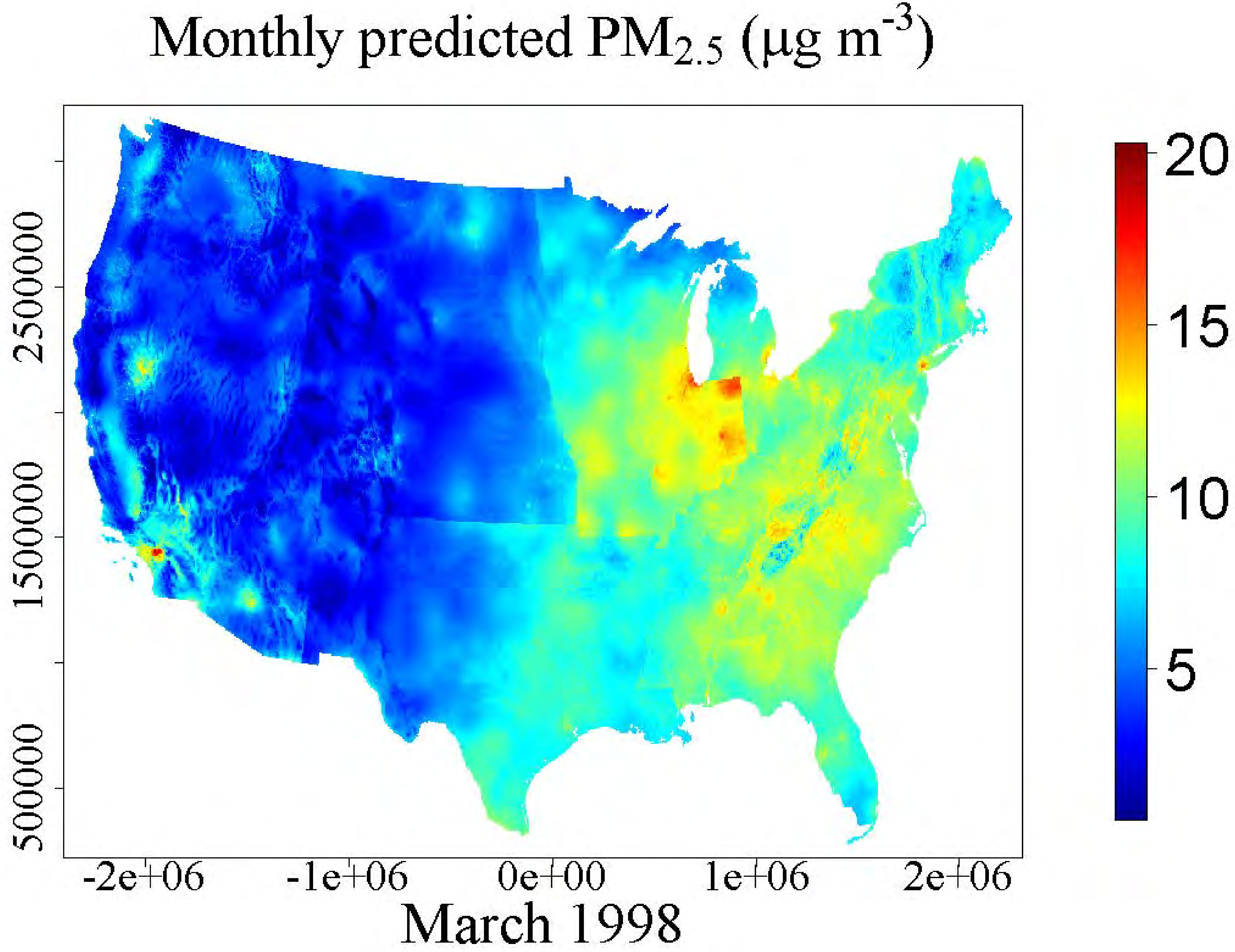
Monthly predicted $PM_{2.5}$ ($\mu g\ m^{-3}$)
25000000
15000000
5000000
-2e+06
-1e+06
0e+00
1e+06
2e+06
March 1998
20
15
10
5

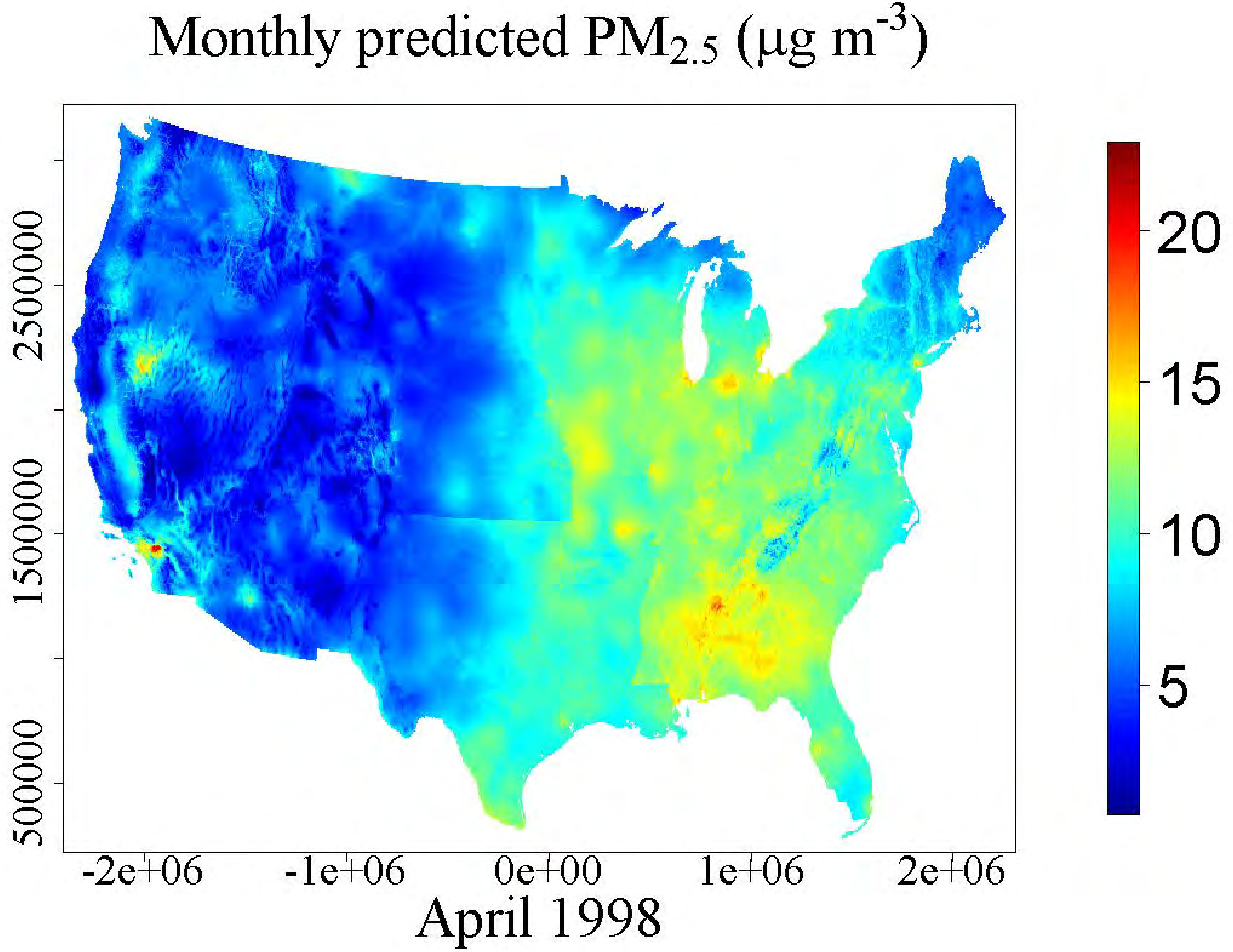

Monthly predicted PM2.5 (μg m-3)
25000000
15000000
5000000
-2e+06
-1e+06
0e+00
1e+06
2e+06
April 1998
20
15
10
5

Monthly predicted $PM_{2.5}$ ($\mu g\ m^{-3}$)

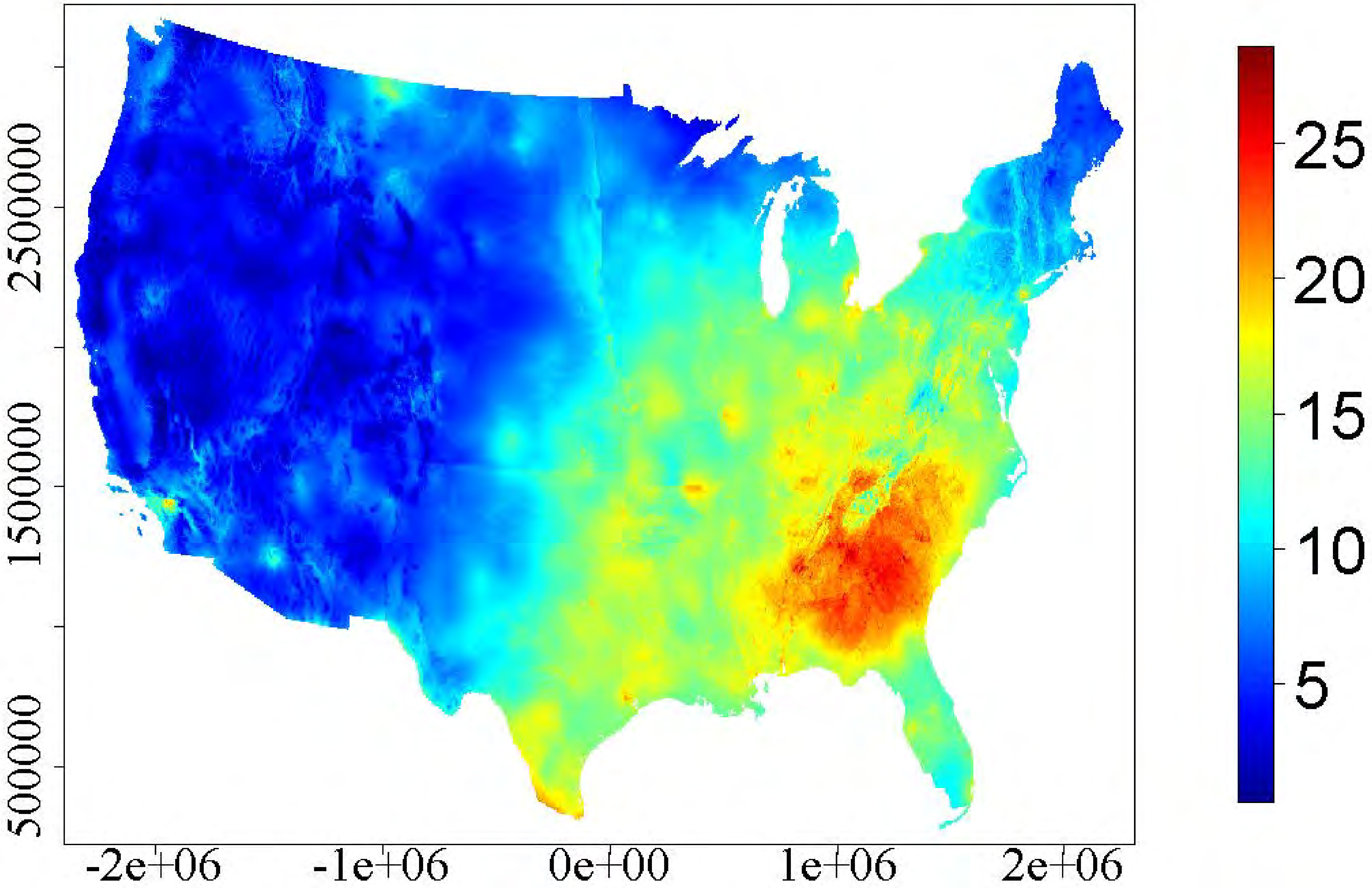

May 1998

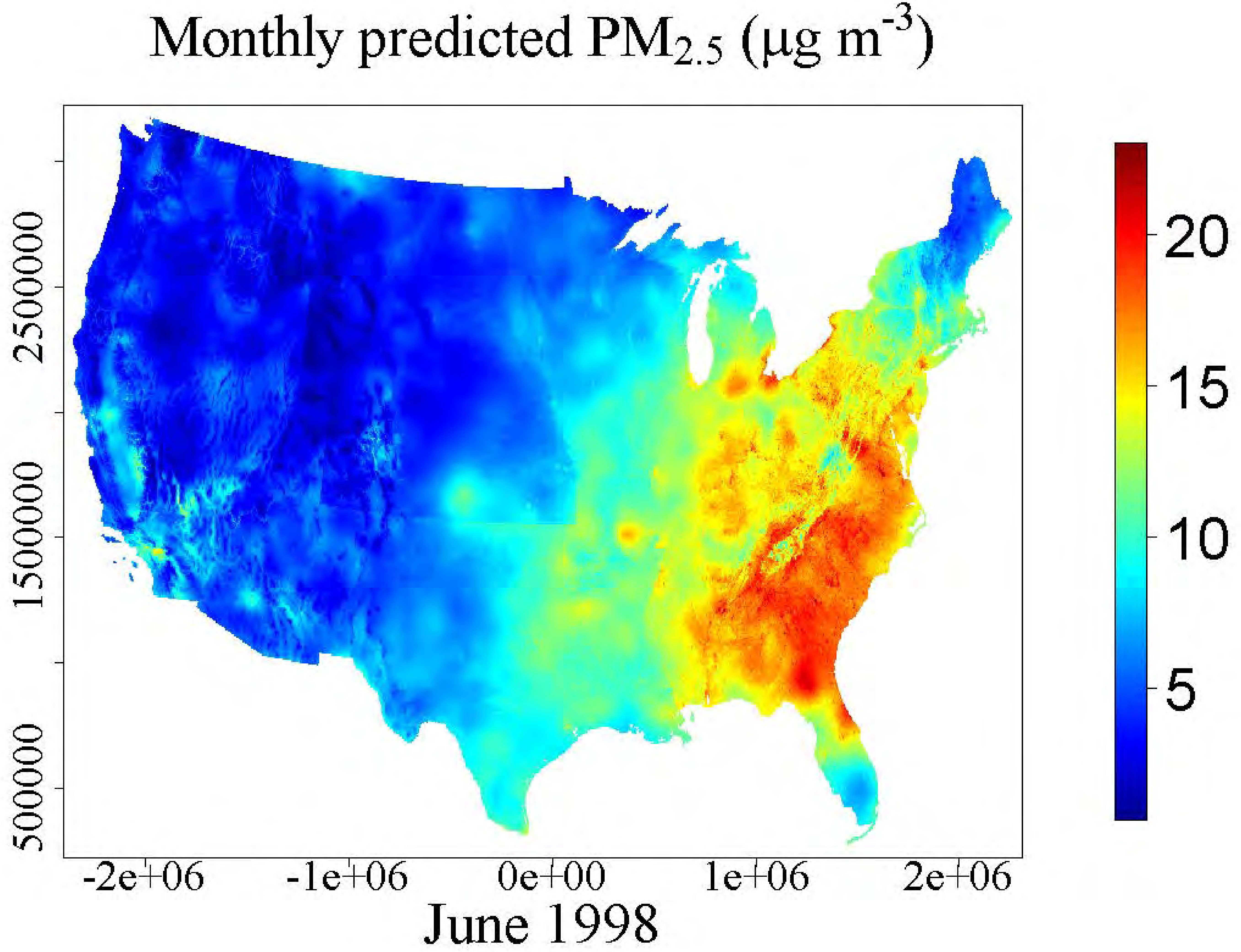

Monthly predicted PM2.5 (μg m-3)
25000000
15000000
5000000
-2e+06
-1e+06
0e+00
1e+06
2e+06
June 1998
20
15
10
5

Monthly predicted $PM_{2.5}$ ($\mu g\ m^{-3}$)

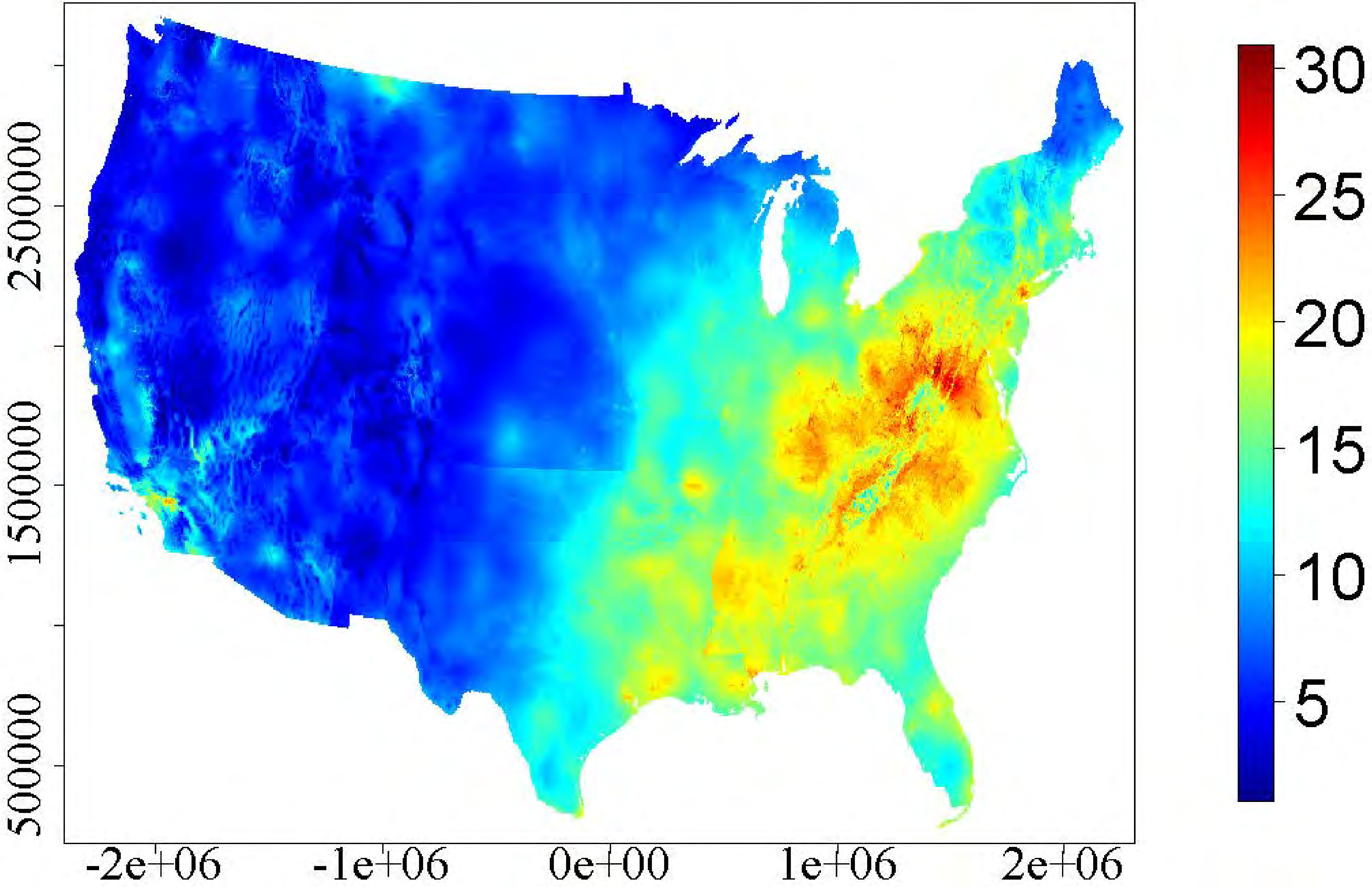

July 1998

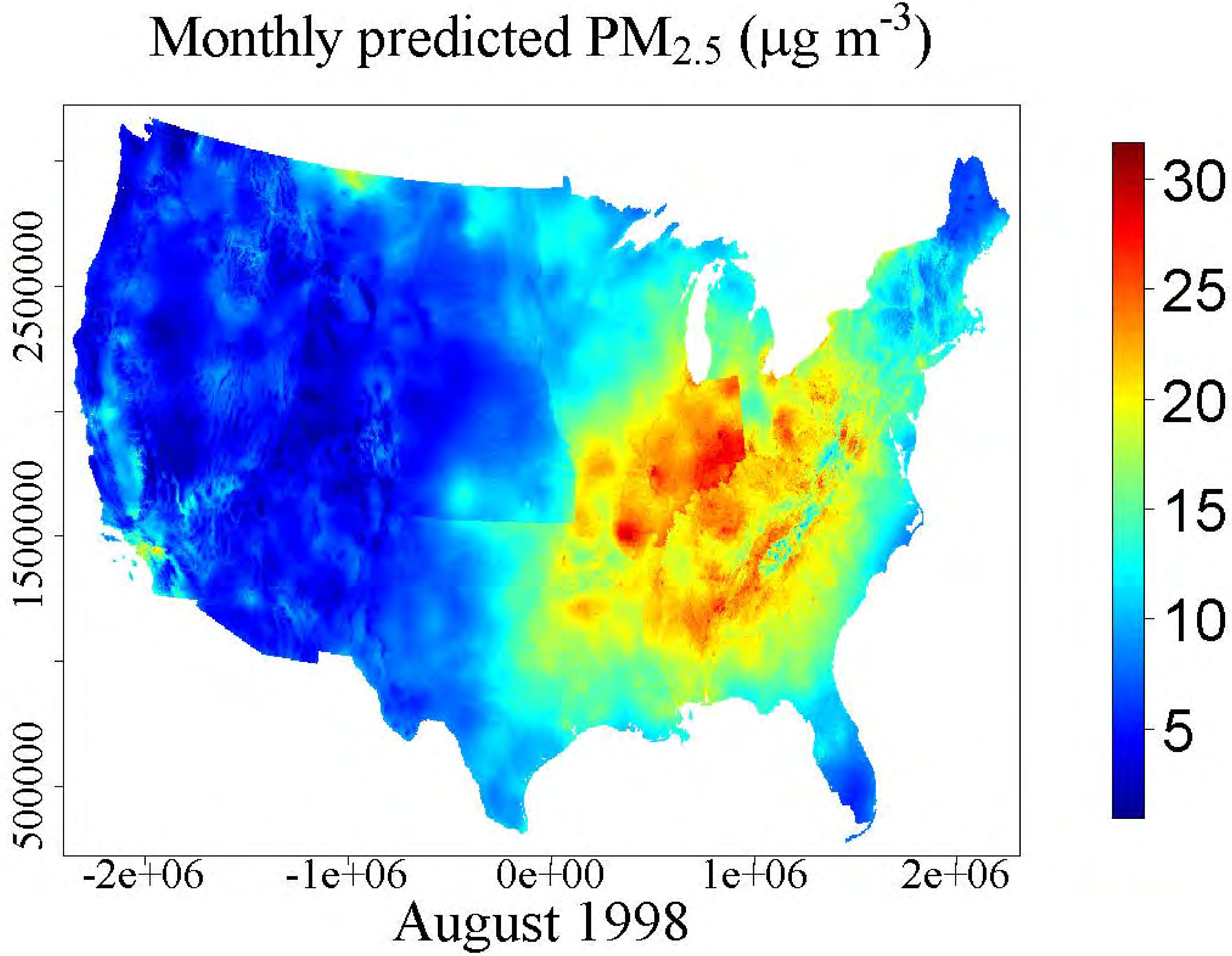
Monthly predicted PM2.5 (μg m-3)
25000000
15000000
5000000
-2e+06
-1e+06
0e+00
1e+06
2e+06
August 1998
30
25
20
15
10
5

Monthly predicted $PM_{2.5}$ ($\mu g\ m^{-3}$)

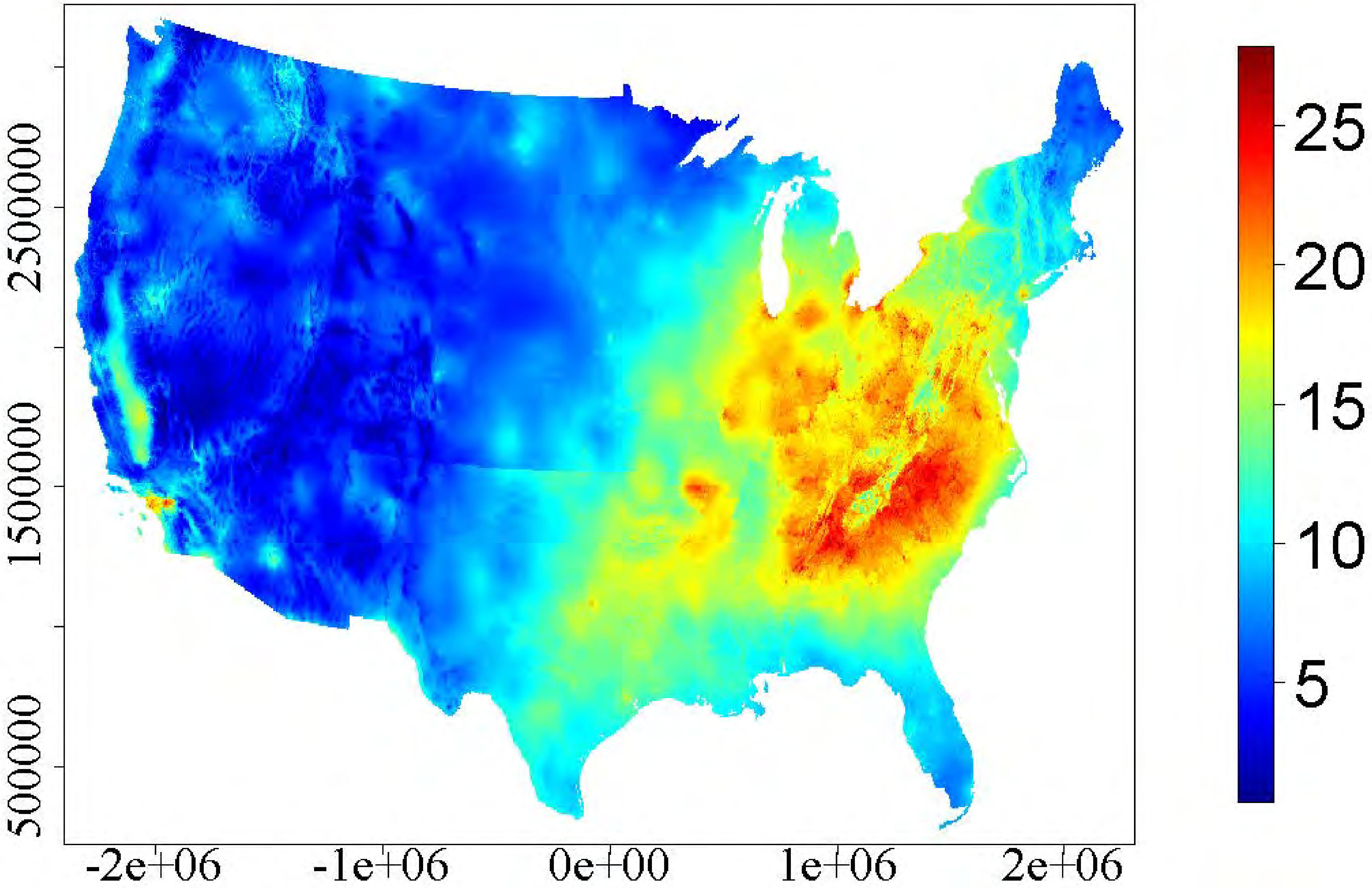

September 1998

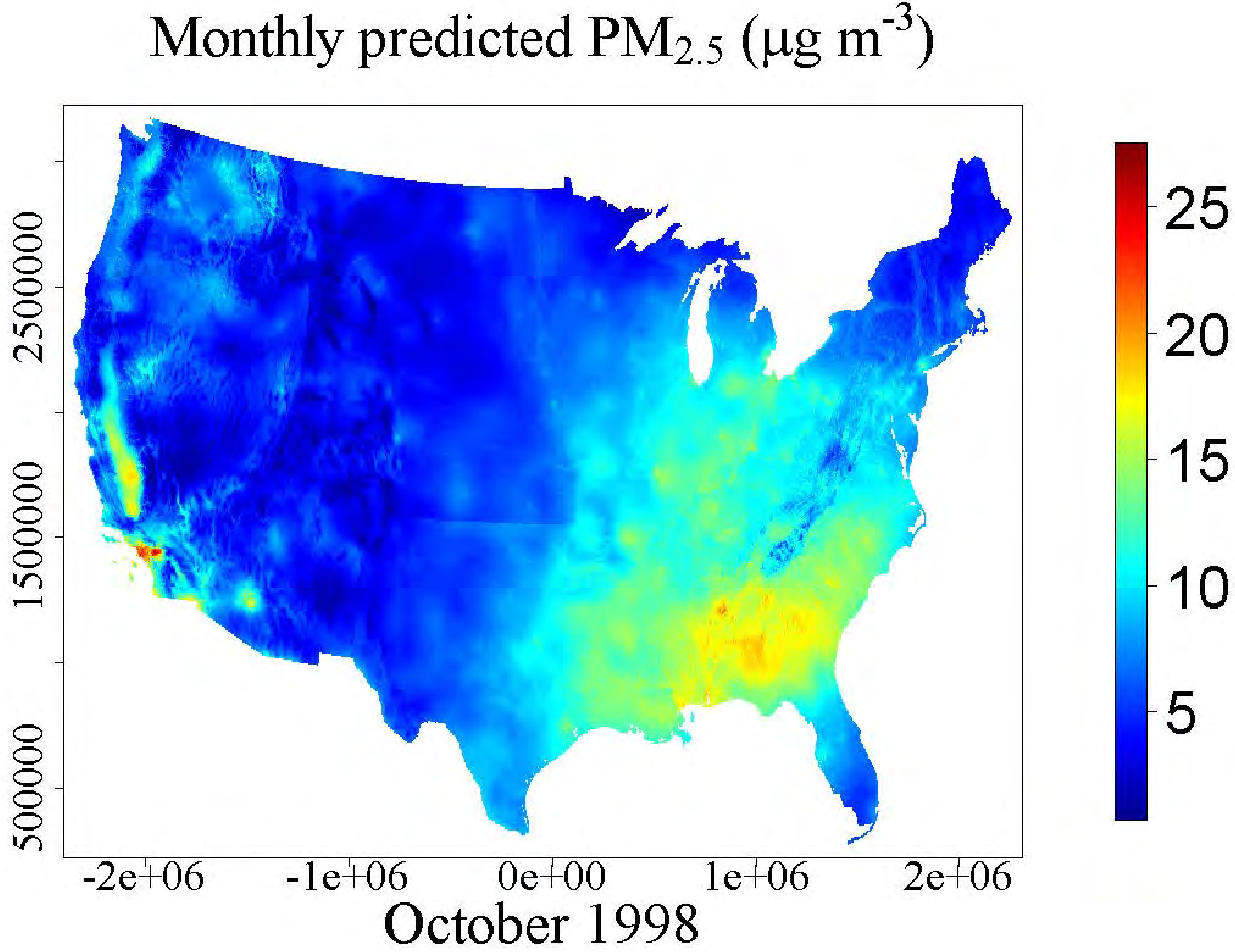

Monthly predicted PM2.5 (µg m-3)
25000000
15000000
5000000
-2e+06
-1e+06
0e+00
1e+06
2e+06
October 1998
25
20
15
10
5

Monthly predicted $PM_{2.5}$ ($\mu g\ m^{-3}$)

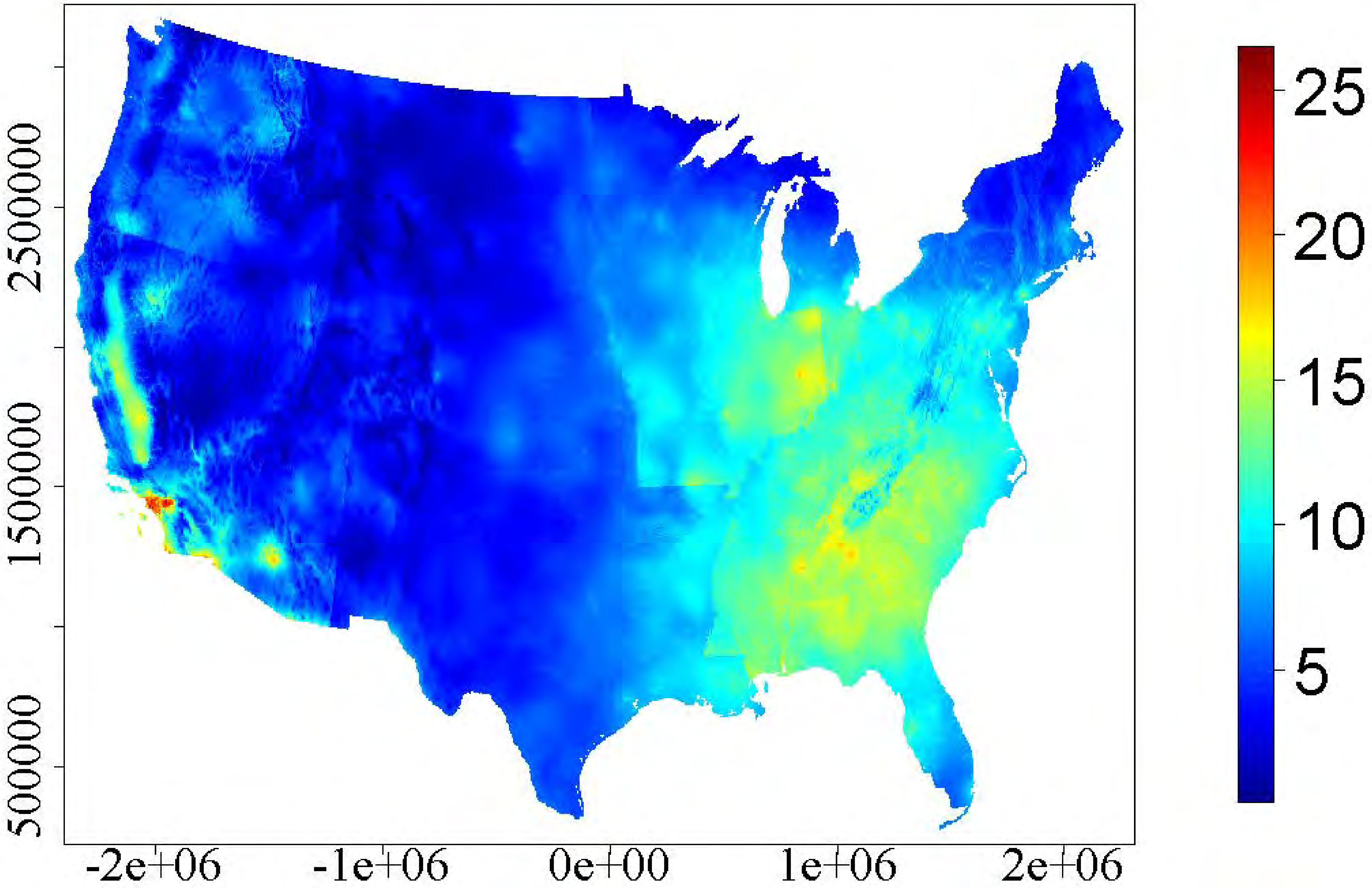

November 1998

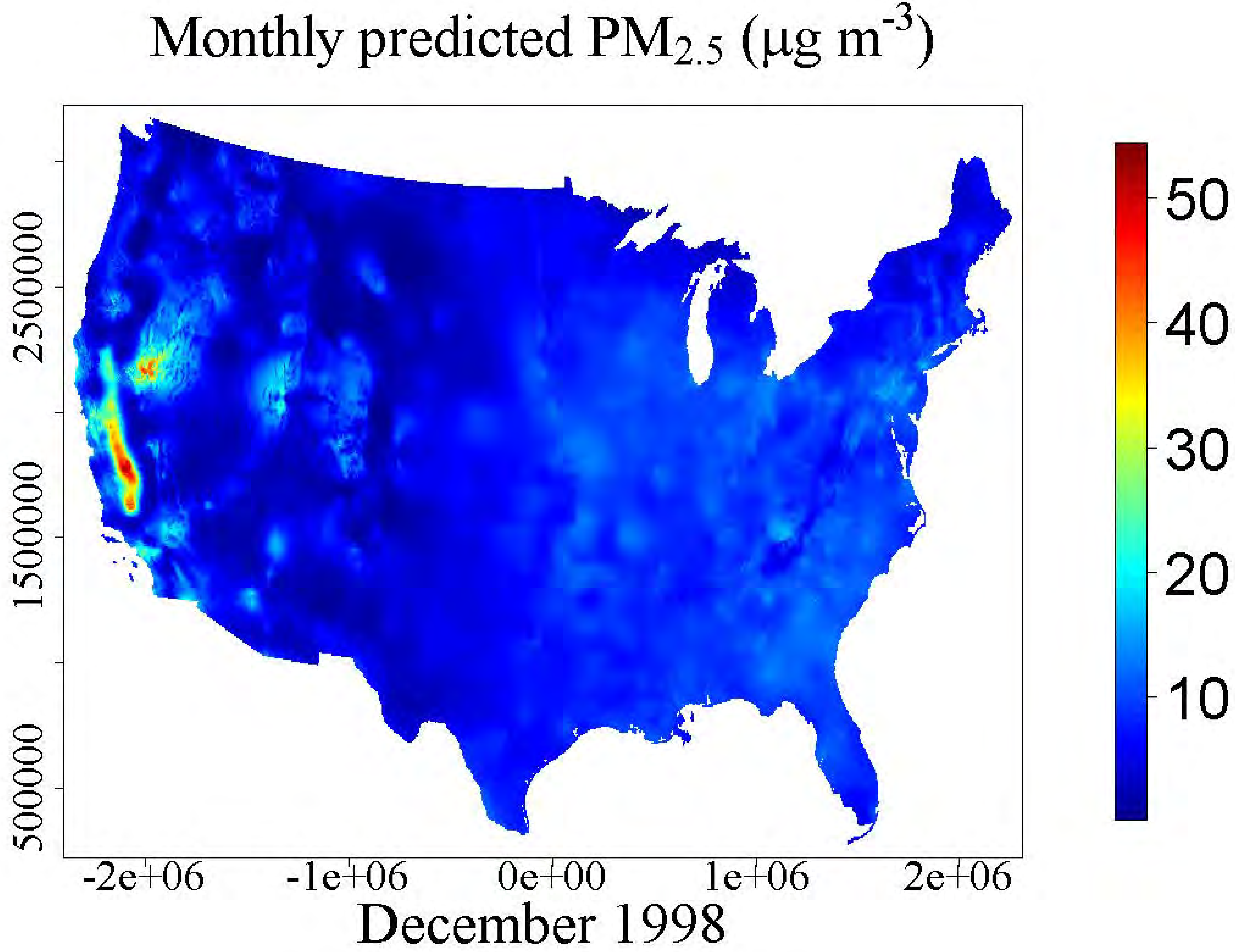
Monthly predicted PM2.5 (µg m-3)
25000000
15000000
5000000
-2e+06
-1e+06
0e+00
1e+06
2e+06
December 1998
50
40
30
20
10

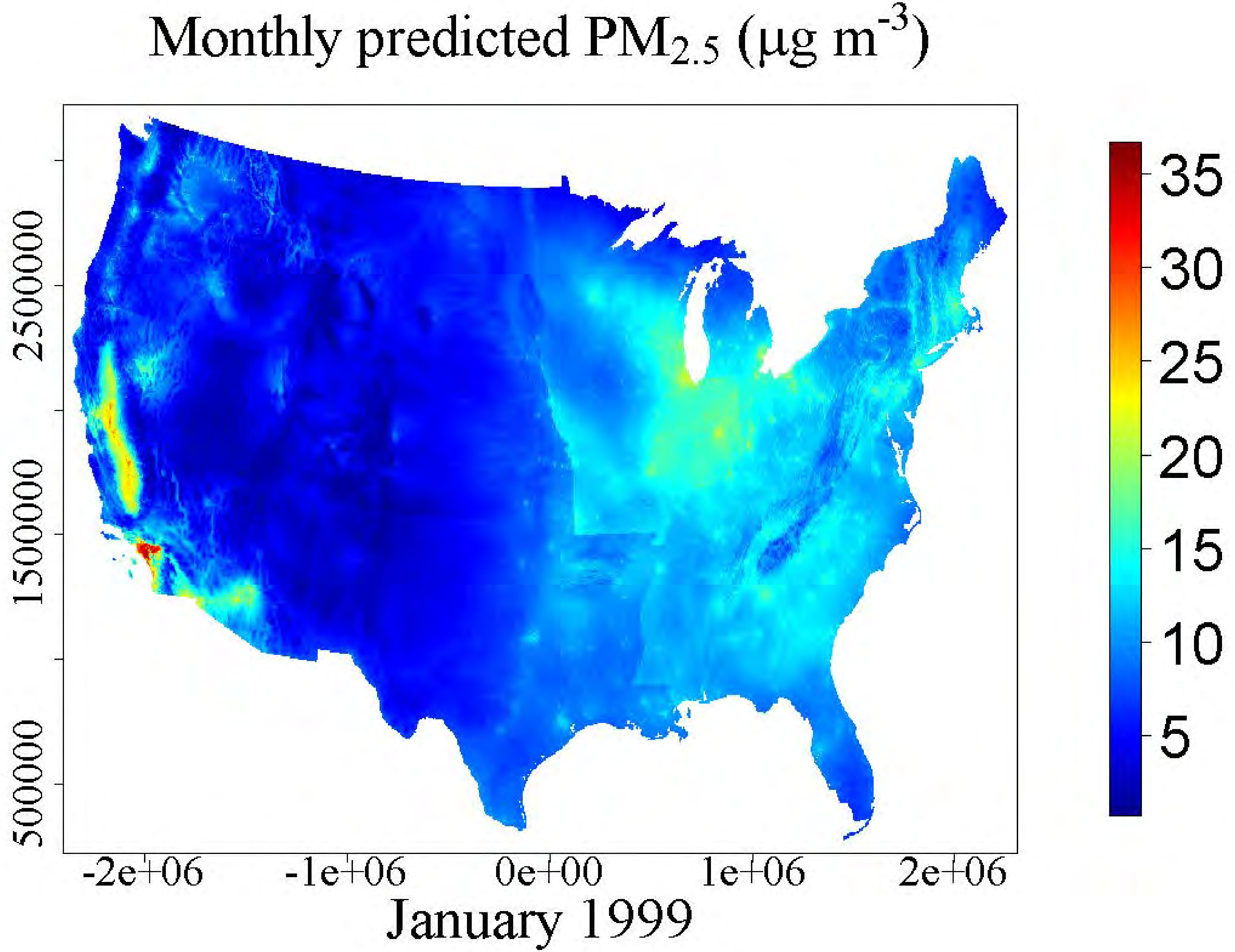
Monthly predicted PM2.5 (μg m-3)
25000000
15000000
5000000
-2e+06
-1e+06
0e+00
1e+06
2e+06
January 1999
35
30
25
20
15
10
5

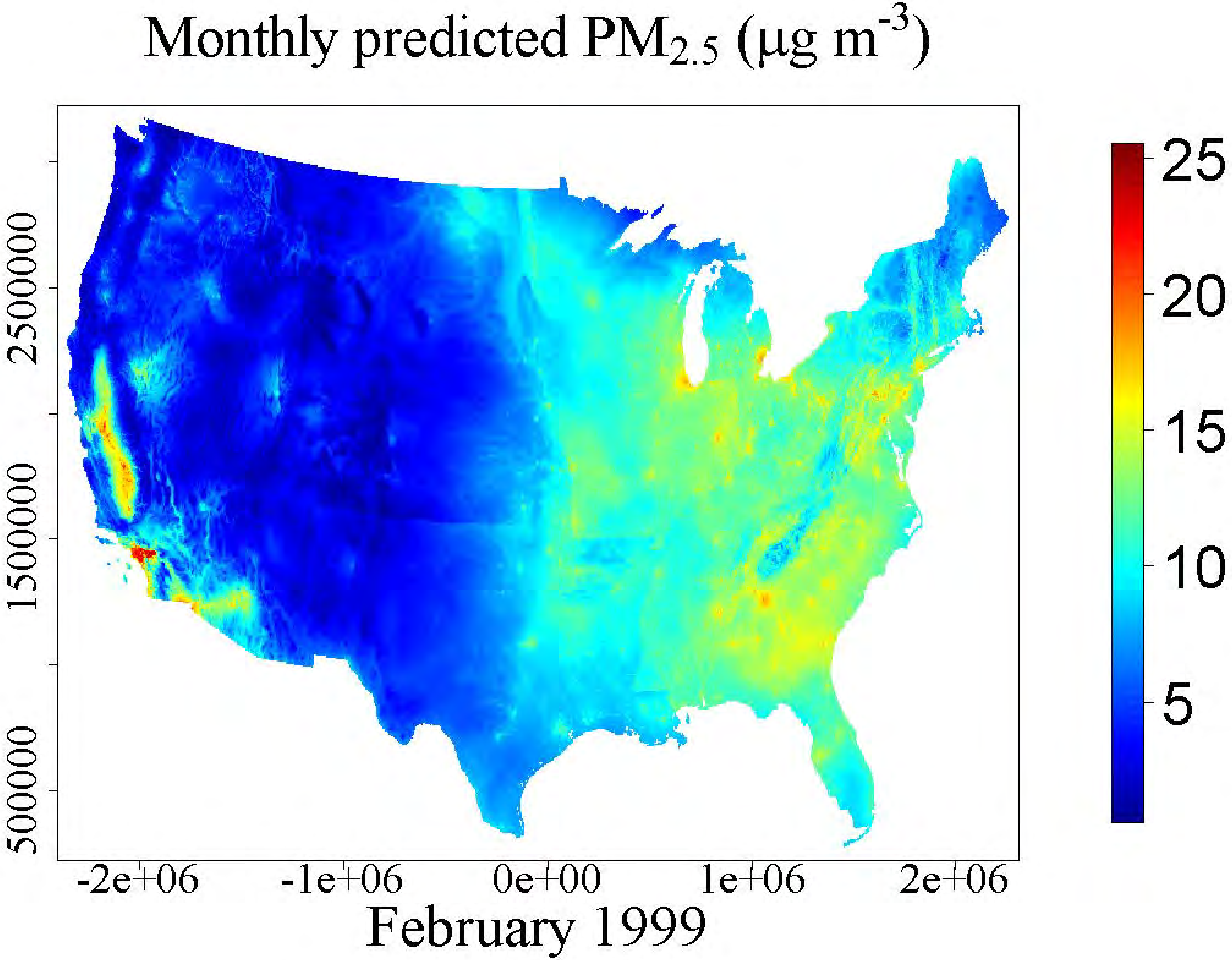
Monthly predicted PM2.5 (μg m-3)
25000000
15000000
5000000
-2e+06
-1e+06
0e+00
1e+06
2e+06
25
20
15
10
5
February 1999

Monthly predicted $PM_{2.5}$ ($\mu g\ m^{-3}$)

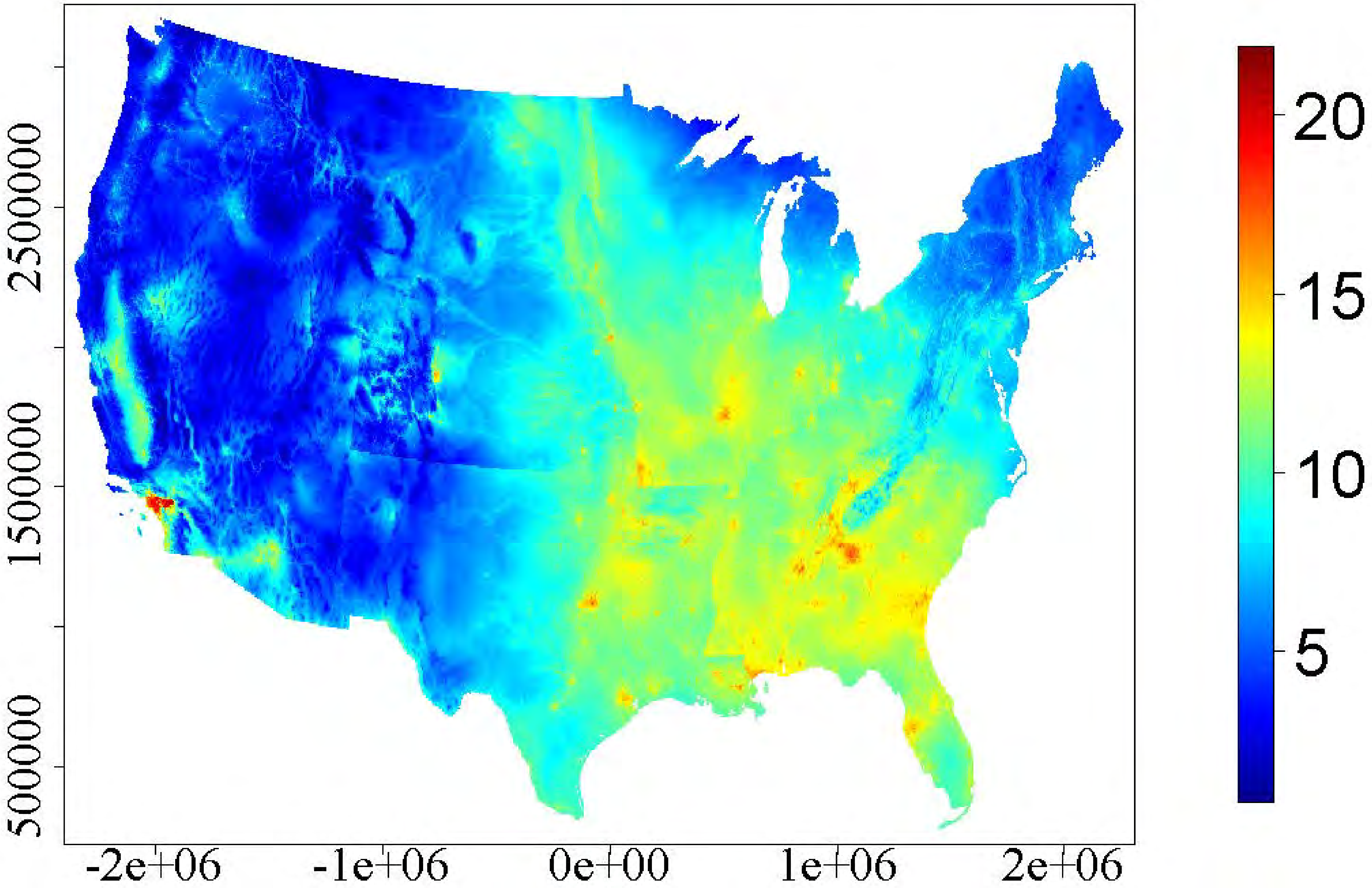

March 1999

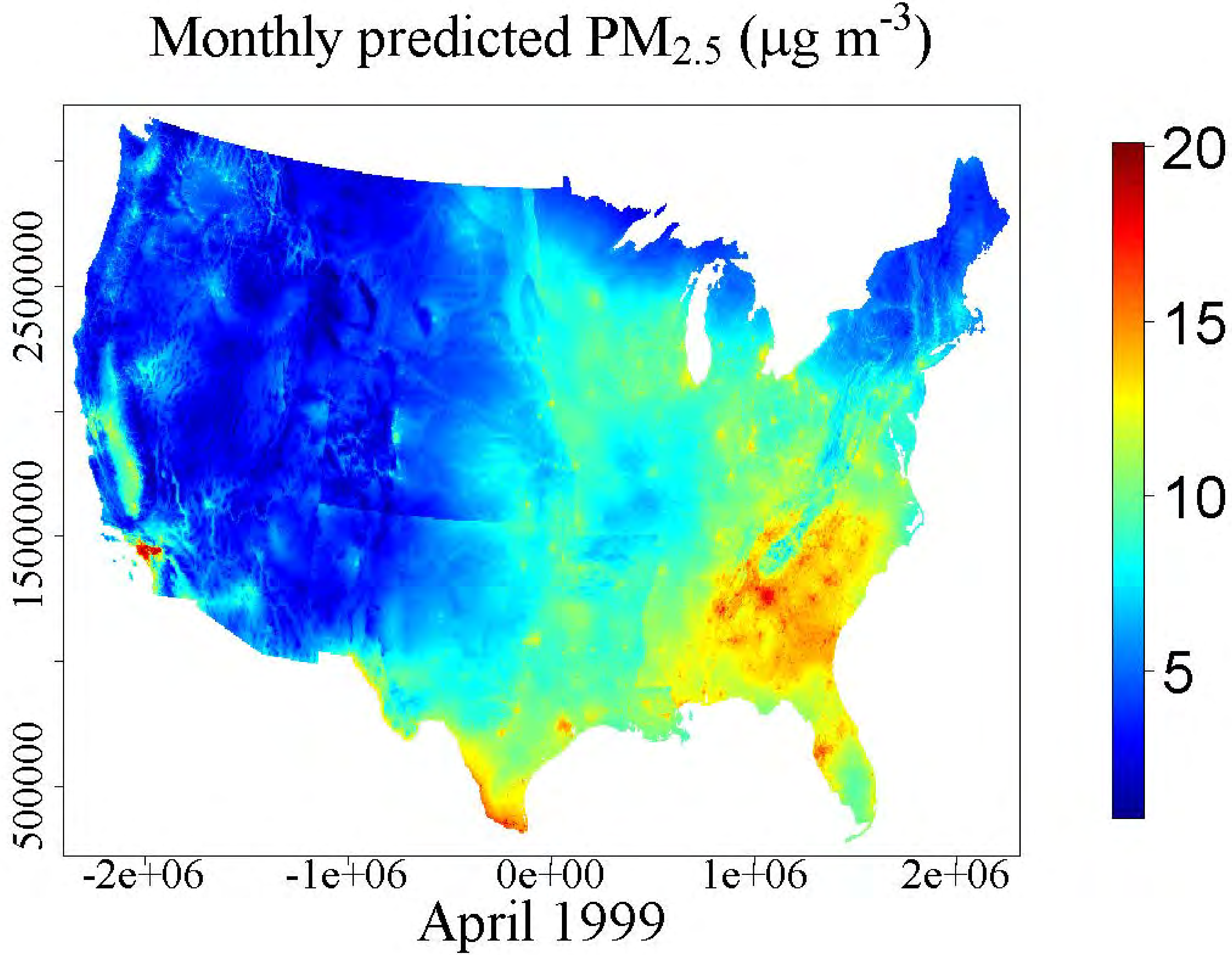
Monthly predicted PM2.5 (μg m-3)
25000000
15000000
5000000
-2e+06
-1e+06
0e+00
1e+06
2e+06
April 1999
20
15
10
5

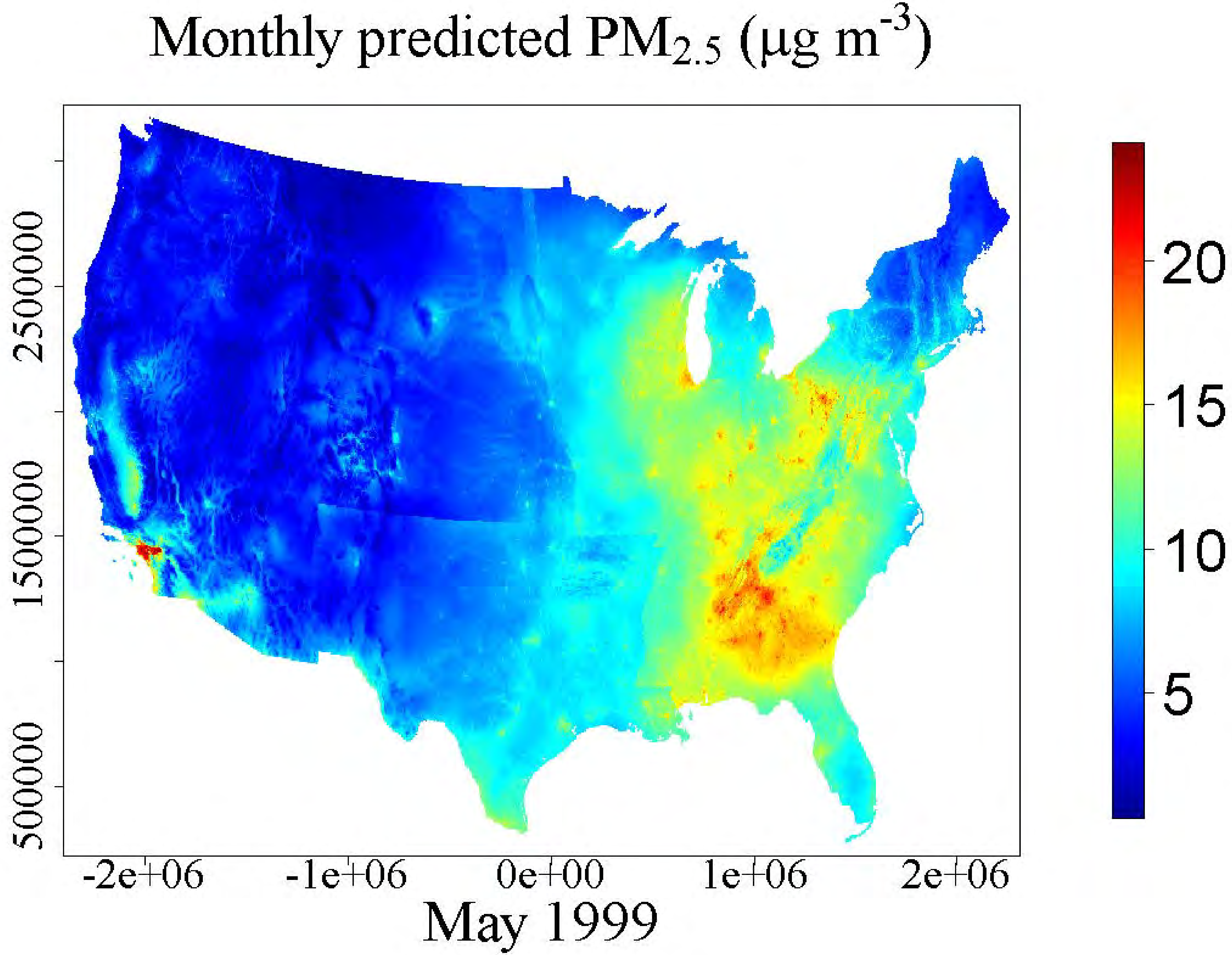
Monthly predicted PM2.5 (μg m-3)
25000000
15000000
5000000
-2e+06
-1e+06
0e+00
1e+06
2e+06
20
15
10
5
May 1999

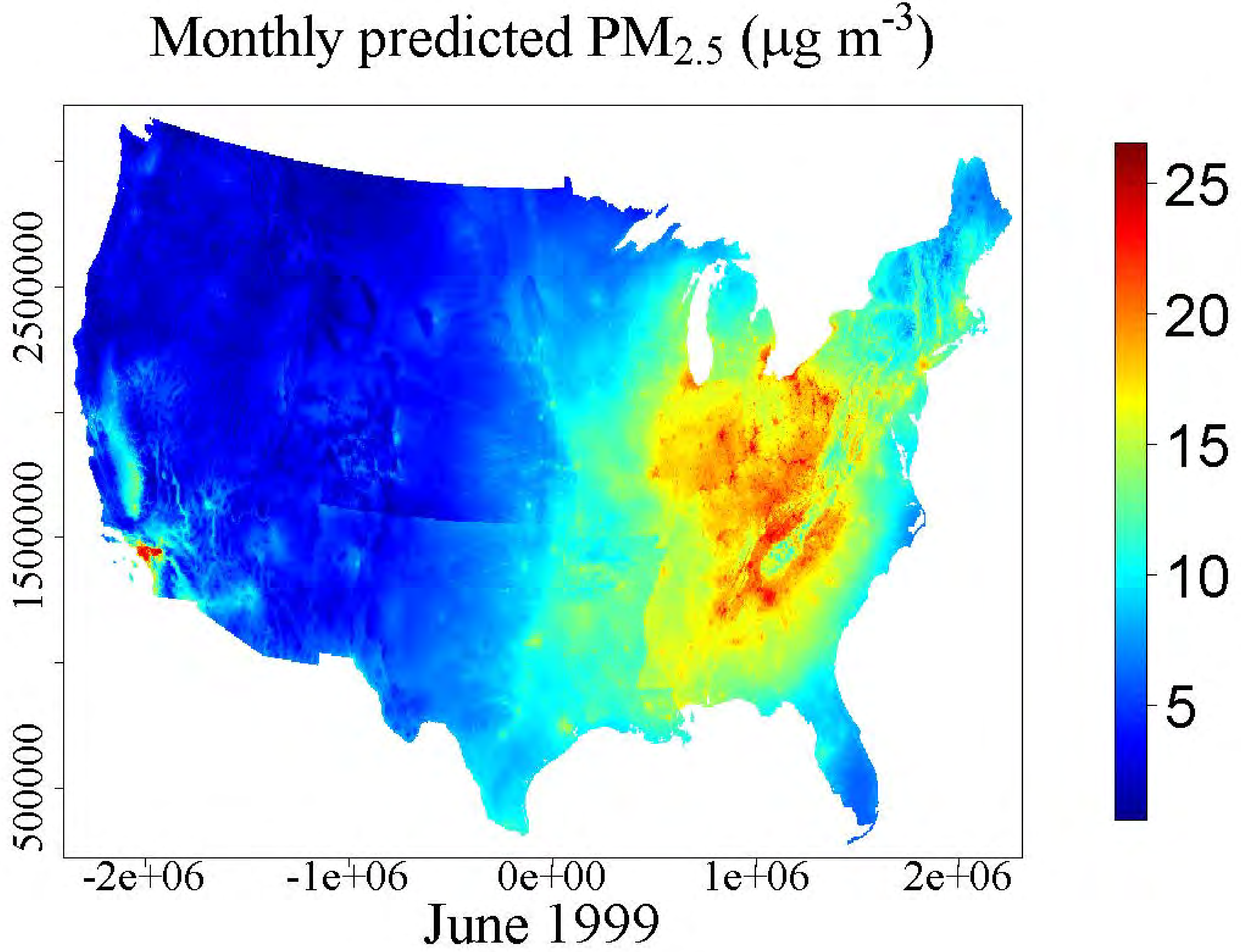
Monthly predicted PM2.5 (μg m-3)
25000000
15000000
5000000
-2e+06
-1e+06
0e+00
1e+06
2e+06
June 1999
25
20
15
10
5

Monthly predicted $PM_{2.5}$ ($\mu g\ m^{-3}$)

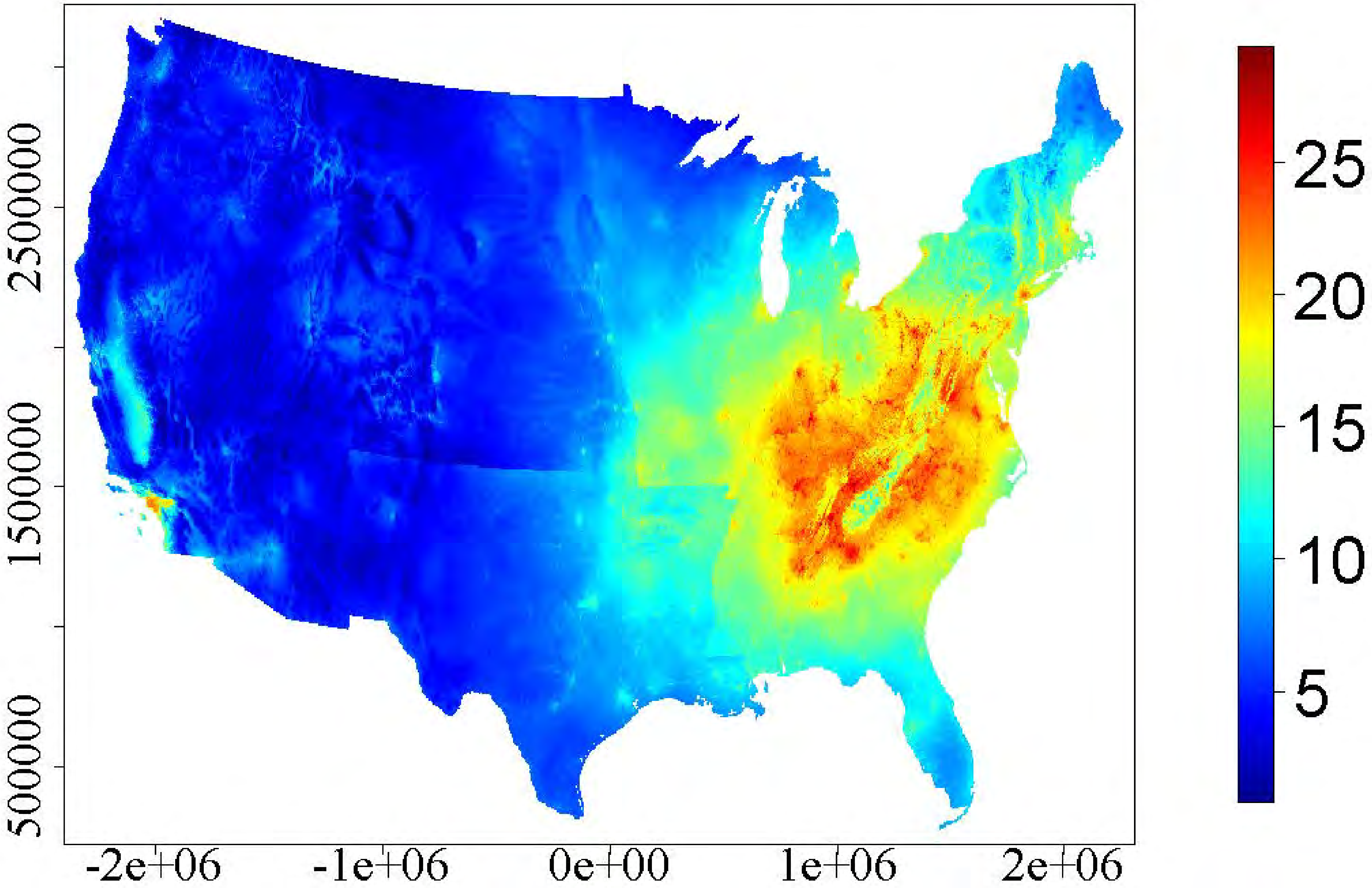

July 1999

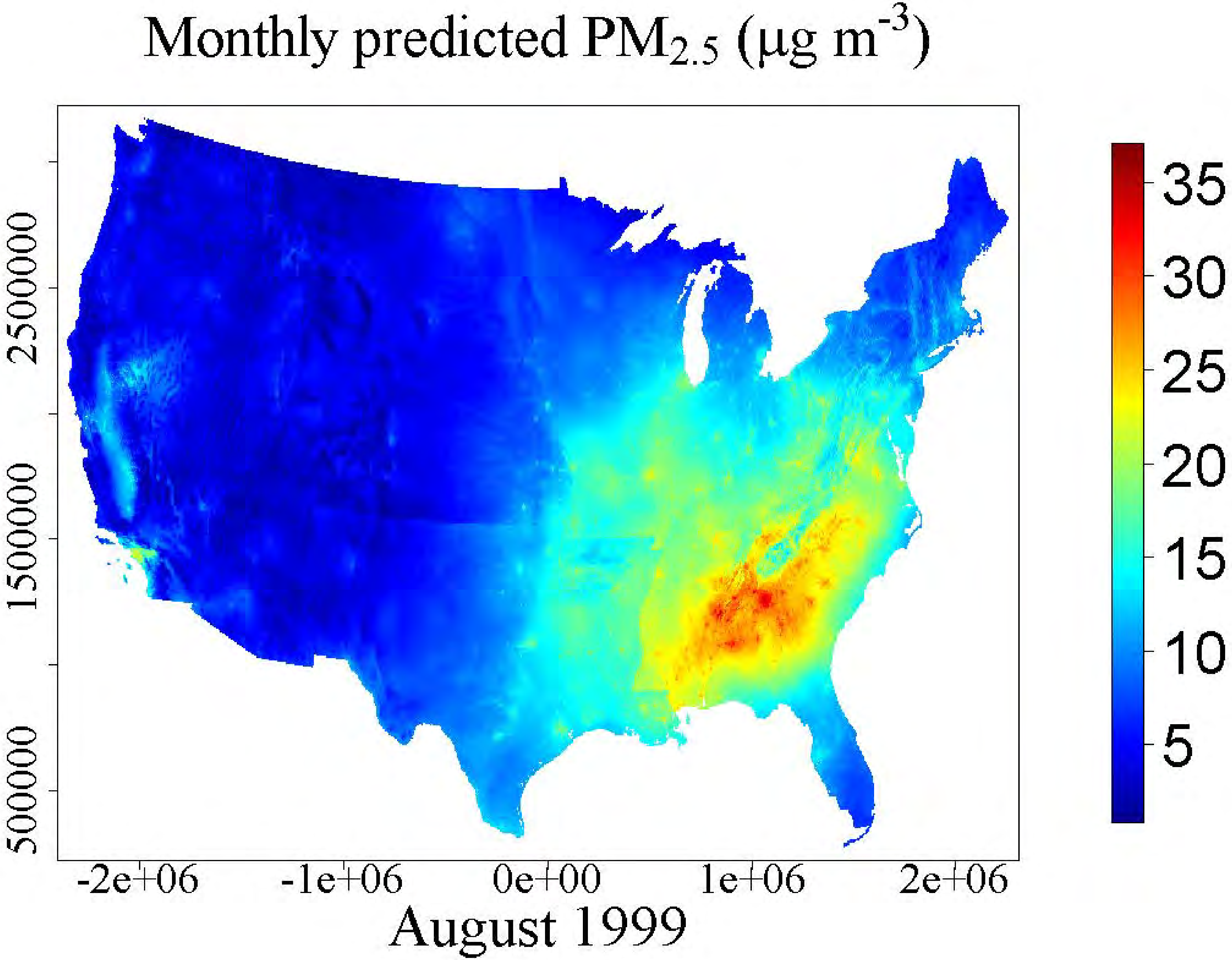
Monthly predicted $PM_{2.5}$ ($\mu g\ m^{-3}$)
25000000
15000000
5000000
-2e+06
-1e+06
0e+00
1e+06
2e+06
August 1999
35
30
25
20
15
10
5

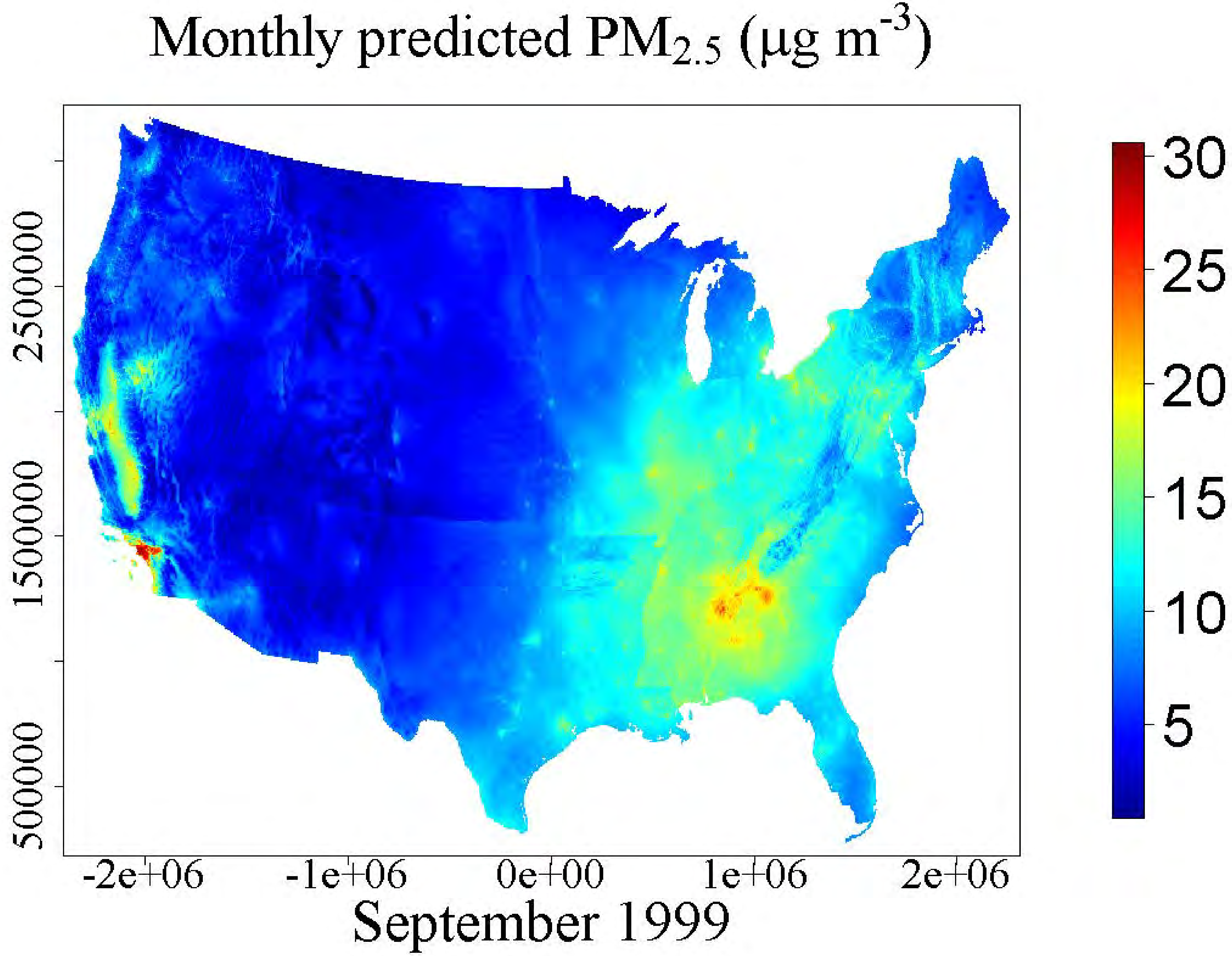

Monthly predicted PM2.5 (μg m-3)
25000000
15000000
5000000
-2e+06
-1e+06
0e+00
1e+06
2e+06
30
25
20
15
10
5
September 1999

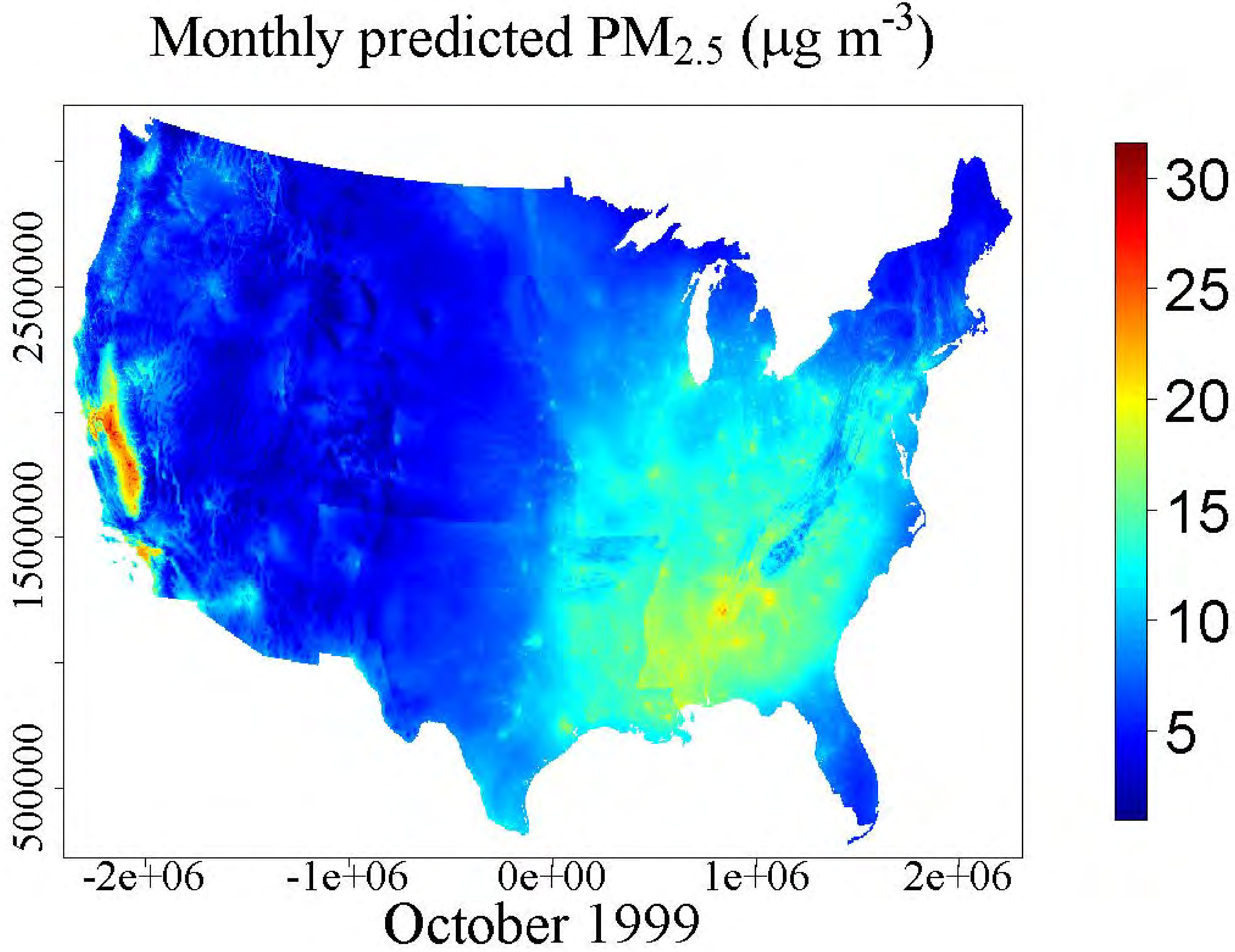
Monthly predicted PM2.5 (μg m-3)
25000000
15000000
5000000
-2e+06
-1e+06
0e+00
1e+06
2e+06
30
25
20
15
10
5
October 1999

Monthly predicted $PM_{2.5}$ ($\mu g\ m^{-3}$)

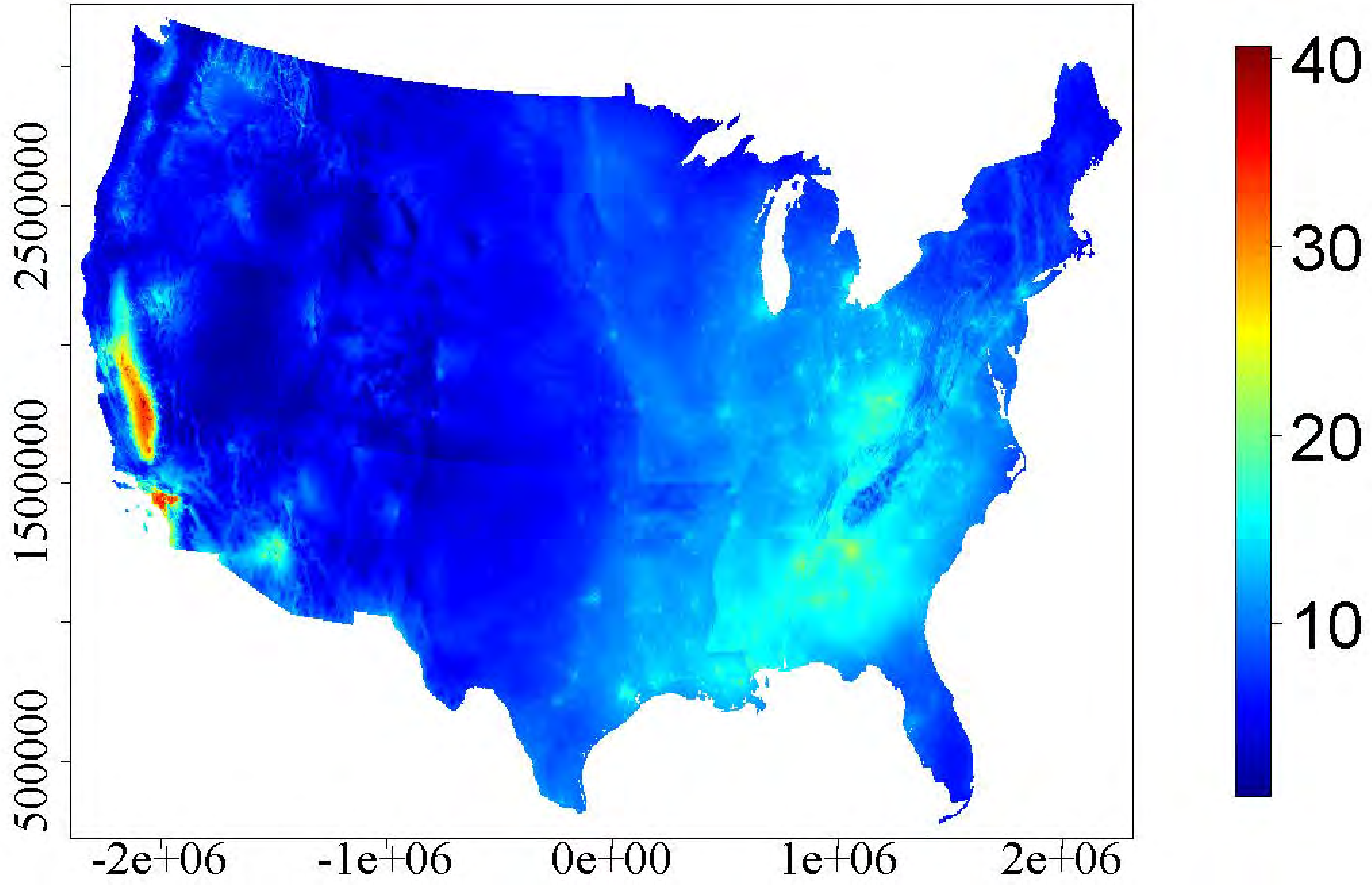

November 1999

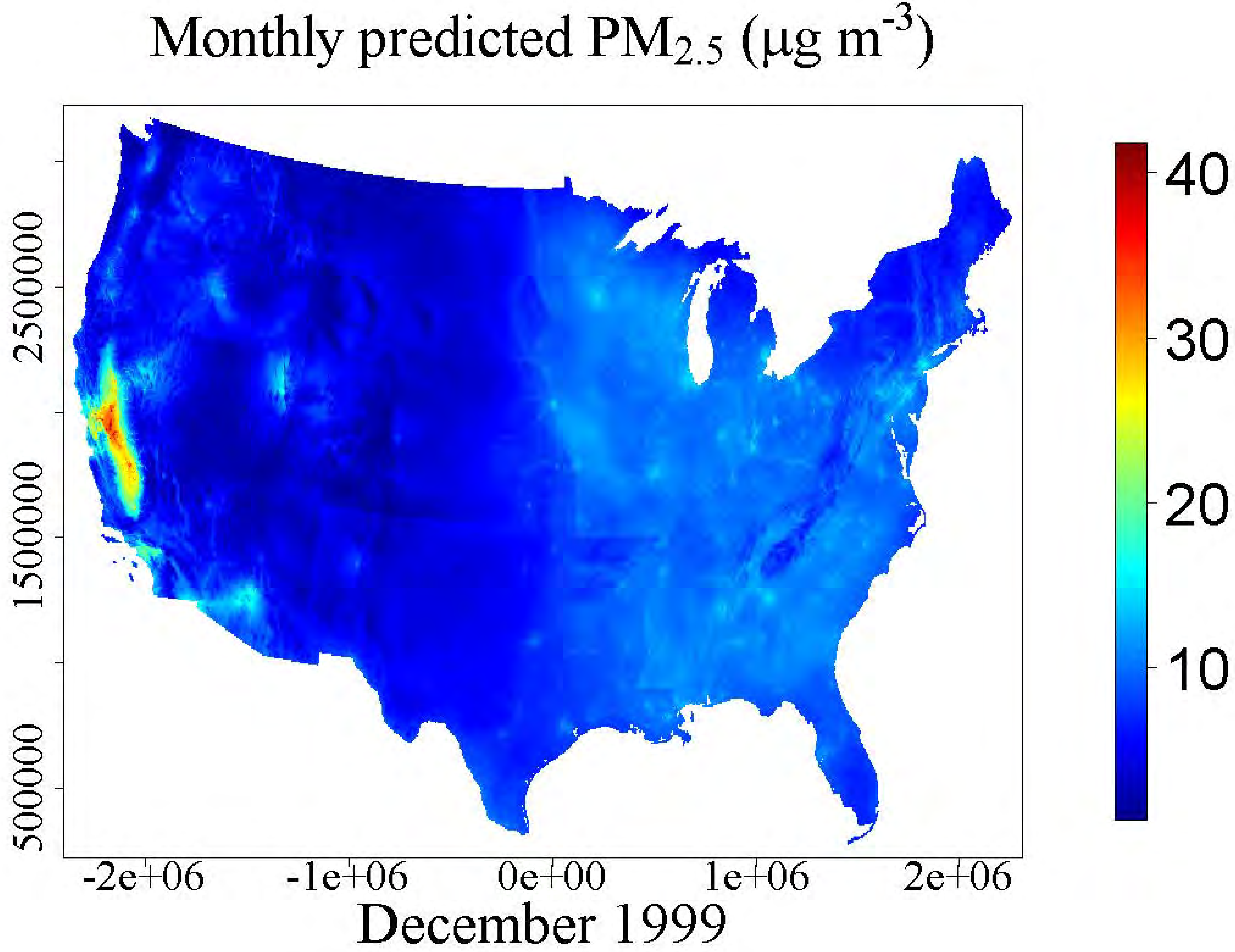

Monthly predicted PM2.5 (μg m-3)
40
30
20
10
25000000
15000000
5000000
-2e+06
-1e+06
0e+00
1e+06
2e+06
December 1999

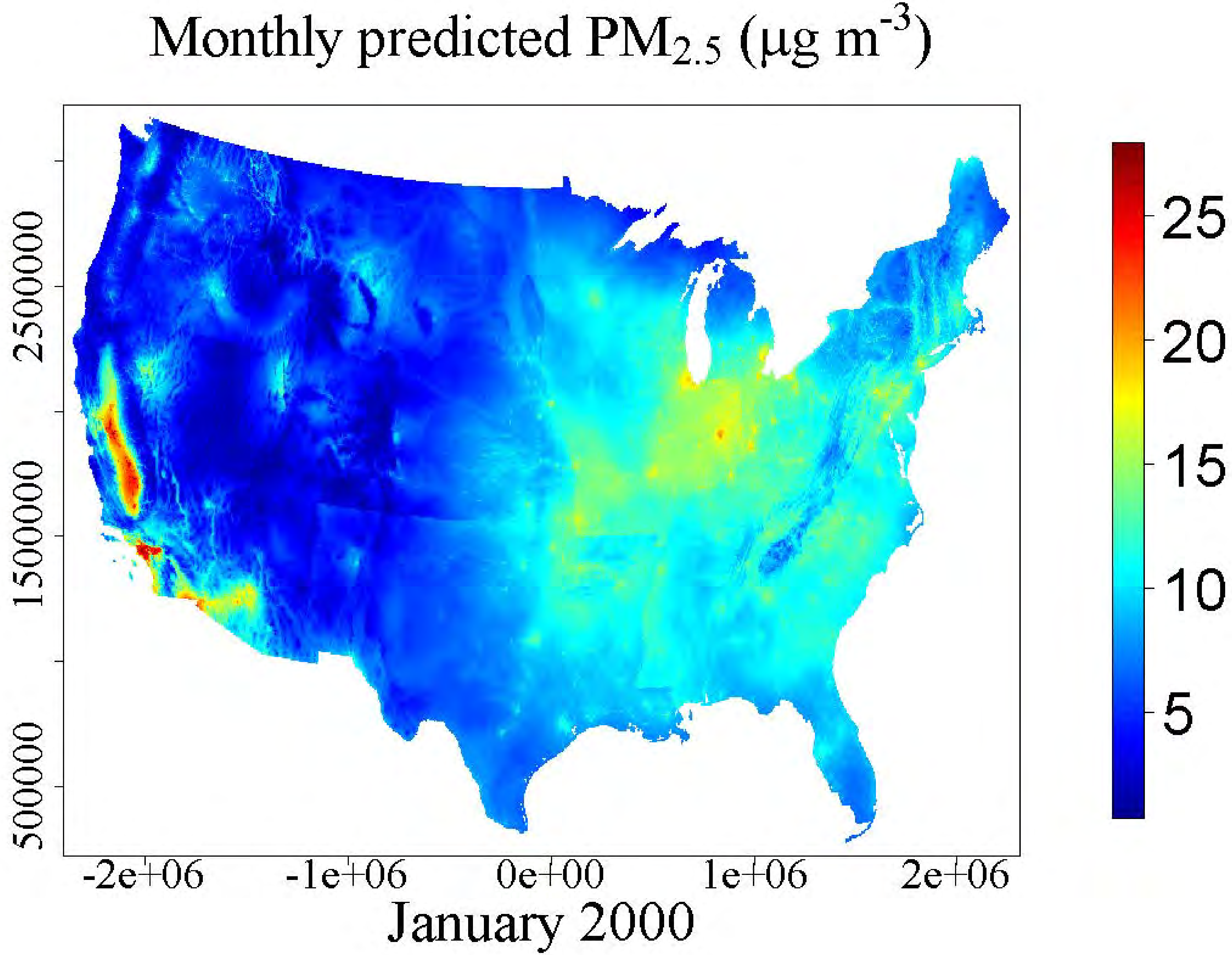
Monthly predicted $PM_{2.5}$ ($\mu g\ m^{-3}$)
25000000
15000000
5000000
-2e+06
-1e+06
0e+00
1e+06
2e+06
25
20
15
10
5
January 2000

Monthly predicted $PM_{2.5}$ ($\mu g\ m^{-3}$)

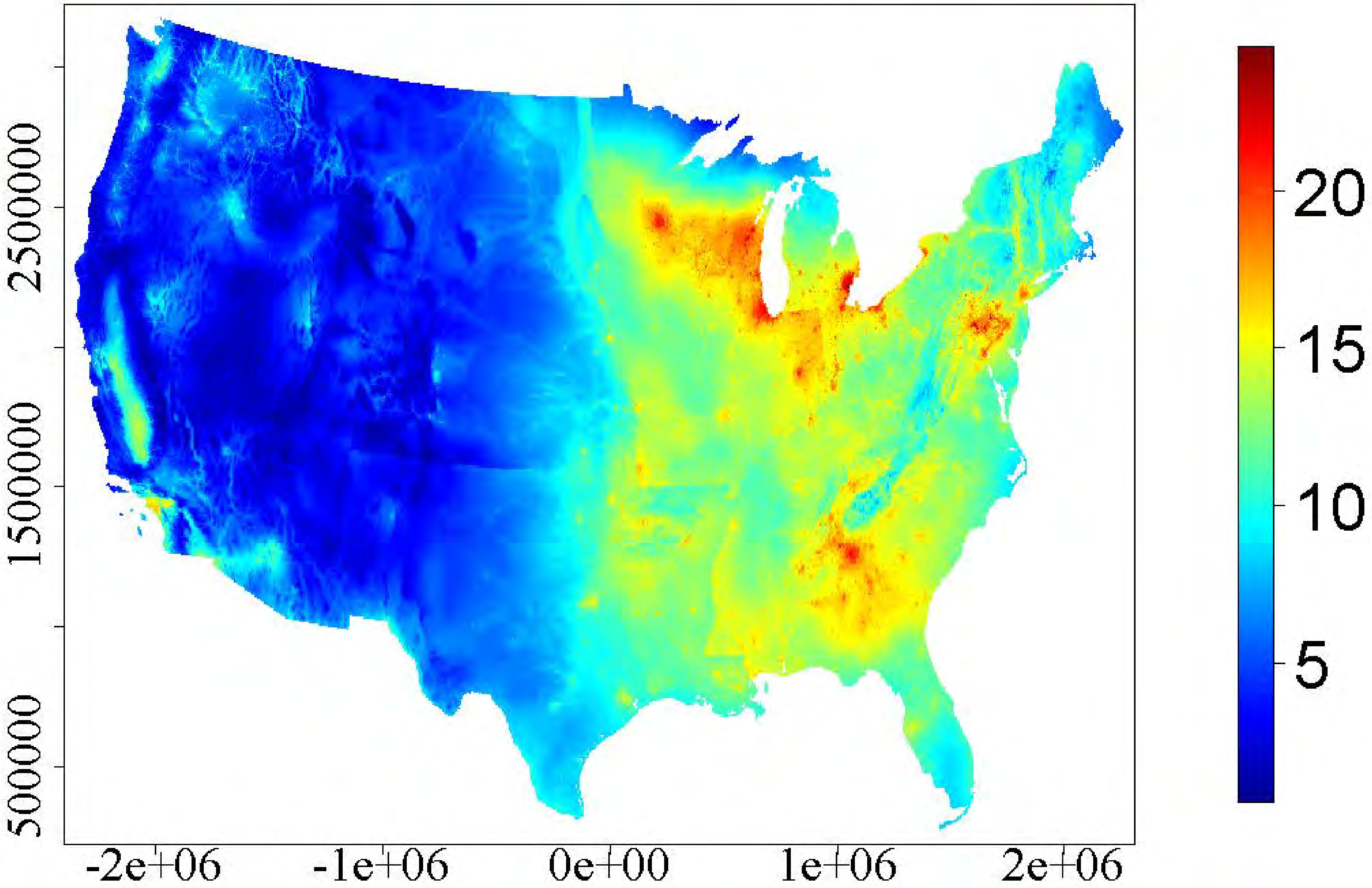

February 2000

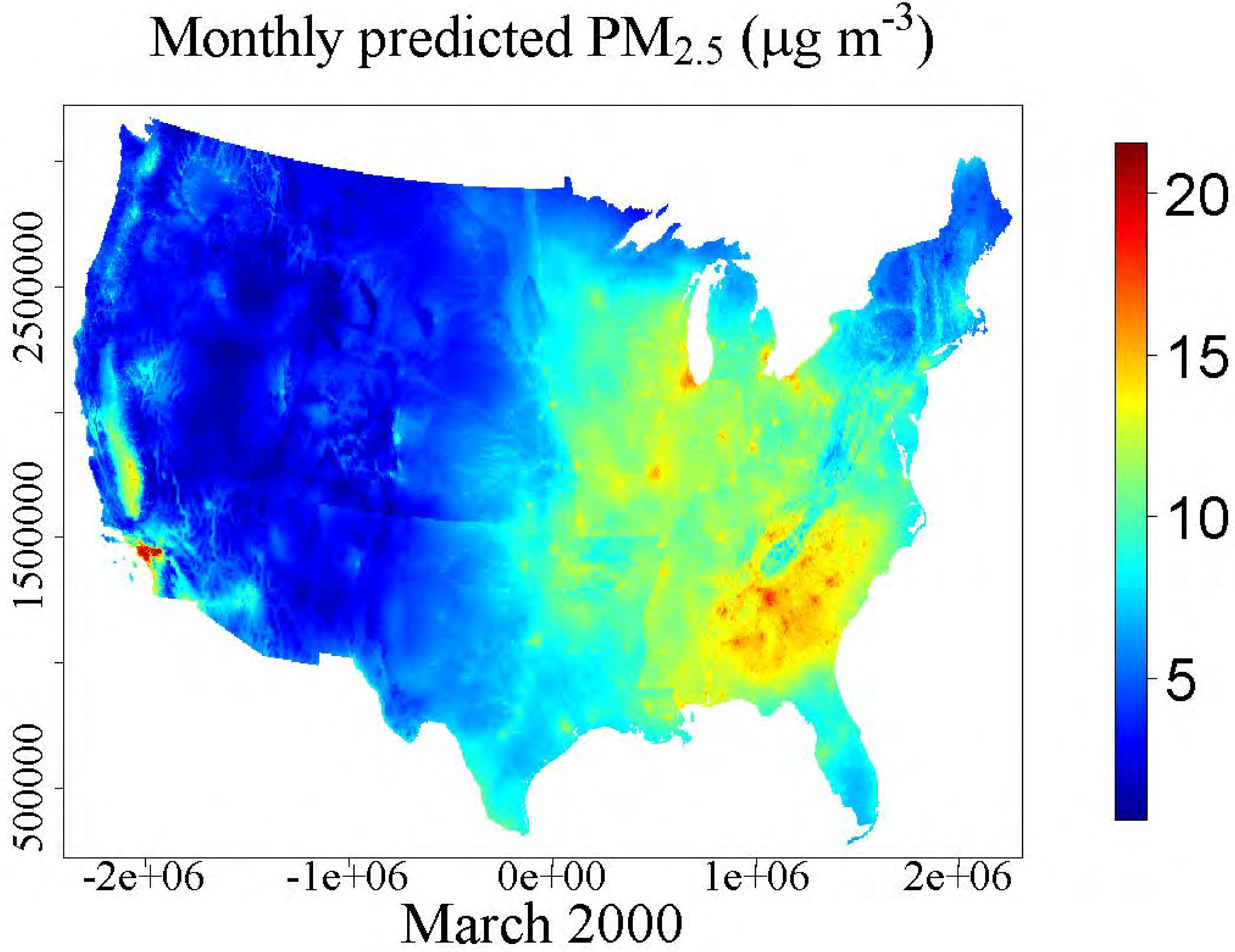
Monthly predicted PM2.5 (µg m-3)
25000000
15000000
5000000
-2e+06
-1e+06
0e+00
1e+06
2e+06
20
15
10
5
March 2000

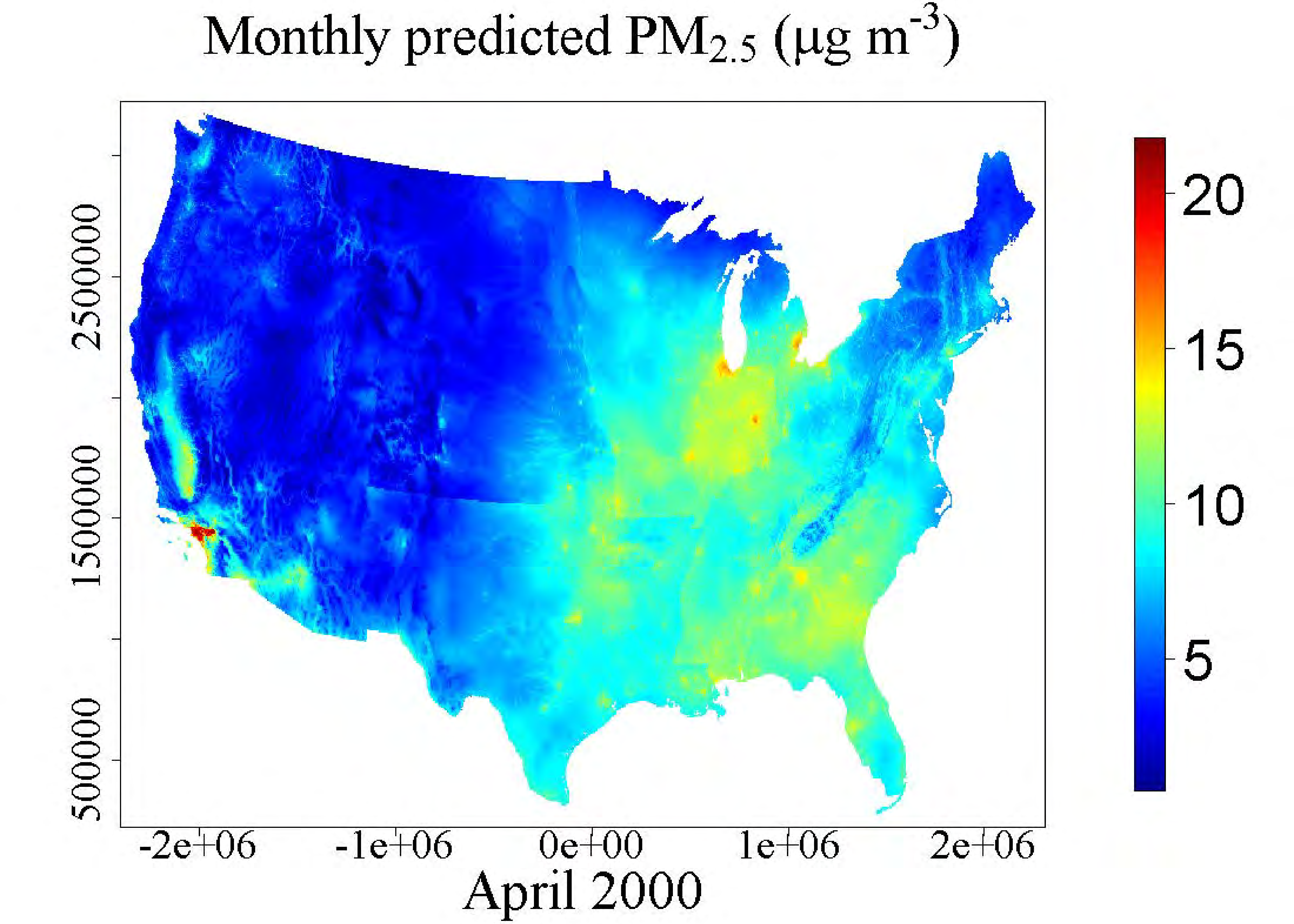

Monthly predicted PM2.5 (μg m-3)
25000000
15000000
5000000
-2e+06
-1e+06
0e+00
1e+06
2e+06
20
15
10
5
April 2000

Monthly predicted $PM_{2.5}$ ($\mu g\ m^{-3}$)

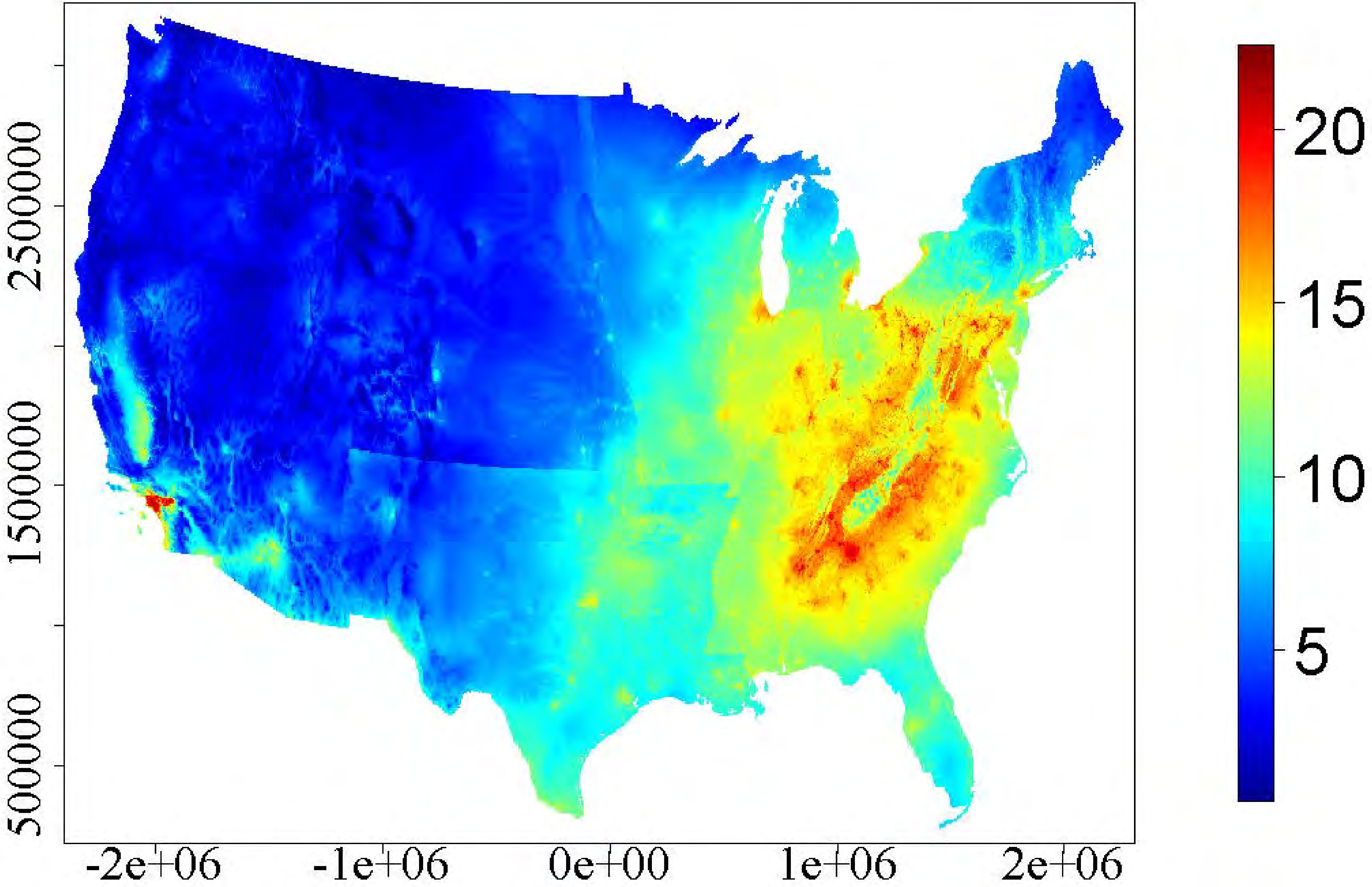

May 2000

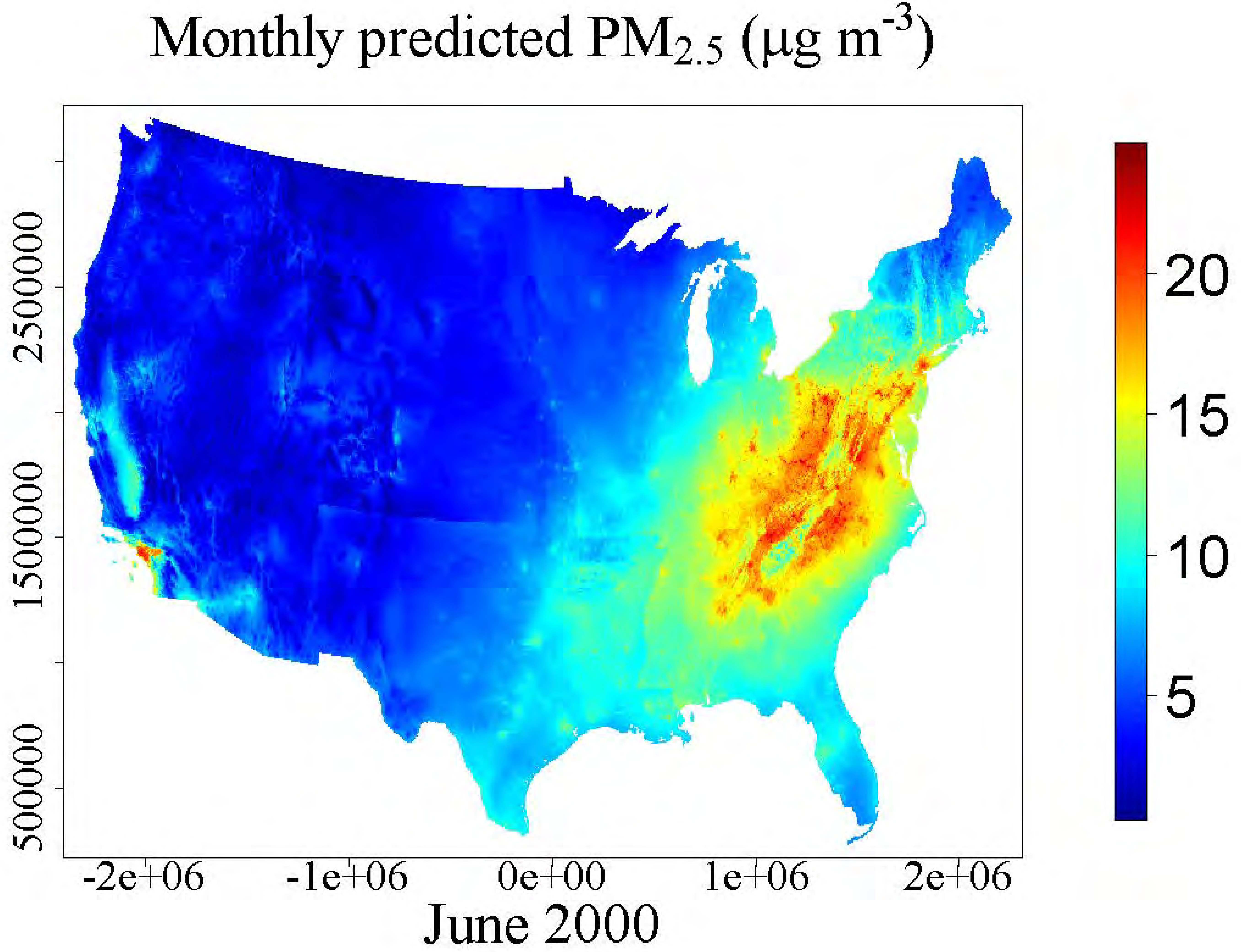
Monthly predicted PM2.5 (µg m-3)
25000000
15000000
5000000
-2e+06
-1e+06
0e+00
1e+06
2e+06
June 2000
20
15
10
5

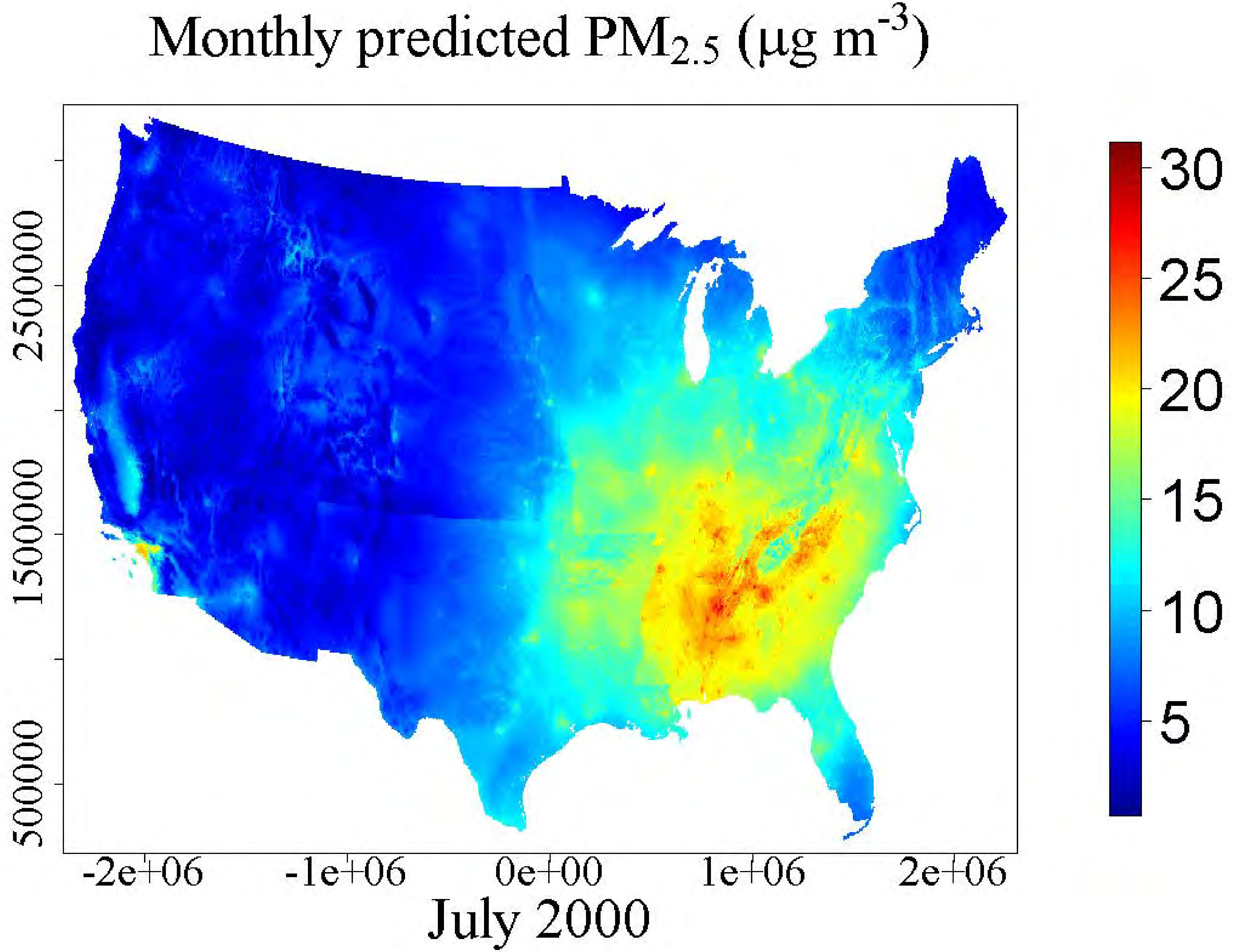

Monthly predicted PM2.5 (μg m-3)
25000000
15000000
5000000
-2e+06
-1e+06
0e+00
1e+06
2e+06
July 2000
30
25
20
15
10
5

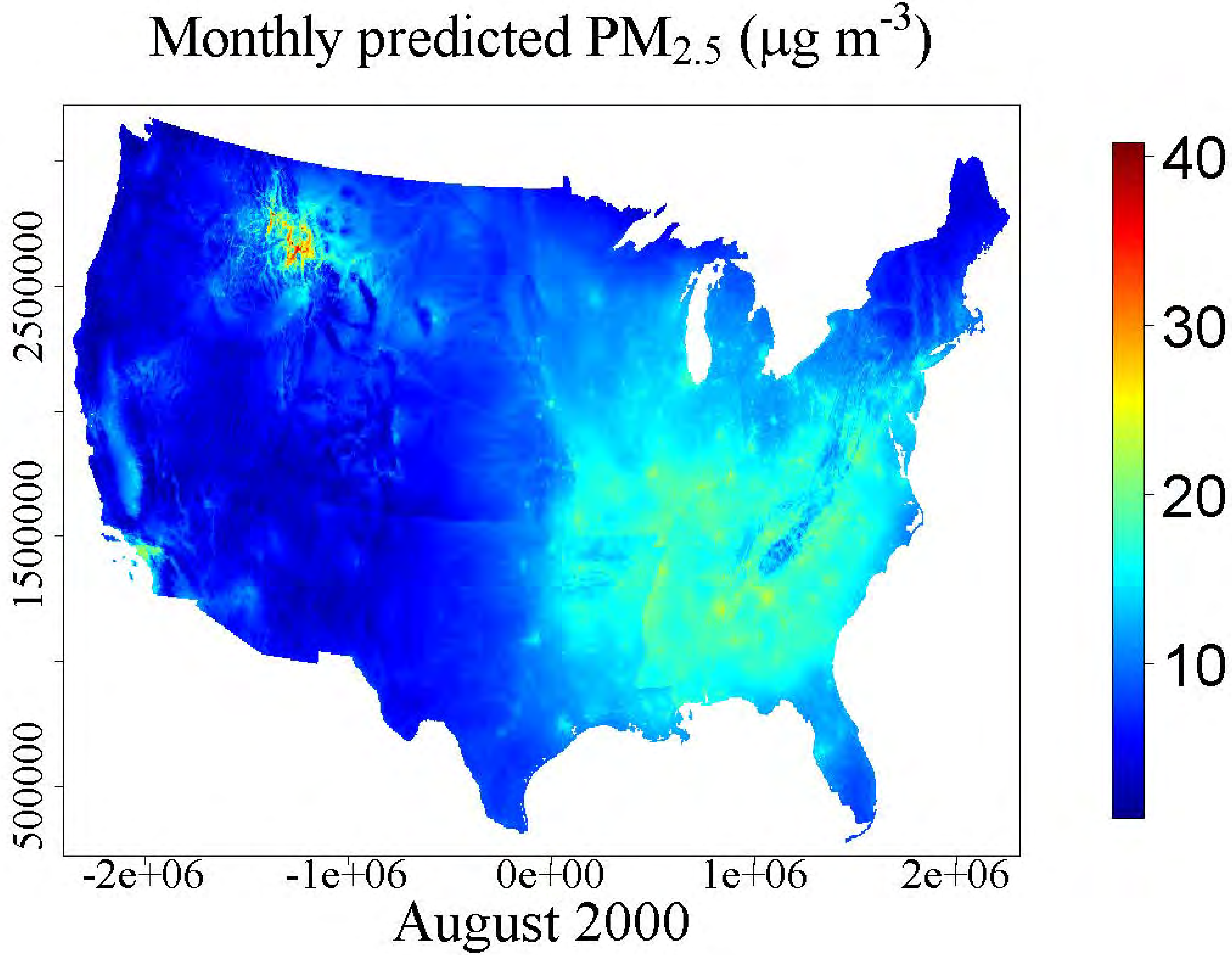

Monthly predicted PM2.5 (μg m-3)
25000000
15000000
5000000
-2e+06
-1e+06
0e+00
1e+06
2e+06
August 2000
40
30
20
10

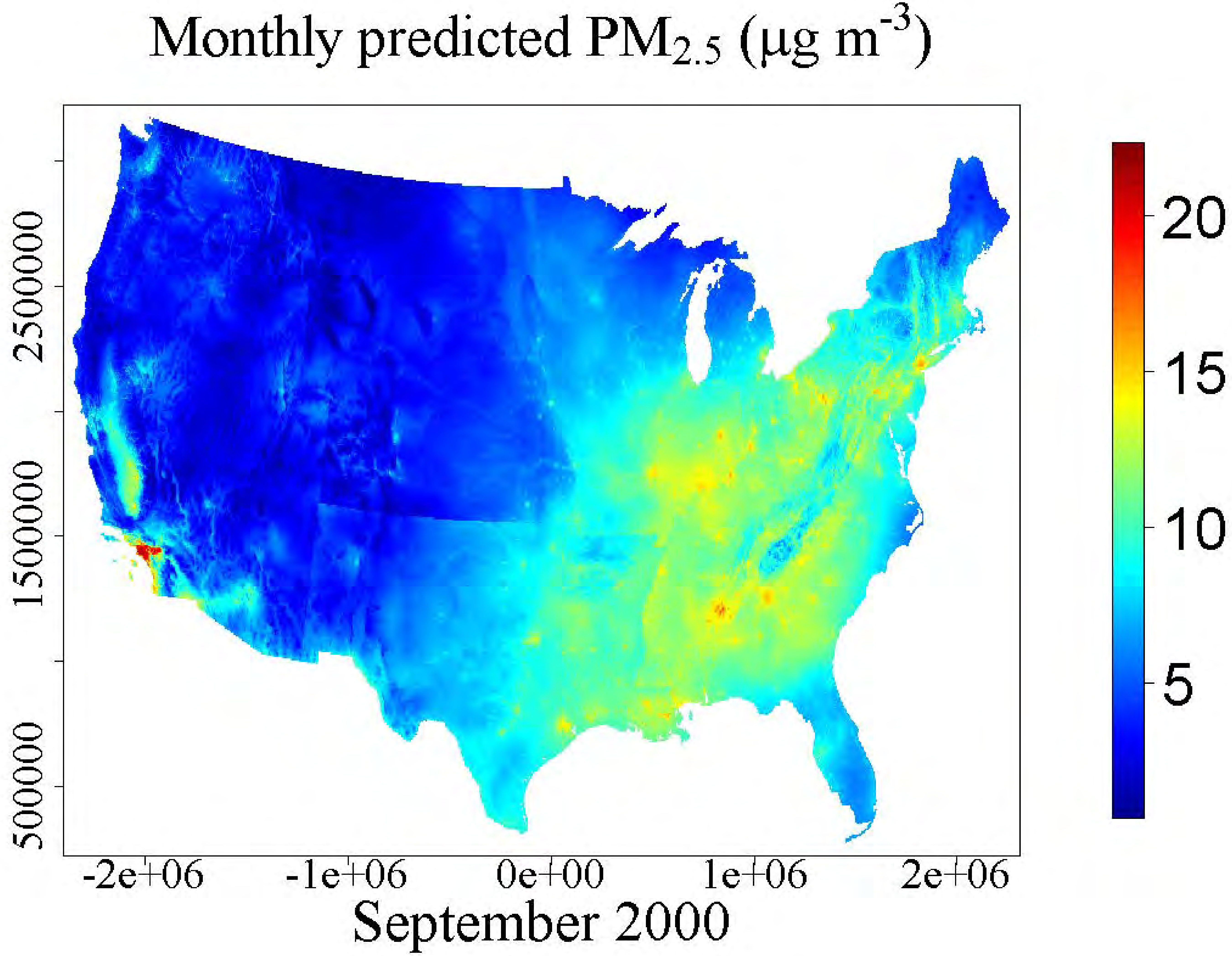
Monthly predicted $PM_{2.5}$ ($\mu g\ m^{-3}$)
25000000
15000000
5000000
-2e+06
-1e+06
0e+00
1e+06
2e+06
September 2000
20
15
10
5

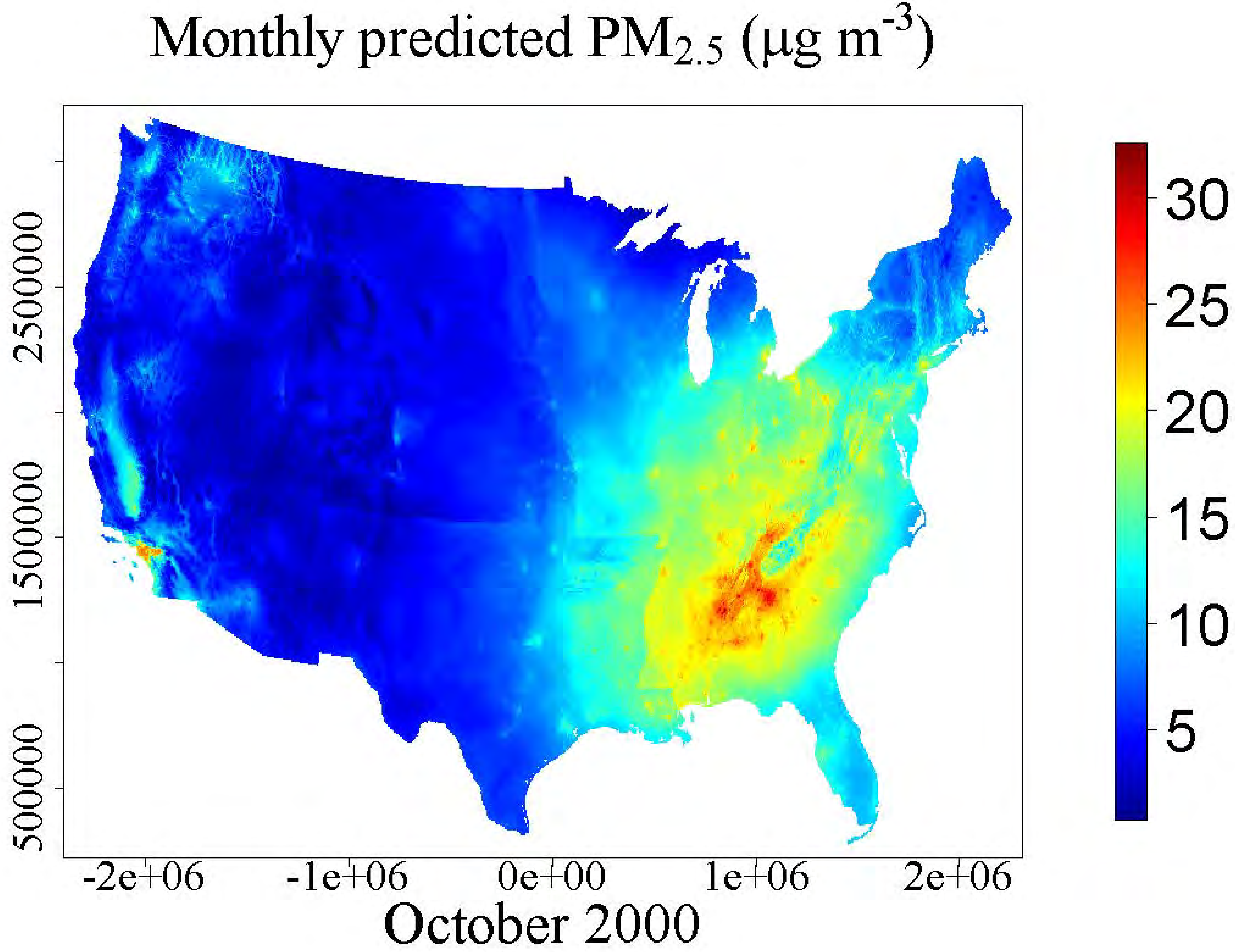
Monthly predicted PM2.5 (μg m-3)
25000000
15000000
5000000
-2e+06
-1e+06
0e+00
1e+06
2e+06
30
25
20
15
10
5
October 2000

Monthly predicted $PM_{2.5}$ ($\mu g\ m^{-3}$)

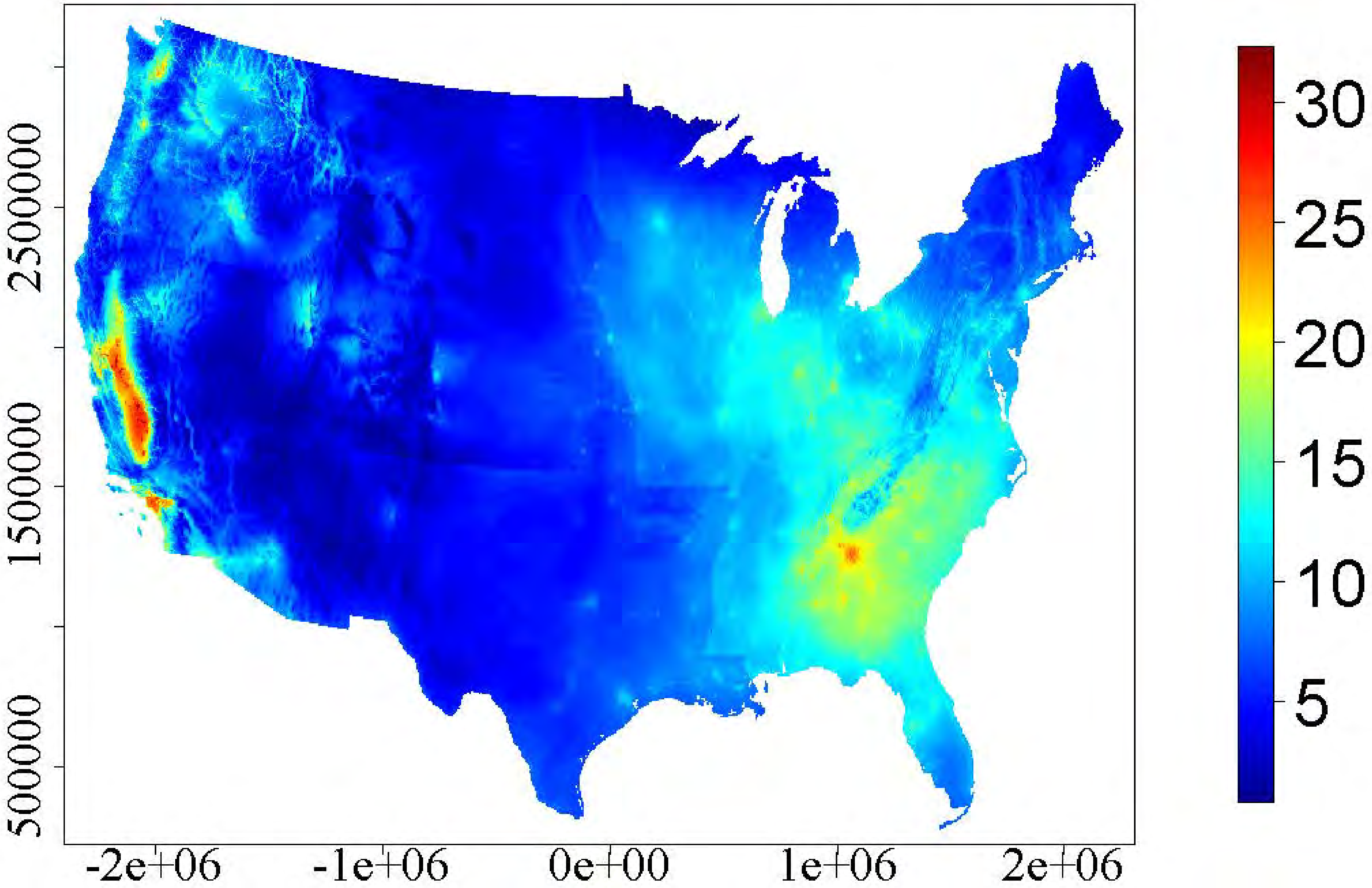

November 2000

Monthly predicted $PM_{2.5}$ ($\mu g\ m^{-3}$)

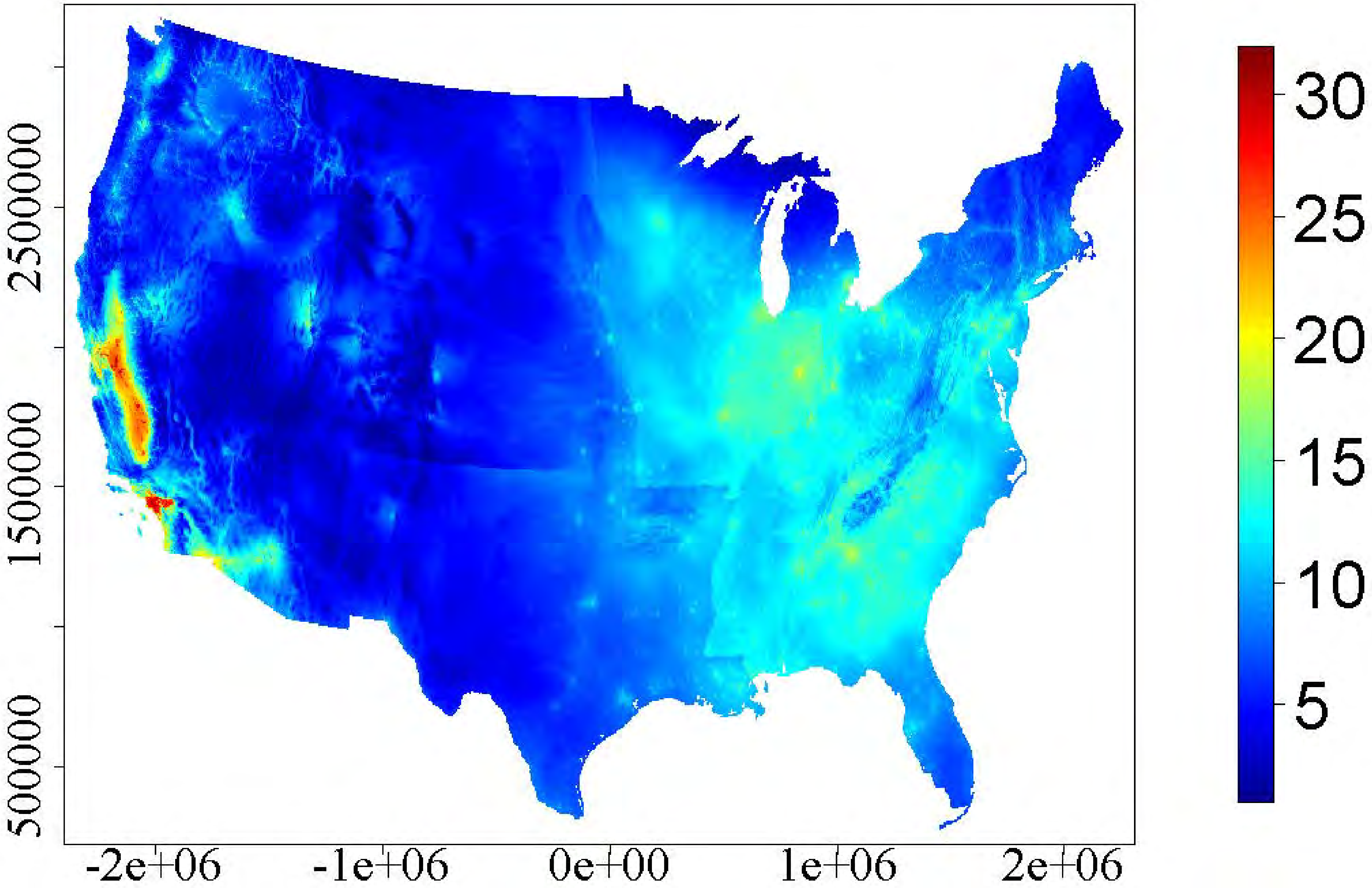

December 2000

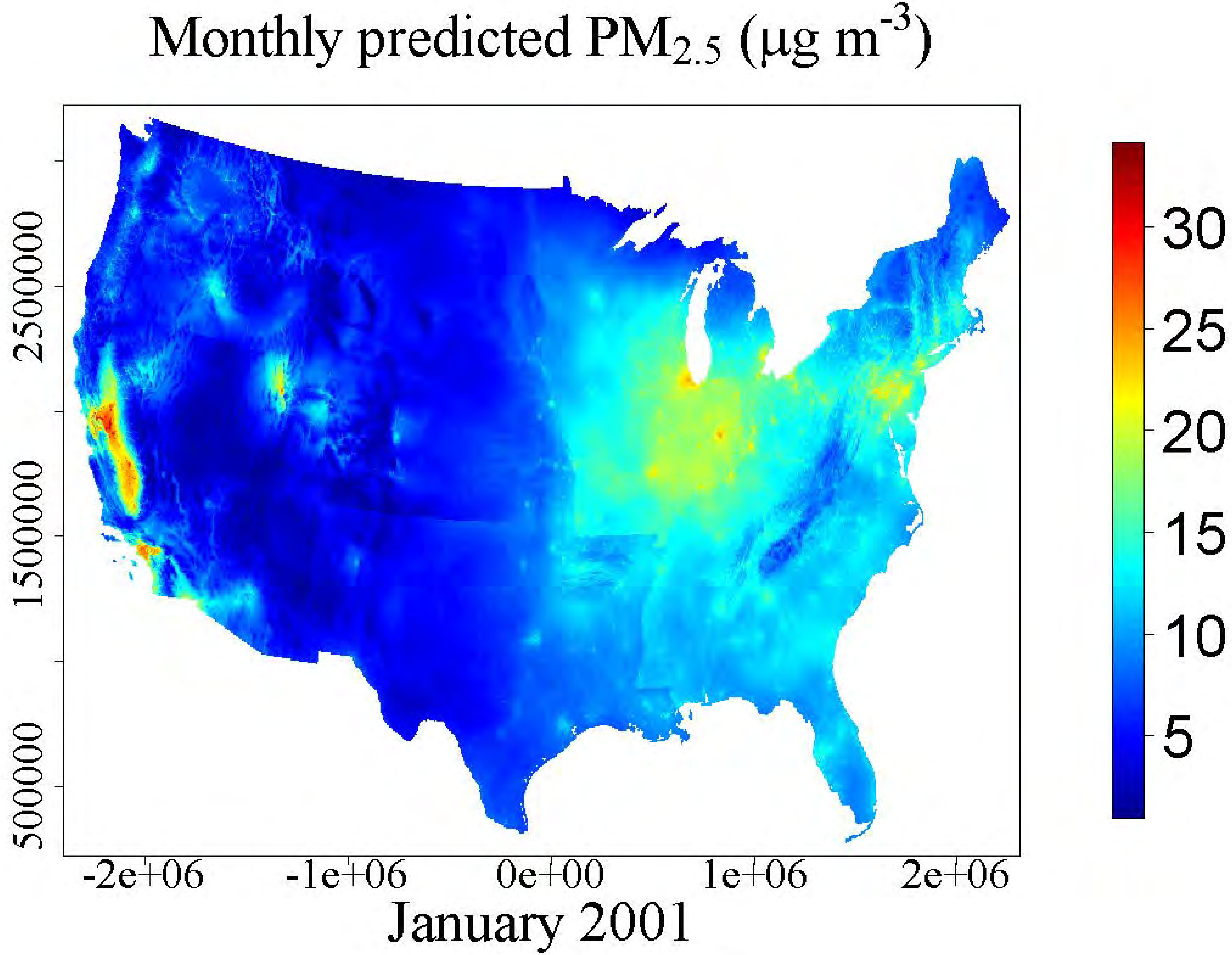

Monthly predicted PM2.5 (μg m-3)
25000000
15000000
5000000
-2e+06
-1e+06
0e+00
1e+06
2e+06
January 2001
30
25
20
15
10
5

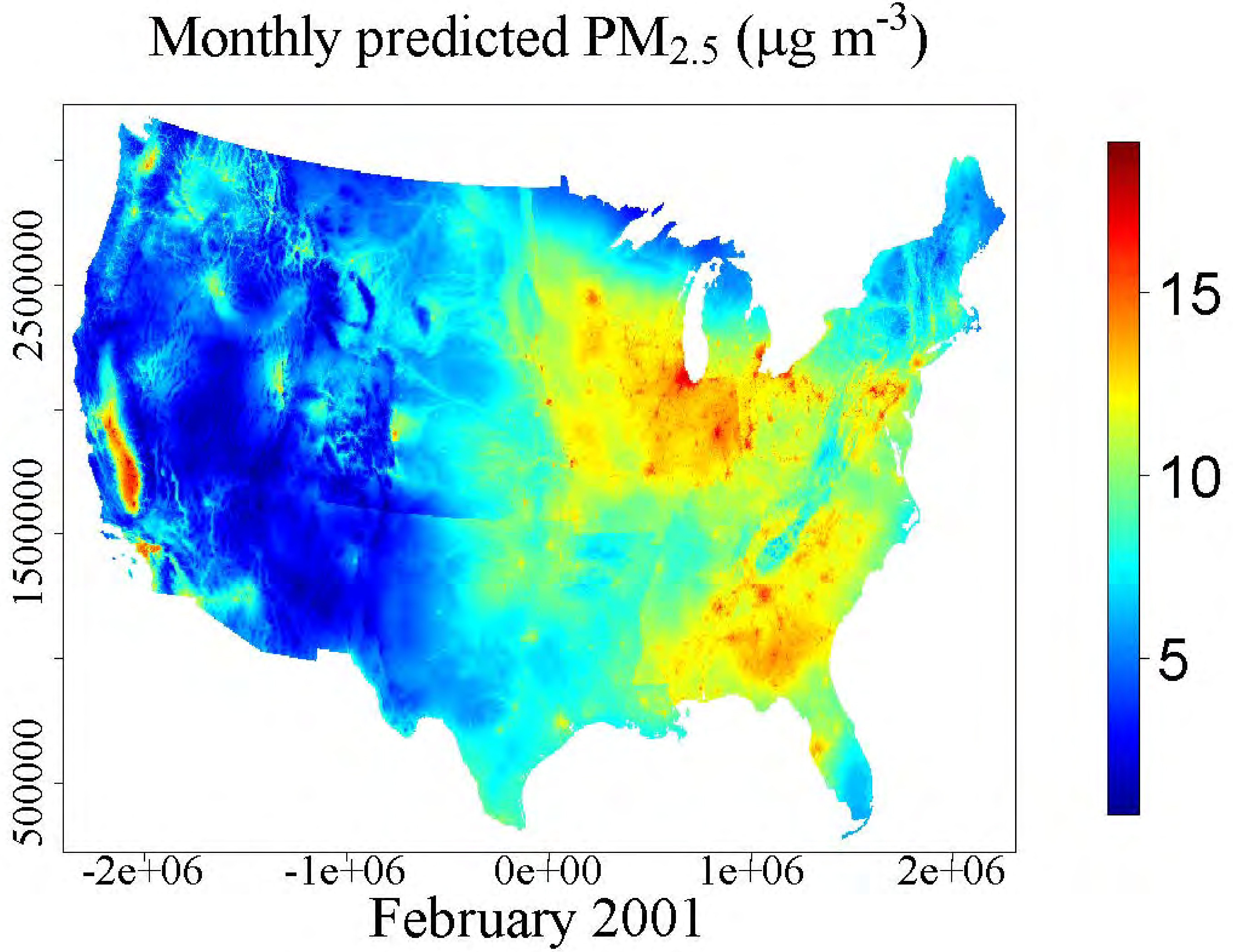

Monthly predicted PM2.5 (μg m-3)
25000000
15000000
5000000
-2e+06
-1e+06
0e+00
1e+06
2e+06
15
10
5
February 2001

Monthly predicted $PM_{2.5}$ ($\mu g\ m^{-3}$)

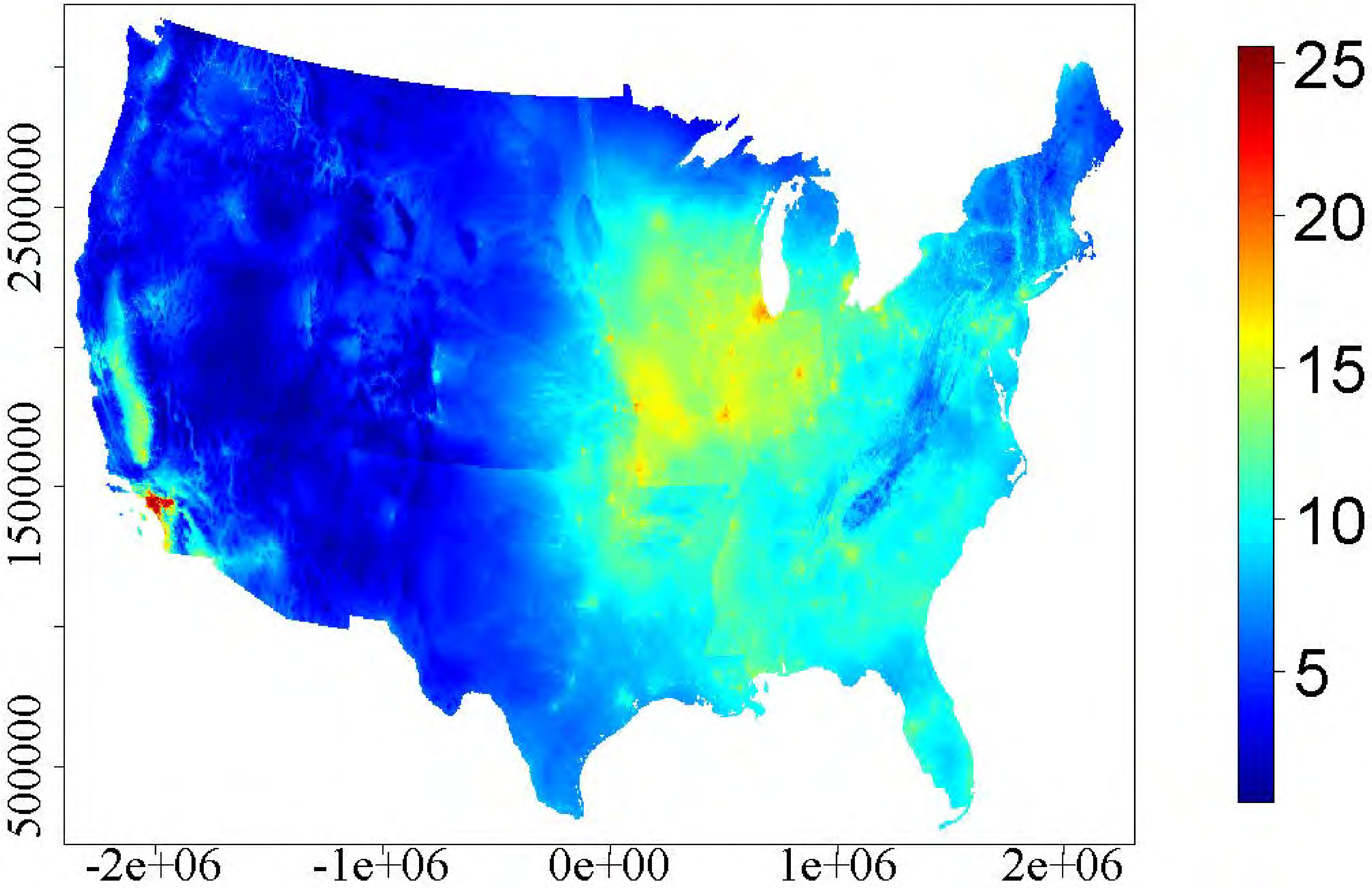

March 2001

Monthly predicted $PM_{2.5}$ ($\mu g\ m^{-3}$)

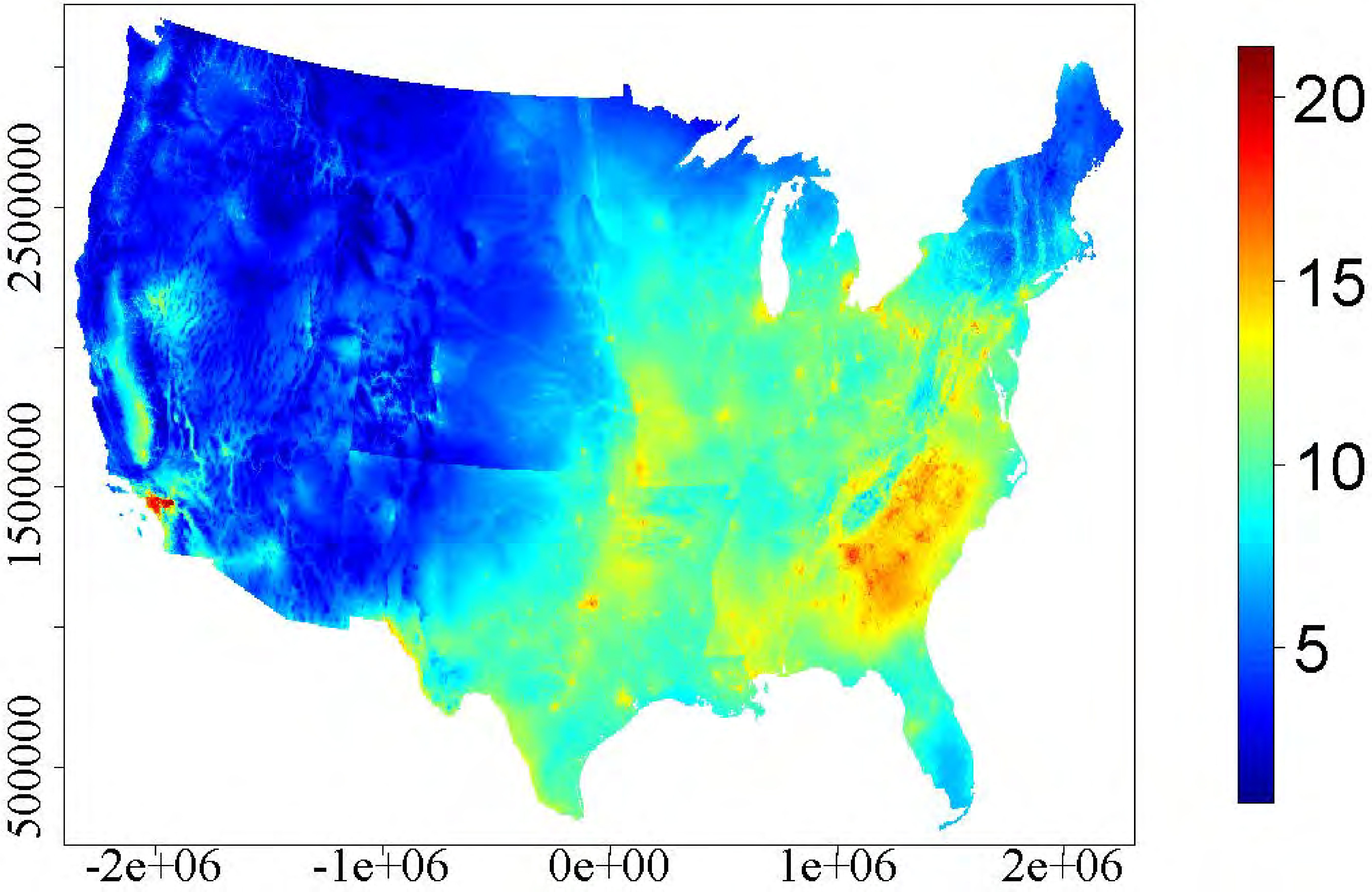

April 2001

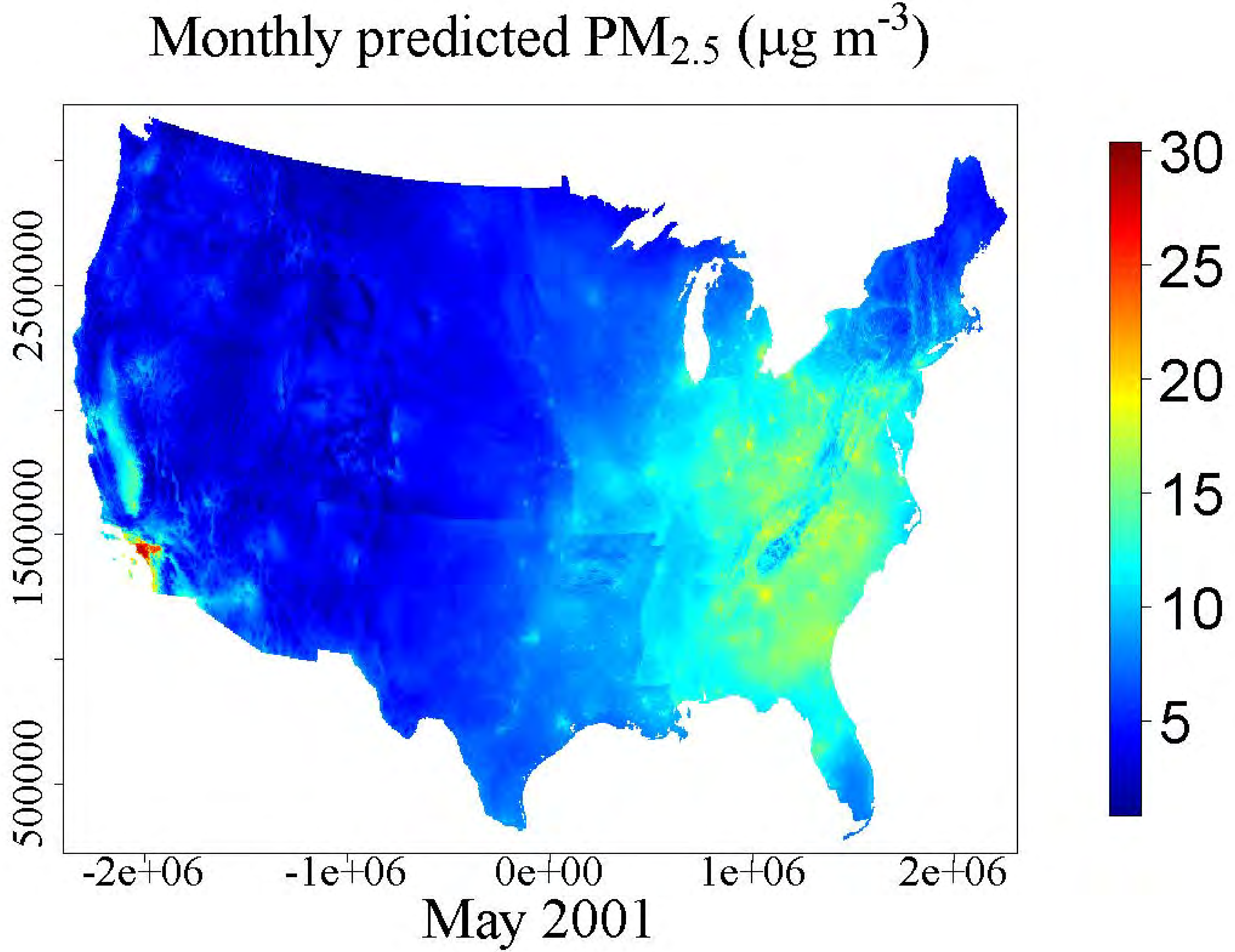
Monthly predicted PM2.5 (μg m-3)
25000000
15000000
5000000
-2e+06
-1e+06
0e+00
1e+06
2e+06
May 2001
30
25
20
15
10
5

Monthly predicted $PM_{2.5}$ ($\mu g\ m^{-3}$)

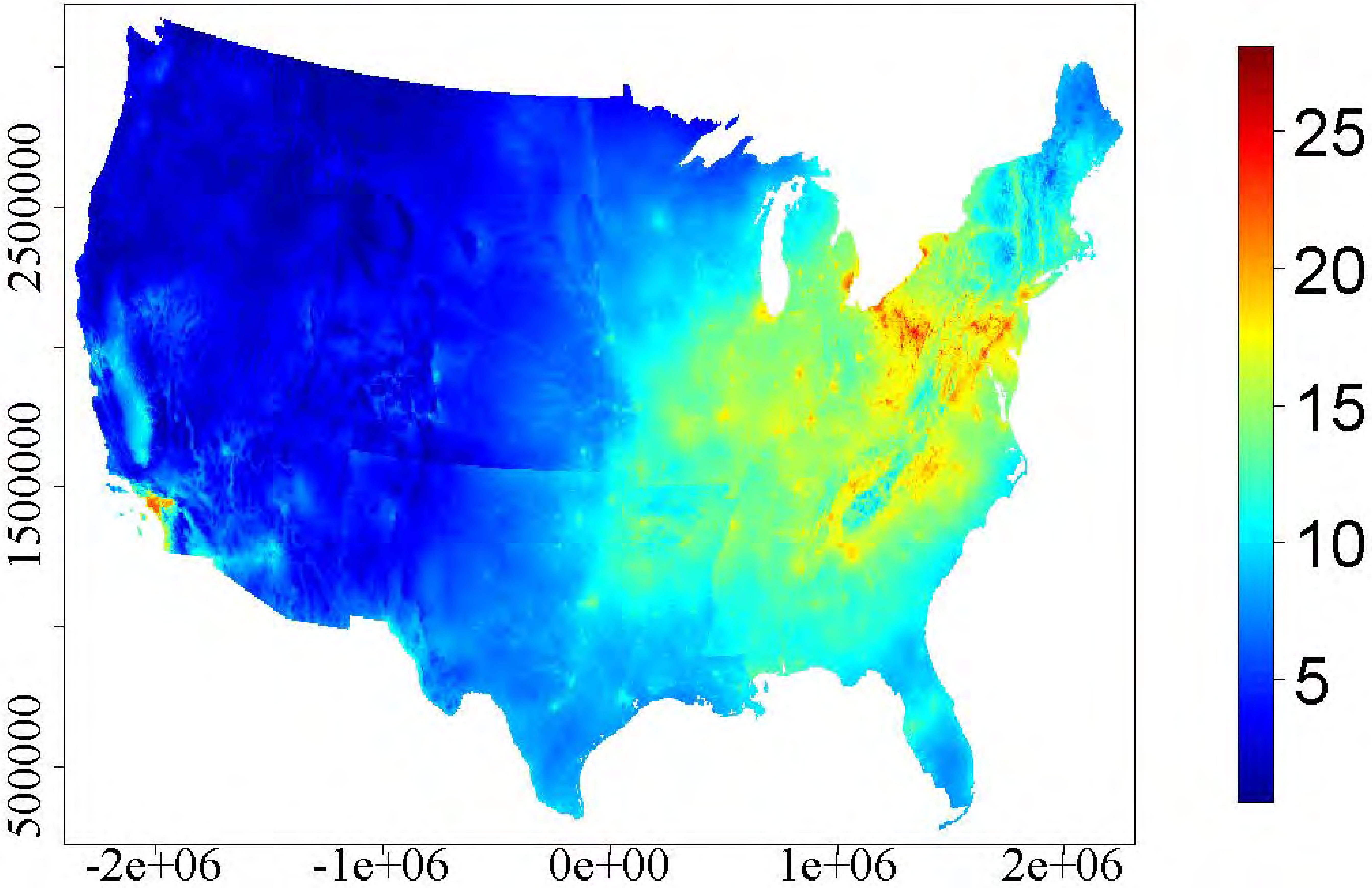

June 2001

Monthly predicted $PM_{2.5}$ ($\mu g\ m^{-3}$)

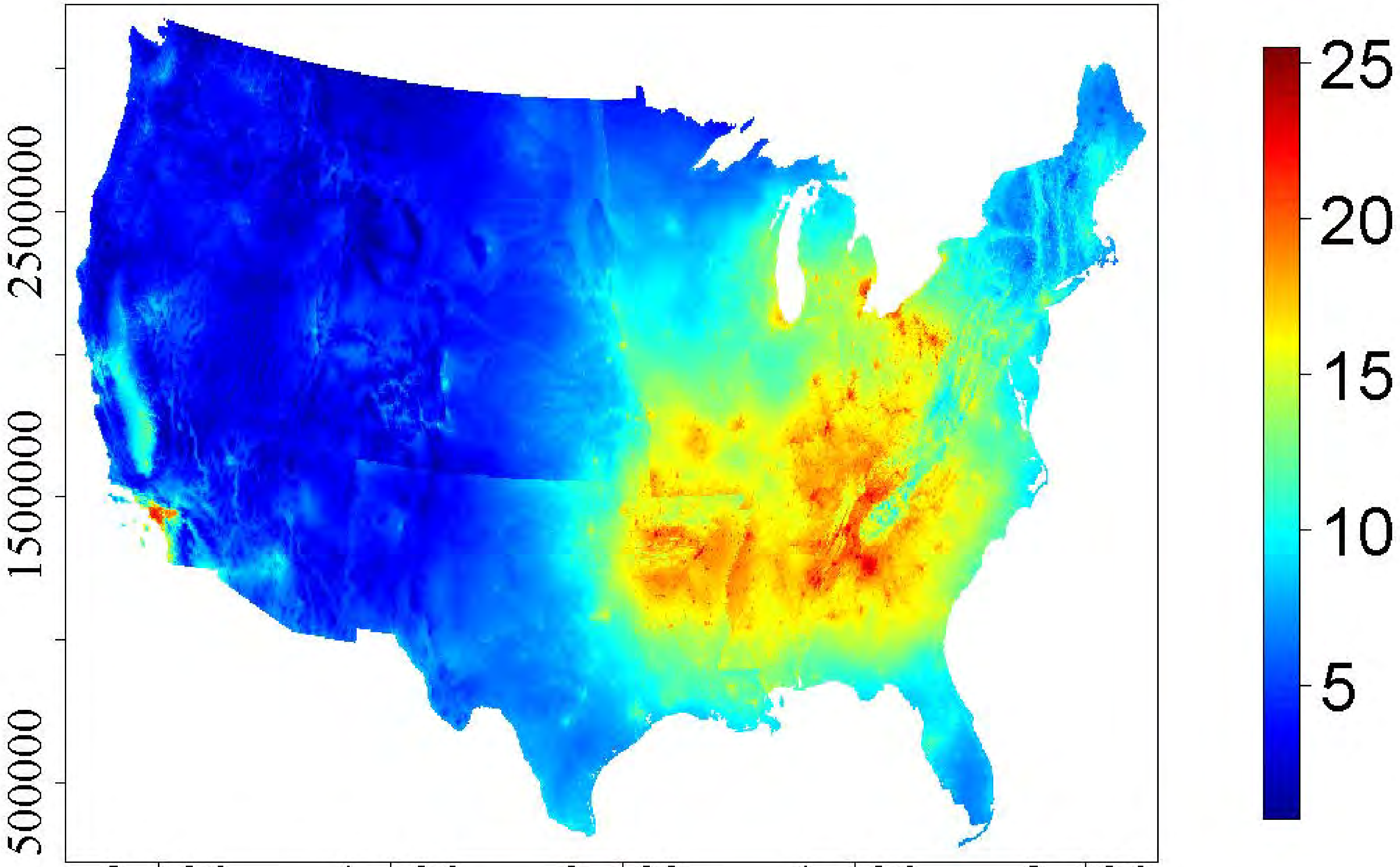

July 2001

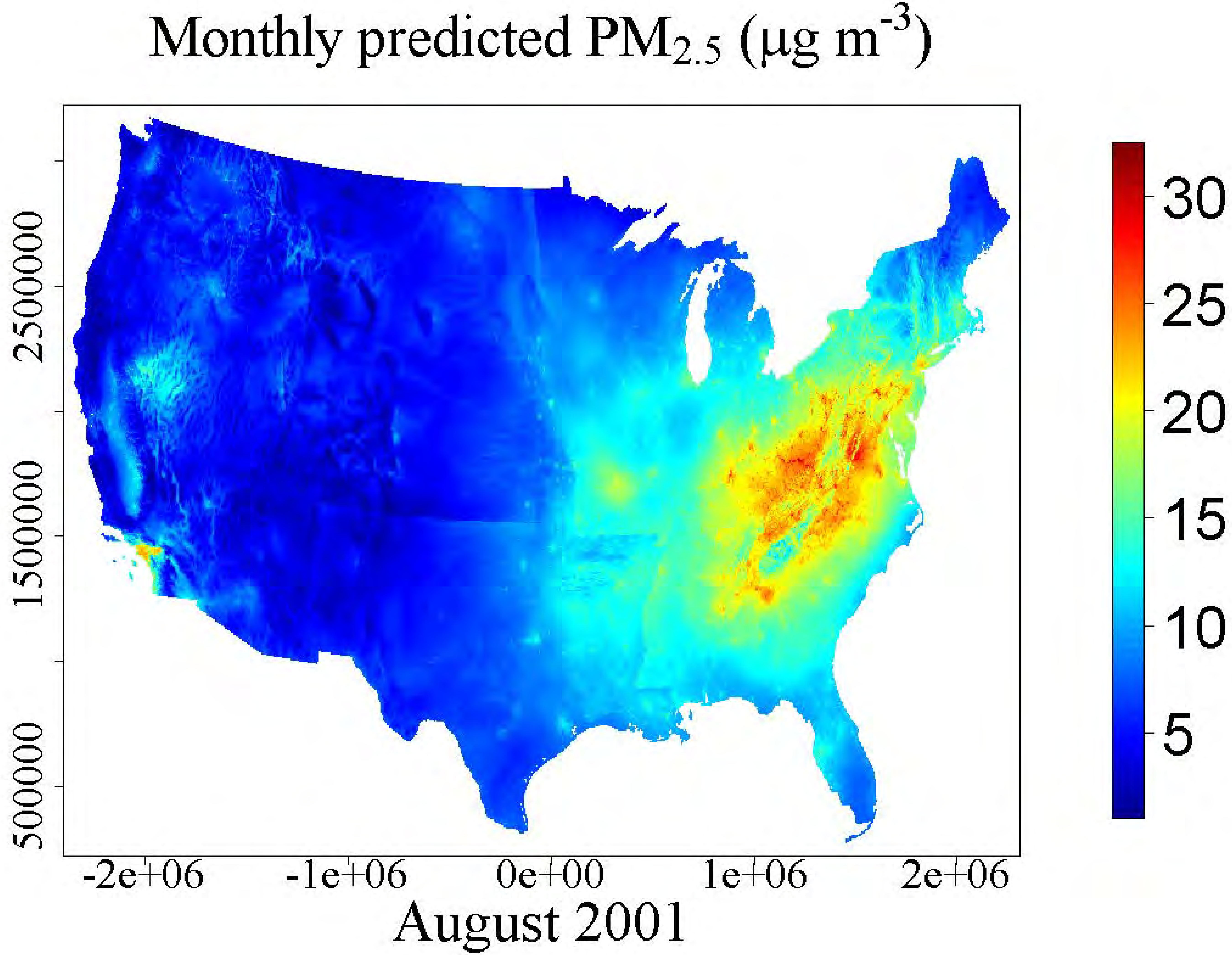

Monthly predicted $PM_{2.5}$ ($\mu g\ m^{-3}$)
25000000
15000000
5000000
-2e+06
-1e+06
0e+00
1e+06
2e+06
August 2001
30
25
20
15
10
5

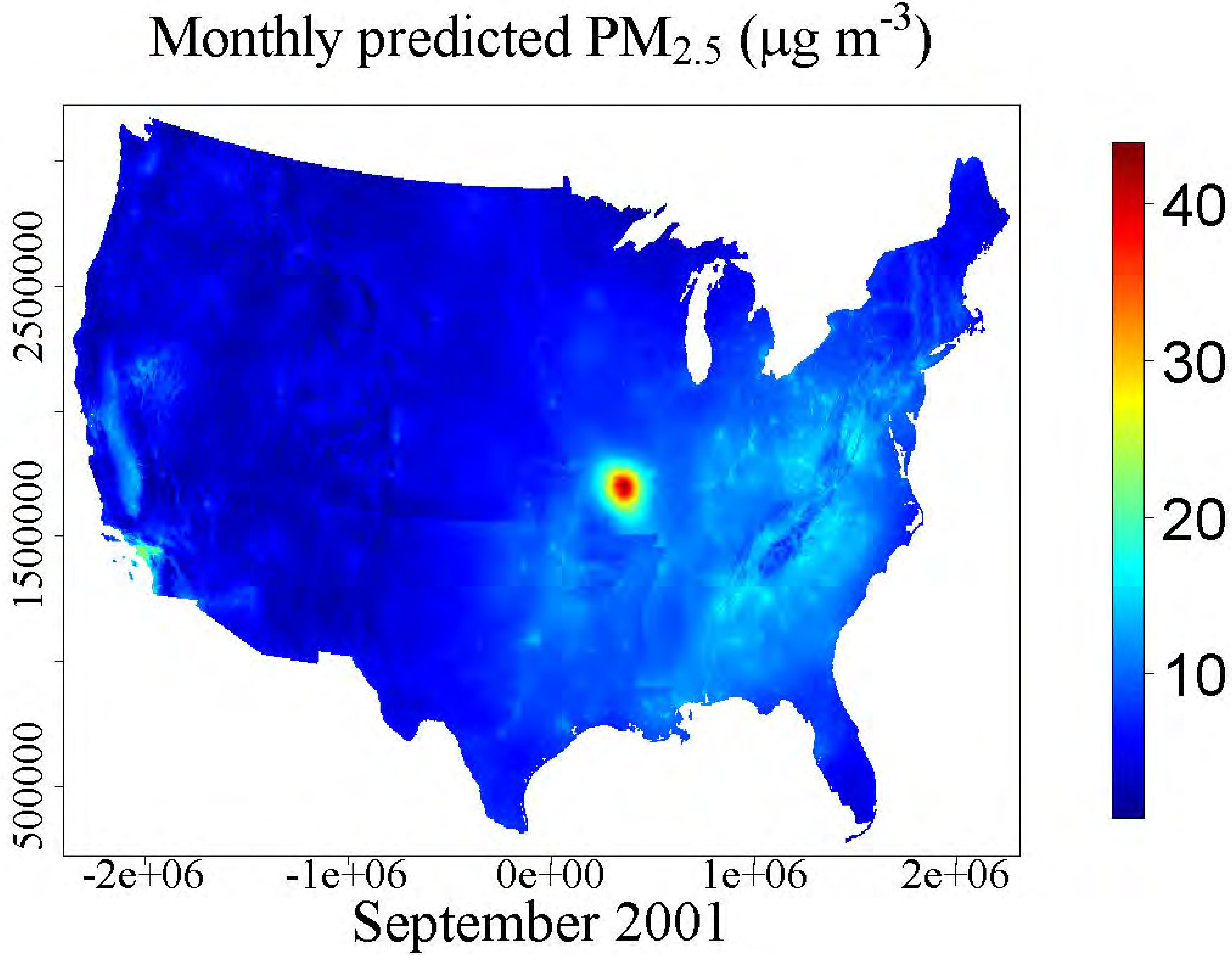

Monthly predicted $PM_{2.5}$ ($\mu g\ m^{-3}$)
25000000
15000000
5000000
-2e+06
-1e+06
0e+00
1e+06
2e+06
40
30
20
10
September 2001

Monthly predicted $PM_{2.5}$ ($\mu g\ m^{-3}$)

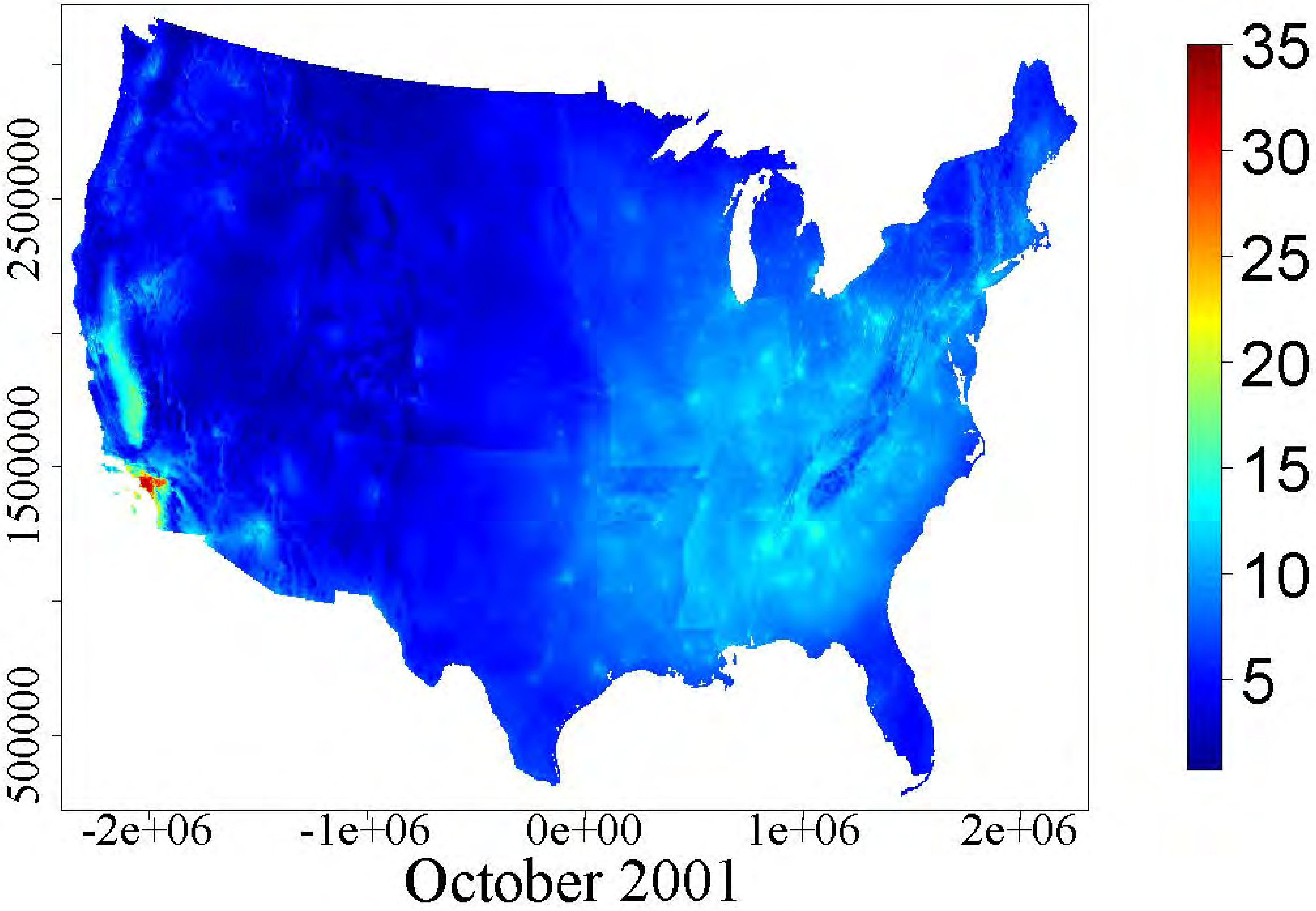

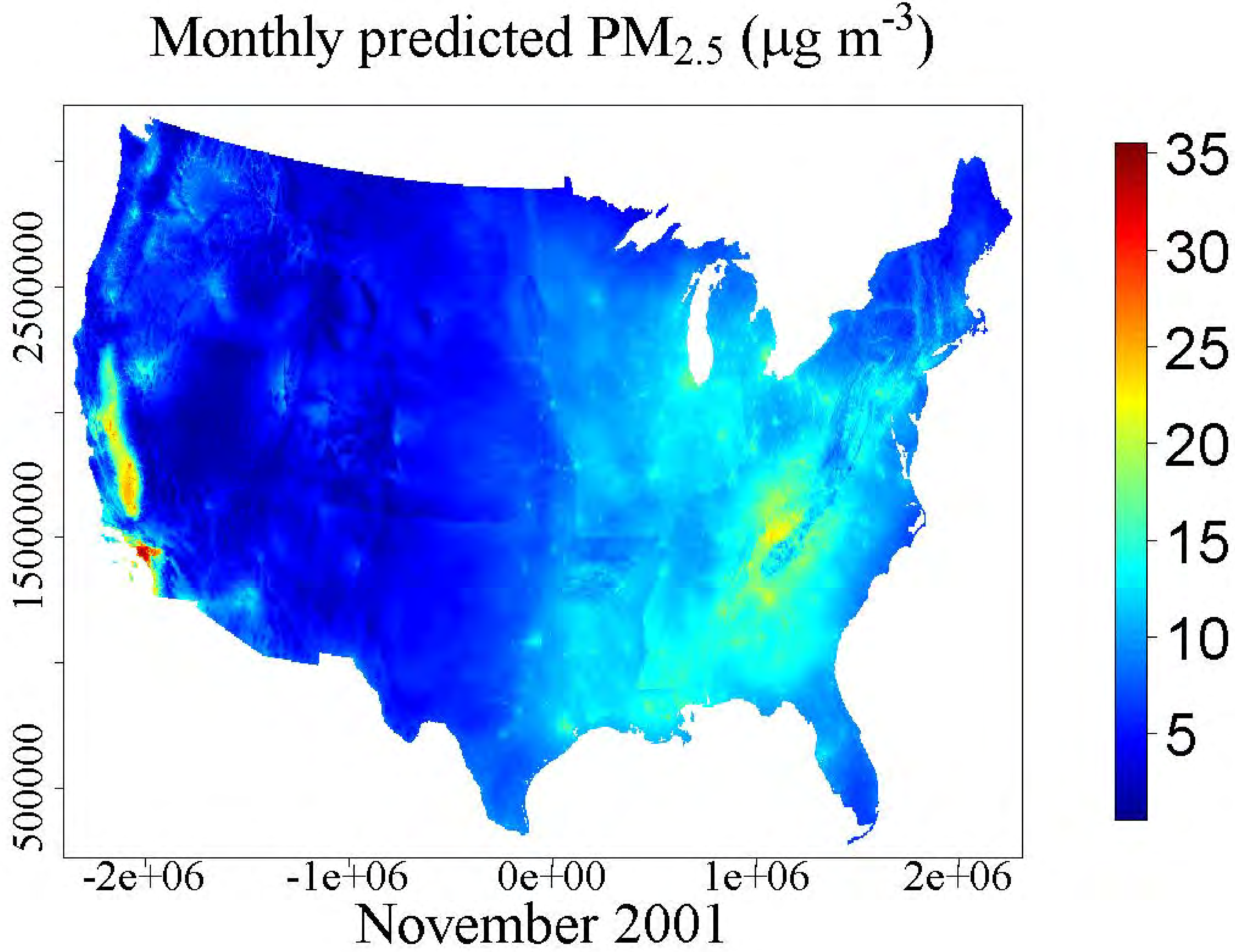

Monthly predicted $PM_{2.5}$ ($\mu g\ m^{-3}$)
5000000
15000000
25000000
-2e+06
-1e+06
0e+00
1e+06
2e+06
35
30
25
20
15
10
5
November 2001

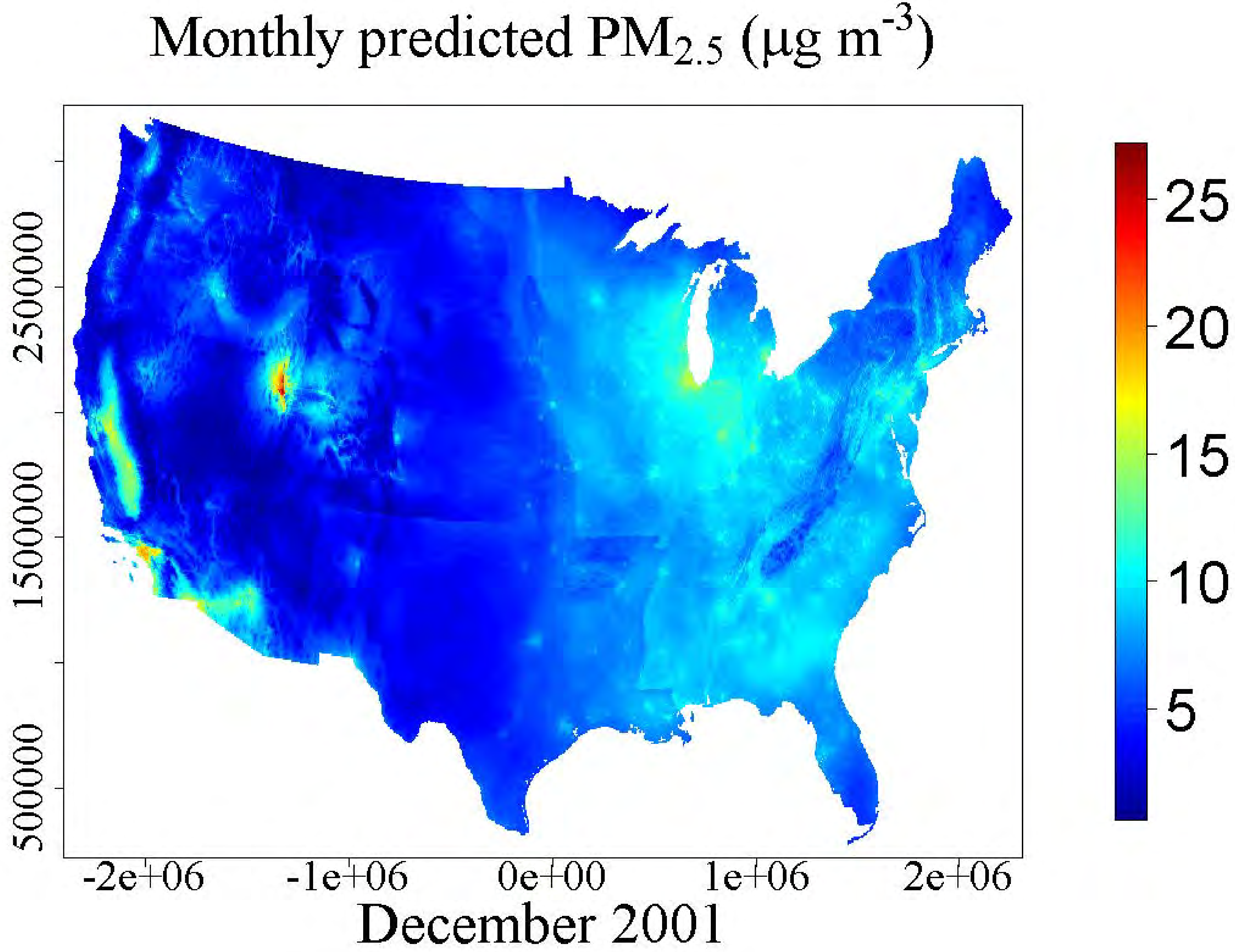
Monthly predicted PM2.5 (μg m-3)
2500000
1500000
500000
-2e+06
-1e+06
0e+00
1e+06
2e+06
25
20
15
10
5
December 2001

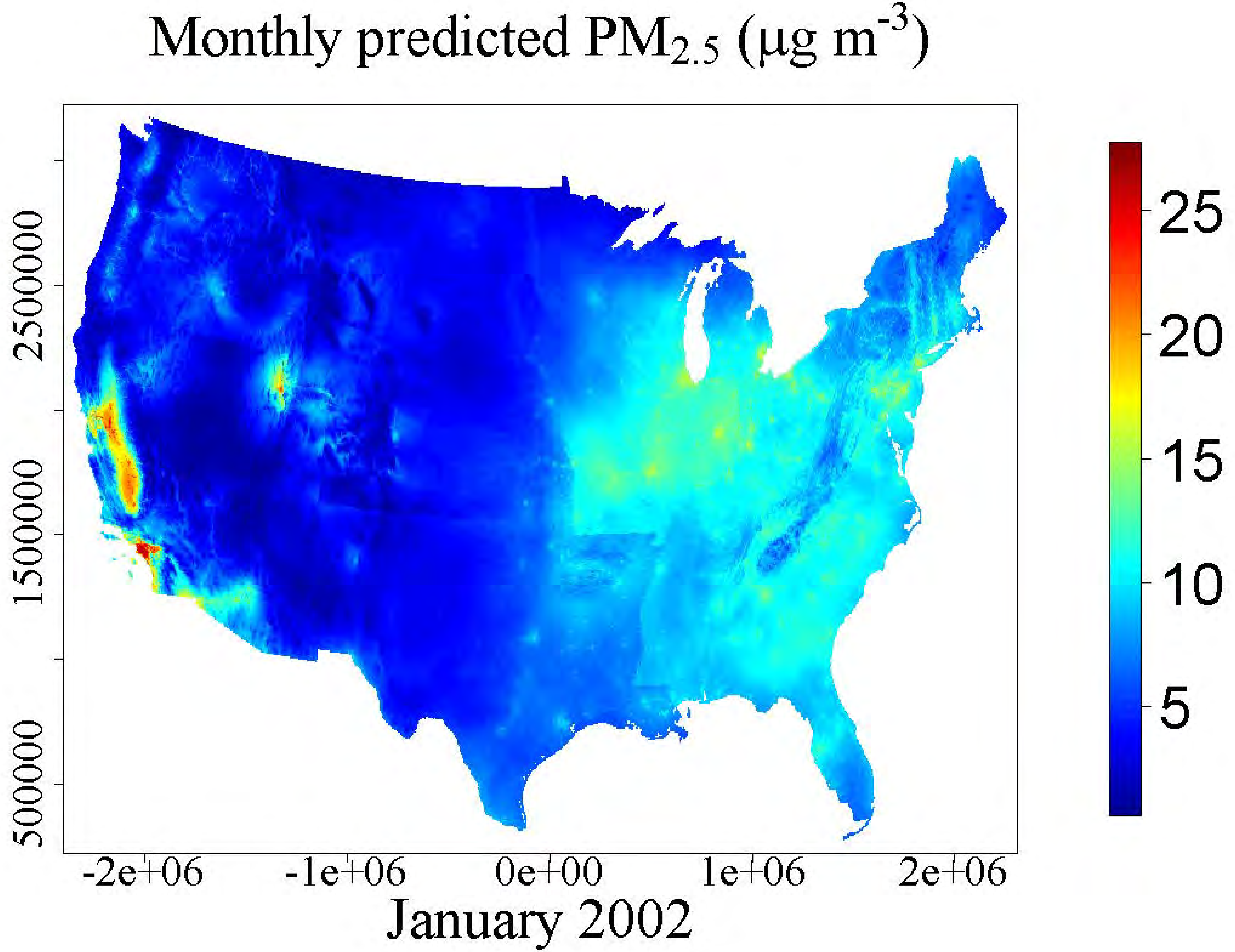

Monthly predicted PM2.5 (μg m-3)
25000000
15000000
5000000
-2e+06
-1e+06
0e+00
1e+06
2e+06
January 2002
25
20
15
10
5

Monthly predicted $PM_{2.5}$ ($\mu g\ m^{-3}$)

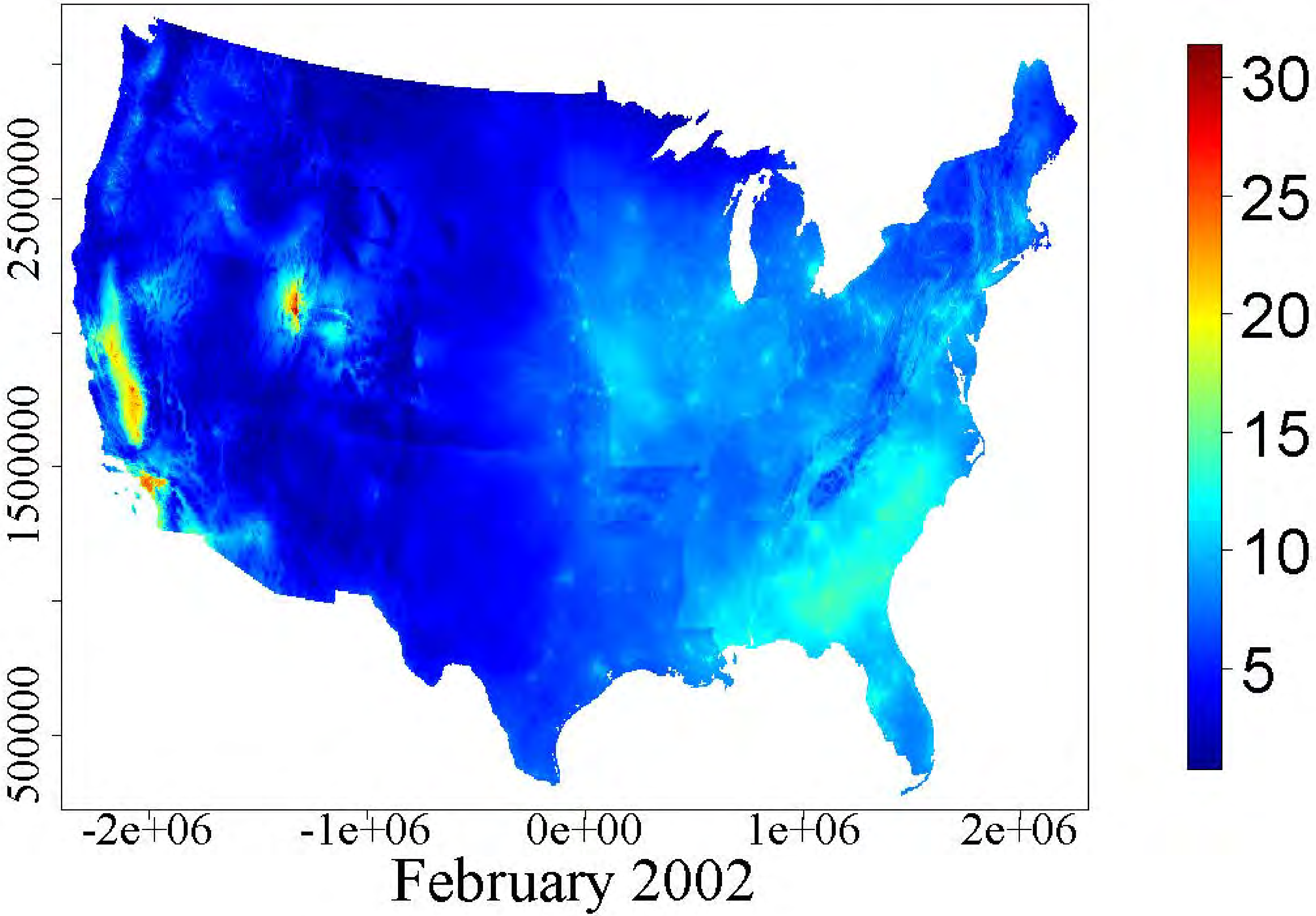

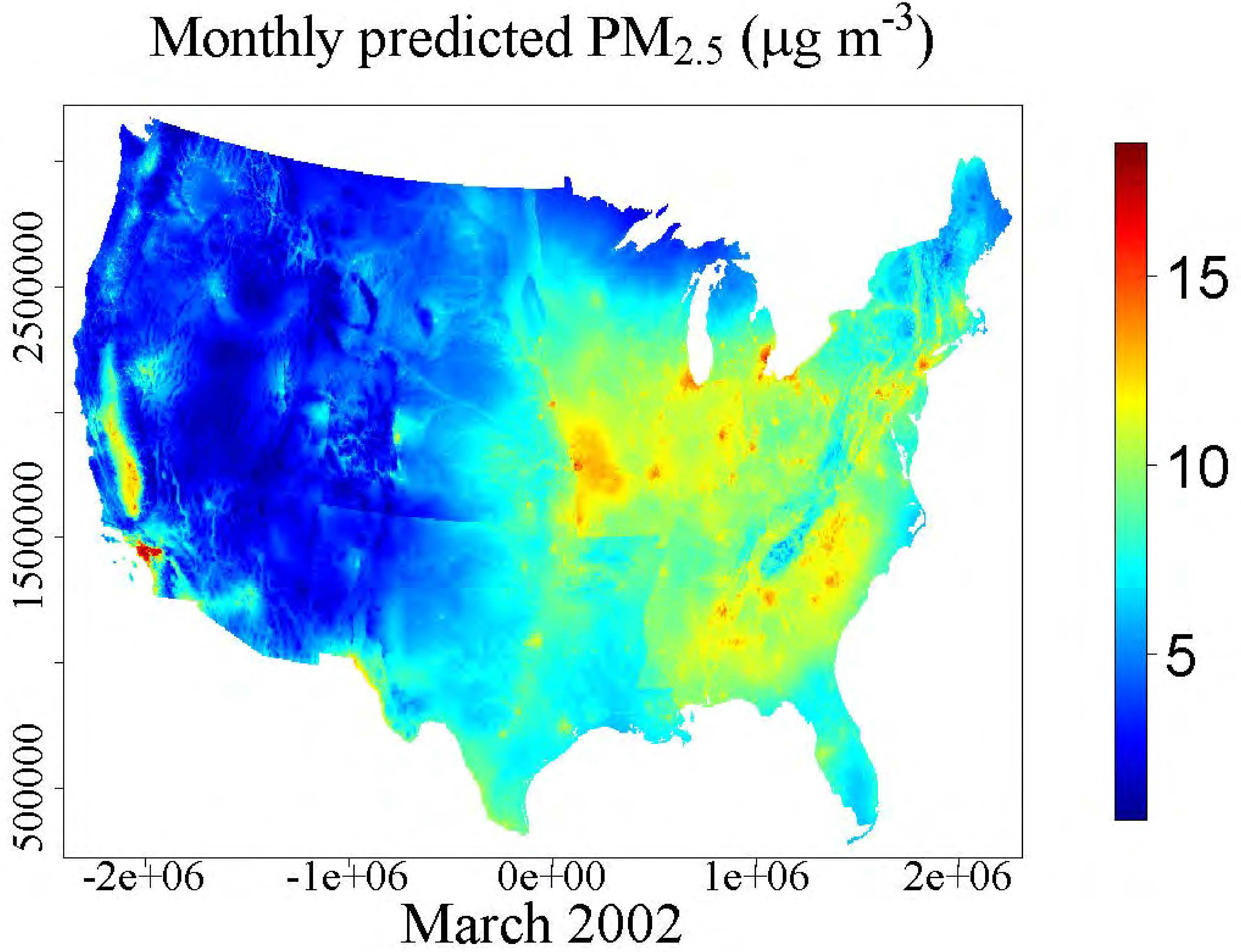
Monthly predicted PM2.5 (µg m-3)
25000000
15000000
5000000
-2e+06
-1e+06
0e+00
1e+06
2e+06
15
10
5
March 2002

Monthly predicted $PM_{2.5}$ ($\mu g\ m^{-3}$)

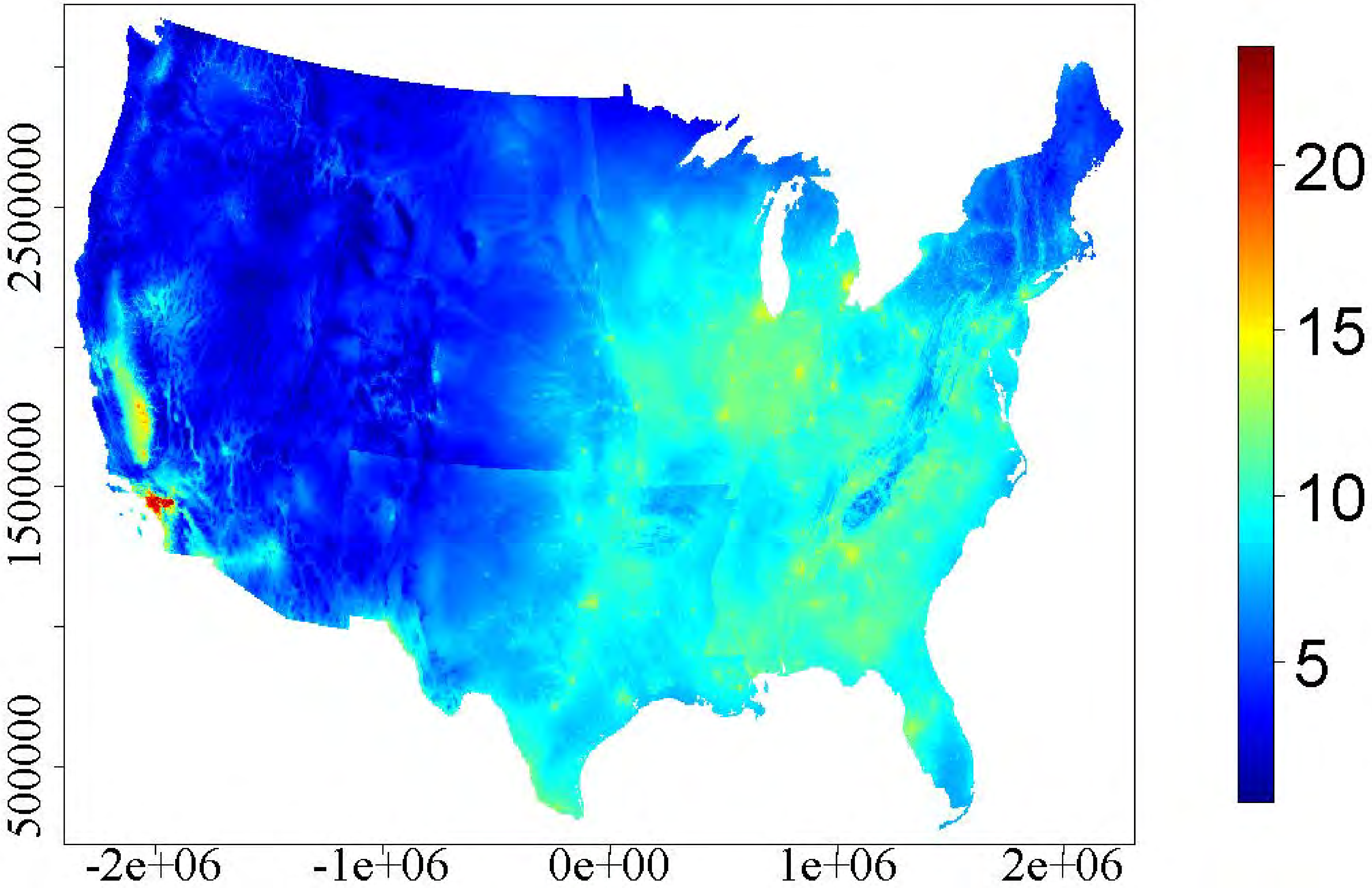

April 2002

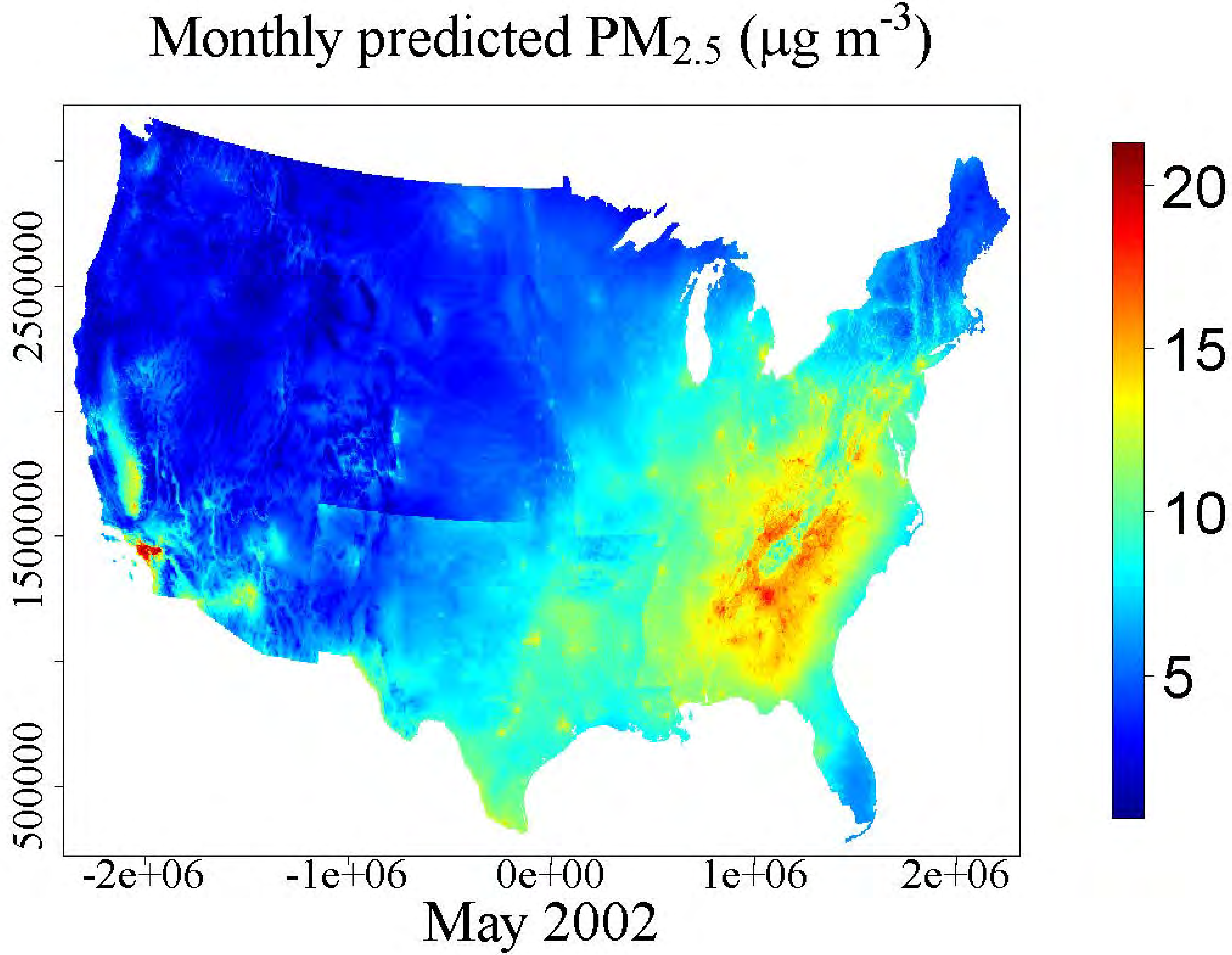
Monthly predicted $PM_{2.5}$ ($\mu g\ m^{-3}$)
25000000
15000000
5000000
-2e+06
-1e+06
0e+00
1e+06
2e+06
May 2002
20
15
10
5

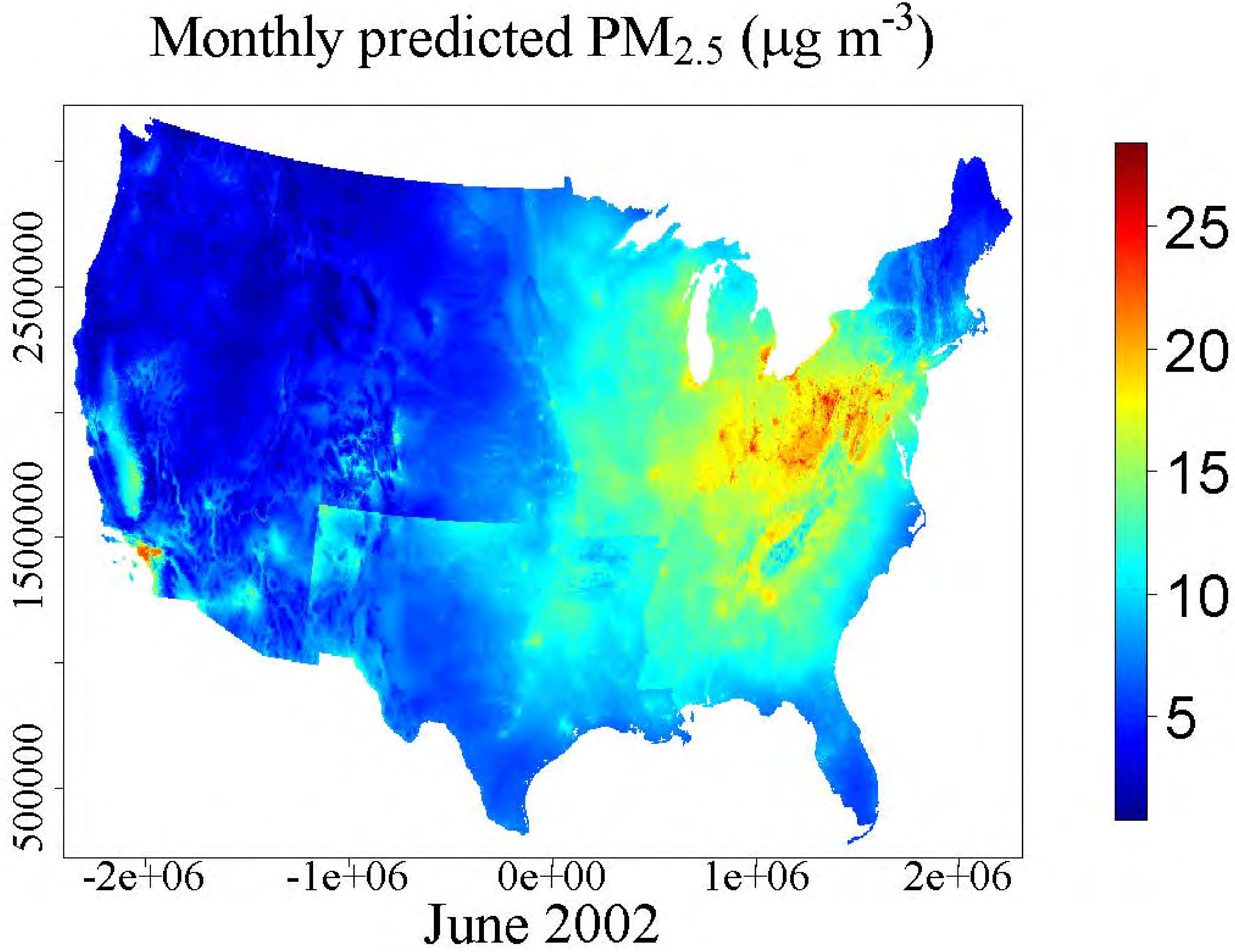
Monthly predicted $PM_{2.5}$ ($\mu g\ m^{-3}$)
25000000
15000000
5000000
-2e+06
-1e+06
0e+00
1e+06
2e+06
June 2002
25
20
15
10
5

Monthly predicted $PM_{2.5}$ ($\mu g\ m^{-3}$)

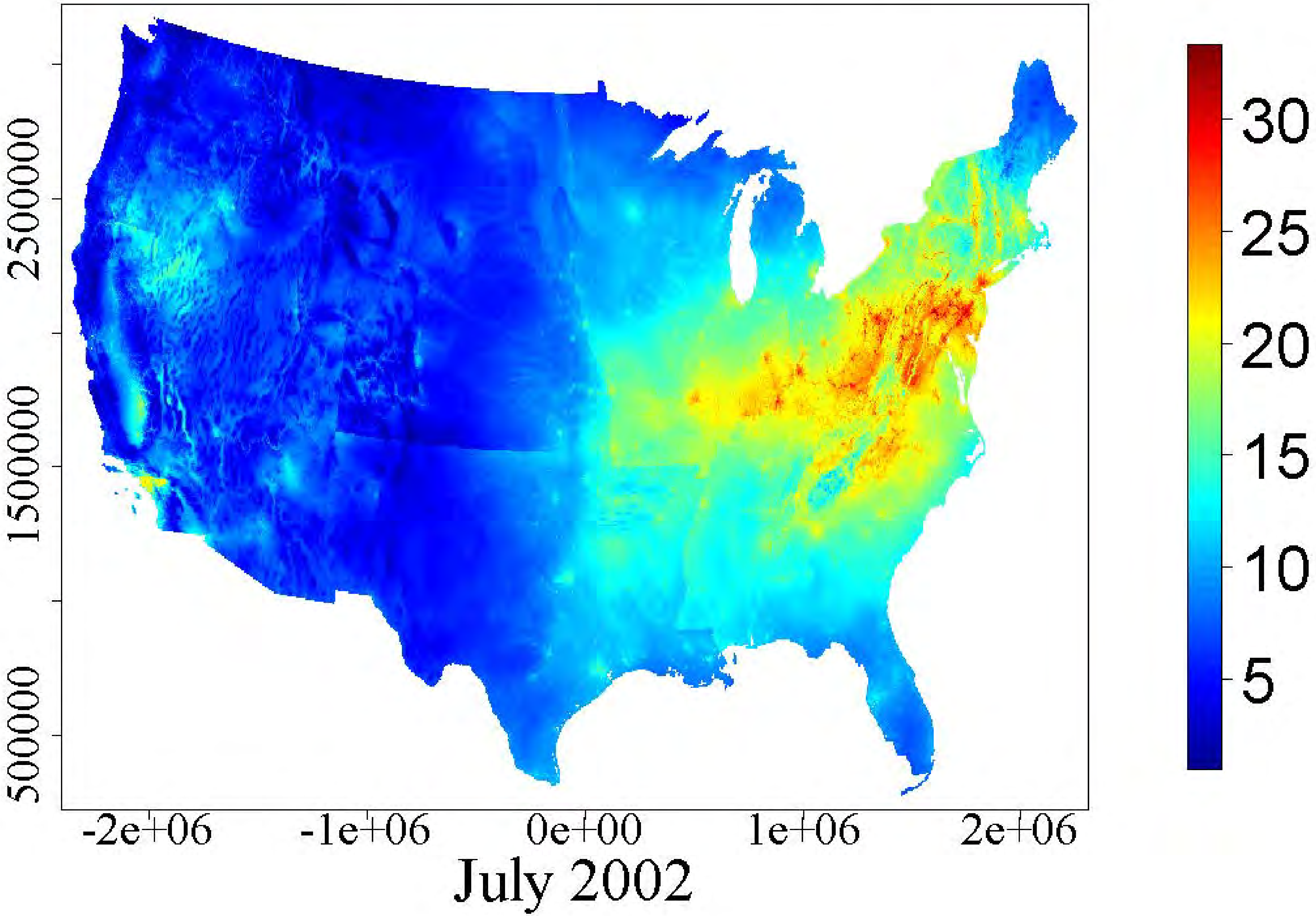

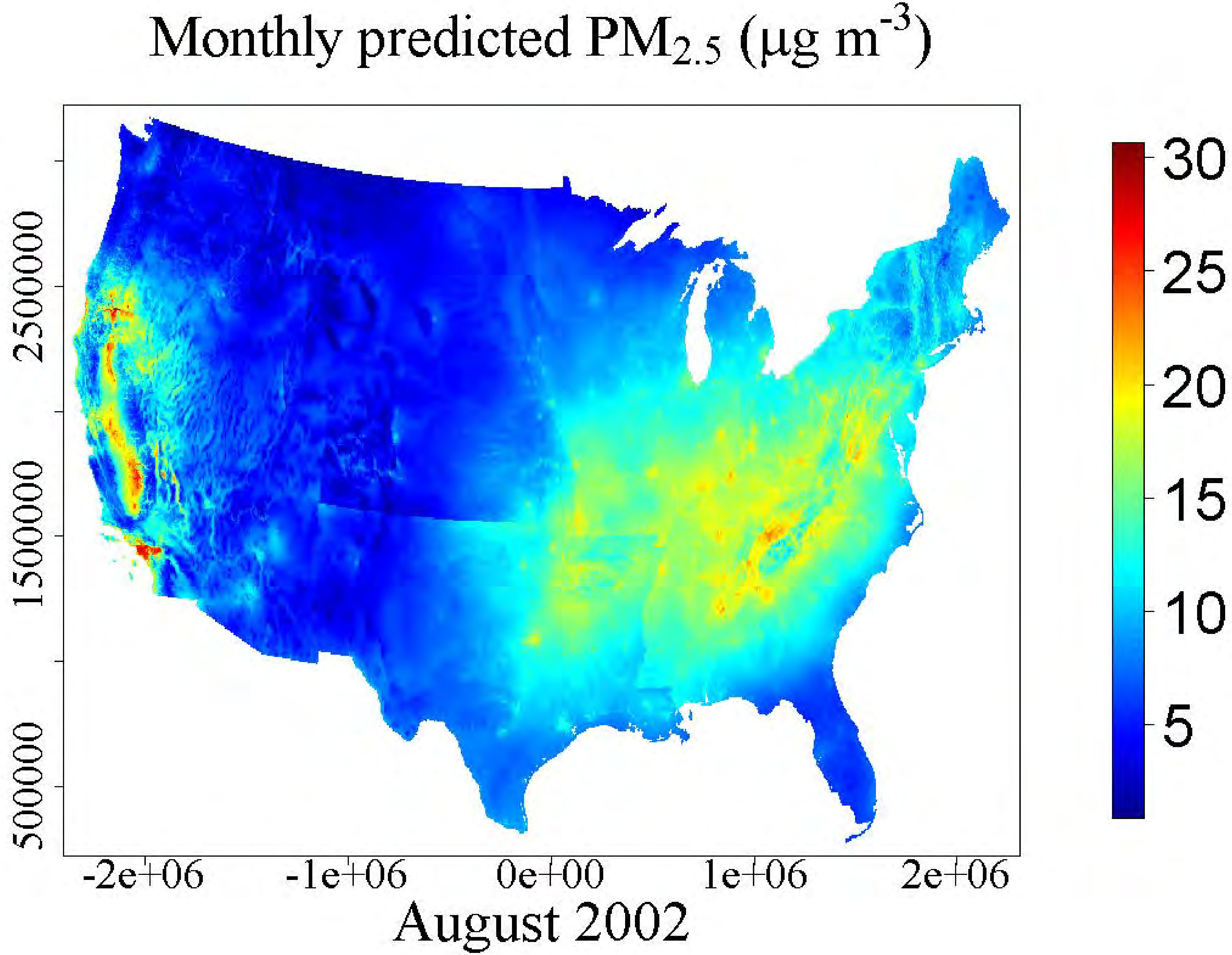
Monthly predicted PM2.5 (μg m-3)
25000000
15000000
5000000
-2e+06
-1e+06
0e+00
1e+06
2e+06
30
25
20
15
10
5
August 2002

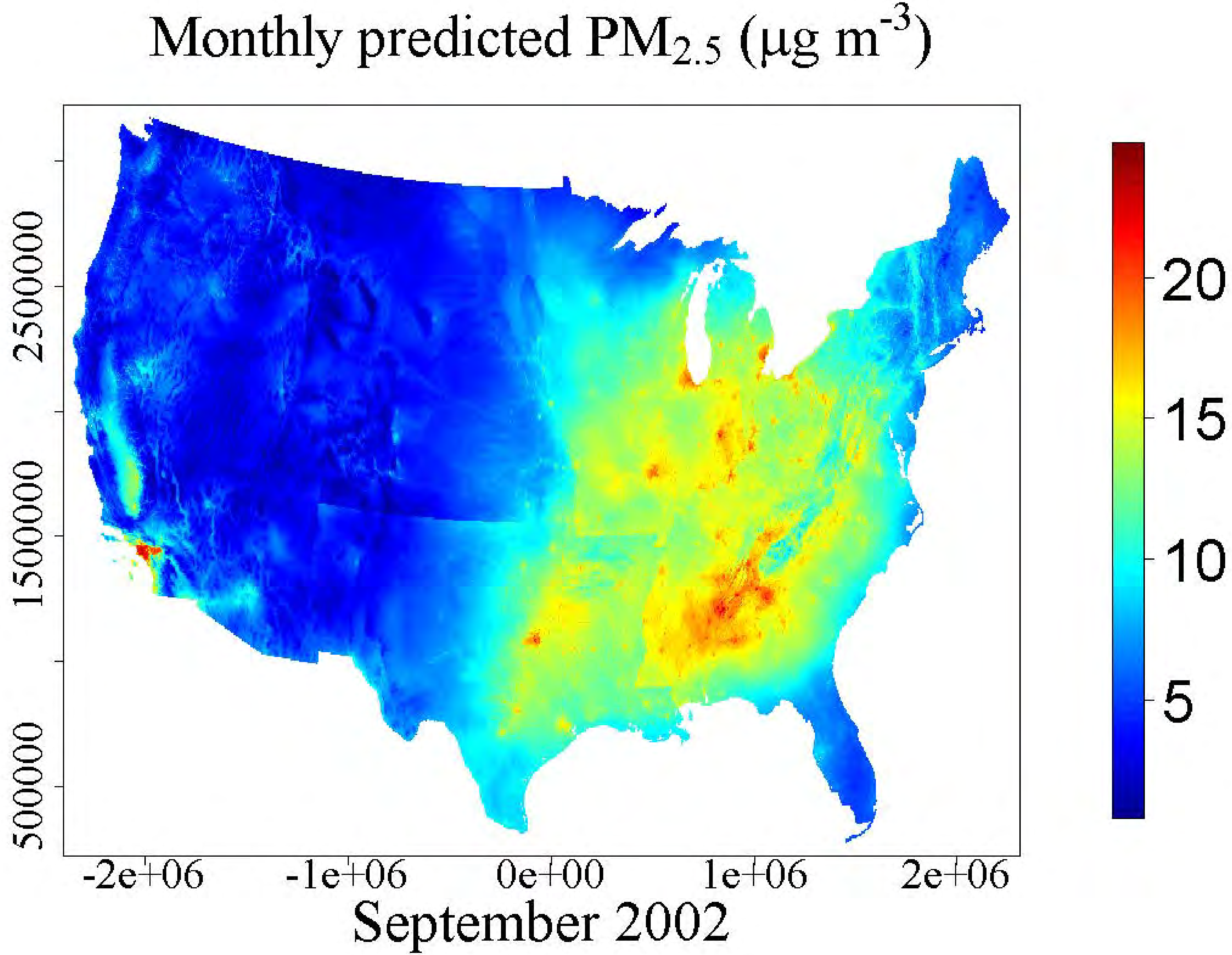

Monthly predicted PM2.5 (μg m-3)
25000000
15000000
5000000
-2e+06
-1e+06
0e+00
1e+06
2e+06
20
15
10
5
September 2002

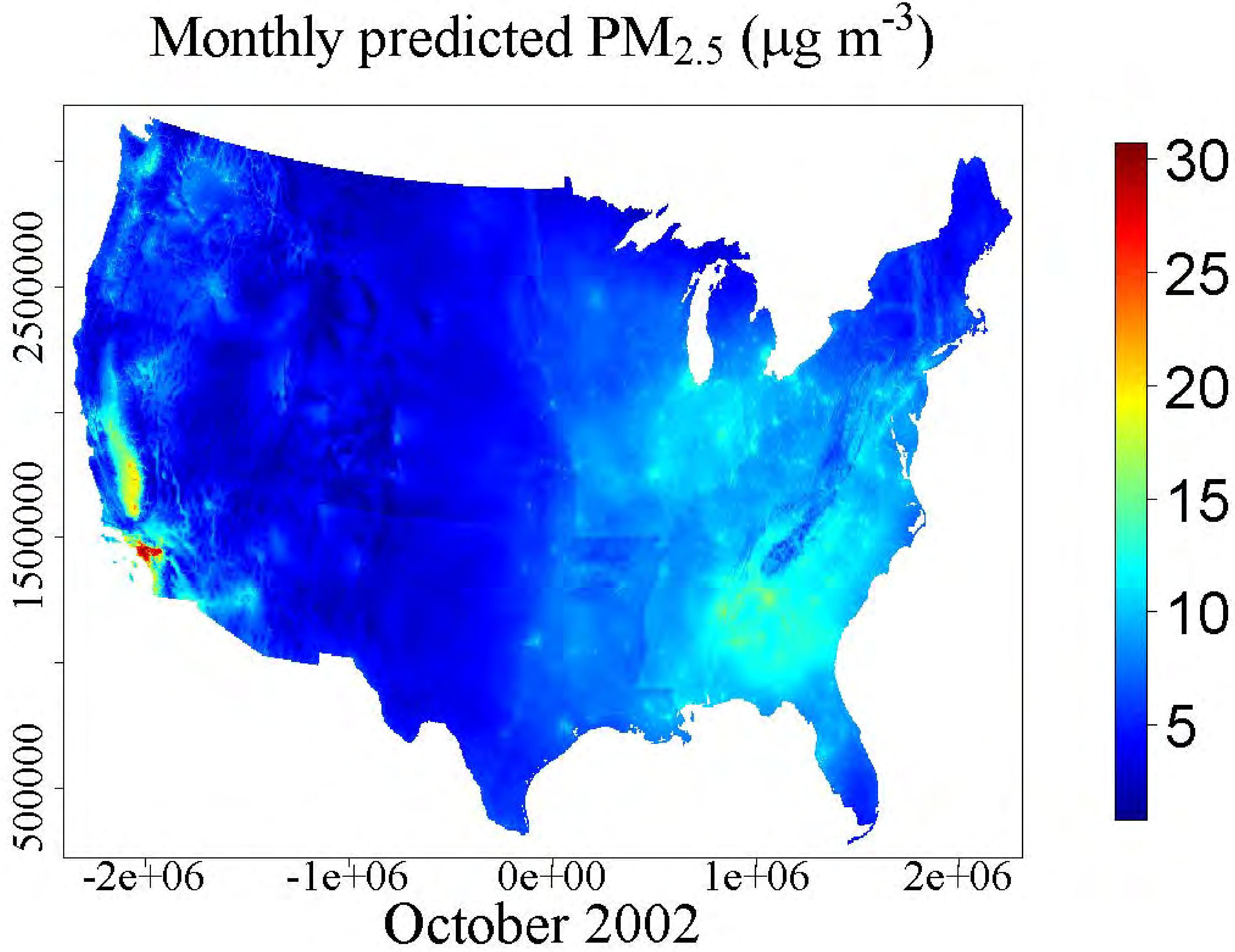

Monthly predicted PM2.5 (µg m-3)
25000000
15000000
5000000
-2e+06
-1e+06
0e+00
1e+06
2e+06
October 2002
30
25
20
15
10
5

Monthly predicted $PM_{2.5}$ ($\mu g\ m^{-3}$)

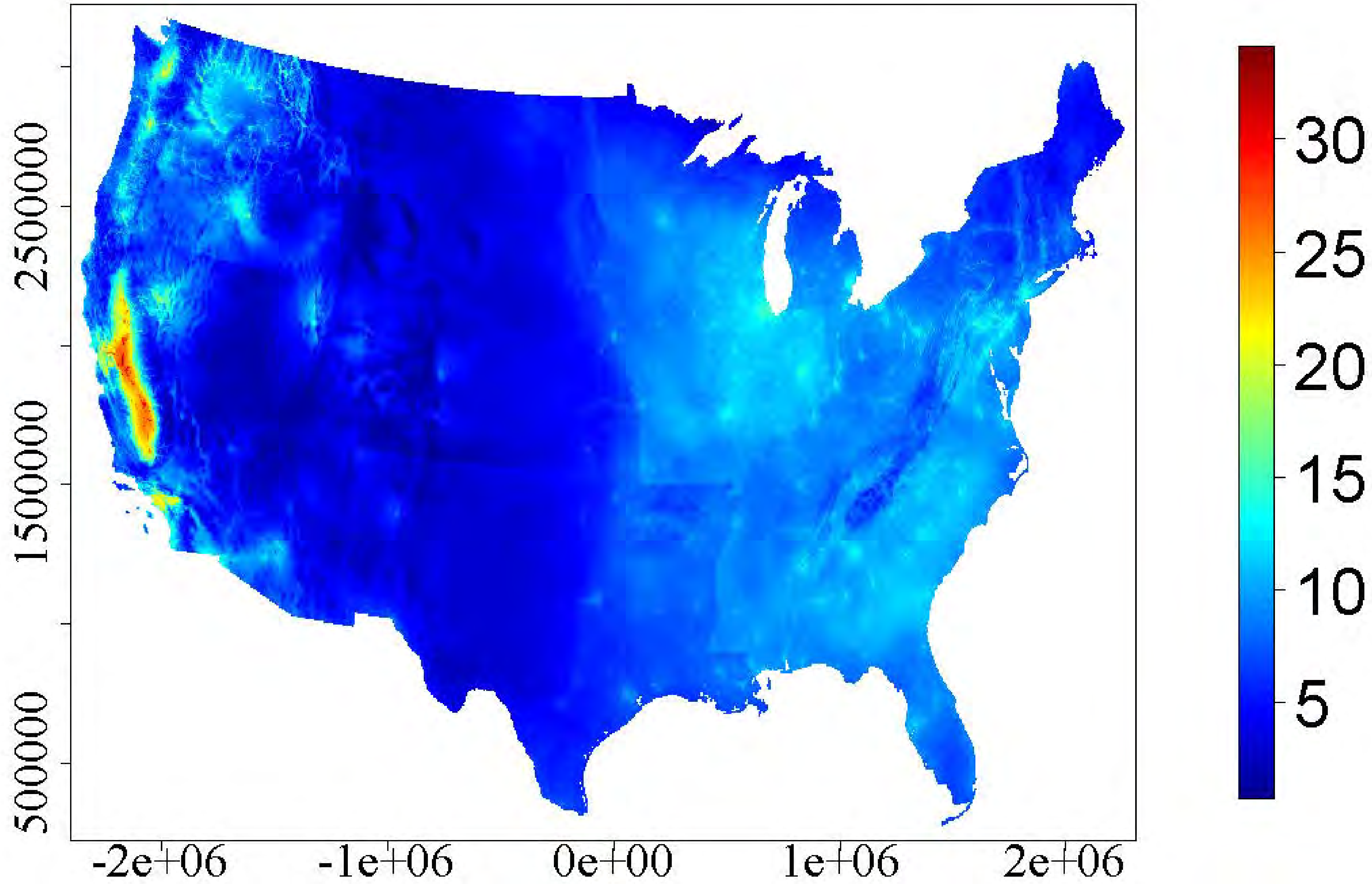

November 2002

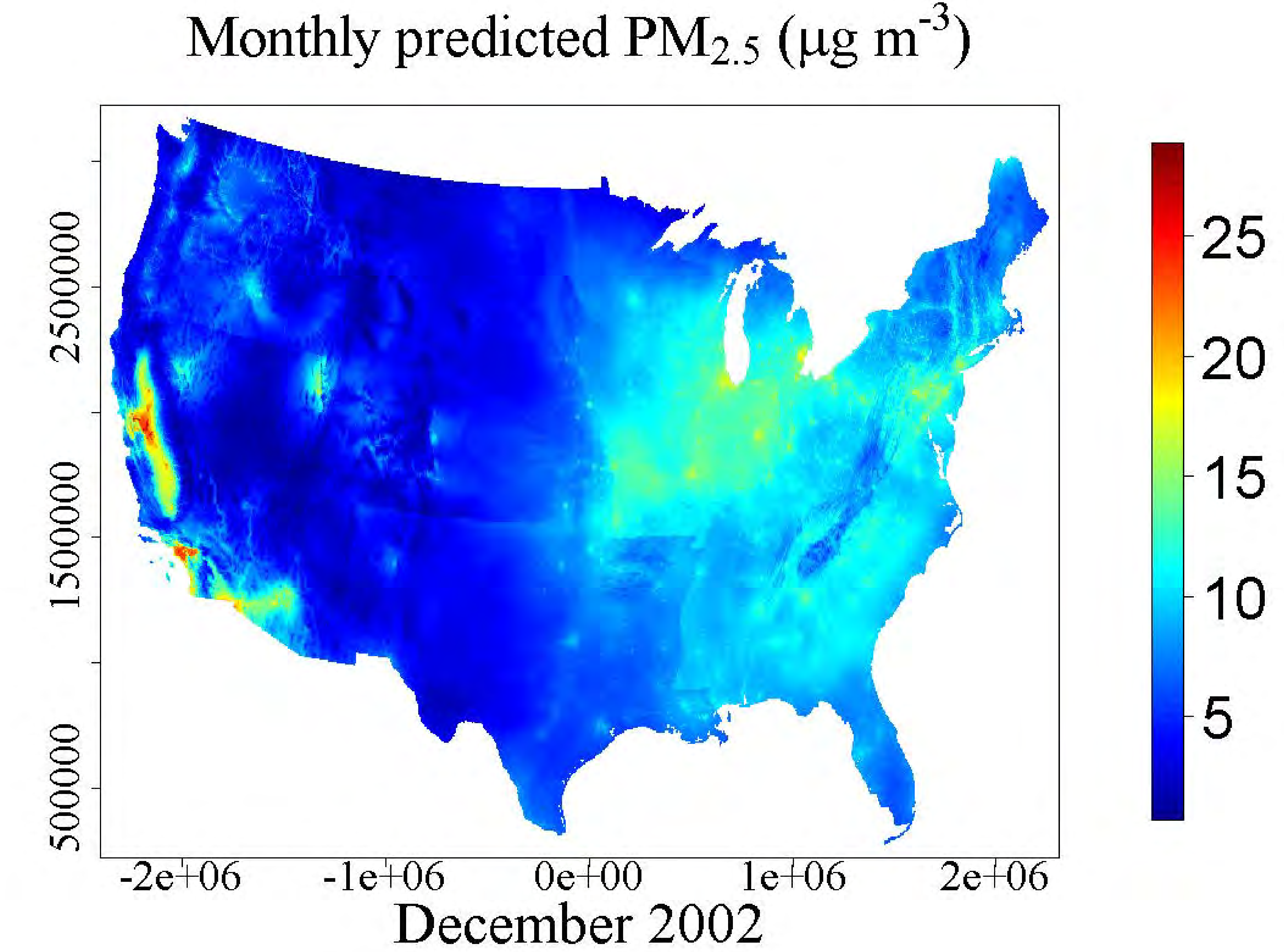

Monthly predicted PM2.5 (μg m-3)
25000000
15000000
5000000
-2e+06
-1e+06
0e+00
1e+06
2e+06
December 2002
25
20
15
10
5

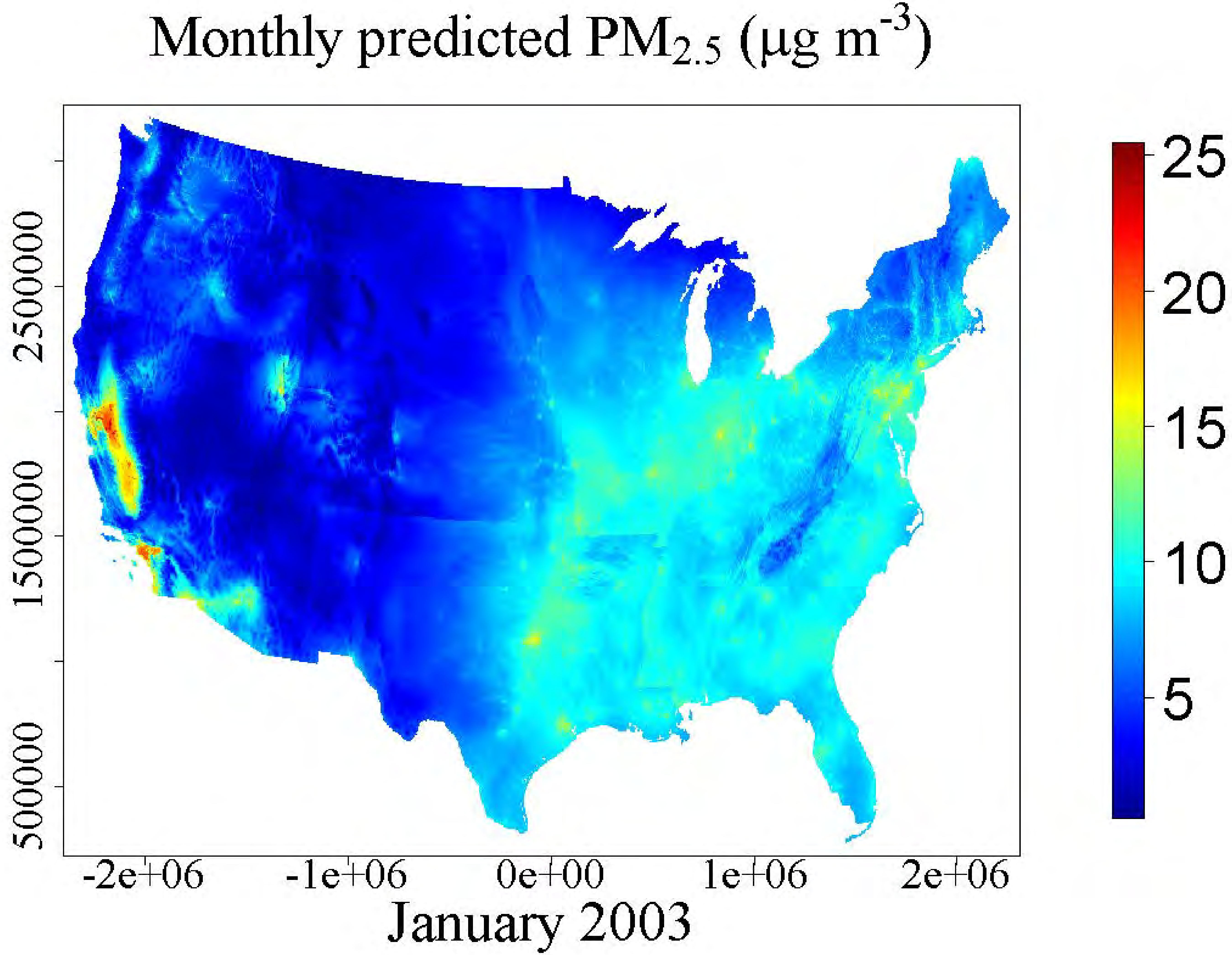
Monthly predicted PM2.5 (μg m-3)
25000000
15000000
5000000
-2e+06
-1e+06
0e+00
1e+06
2e+06
January 2003
25
20
15
10
5

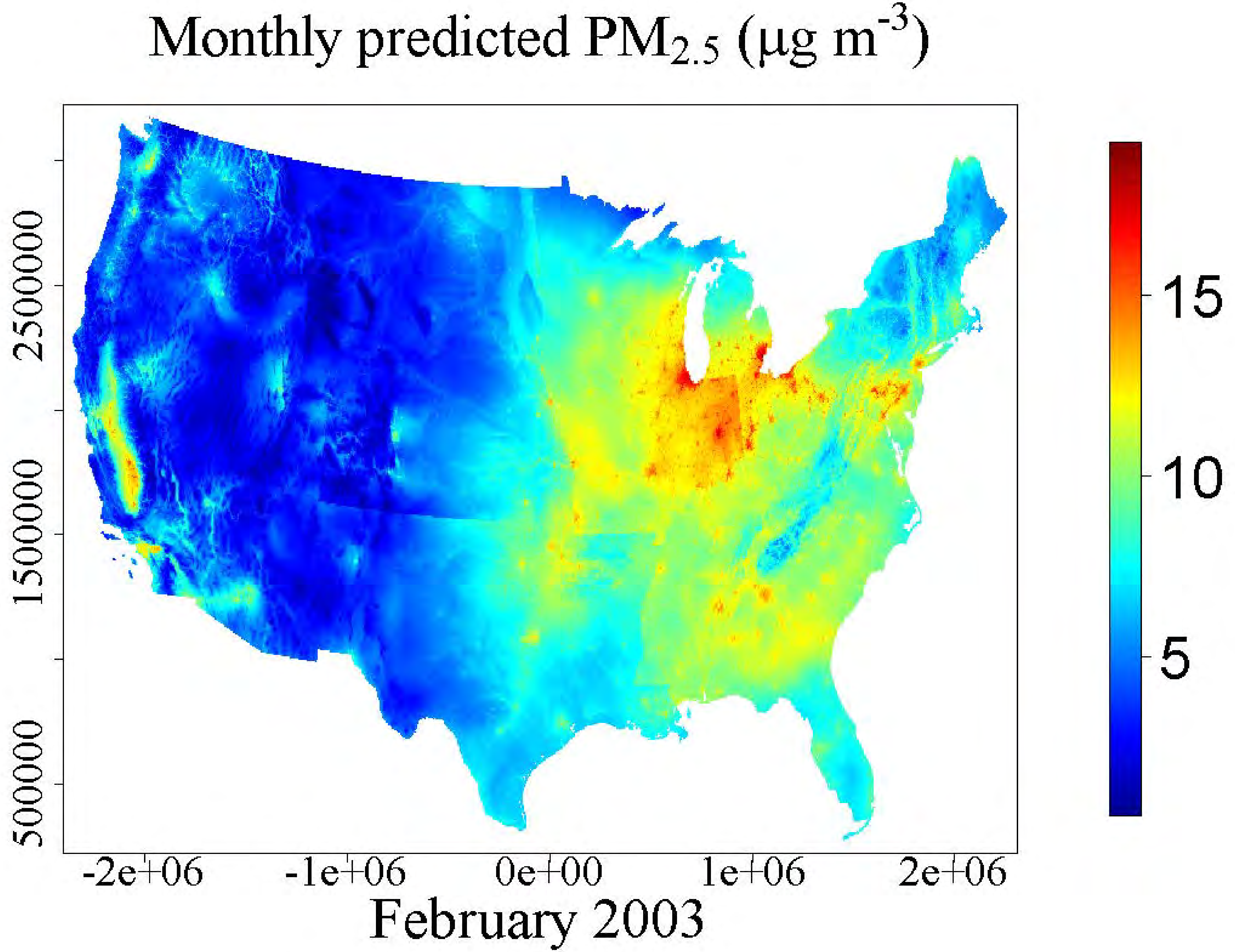
Monthly predicted PM2.5 (μg m-3)
25000000
15000000
5000000
-2e+06
-1e+06
0e+00
1e+06
2e+06
15
10
5
February 2003

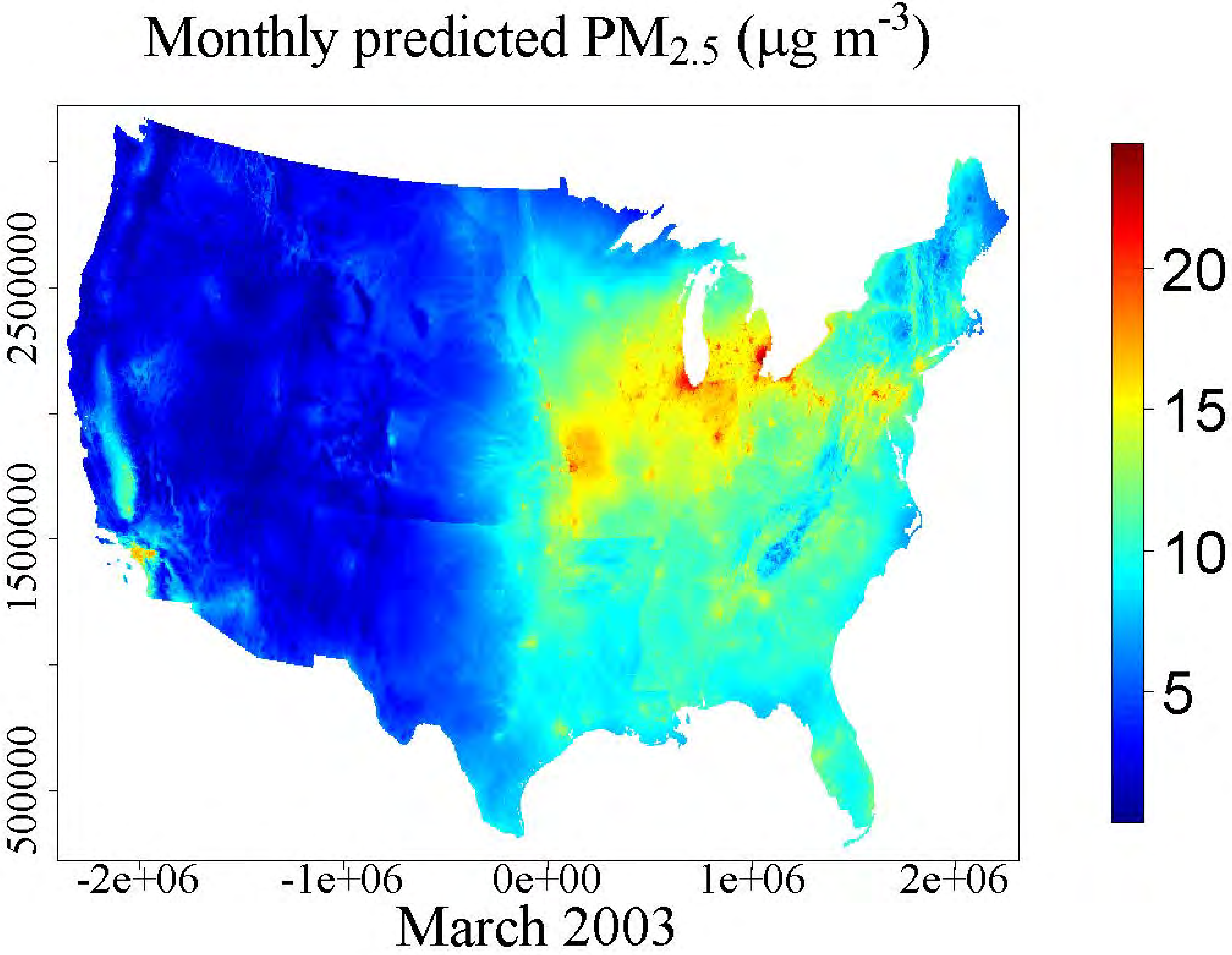

Monthly predicted PM2.5 (μg m-3)
25000000
15000000
5000000
-2e+06
-1e+06
0e+00
1e+06
2e+06
March 2003
20
15
10
5

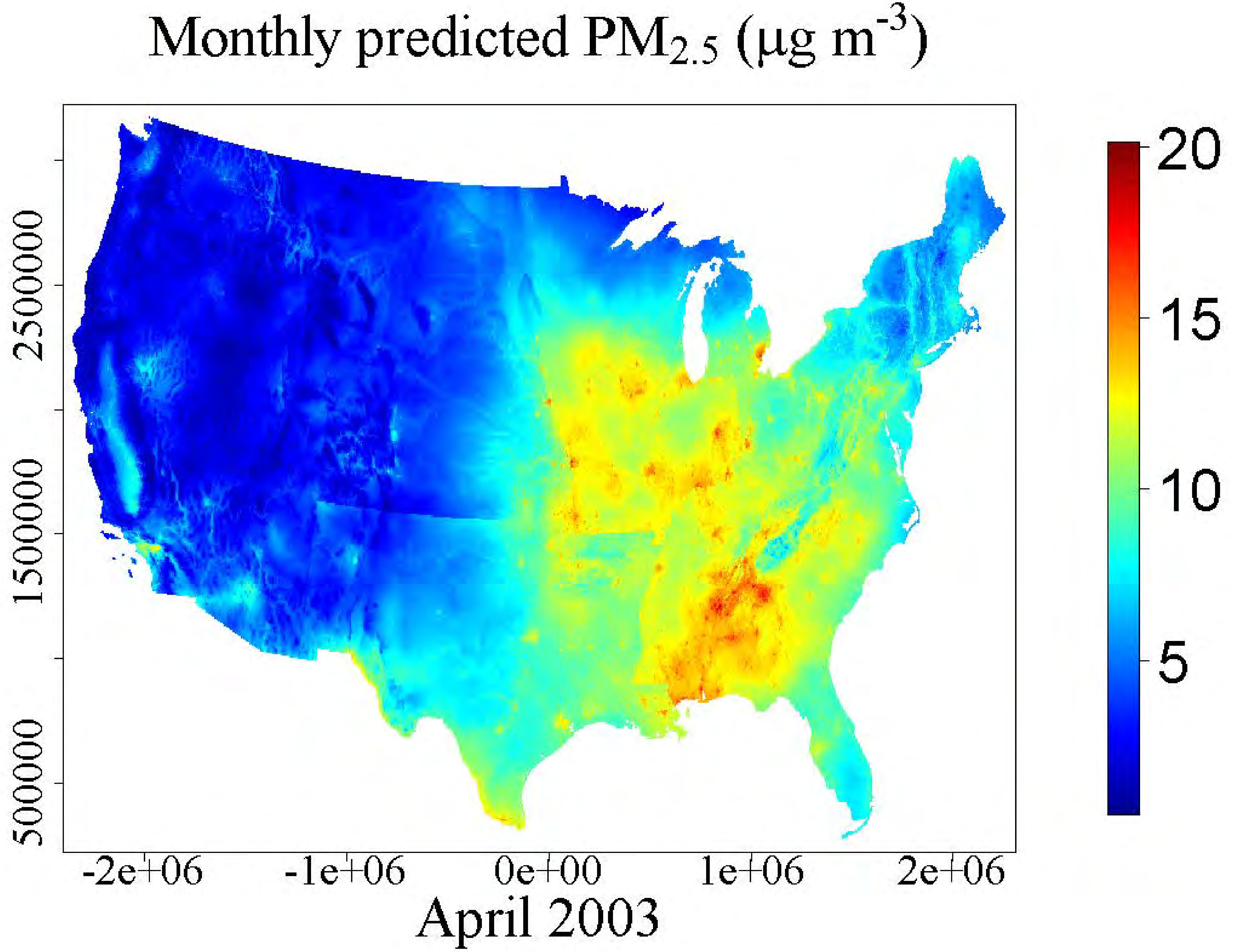

Monthly predicted PM2.5 (μg m-3)
25000000
15000000
5000000
-2e+06
-1e+06
0e+00
1e+06
2e+06
April 2003
20
15
10
5

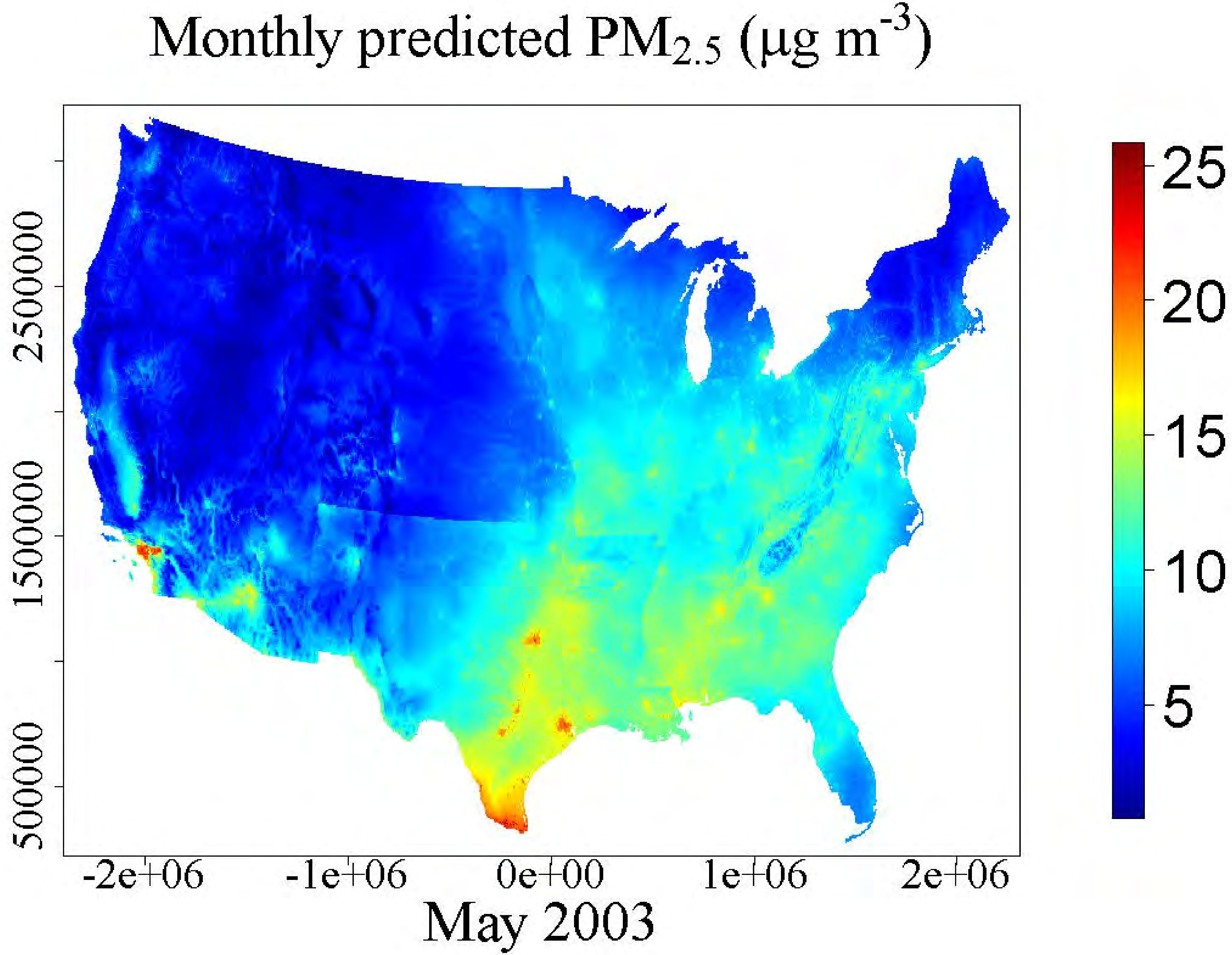
Monthly predicted PM2.5 (μg m-3)
25000000
15000000
5000000
-2e+06
-1e+06
0e+00
1e+06
2e+06
May 2003
25
20
15
10
5

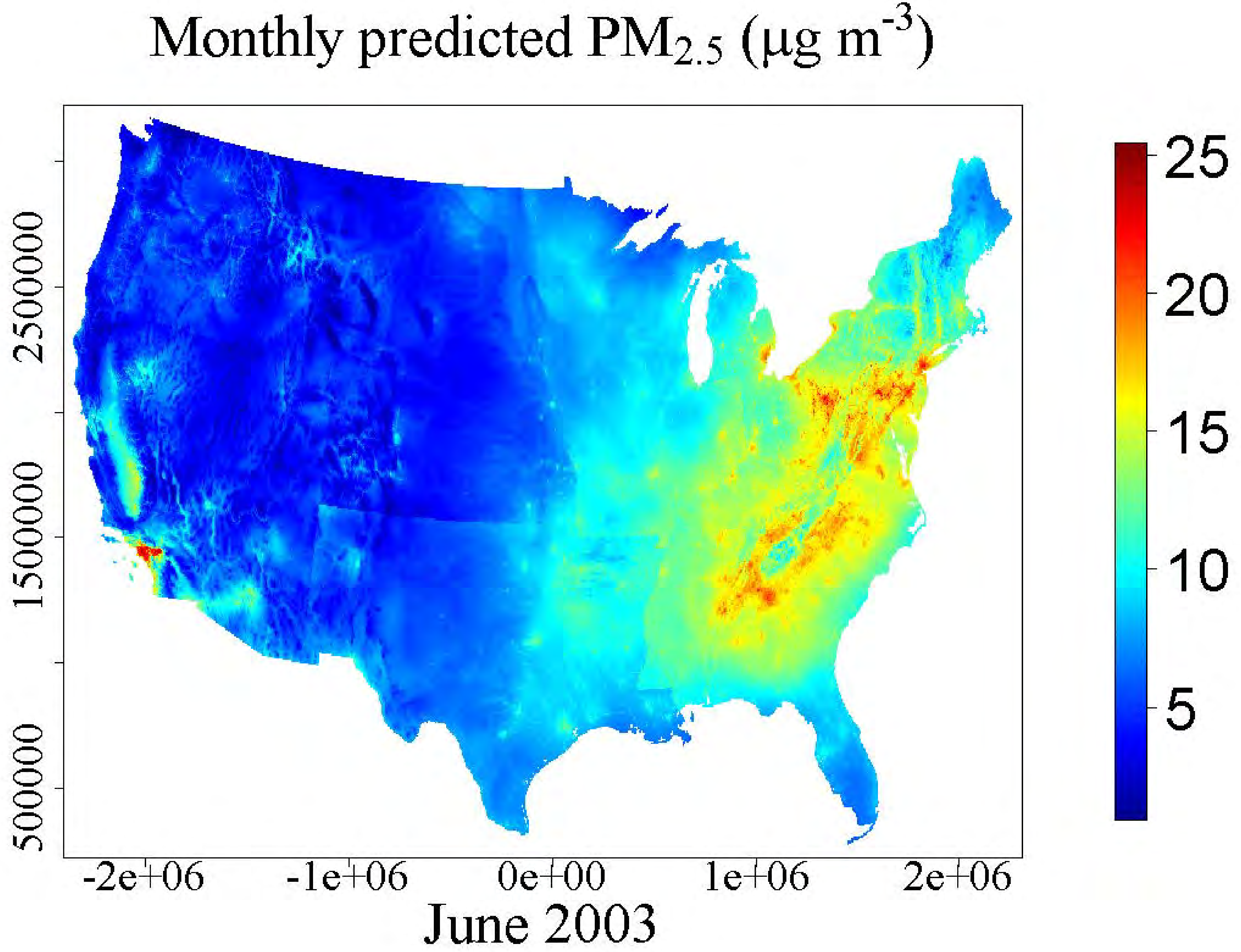
Monthly predicted PM2.5 (μg m-3)
25
20
15
10
5
2500000
1500000
5000000
-2e+06
-1e+06
0e+00
1e+06
2e+06
June 2003

Monthly predicted $PM_{2.5}$ ($\mu g\ m^{-3}$)

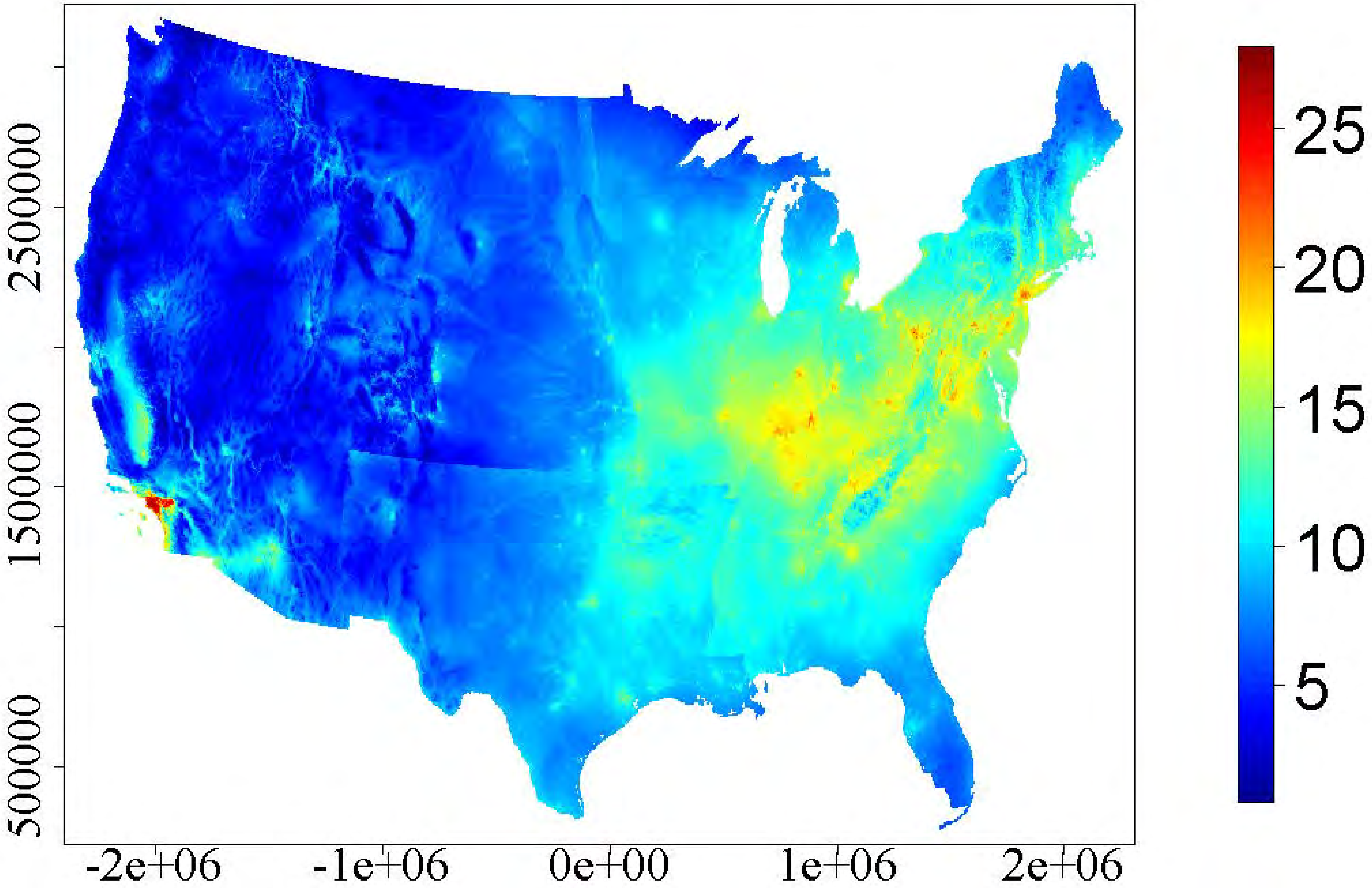

July 2003

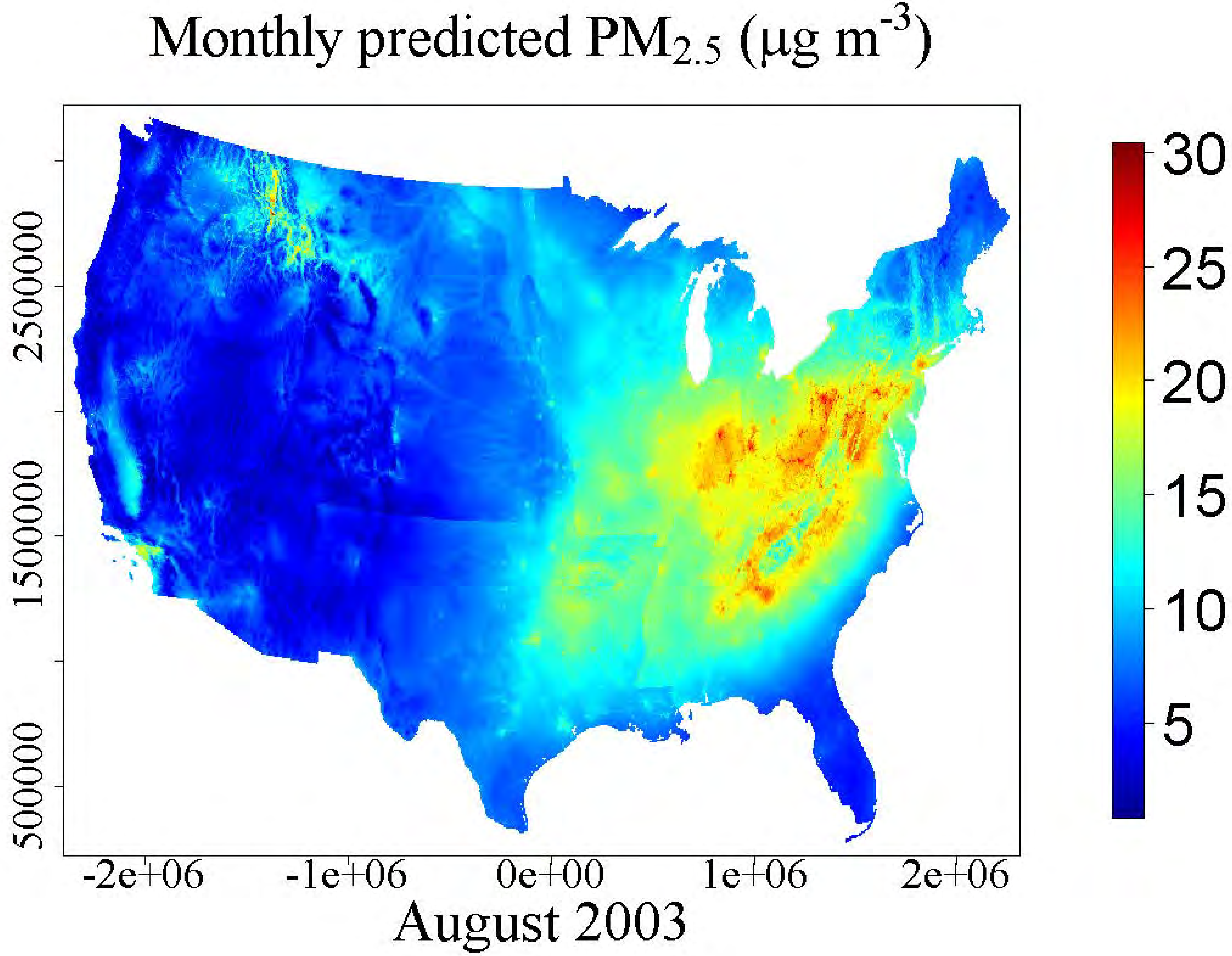

Monthly predicted PM2.5 (μg m-3)
25000000
15000000
5000000
-2e+06
-1e+06
0e+00
1e+06
2e+06
August 2003
30
25
20
15
10
5

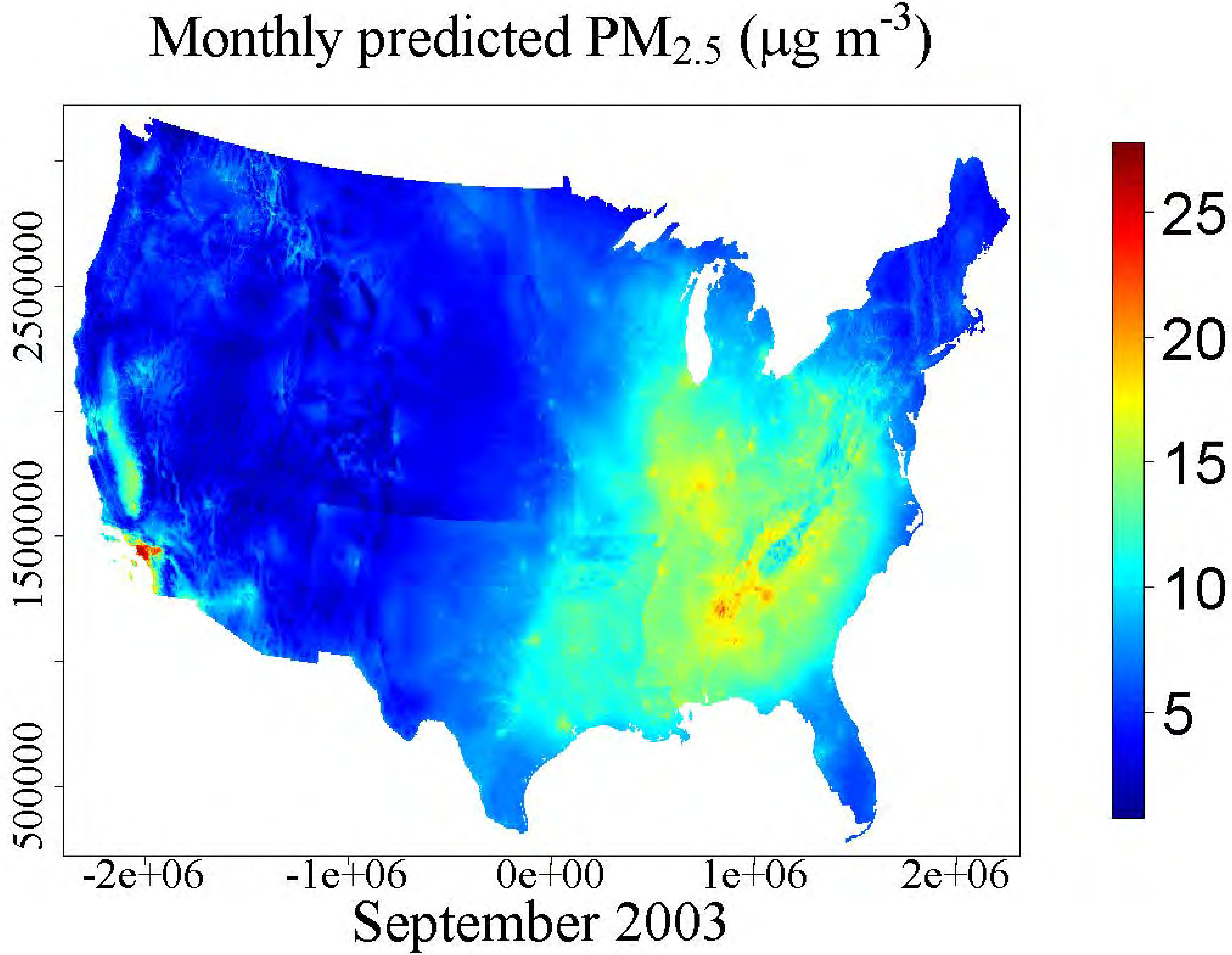

Monthly predicted PM2.5 (μg m-3)
25000000
15000000
5000000
-2e+06
-1e+06
0e+00
1e+06
2e+06
September 2003
25
20
15
10
5

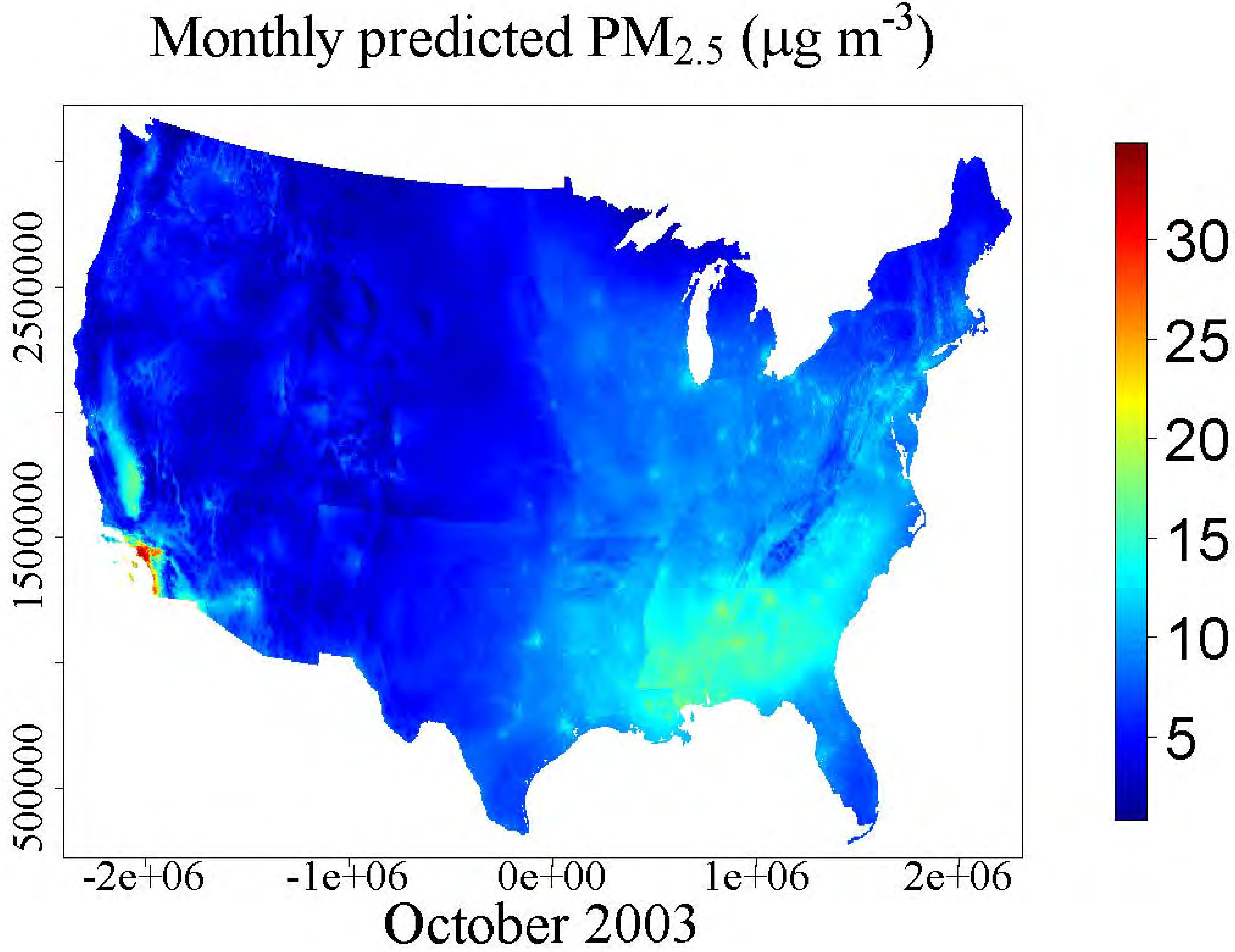
Monthly predicted PM2.5 (μg m-3)
25000000
15000000
5000000
-2e+06
-1e+06
0e+00
1e+06
2e+06
30
25
20
15
10
5
October 2003

Monthly predicted $PM_{2.5}$ ($\mu g\ m^{-3}$)

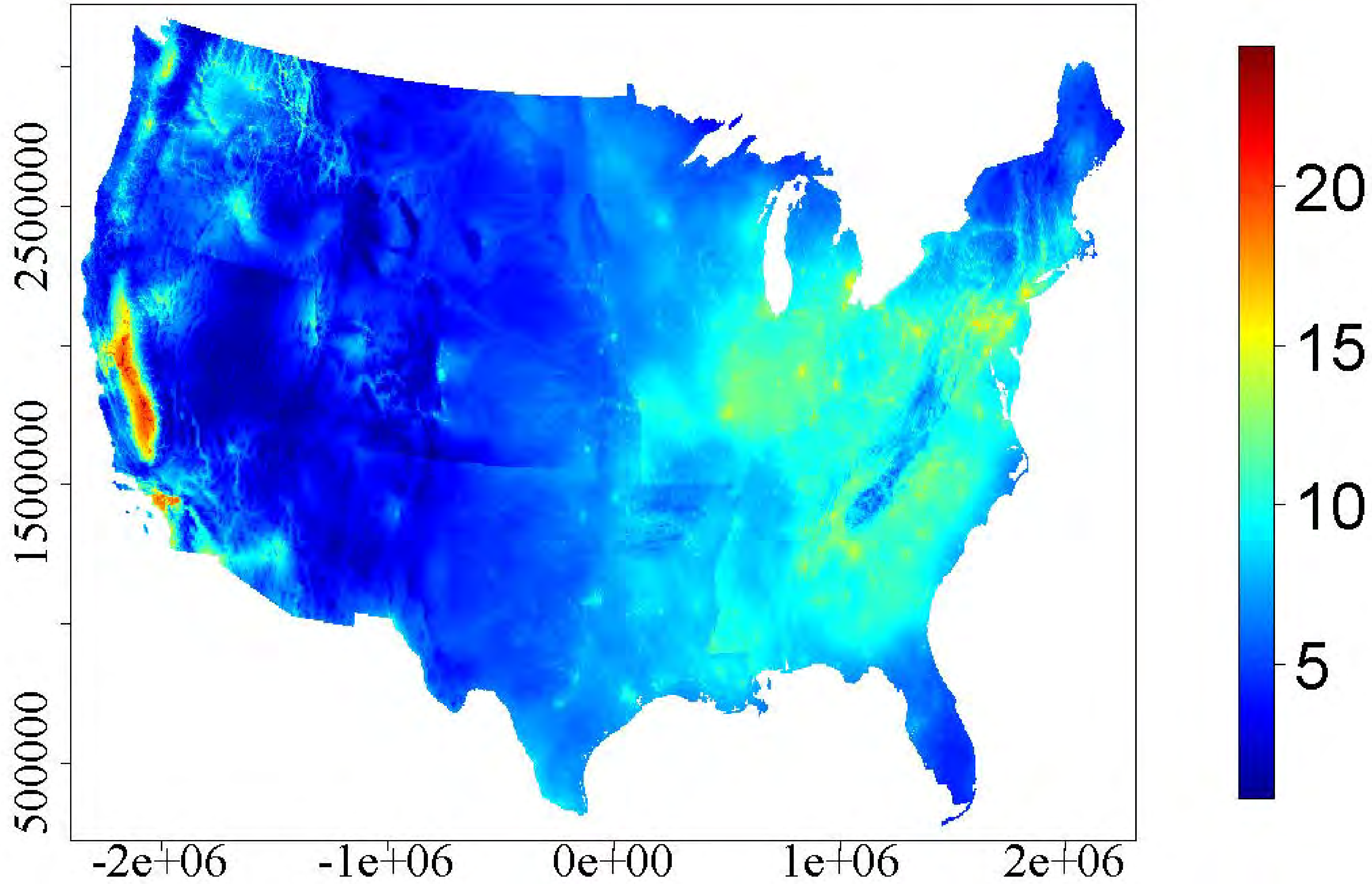

November 2003

Monthly predicted $PM_{2.5}$ ($\mu g\ m^{-3}$)

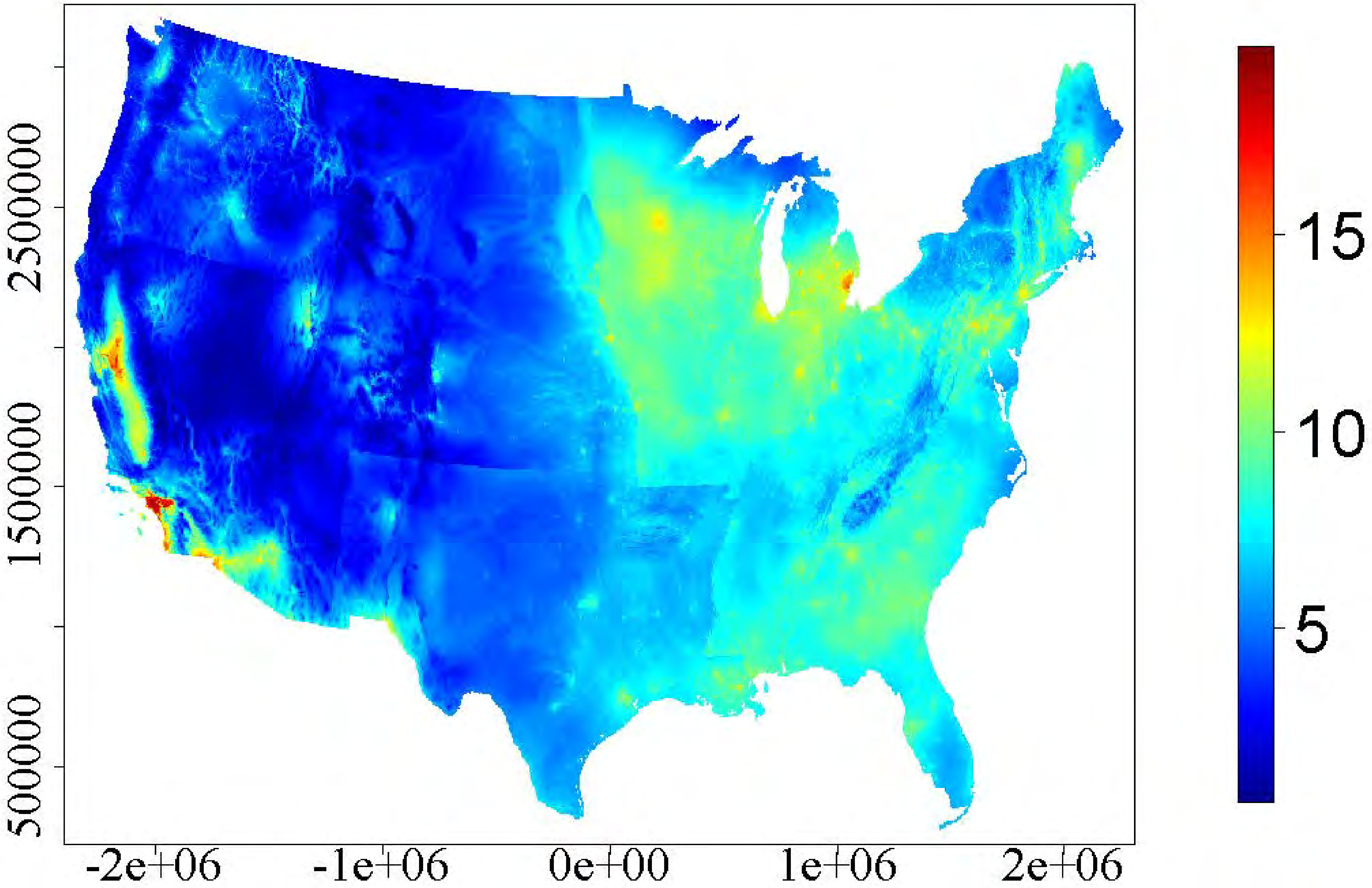

December 2003

Monthly predicted $PM_{2.5}$ ($\mu g\ m^{-3}$)

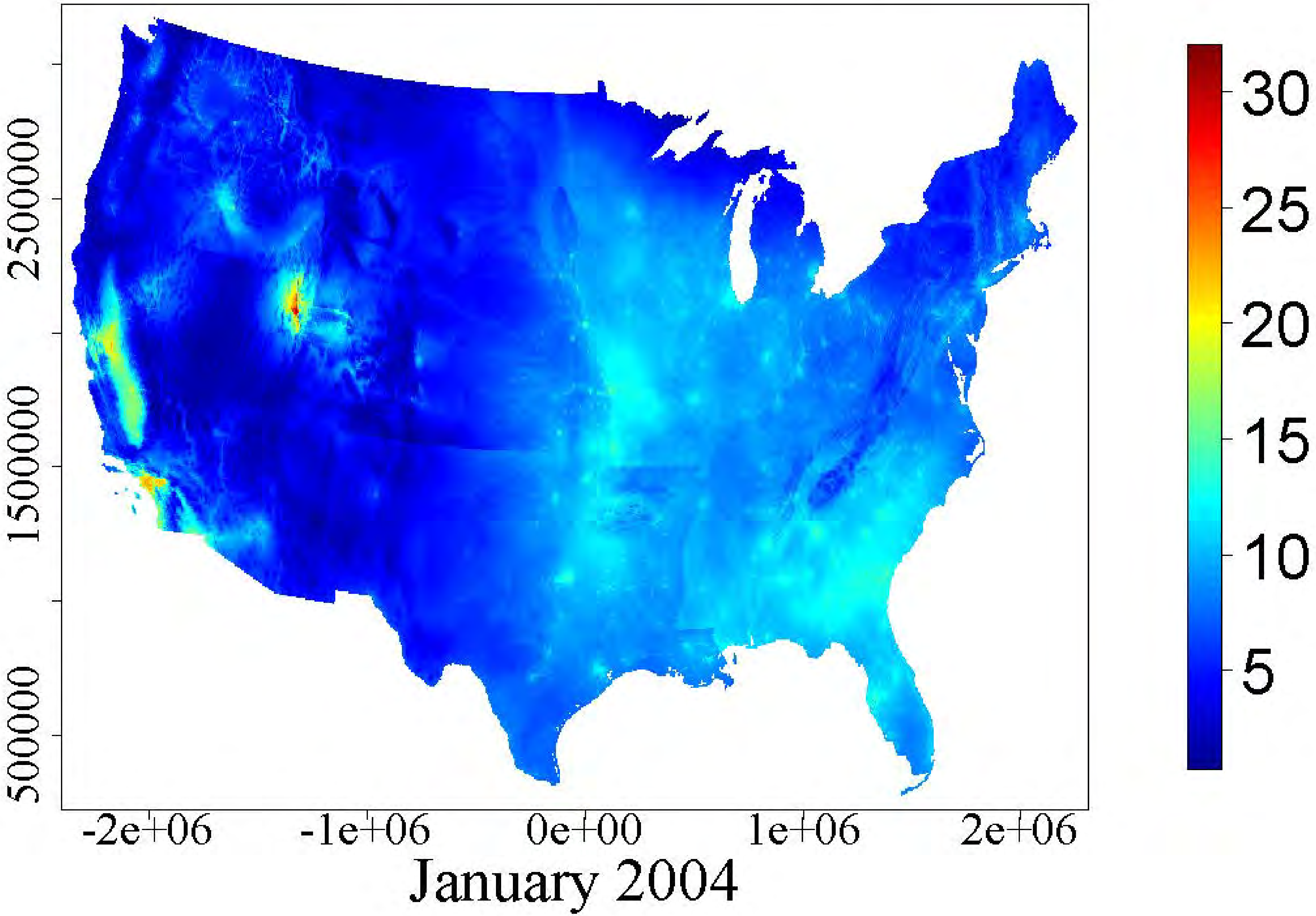

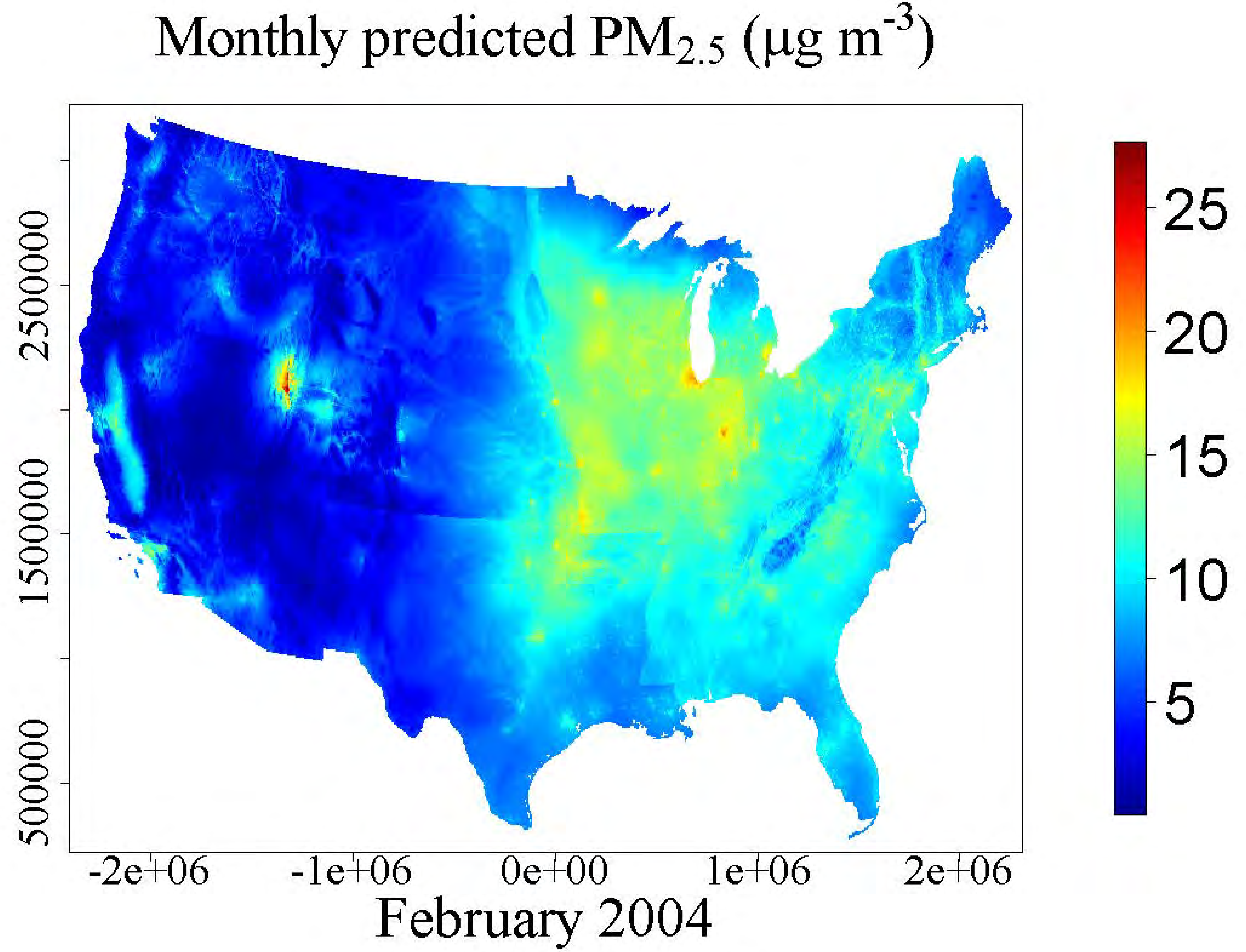
Monthly predicted PM2.5 (μg m-3)
25000000
15000000
5000000
-2e+06
-1e+06
0e+00
1e+06
2e+06
25
20
15
10
5
February 2004

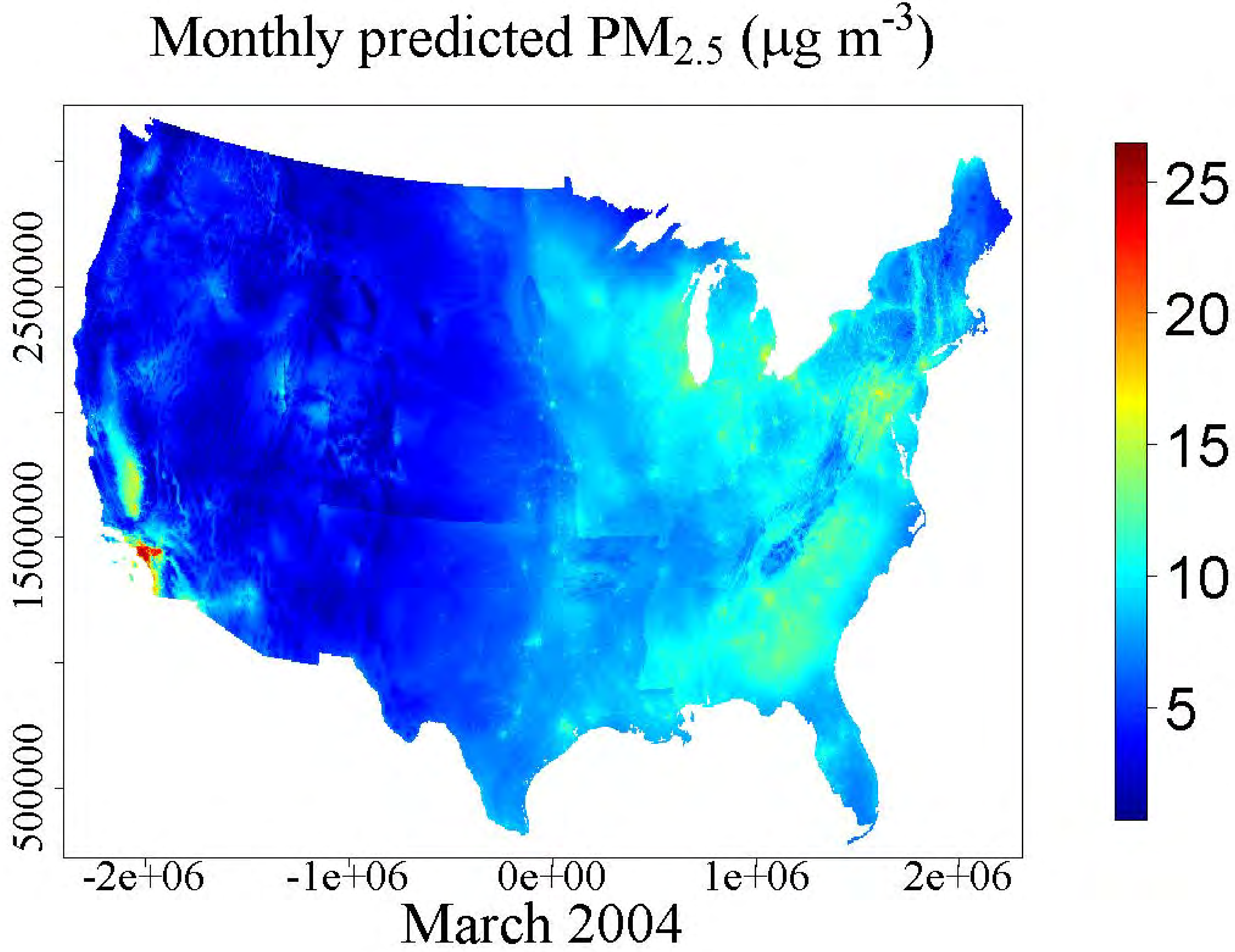
Monthly predicted PM2.5 (μg m-3)
25000000
15000000
5000000
-2e+06
-1e+06
0e+00
1e+06
2e+06
March 2004
25
20
15
10
5

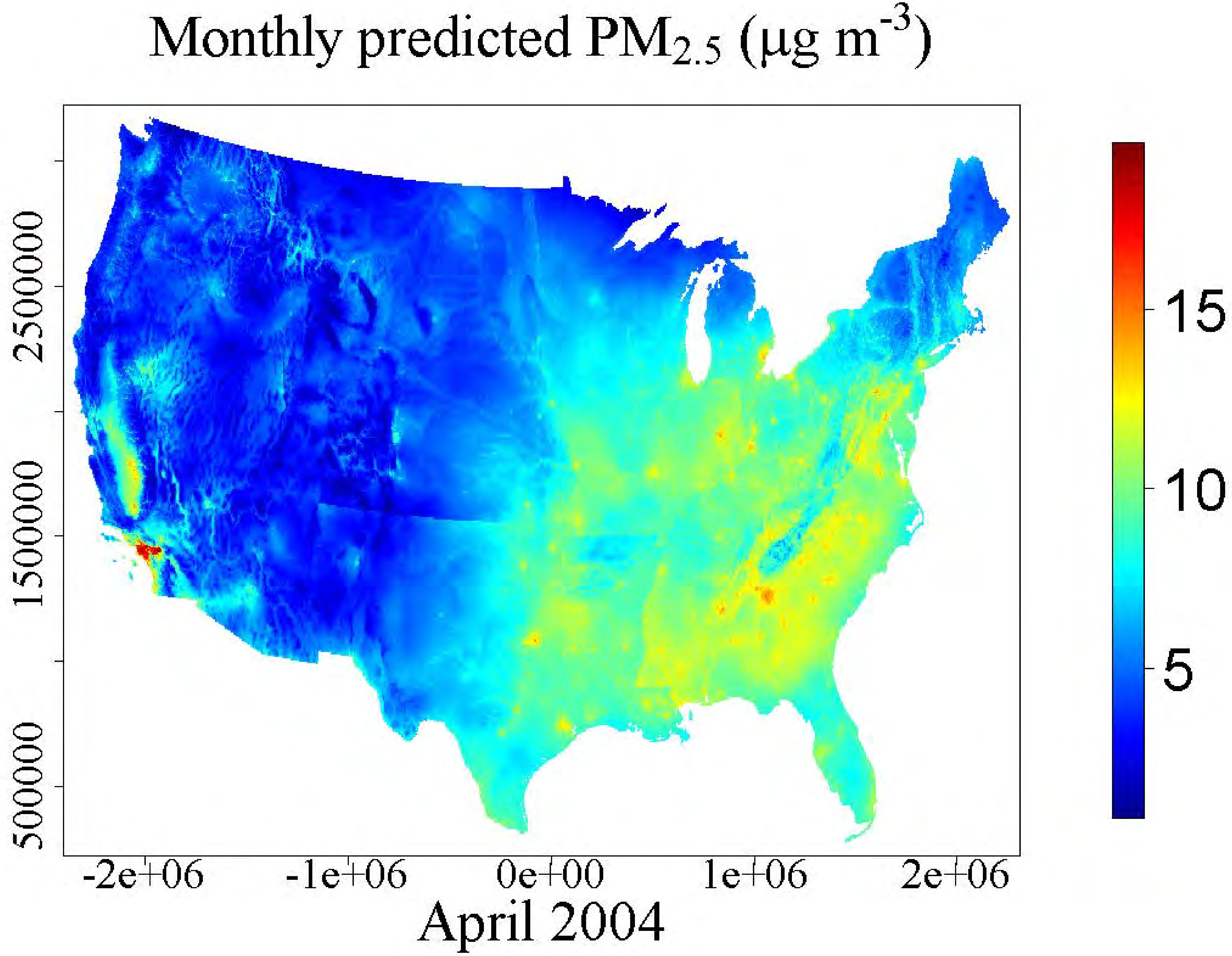
Monthly predicted PM2.5 (μg m-3)
25000000
15000000
5000000
-2e+06
-1e+06
0e+00
1e+06
2e+06
15
10
5
April 2004

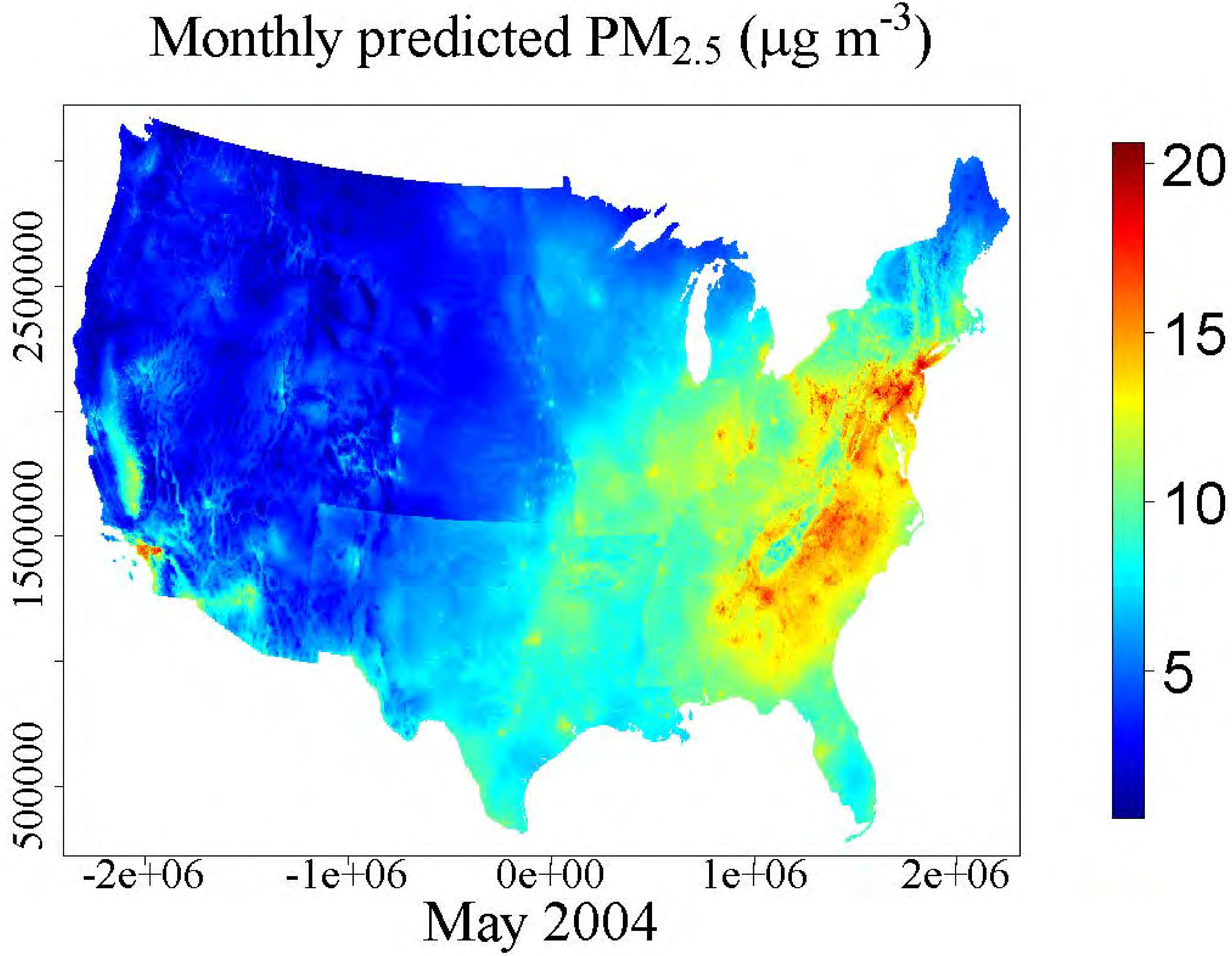
Monthly predicted PM2.5 (μg m-3)
25000000
15000000
5000000
-2e+06
-1e+06
0e+00
1e+06
2e+06
May 2004
20
15
10
5

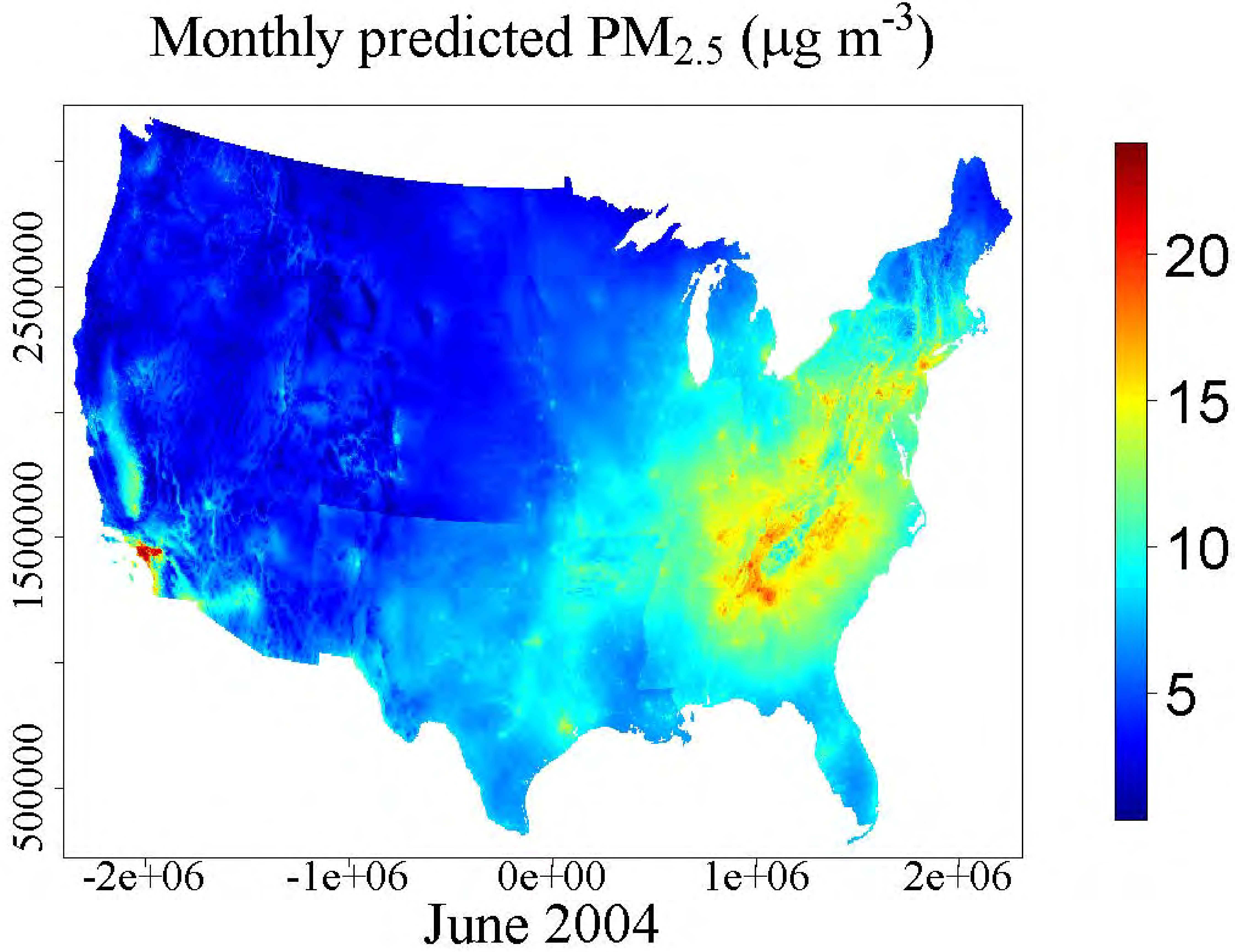

Monthly predicted PM2.5 (µg m-3)
25000000
15000000
5000000
-2e+06
-1e+06
0e+00
1e+06
2e+06
June 2004
20
15
10
5

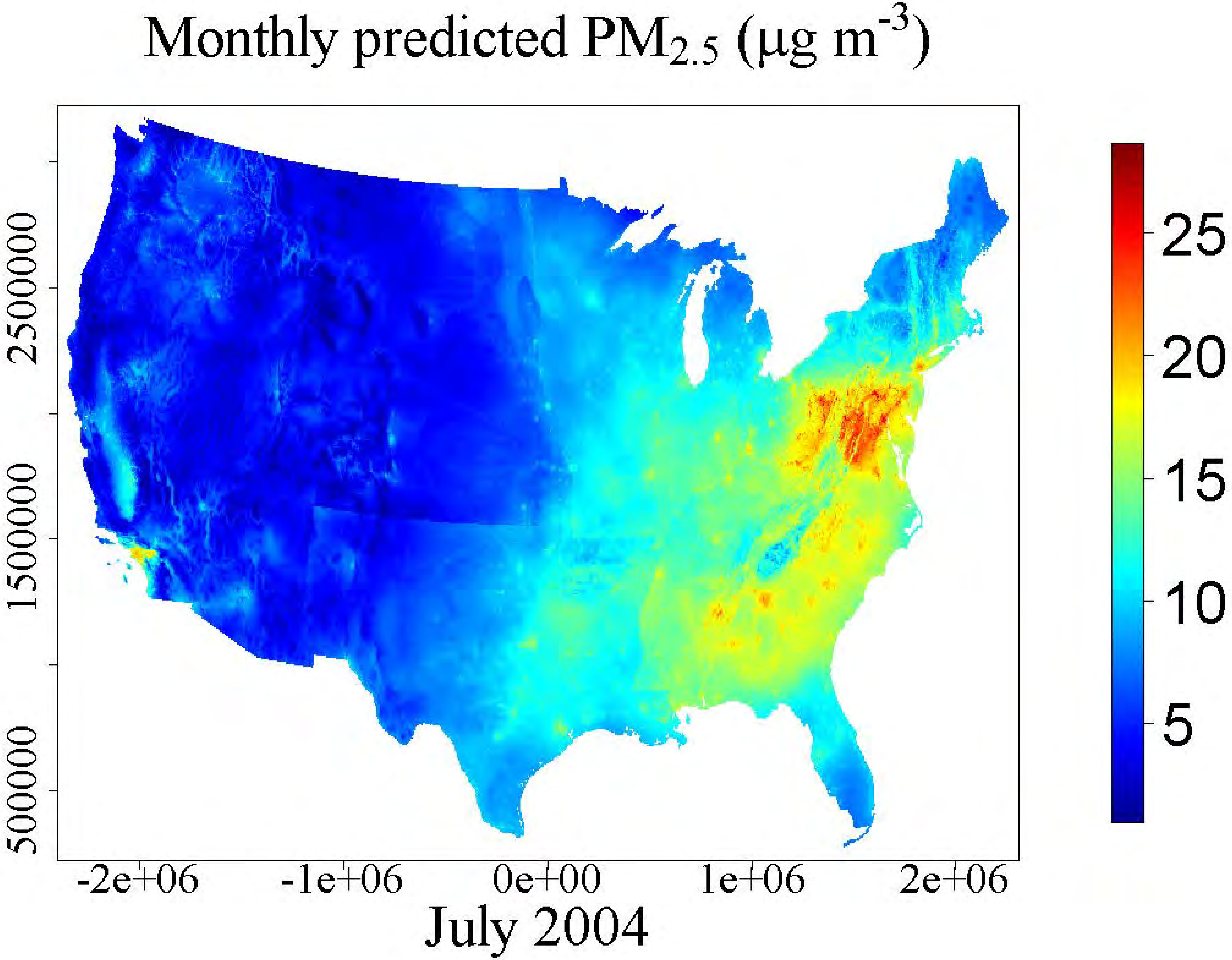
Monthly predicted PM2.5 (μg m-3)
25000000
15000000
5000000
-2e+06
-1e+06
0e+00
1e+06
2e+06
25
20
15
10
5
July 2004

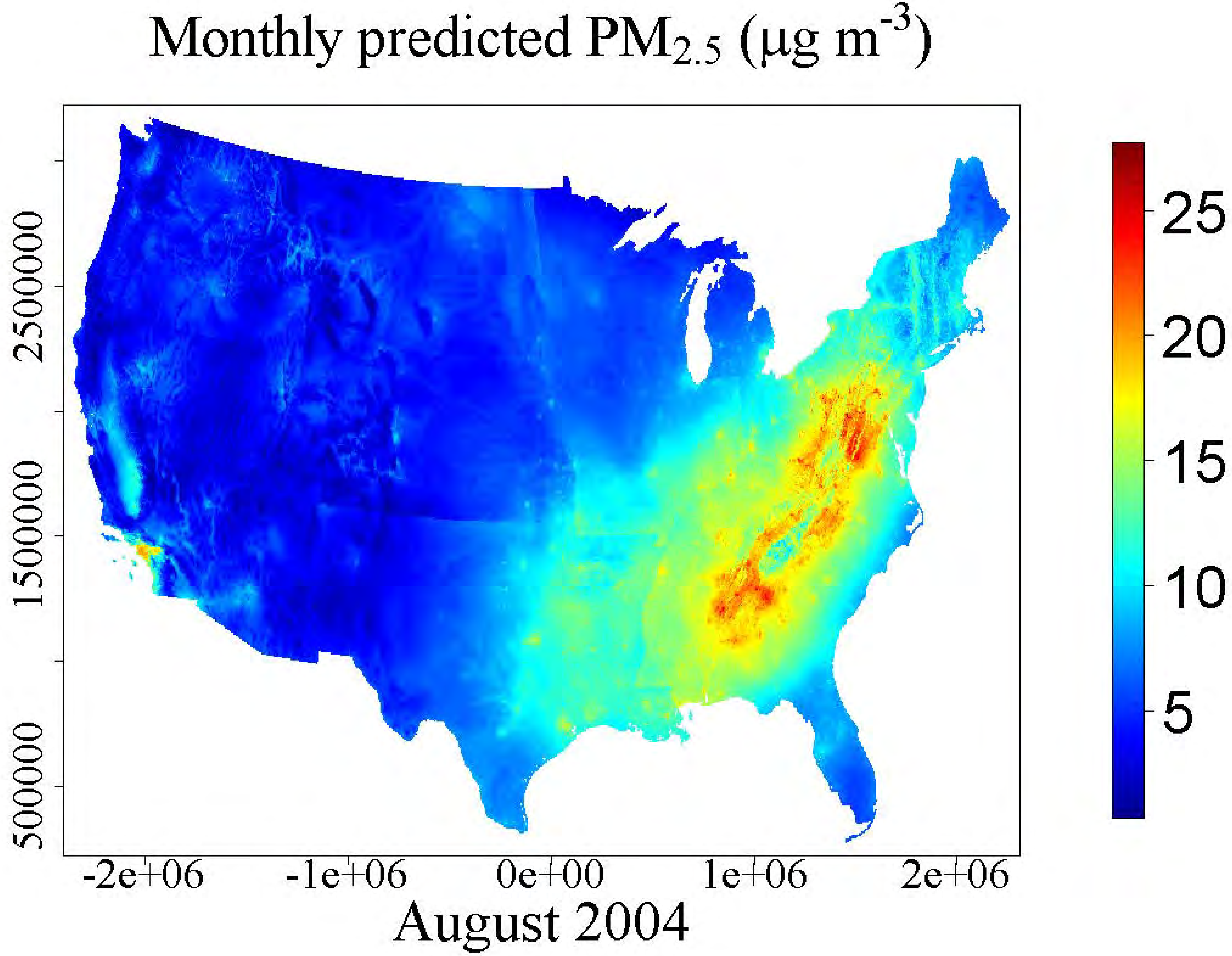
Monthly predicted PM2.5 (μg m-3)
25000000
15000000
5000000
-2e+06
-1e+06
0e+00
1e+06
2e+06
25
20
15
10
5
August 2004

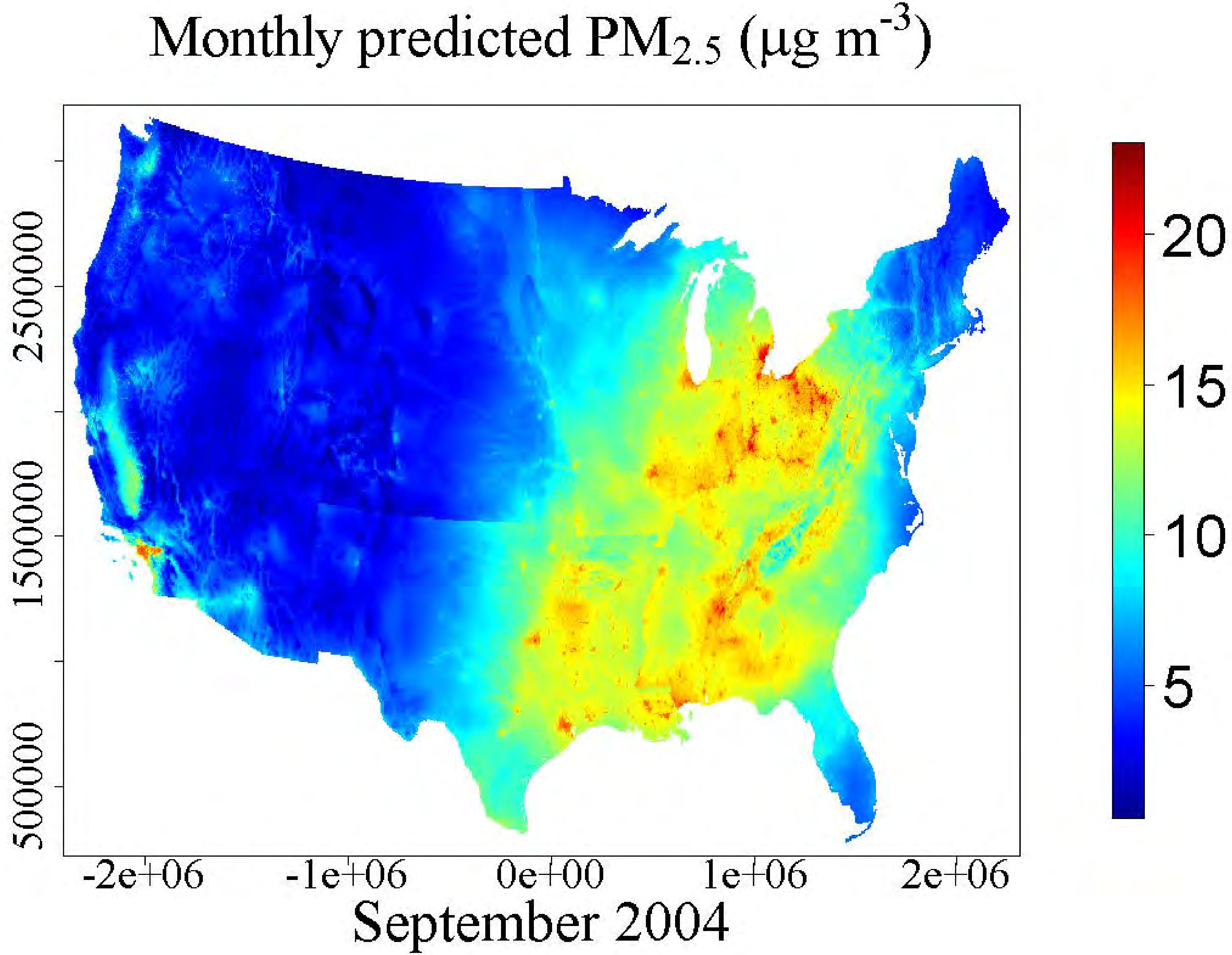
Monthly predicted PM2.5 (μg m-3)
25000000
15000000
5000000
-2e+06
-1e+06
0e+00
1e+06
2e+06
September 2004
20
15
10
5

Monthly predicted $PM_{2.5}$ ($\mu g\ m^{-3}$)

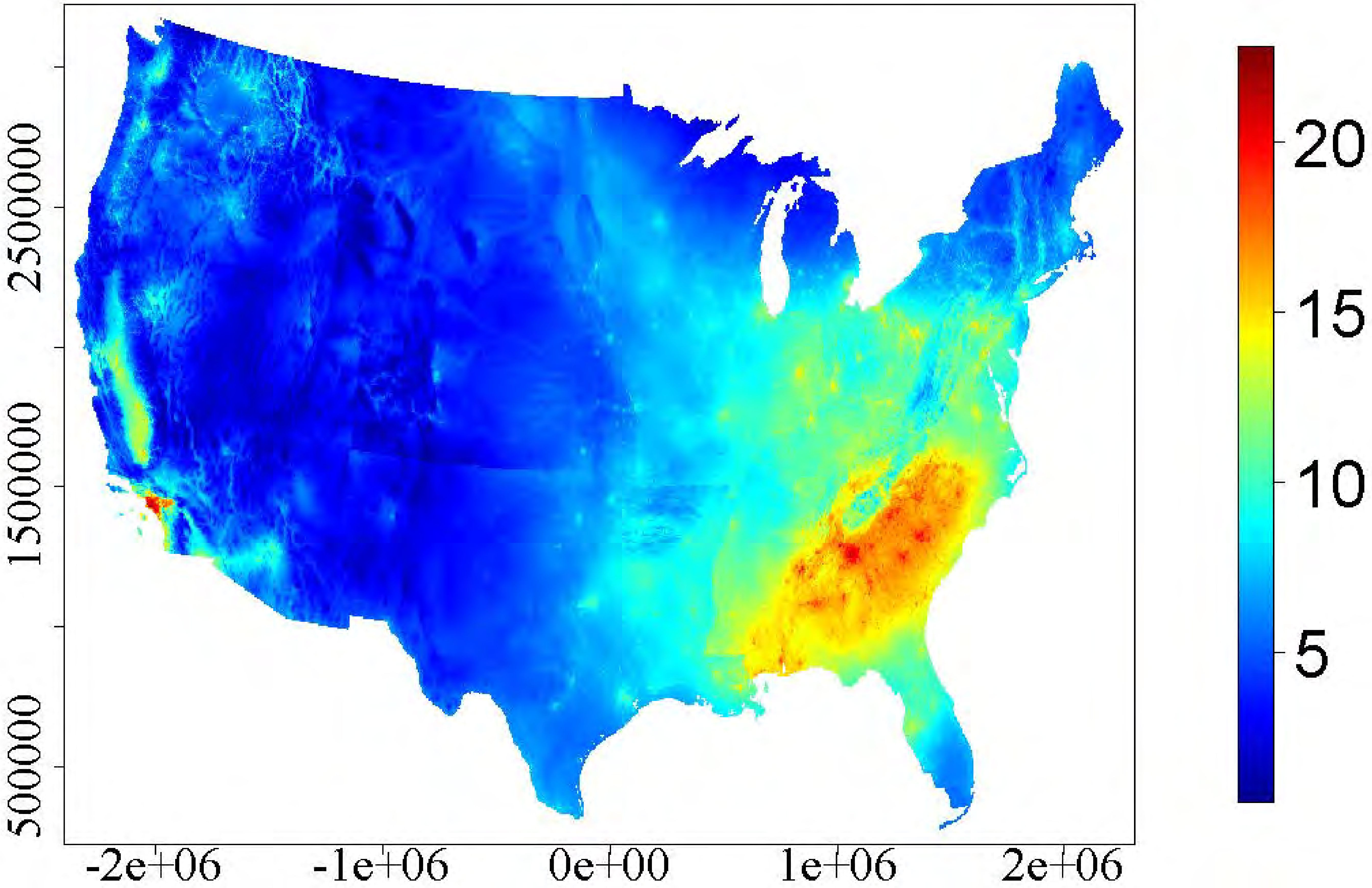

October 2004

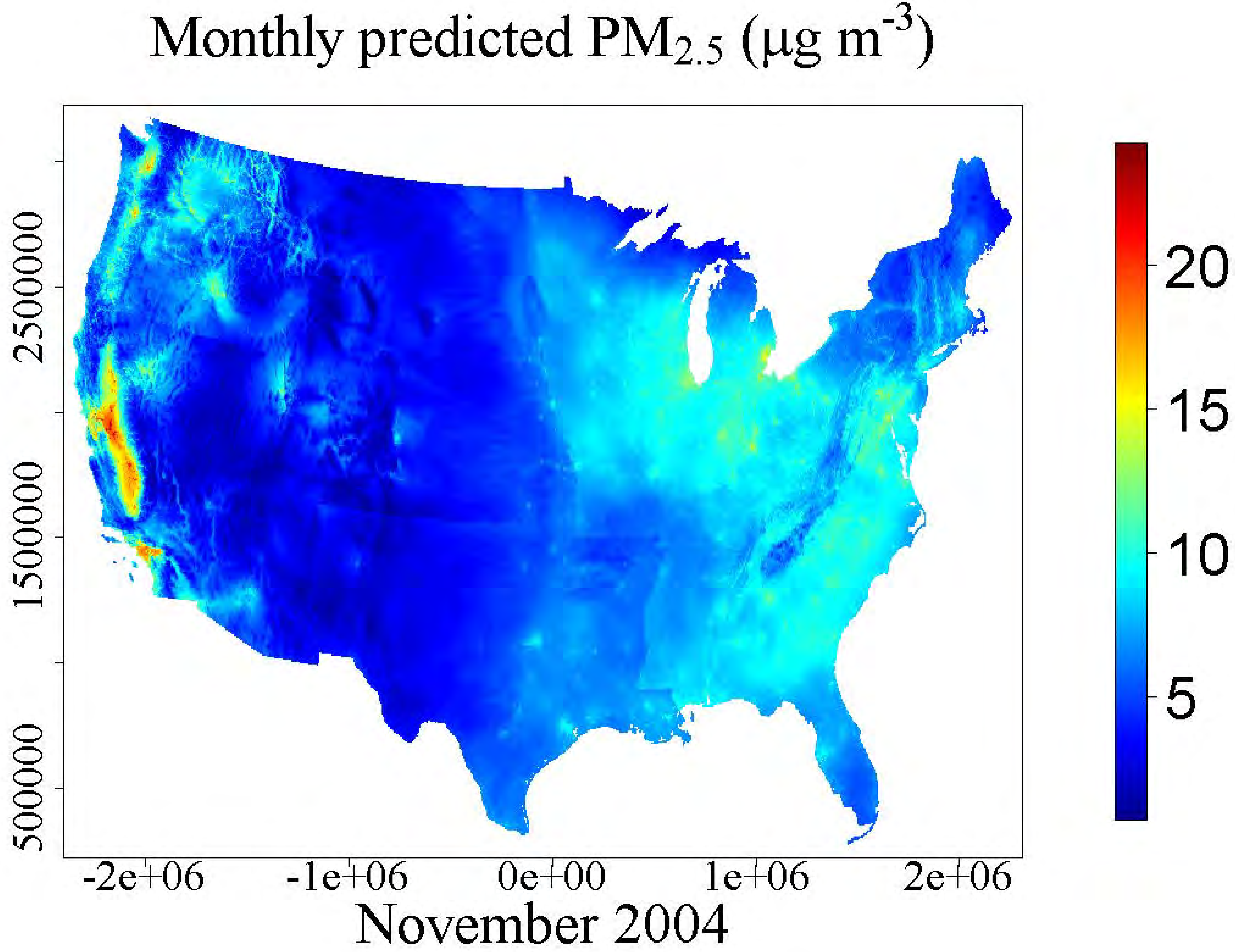
Monthly predicted PM2.5 (μg m-3)
25000000
15000000
5000000
-2e+06
-1e+06
0e+00
1e+06
2e+06
November 2004
20
15
10
5

Monthly predicted $PM_{2.5}$ ($\mu g\ m^{-3}$)

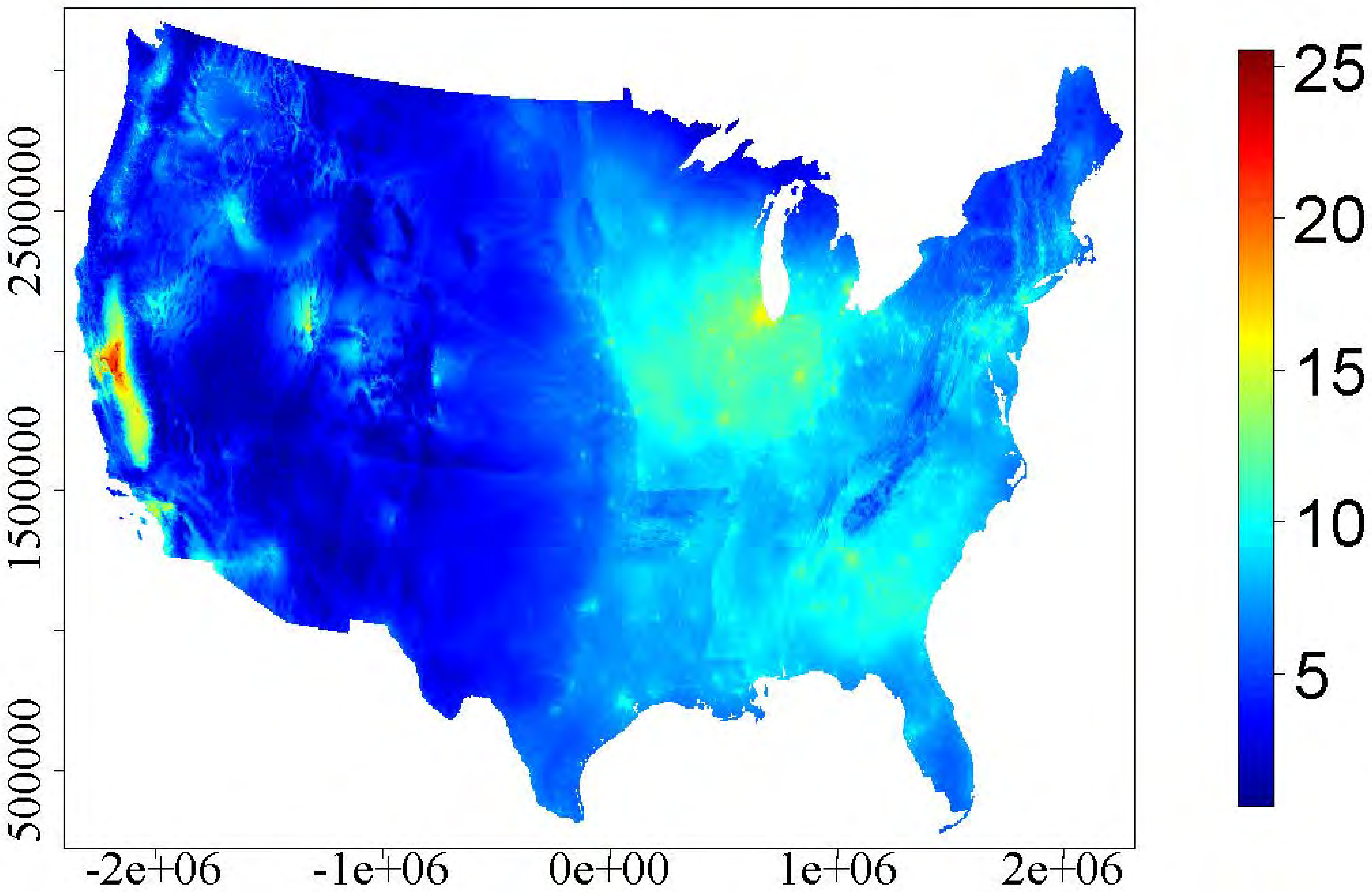

December 2004

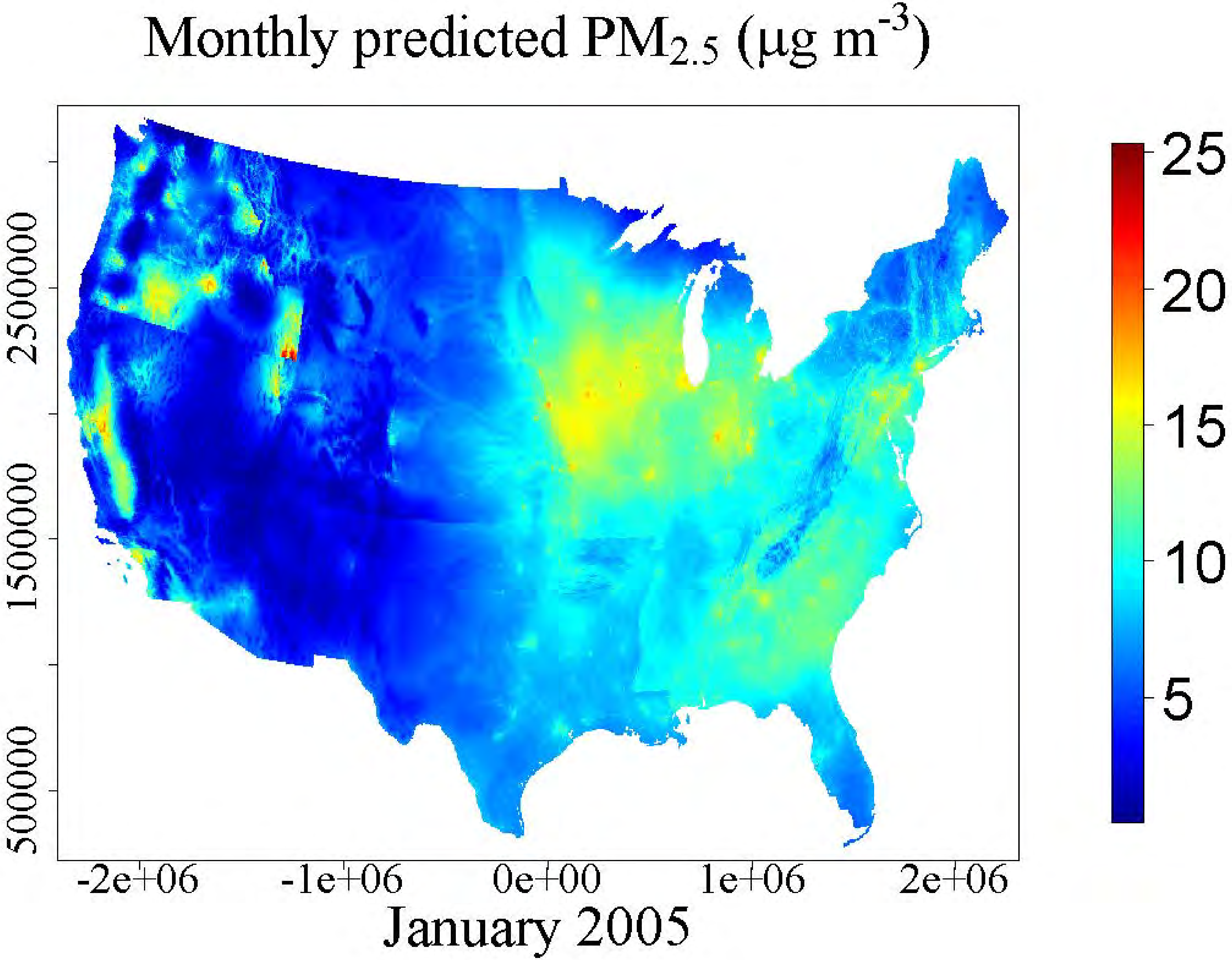
Monthly predicted PM2.5 (μg m-3)
25000000
15000000
5000000
-2e+06
-1e+06
0e+00
1e+06
2e+06
January 2005
25
20
15
10
5

Monthly predicted $PM_{2.5}$ ($\mu g\ m^{-3}$)

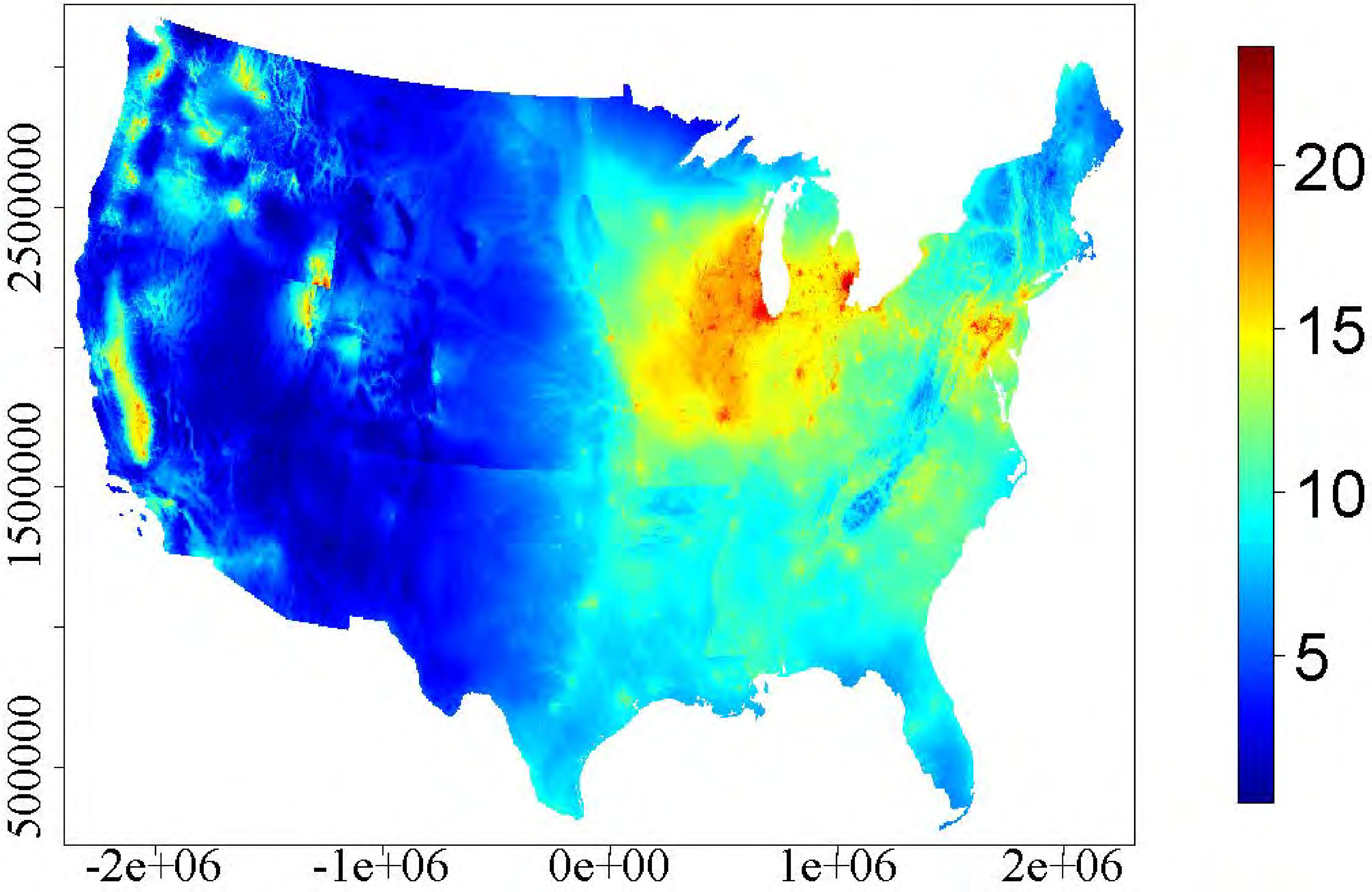

February 2005

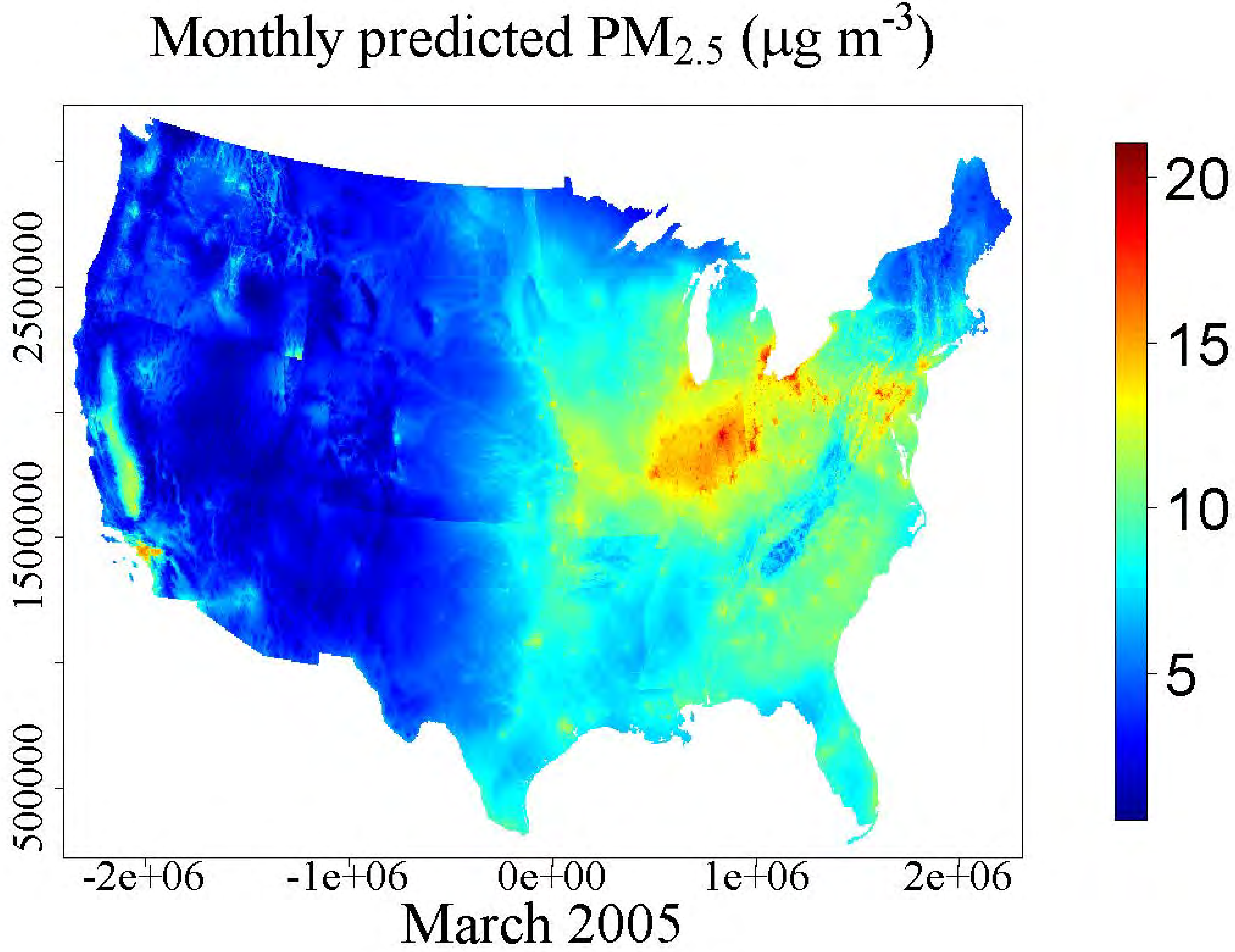
Monthly predicted PM2.5 (μg m-3)
25000000
15000000
5000000
-2e+06
-1e+06
0e+00
1e+06
2e+06
March 2005
20
15
10
5

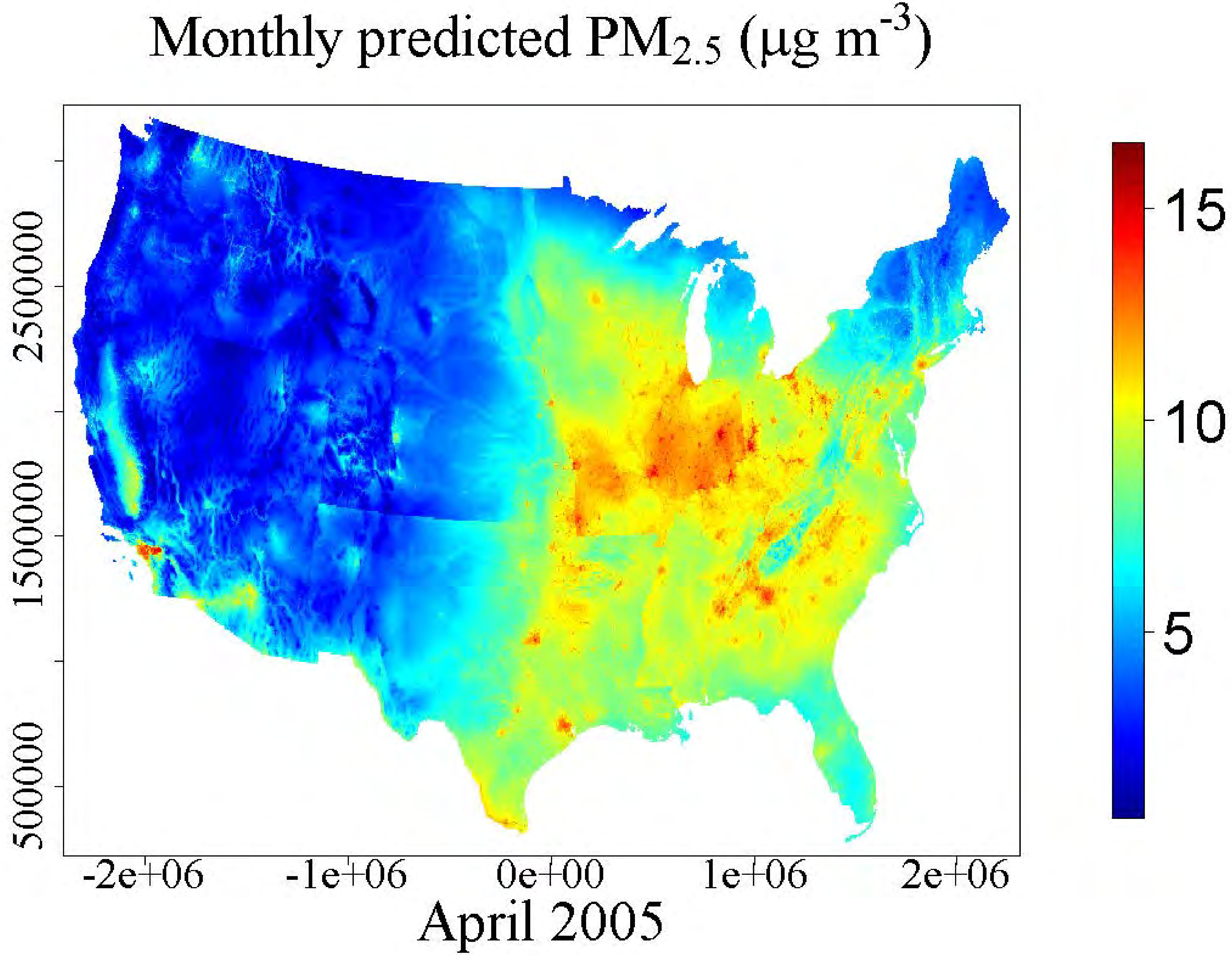

Monthly predicted $PM_{2.5}$ ($\mu g\ m^{-3}$)
25000000
15000000
5000000
-2e+06
-1e+06
0e+00
1e+06
2e+06
April 2005
15
10
5

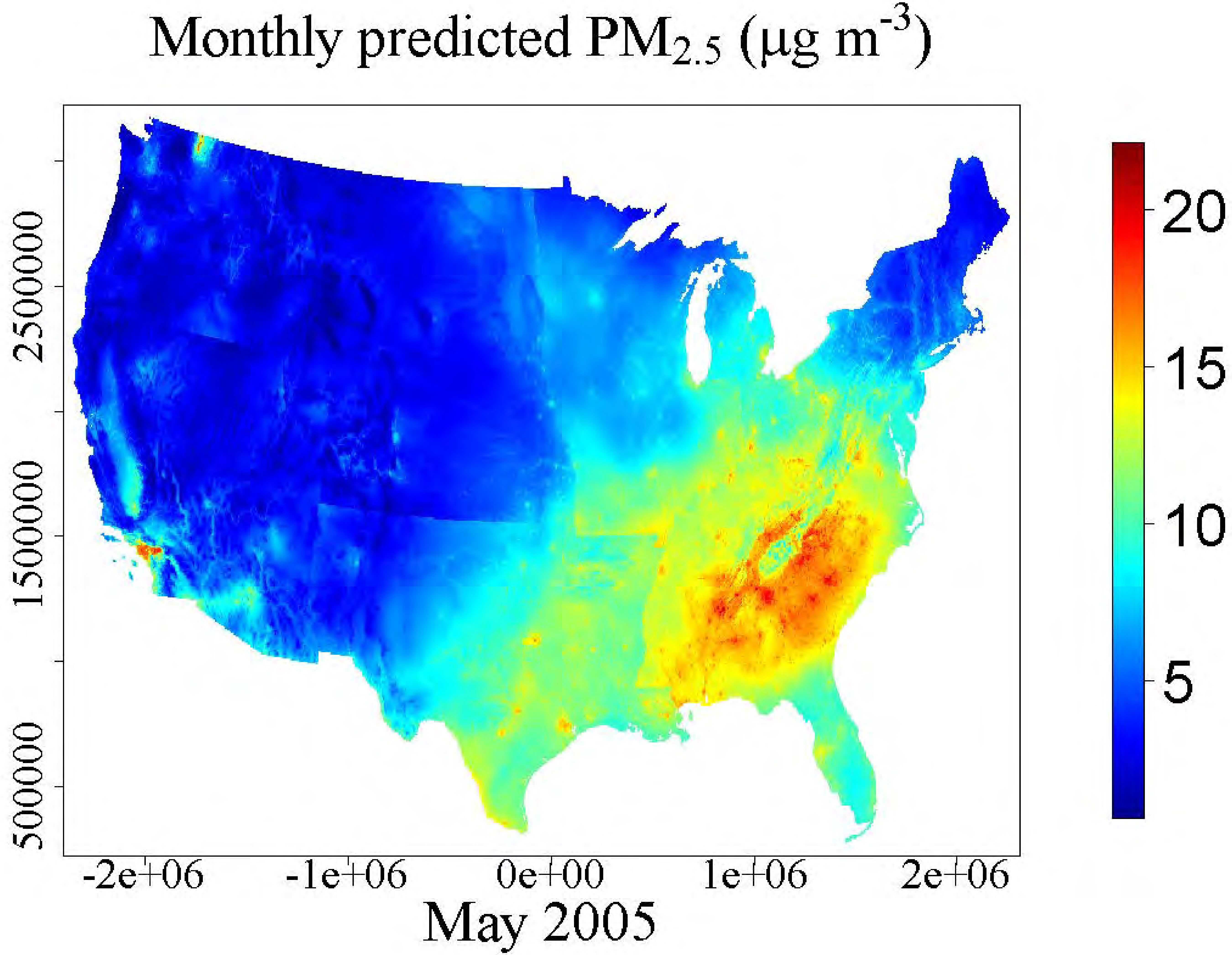

Monthly predicted $PM_{2.5}$ ($\mu g\ m^{-3}$)
25000000
15000000
5000000
-2e+06
-1e+06
0e+00
1e+06
2e+06
May 2005
20
15
10
5

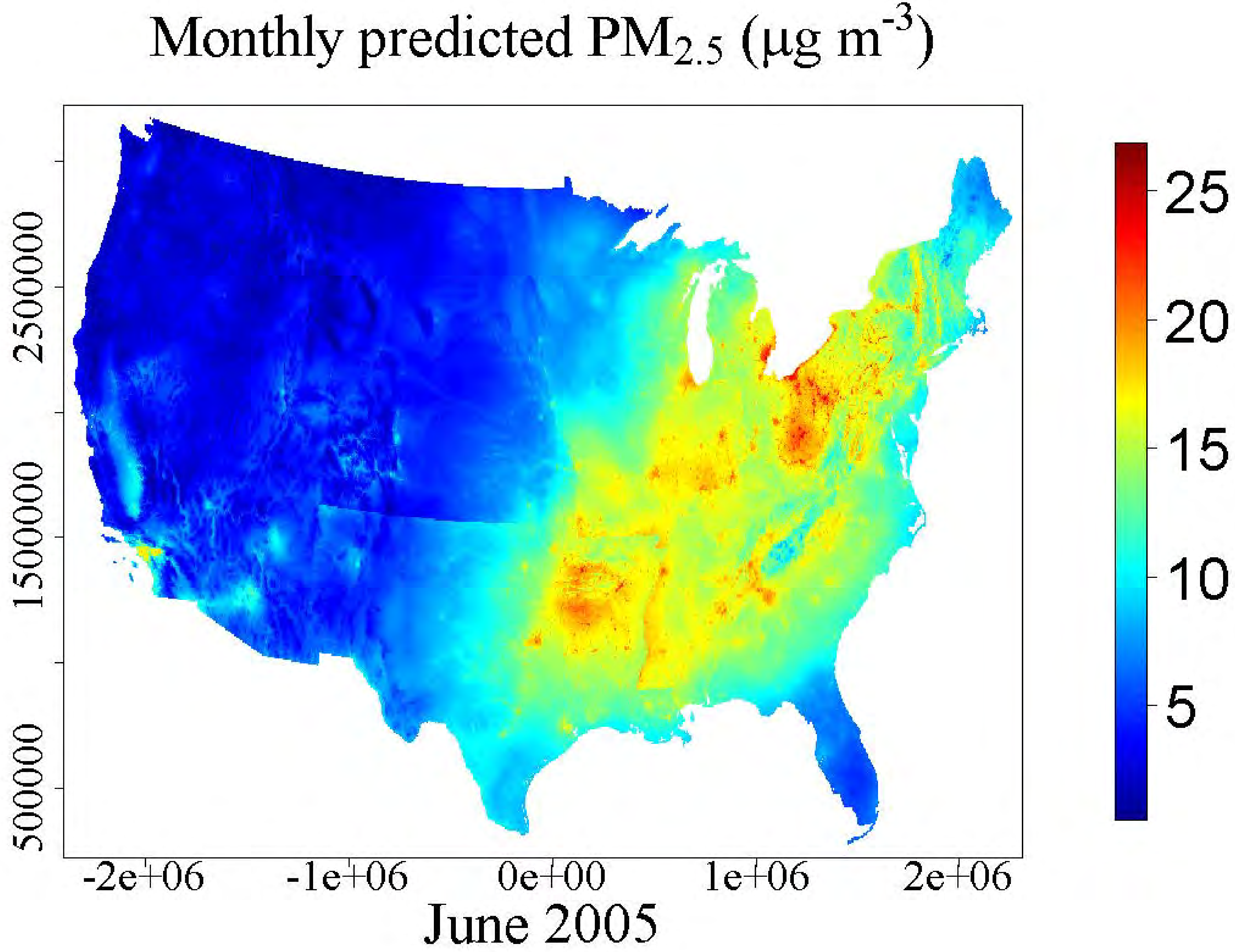

Monthly predicted $PM_{2.5}$ ($\mu g\ m^{-3}$)
25000000
15000000
5000000
-2e+06
-1e+06
0e+00
1e+06
2e+06
June 2005
25
20
15
10
5

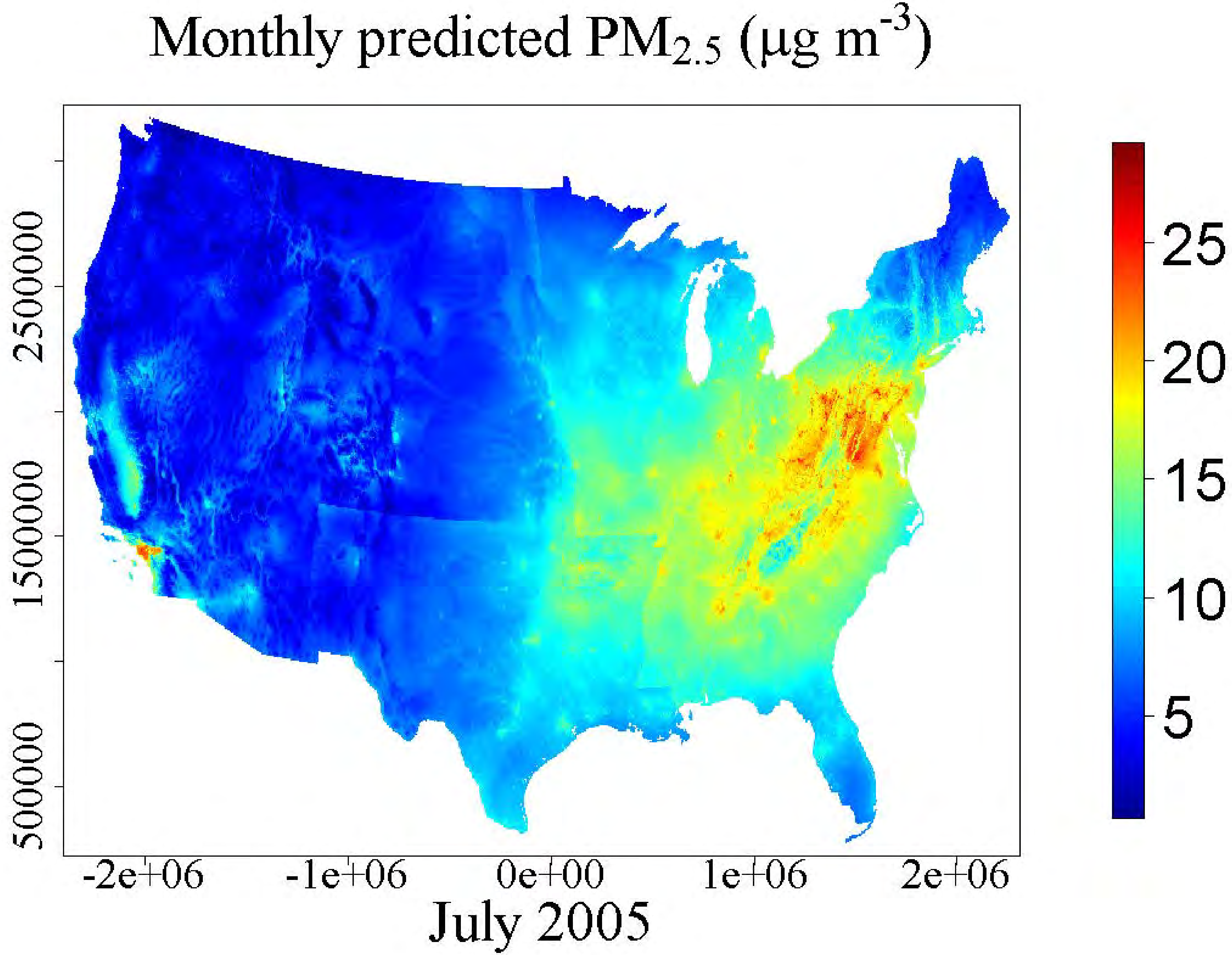
Monthly predicted $PM_{2.5}$ ($\mu g\ m^{-3}$)
25000000
15000000
5000000
-2e+06
-1e+06
0e+00
1e+06
2e+06
July 2005
25
20
15
10
5

Monthly predicted $PM_{2.5}$ ($\mu g\ m^{-3}$)

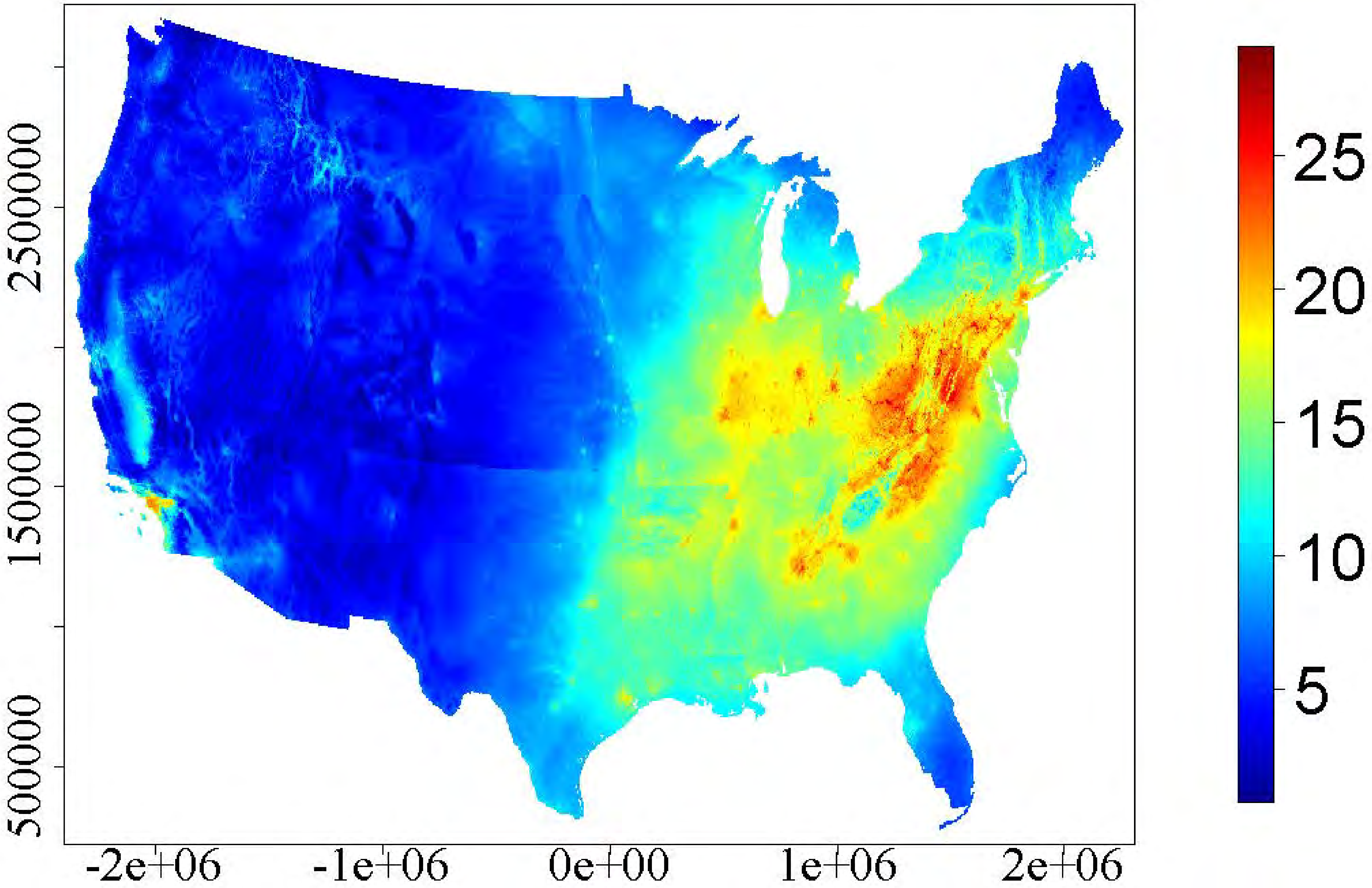

August 2005

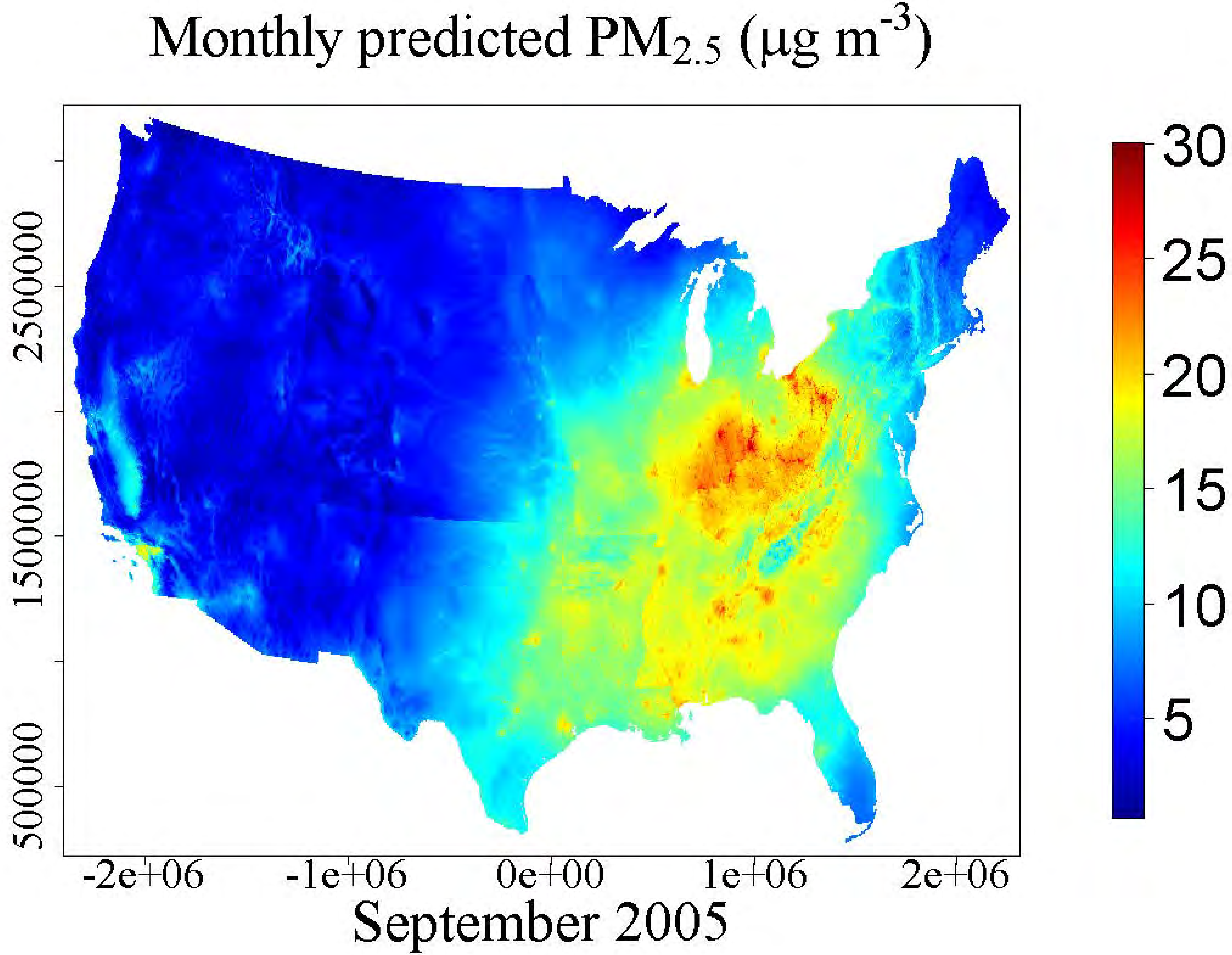
Monthly predicted PM2.5 (μg m-3)
25000000
15000000
5000000
-2e+06
-1e+06
0e+00
1e+06
2e+06
September 2005
30
25
20
15
10
5

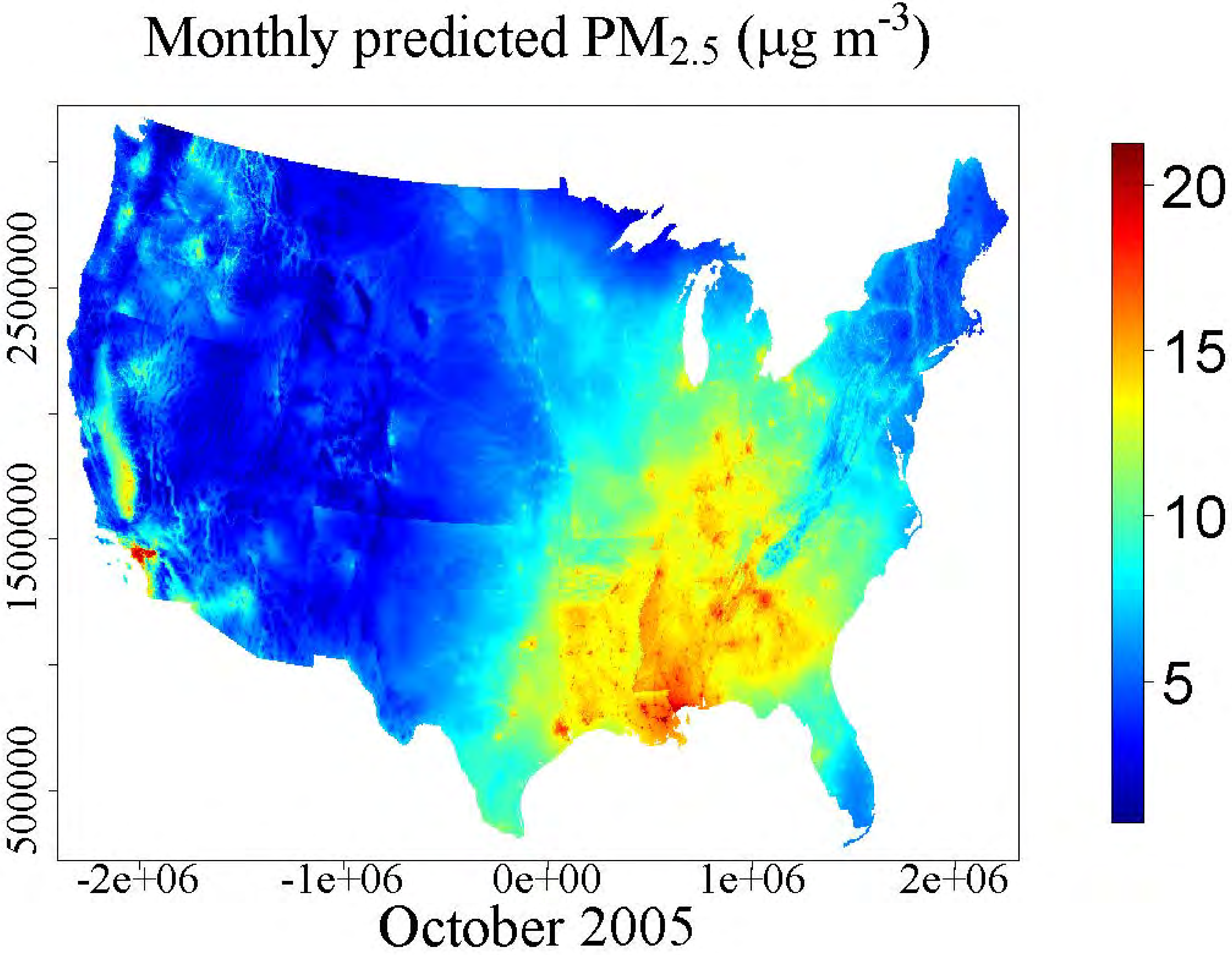
Monthly predicted PM2.5 (μg m-3)
25000000
15000000
5000000
-2e+06
-1e+06
0e+00
1e+06
2e+06
October 2005
20
15
10
5

Monthly predicted $PM_{2.5}$ ($\mu g\ m^{-3}$)

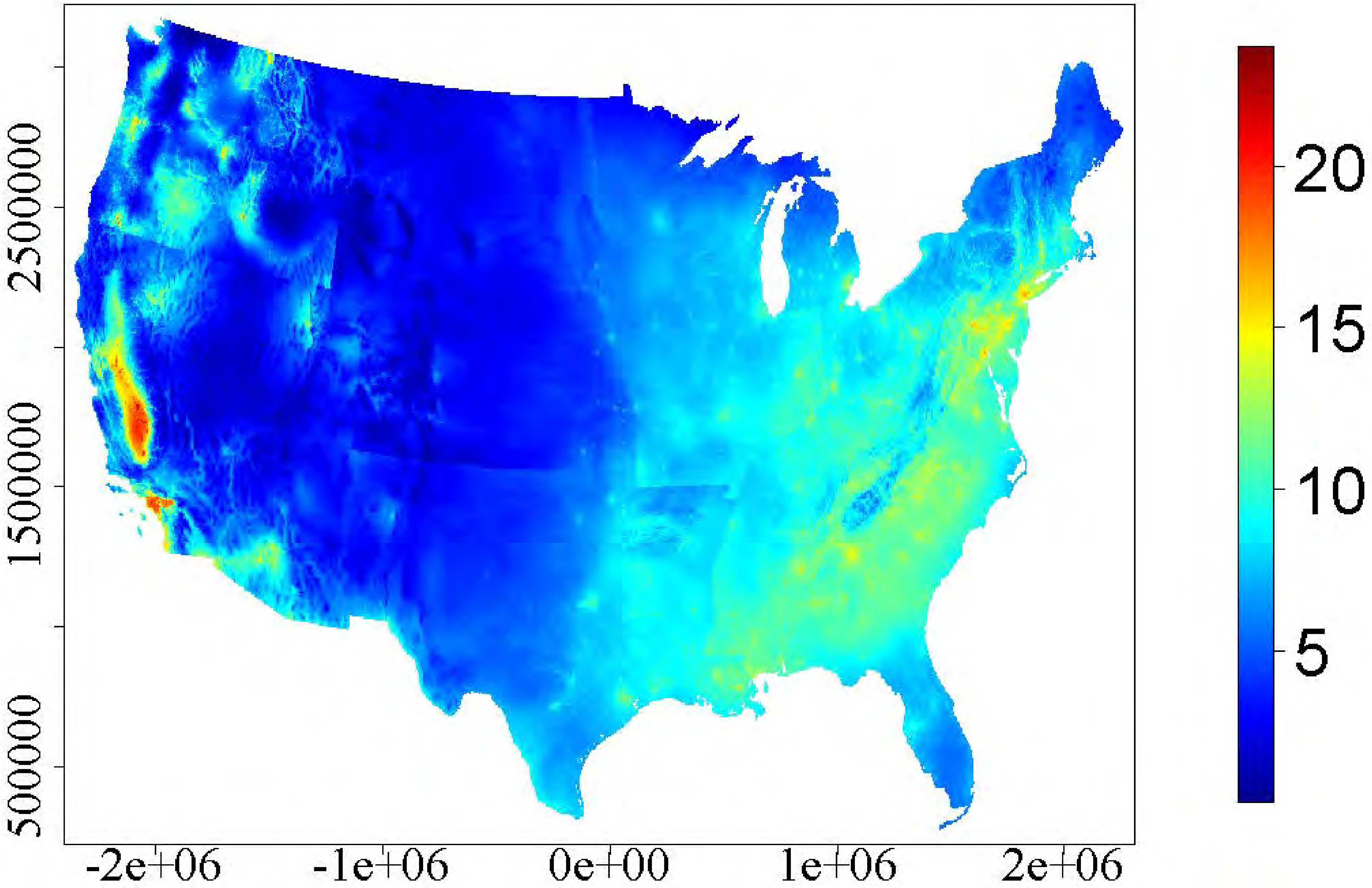

November 2005

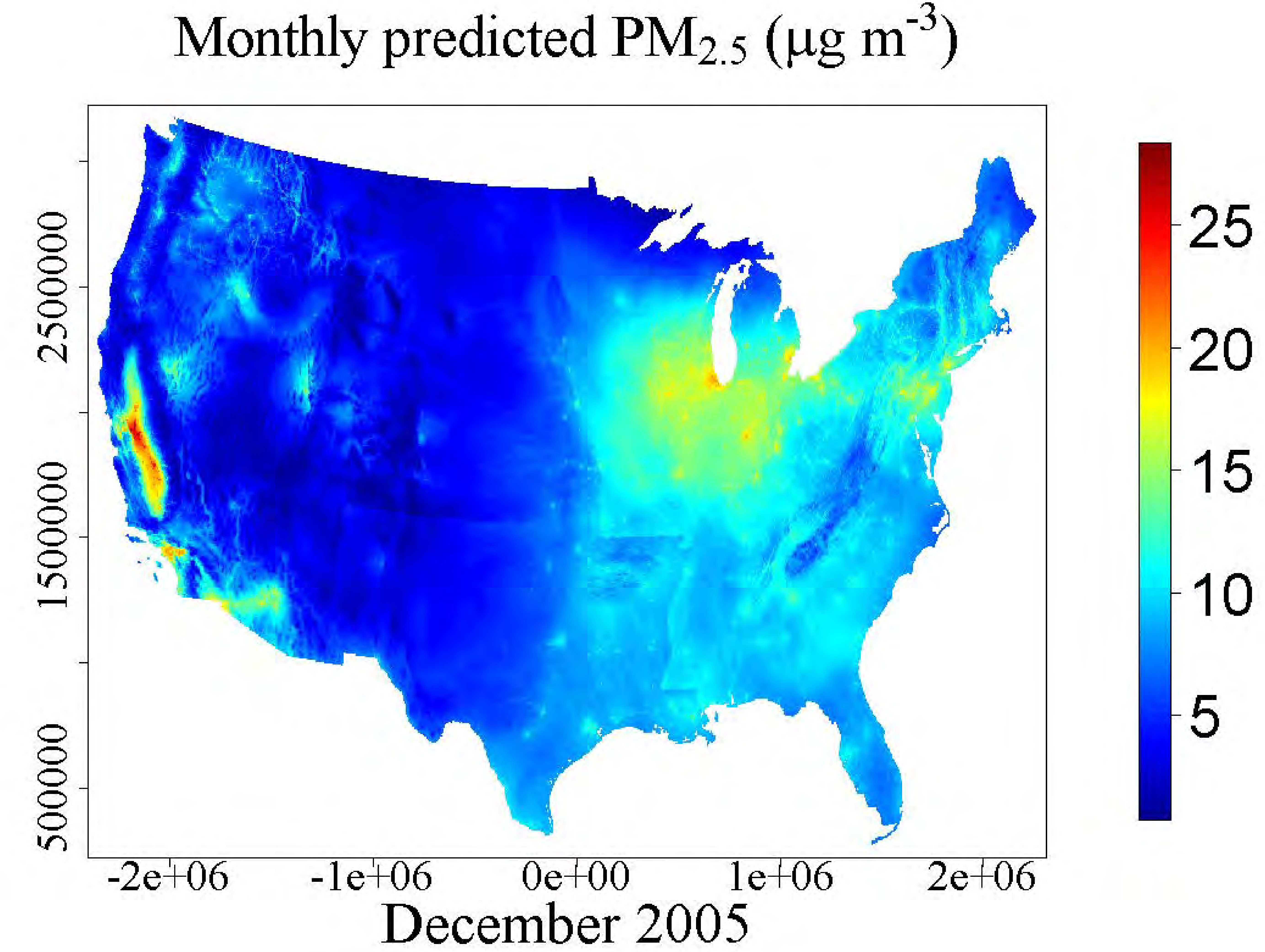

Monthly predicted PM2.5 (μg m-3)
25000000
15000000
5000000
-2e+06
-1e+06
0e+00
1e+06
2e+06
December 2005
25
20
15
10
5

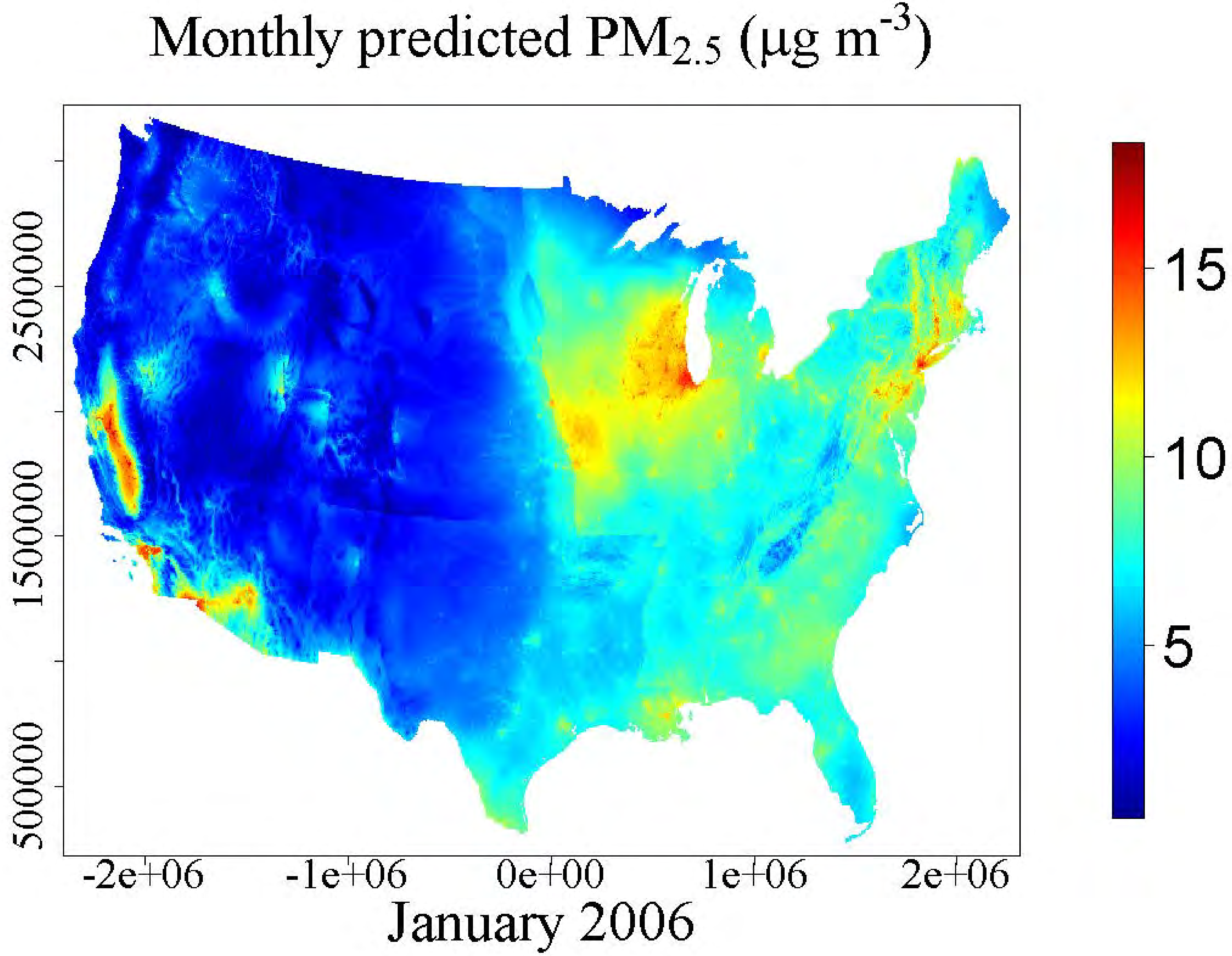
Monthly predicted PM2.5 (µg m-3)
25000000
15000000
5000000
-2e+06
-1e+06
0e+00
1e+06
2e+06
January 2006
15
10
5

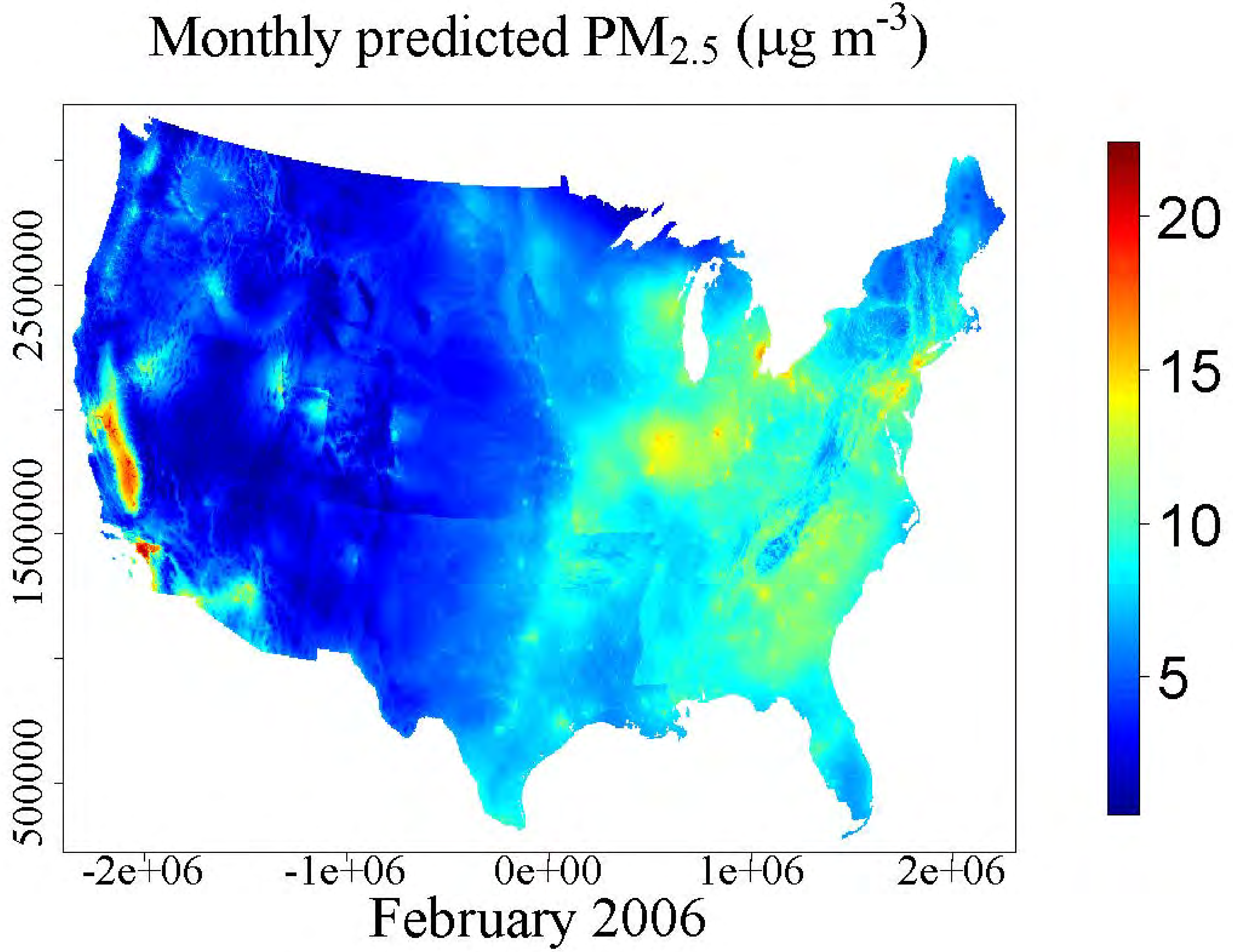
Monthly predicted PM2.5 (μg m-3)
25000000
15000000
5000000
-2e+06
-1e+06
0e+00
1e+06
2e+06
20
15
10
5
February 2006

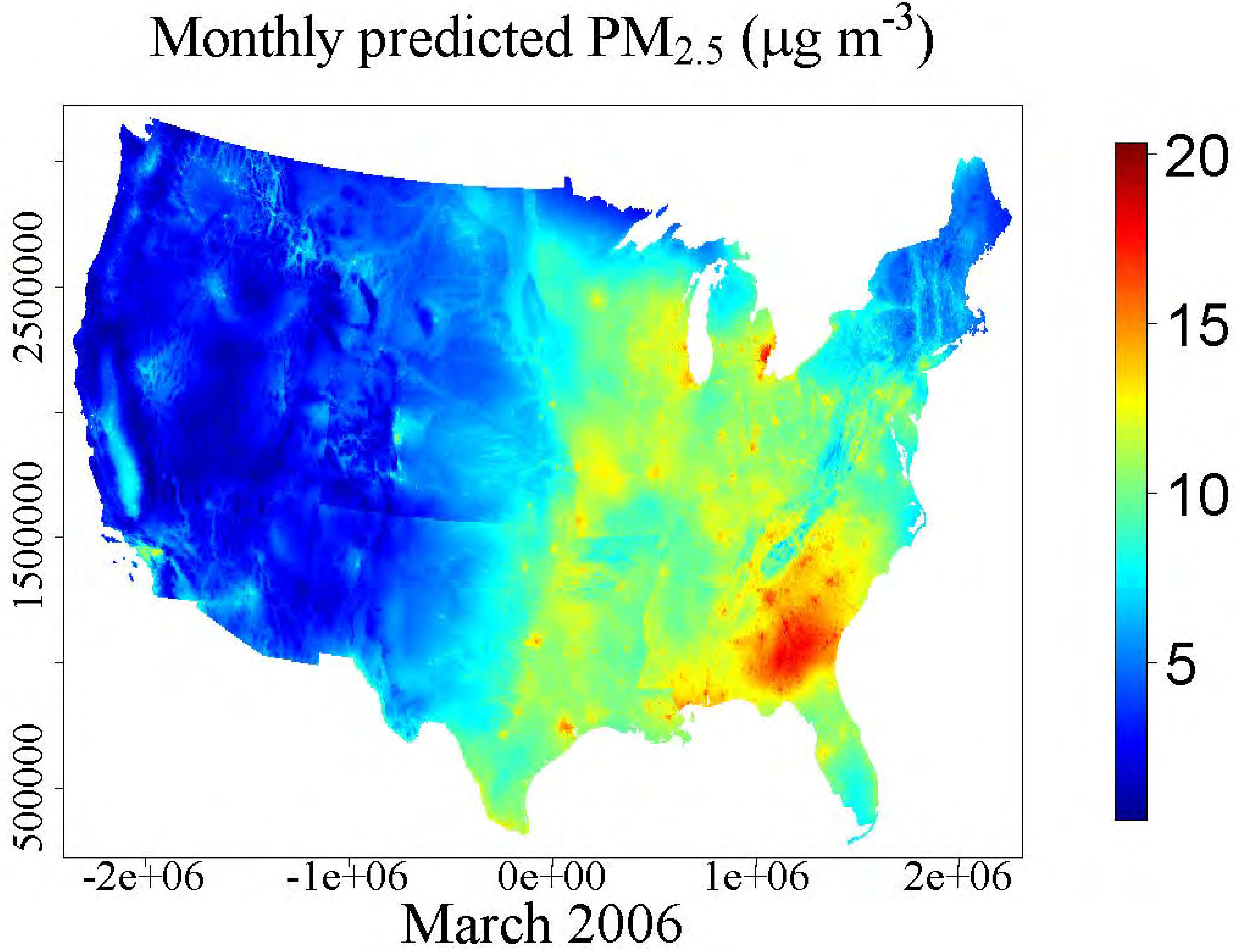

Monthly predicted PM2.5 (μg m-3)
2500000
1500000
5000000
-2e+06
-1e+06
0e+00
1e+06
2e+06
20
15
10
5
March 2006

Monthly predicted $PM_{2.5}$ ($\mu g\ m^{-3}$)

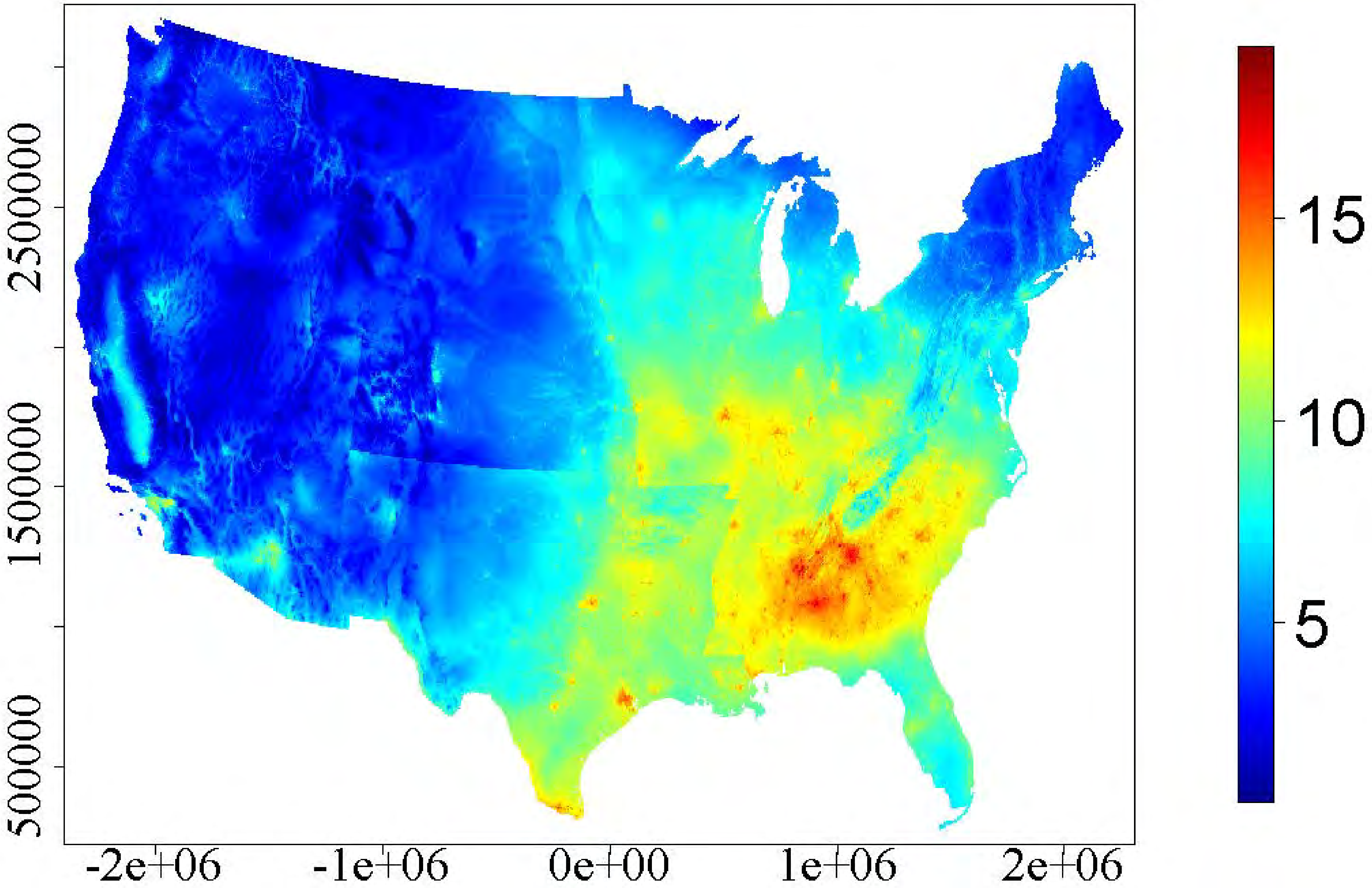

April 2006

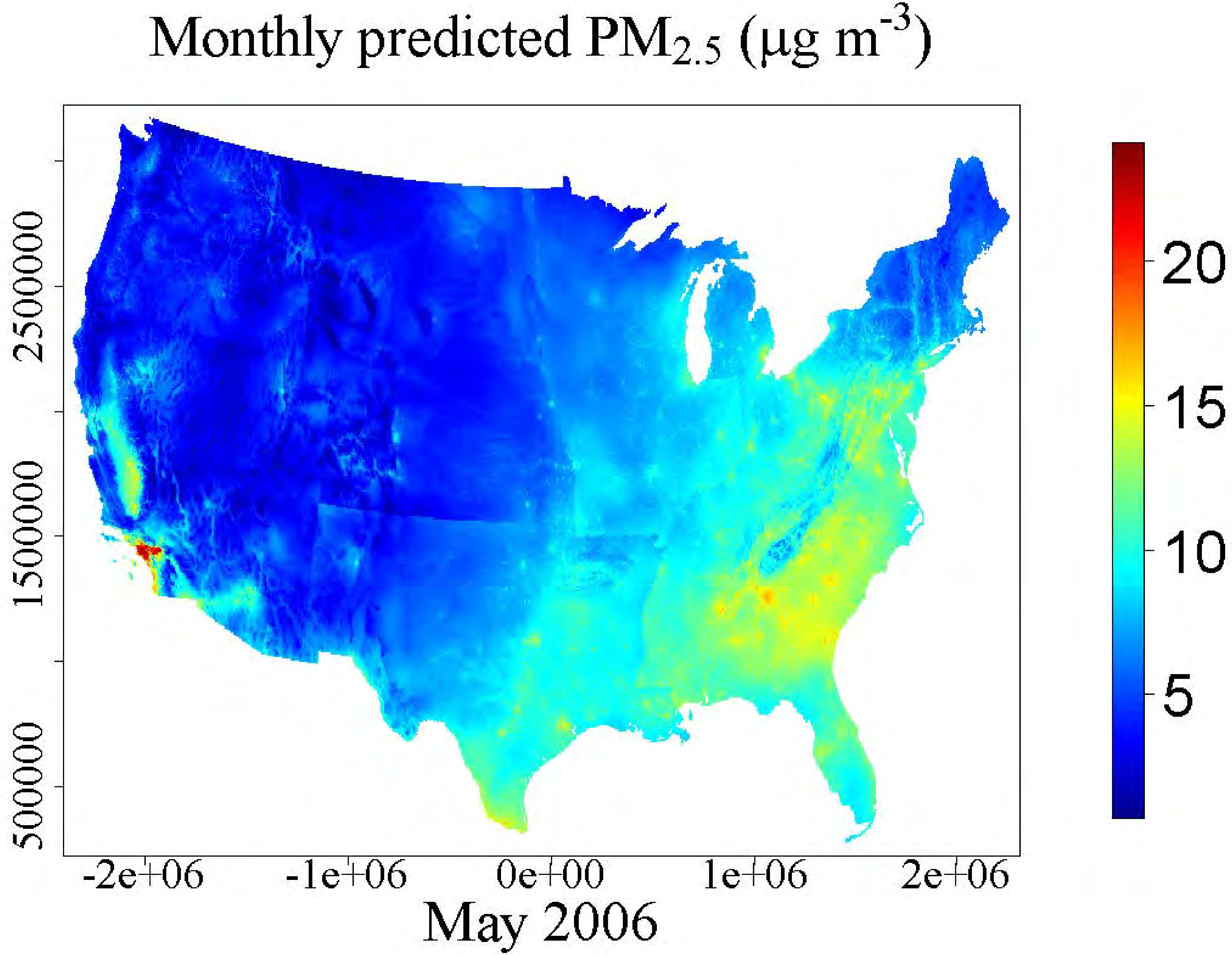
Monthly predicted PM2.5 (μg m-3)
25000000
15000000
5000000
-2e+06
-1e+06
0e+00
1e+06
2e+06
May 2006
20
15
10
5

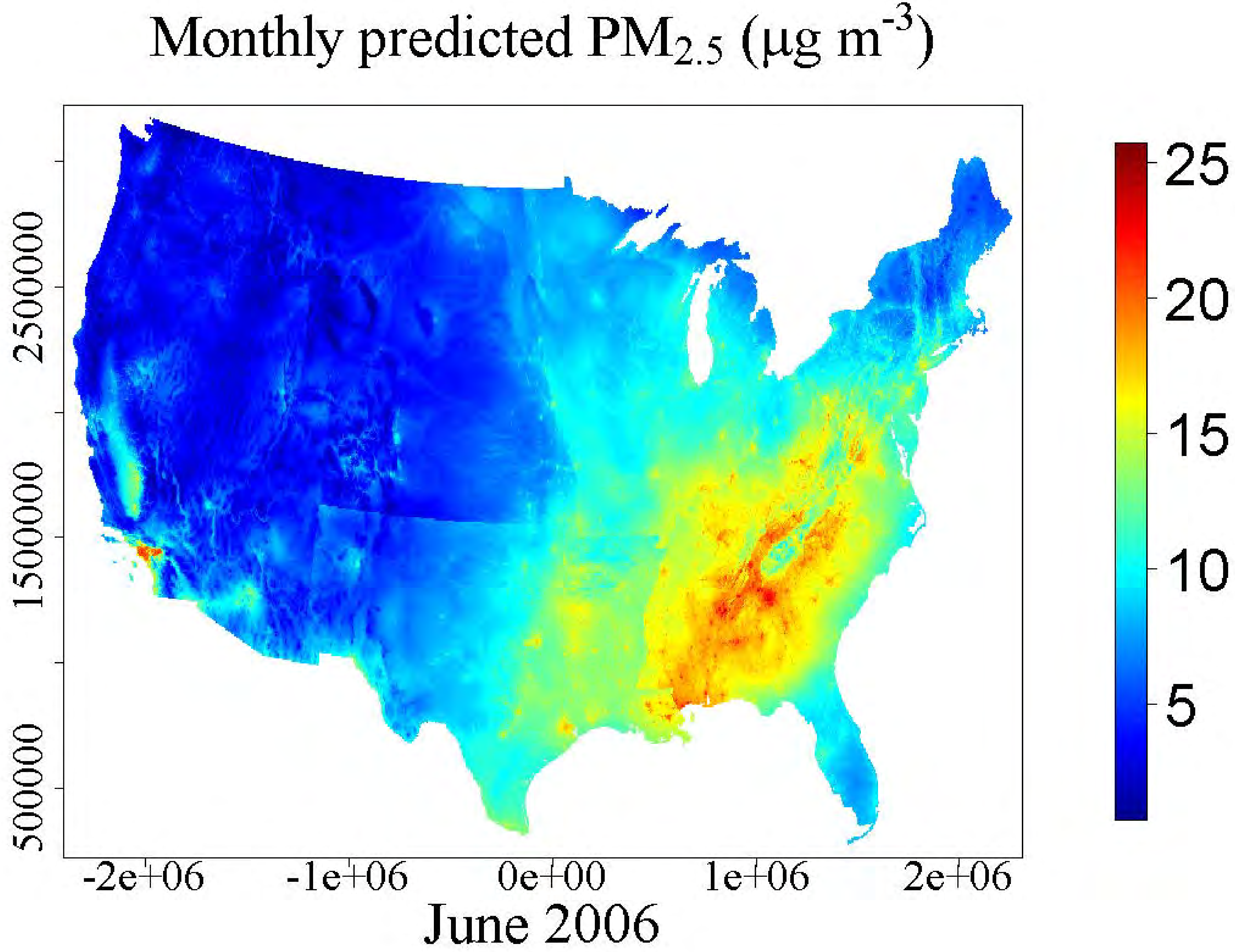

Monthly predicted PM2.5 (μg m-3)
25000000
15000000
5000000
-2e+06
-1e+06
0e+00
1e+06
2e+06
June 2006
25
20
15
10
5

Monthly predicted $PM_{2.5}$ ($\mu g\ m^{-3}$)

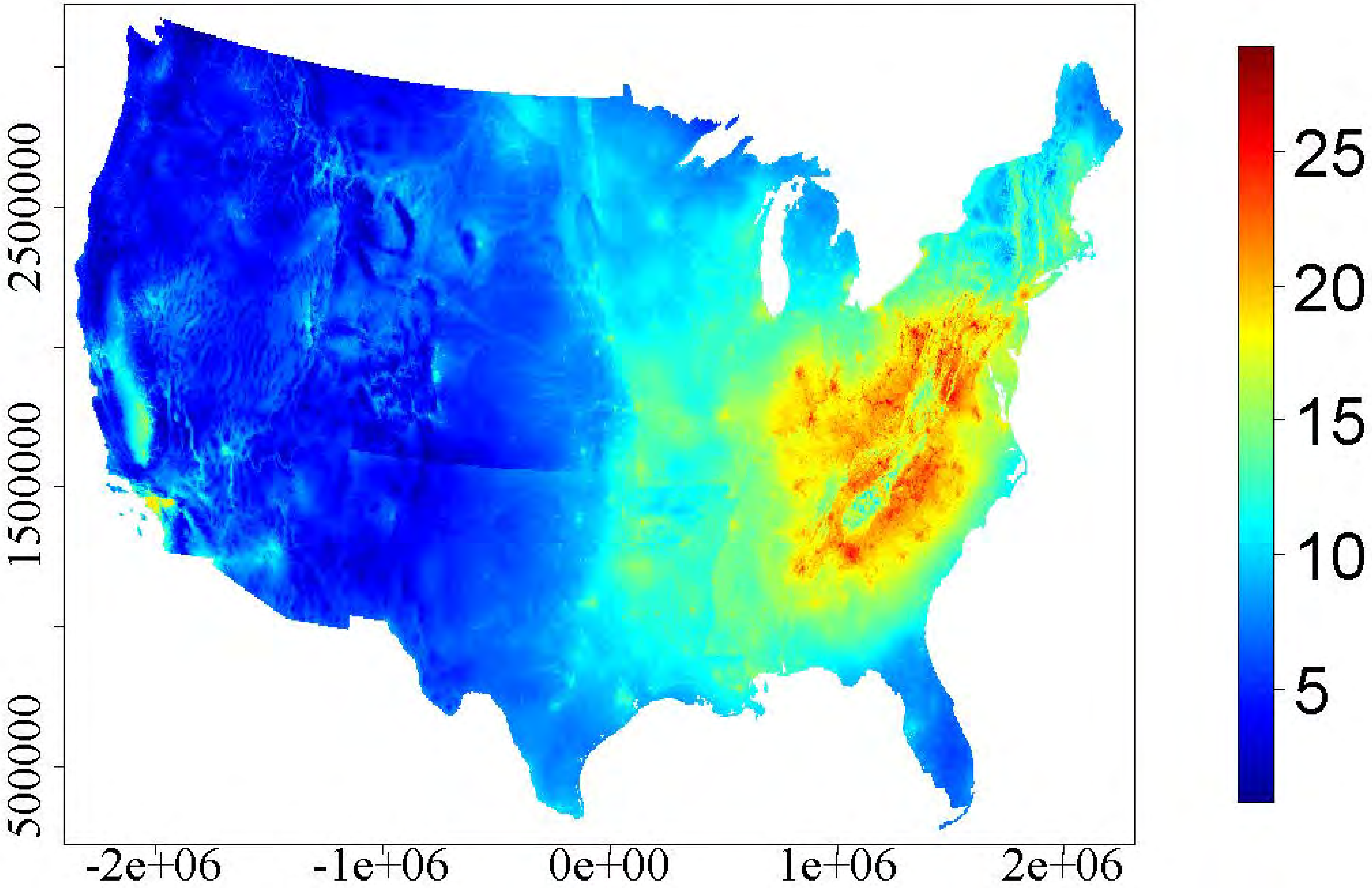

July 2006

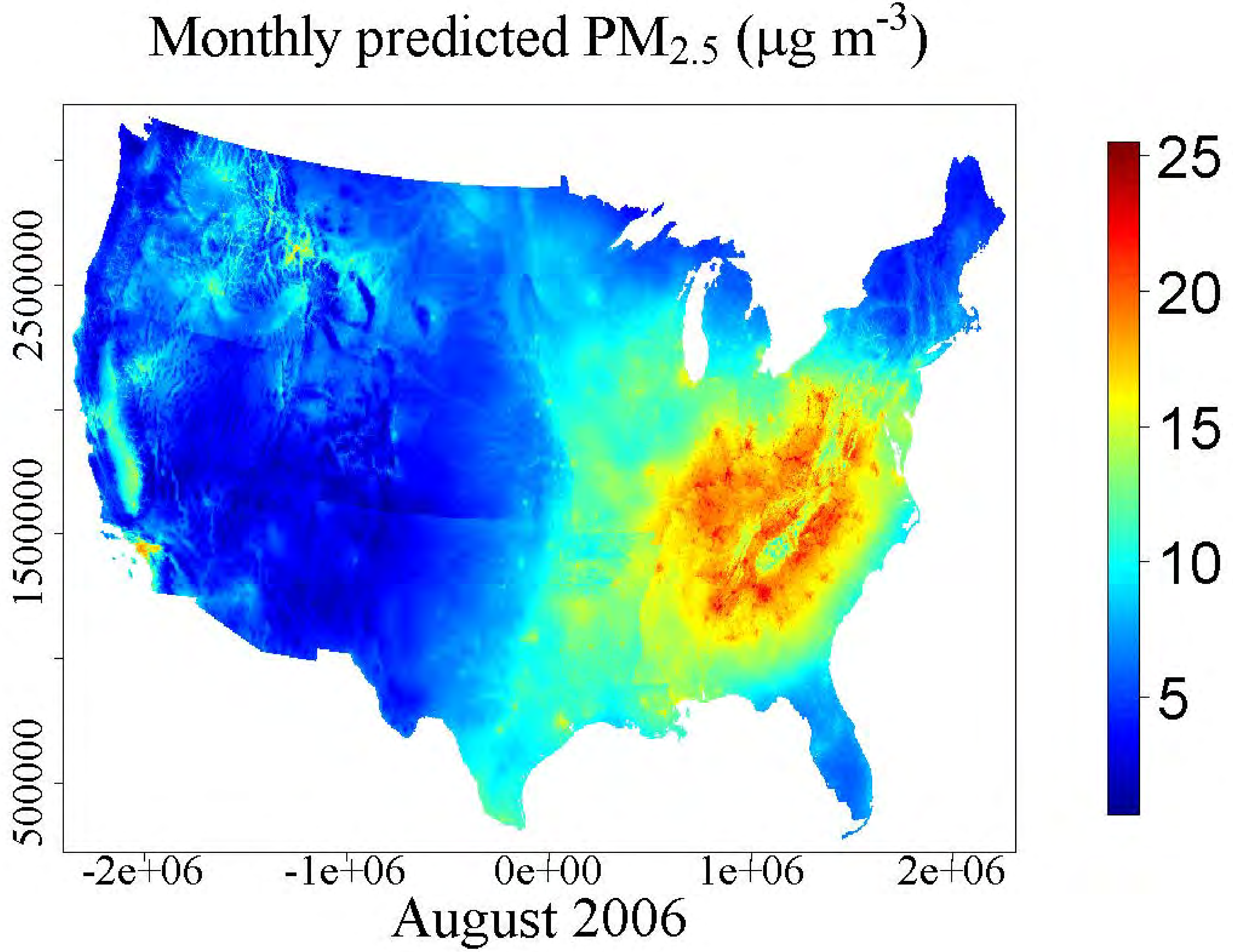

Monthly predicted PM2.5 (µg m-3)
25000000
15000000
5000000
-2e+06
-1e+06
0e+00
1e+06
2e+06
25
20
15
10
5
August 2006

Monthly predicted $PM_{2.5}$ ($\mu g\ m^{-3}$)

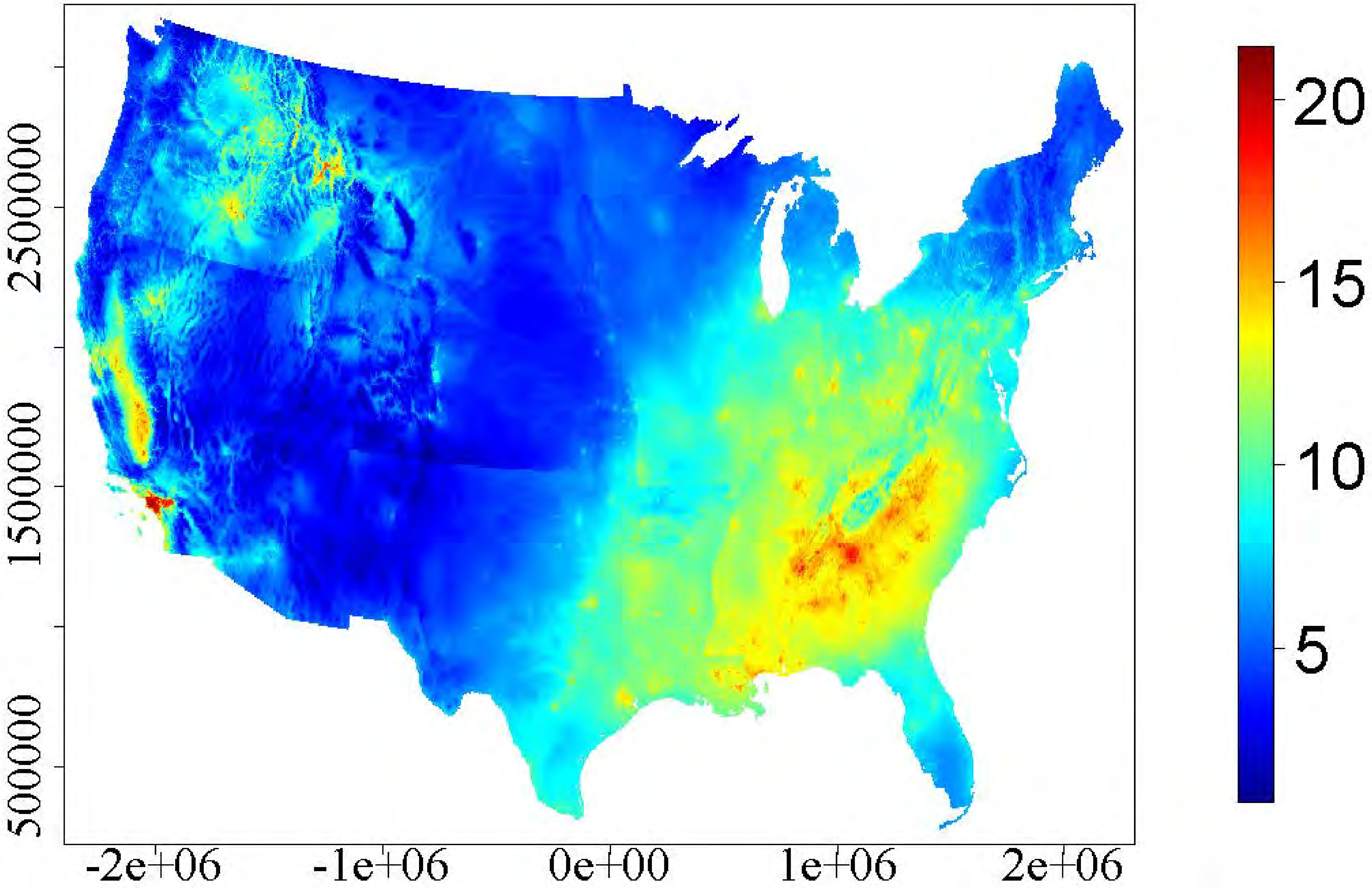

September 2006

Monthly predicted $PM_{2.5}$ ($\mu g\ m^{-3}$)

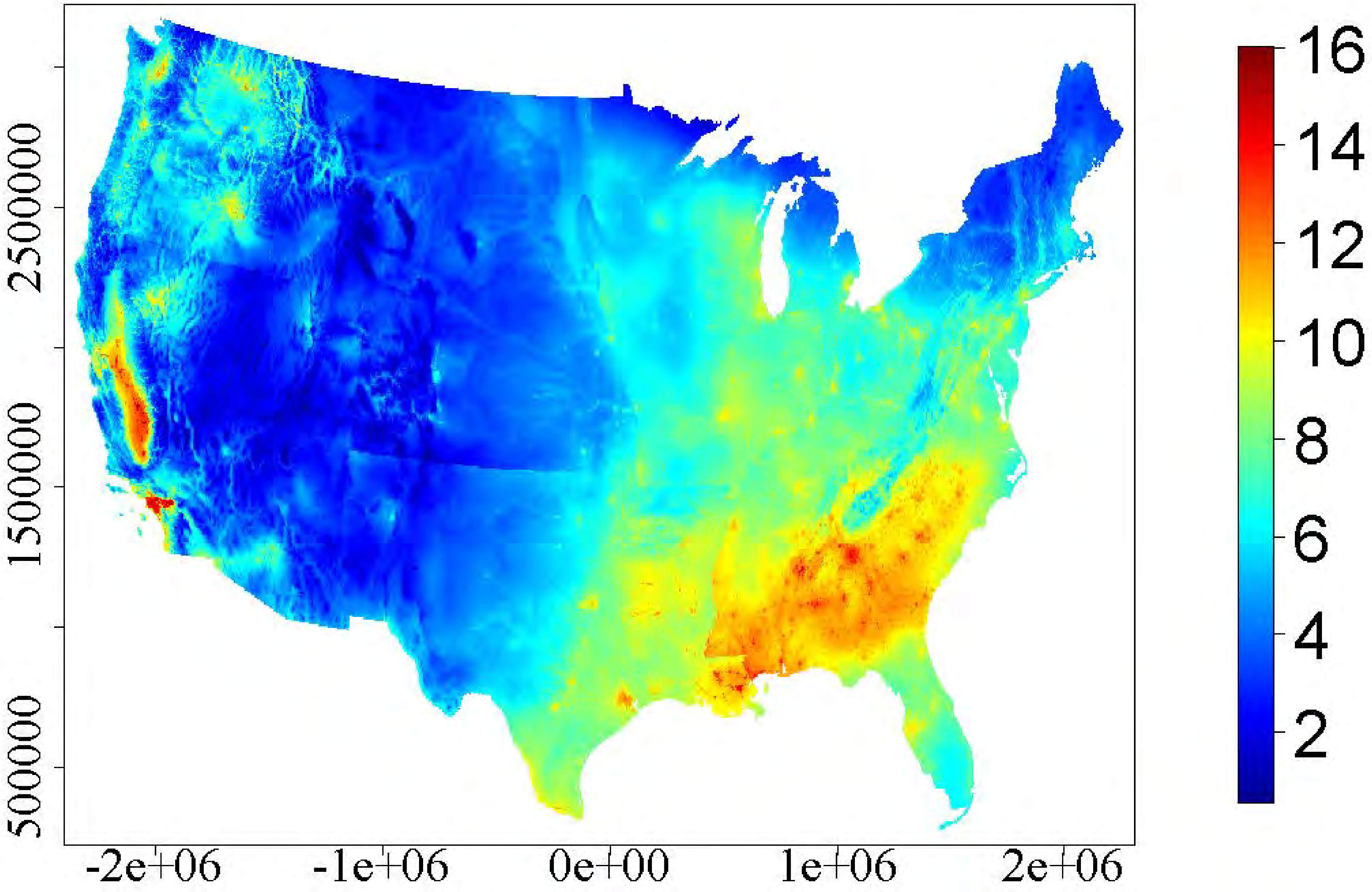

October 2006

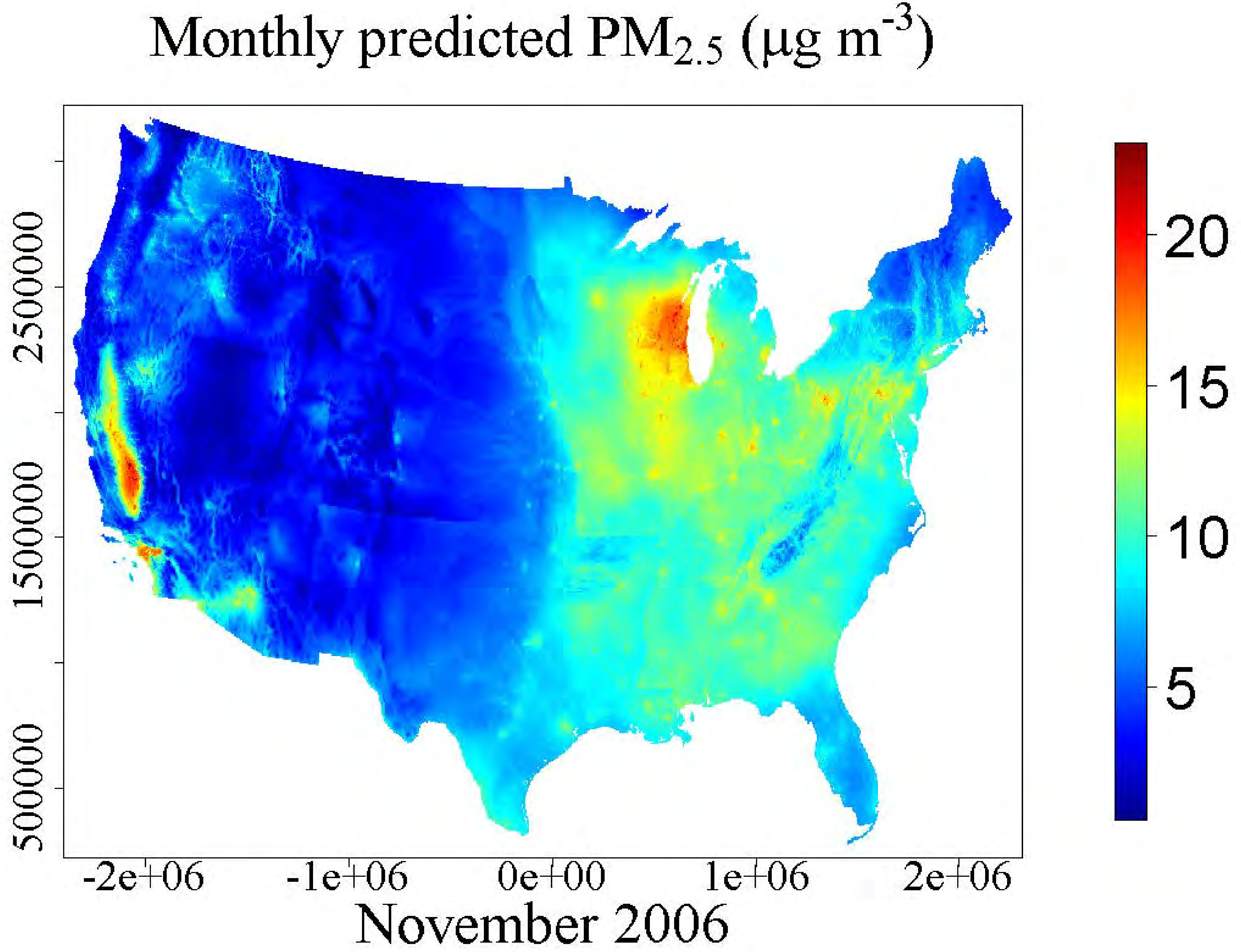

Monthly predicted PM2.5 (μg m-3)
25000000
15000000
5000000
-2e+06
-1e+06
0e+00
1e+06
2e+06
20
15
10
5
November 2006

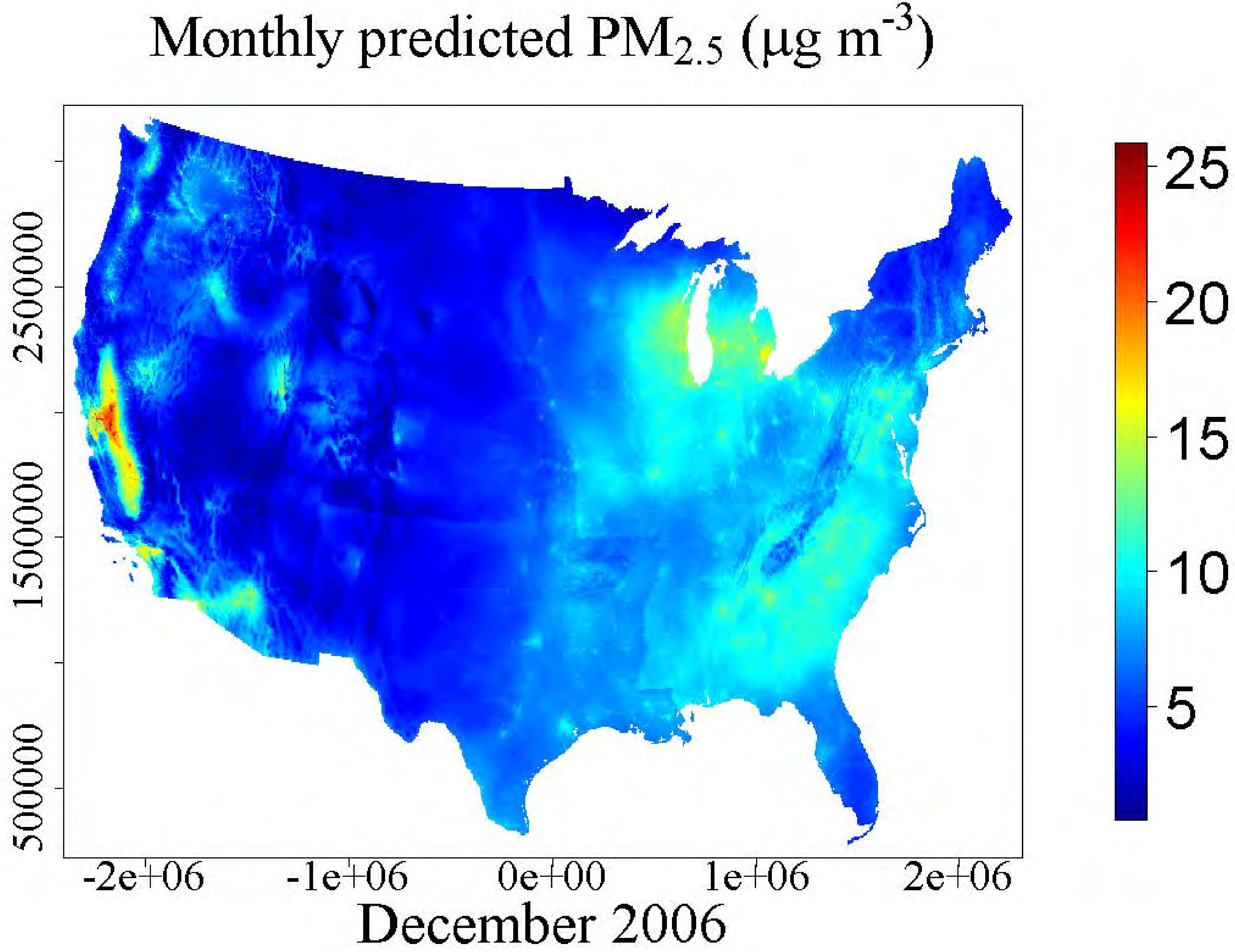

Monthly predicted PM2.5 (μg m-3)
25000000
15000000
5000000
-2e+06
-1e+06
0e+00
1e+06
2e+06
25
20
15
10
5
December 2006

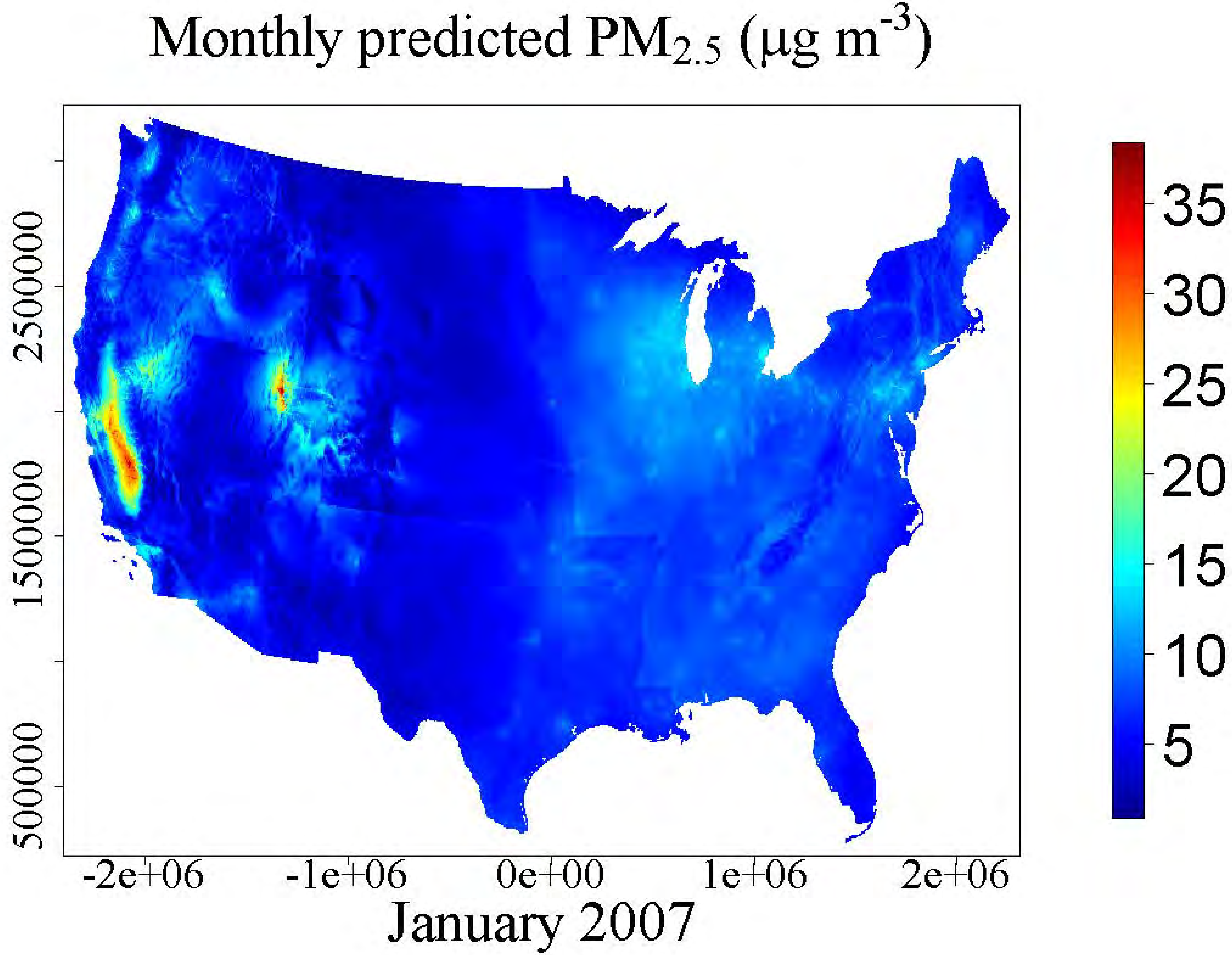

Monthly predicted PM2.5 (μg m-3)
25000000
15000000
5000000
-2e+06
-1e+06
0e+00
1e+06
2e+06
January 2007
35
30
25
20
15
10
5

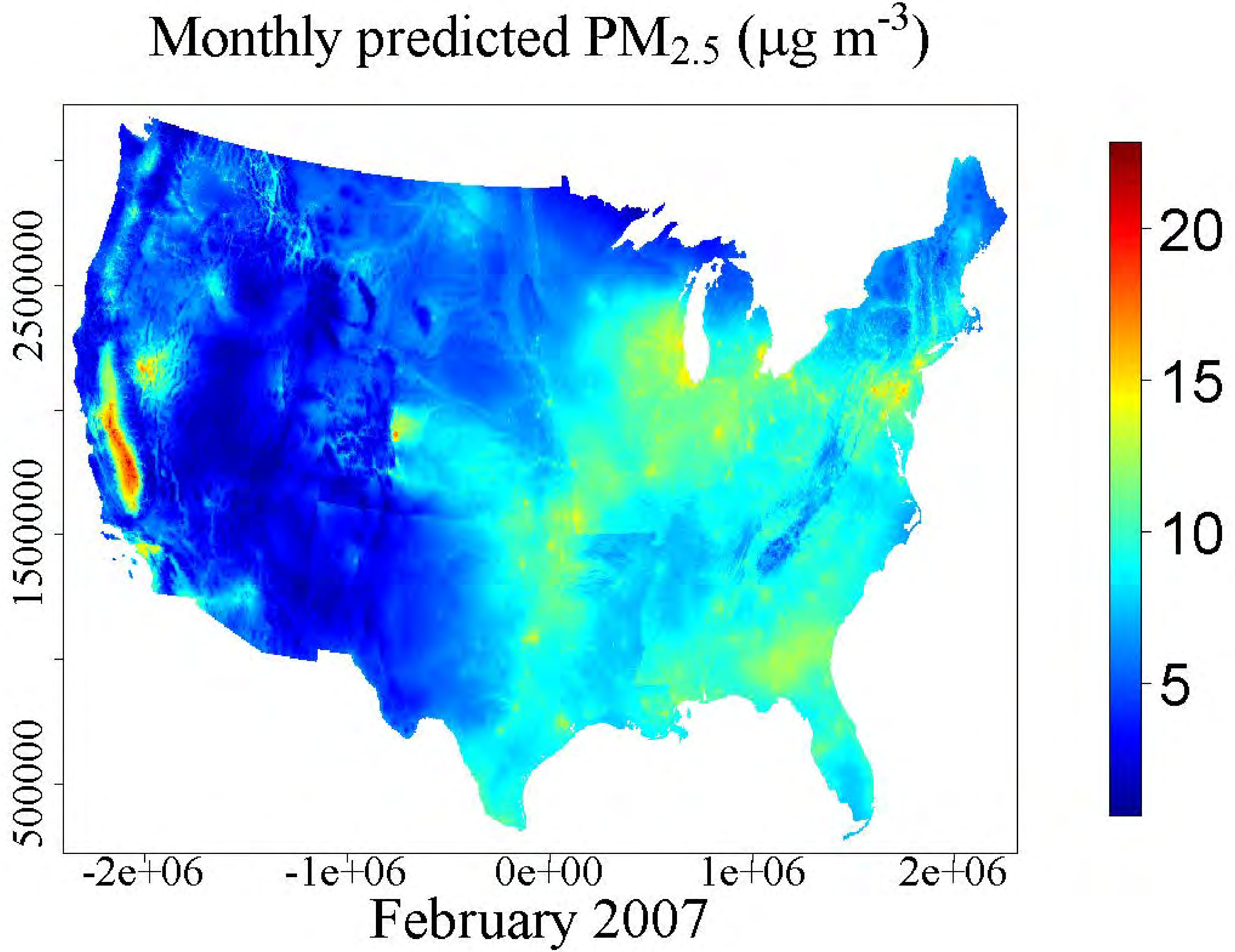
Monthly predicted PM2.5 (μg m-3)
25000000
15000000
5000000
-2e+06
-1e+06
0e+00
1e+06
2e+06
20
15
10
5
February 2007

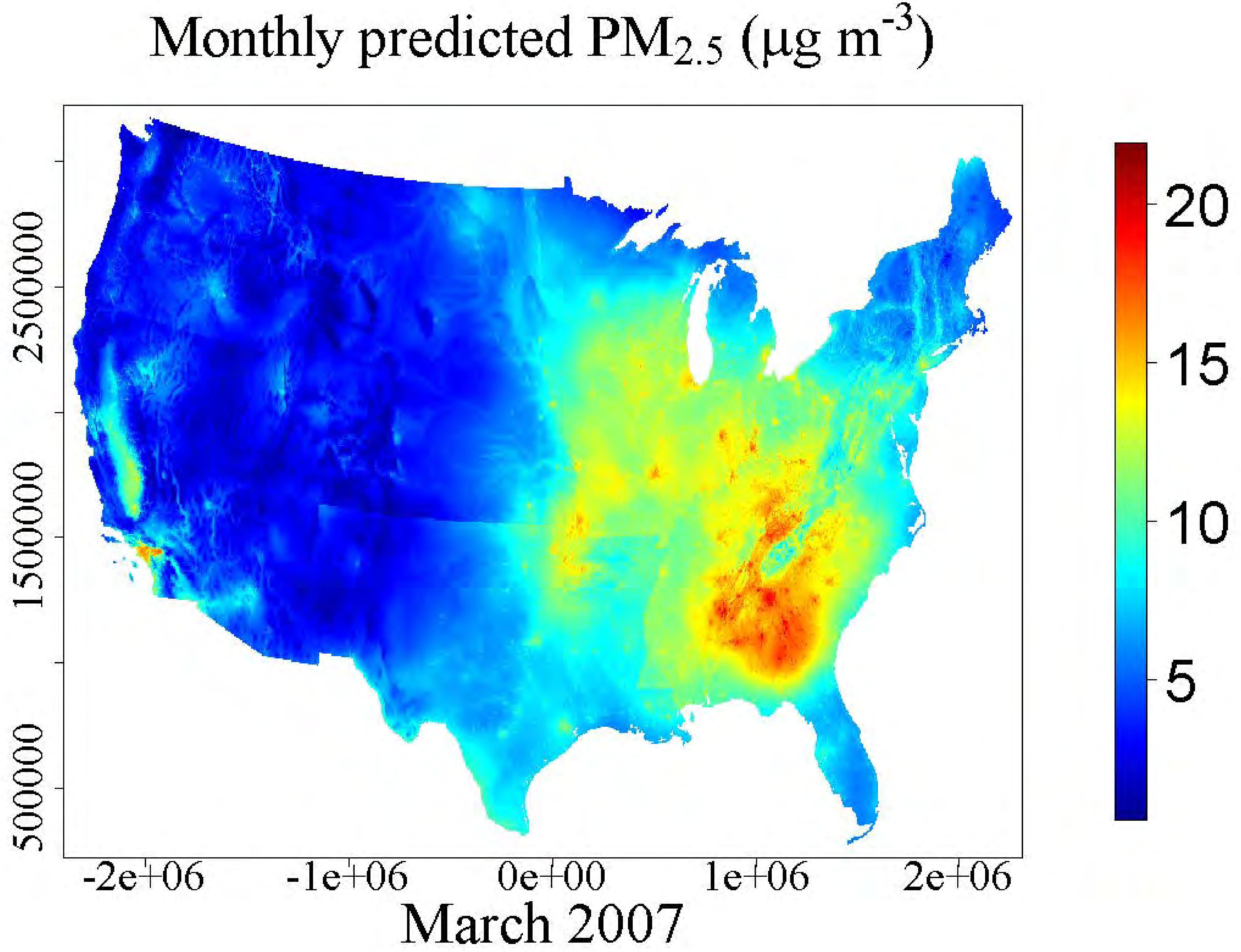

Monthly predicted $PM_{2.5}$ ($\mu g\ m^{-3}$)
5000000
15000000
25000000
-2e+06
-1e+06
0e+00
1e+06
2e+06
March 2007
20
15
10
5

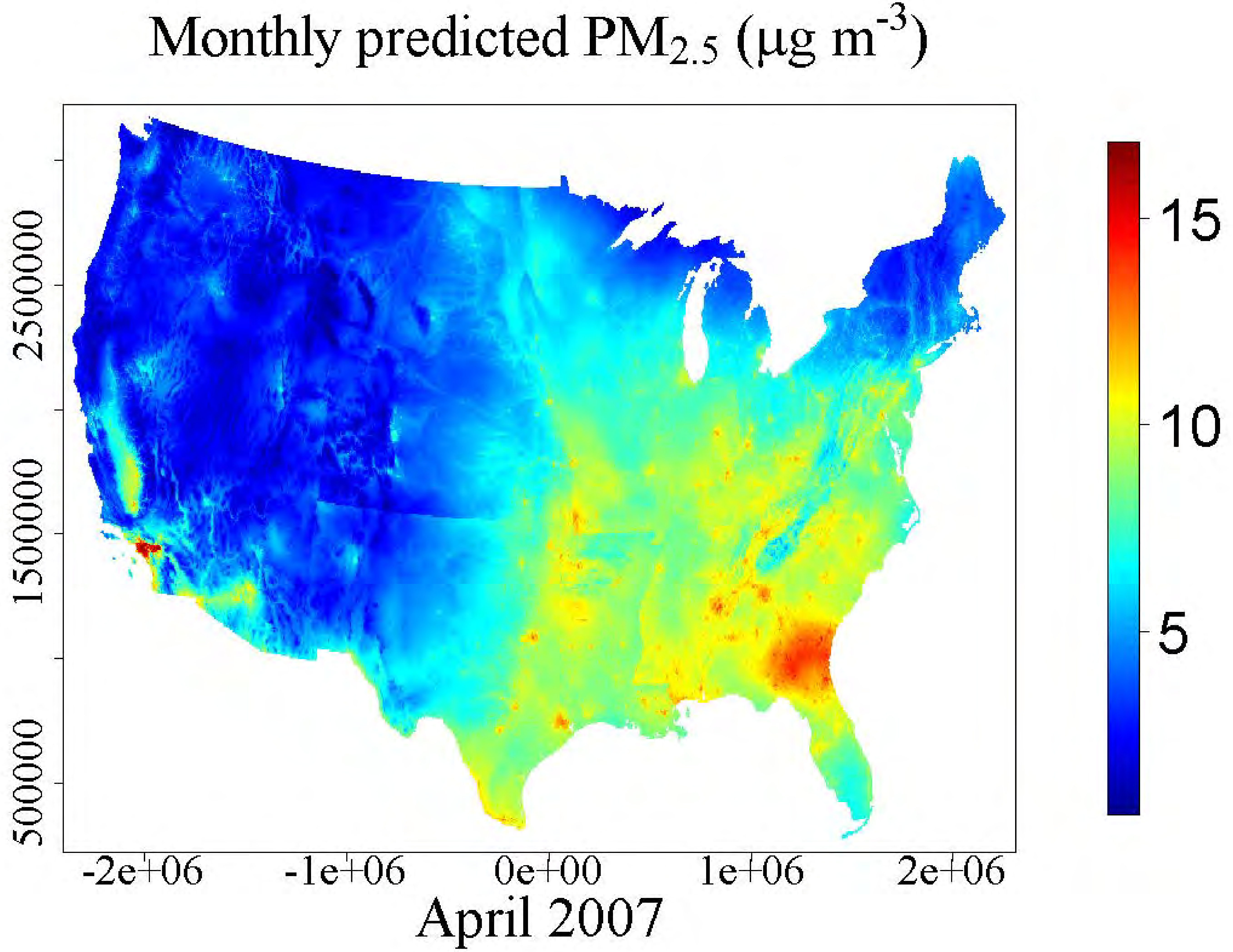
Monthly predicted PM2.5 (μg m-3)
25000000
15000000
5000000
-2e+06
-1e+06
0e+00
1e+06
2e+06
15
10
5
April 2007

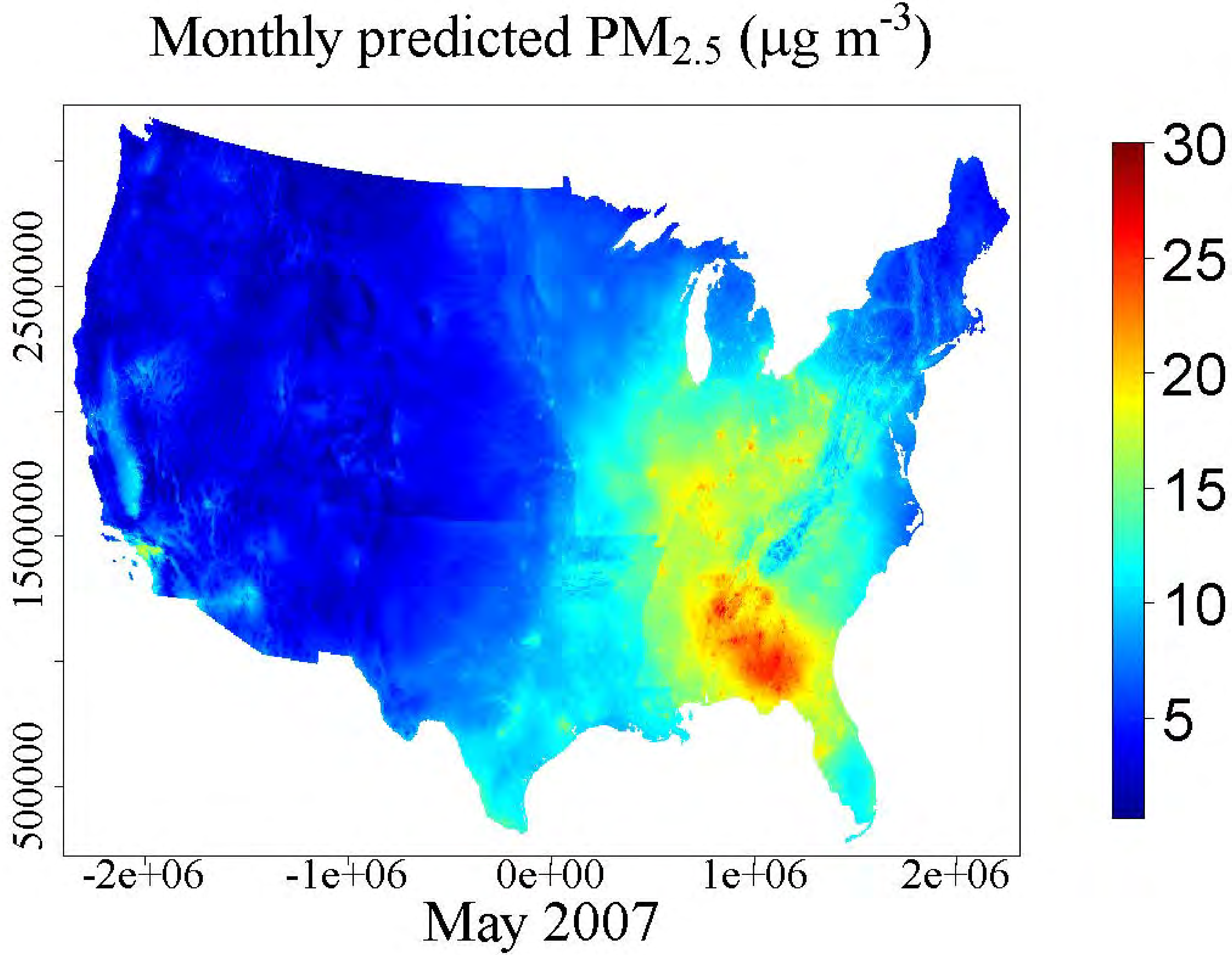
Monthly predicted $PM_{2.5}$ ($\mu g\ m^{-3}$)
25000000
15000000
5000000
-2e+06
-1e+06
0e+00
1e+06
2e+06
May 2007
30
25
20
15
10
5

Monthly predicted $PM_{2.5}$ ($\mu g\ m^{-3}$)

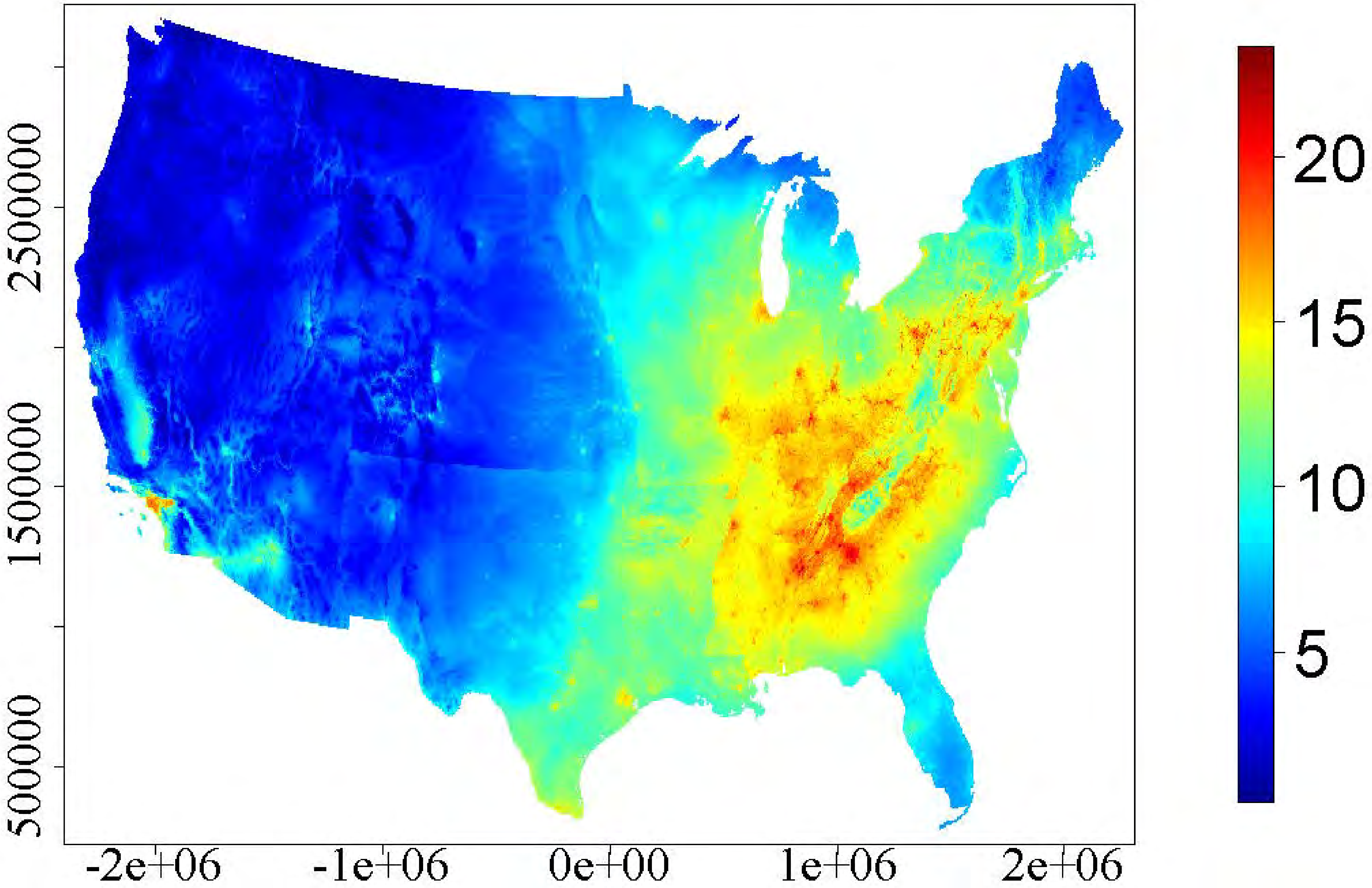

June 2007

Monthly predicted $PM_{2.5}$ ($\mu g\ m^{-3}$)

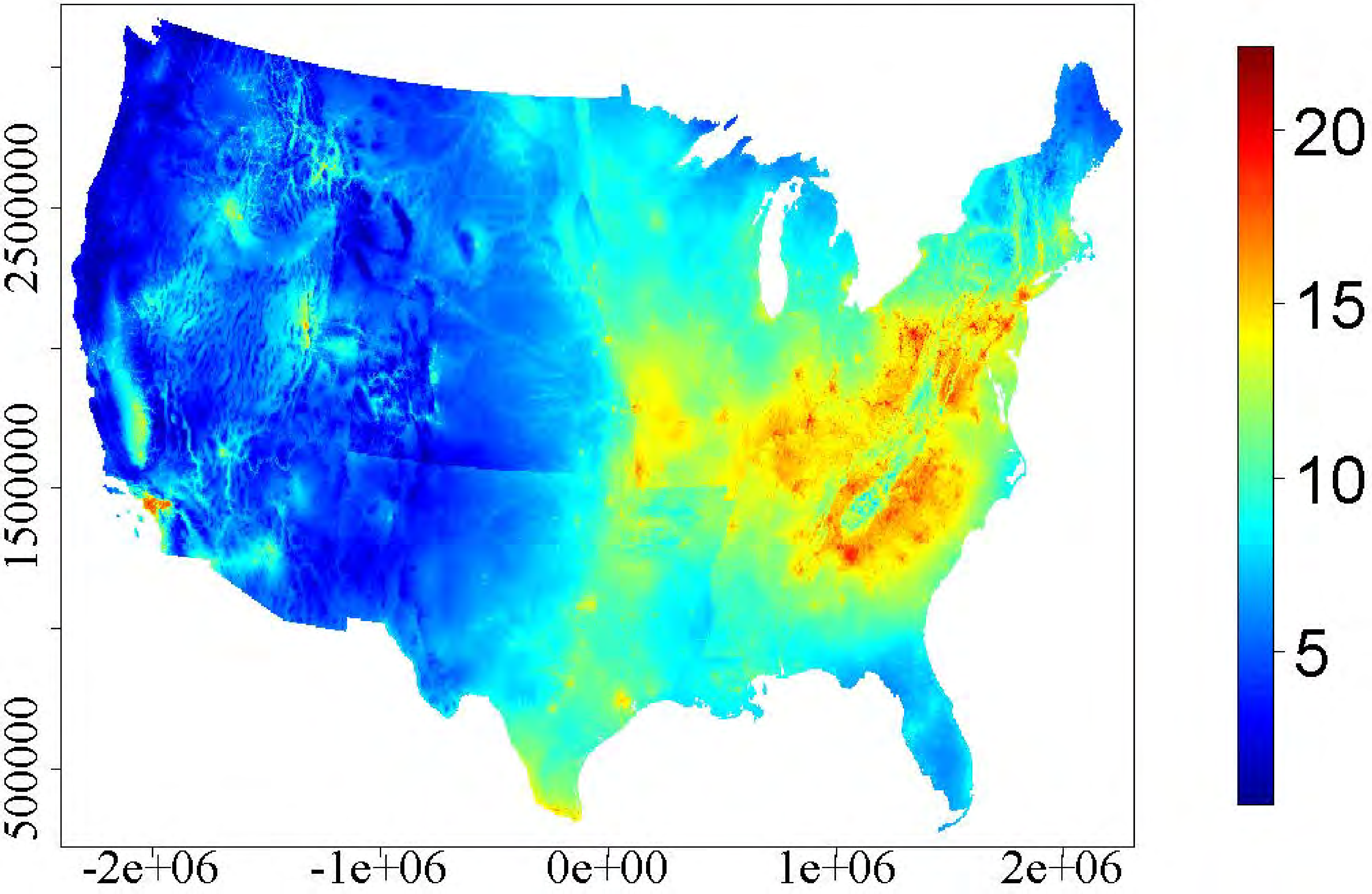

July 2007

Monthly predicted $PM_{2.5}$ ($\mu g\ m^{-3}$)

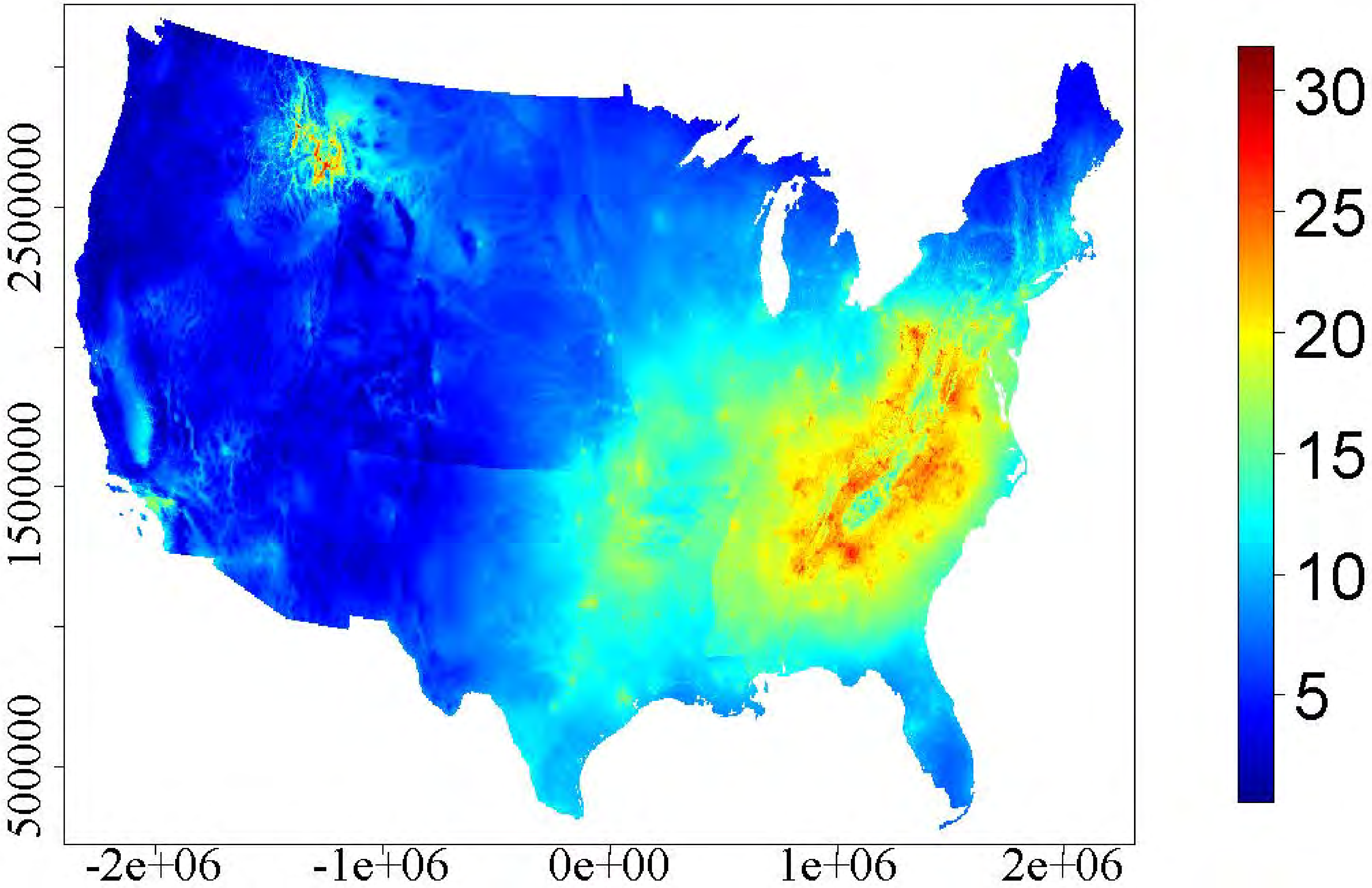

August 2007

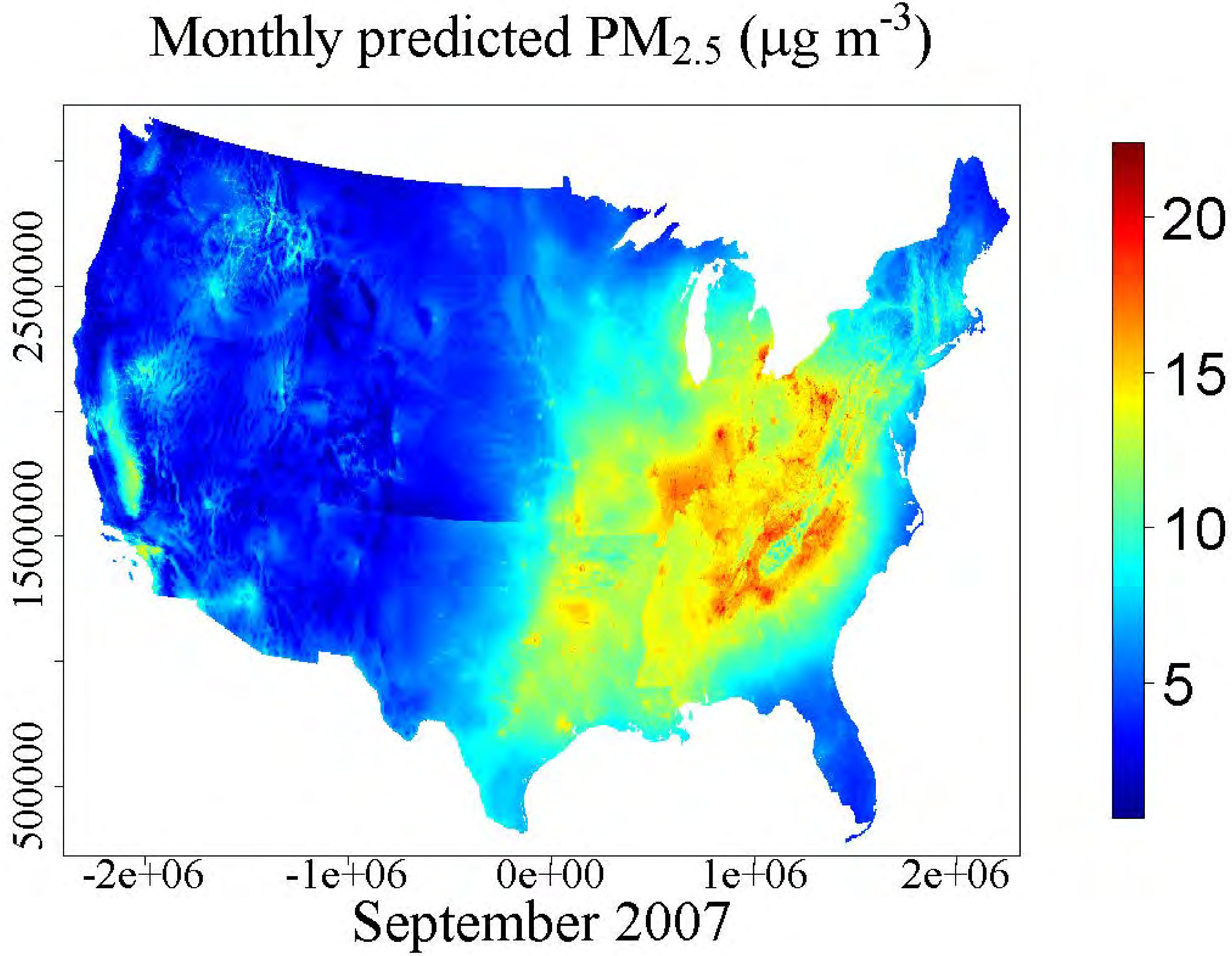

Monthly predicted $PM_{2.5}$ ($\mu g\ m^{-3}$)
25000000
15000000
5000000
-2e+06
-1e+06
0e+00
1e+06
2e+06
September 2007
20
15
10
5

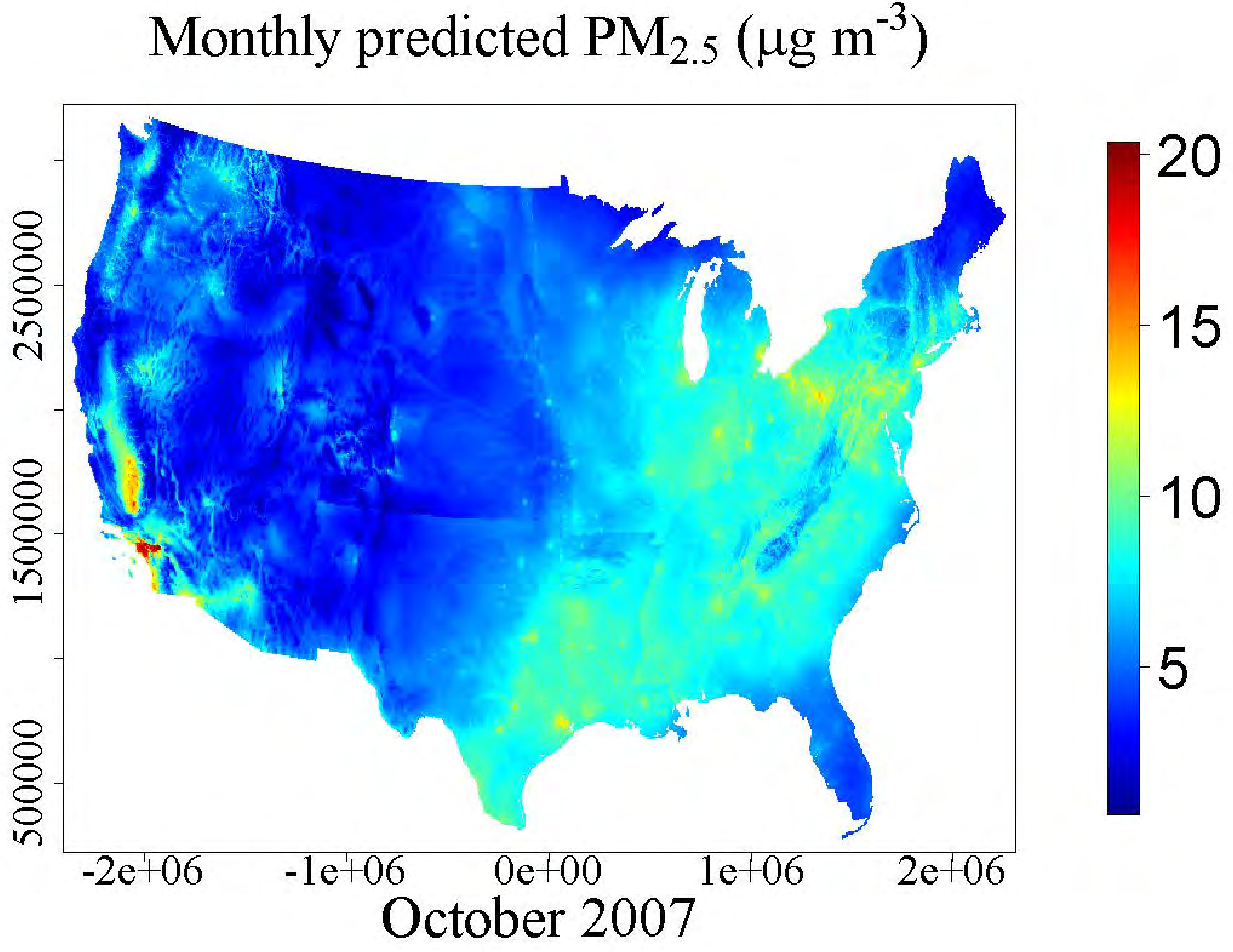
Monthly predicted PM2.5 (µg m-3)
25000000
15000000
5000000
-2e+06
-1e+06
0e+00
1e+06
2e+06
20
15
10
5
October 2007

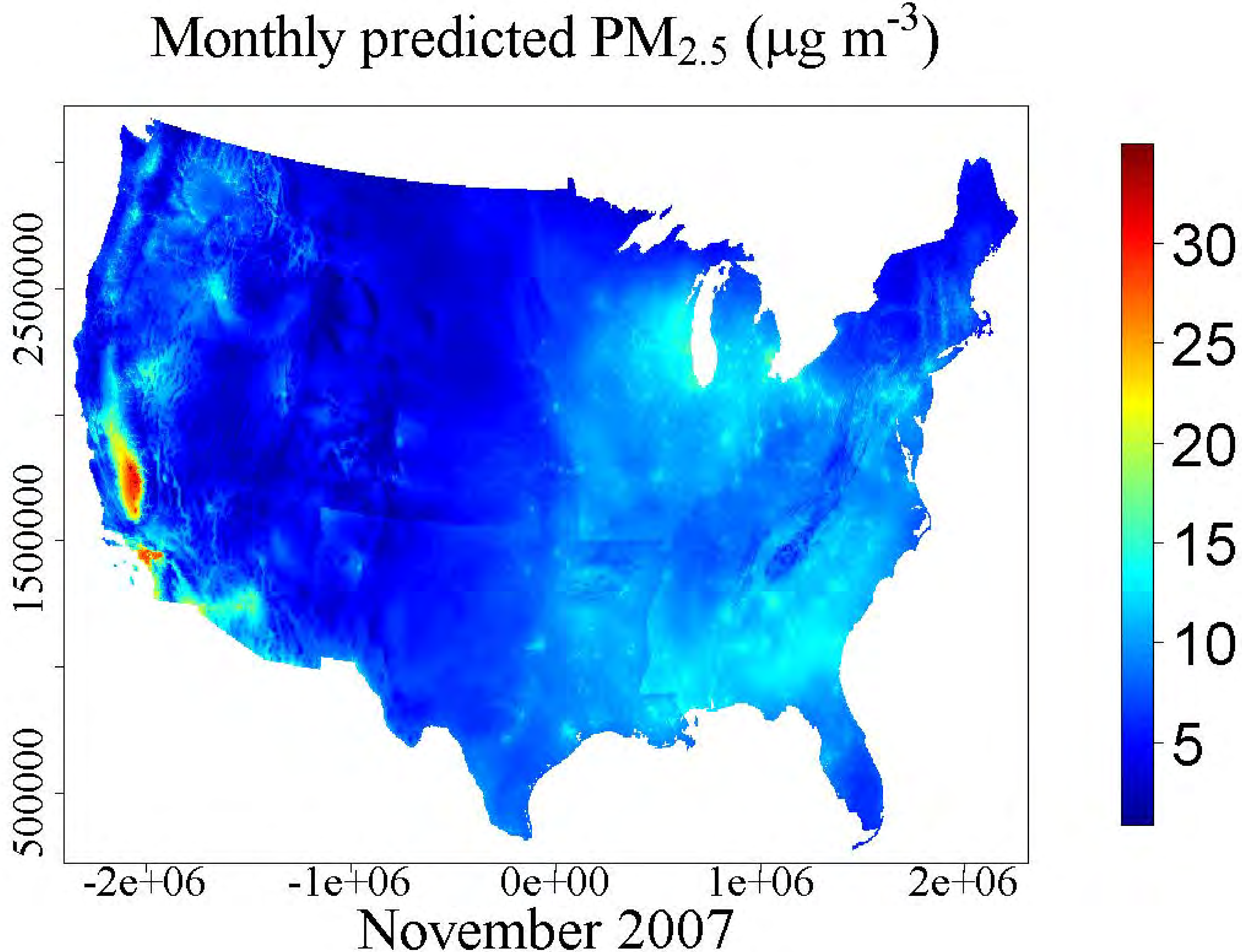
Monthly predicted PM$_{2.5}$ (μg m^{-3})
25000000
15000000
5000000
-2e+06
-1e+06
0e+00
1e+06
2e+06
November 2007
30
25
20
15
10
5

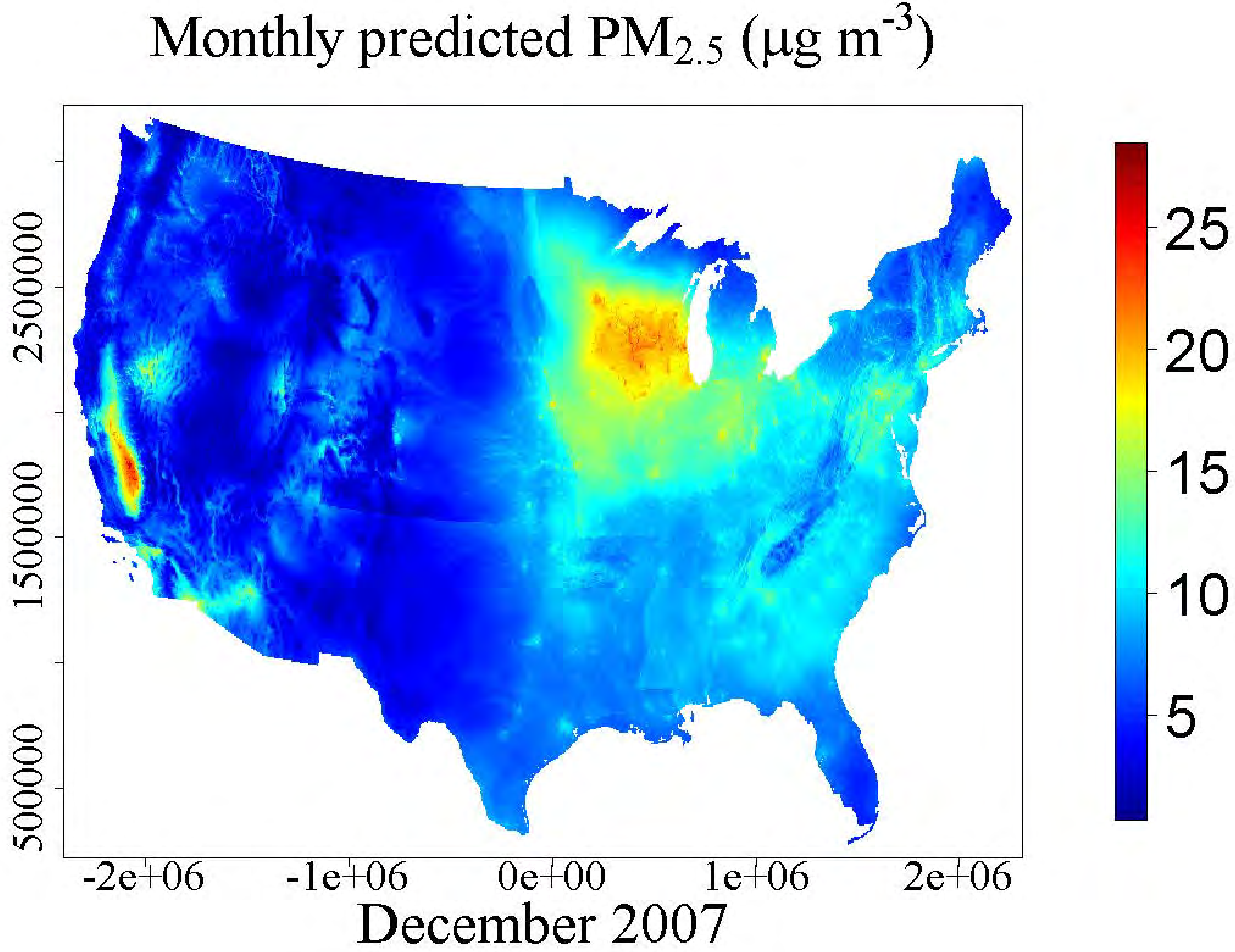
Monthly predicted PM2.5 (μg m-3)
25000000
15000000
5000000
-2e+06
-1e+06
0e+00
1e+06
2e+06
25
20
15
10
5
December 2007
